# Unlocking the Silent Proteome: Chemoselective Asn/Gln Activation for Multidimensional Protein Diversification

**DOI:** 10.1021/jacs.5c22184

**Published:** 2026-03-13

**Authors:** Benjamin Emenike, Zachary E. Paikin, John M. Talbott, Anna Lidskog, Bao Quang Gia Le, Jagannath Swaminathan, Eric V. Anslyn, Monika Raj

**Affiliations:** † Department of Chemistry, 1371 Emory University, Atlanta, Georgia 30322, United States; ‡ Department of Chemistry, 118710 University of Texas at Austin, Austin, Texas 78712, United States

## Abstract

Amides are ubiquitous in pharmaceuticals, natural products, and biomolecules, owing to their exceptional stability and hydrogen-bonding capacity. Among the amino acids, asparagine (Asn) and glutamine (Gln) contain neutral primary amide side chains and constitute over 8% of the human proteome. Despite their abundance, these residues have remained largely inaccessible to selective chemical modification due to their low intrinsic reactivity and the propensity of proteinogenic side chains to poison transition-metal catalysts via chelation. Here, we report a general strategy that converts the primary amides of Asn and Gln into bioorthogonal nitrile handles, which can be further diversified through carbometalation with aryl boronic acids to yield aryl ketone products. This transformation proceeds with exceptional chemoselectivity, enabling the modification of native peptides and proteins. We demonstrate its broad utility in the synthesis of unnatural amino acids, late-stage diversification of peptides, fluorosequencing of Asn residues, and site-selective protein modification, culminating in the synthesis of a functional antibody-fluorophore conjugate. The versatility and selectivity of this approach expand the accessible chemical space of biomolecules and provide a powerful route for uncovering previously uncharacterized Asn/Gln sites within the chemically silent proteome.

## Graphical abstract

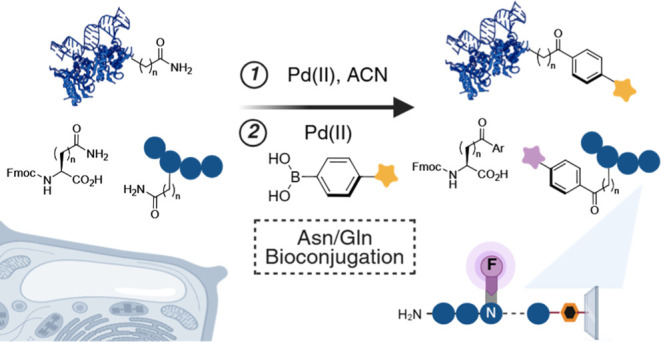

## Introduction

Amides, although neutral, play a significant role in biomolecular interactions and catalyze vital chemical and biochemical reactions through the formation of multiple hydrogen bonds (up to 3 per primary amide).^1,2^ Within the proteome, asparagine (Asn) and glutamine (Gln) together account for approximately 8.1% of all residues, a prevalence comparable to that of serine, the most abundant amino acid. Despite their ubiquity,^3–5^ primary amides have remained unexplored targets for selective modification, owing to their low intrinsic reactivity relative to other native functional groups such as thiols (Cys), amines (Lys), thioethers (Met), phenols (Tyr), imidazoles (His), carboxylate (Asp/Glu), and guanidiniums (Arg) (Figure 1a,b).^6–13^ The challenge is compounded by the difficulty of discriminating these side chains from the numerous secondary amides present within the peptide backbone.^14^

**Figure 1:**
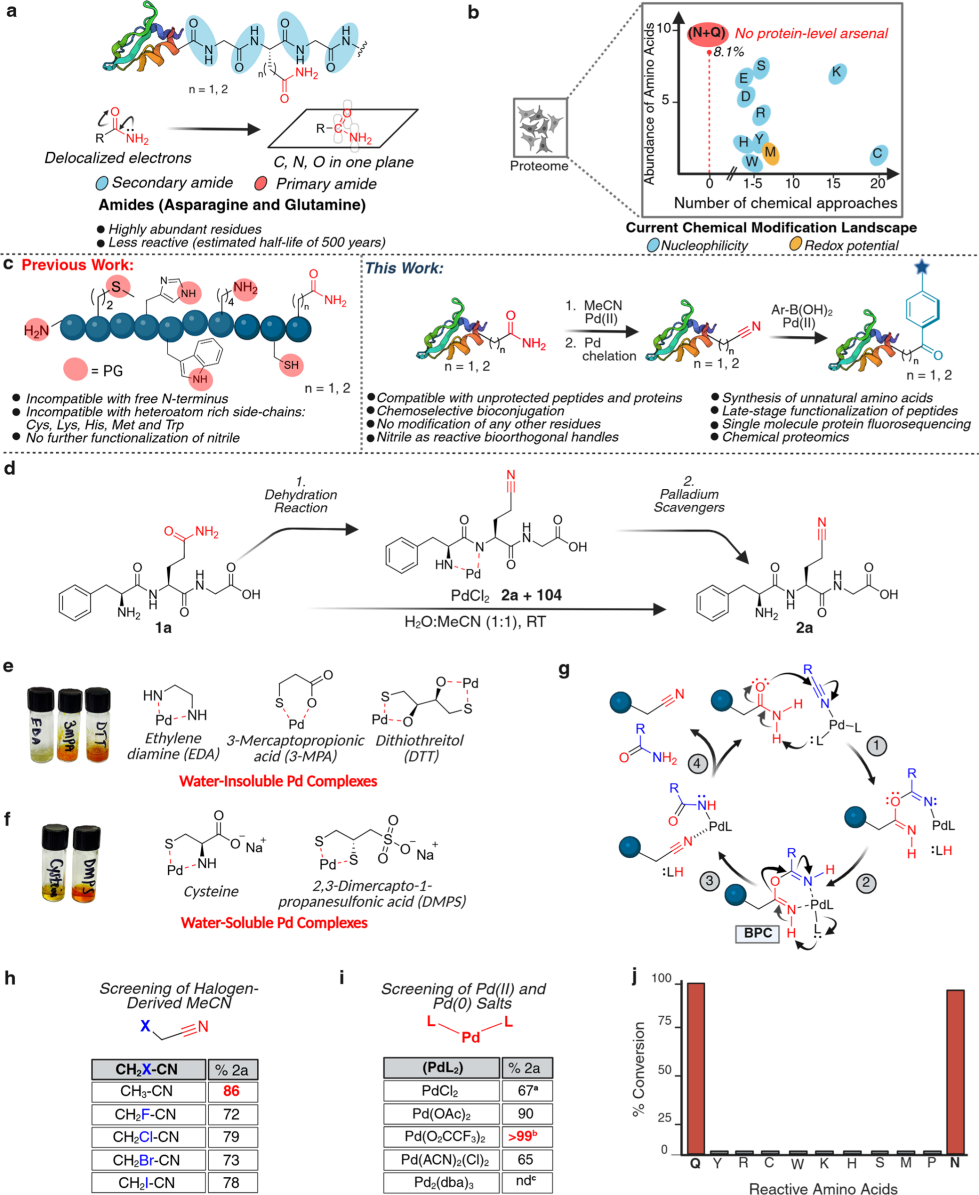
Dehydration reaction for chemoselective modification of asparagine and glutamine to bioorthogonal nitrile handles. (a) Depiction of the high stability and low reactivity of amides. (b) Amino acid abundance and existing chemical methods for selective modification of nucleophilic amino acids, such as tyrosine, arginine, cysteine, tryptophan, lysine, histidine, and redox-sensitive residues such as methionine. Primary amide-containing asparagine and glutamine, constituting approximately 8.1% of the proteome, are largely unreactive, and to date, no unbiased chemical modification strategy has been developed to target them selectively. (c) Previous studies demonstrating Pd-mediated dehydration of primary amide to nitrile suffer from poor functional group and heteroatom compatibility. In this work, we develop Pd chelation strategies to transform the reaction into a protein-compatible bioconjugation tool, with initial dehydration of asparagine and glutamine to form bioorthogonal nitriles and further modification by a carbometalation reaction with boronic acids. (d) Dehydration reaction with a model peptide FQG **1a** using PdCl_2_ in 1:1 H_2_O/MeCN to yield nitrile product FQ­(CN)­G **2a**. (e) Recovery of palladium-bound peptide **2a** via palladium scavengers such as EDA, 3-MPA, or DTT to generate water-insoluble palladium complexes. (f) Recovery of palladium via palladium scavengers such as cysteine and DMPS generates water-soluble palladium complexes that are suitable for downstream protein applications. (g) Proposed catalytic cycle for isohypsic dehydration starts with palladium coordination to MeCN followed by attack of amide nitrogen (steps 1 and 2), dehydration, and release of nitrile and acetoamide products (steps 3 and 4). (h) Screening of Pd (II) and Pd (0) salts (1 equiv.) in converting **1a** to **2a** in a 1:1 solution of H_2_O/MeCN for 8 h. ^a^note: = 67% conversion (reaction time 8 h), 94% conversion (reaction time 24 h); ^b^ = reaction complete in 2 h, ^c^= (nd) not determined due to insolubility of Pd_2_(dba)_3_. (i) Screening of nitriles containing electron-withdrawing groups (F, Cl, Br, I), with Pd­(O_2_CCF_3_)_2_ (1 equiv.) and MeCN for 30 min. (j) Chemoselectivity studies of the isohypsic reaction with varying reactive amino acids under the optimized conditions. Only Asn and Gln generated nitriles without modification of any other amino acids. Created in BioRender. Lab, R. (2026) https://BioRender.com/7tp0sm8.

While palladium-mediated dehydration of primary amides has been reported in synthetic contexts,^15–18^ these methods possess fundamental limitations that have precluded their translation to biological systems. Specifically, previous protocols are restricted to small, protected substrates because the reagents are inherently incompatible with the complex chemical landscape of proteins. In native biomolecules, the abundance of heteroatom-rich side chains (e.g., histidine, lysine, cysteine, and methionine) and free N-termini act as thermodynamic sinks, rapidly coordinating Pd­(II) to form stable, nonreactive chelates that poison the catalyst and prevent nitrile formation (previous work, Figure 1c). Furthermore, prior studies established the nitrile solely as a synthetic end point, failing to exploit its potential as a reactive handle for downstream diversification. Consequently, despite the ubiquity of Asn and Gln, there remains no general strategy to unlock these “silent” residues for bioconjugation. In stark contrast, we have reimagined this transformation from a limited synthetic reaction into a robust, biocompatible platform (This work, Figure 1c). By engineering a sequence that couples kinetically controlled dehydration with effective metal scavenging, we overcome the chelation barrier, enabling the first chemoselective modification of native Asn and Gln residues in the presence of all unprotected proteinogenic functionalities. We further advance this chemistry beyond simple nitrile formation through Pd-mediated carbometalation, generating bioorthogonal labeling. This methodology effectively unlocks the ∼8% of the proteome previously inaccessible to chemical probing, providing a powerful new multidimensional tool for protein engineering, fluorosequencing, and chemical proteomics.

We demonstrate the versatility of this method in multiple contexts. First, it enables the synthesis of unnatural amino acids and the late-stage diversification of native, fully unprotected peptides through the coupling of Asn/Gln-modified nitrile with structurally varied aryl boronic acids, producing aryl-ketone-containing peptides with distinct chemical and pharmacological profiles.^2^

This late-stage, handle-free strategy obviates the need for de novo analogue synthesis, streamlining the generation of SAR libraries and accelerating peptide and protein lead optimization.^20–31^

To showcase its translational potential, we applied this Asn/Gln modification pathway to a cytotoxic peptide,^32^ incorporating a range of aryl boronic acids at Asn/Gln sites to create a chemically diverse aryl-ketone library with tunable cytotoxicity. These results underscore the method’s value in modulating bioactivity through the selective introduction of new functional groups onto Asn/Gln residues.^33^ Furthermore, by coupling fluorophores to modified sites, we established the reaction’s compatibility with fluorosequencing, enabling single-residue detection of Asn at a single molecule level. Finally, we extended this workflow to protein modification, selectively introducing ketone handles onto Asn/Gln nitriles across proteins of varying sizes and complexity. Using this strategy, we synthesized a functional antibody-fluorophore conjugate, demonstrating the method’s utility for site-specific modification of complex biomolecules while retaining biological activity. Taken together, this chemistry transforms Asn/Gln from largely “silent” residues into programmable entry points for late-stage functionalization, complementing existing Cys, Lys, and Met bioconjugation platforms with the first general strategy to exploit neutral primary amide side chains in native biomolecules.

## Results and Discussion

### Development of the Dehydration Reaction for Selective Modification of Asparagine and Glutamine

We began by exploring isohypsic dehydration^15–18^ as a strategy for selectively converting the side-chain amides of asparagine and glutamine into bioorthogonal nitrile handles, seeking to transform the reaction from a synthetic tool with limited functional-group compatibility into the first step of a robust bioconjugation workflow. Our initial reaction employed the model tripeptide FQG **1a**, treated with PdCl_2_ (10 mol %) in a 1:1 mixture of H_2_O/acetonitrile (MeCN) (Figures 1d and S1a). As expected, the reaction afforded the desired peptide nitrile FQ­(CN)­G **2a** in only 52% conversion after 24 h, as determined by high-performance liquid chromatography (HPLC) and mass spectrometry (MS). The observed +104 Da mass shift of both the starting material and product suggested palladium complexation with the free N-terminal amine. To test this, we synthesized N-terminally acetylated Ac-FQG **1b**, which cleanly produced Ac-FQ­(CN)­G **2b** in 86% conversion without Pd adduct formation (Figure S1a).

To dissociate these Pd-peptide complexes and allow this reaction to be broadly applicable, we evaluated several palladium scavengers. Treatment with ethylenediamine (EDA), 3-mercaptopropionic acid (3-MPA), or dithiothreitol (DTT) quantitatively removed Pd, forming water-insoluble palladium complexes and liberating the nitrile product FQG-CN **2a** within 5 min (Figures 1e and S1b). Crucially, treatment with cysteine or 2,3-dimercapto-1-propanesulfonic acid (DMPS) sequestered Pd into highly water-soluble thiolate complexes. This serves a dual purpose: it rapidly quenches the reaction to prevent nonspecific metal coordination and enables efficient removal of palladium during downstream filtration or dialysis, consistent with prior reports that thiol-based scavengers can reduce residual Pd in peptide and protein products to below ICP–MS detection limits under similar conditions^34–36^ (Figures 1f and S1b).

The modest conversion in the initial reaction with **1a** was attributed to Pd sequestration by the peptide backbone and the N-terminus. Increasing the PdCl_2_ loading to 1 equiv., followed by Pd quenching, overcame this limitation, affording 94% conversion to the desired nitrile FQ­(CN)­G **2a** after 24 h. The MS analysis of the N-terminal fragments confirmed that the N-terminus acts solely as a transient ligand for palladium; no permanent chemical modification of the N-terminal amine was observed following Pd removal (Figure S1b). The mechanism is proposed via coordination between Pd­(II) and MeCN, forming a bivalent Pd complex (BPC) that mediates amide dehydration to generate nitrile and acetoamide byproducts^15–18^ (Figure 1g). In essence, the mechanism is a reversible metathesis reaction of the primary amides of asparagine and glutamine with acetonitrile, forming nitriles at these residues and acetamide. To further optimize conditions, we screened a series of Pd­(II) and Pd(0) salts (Figures 1h and S1b). Pd­(OAc)_2_ and Pd­(O_2_CCF_3_)_2_ (1 equiv. each) produced 90% and >99% conversion, respectively, within 8 h under identical aqueous MeCN conditions. Neither Pd­(MeCN)_2_Cl_2_ nor the Pd(0) complex Pd_2_(dba)_3_ offered improvements, with the latter showing no reactivity due to poor solubility.

We next investigated the influence of EWG nitrile solvents, including CH_2_FCN, CH_2_ClCN, CH_2_BrCN, and CH_2_ICN, but none improved the reaction efficiency compared to MeCN, instead yielding multiple side products. These byproducts likely arose from nucleophilic attack of the peptide N-terminus on electrophilic halogenated nitriles (Figures 1i and S1b). To confirm product identity by nuclear magnetic resonance (NMR), the reaction was performed on a model small-molecule 3-phenylpropanamide **1c**, which was quantitatively converted to 3-phenylpropionitrile **2c** (98% yield) using 10 mol % Pd­(O_2_CCF_3_)_2_ in 1:1 H_2_O/MeCN at room temperature for 16 h (Figure S1c).

The optimized system using MeCN as the dehydration medium, Pd­(O_2_CCF_3_)_2_ as a mediator, and 3-MPA as a Pd scavenger displayed remarkable chemoselectivity toward Gln and Asn while leaving other reactive residues (Tyr, Arg, Cys, Trp, Lys, His, Ser, Met, and Pro) unmodified under the same conditions using tripeptides **1d–1l** (Figures 1j and S2). Notably, the Asn-containing peptide FNG **1m** underwent quantitative conversion (>95%) to its nitrile analogue FN­(CN)­G **2m**, demonstrating the general applicability of this dehydration method to primary amides. Furthermore, the peptide nitrile FQ­(CN)­G **2a** exhibited exceptional stability under harsh conditions, supporting its suitability for selective Asn/Gln modification in complex biological systems (Figure S3). Collectively, these results establish isohypsic dehydration as a robust and chemoselective route for transforming Asn and Gln residues into bioorthogonal nitriles directly on native peptides without perturbing any other side chain. To the best of our knowledge, this represents the first general platform for selectively activating primary amide side chains in complex peptide environments.

### Exploration of Asn/Gln Nitrile Modification Methods

Having optimized the conditions for converting primary amides to nitriles, we next aimed to develop efficient strategies to diversify this electrophilic nitrile handle. To identify a suitable nitrile modification strategy, we first examined the subtle differences in reactivity among Asn-, Gln-, and C-terminal-derived nitriles. Using density functional theory (DFT) calculations, we compared the relative LUMO energies of model compounds as indicators of electrophilicity. The LUMO energy of H_2_N-Gln­(CN)-CO_2_H was calculated to be −40.67 kJ/mol, whereas H_2_N-Asn­(CN)-CO_2_H and the C-terminal nitrile H_2_N-Asp-(CN) exhibited lower values of −55.69 kJ/mol and −67.92 kJ/mol, respectively (Figures 2a and S4). These data indicate that the Gln nitrile is the least electrophilic, whereas the C-terminal nitrile is the most reactive, likely due to inductive effects from the adjacent peptide backbone.

**Figure 2:**
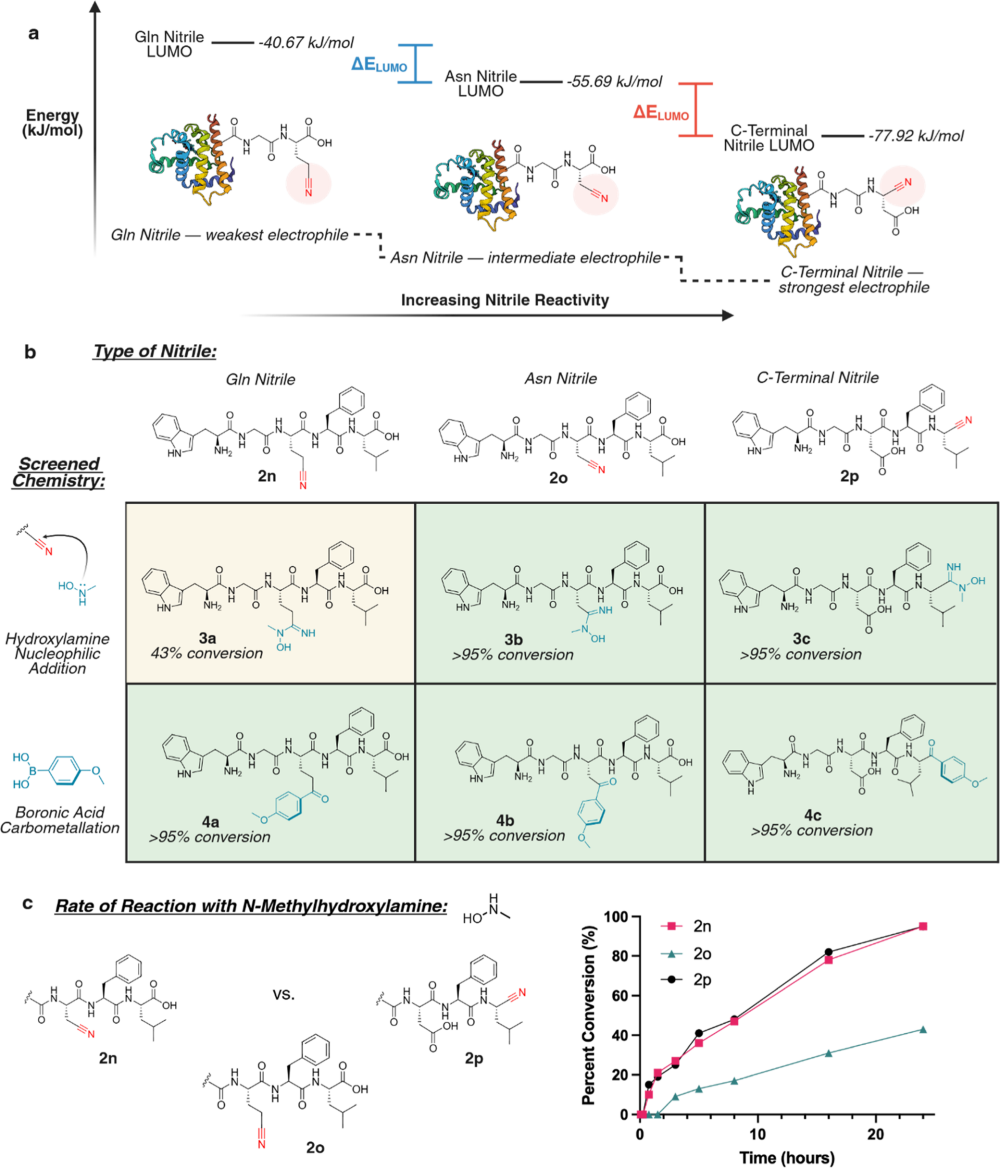
Reactivity profiling of Asn/Gln-derived nitriles. (a) Evaluation of nitrile electrophilicity by DFT calculations showing relative LUMO energies for model compounds: H_2_N-Gln­(CN)-CO_2_H, H_2_N-Asn­(CN)-CO_2_H, and the C-terminal nitrile H_2_N-Asp-(CN). This electrophilicity trend was observed with a large variety of peptide sequences. (b) Synthesis of nitrile-containing peptide analogues WGQ­(CN)­FL-CO_2_H **2n**, WGN­(CN)­FL-CO_2_H **2o**, and WGDFL-(CN) **2p**, followed by assessment of the reactivity toward *N*-methylhydroxylamine. While Asn and C-terminal nitriles reacted with >95% conversion (**3b** and **3c**), the Gln nitrile displayed slower conversion to **3a** (43% after 24 h). In contrast, Pd-mediated carbometalation with boronic acids efficiently converted all three nitriles to the corresponding ketone peptides **4a–4c**. (c) Kinetic analysis of hydroxylamine addition to nitrile peptides **2n**–**2p** showed comparable reaction rates for Asn and C-terminal nitriles, with Gln nitrile reacting more slowly. Created in BioRender. Lab, R. (2026) https://BioRender.com/02bn0rx.

To experimentally explore these predictions, we prepared three model peptide analogues WGQFL-CO_2_H **1n**, WGNFL-CO_2_H **1o**, and WGDFL-CONH_2_ **1p** and converted them to the corresponding nitriles **2n**–**2p** on a 20 mg scale using 2 equiv. Pd­(O_2_CCF_3_)_2_, achieving >95% conversion in all cases (Figure S5a). We first explored nucleophilic addition to the nitrile using *N*-methylhydroxylamine (10 equiv.) in PBS (pH 7.4)/ EtOH (1:1) at 40 °C for 24 h. The Gln nitrile (**2n**), being less electrophilic, exhibited only 43% conversion to the hydroxylamine adduct **3a** after 24 h (Figures 2b and S5b). In contrast, the more reactive Asn nitrile (**2o**) and C-terminal nitrile (**2p**) underwent near-quantitative conversion to the corresponding hydroxylamine adducts **3b** and **3c**, respectively. Kinetic analysis further supported this trend: after 8 h, the Asn and C-terminal nitriles achieved nearly 50% conversion, whereas the Gln nitrile showed only 17% conversion (Figures 2c and S5b).

Aiming to improve the reaction efficiency and enable Gln modification, we used 3-phenylpropionitrile **2c** as a model substrate and tested various Cu­(I)/Cu­(II) salts and a diamine ligand, which failed to enhance the reactivity. Additionally, O-substituted hydroxylamines were unreactive under all tested conditions (Figure S5b). Many other strong, aqueous- compatible nucleophiles were screened in various conditions, but were unreactive with the nitriles. These findings underscore the limited substrate scope and low efficiency of hydroxylamine addition to the nitrile obtained from Gln. Other strong nucleophiles provided trace products. Given the sluggish reactivity of Gln nitriles, we turned to a Pd-mediated carbometalation strategy between nitriles and aryl boronic acids, previously explored for nitrile modification on simple small molecule systems.^19,37,38^ Remarkably, using 4-methoxyphenylboronic acid (6 equiv.), Pd­(OAc)_2_ (20 mol %), bipyridine (20 mol %), and TFA (10 equiv.) in H_2_O/THF (1:1) at 60 °C for 12 h, we achieved >95% conversion of all three nitrile peptides (Asn, Gln, and C-terminal) to the corresponding aryl ketone products **4a-4c** (Figures 2b and S5c). Encouraged by this broad reactivity and the ability to transform Asn/Gln residues into bioorthogonal ketone handles, we pursued this Pd-mediated carbometalation as a general platform for dual functionalization of peptides and proteins through diverse boronic acid coupling partners.

### Diversification of Small Molecule Nitrile by Carbometalation with Boronic Acid

The remarkable efficiency of Pd-mediated carbometalation for Asn- and Gln-derived nitriles prompted us to explore its full synthetic potential on simpler molecular systems. To better understand the reaction scope, electronic effects, and reaction conditions of this transformation, we turned to a model small molecule, nitrile **2c**. Using phenylboronic acid as a coupling partner, we optimized conditions to convert **2c** into the corresponding aryl ketone **4d**. Our optimization efforts gave an 85% isolated yield of aryl ketone **4d** by employing Pd­(OAc)_2_ (10 mol %), bipyridine (bpy) (20 mol %), and trifluoroacetic acid (TFA) (10 equiv.), in H_2_O/THF (1:5) at 40 °C within 8 h (Figure S5).^37^ Encouraged by this efficiency, we next examined the substrate scope with a variety of aryl- and heteroaryl boronic acids bearing both electron-donating (EDG) and EWG substituents. Reactions with EDG-containing boronic acids such as 4-methoxy, 4-hydroxyl, 1,4-benzodioxane, and 4-diphenylamine proceeded smoothly at 40 °C to furnish aryl ketones **4e**–**4h** in 73–89% isolated yields (Figures 3a and S6). In contrast, EWG-substituted boronic acids, including 4-fluoro, 3-sulfonamide, 4-acetyl, 2-trifluoromethyl, and 3-nitro, required higher temperatures (80 °C) and longer reaction times (24–48 h) to afford products **4i**–**4m** in moderate to high isolated yields (47–92%). Mechanistically, the superior reactivity of EDG-substituted substrates likely arises from faster transmetalation of the arylboronic acid to cationic Pd­(II) and more favorable intramolecular carbometalation.^37^ This kinetic advantage of electron-rich substrates guided our selection of boronic acids for protein functionalization (vide infra), where mild temperatures (40–60 °C) are preferred over the elevated conditions (80 °C) required for highly electron-deficient partners. Heteroaryl boronic acids such as thiophene, furan, and indolyl also coupled efficiently with nitrile **2c** to yield aryl ketones **4n**–**4p** in 52–86% isolated yields under mild conditions, although indolylboronic acid required 80 °C for full conversion. Boronic acids bearing unique aromatic scaffolds (fluorenyl) and thioether substituents (4-butylthio) also coupled smoothly to furnish aryl ketones **4q** and **4r** in 65–68% isolated yields. Notably, alkyl and vinyl boronic acids failed to generate any detectable products under the optimized conditions (Figure S7). All aryl ketone products (**4d**–**4r**) were characterized by ^1^H and ^13^C NMR and HRMS (Figure S6).

**Figure 3:**
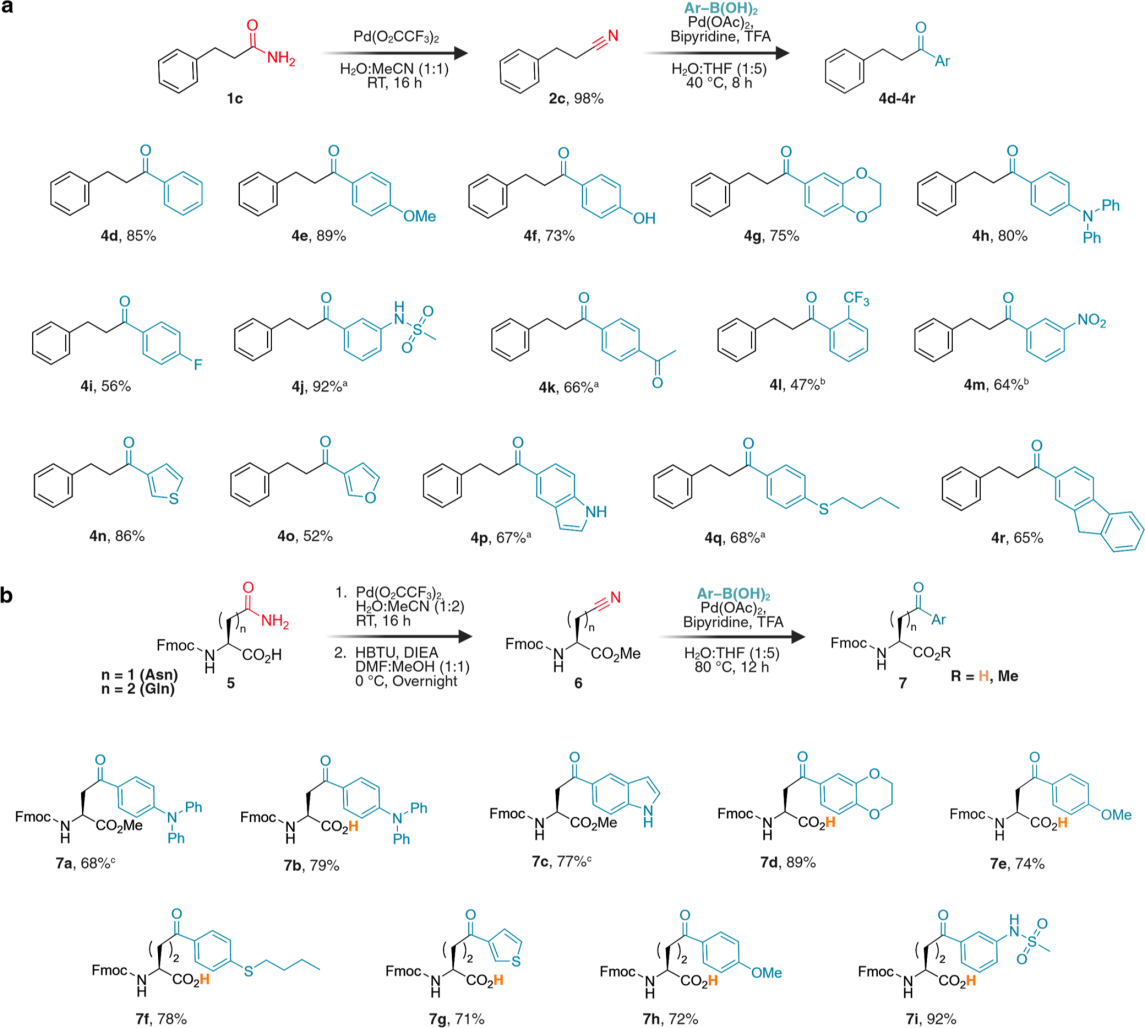
Substrate scope of the carbometalation reaction for synthesis of diverse small molecules and unnatural Fmoc-aryl ketone amino acids from nitriles. (a) Generation of small molecule nitrile **2c** and scope of the boronic acid carbometalation reaction with various aryl boronic acids, including heteroaryl and boronic acids containing EDG and EWG. Yield values provided refer specifically to carbometalation reaction with nitrile **2c**. Nitrile **2c** (0.4 mmol, 1 equiv.) and boronic acid (1.6 mmol, 4 equiv.) were heated at 40 °C for 8 h with Pd­(OAc)_2_ (10 mol %), bpy (20 mol %), and TFA (10 equiv.) under a N_2_ atmosphere in 1:5 H_2_O/THF (2.4 mL total). ^*a*^24 h of reaction time at 80 °C. ^*b*^Pd­(OAc)_2_ (20 mol %), bpy (40 mol %) with a 48 h reaction time at 80 °C. (b) Generation of Fmoc-aryl ketone amino acids through nitrile formation on Fmoc-Asn/Gln, followed by rapid diversification with assorted boronic acids. Yields refer specifically to the reaction of the Asn/Gln­(CN) ester **6** intermediate with aryl boronic acids. Asn/Gln­(CN) ester **6** (0.3 mmol, 1 equiv.) and boronic acid (1.2 mmol, 4 equiv.) were heated at 80 °C for 12 h with Pd­(OAc)_2_ (10 mol %), bpy (20 mol %), and TFA (10 equiv.) under a N_2_ atmosphere in 1:5 H_2_O/THF (3 mL total). ^c^3 h reaction time at 80 °C. Created in BioRender. Lab, R. (2026) https://BioRender.com/afe4uxk.

### Synthesis of Nitrile and Ketone-Functionalized Unnatural Amino Acids

The high substrate scope of the reaction of nitriles with varying boronic acids and the formation of ketones for further functionalization make this approach ideal for the synthesis of unnatural amino acids with diverse pharmacological properties. To this end, we applied this reaction for the synthesis of unnatural Fmoc-amino acids. Beginning with native Fmoc-Asn/Gln-CO_2_H (**5a**–**5b**), we converted them into Fmoc-Asn/Gln­(CN)–CO_2_Me **6a** and **6b** via dehydration using Pd­(O_2_CCF_3_)_2_ and MeCN/H_2_O (2:1), followed by esterification using HBTU and DIEA in MeOH/DMF (1:1) (Figures 3b and S8). Esterification of the nitrile amino acids limits Pd chelation during the subsequent carbometalation reaction, increasing the efficiency of product formation.

With Fmoc-nitriles **6a** and **6b** as starting materials, we synthesized various unnatural Fmoc-aryl ketone amino acids and esters **7a**–**7i**, by utilizing diverse aryl boronic acids such as 4-diphenylamine, indolyl, 1,4-benzodioxane, 4-butylthio, thiophene, 4-methoxy, and 3-sulfonamide (Figures 3b and S9a). The synthesis furnished Fmoc-aryl ketone amino acids and esters **7a**–**7i** in high isolated yields (68–92%), irrespective of the nature of boronic acids and the employed nitriles, Fmoc-glutamine and Fmoc-asparagine nitrile **6a** and **6b**. Notably, the acidic reaction conditions and higher temperatures used in this transformation can hydrolyze the C-terminal ester to carboxylic acid. However, as ester hydrolysis proceeded slower than the carbometalation reaction, formation of aryl ketone products **7a** and **7c** were stopped after 3 h to furnish the ester product. A longer reaction time of 12 h produced a C-terminal carboxylic acid in all other substrates. We confirmed the formation of Fmoc-aryl ketone amino acids by ^1^H and ^13^C NMR (Figure S9a). To establish that the nitrile and aryl ketone Fmoc-Asn monomers are practical building blocks for peptide synthesis, we incorporated Fmoc-Asn­(CN)-CO_2_H **6a′** and Fmoc-Asn­(*p*-MeO-aryl ketone)-CO_2_H **7e** into the model sequence H_2_N-YKGXHRA-CONH_2_ (X = Asn­(CN) for **S1**; X = Asn­(*p*-MeO-aryl ketone) for **S2**) using standard Fmoc SPPS. Both monomers were fully compatible with routine coupling and Fmoc-removal conditions and furnished the desired peptides without observable complications (Figure S9b). These monomers enable position-specific installation of nitrile and aryl ketone side chains during SPPS, providing a general route to peptides bearing embedded electrophilic/diversification handles that are difficult to access by postsynthetic modification.

### Late-Stage Functionalization of Peptides through Primary Amide Dehydration and Carbometalation

Due to the remarkable diversification potential observed in the transformation of native Fmoc-Asn and Fmoc-Gln amino acids into unnatural counterparts via this two-step pathway for modification of the primary amide, we expanded its utility to the LSF of native peptides.

A crucial requirement for an effective LSF method applicable to native peptides is a high chemoselectivity and broad functional group tolerance. To evaluate the chemoselectivity of the carbometalation reaction toward Asn and Gln in the presence of other reactive amino acids, we conducted a screening experiment using the peptide KYWCSMEHR **S3** containing various reactive residues, such as Lys, Tyr, Trp, Cys, Ser, Met, Glu, His, and Arg. The peptide KYWCSMEHR was subjected to the optimized boronic acid cross-coupling conditions for 24 h. LCMS analysis revealed no conversion of any amino acid with the starting material fully intact (Figures 4a and S10).

**Figure 4:**
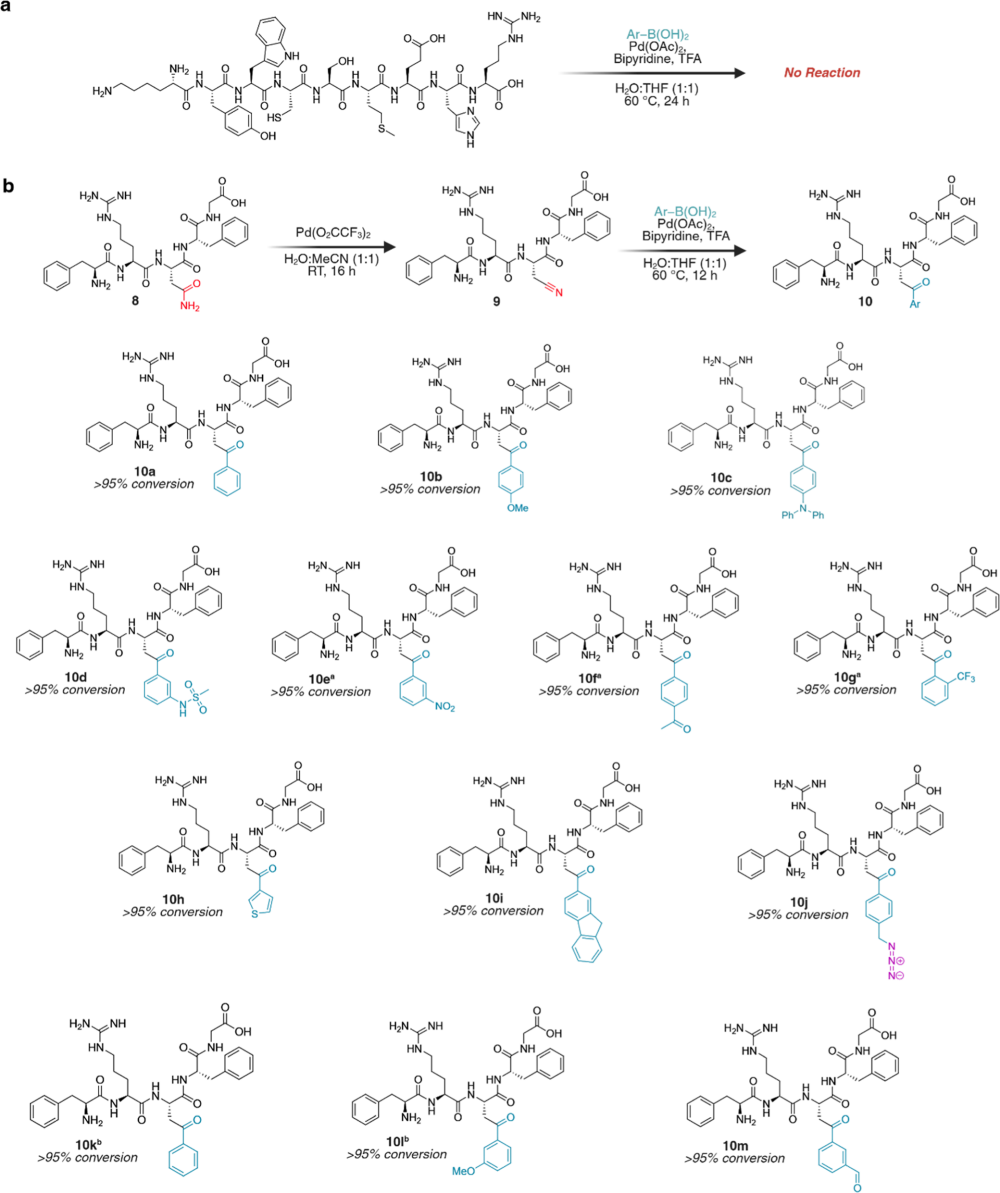
Chemoselectivity assessment and late-stage peptide diversification through dehydration of neutral amides to electrophilic nitriles and subsequent diversification by carbometalation. (a) Demonstration of the chemoselectivity of boronic acid cross-coupling using peptide, KYWCSMEHR, containing various reactive amino acids. No modification of the peptide was observed under our optimized conditions 24 h after treatment with 3-MPA for Pd chelation. (b) Scope of LSF of peptides using the Asn peptide FRNFG **8**. Initial bioorthogonal dehydration of the primary amide to electrophilic nitrile was followed by the facile generation of novel chemical space using various boronic acids. Nitrile peptide **9** (1.6 μmol, 1 equiv.) and boronic acid (9.6 μmol, 6 equiv.) were heated at 60 °C for 12 h with Pd­(OAc)_2_ (20 mol %), bpy (40 mol %), and TFA (10 equiv.) under a N_2_ atmosphere in 1:1 H_2_O/THF (300 μL total). ^a^Pd­(OAc)_2_ (50 mol %), bpy (1 equiv.) with a 48 h reaction time at 80 °C. ^b^ortho-deformylation products. Created in BioRender. Lab, R. (2026) https://BioRender.com/wi1pnwk.

Having established the chemoselectivity of both the Asn/Gln-to-nitrile conversion and the subsequent nitrile-to-ketone transformation, we next applied this strategy to LSF of a peptide FRNFG **8**. The peptide was first converted to its corresponding nitrile **9,** which was then subjected to carbometalation with a variety of boronic acids (Figures 4b and S11). Boronic acids containing EDG underwent smooth and quantitative transformation (>95%) to the corresponding ketones **10a**–**10d** under the optimized conditions, whereas boronic acids with EWG required slightly elevated temperatures (80 °C) and longer reaction times (48 h) to achieve quantitative conversions to **10e**–**10i** (Figures 4b and S11). Notably, peptide **10j** was synthesized using a boronic acid containing an azide functional handle, demonstrating the method’s ability to introduce affinity tags or bioconjugation sites (See Figure S12 for synthesis). We also explored formyl-functionalized boronic acids to afford ketone products (**10k–10m**)^38^ (Figures 4b and S13). Together, these results highlight an efficient and highly chemoselective LSF manifold that uniquely targets native Asn/Gln residues, enabling rapid access to peptide variants and functional handles that are difficult or impossible to obtain by de novo synthesis or existing residue-selective methods.

### Solid-Phase Synthesis of a Library of Aryl-Ketone Cytotoxic Peptides

Next, we applied this LSF strategy to improve the cytotoxicity of a peptide AERQ **11** containing Gln (EC_50_ = 4.9 mM, EC_90_ = 10 mM) toward HeLa cells.^31^ Encouraged by the remarkable robustness and selectivity of this bioconjugation reaction pathway for primary amides, we embarked on synthesizing this library on solid support, conducting both peptide-nitrilation and peptide-ketonylation on the peptide attached to the resin. In pursuit of this goal, we first synthesized AERQ **11** on solid support with unprotected Gln via Fmoc-Solid Phase Peptide Synthesis (Fmoc-SPPS). Next, we successfully executed the dehydration reaction, converting Gln to nitrilated peptide **12** on a solid support (Figures 5a and S14a). Subsequently, we conducted reactions with various aryl and heteroaryl boronic acids, incorporating EWG and EDGs, to yield a versatile library of peptide-aryl ketones **13a**–**13f**, as characterized by HPLC and MS (Figures 5a and S14a). Impressively, this potent reaction of primary amides to bioorthogonal nitriles and subsequently to bioorthogonal ketones occurred efficiently in high conversion on a solid support. Because Asn/Gln-containing sequences are common in functional peptides, we evaluated the compatibility of Asn with the on-resin workflow and assessed the risk of aspartimide formation. Asn­(Trt) was fully compatible with selective deprotection and subsequent on-resin diversification on Merrifield resin, enabling preparation of the corresponding amide peptide LFANFG **S4** and its conversion to the nitrile **S5** and aryl ketone **S6** derivatives (Figure S14b). Notably, liquid chromatography–mass spectrometry (LC–MS) analysis of the crude reaction mixtures for **S5** and **S6** showed no detectable levels of aspartimide-related side products under the standard conditions. To further address this concern , we previously used unprotected Asn for the conversion of WGNFL-CO_2_H **1o** to WGN­(CN)­FL-CO_2_H **2o** and FRNFG-CO_2_H **8** to FRN­(CN)­FG-CO_2_H **9**, which proceeded without observable aspartimide formation (Figures 2, 3, S5a, and S11), indicating that aspartimide is not a concern for this platform. Moreover, subsequent treatment of **2o** with *N*-methylhydroxylamine (24 h, 40 °C) likewise did not generate aspartimide (Figures 2 and S5a), demonstrating that the nitrile-containing peptides do not undergo aspartimide formation even under harsh, challenging conditions, underscoring the robustness of the nitrile-containing products under downstream functionalization conditions.

**Figure 5:**
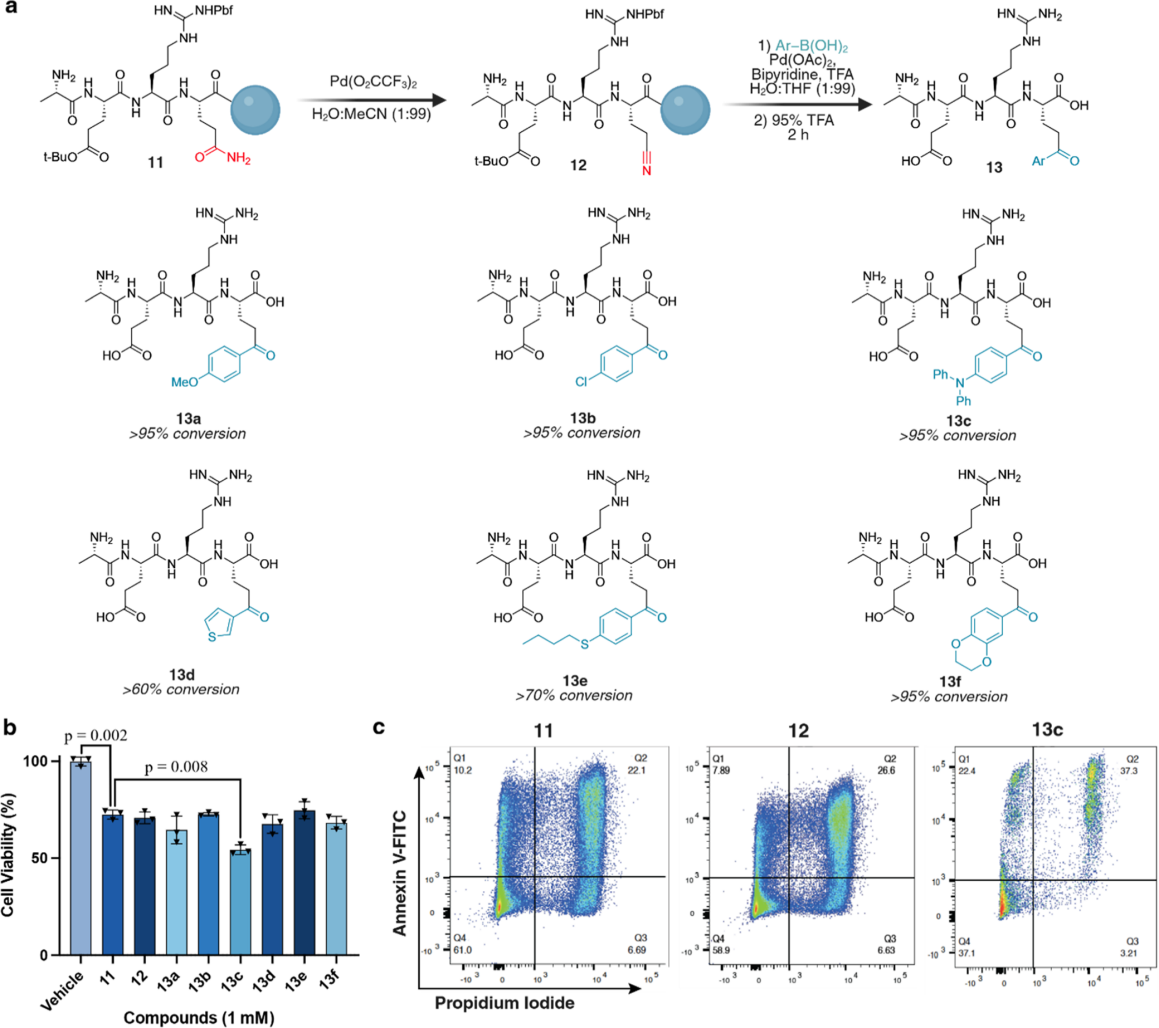
Nitrile formation and boronic acid carbometalation on solid support for synthesis of a cytotoxic peptide library. (a) Converting primary amides to nitriles to ketones on the solid phase, using cytotoxic peptide sequence AERQ **11**. This protocol was used to synthesize a diverse library of cytotoxic peptides with novel pharmacological properties through the carbometalation reaction with an array of boronic acids. Percent conversion refers to the formation of the ketone carbometalation product from an isolated nitrile peptide. (b) Evaluating the cytotoxicity of the peptide library. HeLa cells were incubated with 1 mM of each peptide for 48 h before AV/PI staining and cell viability quantification by flow cytometry. Triphenylamine analogue **13c** showed the greatest statistically significant increase in cell death. All data were normalized to 0% vehicle (DMSO) death. Statistical significance determined by two-sided Student’s *t*-test (*n* = 3). All experiments were performed in triplicate as biological replicates. Error bars represent mean ± standard deviation. (c) Representative flow cytometry graphs of **11,** **12**, and **13c** stained with Annexin V for apoptosis and propidium iodide for necrosis. Created in BioRender. Lab, R. (2026) https://BioRender.com/ew07yts.

With this diverse library of peptide-aryl ketone cytotoxic analogues **13a**–**13f** in hand, we conducted cytotoxicity assays by exposing HeLa cells to varying concentrations of these peptides, followed by analysis via flow cytometry (Figures 5b and S15). The data revealed a notable decrease in the cell viability of HeLa cells induced by peptide-aryl ketones, with the greatest increase in cytotoxicity produced by the 4-diphenylamino aryl ketone peptide **13c** (*p* < 0.001), which was a statistically significant increase from the WT peptide **11** (Figures 5c and S15). As the cytotoxic activity of the AERQ peptide is likely dependent on its ability to puncture holes in the membrane of the target cells, we hypothesize that the attachment of bulky aryl groups enhances this mode of action. However, the cytotoxic mechanism of the ketone peptide library is beyond the scope of our study. In practical terms, this demonstrates that a single Asn/Gln-to-ketone conversion can be leveraged to explore new pharmacological space around bioactive peptides without re-engineering the sequence or installing non-native handles during synthesis.

### Fluorosequencing for Identification of Asn Residues

Having demonstrated the chemical versatility of the ketone handle, we sought to exploit its site-specificity for analytical applications, specifically single-molecule protein sequencing. To achieve this goal, we synthesized the peptide WNGRNFG **14**, which contains two asparagine residues. Dehydration of both Asn residues furnished the corresponding dinitrile peptide **15** in >95% conversion, which was subsequently transformed into the diketone peptide **16** with complete conversion (Figure S16). This efficient dual modification underscores the method’s robustness and general applicability for simultaneous functionalization of multiple amide sites, establishing a foundation for fluorosequencing applications^39,40^ aimed at residue-specific identification of Asn.

To assess the compatibility with single-molecule protein sequencing, we synthesized two peptides, AVNGAYSYRA **17** and GANAGNAYGYR **18**, containing one and two Asn residues, respectively.

The Asn residues were dehydrated to nitriles, generating peptides **19**–**20**, which were subsequently converted into ketones using azide-substituted boronic acid, yielding single Asn ketone peptide **21** and diketone peptide **22** (Figures 6a and S17). In order to add the fluorophore(s) and introduce an alkyne on the C-terminus, the azide-functionalized peptides were captured on a 2-pyridinecarboxaldehyde-functionalized (PCA) resin^41^ (Figures 6b and S17). The use of the solid-phase capture-release strategy enabled easier purification as well as simplified coupling at the C-terminus, while the N-terminus remained attached to the resin and thus was inaccessible. The fluorophore(s) were added through copper-free Click chemistry between the azide handles on the peptides and Atto643-PEG4-DBCO. Subsequently, an alkyne was added to the C-terminus through coupling with propargylamine. Attempts to incorporate an alkyne handle earlier in the peptide synthesis proved to be inefficient, as the nitrile dehydration step proceeded poorly in the presence of the alkyne. Release of the peptide from the PCA-resin by heating in MeCN/200 mM TRIS pH 8 (1:1) afforded the fully labeled peptides **23**–**24**, which were immobilized on an azide-functionalized glass slide through copper-catalyzed click chemistry for fluorosequencing.

**Figure 6:**
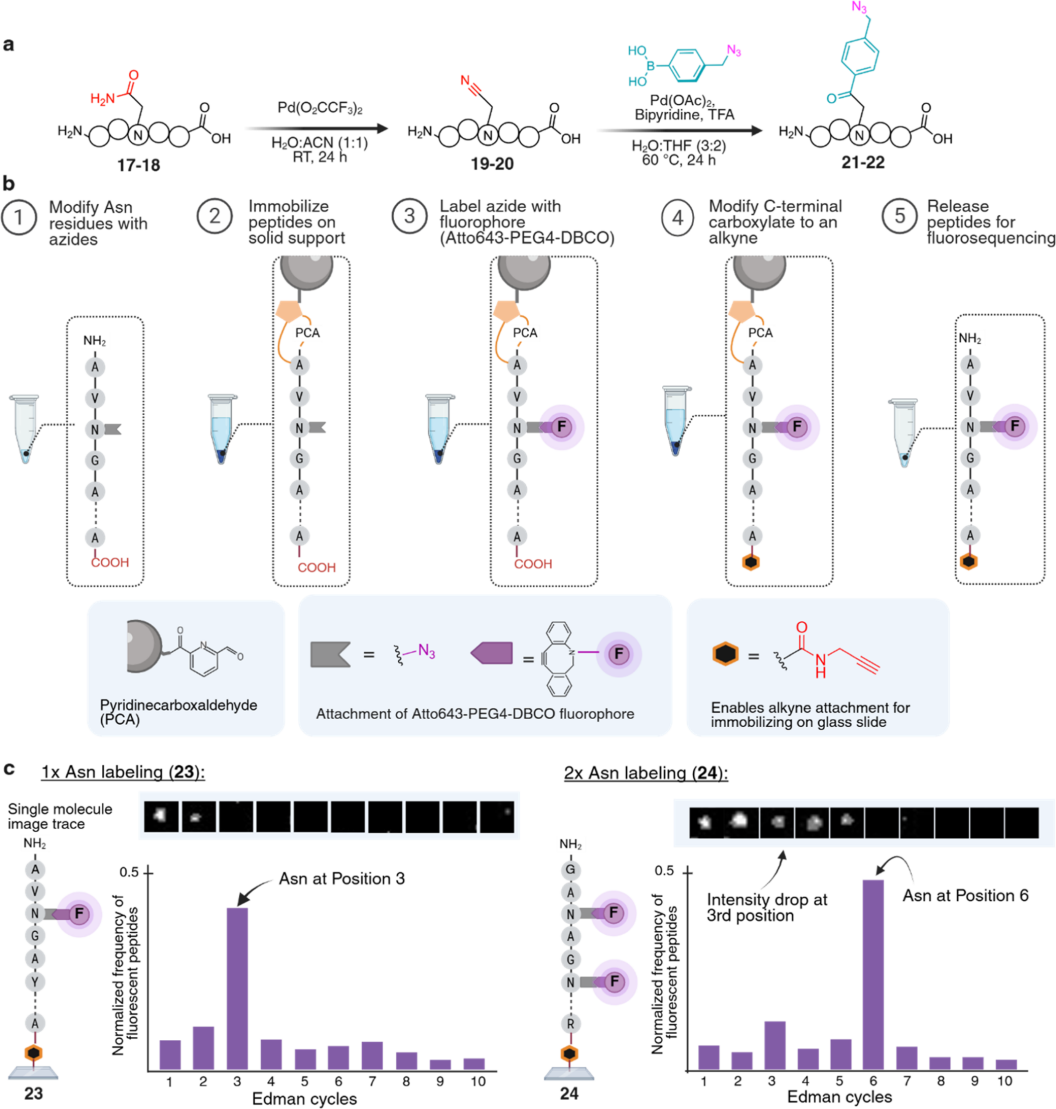
Application of the Asn/Gln modification workflow for fluorosequencing. (a) Labeling of Asn residues with azides generated ketone-modified peptides **21**–**22**. (b) Overview of end-to-end labeling process for fluorosequencing. The azide-functionalized peptides were captured through their N-terminus onto a PCA-resin. The fluorophores (Atto643-PEG4-DBCO) were added through copper-free Click chemistry, and an alkyne was introduced to the C-terminus through coupling with propargylamine. The fully functionalized peptides (**23**–**24**) were released from the PCA resin and immobilized on a glass slide through copper-mediated click chemistry. (c) Normalized frequency of Asn-labeled peptides, showing which cycle of the Edman degradation fluorescence intensity was lost. Immobilized peptides were subjected to sequential Edman degradation while fluorescence was monitored by total internal reflection fluorescence (TIRF) microscopy. A decrease in fluorescence intensity was observed only during the degradation cycle(s), removing a modified Asn residue, confirming site-specific identification. Created in BioRender. Lab, R. (2026) https://BioRender.com/j6xbtlt.

Fluorosequencing was performed as previously described.^42^ Briefly, the fluorophore-labeled peptides were immobilized and subjected to sequential Edman degradation cycles, while the fluorescence was monitored via TIRF microscopy. In each case, a step decrease in fluorescence intensity occurred exclusively during the degradation cycle corresponding to the removal of the modified Asn residue, precisely revealing its position within the peptide sequence (Figures 6c and S17). These findings demonstrate that our modification workflow gives single-molecule sequencing platforms a new, orthogonal chemical marker to visualize and identify previously undetectable residues, transforming Asn from a largely invisible residue into a sequence-defining chemical landmark and opening a previously inaccessible layer of primary-amide information to single-molecule proteomics.

### Selective Modification of Proteins

We next investigated the feasibility of selective Asn/Gln to nitrile modification in intact proteins using ubiquitin as a model system. Ubiquitin (∼0.2 mM), which contains six glutamine and two asparagine residues, was treated with varying concentrations of Pd­(OAc)_2_ (2 mM–10 mM) at room temperature for 2 h in a 4:1 solution of NaP buffer (10 mM, pH 7.4) and MeCN. Efficient modification (>95%) was achieved at Pd­(OAc)_2_ concentrations above 5 mM, with the extent of labeling increasing at higher Pd loadings (Figures 7a and S18). The nitrile-modified ubiquitin was additionally digested and analyzed by MS/MS, confirming selective modification of Asn/Gln residues (Figure S18).

**Figure 7:**
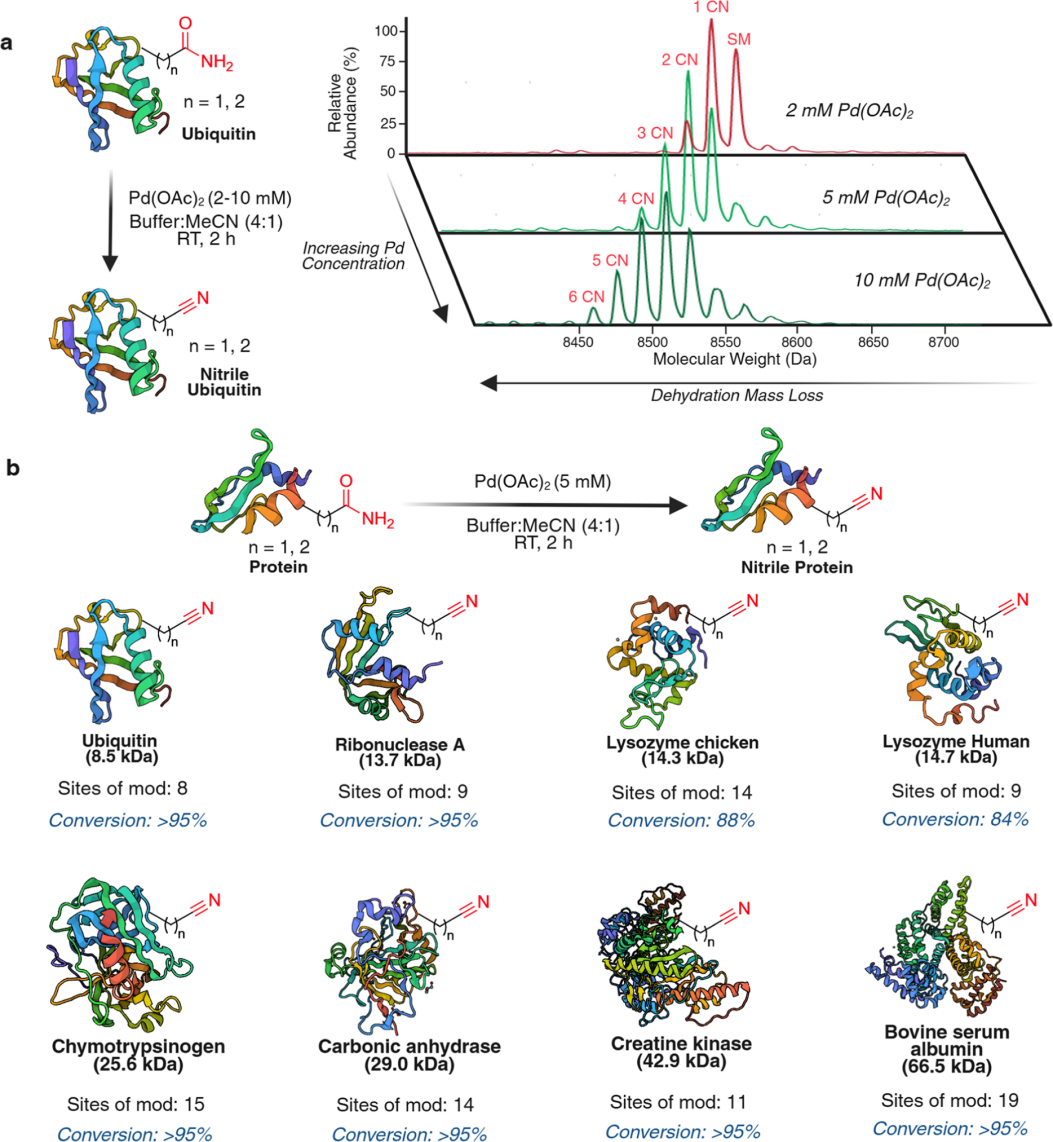
Selective modification of diverse protein substrates to nitrile. (a) Optimization of isohypsic dehydration reaction on ubiquitin (∼0.2 mM) with varying concentrations of Pd­(OAc)_2_ (2–10 mM) in 4:1 NaP buffer (10 mM, pH 7.4): MeCN, followed by palladium scavenging using 500 μL of 1 M aqueous solution of l-cysteine and 10 μL of 1 M NaOH solution. 5 mM Pd­(OAc)_2_ enabled >95% conversion of ubiquitin to nitrile, with modification further increasing at higher Pd­(OAc)_2_ concentrations. (b) Isohypsic dehydration of Asn/Gln to nitriles in various proteins (30–200 μM) independent of their sizes and 3D-structures with 5 mM of Pd­(OAc)_2_. High conversion (84%–95%) of asparagine and glutamine to nitriles on proteins was observed without the formation of side products with any other amino acids. Created in BioRender. Lab, R. (2026) https://BioRender.com/p3fx22p.

While the transformation proceeds via a catalytic cycle, stoichiometric palladium loadings (5 mM) were employed to drive reaction kinetics on low-concentration macromolecular substrates. Residual palladium was removed by sequential treatment with cysteine, which generates water-soluble Pd-thiolate complexes that are readily separable from the protein. This scavenging strategy is consistent with established Pd-mediated bioconjugation workflows known for effectively minimizing metal contaminants.^34–37^ ICP–MS analysis of the modified protein following dechelation showed a residual Pd concentration of 43.9 ng per 1 mg protein (43.9 ppm), corresponding to a molar Pd/protein ratio of 0.0036:1, indicating a Pd removal efficiency exceeding 99.99% (Figure S18). These trace levels satisfy ICH Q3D pharmaceutical standards^43^ and permit downstream analytical applications, including single-molecule fluorosequencing, activity-based protein profiling, and PTM identification.

Having established efficient Asn/Gln to nitrile conversion on protein using 5 mM Pd­(OAc)_2_, we evaluated the generality of this transformation across a panel of proteins differing in size (8.5–66.5 kDa) and tertiary structure, including ubiquitin, ribonuclease A, lysozyme (chicken), lysozyme human, chymotrypsinogen, carbonic anhydrase, creatine kinase, and bovine serum albumin (Figures 7b and S19a). All substrates underwent high-yield conversion (84–95%) with excellent selectivity for Asn/Gln, even at low protein concentrations (30 μM). Furthermore, systematic variation of Pd­(OAc)_2_ loading (2–10 mM) in the modification of chicken lysozyme demonstrates that the degree of labeling can be tuned by reaction conditions, enabling conditions that bias the reaction toward predominantly monomodified protein at low Pd loadings and higher modification states at increased Pd loadings (Figure S19a). Site mapping by tryptic digestion and MS/MS analysis confirmed nitrile formation on Asn/Gln residues across eight representative proteins (Figure S19a). We sought to evaluate whether Asn or Gln may be more prone to nitrile formation. Based on analysis of our MS/MS data, across the eight proteins, 52 out of 93 Asn sites (56%) and 46 out of 75 Gln sites (61%) were modified to nitrile, indicating that the two residues exhibit similar reactivity. To investigate whether surface accessibility dictates site selectivity, we performed a solvent accessible surface area (SASA) analysis.^44^ Utilizing a relative solvent-accessible surface area (RSA) threshold of 0.2, we found that nitrile formation is relatively independent of steric accessibility. Specifically, 66.3% of nitrile-modified Asn and 63.0% of modified Gln residues were solvent-exposed, values that closely parallel the overall exposure frequencies of these residues (73.6% and 67.6%, respectively) across the proteins studied (Figure S19b). These results suggest that factors beyond simple surface exposure govern the observed selectivity. Together, these results demonstrate a robust and broadly applicable strategy for the chemoselective nitrile installation in proteins.

### Boronic Acid Carbometalation for Protein Diversification

We next sought to leverage the nitrile handle for site-selective protein diversification with affinity and reporter motifs, including alkyne, biotin, and fluorophore tags.

Beginning with nitrile-modified ubiquitin (∼0.5 mM), we carried out a Pd-mediated carbometalation reaction in 4:1 H_2_O/THF containing Pd­(OAc)_2_ (9 mM), bpy (12.8 mM), 4-methoxyphenylboronic acid (25 mM), and TFA (1 μL). After incubation at 60 °C for 12 h, complete conversion of nitrile-modified sites to the corresponding aryl ketone products was observed (Figures 8a and S20). Notably, following the carbometalation chemistry, our Pd chelation workflow using l-cysteine once again enabled Pd removal efficiency exceeding 99.99% by ICP–MS.

**Figure 8:**
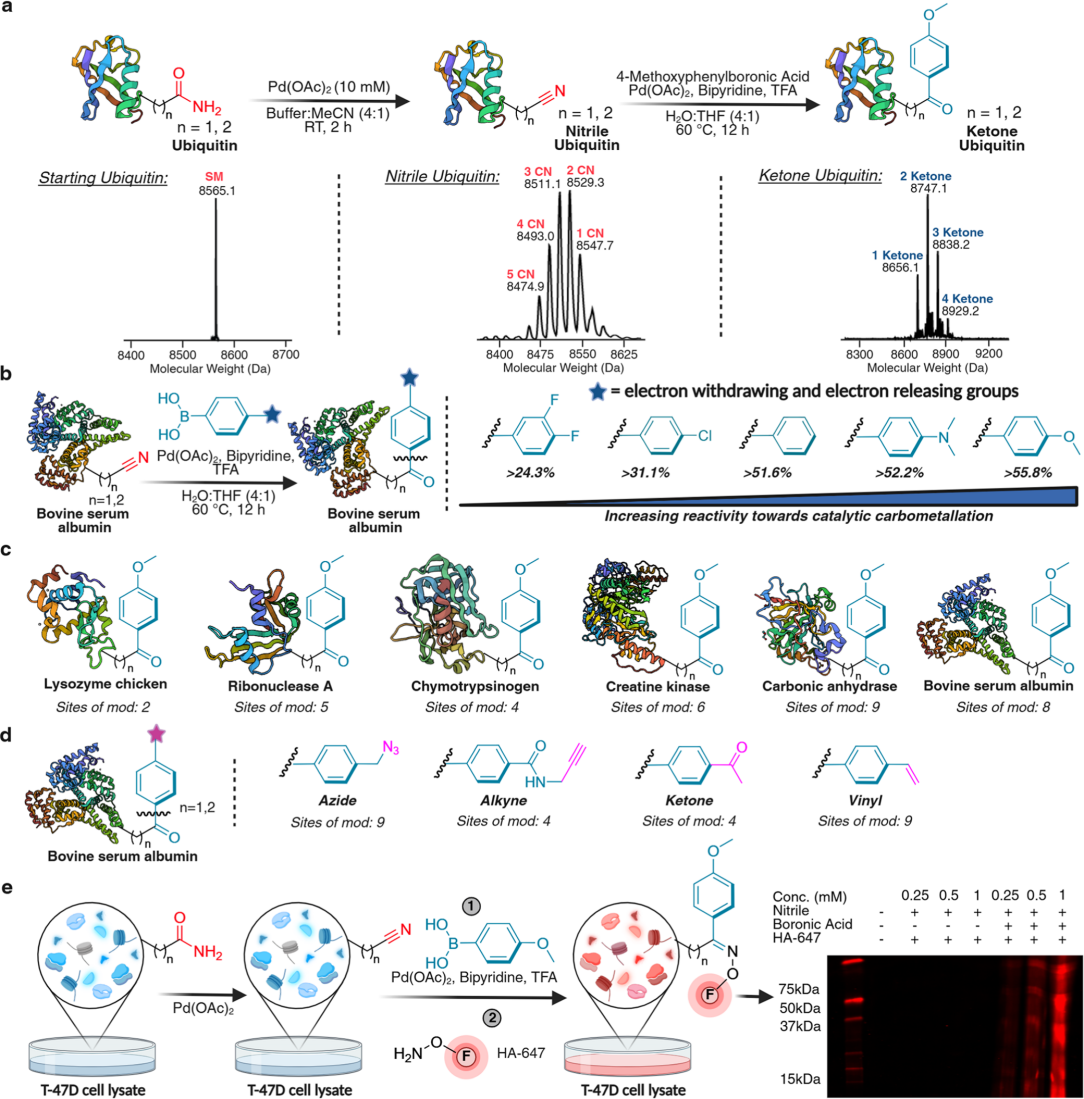
Selective dual modification and functionalization of asparagine and glutamine in proteins and complex cell lysates. (a) Modification of ubiquitin Asn/Gln residues to nitrile using 10 mM Pd­(OAc)_2_, followed by carbometalation of nitrile sites using EDG 4-methoxyboronic acid. Ubiquitin concentration was ∼0.2 mM for the nitrile formation step and ∼0.5 mM for carbometalation. Quantitative conversion of nitrile to a ketone carbometalation product was observed. (b) Evaluation of boronic acid electronic effects on the carbometalation of nitrile-modified bovine serum albumin (BSA) (60 μM final concentration). Electron-donating boronic acids (OMe and NMe_2_) gave significantly higher conversions than EWG boronic acids (*para*-Cl, 3,4-difluoro). (c) High substrate scope for Asn/Gln-specific protein functionalization (60–500 μM protein concentration) with 4-methoxyphenylboronic acid. 2–9 nitrile sites were modified to aryl ketones via carbometalation reaction. Note that not all sites previously modified with nitrile were observed to convert to the ketone product. (d) Dual functionalization of BSA (60 μM final concentration) with affinity handles using boronic acid derivatives containing azide, alkyne, ketone, and vinyl. (e) Dual modification of T-47D cell lysate with 4-methoxyphenylboronic acid and subsequent labeling of ketone handle with hydroxylamine AlexaFluor647 fluorophore, followed by in-gel fluorescence analysis. A dose-dependent increase in a fluorescence signal is observed across 0.25–1 mM of Pd­(OAc)_2_. Created in BioRender. Lab, R. (2026) https://BioRender.com/9s6gjqr.

TFA was included in a low concentration solely to promote proto-depalladation and Pd turnover. Importantly, the intrinsic buffering capacity of the protein solution maintained the reaction pH within a range compatible with native protein structure, preventing acid-induced denaturation despite the presence of TFA. The carbometalation reaction with nitrile-modified ubiquitin was also performed successfully in 10 mM sodium phosphate buffer (pH 7.45) in place of water, further supporting that TFA is present to promote Pd turnover rather than to acidify the reaction mixture, demonstrating that the transformation can proceed under physiologically mild pH conditions (Figure S20).

We then employed these conditions to evaluate various aryl boronic acids with EWG (3,4-difluoro, 4-chloro) and EDG (4 *N*,*N*-dimethyl and 4-methoxy) for the modification of nitrile-modified BSA protein. Mirroring our small-molecule kinetic profiling (vide supra), EDG-substituted boronic acids exhibited higher efficiency than EWG variants under these mild conditions (40–60 °C), validating our selection of electron-rich partners for temperature-sensitive protein bioconjugation (Figures 8b and S21). The reaction proved general across diverse proteins, including lysozyme (chicken), ribonuclease A, chymotrypsinogen, creatine kinase, carbonic anhydrase, and bovine serum albumin, all of which underwent selective nitrile-to-aryl ketone transformation (2–9 ketone sites from 8 to 19 nitrile sites) when treated with 4-methoxyphenylboronic acid (Figures 8c and S22a).

The observed variability in carbometalation efficiency appears to be governed by the steric microenvironment of the target site. Analysis of the Protein Data Bank (PDB) structures for the substrates in Figure 8c indicates that while successful ketone formation predominantly occurs at highly solvent-exposed residues, buried sites can remain reactive if surrounded by aromatic residues. In these instances, favorable pi-stacking interactions between the local aromatic microenvironment and the bipyridine ligand/arylboronic acid likely compensate for reduced steric accessibility (Figure S22b).

To confirm the preservation of the secondary structure under the reaction conditions required for converting Asn/Gln to nitrile to ketone, we performed CD studies on nitrile- and ketone-modified ubiquitin, lysozyme (chicken), ribonuclease A, and creatine kinase. For lysozyme (chicken) nitrile formation, CD spectra were additionally collected across an expanded range of Pd concentrations (2–10 mM) to assess structural robustness under varied Pd loadings. The data indicate that secondary structure is largely retained in all the proteins without evidence of aggregation, although additional caution may be warranted for thermally sensitive proteins with melting temperatures below around 50 °C (Figure S22c).^45^

Extending this approach, nitrile-modified BSA was efficiently dual-functionalized using boronic acids bearing azide, alkyne, ketone, or alkene groups, affording high conversions (4–9 sites) to the corresponding aryl ketones (Figures 8d and S23). Finally, the method was applied to a complex cell lysate where proteins were modified to aryl ketones and subsequently labeled with AlexaFluor647 hydroxylamine. In-gel fluorescence analysis confirmed successful coupling to the orthogonal ketone handle on multiple proteins (lanes 6–8, Figures 8e and S24).

### Synthesis of Antibody-Fluorophore Conjugates

The orthogonal ketone handle generated at Asn/Gln sites on proteins was next employed for synthesizing AFCs using trastuzumab mAb, a monoclonal antibody that targets the human epidermal growth factor receptor 2 (HER2) that is overexpressed in certain cancer cells.^46^ Trastuzumab was first subjected to amide dehydration using a lower concentration of 500 μM Pd­(OAc)_2_, which afforded site-specific modification at N437, Q441, and Q648 with an overall nitrile-to-antibody ratio of 3:1, as confirmed by LC–MS/MS (Figures 9a and S25). The triply nitrile-modified trastuzumab was then subjected to Pd-mediated carbometalation with 4-methoxyphenylboronic acid under optimized conditions to generate aryl ketone intermediates, which were subsequently functionalized with hydroxylamine-AlexaFluor647 to yield the desired AFC. Formation of the AFC was verified by in-gel fluorescence (Figures 9b and S25).

**Figure 9:**
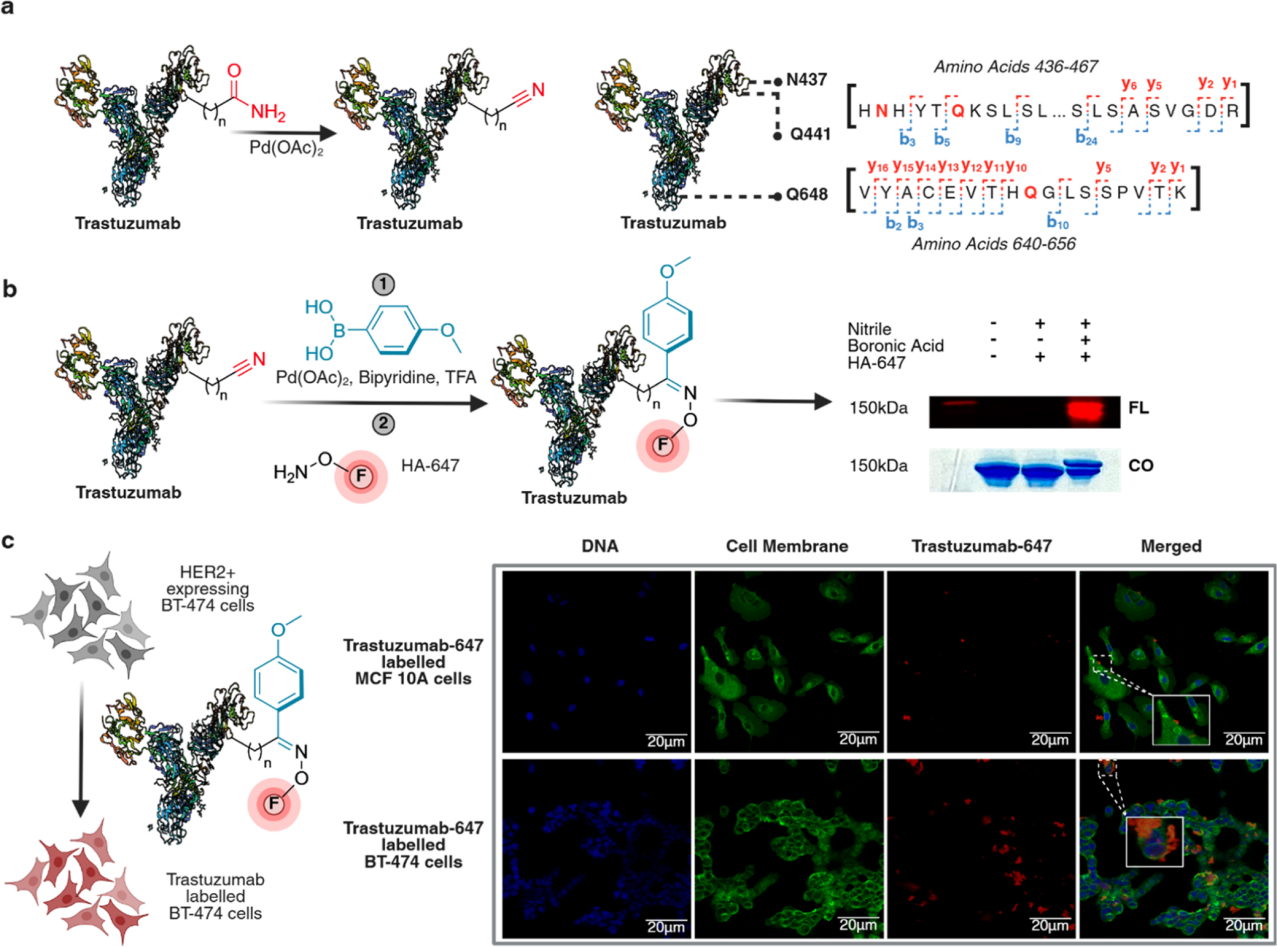
Synthesis of an Asn/Gln antibody-fluorophore conjugate through amide dehydration, followed by carbometalation. (a) Synthesis of trastuzumab-fluorophore conjugate using a lower concentration of 500 μM Pd­(OAc)_2_ for site-specific modification of trastuzumab (8.6 μM), leading to the modification of N437, Q441, and Q648 by nitrile formation as analyzed by LC-MS/MS. (b) Carbometalation reaction on nitrile-modified trastuzumab (6.9 μM) to generate ketone-modified trastuzumab. Subsequent functionalization of the ketone handle on modified trastuzumab with hydroxylamine AlexaFluor647 fluorophore and in-gel fluorescence analysis (lane 4). No fluorescence was observed without carbometalation (lane 3). (c) Fluorescence colocalization imaging of trastuzumab-647 with BT-474 human breast carcinoma cells characterized by the overexpression of human epidermal growth factor receptor 2 (HER2) and estrogen receptors (ER). Incubation led to a significant cell membrane localization of trastuzumab-AlexaFluor647, indicating binding of trastuzumab-647 to HER receptors on BT-474 cells. Incubation with nontumorigenic MCF 10A cells lacking significant overexpression of the HER2 receptor led to a minimal binding of trastuzumab-AlexaFluor647, further supporting the retention of activity of trastuzumab-647 after modification. The imaging was done in duplicate with separate cell passages. All images use the same scale bar: 20 μm. Created in BioRender. Lab, R. (2026) https://BioRender.com/am9gh0p.

To assess biological activity, the modified trastuzumab mAb was applied to HER2+ BT-474 breast cancer cells, showing strong and specific membrane labeling by confocal microscopy (Figures 9c and S26). In contrast, nontumorigenic MCF 10A cells lacking HER2 expression exhibited negligible signal. Critically, the retention of strong, specific binding to HER2+ cells (Figure 9c) combined with the lack of nonspecific binding to HER2-cells confirms that the antibody maintains its antigen-recognition capability under the carbometalation conditions. This stability is expected, as the reaction temperature (60 °C) remains below the typical unfolding threshold of IgG1 antibodies (*T*_m_ ∼ 70–80 °C), and the catalytic loading of TFA is effectively neutralized by the intrinsic buffering capacity of the protein solution.^47^

Furthermore, although mild thermal stress is a consideration for therapeutic development, these conditions are ideal for the primary analytical applications of this platform. In workflows such as single-molecule fluorosequencing, gel-based profiling, and bottom-up proteomics, proteins are often intentionally denatured or digested prior to analysis. In these contexts, the maintenance of a tertiary structure is not a prerequisite; rather, the priority is the chemical stability of the label. Therefore, this platform offers a distinct advantage for denaturing workflows: it provides a robust, covalent handle on “silent” Asn/Gln residues that survives harsh downstream processing, such as digestion or Edman degradation, unlocking a layer of proteomic information that is inaccessible to conformation-dependent or labile bioconjugation methods. These findings highlight the robustness and compatibility of the Asn/Gln-to-nitrile approach for the selective protein functionalization of stable proteins while preserving native structure and binding activity.

## Conclusion

In summary, we have established the first generalizable platform for activating the “silent” primary amides of Asn and Gln, overcoming the thermodynamic chelation barriers that previously restricted Pd-mediated dehydration to small molecules or protected peptides. By decoupling amide activation from competing metal-sequestration pathways inherent to complex proteins, we enable the selective generation of bio-orthogonal nitriles on native biomolecules of varying complexity. The resulting nitriles exhibit exceptional functional-group tolerance, readily undergoing Pd-mediated carbometalation with diverse aryl boronic acids to yield aryl ketone derivatives. This transformation provides access to noncanonical aryl-ketone amino acids and LSF of native peptides containing Asn and Gln residues with high selectivity. In contrast to established residue-selective chemistries that focus on nucleophilic side chains, our platform redefines neutral primary amides as programmable entry points for diversification. By coupling chemoselective dehydration with carbometalation, we create a unified, operationally simple route from native Asn/Gln to a broad range of functional outputs on peptides and proteins. Its operational simplicity, aqueous compatibility, and use of readily available boronic acid reagents facilitate the rapid generation of peptide libraries that explore previously inaccessible regions of chemical space. The method also translates directly to proteins, allowing site-selective modification of Asn/Gln residues across diverse structural classes and the synthesis of functional AFCs while preserving biological activity. Finally, the demonstrated compatibility of this chemistry with single molecule fluorosequencing introduces an orthogonal chemical marker for visualizing and identifying previously undetectable residues, transforming primary amides from silent participants into sequence-defining landmarks in the proteome.

## Supplementary Material



## Notes

Synthesis of the probes, optimization of the reaction conditions, the procedure of modification on peptides, intact proteins, cell lysates, chemoselectivity studies, cytotoxicity studies, cell imaging, fluorosequencing, and product characterization by NMR, HPLC, CD, ICP–MS, LC-MS, MS/MS, and HRMS (PDF)

## Abbreviations

LSF: late-stage functionalization
3-MPA: 3-mercaptopropionic acid
EDG: electron-donating group
EWG: electron-withdrawing group
BSA: bovine serum albumin
HER2: human epidermal growth factor receptors 2
ER: estrogen receptor
