# Supplementary material for: Unlocking the Silent Proteome: Chemoselective Asn/Gln Activation for Multidimensional Protein Diversification
Source: J Am Chem Soc. 2026 Mar 13;148(11):11974–90. doi: 10.1021/jacs.5c22184 (PMC13022855; doi:10.1021/jacs.5c22184)
Supplement: Supplementary file 1 [file ja5c22184_si_001.pdf]

Supporting Information

Unlocking the Silent Proteome: Chemoselective Asn/Gln Activation for Multidimensional Protein Diversification

Benjamin Emenike[1,#], Zachary E. Paikin[1,#], John M. Talbott[1], Anna Lidskog[2], Bao Quang Gia Le[1], Jagannath Swaminathan[2], Eric V. Anslyn[2], Monika Raj[1*]

[1]Department of Chemistry, Emory University, Atlanta, GA 30322, United States.

[2]Department of Chemistry, University of Texas at Austin, Austin, TX 78712, United States.

[#]These authors contributed equally.

Table of Contents

I. General 2

II. Materials 3

III. Analytical Methods 3

IV. Fmoc Solid-Phase Peptide Synthesis (Fmoc-SPPS). 3

V. Purification. 4

VI. Cell Culture Technique 4

Supplementary Fig. 1: Development of Amide Dehydration to Nitrile 4

- Supplementary Fig. 1a: Initial Reaction Exploration 4
- Supplementary Fig. 1b: Further Reaction Optimization 9
- Supplementary Fig. 1c: NMR Characterization of Nitrile Product 11

Supplementary Fig. 2: Chemoselectivity of Amide Dehydration to Nitrile 13

Supplementary Fig. 3: Evaluating Stability of Peptide Nitrile 27

Supplementary Fig. 4: Computational Analysis of Nitrile Reactivity 27

Supplementary Fig. 5: Exploration of Nitrile Modification Methods 31

- Supplementary Fig. 5a: Peptide Level Modification – Asn, Gln, and C-Terminal Nitrile Comparison 31
- Supplementary Fig. 5b: Further Exploration of Hydroxylamine Modification of Small Molecule Nitrile 46
- Supplementary Fig. 5c: Further Exploration of Boronic Acid Carbometallation on Small Molecule Nitrile 55

Supplementary Fig. 6: Small Molecule Boronic Acid Carbometallation Substrate Scope 57

Supplementary Fig. 7: Exploration of the Reactivity of Alkyl and Vinyl Boronic Acids 86

Supplementary Fig. 8: Synthesis of Unnatural Nitrile Amino Acids 87

Supplementary Fig. 9: Unnatural Aryl-Ketone Amino Acid Synthesis and Application 95

- Supplementary Fig. 9a: Carbometallation for Generation of Unnatural Aryl-Ketone Amino Acids 95

Supplementary Fig. 9b: Synthesis of Peptides Using Unnatural Amino Acid Building Blocks 114
Supplementary Fig. 10: Chemoselectivity of Boronic Acid Cross-Coupling........ 117
Supplementary Fig. 11: Solution Phase Peptide Diversification with Aryl Boronic Acids 118
Supplementary Fig. 12: Synthesis of Azide Boronic Acid 131
Supplementary Fig. 13: Decarbonylation During Carbometallation Reaction on Peptide 133
Supplementary Fig. 14: On-Resin Diversification of Asn/Gln to Nitrile and Aryl Ketones 137
Supplementary Fig. 14a: On-Resin Diversification Using Gln 137
Supplementary Fig. 14b: Asn Peptide Diversification Using On-Resin Deprotection of Asn(Trt) 146
Supplementary Fig. 15: Flow Cytometry of H_2H-AERQ-CO_2H Analogs 149
Supplementary Fig. 16: Modification of Two Asn Residues with Boronic Acid 153
Supplementary Fig. 17: Asn/Gln Fluorosequencing 156
Supplementary Fig. 18: Optimization of Amide Dehydration to Nitrile on Ubiquitin 165
Supplementary Fig. 19: Nitrile Dehydration on Diverse Proteins 175
Supplementary Fig. 19a: Scope of Nitrile Formation on Protein 175
Supplementary Fig. 19b: Analysis of Nitrile-Modified Sites 245
Supplementary Fig. 20: Modification of Ubiquitin Nitrile to Ketone Using Boronic Acid 249
Supplementary Fig. 21: Screening of Electronic Effects for Boronic Acid Nitrile Modification on Protein 254
Supplementary Fig. 22: Boronic Acid Modification of Nitrile on Diverse Proteins 268
Supplementary Fig. 22a: Scope of Boronic Acid Modification of Nitrile on Protein 268
Supplementary Fig. 22b: Analysis of Ketone-Modified Sites 288
Supplementary Fig. 22c: CD Spectroscopy Studies on Modified Proteins 288
Supplementary Fig. 23: Incorporation of Affinity Handles via Boronic Acid Carbometallation 291
Supplementary Fig. 24: Tagging of Boronic Acid Carbometallation Ketone Product with Hydroxylamine-647 Fluorophore in Cell Lysate 308
Supplementary Fig. 25: Boronic Acid Carbometallation for Synthesis of Trastuzumab-Fluorophore Conjugate 310
Supplementary Fig. 26: Immunofluorescence Microscopy Imaging Cells with Trastuzumab Fluorophore Conjugate 314
References 316

I. General. All commercial materials (Sigma-Aldrich, Ambeed, and ThermoFisher) were used without further purification. All solvents were reagent or HPLC (Fisher) grade. Percent conversions refer to chromatographically pure compounds. Reaction progress was monitored by TLC plates (TLC Silica gel 60 F_{254}) and visualized with UV lamps.

II. Materials. Fmoc-amino acids, Rink amide resin, and hexafluorophosphate benzotriazole tetramethyl uronium (HBTU) were obtained from CreoSalus (Louisville, Kentucky). Wang resin was obtained from Sigma Aldrich (St. Louis, Missouri). N,N′-diisopropylethylamine (DIPEA), 3-phenylpropionitrile, and 2,2'-bipyridyl ligand was obtained from TCI (Portland, Oregon). Piperidine and trifluoroacetic acid (TFA) were obtained from Alfa Aesar (Ward Hill, Massachusetts). N,N-dimethylformamide (DMF), dichloromethane (DCM), methanol (MeOH), and acetonitrile (ACN) were obtained from VWR (100 Matsonford Road Radnor, Pennsylvania). All other small molecules were obtained from Sigma and Combi-Blocks (San Diego, California). Commercially available proteins: lysozyme from chicken egg white, carbonic anhydrase, creatine kinase, bovine serum albumin (BSA), chymotrypsinogen, Ribonuclease A, and Trastuzumab mAb were obtained from Sigma. AlexaFluor™ 647 hydroxyl amine dye was obtained from Thermo Fisher Scientific. For gel analysis: 30% acrylamide mix, 1.5 M Tris buffer (pH 8.8), 10% SDS, 10% ammonium persulfate, and ladders were obtained from Bio-Rad.

III. Analytical Methods.

NMR: NMR spectra were recorded on a 400 MHz or 600 MHz Bruker NMR spectrometer. Proton chemical shifts were referenced to residual $CDCl_3$ at 7.26 ppm and carbon chemical shifts were referenced to $CDCl_3$ at 77.16 ppm. Spectra were processed using MestReNova ver. 12.0.4 and TOPSPIN software. The following abbreviations (or combinations thereof) are used to refer to multiplicities: s = singlet, d = doublet, t = triplet, q = quartet, p = quintet, and m = multiplet. Coupling constants (*J*), are reported in Hertz units (Hz).

HPLC: Peptide reactions were analyzed using high performance liquid chromatography (HPLC) on an Agilent 1100 series equipped with a 5 μm particle size, C-18 reversed-phase column. All separations involved a mobile phase of water with 0.1% formic acid (solvent A) and acetonitrile with 0.1% formic acid (solvent B) with a flow rate of 1 mL/min. The eluent was monitored by absorbance at 220 nm and 254 nm. **HPLC METHOD A:** Gradient: 0-80 % B over 30 min. **HPLC METHOD B:** Gradient: 2-60 % B over 30 min. **HPLC METHOD C:** Gradient: 2-20 % B over 30 min. **HPLC METHOD D:** Gradient: 2-30 % B over 30 min.

HRMS. High resolution MS data were acquired on Thermo Exactive Plus using a heated electrospray source. The solution was infused at a rate of 10-25 $\mu L\ min^{-1}$ electrospray using 3.3 kV. The typical settings were Capillary temp 320 °C. S-lens RF level was between 30-80 with an AGC setting of 1 E6. The maximum injection time was set to 50 ms. Spectra were taken at 140,000 resolutions at m/z 200 using Tune software and analyzed with ThermoFischer's Freestyle software. ver. 1.8.63.0.

IV. Fmoc Solid-Phase Peptide Synthesis (Fmoc-SPPS).[1] Peptides were synthesized using standard protocols. Peptides were synthesized manually on a 0.25 or 0.40 mmol scale using Rink amide resin or Wang resin. Resin was swollen with DCM for 1 hour at RT. Fmoc was deprotected using 20 % piperidine in DMF for 30 min to obtain a deprotected resin. Fmoc protected amino acid (1.25 mmol or 2.00 mmol, 5 equiv.) was coupled using HBTU (1.25 mmol or 2.00 mmol, 5 equiv.) and DIPEA (1.25 mmol or 2.00 mmol, 5 equiv.) in DMF for 25 min at RT. Fmoc deprotection was achieved using 20% piperidine in DMF for 20 min at RT. Peptides were cleaved from the resin using 10 mL of a cocktail consisting of 95:2.5:2.5 trifluoroacetic acid : water :

triethylsilane for 2 hours. The resin was removed by filtration and the resulting solution was concentrated via air. Peptides were precipitated and centrifugated with cold diethyl ether (3 x 10 mL) to obtain the crude product. Crude peptides were dissolved in ACN:H_2O and purified by preparatory HPLC.

V. Purification. Purification of peptide starting materials was performed using high performance liquid chromatography (HPLC) on an Agilent 1100 series HPLC equipped with a C-18 reverse phase column with a particle size of 5 µm or Teledyne ISCO ACCQ Prep HP150 equipped with a C-18 reverse phase 9.4x250 mm column with a particle size of 5 µm. All separations involved a mobile phase of 0.1 % formic acid in water (solvent A) and 0.1 % formic acid in acetonitrile (solvent B). The HPLC method used a linear gradient at RT with a flow rate of 1 mL min^{-1}. The eluent was monitored by absorbance at 220 nm and 254 nm.

VI. Cell Culture Technique. Cells were maintained at 37 °C and 5% CO_2. T-47D, HeLa, and BT-474 cells were cultured in RPMI 1640 media supplemented with 10% (V/V) fetal bovine serum (FBS) and 1% (V/V) penicillin/streptomycin (100 µg/mL). MCF 10A cells were cultured in DMEM/F12 (1:1) media supplemented with 3 mL FBS, 1% (V/V) penicillin/streptomycin (100 µg/mL), EGF (20 ng/mL), hydrocortisone (0.5 mg/mL), cholera toxin (100 ng/mL), and insulin (10 µg/mL).

Cell Lysis. Whole cell lysate was generated by lysing cells on ice in RIPA buffer (50 mM TrisHCl [pH 8], 150 mM NaCl, 1% NP-40, 0.5% sodium deoxycholate, 0.1% SDS) supplemented with protease and phosphatase inhibitors. Lysates were centrifuged 6,500 x g, 10 min at 4 °C, and soluble lysate was collected. Whole cell lysate proteins were separated using 16% SDS-PAGE. SDS-PAGE gels were stained with Coomassie brilliant blue dye.

Supplementary Fig. 1: Development of Amide Dehydration to Nitrile

Supplementary Fig. 1a: Initial Reaction Exploration

Model reaction on H_2N-FQG Peptide 1a – Palladium Adducts Observed

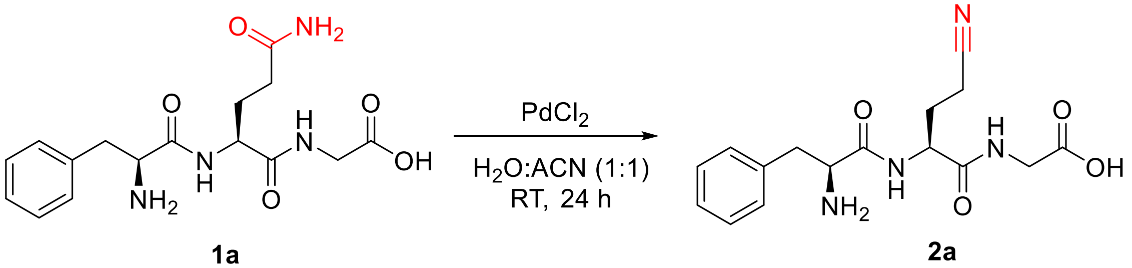

H_2N-FQG-CO_2H (**1a**) (1.0 mg, 2.86 µmol, 1 equiv.) was dissolved in 600 µL of 1:1 H_2O:ACN in a 1/2” dram vial. Next, 10 mol% of $PdCl_2$ was added in one portion. The reaction was stirred at room temperature for 24 h and analyzed via **HPLC Method B** to determine percent conversion to **2a**. Analysis led to the observation of palladium adducts on peptide **1a** and product **2a**. 52% conversion to palladium adduct of nitrile product **2a** was observed.

NH_2-FQ(Nitrile)G-CO_2H 2a-peptide palladium adduct. LCMS, m/z 437.0418 (calcd. $[M+H^+]$ = 437.0436), Purity: >99% (HPLC analysis at 220 nm). Retention time in HPLC: 8.0 min.

NH_2-FQG-CO_2H 1a-peptide palladium adduct. LCMS, m/z 455.0516 (calcd. $[M+H^+]$ = 455.0469), Purity: >99% (HPLC analysis at 220 nm). Retention time in HPLC: 3.9 min.

Structures of proposed palladium-peptide adducts:

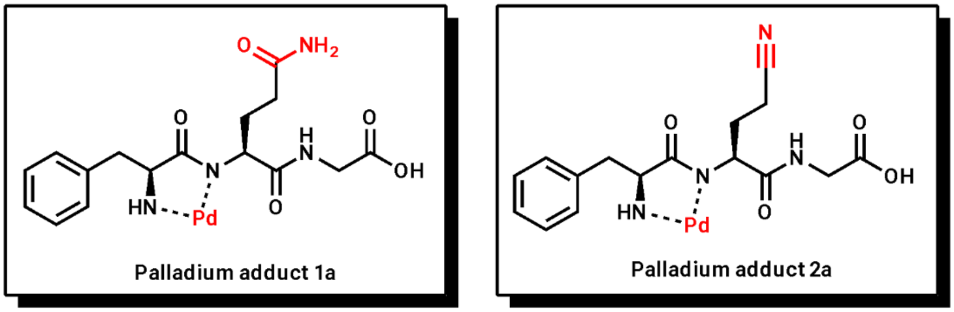

HPLC Trace for Reaction of H_2N-FQG-CO_2H 1a with $PdCl_2$

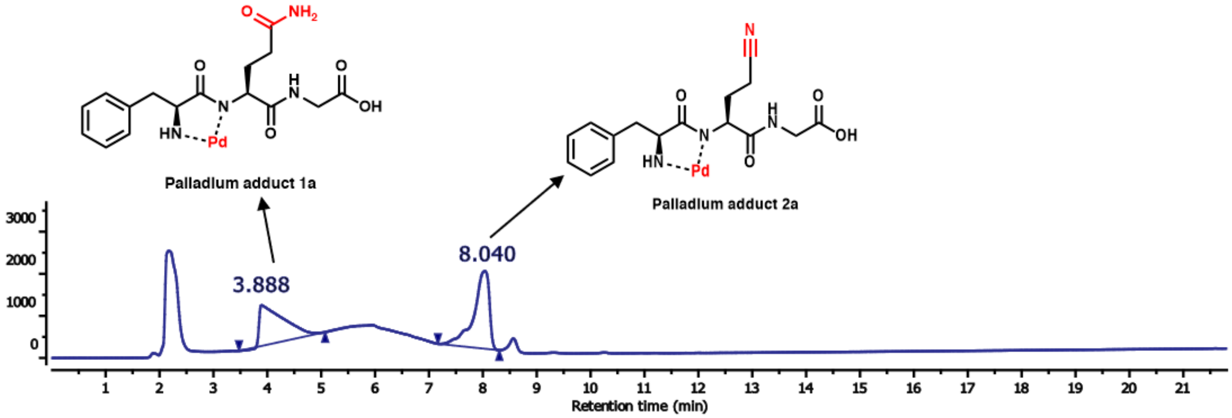

HRMS of Palladium Adduct with Nitrile Product 2a (peak 8.0 min)

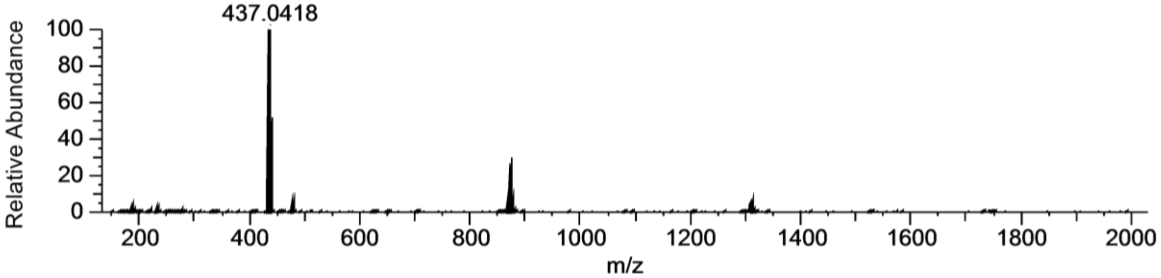

HRMS of Palladium Adduct with H_2N-FQG 1a (peak 3.9 min)

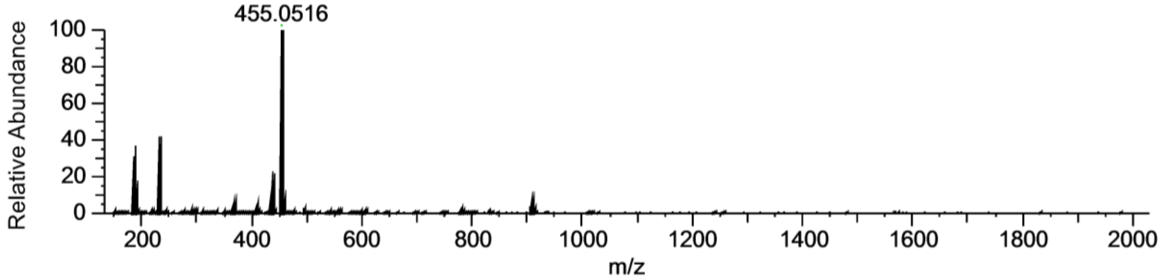

Model Reaction for Nitrile Formation on Ac-FQG Peptide 1b

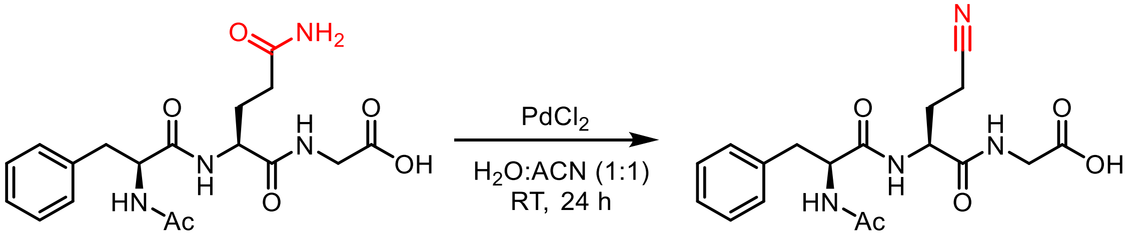

1b **2b**

Ac-FQG-CO_2H (**1b**) (1.0 mg, 2.86 µmol, 1 equiv.) was dissolved in 600 µL of 1:1 H_2O:ACN in a 1/2” dram vial. Next, 10 mol% of $PdCl_2$ was added in one portion. The reaction was stirred at room temperature for 24 h and analyzed via **HPLC Method B** to determine percent conversion to **2b**. 86% conversion to nitrile product **2b** was observed.

Ac-FQG-CO_2H peptide, 1b. LCMS, m/z 393.1752 (calcd. [$M+H^+$] = 393.1769), m/z 415.1570 (calcd. [$M+Na^+$] = 415.1588), m/z 785.3436 (calcd. [$2M+H^+$] = 785.3464), m/z 807.3254 (calcd. [$2M+Na^+$] = 807.3284), Purity: > 99% (HPLC analysis at 220 nm). Retention time in HPLC: 7.1 min.

Ac-FQ(Nitrile)G-CO_2H peptide, 2b. LCMS, m/z 375.1645 (calcd. [$M+H^+$] = 375.1663), m/z 397.1463 (calcd. [$M+Na$] = 397.1482) m/z 749.3223 (calcd. [$2M+H^+$] = 749.3253), Purity: > 99% (HPLC analysis at 220 nm). Retention time in HPLC: 8.0 min.

HPLC Trace for 1b (Ac-FQG-CO_2H) Reaction with $PdCl_2$

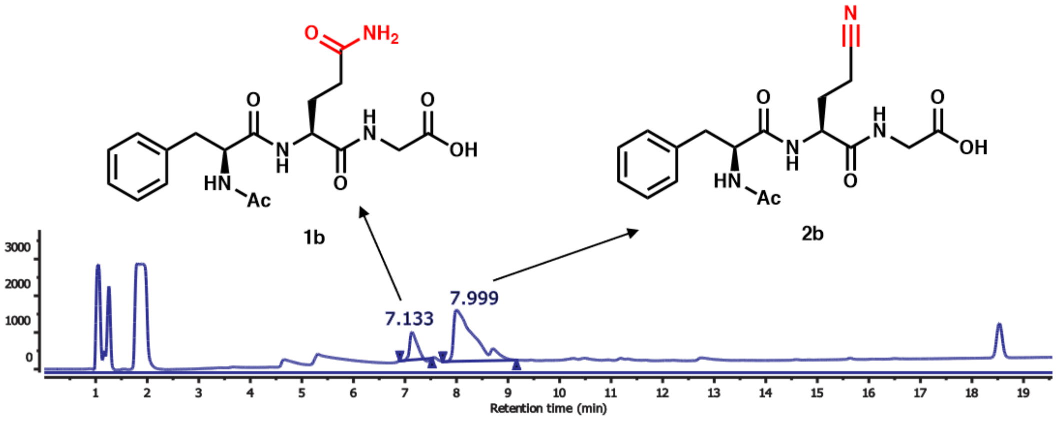

HRMS of 1b (peak 7.1 min)

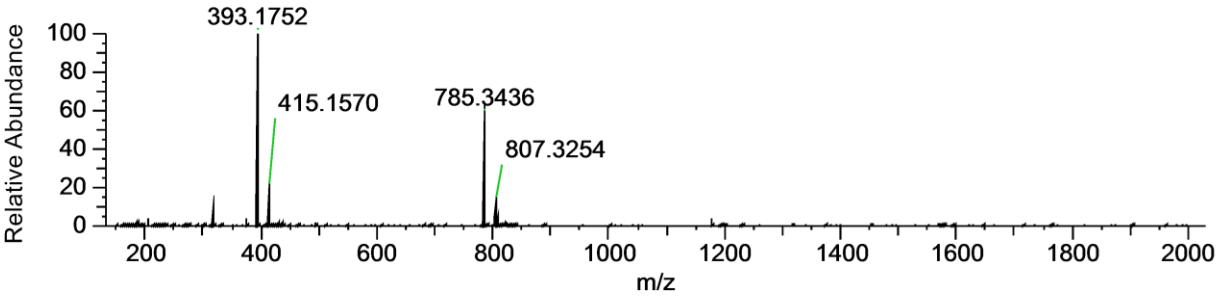

HRMS of 2b (peak 8.0 min)

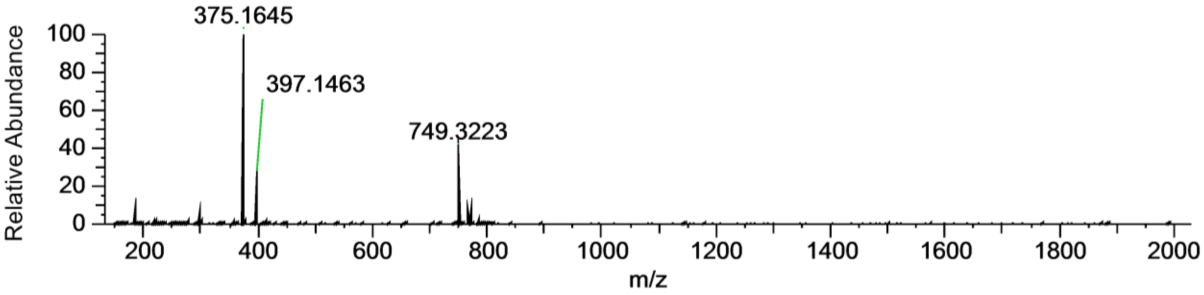

Scavenging of Palladium-Peptide Adduct

To apply nitrile dehydration to peptides containing a free N-terminus (location prone to Pd chelation), we needed to develop a method to scavenge Pd adducts from peptides. We evaluated various palladium scavenging conditions using 1 M sodium hydroxide, 1 M hydrochloric acid, 1 M solutions of ethylene diamine (EDA), 3-mercaptopropionic acid (3-MPA), and dithiothreitol (DTT). The use of EDA, 3-MPA, or DTT led to the complete removal of palladium from peptides.

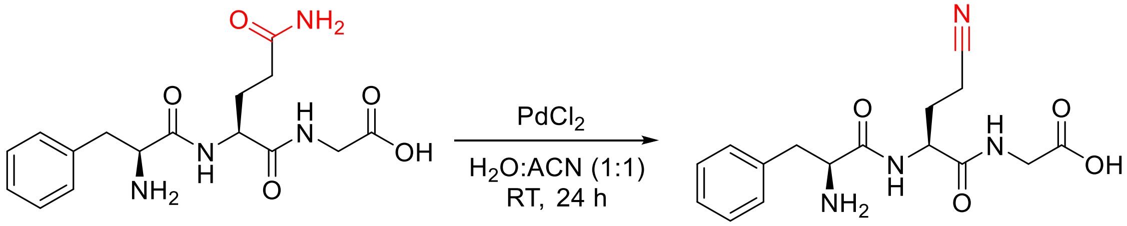

1a **2a**

H_2N-FQG-CO_2H (**1a**) (1.0 mg, 2.86 µmol, 1 equiv.) was dissolved in 600 µL of 1:1 H_2O:ACN in a 1/2” dram vial. Next, 1 equiv. of $PdCl_2$ was added in one portion. The reaction was stirred at room temperature for 24 h and analyzed via **HPLC Method A** to determine percent conversion to **2a**. To scavenge palladium-adduct, we screened scavengers such as ethylene diamine (EDA), 3-mercaptopropionic acid (3-MPA), and dithiothreitol (DTT) and observed the complete dissociation of palladium complex from peptide **1a** and **2a**. 94% conversion of starting peptide to nitrile product **2a** was observed after using EDA for chelation. Note: excess EDA elutes at ~1.3 min in **HPLC method A**.

H_2N-FQG-CO_2H peptide 1a. LCMS, m/z 351.1647 (calcd. [M+H+] = 351.1668), m/z 701.3228 (calcd. [2M+H+] = 701.3259), Purity: > 99 % (HPLC analysis at 220 nm). Retention time in HPLC: 2.7 min.

H_2N-FQ(Nitrile)G-CO_2H peptide 2a. LCMS, m/z 333.1541 (calcd. [M+H+] = 333.1563), Purity: >99 % (HPLC analysis at 220 nm). Retention time in HPLC: 3.6 min.

HPLC Trace of 1a (H_2N-FQG-CO_2H)

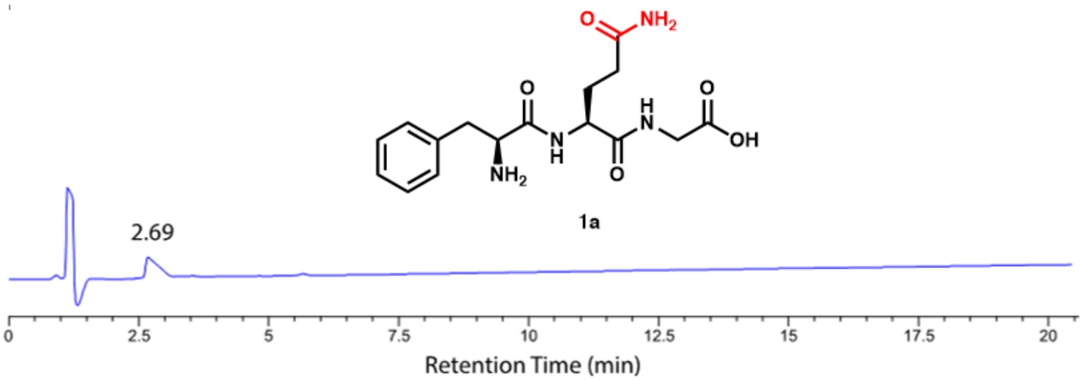

HPLC Trace of 2a (H_2N-FQ(CN)G-CO_2H)

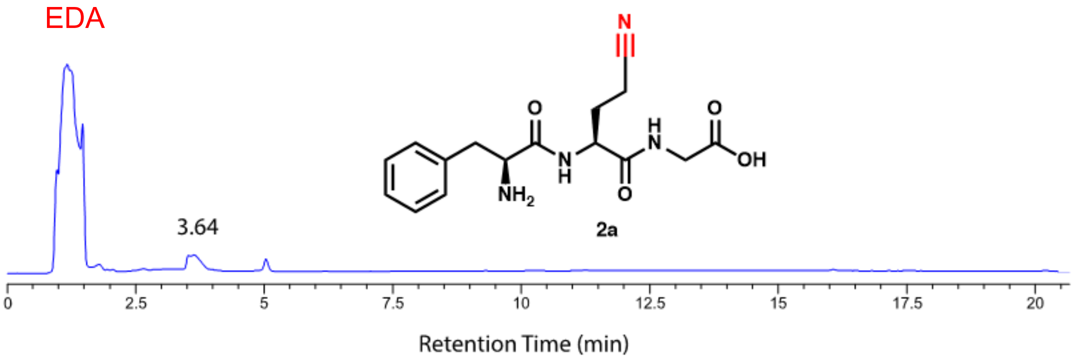

HRMS of 1a (peak 2.69 min)

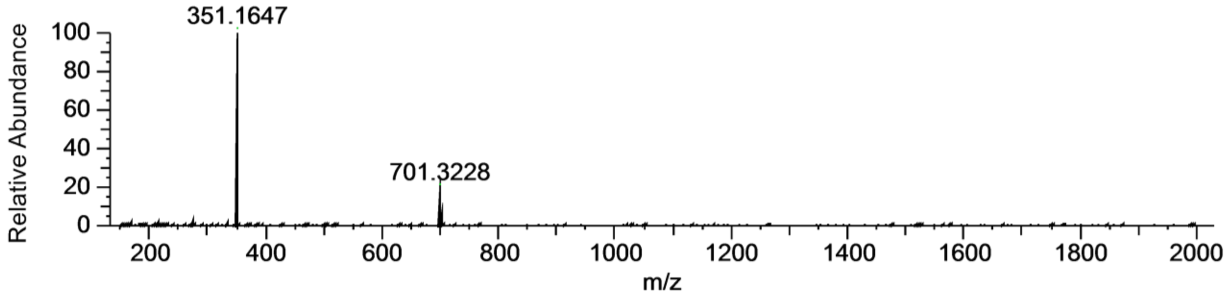

HRMS of 2a (peak 3.64 min)

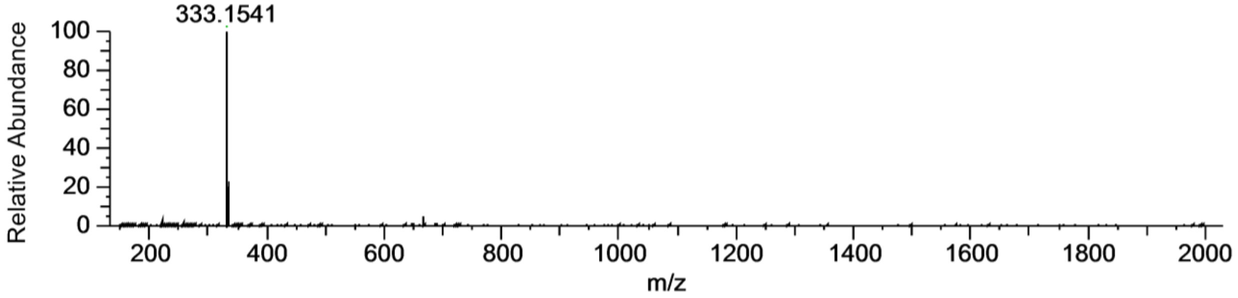

Supplementary Fig. 1b: Further Reaction Optimization

Screening of Water-Soluble Palladium Scavengers

To address the issue of water-insoluble Pd complexes that could form upon Pd scavenging in protein reactions, we evaluated generation of water-soluble palladium complexes. These complexes could then be easily removed from protein mixtures by filtration through an Amicon™ Ultra 3 kDa centrifugal filter. Palladium complexes formed with ethylene diamine, 3-mercaptopropionic acid and dithiothreitol were insoluble while L-cysteine and 2,3-dimercapto-1-propanesulfonic acid led to generation of water-soluble palladium complexes.

Ultimately, we opted to move forward with L-cysteine (under basic conditions) as our Pd chelator for purification of subsequent protein reactions.

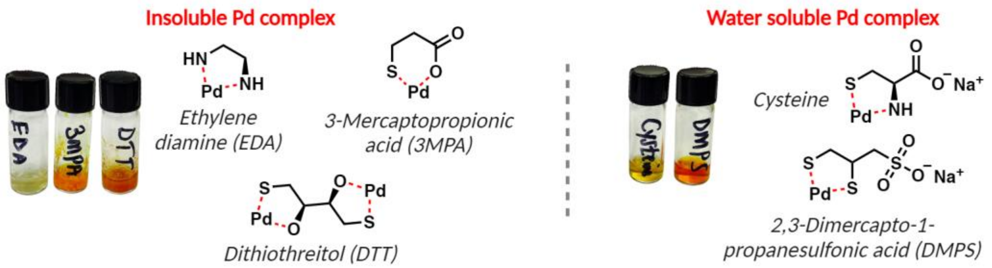

Screening of Palladium Salts

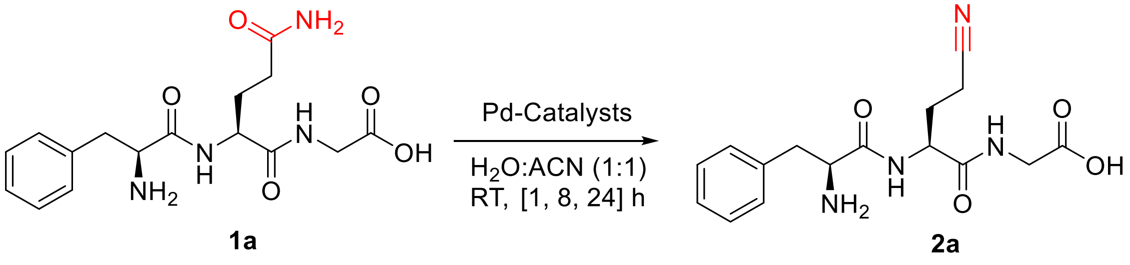

H_2N-FQG-CO_2H (**1a**) (1.0 mg, 2.86 µmol, 1 equiv.) was dissolved in 600 µL of 1:1 H_2O:ACN in a 1/2” dram vial. Next, 1 equiv. of various Pd(II) catalysts including $Pd(OAc)_2$, $PdCl_2$, $PdCl_2(CH_3CN)_2$, $Pd(O_2CCF_3)_2$, or Pd_2dba_3 was added in one portion in separate reactions. A Pd(0) example was conducted by adding $Pd(O_2CCF_3)_2$ with sodium ascorbate (50 equiv.), which is known to reduce Pd(II) to Pd(0). All solutions turned transparent light brown upon Pd addition except Pd_2dba_3 which was insoluble. The concentration of peptide in solution was 4.76 mM. The vial was stirred at room temperature and an aliquot was removed after 1 h, 8 h, and 24 h then

quenched with ethylene diamine (5 µL, 28 equiv.). Reaction was analyzed via **HPLC Method A** to determine percent conversion to **2a**.

	% Conversion to **2a**[a]		
Pd Catalyst	1 hour	8 h	24 h
**$PdCl_2$**	20%	67%	94%
**$Pd(OAc)_2$**	61%	90%	93%
**$PdCl_2(CH_3CN)_2$**	15%	65%	99%
**$Pd(O_2CCF_3)_2$ w/ Sodium Ascorbate**	5%	6%	8%
**Pd_2dba_3**	N/A[b]	N/A[b]	N/A[b]
**$Pd(O_2CCF_3)_2$**	**90%**	**>99%** (2 h)	ND[c]

[a]Note: **2a** is seen at retention times of ~2.5 min and ~5.0 min due to palladium chelation. Scavenging was done with ethylene diamine. [b]Pd_2dba_3 was insoluble in 1:1 ACN/H_2O. [c]Data not collected.

Screening of Activated Dehydrating Reagents

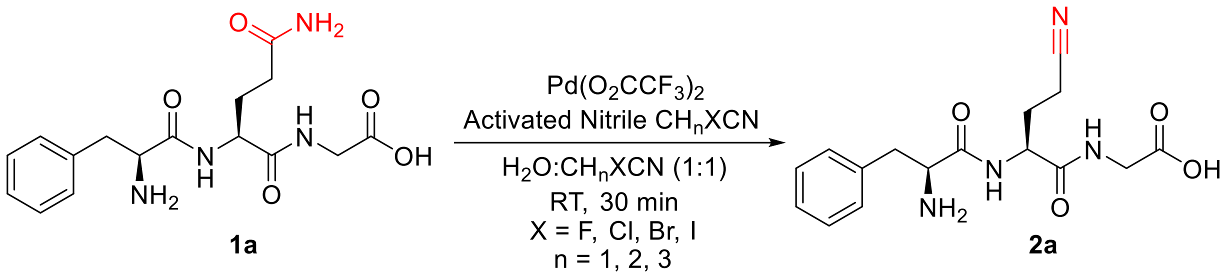

H_2N-FQG-CO_2H (**1a**) (1.0 mg, 2.86 µmol, 1 equiv.) was dissolved in 600 µL of 1:1 H_2O:ACN in a 1/2" dram vial. Next, the activated nitrile (fluoro- or chloro-, bromo- or iodo- or dichloro- 10 equiv.) was added. Finally, $Pd(O_2CCF_3)_2$ (1.0 mg, 3.07 µmol, 1 equiv.) from a freshly prepared stock solution in ACN was added in one portion. The concentration of peptide in solution was 4.76 mM. The vial was stirred at room temperature for 30 min then quenched with ethylene diamine (5 µL, 28 equiv.). Reaction was analyzed via HPLC Method A to determine percent conversion to **2a**. Ultimately, the addition of activated nitrile did not accelerate conversion to nitrile peptide **2a**, and we opted to move forward without utilizing activated nitrile additives.

Solvent	Percent Conversion to **2a**
Activated Nitrile	**T = 30 min**
CH_3CN	**86%**
CH_2FCN	72%
CH_2ClCN	79%
CH_2BrCN	73%
CH_2ICN	78%
$CHCl_2CN$	71%

Solvent Screening for Dehydration to Nitrile

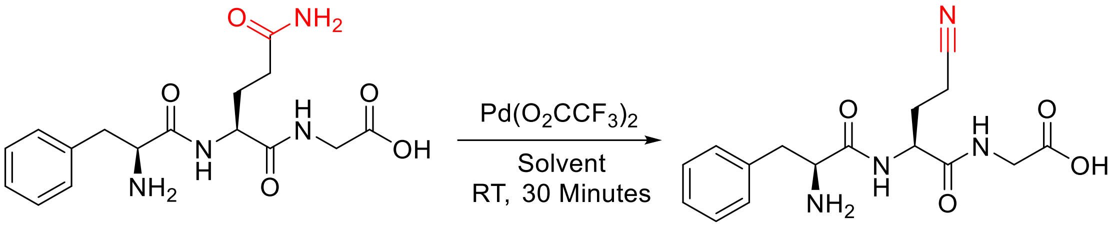

1a **2a**

H_2N-FQG-CO_2H **1a** (1.0 mg, 2.86 µmol, 1 equiv.) was dissolved in 150 µL of ACN in a 1/2" dram vial. Next, 450 µL of aqueous solvent (H_2O or 10 mM NaP buffer pH 7.4) was added to the vial. Finally, $Pd(O_2CCF_3)_2$ (1.0 mg, 3.07 µmol, 1 equiv.) from a freshly prepared stock solution in ACN was added in one portion. The concentration of peptide in solution was 4.76 mM. The vial was stirred at room temperature for 30 min then quenched with ethylene diamine (5 µL, 28 equiv.). The reaction was analyzed via **HPLC Method A** to determine percent conversion to **2a**. No significant difference was observed using the different solvent combinations.

Solvent System	Percent Conversion to 2a
1:1 H_2O:ACN	86%
4:1 H_2O:ACN	84%
4:1 Buffer:ACN	83%

HPLC Trace of Reaction with Varying Solvent Systems

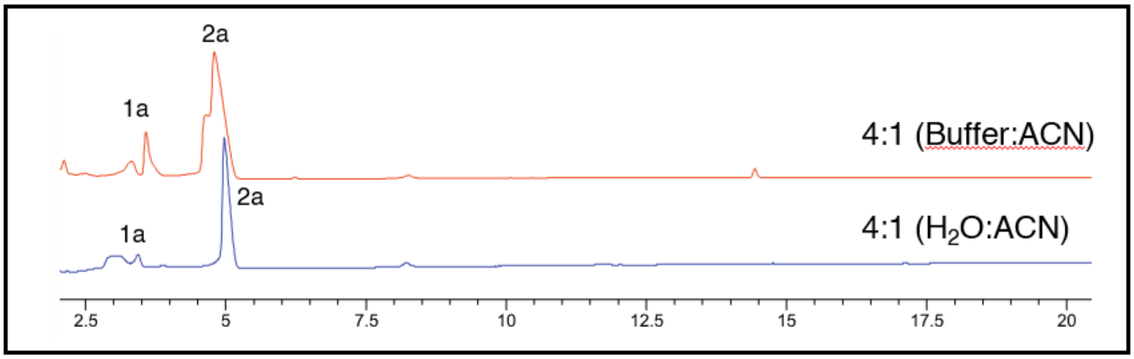

Supplementary Fig. 1c: NMR Characterization of Nitrile Product

NMR Characterization of Nitrile Product

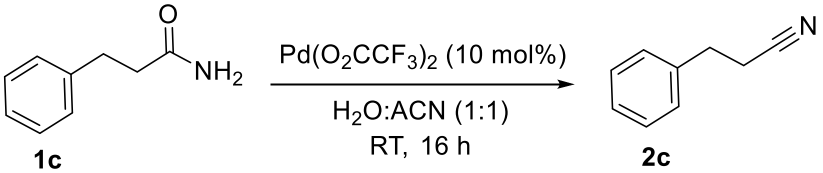

1c **2c**

3-phenylpropamide **1c** (1 g, 6.7 mmol, 1 equiv.) was added to a 25 mL RBF and dissolved in 12 mL of 1:1 H_2O:ACN. Then, $Pd(O_2CCF_3)_2$ (223 mg, 0.67 mmol, 10 mol%) was added and stirred at room temperature for 16 hours. Upon completion, ethylene diamine (450 µL, 1 equiv.) was added to chelate Pd. Next, the reaction mixture was extracted with EtOAc (2 x 40 mL), and the organic layer was dried over Na_2SO_4, concentrated, and purified by column chromatography (5 x

17 cm column, 100% EtOAc, 18 mL fractions) to afford compound 3-phenylpropionitrile **2c** as a clear liquid (0.86 g, 98%). Analytical TLC, 7:3 EtOAc:Hex eluent, $R_f = 0.90$.

^{1}H NMR (400 MHz, $CDCl_3$) δ: 7.40-7.22 (5H, m), 2.99 (2H, t, *J* = 7.4 Hz), 2.65 (2H, t, *J* = 7.4 Hz). **^{13}C NMR** (101 MHz, $CDCl_3$) δ: 138.2, 129.0, 128.4, 119.3, 31.7, 19.5. **HRMS**: calcd. for $C_9H_{10}N^+$ [M+H$^+$] 132.0808; found 132.0809.

^{1}H NMR of 3-Phenylpropionitrile 2c

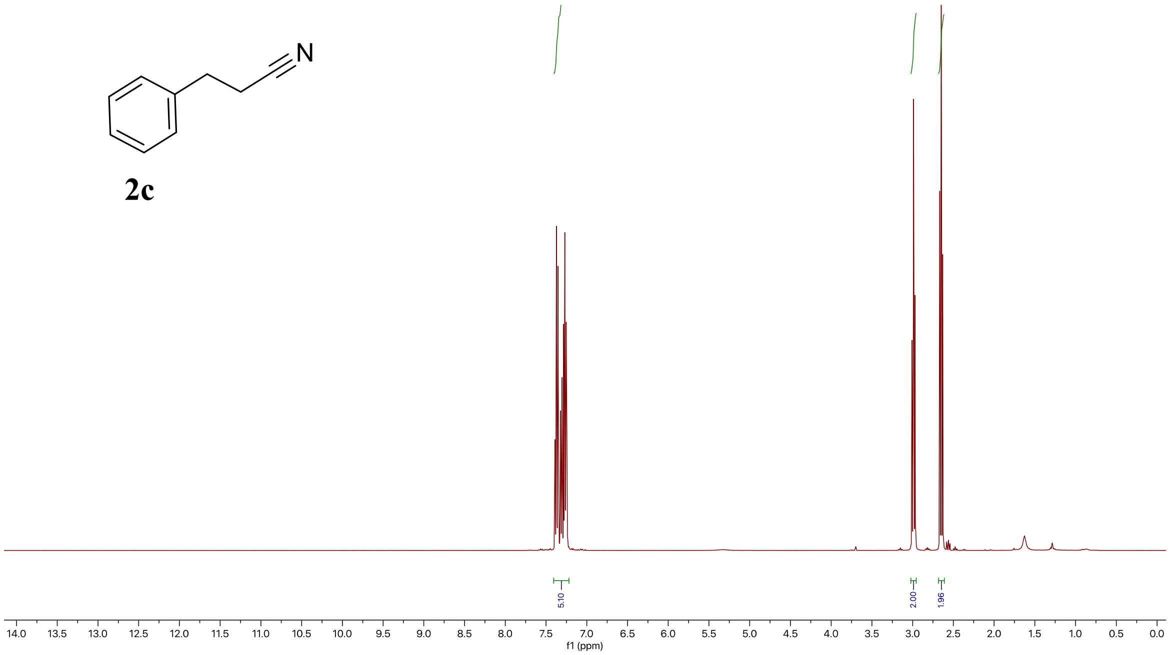

^{13}C NMR of 3-Phenylpropionitrile 2c

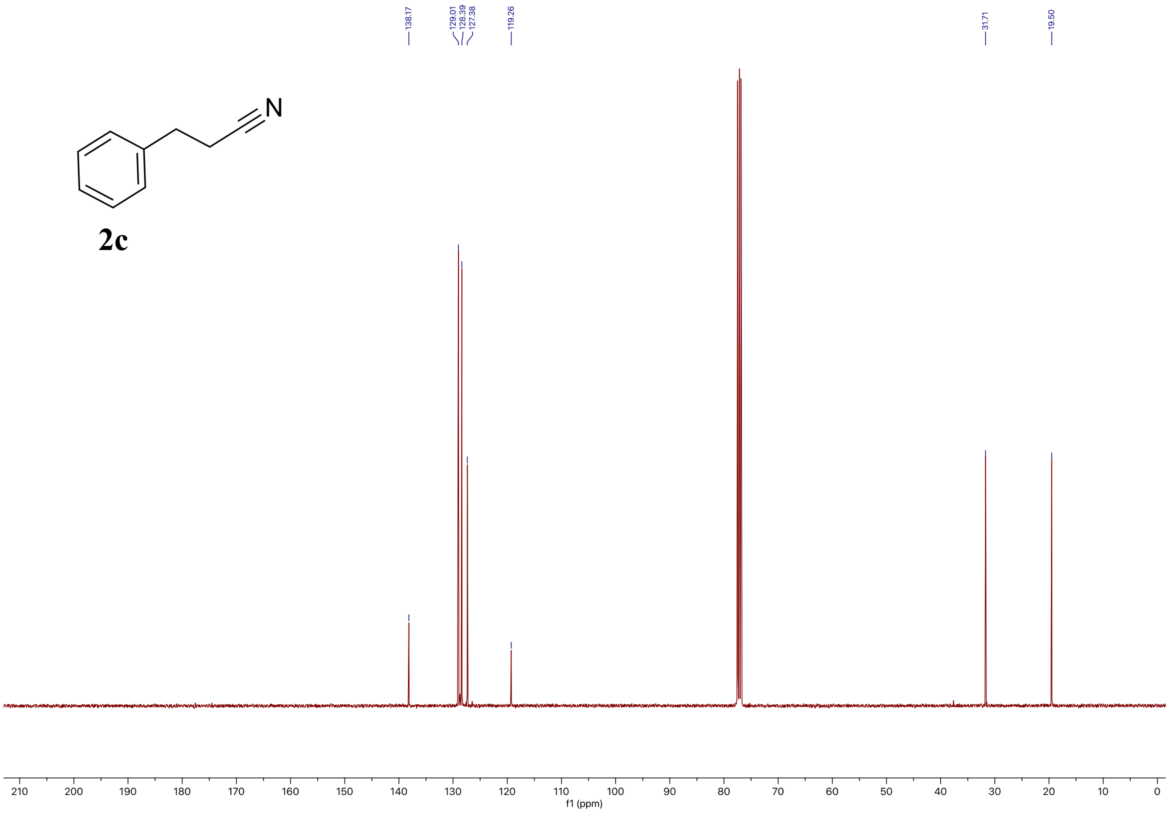

Supplementary Fig. 2: Chemoselectivity of Amide Dehydration to Nitrile

Chelation of Pd with 3-MPA after the reaction leads to the observation of 3-MPA and Palladium-complexes on the HPLC traces. To account for these, we incubated 2 µL of 3-MPA with 1 mg of $Pd(O_2CCF_3)_2$ in a 1:1 solution of H_2O:ACN for 5 min. The reaction was analyzed via **HPLC Method A**, and representative peaks for 3-MPA and Pd complexes were identified. These peaks correspond to the additional peaks observed in the chemoselectivity reactions discussed below.

HPLC Trace of 3-MPA and Palladium Complexes

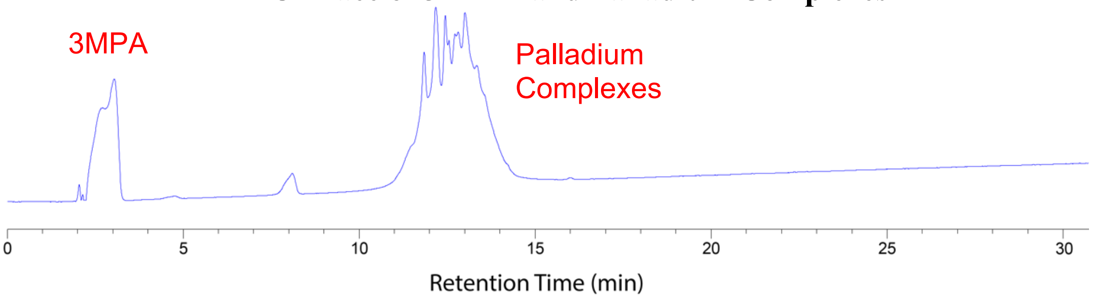

Reaction with Tyrosine Peptide H_2N-FYG-CO_2H 1d

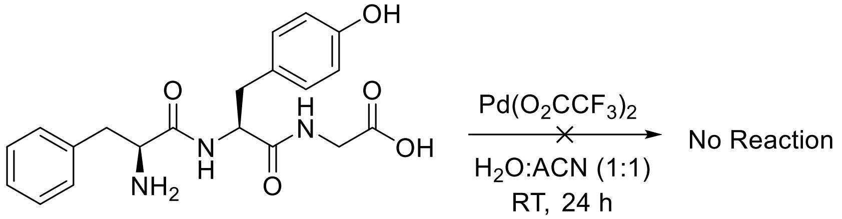

1d

H_2N-FYG-CO_2H **1d** (1.0 mg, 2.6 µmol, 1 equiv.) was dissolved in 600 µL of 1:1 H_2O:ACN in a 1/2" dram vial. Next, $Pd(O_2CCF_3)_2$ (0.86 mg, 2.6 µmol, 1 equiv.) from a freshly prepared stock solution in ACN was added in one portion. The concentration of peptide in solution was 4.33 mM. The vial was stirred at room temperature for 24 h then quenched with 3-MPA (5.5 µL, 25 equiv.). Reaction was analyzed via **HPLC Method A**. No conversion was observed.

H_2N-FYG-CO_2H peptide 1d. LCMS, m/z 386.1585 (calcd. [M+H+] = 386.1716) Purity: > 99 % (HPLC analysis at 220 nm). Retention time in HPLC: 5.1 min, 5.5 min (reaction).

HPLC Trace of 1d

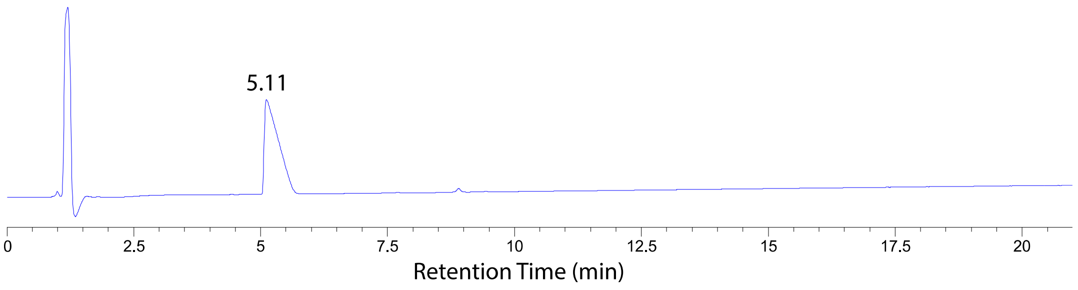

HPLC Trace of Reaction

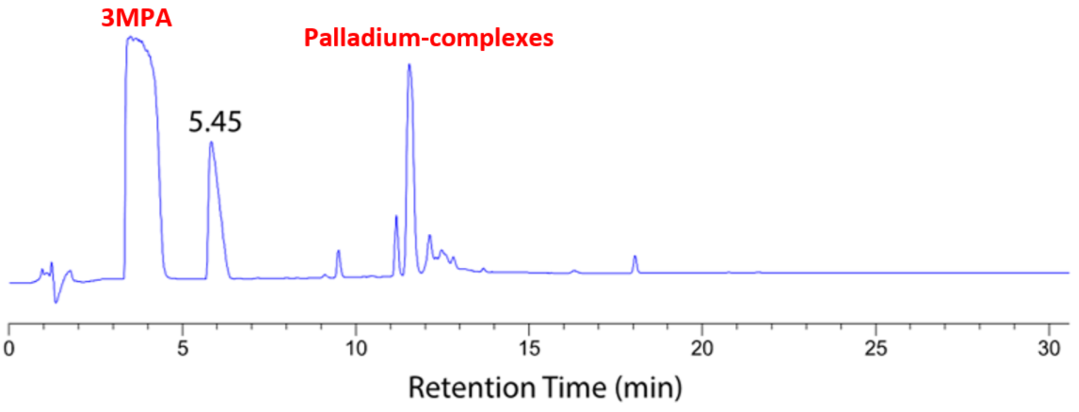

HRMS of 1d

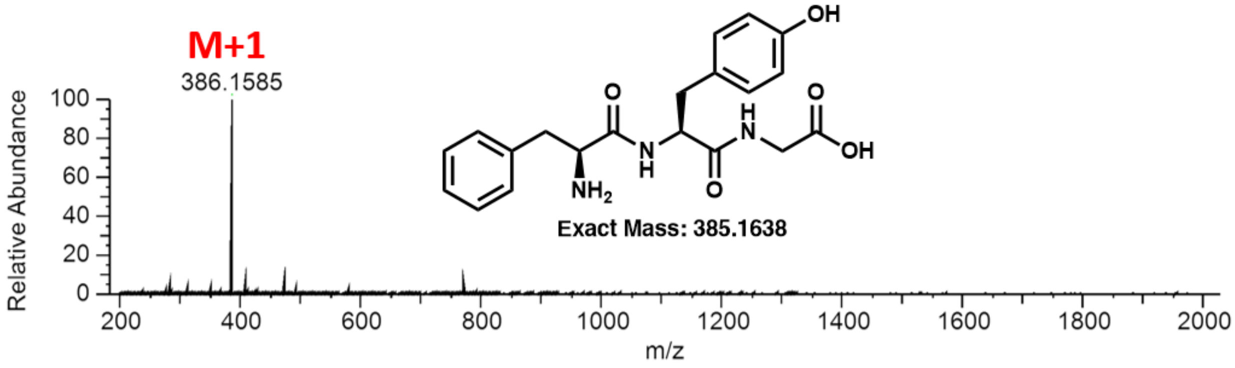

Reaction with Arginine Peptide H_2N-FRG-CO_2H 1e

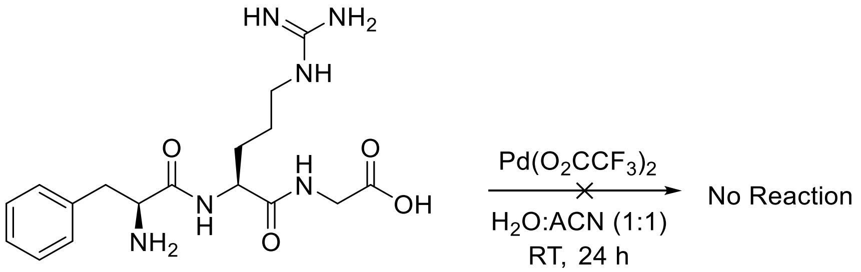

1e

H_2N-FRG-CO_2H (**1e**) (1.0 mg, 2.6 µmol, 1 equiv.) was dissolved in 600 µL of 1:1 H_2O:ACN in a 1/2" dram vial. Next, $Pd(O_2CCF_3)_2$ (0.88 mg, 2.6 µmol, 1 equiv.) from a freshly prepared stock solution in ACN was added in one portion. The concentration of peptide in solution was 4.33 mM. The vial was stirred at room temperature for 24 h then quenched with 3-MPA (5.5 µL, 25 equiv.). Reaction was analyzed via **HPLC Method A**. No conversion was observed.

H_2N-FRG-CO_2H peptide 1e. LCMS, m/z 379.2185 (calcd. $[M+H^+]$ = 379.2094) Purity: > 99 % (HPLC analysis at 220 nm). Retention time in HPLC: 1.9 min, 2.1 min (reaction).

HPLC Trace of 1e

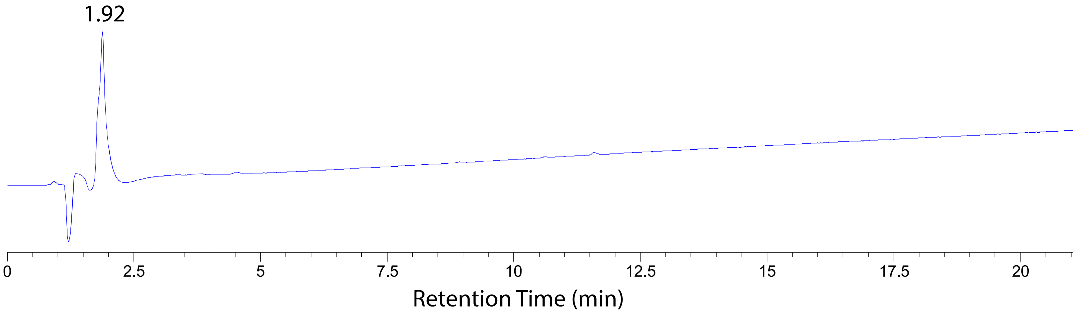

HPLC Trace of Reaction

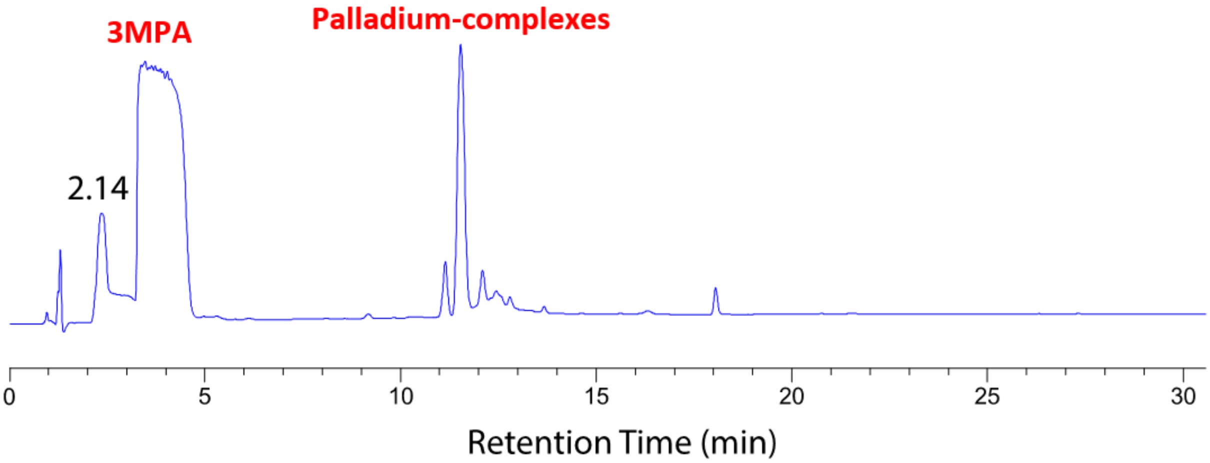

HRMS of 1e

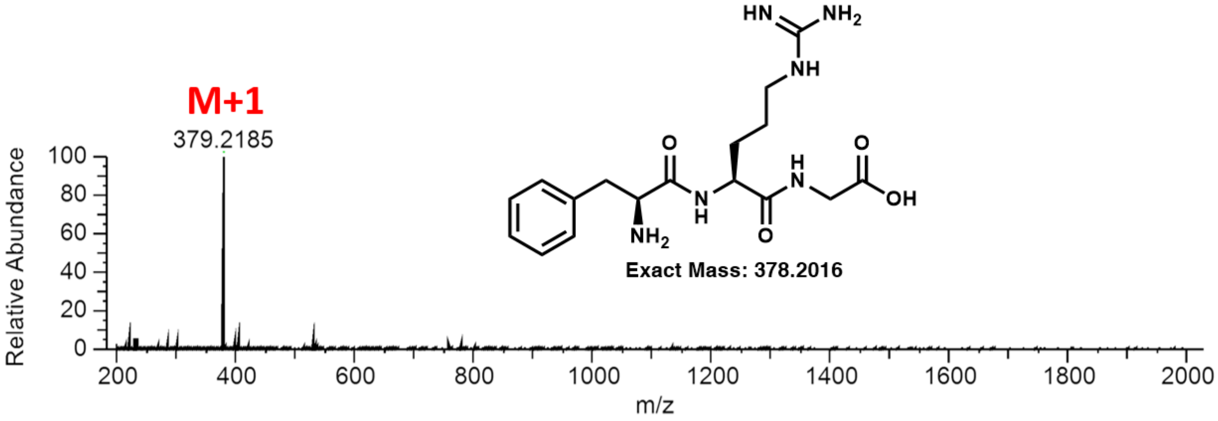

Reaction with Cysteine Peptide H_2N-FCG-CO_2H 1f

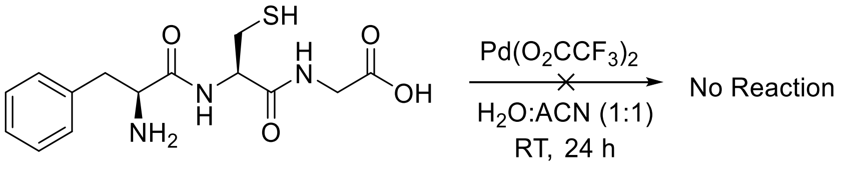

1f

H_2N-FCG-CO_2H **1f** (1.0 mg, 3.07 µmol, 1 equiv.) was dissolved in 600 µL of 1:1 H_2O:ACN in a 1/2" dram vial. Next, $Pd(O_2CCF_3)_2$ (1.0 mg, 3.07 µmol, 1 equiv.) from a freshly prepared stock solution in ACN was added in one portion. The concentration of peptide in solution was 5.12 mM. The vial was stirred at room temperature for 24 h then quenched with 3-MPA (7 µL, 25 equiv.). TCEP was added before the reaction was analyzed via **HPLC Method A**. No conversion was observed.

H_2N-FCG-CO_2H peptide 1f. LCMS, m/z 326.1227 (calcd. [M+H+] = 326.1175), m/z 651.2379 (calcd. [M+H+] = 651.2453. Purity: > 99 % (HPLC analysis at 220 nm). Retention time in HPLC: 8.4 min. Reaction retention time in HPLC was 6.1 min due to addition of TCEP.

HPLC Trace of 1f

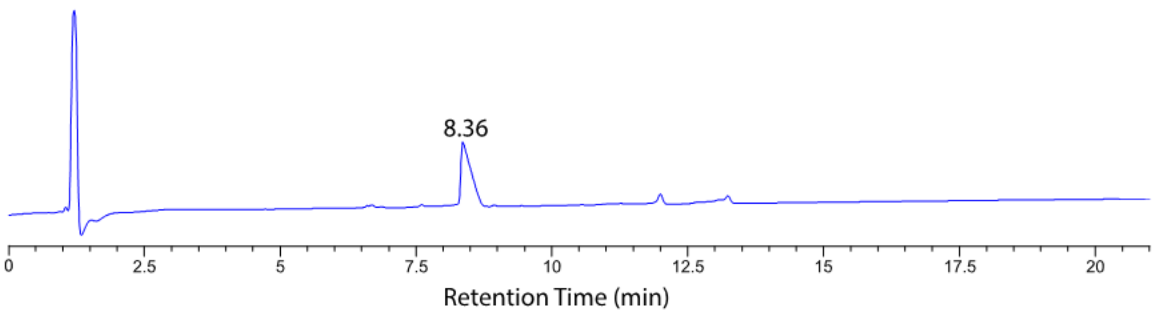

HPLC Trace of Reaction

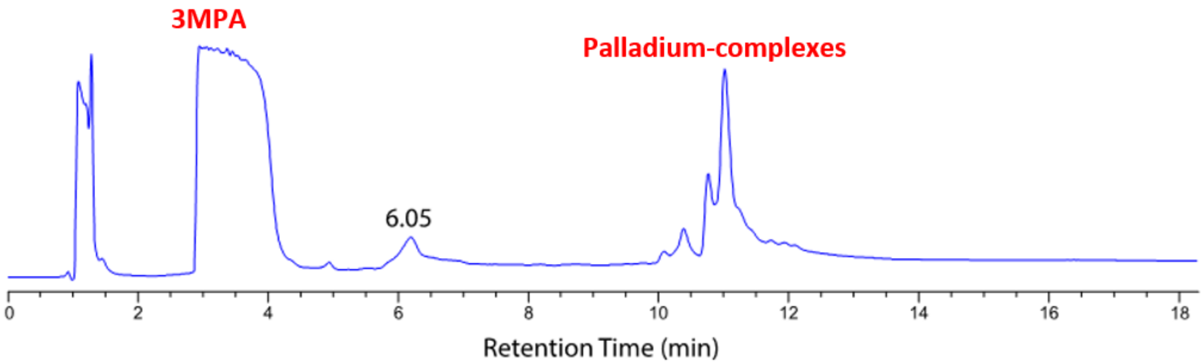

HRMS of 1f

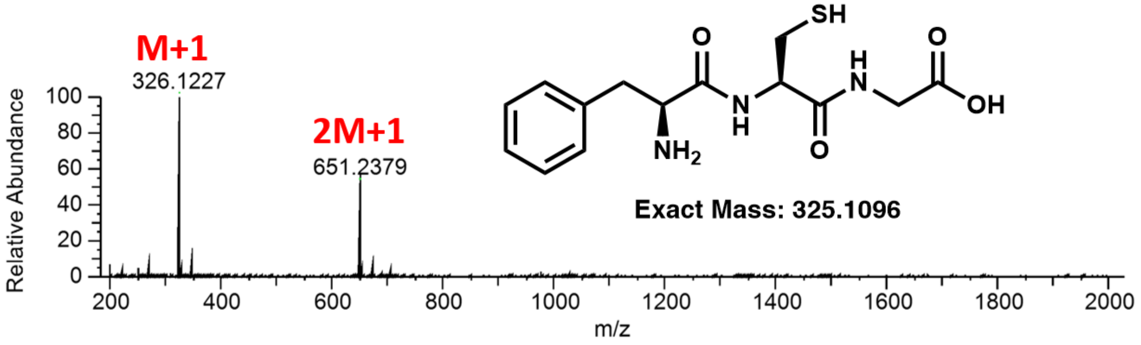

Reaction with Tryptophan Peptide H_2N-FWG-CO_2H 1g

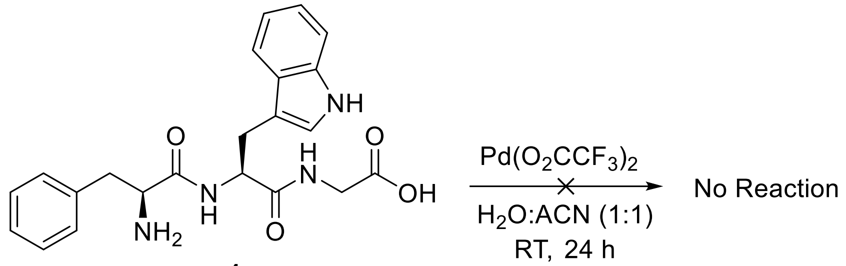

1g

H_2N-FWG-CO_2H **1g** (1.0 mg, 2.45 µmol, 1 equiv.) was dissolved in 600 µL of 1:1 H_2O:ACN in a 1/2” dram vial. Next, $Pd(O_2CCF_3)_2$ (0.80 mg, 2.45 µmol, 1 equiv.) from a freshly prepared stock solution in ACN was added in one portion. The concentration of peptide in solution was 4.08 mM. The vial was stirred at room temperature for 24 h then quenched with 3-MPA (5.5 µL, 25 equiv.). Reaction was analyzed via **HPLC Method A**. No conversion was observed.

H_2N-FWG-CO_2H peptide 1g. LCMS, m/z 409.1734 (calcd. [M+H^+] = 409.1876) Purity: > 99 % (HPLC analysis at 220 nm). Retention time in HPLC: 8.0 min, 7.5 min (reaction).

HPLC Trace of 1g

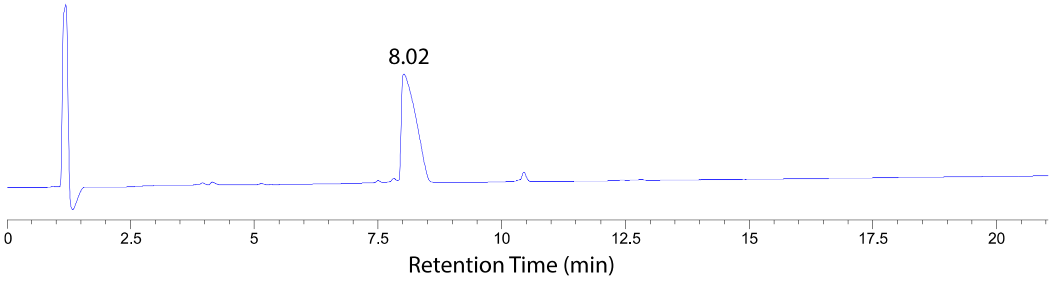

HPLC Trace of Reaction

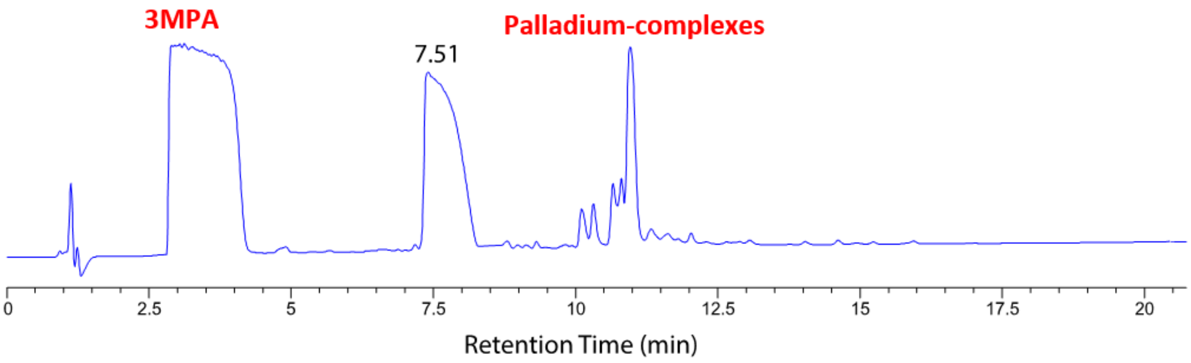

HRMS of 1g

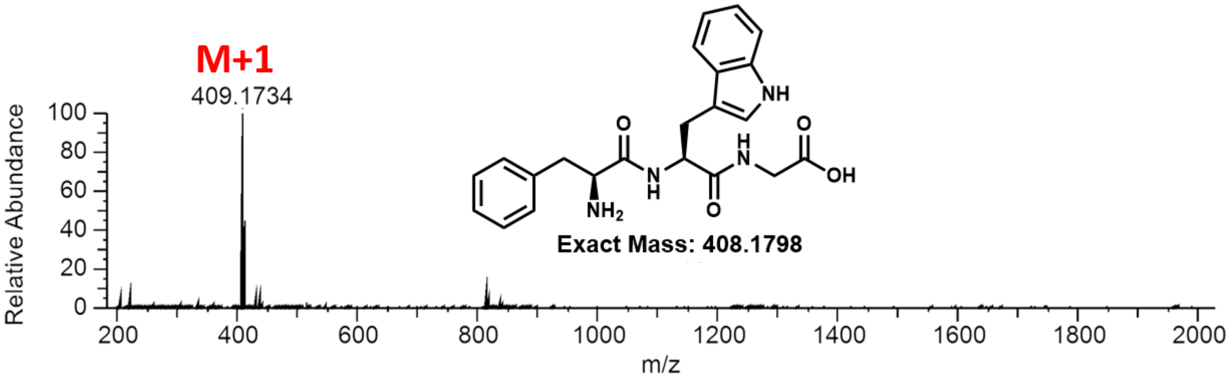

Reaction with Lysine Peptide H_2N-FKG-CO_2H 1h

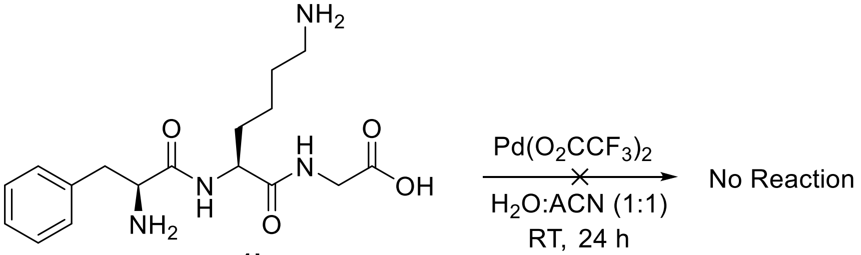

1h

H_2N-FKG-CO_2H (**1h**) (1.0 mg, 2.86 µmol, 1 equiv.) was dissolved in 600 µL of 1:1 H_2O:ACN in a 1/2" dram vial. Next, $Pd(O_2CCF_3)_2$ (0.95 mg, 2.86 µmol, 1 equiv.) from a freshly prepared stock solution in ACN was added in one portion. The concentration of peptide in solution was 4.76 mM. The vial was stirred at room temperature for 24 h then quenched with 3-MPA (6 µL, 25 equiv.). Reaction was analyzed via **HPLC Method A**. No conversion was observed.

H_2N-FKG-CO_2H peptide 1h. LCMS, m/z 351.1913 (calcd. $[M{+}H^{+}]$ = 351.2032) Purity: > 99 % (HPLC analysis at 220 nm). Retention time in HPLC: 1.9 min, 1.8 min (reaction).

HPLC Trace of 1h

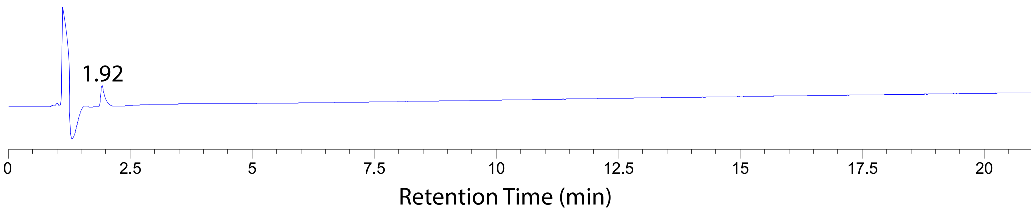

HPLC Trace of Reaction

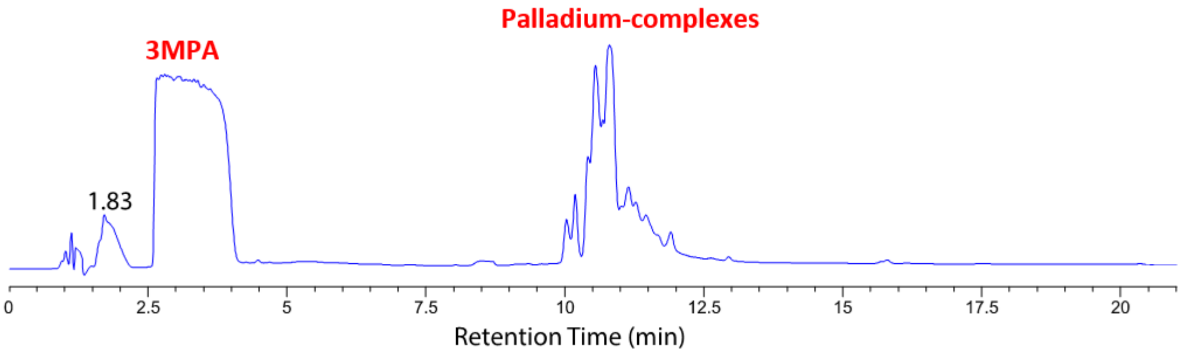

HRMS of 1h

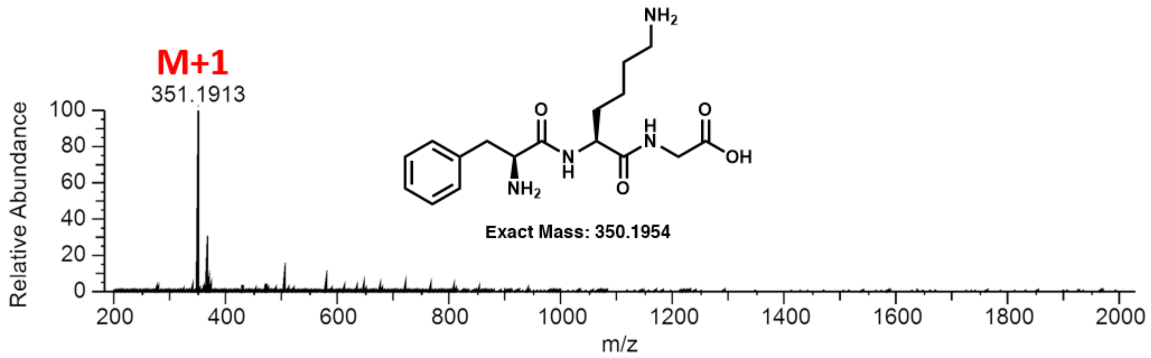

Reaction with Histidine Peptide H_2N-FHG-CO_2H 1i

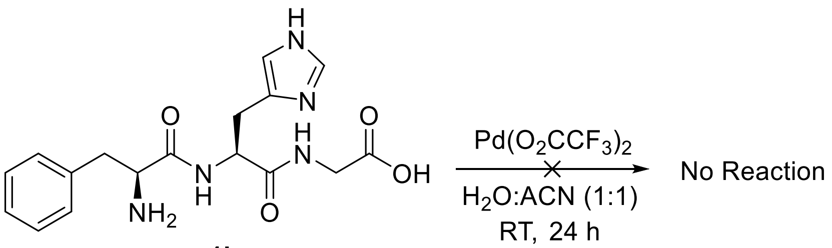

1i

H_2N-FHG-CO_2H (**1i**) (1.0 mg, 2.78 µmol, 1 equiv.) was dissolved in 600 µL of 1:1 H_2O:ACN in a 1/2" dram vial. Next, $Pd(O_2CCF_3)_2$ (0.93 mg, 2.78 µmol, 1 equiv.) from a freshly prepared stock solution in ACN was added in one portion. The concentration of peptide in solution was 4.64 mM. The vial was stirred at room temperature for 24 h then quenched with 3-MPA (6 µL, 25 equiv.). Reaction was analyzed via **HPLC Method A**. No conversion was observed.

H_2N-FHG-CO_2H peptide 1i. LCMS, m/z 360.1757 (calcd. [M+H^+] = 360.1672) Purity: > 99 % (HPLC analysis at 220 nm). Retention time in HPLC: 2.0 min, 1.7 min (reaction).

HPLC Trace of 1i

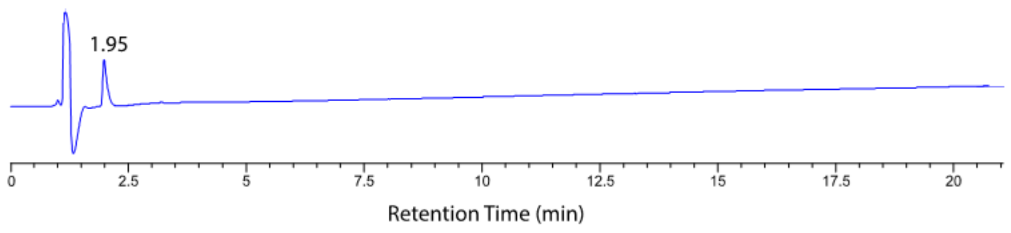

HPLC Trace of Reaction

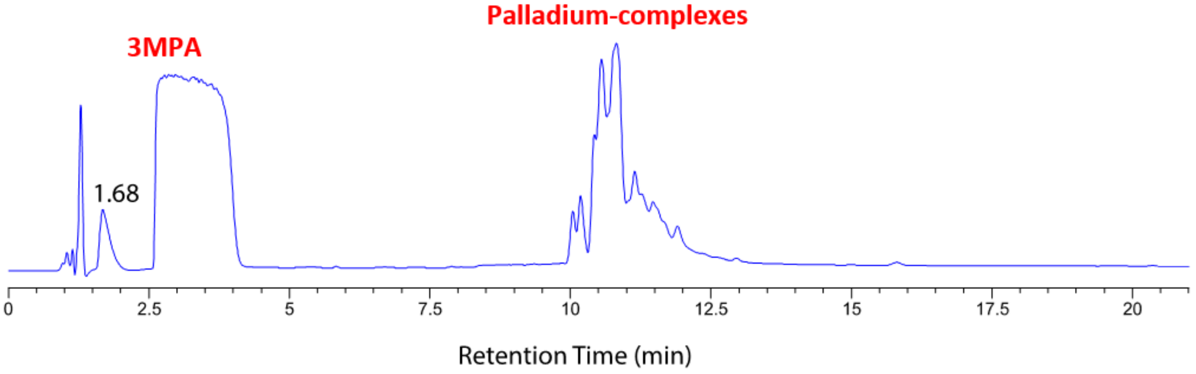

HRMS of 1i

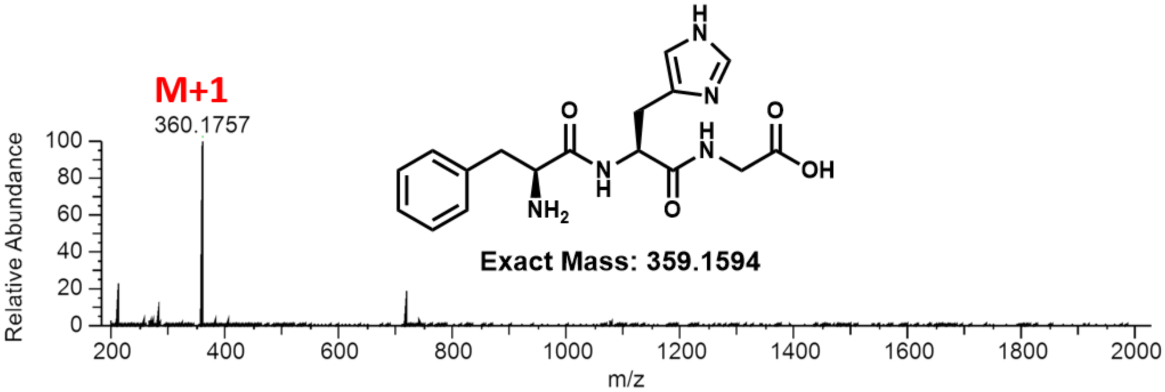

Reaction with Serine Peptide H_2N-FSG-CO_2H 1j

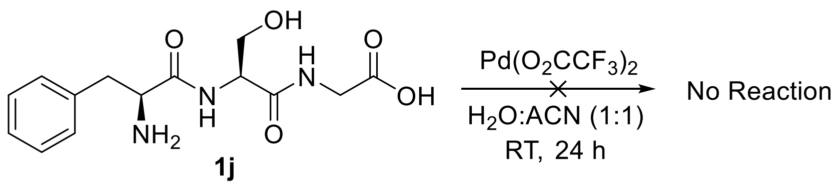

H_2N-FSG-CO_2H (**1j**) (1.0 mg, 3.2 µmol, 1 equiv.) was dissolved in 600 µL of 1:1 H_2O:ACN in a 1/2” dram vial. Next, $Pd(O_2CCF_3)_2$ (1.08 mg, 3.2 µmol, 1 equiv.) from a freshly prepared stock solution in ACN was added in one portion. The concentration of peptide in solution was 5.33 mM.

The vial was stirred at room temperature for 24 h then quenched with 3-MPA (6.5 µL, 25 equiv.). Reaction was analyzed via **HPLC Method C**. No conversion was observed.

H_2N-FSG-CO_2H peptide 1j. LCMS, m/z 310.1294 (calcd. [$M+H^+$] = 310.1403) Purity: > 99 % (HPLC analysis at 220 nm). Retention time in HPLC: 7.5 min, 7.4 min (reaction).

HPLC Trace of 1j

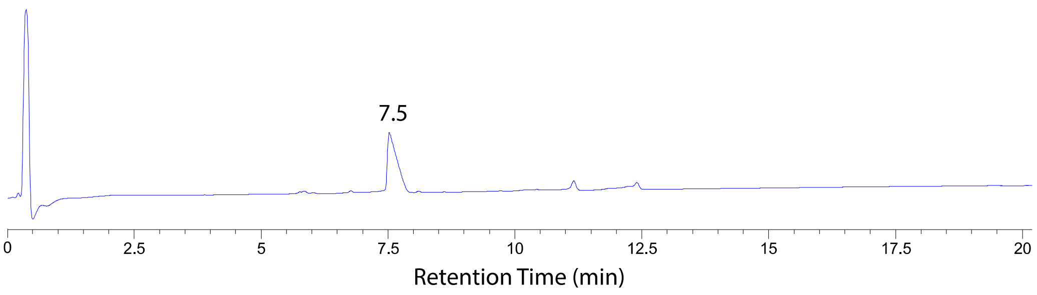

HPLC Trace of Reaction

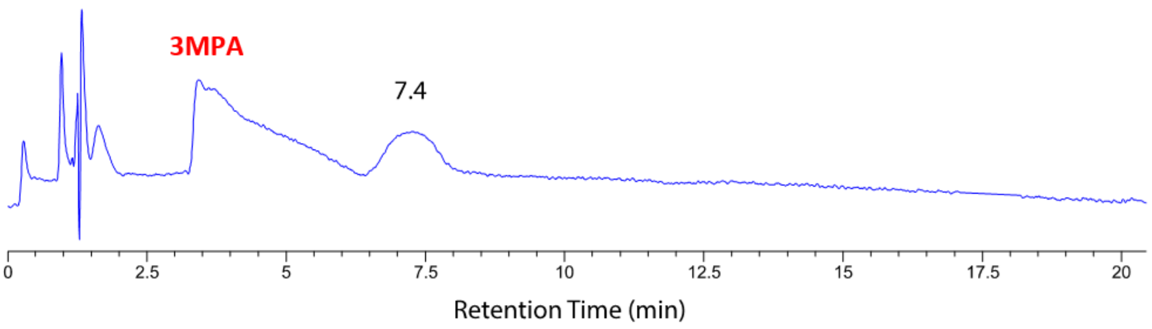

HRMS of 1j

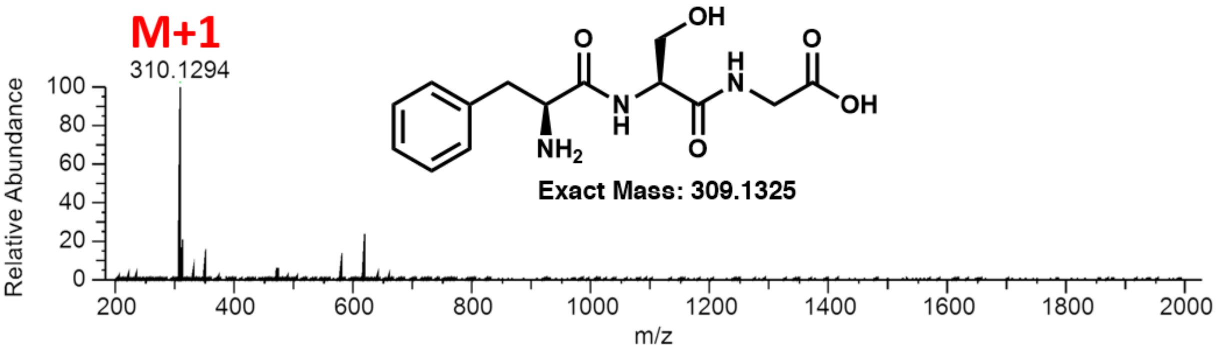

Reaction with Methionine Peptide H_2N-FMG-CO_2H 1k

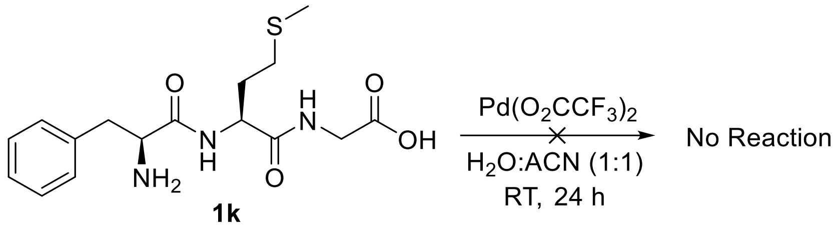

H_2N-FMG-CO_2H (**1k**) (1.0 mg, 2.8 µmol, 1 equiv.) was dissolved in 600 µL of 1:1 H_2O:ACN in a 1/2" dram vial. Next, $Pd(O_2CCF_3)_2$ (0.94 mg, 2.8 µmol, 1 equiv.) from a freshly prepared stock solution in ACN was added in one portion. The concentration of peptide in solution was 4.67 mM. The vial was stirred at room temperature for 24 h then quenched with 3-MPA (6 µL, 25 equiv.). Reaction was analyzed via **HPLC Method A**. No conversion was observed.

H_2N-FWG-CO_2H peptide 1k. LCMS, m/z 354.1569 (calcd. [M+H^+] = 354.1488), m/z 707.3066 (calcd. [$2M$+H^+] = 707.2897) Purity: > 99 % (HPLC analysis at 220 nm). Retention time in HPLC: 5.1 min, 6.1 min (reaction).

HPLC Trace of 1k

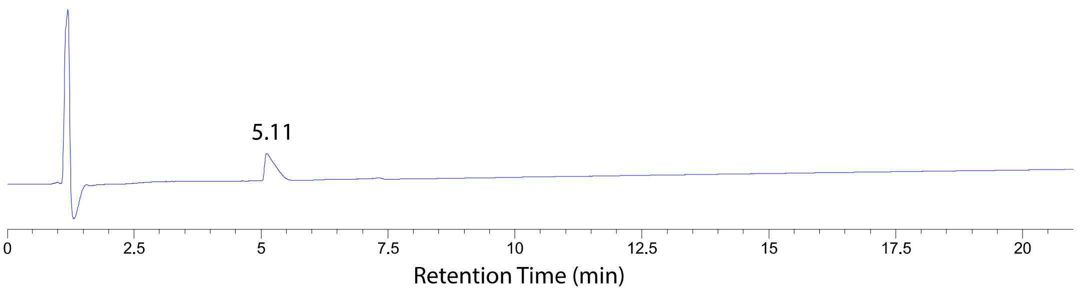

HPLC Trace of Reaction

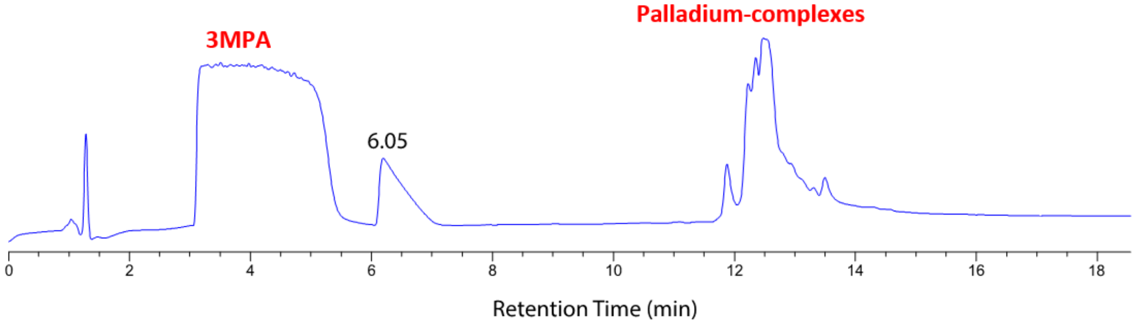

HRMS of 1k

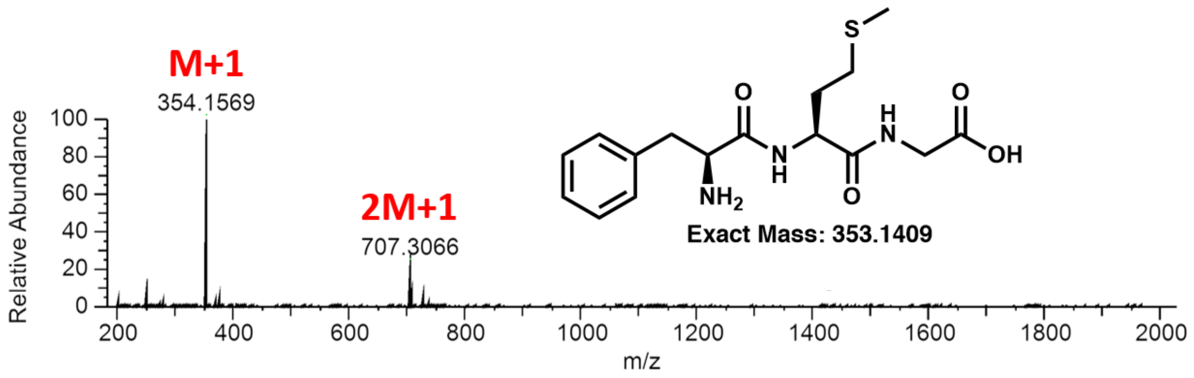

Reaction with Proline Peptide H_2N-FPG-CO_2H 1l

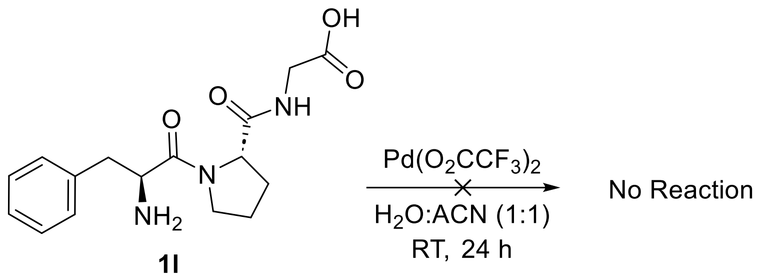

H_2N-FPG-CO_2H (**1l**) (1.0 mg, 3.1 µmol, 1 equiv.) was dissolved in 600 µL of 1:1 H_2O:ACN in a 1/2" dram vial. Next, $Pd(O_2CCF_3)_2$ (1.04 mg, 3.1 µmol, 1 equiv.) from a freshly prepared stock solution in ACN was added in one portion. The concentration of peptide in solution was 5.17 mM. The vial was stirred at room temperature for 24 h then quenched with 3-MPA (6.5 µL, 25 equiv.). Reaction was analyzed via **HPLC Method B**. No conversion was observed.

H_2N-FPG-CO_2H peptide 1l. LCMS, m/z 320.1496 (calcd. $[M+H^+]$ = 320.1610), m/z 342.1308 (calcd. [M+Na+] = 342.1430), m/z 639.2923 (calcd. $[2M+H^+]$ = 639.3142) Purity: > 99 % (HPLC analysis at 220 nm). Retention time in HPLC: 8.2 min, 8.3 (reaction).

HPLC Trace of 1l

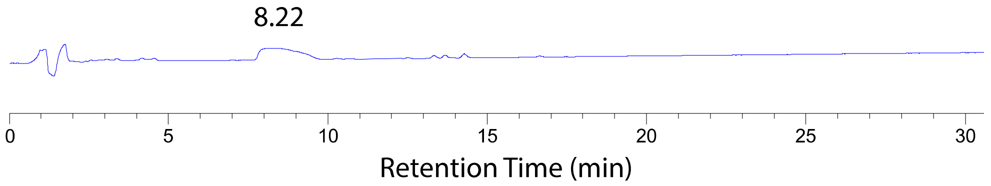

HPLC Trace of Reaction

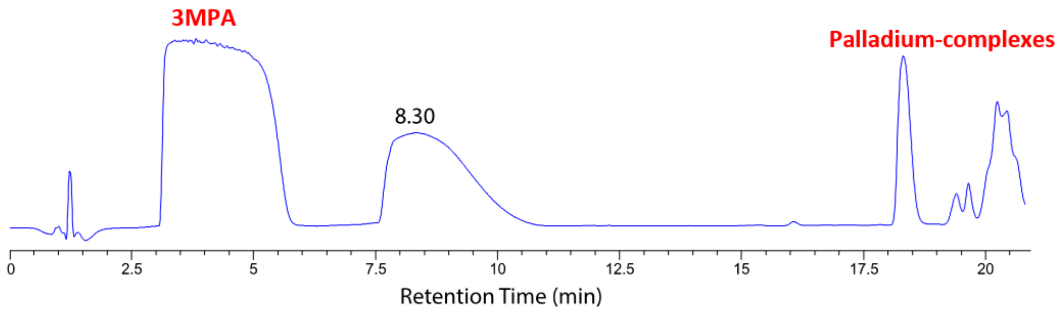

HRMS of 1l

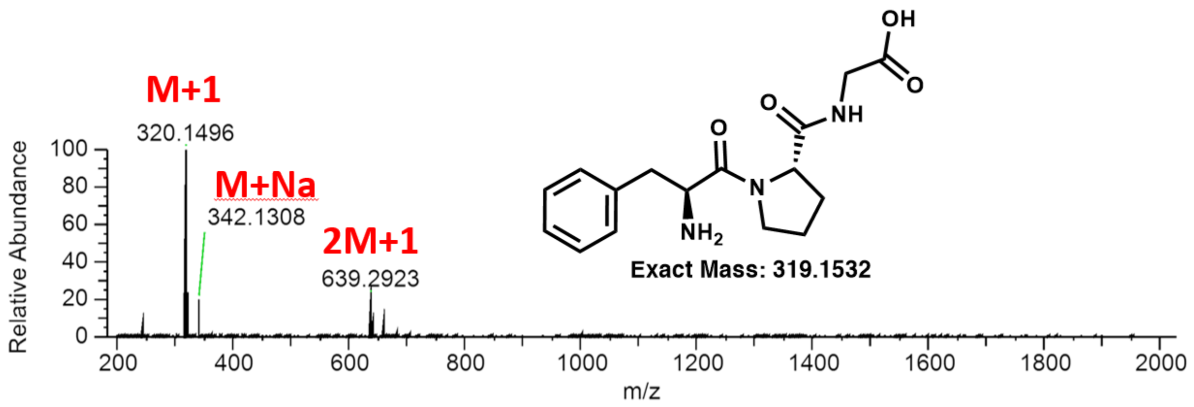

Reaction with Asparagine Peptide H_2N-FNG-CO_2H 1m

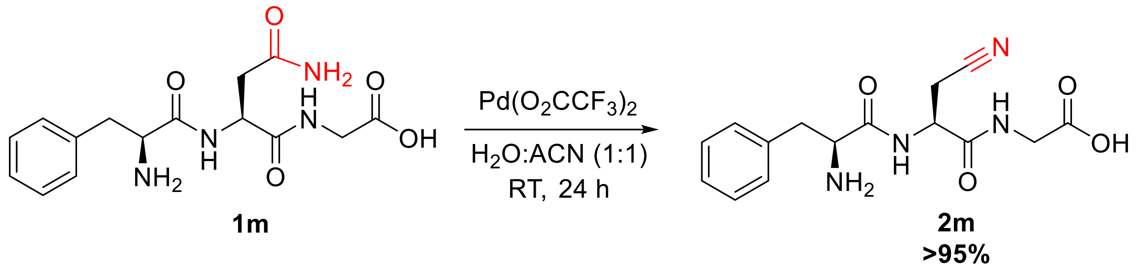

H_2N-FNG-CO_2H (**1m**) (1.0 mg, 3.0 µmol, 1 equiv.) was dissolved in 600 µL of 1:1 H_2O:ACN in a 1/2" dram vial. Next, $Pd(O_2CCF_3)_2$ (0.99 mg, 3.0 µmol, 1 equiv.) from a freshly prepared stock solution in ACN was added in one portion. The concentration of peptide in solution was 4.96 mM. The vial was stirred at room temperature for 24 h then quenched with ethylene diamine (5 µL, 28 equiv.). The reaction was analyzed via **HPLC Method A**, revealing >95% conversion to nitrile product **2m**.

H_2N-FNG-CO_2H peptide 1m. LCMS, m/z 337.1588 (calcd. $[M+H^+]$ = 337.1512), m/z 673.3103 (calcd. [2M+H+] = 673.2946) Purity: > 99 % (HPLC analysis at 220 nm). Retention time in HPLC: 3.3 min.

H_2N-FN(Nitrile)G-CO_2H peptide 2m. LCMS, m/z 319.1399 (calcd. $[M+H^+]$ = 319.1406), m/z 637.2725 (calcd. [M+H+] = 637.2734) Purity: > 99 % (HPLC analysis at 220 nm). Retention time in HPLC: 4.5 min.

HPLC Trace of 1m

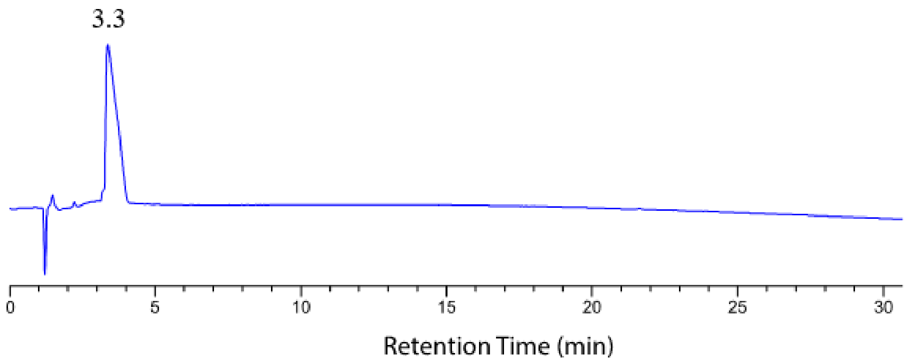

HPLC Trace of Reaction

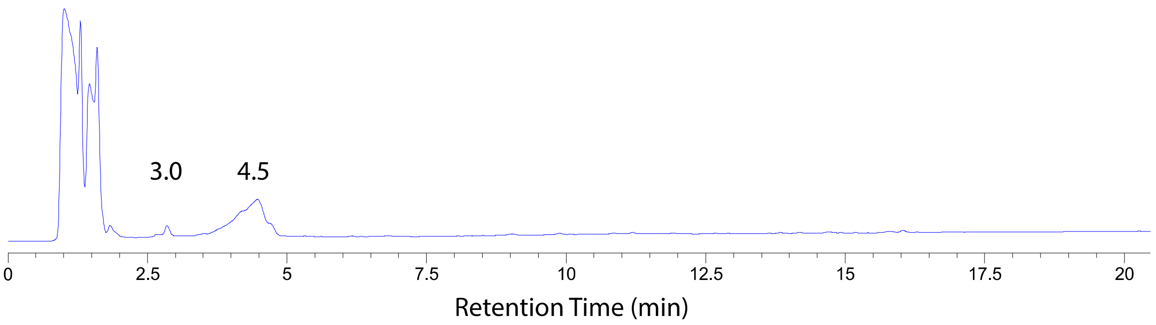

HRMS of 1m

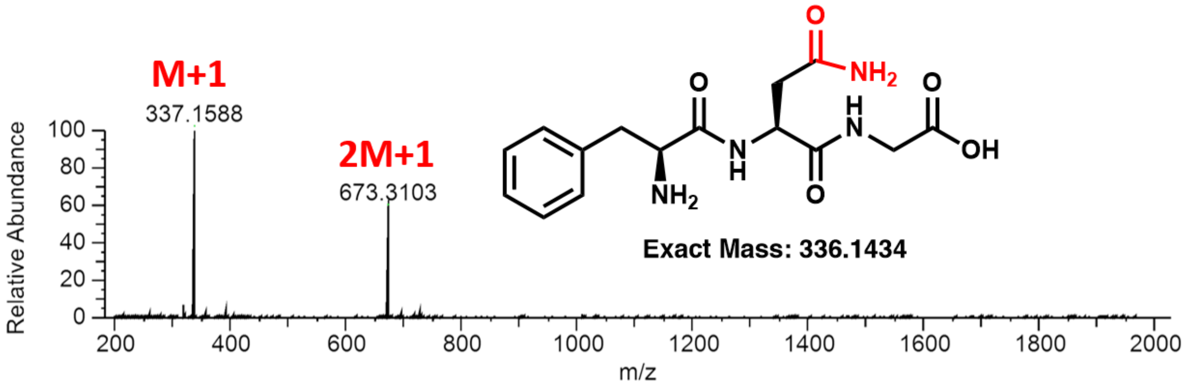

HRMS of 2m

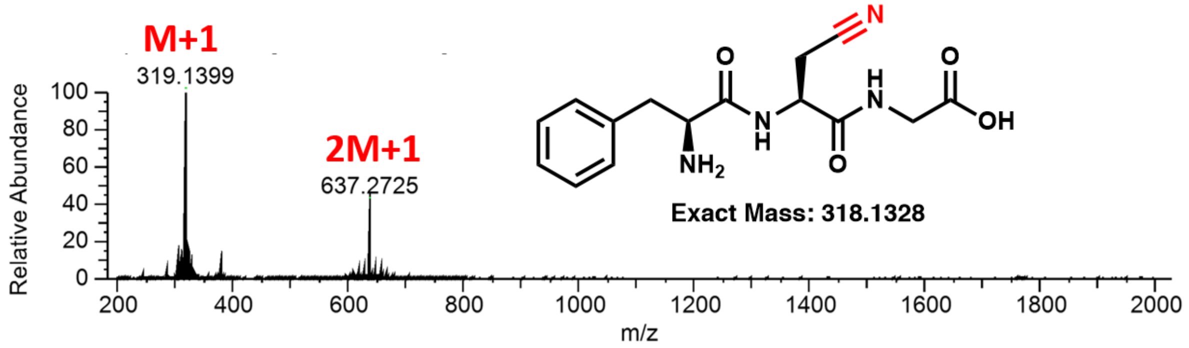

Supplementary Fig. 3: Evaluating Stability of Peptide Nitrile

1 mg of nitrile containing peptide FQ(Nitrile)G **2a** was dissolved in ACN:10 mM NaP buffer (1:4, 200 µL), and the pH was adjusted using 1 M NaOH or 1 M HCl to the following values: 3.0, 5.0, 7.0, 9.0, and 12.0. The reactions were incubated at RT for 24 h and analyzed using LC-MS. No degradation of peptide nitrile product was observed.

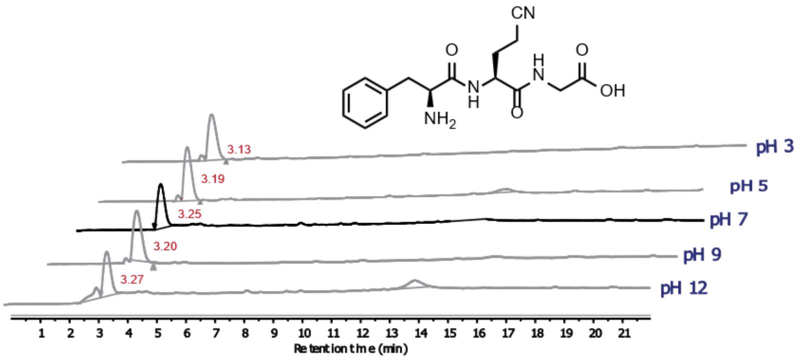

Supplementary Fig. 4: Computational Analysis of Nitrile Reactivity

Computations were performed in Gaussian 10 through GaussView interface. Ab initio calculations were performed at B3LYP/6-311+G(d,p) level. Below are the reported geometry optimizations of model amino acids (a) H_2N-Asp-CN (b) H_2N-Asn(CN)-CO_2H (c) H_2N-Glu-CN (d) H_2N-Gln(CN)-CO_2H. The grey, white, red, and blue balls represent the carbon, hydrogen, oxygen, and nitrogen atoms respectively.

(a) H_2N-Asp-CN – Ground State Minimization (left) and LUMO molecular orbitals (right)

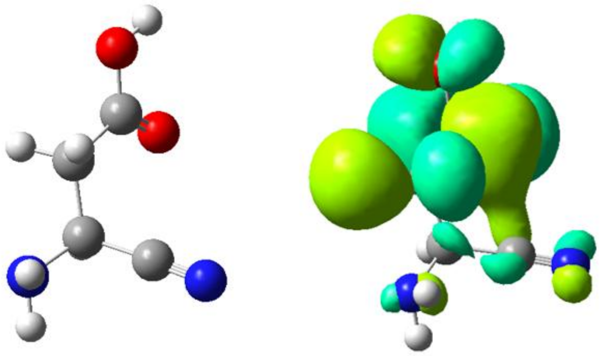

(b) H_2N-Asn(CN)-CO_2H – Ground State Minimization (left) and LUMO molecular orbitals (right)

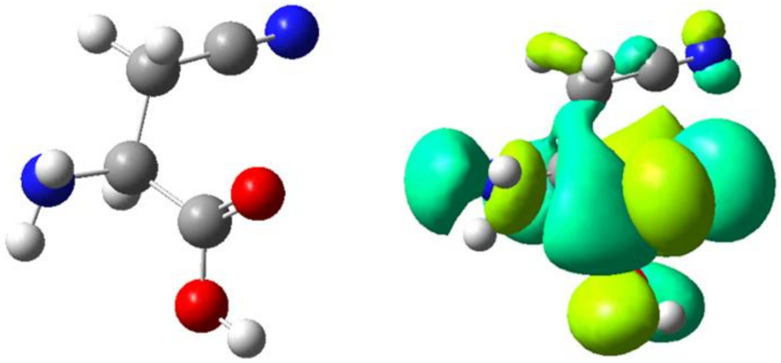

(c) H_2N-Glu-CN – Ground State Minimization (left) and LUMO molecular orbitals (right)

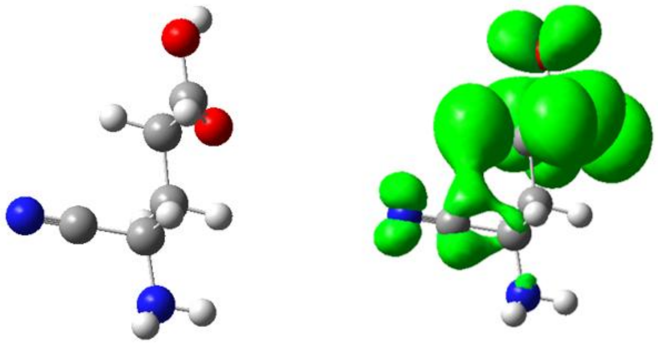

(d) H_2N-Gln(CN)-CO_2H – Ground State Minimization (left) and LUMO molecular orbitals (right)

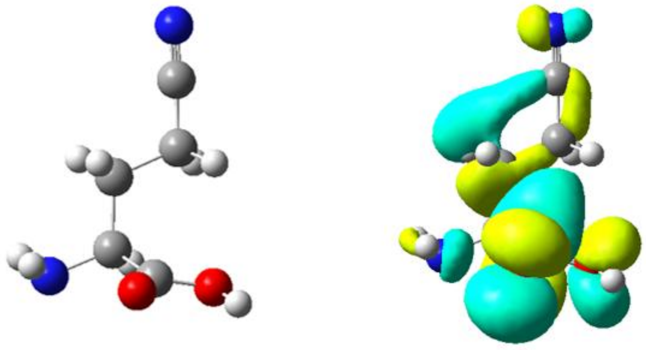

Geometry Optimizations

Cartesian coordinates and total energies for geometry optimized model peptides obtained at B3LYP/6-311+G(d, p) level.

Geometry optimized H_2N-Asp-CN

Center	Atomic	Atomic	Coordinates (Angstroms)		
Number	Number	Type	X	Y	Z
1	7	0	-2.278957	-1.374790	0.052510
2	1	0	-1.537737	-1.473597	0.742762
3	6	0	-2.791294	-0.006557	0.012490
4	1	0	-3.516434	0.061459	-0.804498
5	6	0	-3.509037	0.320162	1.332454
6	1	0	-4.218784	-0.489650	1.534652
7	1	0	-2.808237	0.340154	2.173031
8	1	0	-1.895610	-1.643734	-0.849766
9	6	0	-1.735052	1.000898	-0.282619
10	7	0	-0.871938	1.742332	-0.520657
11	6	0	-4.282563	1.617697	1.296992
12	8	0	-4.492389	2.286406	0.301549
13	8	0	-4.746255	1.950471	2.516103
14	1	0	-5.261227	2.773626	2.438778

LUMO Energy = -0.02968 Hartree

Geometry optimized H_2N-Asn(CN)-CO_2H

Center	Atomic	Atomic	Coordinates (Angstroms)		
Number	Number	Type	X	Y	Z

1	7	0	-3.877378	-0.153285	1.489917
2	1	0	-3.126997	-0.227402	2.174004
3	6	0	-4.491402	1.176660	1.507948
4	1	0	-5.166522	1.240877	0.650611
5	6	0	-5.299639	1.328822	2.811955
6	1	0	-5.995670	0.488180	2.882940
7	1	0	-4.632522	1.288619	3.679314
8	1	0	-3.483048	-0.356942	0.575515
9	6	0	-3.441662	2.286034	1.371035
10	8	0	-2.950475	2.905723	2.296701
11	8	0	-3.070525	2.456229	0.088840
12	1	0	-2.353734	3.114956	0.052731
13	6	0	-6.064529	2.575070	2.879714
14	7	0	-6.678352	3.560395	2.926009

LUMO Energy = -0.02121 Hartree

Geometry optimized H_2N-Glu-CN

Center Number	Atomic Number	Atomic Type	Coordinates (Angstroms) X	Y	Z
1	7	0	-1.884268	-2.422697	0.220111
2	1	0	-1.347263	-2.587662	-0.628655
3	6	0	-2.694169	-1.202633	0.080621
4	1	0	-3.307614	-1.194356	-0.834444
5	6	0	-3.628854	-1.050458	1.298858
6	1	0	-4.232955	-1.961886	1.352673
7	1	0	-3.022936	-1.009397	2.208573
8	6	0	-4.547434	0.167813	1.206442
9	1	0	-3.976666	1.103423	1.170828
10	1	0	-5.147172	0.146039	0.288041
11	1	0	-2.510389	-3.218688	0.324868
12	6	0	-1.779940	-0.045078	-0.032075
13	7	0	-1.067696	0.867374	-0.129473
14	6	0	-5.507674	0.268840	2.368552
15	8	0	-5.601467	-0.521859	3.289902
16	8	0	-6.285126	1.369035	2.276260
17	1	0	-6.885187	1.386546	3.042891

LUMO Energy = -0.02415 Hartree

Geometry optimized H_2N-Gln(CN)-CO_2H

Center Number	Atomic Number	Atomic Type	Coordinates (Angstroms) X	Y	Z
1	7	0	-0.385299	-0.590185	0.296236
2	1	0	0.255546	-0.675732	1.082565
3	6	0	-1.047290	0.713423	0.321987
4	1	0	-1.689853	0.789692	-0.560871
5	6	0	-1.915570	0.948522	1.589272
6	1	0	-2.585261	0.087559	1.679023
7	1	0	-1.270226	0.955143	2.474309
8	6	0	-2.762508	2.240651	1.540996
9	1	0	-2.132181	3.134032	1.487708
10	1	0	-3.403899	2.247334	0.652311
11	1	0	-1.076298	-1.331094	0.383254
12	6	0	0.028541	1.787637	0.214556
13	8	0	1.126379	1.717758	0.734237
14	8	0	-0.381077	2.856529	-0.495163
15	1	0	0.327076	3.526115	-0.488677
16	6	0	-3.623246	2.371422	2.718055
17	7	0	-4.301201	2.464011	3.657691

LUMO Energy = -0.01549 Hartree

Supplementary Fig. 5: Exploration of Nitrile Modification Methods

Supplementary Fig. 5a: Peptide Level Modification – Asn, Gln, and C-Terminal Nitrile Comparison

Synthesis of H_2N-WGQFL-CO_2H 1n

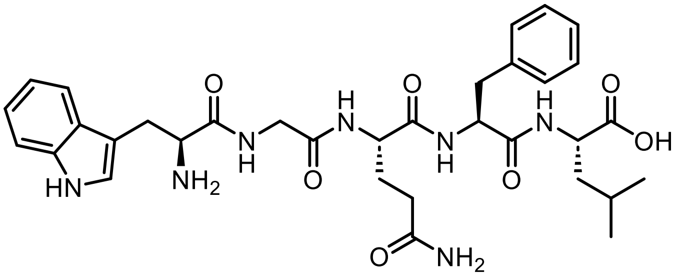

H_2N-WGQFL-CO_2H (1n): LCMS, *m/z* 650.3295 (calcd. [M+H$^+$] = 650.3297), *m/z* 1299.6516 (calcd. [2M+H$^+$] = 1299.6521), Purity: >99% (HPLC analysis at 220 nm). Retention time using **HPLC Method A**: 11.0 min.

HPLC Trace for H_2N-WGQFL-CO_2H 1n

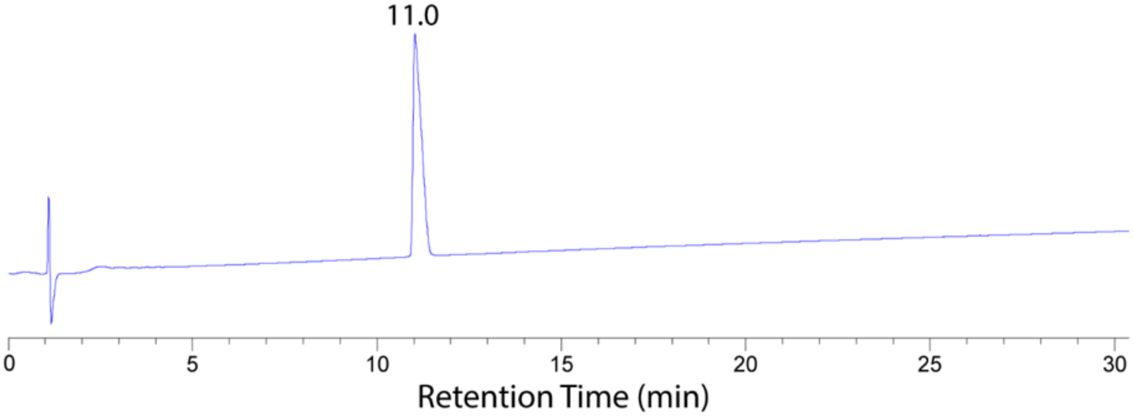

HRMS of H_2N-WGQFL-CO_2H 1n

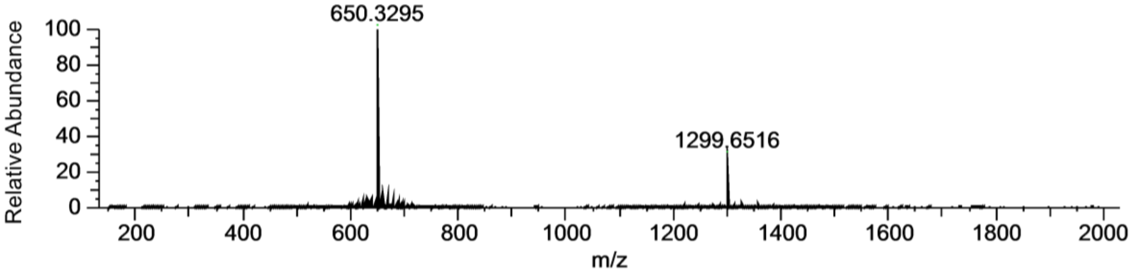

Synthesis of H_2N-WGNFL-CO_2H 1o

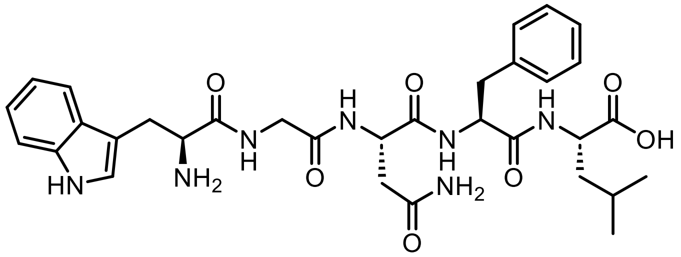

H_2N-WGNFL-CO_2H (1o): LCMS, *m/z* 636.3138 (calcd. [$M+H^+$] = 636.3140), *m/z* 1271.6204 (calcd. [$2M+H^+$] = 1271.6207), Purity: >99% (HPLC analysis at 220 nm). Retention time using **HPLC Method A**: 11.1 min.

HPLC Trace for H_2N-WGNFL-CO_2H 1o

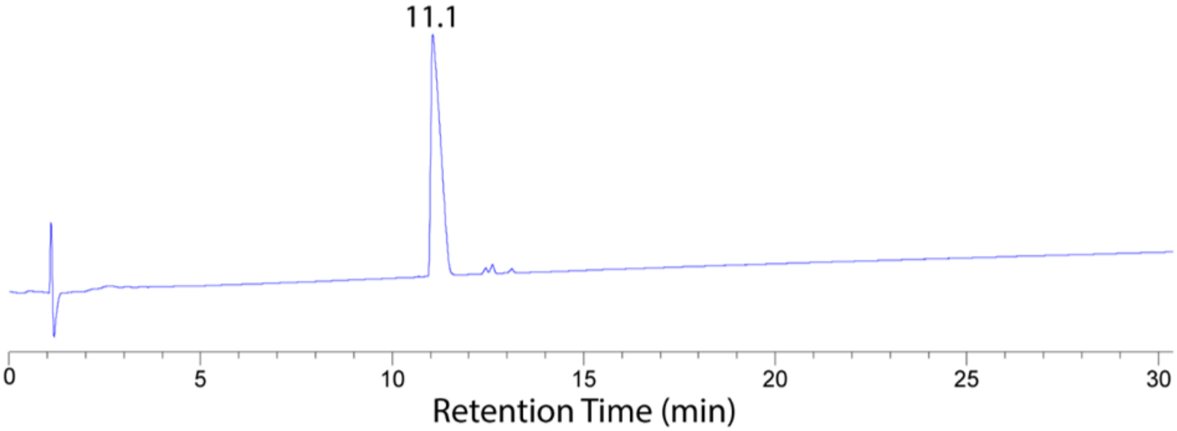

HRMS of H_2N-WGNFL-CO_2H 1o

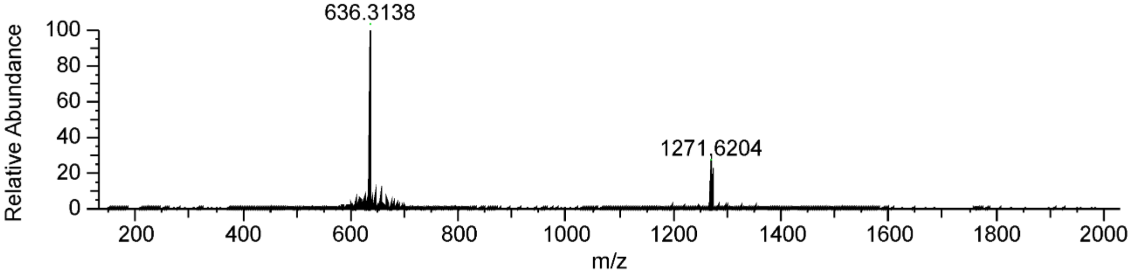

Synthesis of H_2N-WGDFL-$CONH_2$ 1p

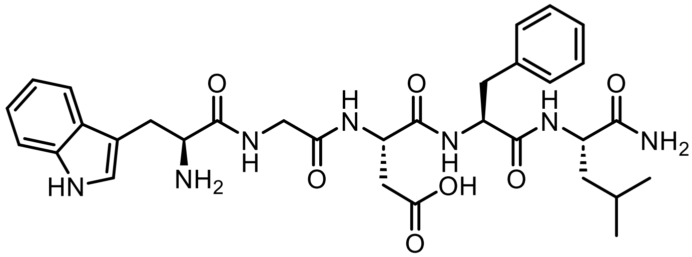

H_2N-WGDFL-$CONH_2$ (1p): LCMS, *m/z* 636.3139 (calcd. [*M*+H^+] = 636.3140), *m/z* 1271.6205 (calcd. [2M+H^+] = 1271.6207), Purity: >99% (HPLC analysis at 220 nm). Retention time using **HPLC Method A**: 10.7 min.

HPLC Trace for H_2N-WGDFL-$CONH_2$ 1p

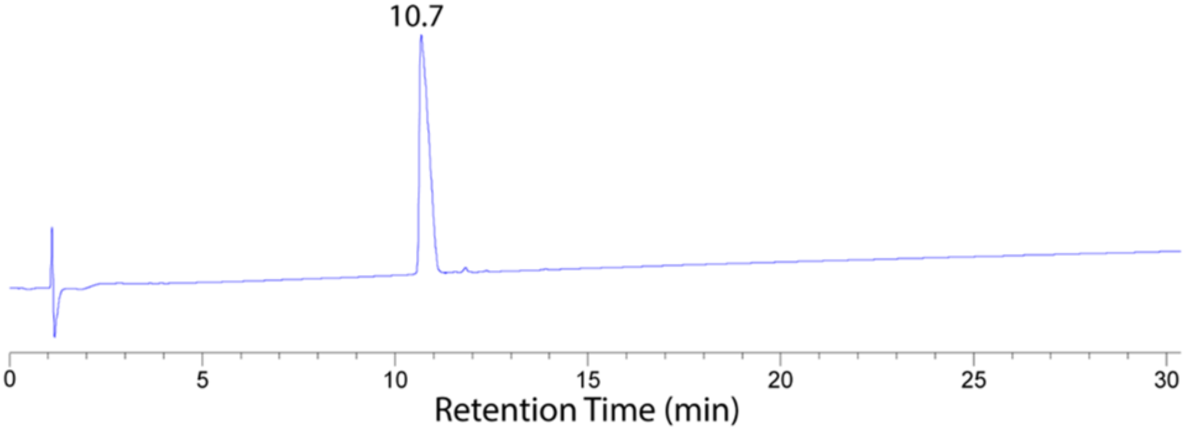

HRMS of H_2N-WGDFL-$CONH_2$ 1p

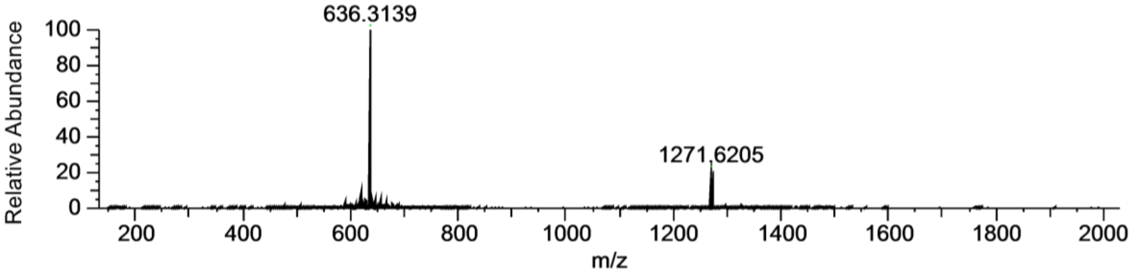

Synthesis of H_2N-WGQ(Nitrile)FL-CO_2H 2n

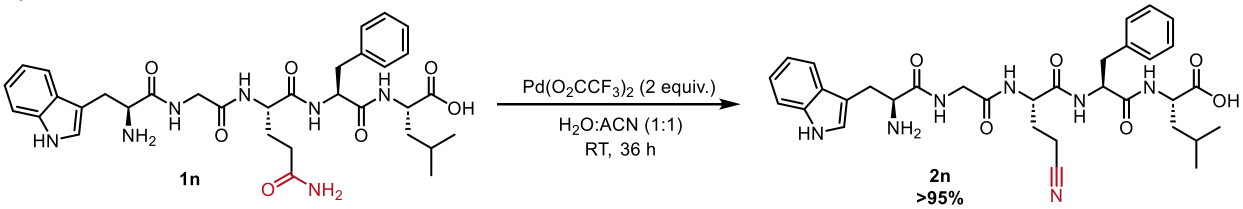

H_2N-WGQFL-CO_2H **1n** (20 mg, 30.8 µmol, 1 equiv.) was dissolved in 1.5 mL of 1:1 H_2O:ACN in a 1" dram vial. Next, $Pd(O_2CCF_3)_2$ (20.5 mg, 61.6 µmol, 2 equiv.) was added in one portion. The vial was stirred at room temperature for 36 hours then quenched with 3-MPA (53.6 µL, 20 equiv.). The reaction was analyzed via **HPLC Method A**, revealing >95% conversion to nitrile product **2n**. Fractions were then collected via preparative HPLC and lyophilized to produce pure H_2N-WGQ(Nitrile)FL-CO_2H **2n** as a fluffy white-yellow powder (10.8 mg, 56% yield).

H_2N-WGQ(Nitrile)FL-CO_2H 2n. LCMS, m/z 632.3185 (calcd. $[M+H^+]$ = 632.3191), m/z 1263.6295 (calcd. $[2M+H^+]$ = 1263.6309) Purity: >99% (HPLC analysis at 220 nm). Retention time in HPLC: 12.1 min.

HPLC Trace for H_2N-WGQ(Nitrile)FL-CO_2H 2n

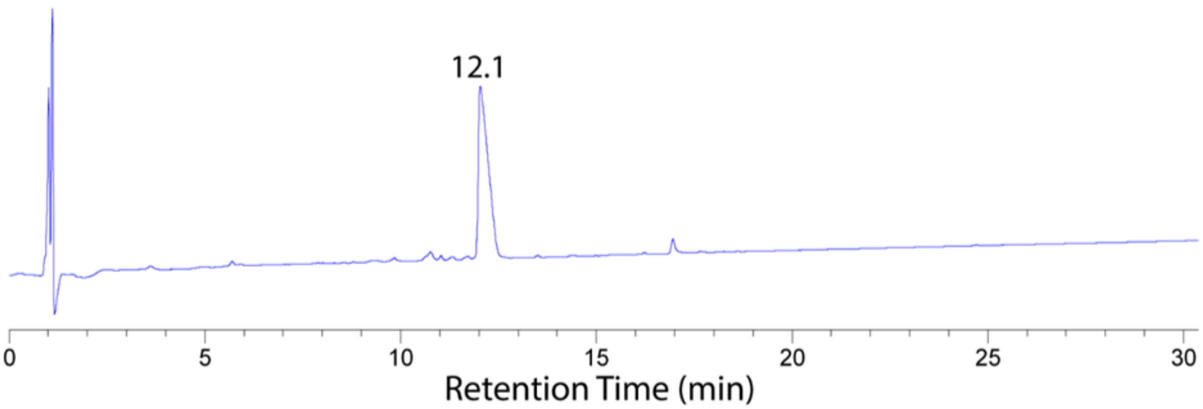

HRMS of H_2N-WGQ(Nitrile)FL-CO_2H 2n

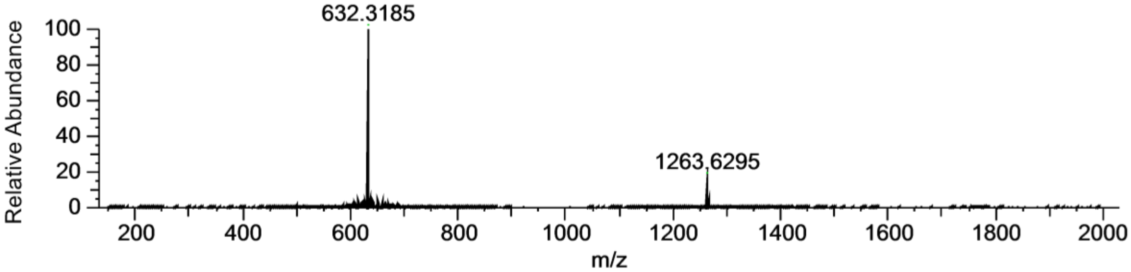

Synthesis of H_2N-WGN(Nitrile)FL-CO_2H 2o

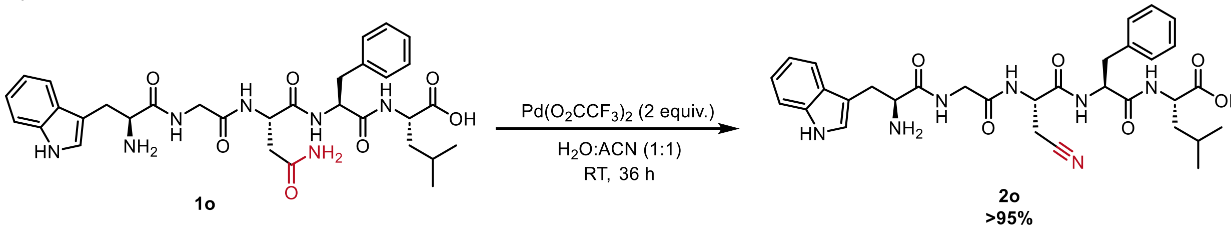

H_2N-WGNFL-CO_2H **1o** (20 mg, 31.5 µmol, 1 equiv.) was dissolved in 1.5 mL of 1:1 H_2O:ACN in a 1” dram vial. Next, $Pd(O_2CCF_3)_2$ (20.9 mg, 62.9 µmol, 2 equiv.) was added in one portion. The vial was stirred at room temperature for 36 hours then quenched with 3-MPA (54.8 µL, 20 equiv.). The reaction was analyzed via **HPLC Method A**, revealing >95% conversion to nitrile product **2o**. Fractions were then collected via preparative HPLC and lyophilized to produce pure H_2N-WGN(Nitrile)FL-CO_2H **2o** as a fluffy white-yellow powder (11.4 mg, 59% yield).

H_2N-WGN(Nitrile)FL-CO_2H 2o. LCMS, m/z 618.3034 (calcd. $[M+H^+]$ = 618.3035), m/z 1235.5995 (calcd. $[2M+H^+]$ = 1235.5997) Purity: >99% (HPLC analysis at 220 nm). Retention time in HPLC: 12.2 min.

HPLC Trace for H_2N-WGN(Nitrile)FL-CO_2H 2o

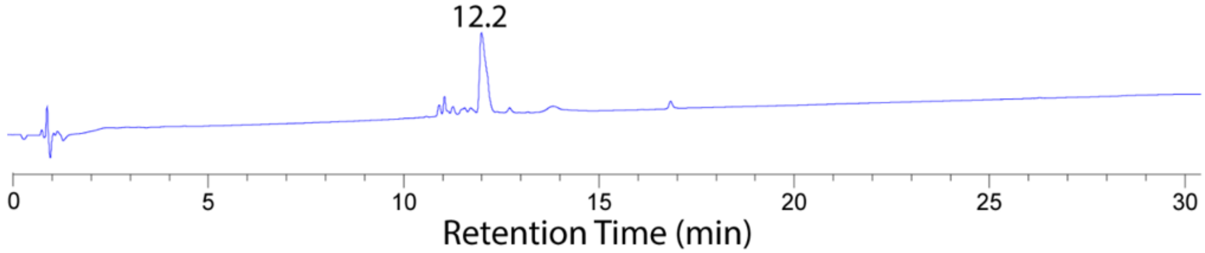

HRMS of H_2N-WGN(Nitrile)FL-CO_2H 2o

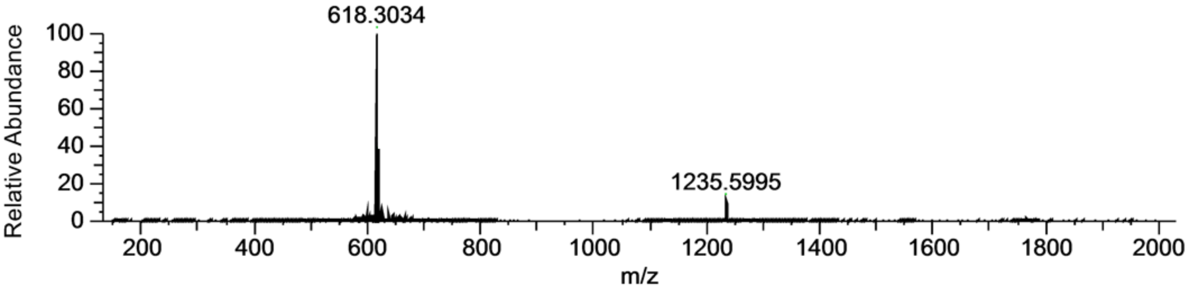

Synthesis of H_2N-WGDFL-(Nitrile) 2p

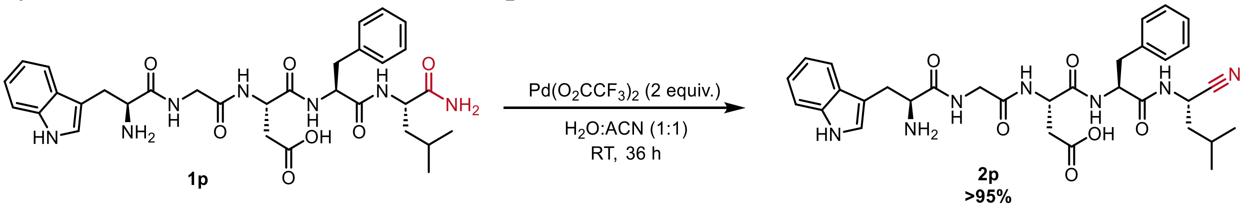

H_2N-WGDFL-$CONH_2$ **1p** (20 mg, 31.5 μmol, 1 equiv.) was dissolved in 1.5 mL of 1:1 H_2O:ACN in a 1” dram vial. Next, $Pd(O_2CCF_3)_2$ (20.9 mg, 62.9 μmol, 2 equiv.) was added in one portion. The vial was stirred at room temperature for 36 hours then quenched with 3-MPA (54.8 μL, 20 equiv.). The reaction was analyzed via **HPLC Method A**, revealing >95% conversion to nitrile product **2p**. Fractions were then collected via preparative HPLC and lyophilized to produce pure H_2N-WGDFL-(Nitrile) **2p** as a fluffy white-yellow powder (11.9 mg, 61% yield).

H_2N-WGDFL-(Nitrile) 2p. LCMS, m/z 618.3029 (calcd. [$M+H^+$] = 618.3035), m/z 1235.5986 (calcd. [$2M+H^+$] = 1235.5997) Purity: >99% (HPLC analysis at 220 nm). Retention time in HPLC: 12.8 min.

HPLC Trace for H_2N-WGDFL-(Nitrile) 2p

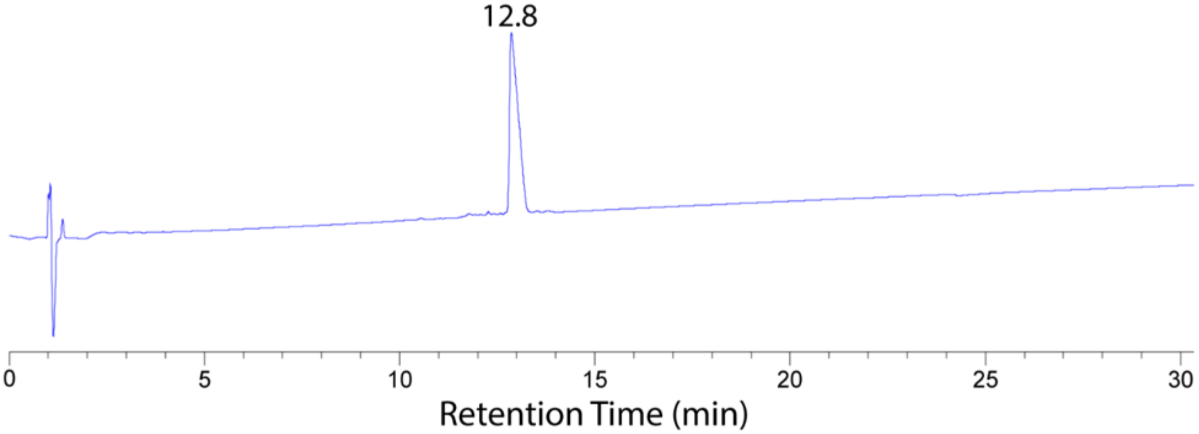

HRMS of H_2N-WGDFL-(Nitrile) 2p

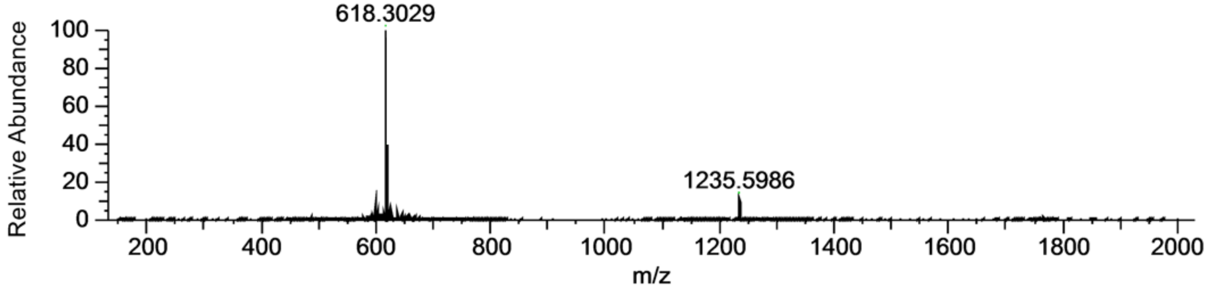

Reacting H_2N-WGQ(Nitrile)FL-CO_2H 2n with *N*-Methylhydroxylamine

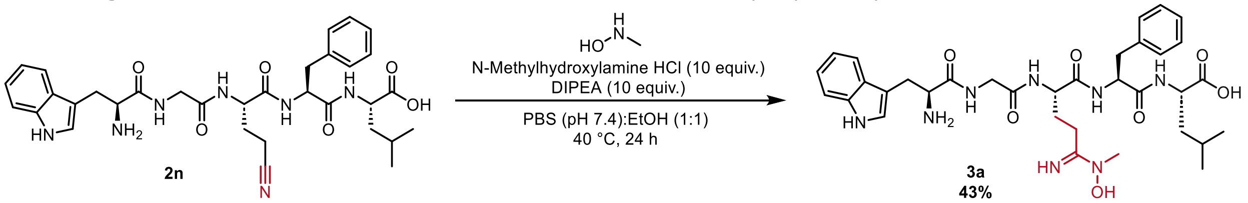

H_2N-WGQ(Nitrile)FL-CO_2H **2n** (1 mg, 1.6 µmol, 1 equiv.) was dissolved in 500 µL of 1:1 PBS (pH 7.4):EtOH in a 1/2” dram vial. Next, *N*-methylhydroxylamine HCl (1.32 mg, 15.8 µmol, 10 equiv.) was added in one portion along with DIPEA (2.8 µL, 15.8 µmol, 10 equiv.). The vial was stirred at 40 °C for 24 hours. The reaction was then analyzed via **HPLC Method A**, revealing 43% conversion to hydroxylamine-modified product **3a**.

Hydroxylamine-Modified H_2N-WGQFL-CO_2H 3a. LCMS, m/z 679.3559 (calcd. $[M+H^+]$ = 679.3562), m/z 340.1816 (calcd. $[(M+2H^+)/2]$ = 340.1818) Purity: >99% (HPLC analysis at 220 nm). Retention time in HPLC: 9.7 min.

H_2N-WGQ(Nitrile)FL-CO_2H 2n. LCMS, m/z 632.3185 (calcd. $[M+H^+]$ = 632.3191), m/z 1263.6295 (calcd. $[2M+H^+]$ = 1263.6309) Purity: >99% (HPLC analysis at 220 nm). Retention time in HPLC: 12.0 min.

HPLC Trace for Formation of Modified H_2N-WGQFL-CO_2H 3a

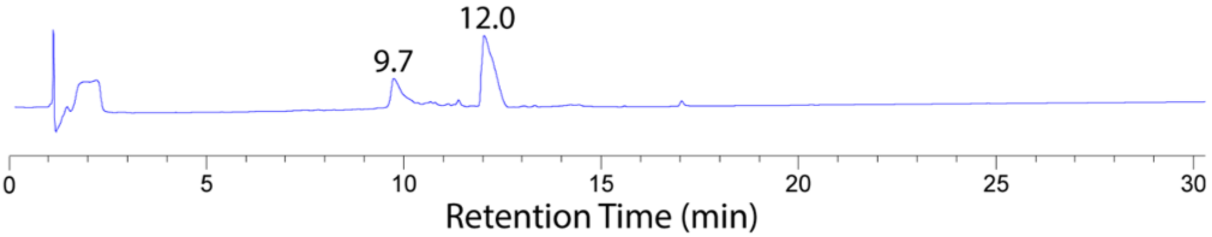

HRMS of Modified H_2N-WGQFL-CO_2H 3a

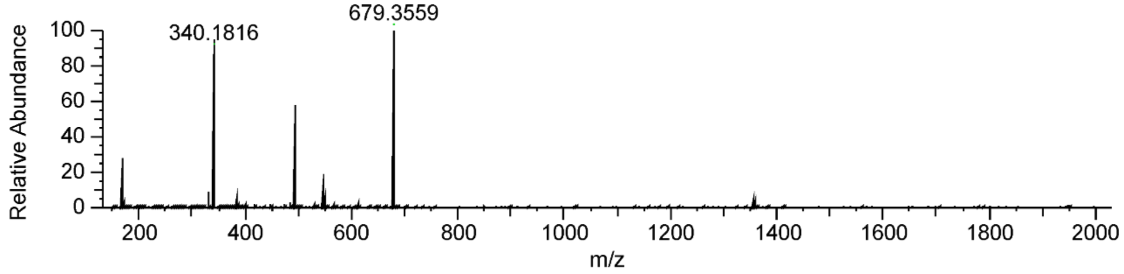

Reacting H_2N-WGN(Nitrile)FL-CO_2H 2o with *N*-Methylhydroxylamine

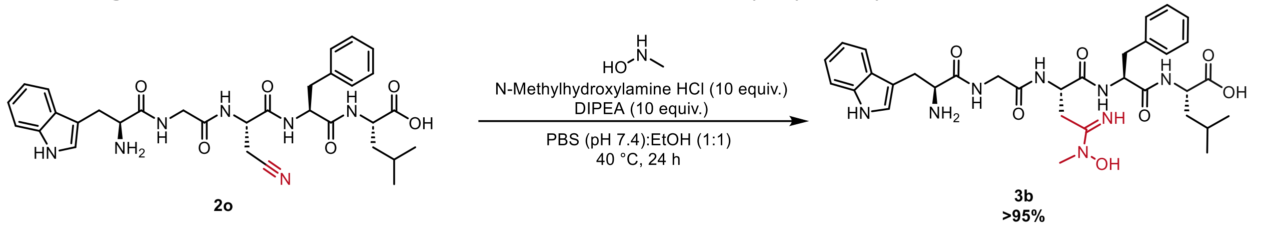

H_2N-WGN(Nitrile)FL-CO_2H **2o** (1 mg, 1.6 µmol, 1 equiv.) was dissolved in 500 µL of 1:1 PBS (pH 7.4):EtOH in a 1/2” dram vial. Next, *N*-methylhydroxylamine HCl (1.35 mg, 16.2 µmol, 10 equiv.) was added in one portion along with DIPEA (2.8 µL, 16.2 µmol, 10 equiv.). The vial was stirred at 40 °C for 24 hours. The reaction was then analyzed via **HPLC Method A**, revealing >95% conversion to hydroxylamine-modified product **3b**.

Hydroxylamine-Modified H_2N-WGNFL-CO_2H 3b. LCMS, m/z 665.3409 (calcd. [M+H^+] = 665.3406), m/z 333.1741 (calcd. [(M+2H^+)/2] = 333.1739) Purity: >99% (HPLC analysis at 220 nm). Retention time in HPLC: 9.8 min.

HPLC Trace for Formation of Modified H_2N-WGNFL-CO_2H 3b

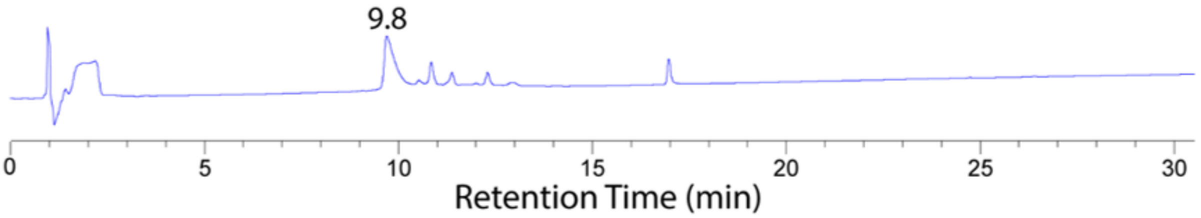

HRMS of Modified H_2N-WGNFL-CO_2H 3b

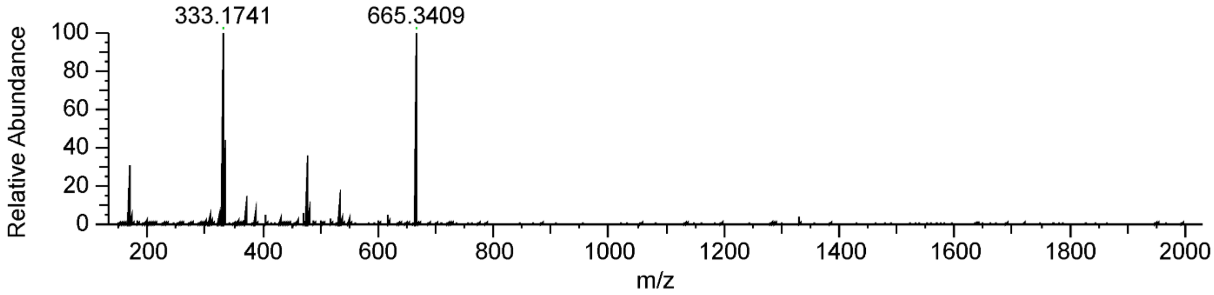

Reacting H_2N-WGDFL-(Nitrile) 2p with *N*-Methylhydroxylamine

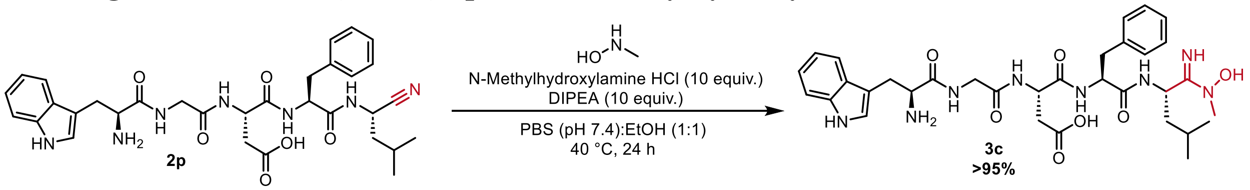

H_2N-WGDFL-(Nitrile) **2p** (1 mg, 1.6 µmol, 1 equiv.) was dissolved in 500 µL of 1:1 PBS (pH 7.4):EtOH in a 1/2” dram vial. Next, *N*-methylhydroxylamine HCl (1.35 mg, 16.2 µmol, 10 equiv.) was added in one portion along with DIPEA (2.8 µL, 16.2 µmol, 10 equiv.). The vial was stirred at 40 °C for 24 hours. The reaction was then analyzed via **HPLC Method A**, revealing >95% conversion to hydroxylamine-modified product **3c**.

Hydroxylamine-Modified H_2N-WGDFL-$CONH_2$ 3c. LCMS, m/z 665.3404 (calcd. $[M+H^+]$ = 665.3406), m/z 333.1737 (calcd. $[(M+2H^+)/2]$ = 333.1739) Purity: >99% (HPLC analysis at 220 nm). Retention time in HPLC: 9.5 min.

HPLC Trace for Formation of Modified H_2N-WGDFL-$CONH_2$ 3c

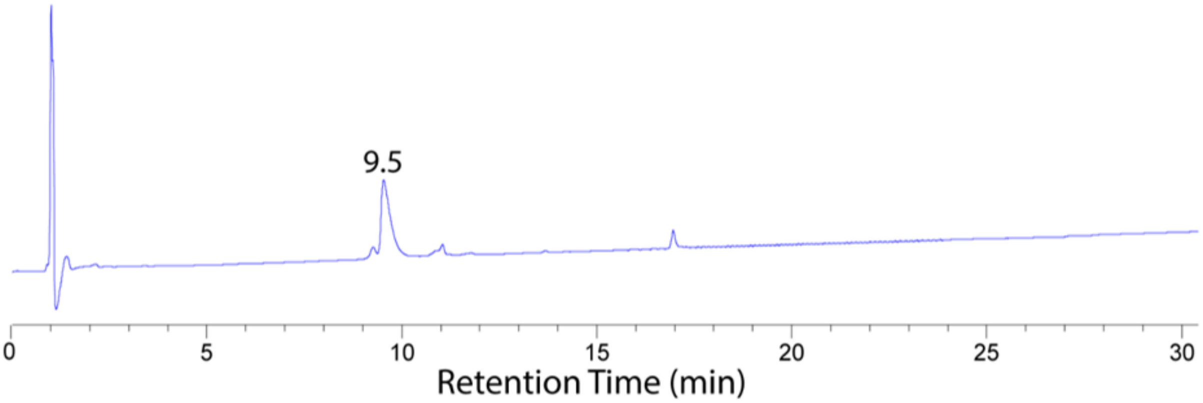

HRMS of Modified H_2N-WGDFL-$CONH_2$ 3c

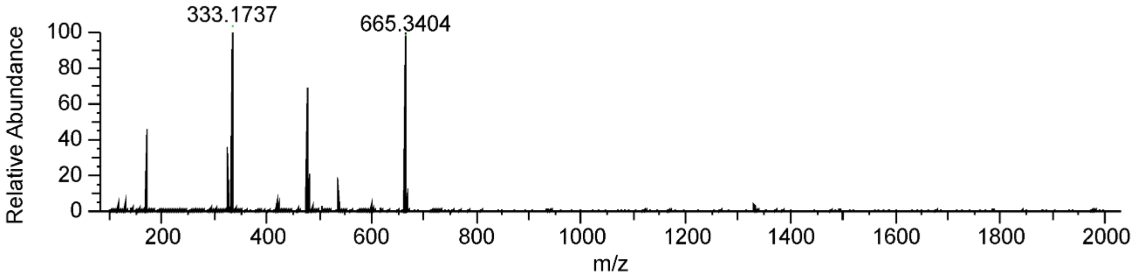

Peptide Rate Comparison for Reaction with *N*-Methylhydroxylamine

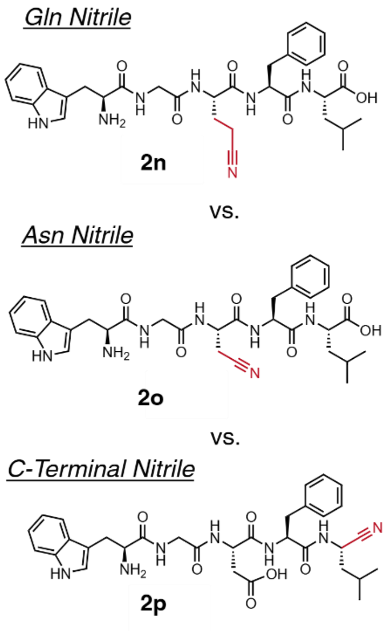

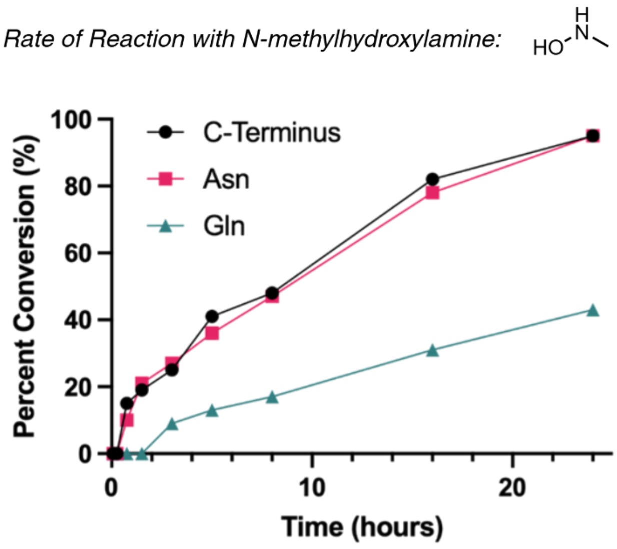

To evaluate the differential reactivity of different types of nitrile (Asn, Gln, C-terminus), three separate reactions were set between *N*-methylhydroxylamine and H_2N-WGQ(Nitrile)FL-CO_2H **2n**, H_2N-WGN(Nitrile)FL-CO_2H **2o**, or H_2N-WGDFL-(Nitrile) **2p**.

Procedure: Nitrile-containing peptide (1 mg, ~1.6 µmol, 1 equiv.) was dissolved in 500 µL of 1:1 PBS (pH 7.4):EtOH in a 1/2" dram vial. Next, *N*-methylhydroxylamine HCl (1.3 mg, ~16 µmol, 10 equiv.) was added in one portion along with DIPEA (2.8 µL, ~16 µmol, 10 equiv.). The vial was stirred at 40 °C and aliquots were taken at various timepoints and analyzed via **HPLC Method A** to monitor conversion to the hydroxylamine product (see table with conversions) and HPLC traces below.

	Type of Nitrile		
Time	Gln (**2n**)	Asn (**2o**)	C-terminus (**2p**)
5 min	<5%	<5%	<5%
15 min	<5%	<5%	<5%
45 min	<5%	10%	15%
1.5 h	<5%	21%	19%

3 h	9%	27%	25%
5 h	13%	36%	41%
8 h	17%	47%	48%
16 h	31%	78%	82%
24 h	43%	>95%	>95%

Stacked HPLC Traces for Hydroxylamine Modification of Gln Nitrile 2n to 3b

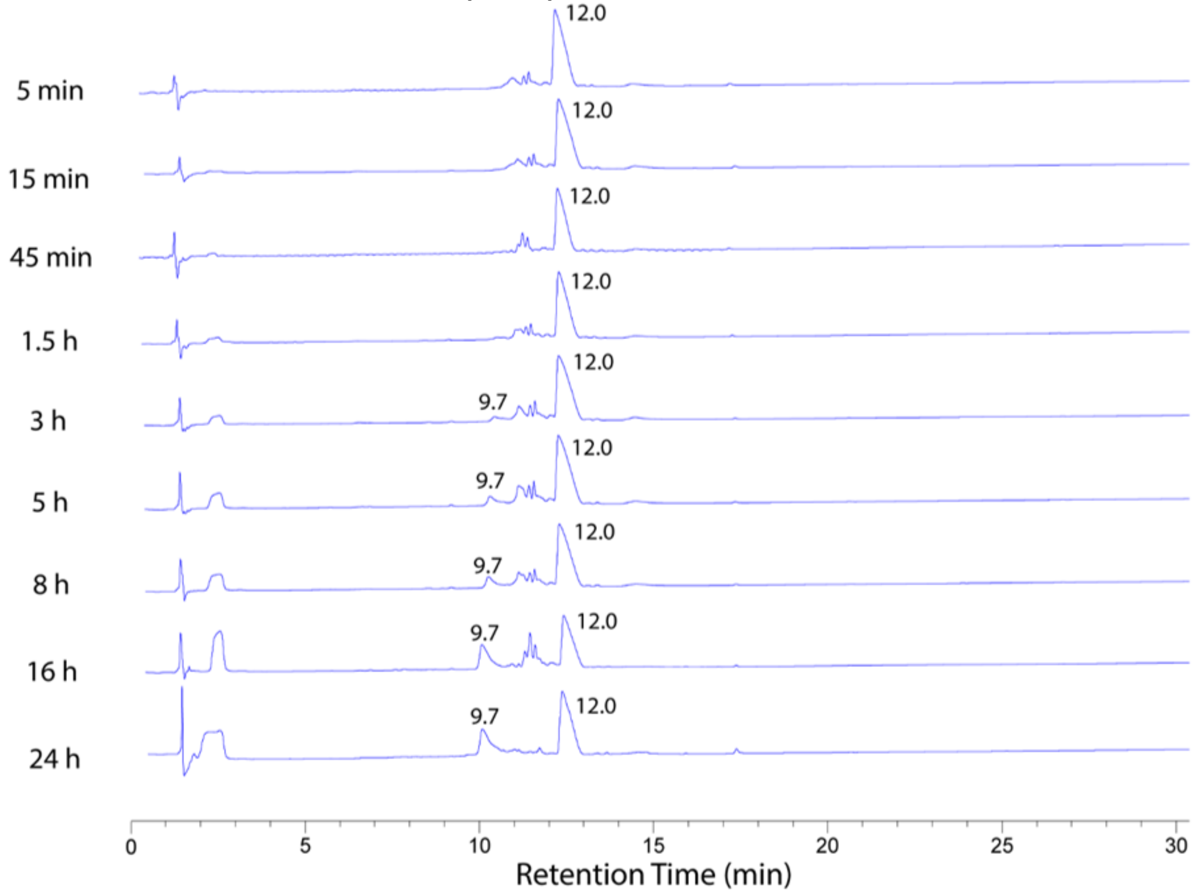

Stacked HPLC Traces for Hydroxylamine Modification of Asn Nitrile 2o to 3b

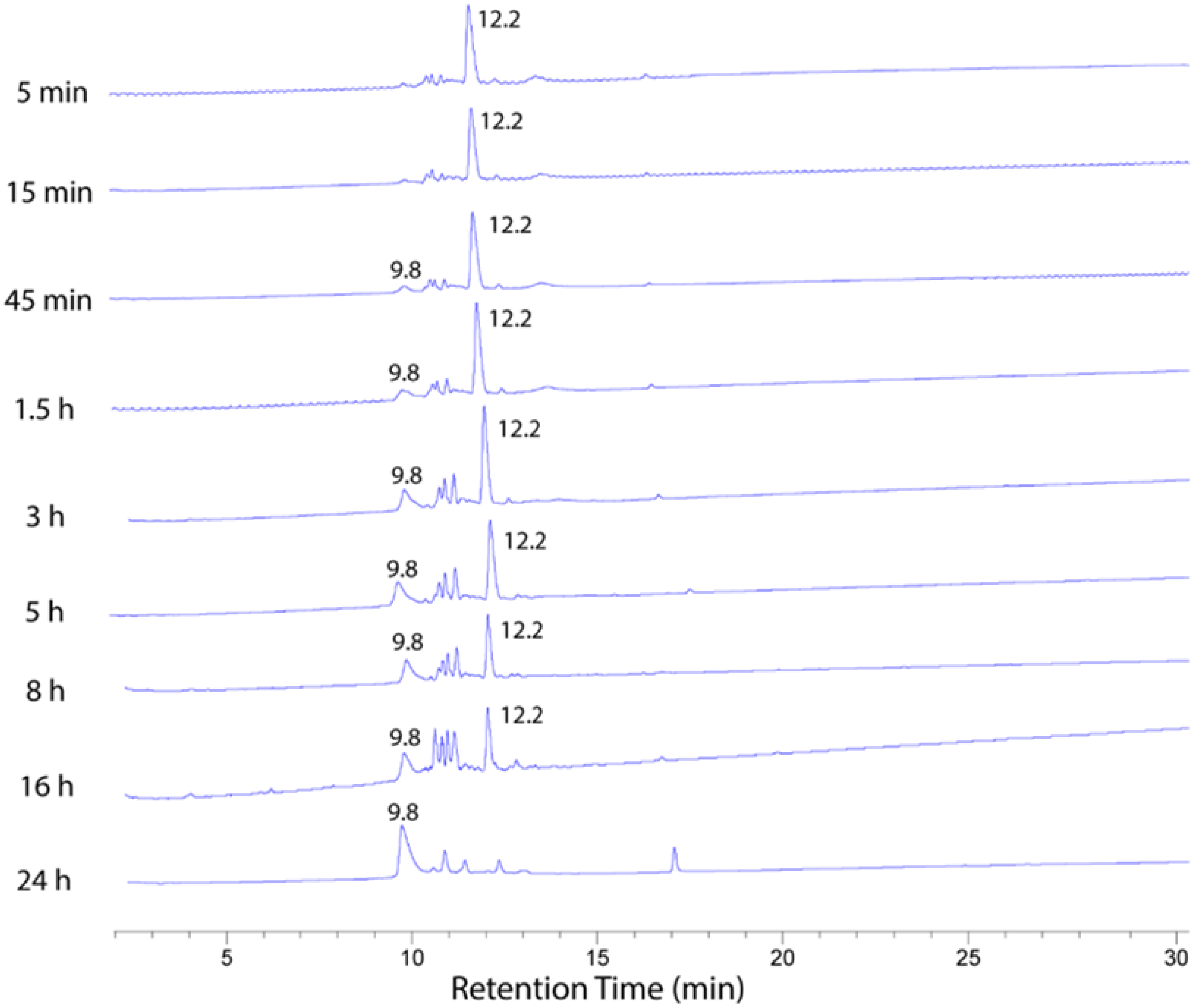

Stacked HPLC Traces for Hydroxylamine Modification of C-Terminal Nitrile 2p to 3c

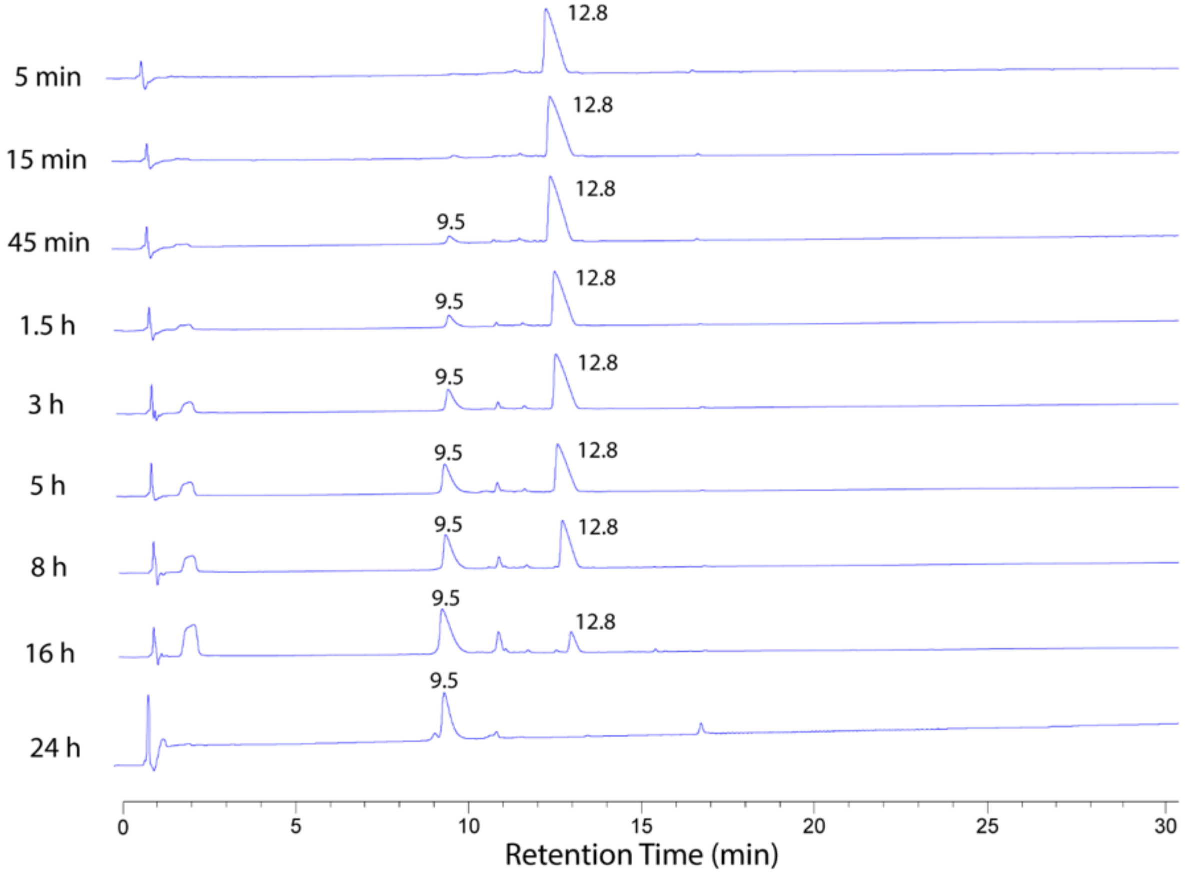

Reacting H_2N-WGQ(Nitrile)FL-CO_2H 2n with 4-Methoxyphenylboronic Acid

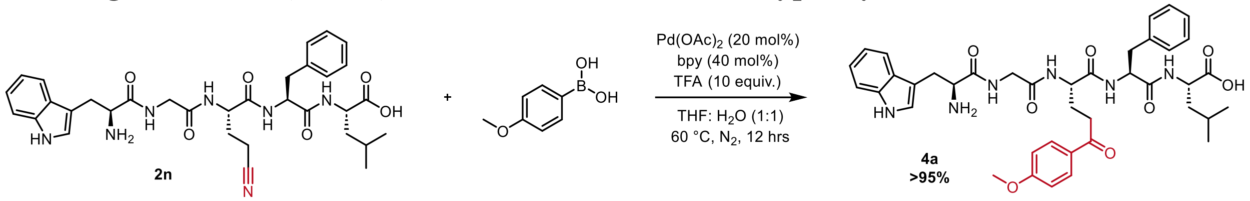

H_2N-WGQ(Nitrile)FL-CO_2H **2n** (1.0 mg, 1.6 µmol, 1 equiv.) and 4-methoxyphenylboronic acid (1.4 mg, 9.5 µmol, 6 equiv.) were added to a 1/2" dram vial. Next, 2,2'-bipyridyl ligand (0.10 mg, 0.63 µmol, 40 mol%) was added from a freshly prepared stock solution (30 µL, 1:1 H_2O:THF). TFA (~2 µL, 15.8 µmol, 10 equiv.) was then transferred to the solution, after which 270 µL H_2O:THF (1:1) was added to bring the total reaction volume to 300 µL. N_2 was bubbled for 2 minutes using an 18G x 1 ½" needle and balloon. Finally, $Pd(OAc)_2$ (0.07 mg, 0.32 µmol, 20 mol%) was added in 10 µL from a freshly prepared stock solution (1:1 H_2O:THF). N_2 was bubbled for another 60 seconds using an 18G x 1 ½" needle and balloon, and then the vial was flushed with N_2. The reaction was left stirring for 12 hours at 60 °C. The reaction was quenched with 3-MPA (3 µL, 20 equiv.) and analyzed via **HPLC Method A**, revealing >95% conversion to the ketone product H_2N-WGQ(paramethoxyphenylketone)FL-CO_2H **4a** based on remaining starting material.

H_2N-WGQ(paramethoxyphenylketone)FL-CO_2H (4a): LCMS, *m/z* 741.3608 (calcd. $[M+H^+]$ = 741.3606). Purity: >99% (HPLC analysis at 220 nm). Retention time using **HPLC Method A:** 15.5 min.

HPLC Trace for H_2N-WGQ(paramethoxyphenylketone)FL-CO_2H 4a

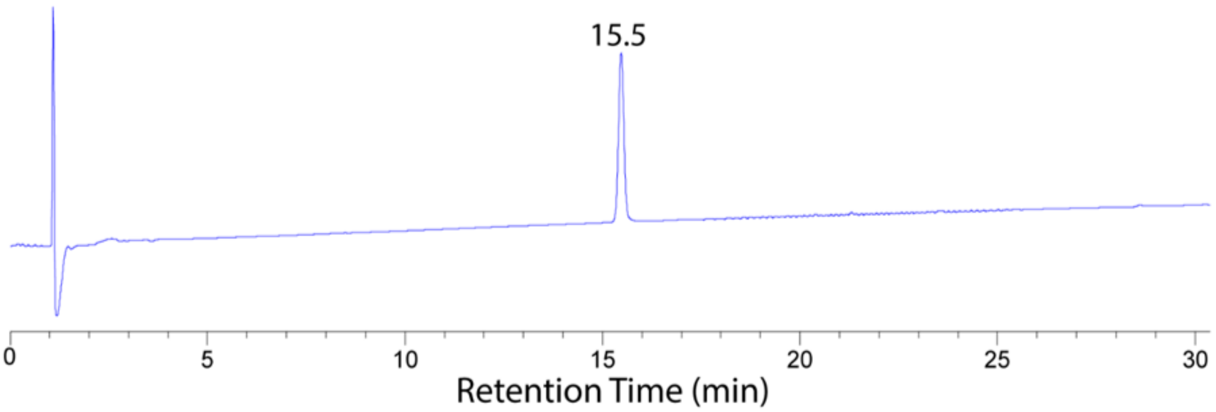

HRMS of H_2N-WGQ(paramethoxyphenylketone)FL-CO_2H 4a

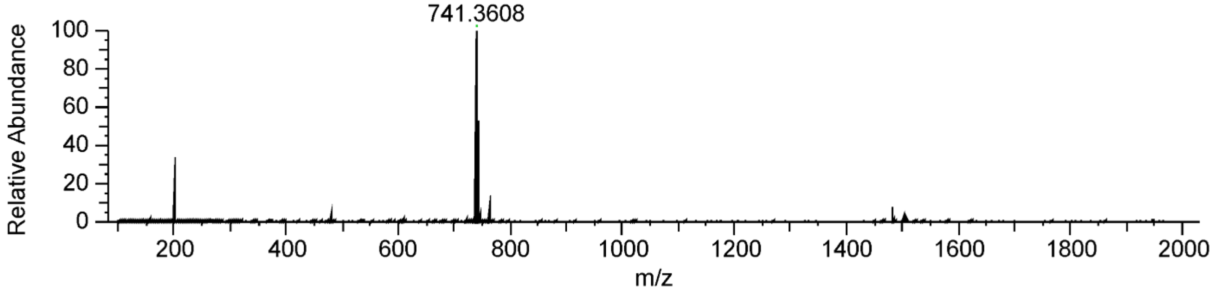

Reacting H_2N-WGN(Nitrile)FL-CO_2H 2o with 4-Methoxyphenylboronic Acid

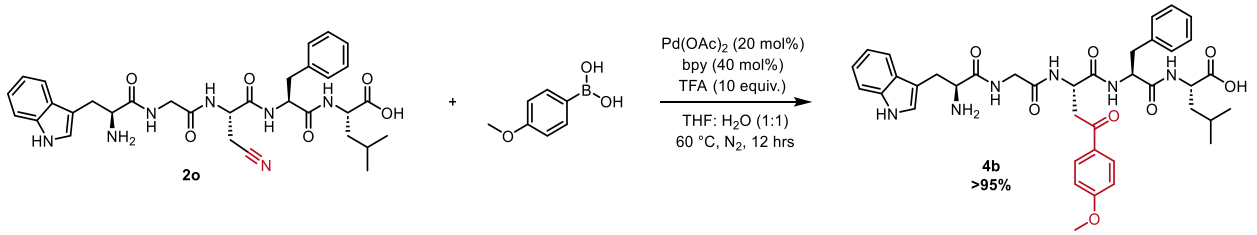

H_2N-WGN(Nitrile)FL-CO_2H **2o** (1.0 mg, 1.6 µmol, 1 equiv.) and 4-methoxyphenylboronic acid (1.5 mg, 9.7 µmol, 6 equiv.) were added to a 1/2" dram vial. Next, 2,2'-bipyridyl ligand (0.10 mg, 0.65 µmol, 40 mol%) was added from a freshly prepared stock solution (30 µL, 1:1 H_2O:THF). TFA (~2 µL, 16.2 µmol, 10 equiv.) was then transferred to the solution, after which 270 µL H_2O:THF (1:1) was added to bring the total reaction volume to 300 µL. N_2 was bubbled for 2 minutes using an 18G x 1 ½" needle and balloon. Finally, $Pd(OAc)_2$ (0.07 mg, 0.32 µmol, 20 mol%) was added in 10 µL from a freshly prepared stock solution (1:1 H_2O:THF). N_2 was bubbled for another 60 seconds using an 18G x 1 ½" needle and balloon, and then the vial was flushed with N_2. The reaction was left stirring for 12 hours at 60 °C. The reaction was quenched with 3-MPA (3 µL, 20 equiv.) and analyzed via **HPLC Method A**, revealing >95% conversion to the ketone product H_2N-WGN(paramethoxyphenylketone)FL-CO_2H **4b** based on remaining starting material.

H_2N-WGN(paramethoxyphenylketone)FL-CO_2H (4b): LCMS, *m/z* 727.3450 (calcd. $[M+H^+]$ = 727.3450). Purity: >99% (HPLC analysis at 220 nm). Retention time using **HPLC Method A:** 15.3 min.

HPLC Trace for H_2N-WGN(paramethoxyphenylketone)FL-CO_2H 4b

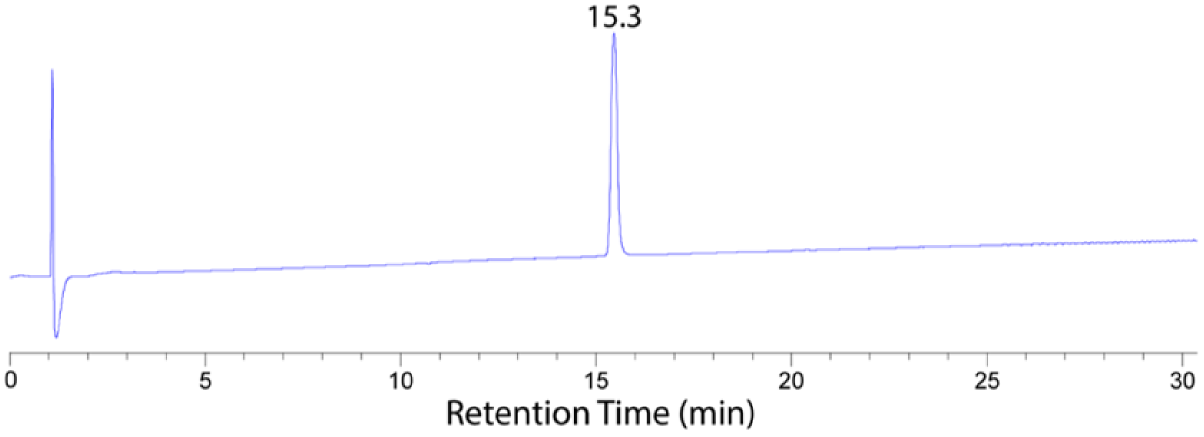

HRMS of H_2N-WGN(paramethoxyphenylketone)FL-CO_2H 4b

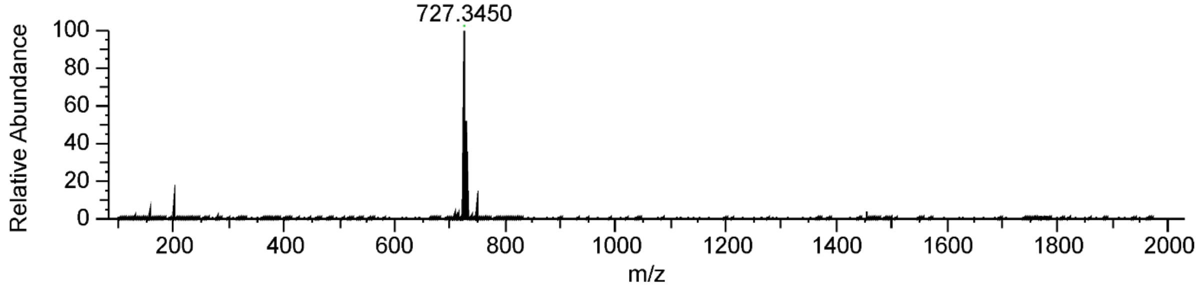

Reacting H_2N-WGDFL-(Nitrile) 2p with 4-Methoxyphenylboronic Acid

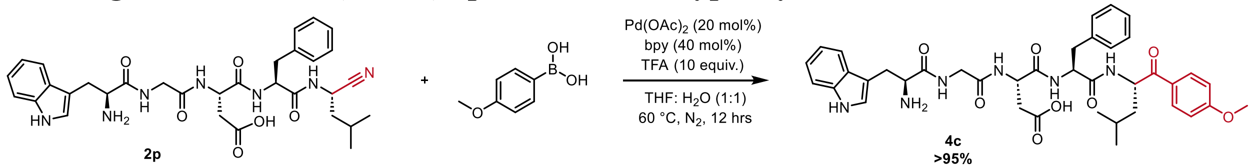

H_2N-WGDFL-(Nitrile) **2p** (1.0 mg, 1.6 µmol, 1 equiv.) and 4-methoxyphenylboronic acid (1.5 mg, 9.7 µmol, 6 equiv.) were added to a 1/2" dram vial. Next, 2,2'-bipyridyl ligand (0.10 mg, 0.65 µmol, 40 mol%) was added from a freshly prepared stock solution (30 µL, 1:1 H_2O:THF). TFA (~2 µL, 16.2 µmol, 10 equiv.) was then transferred to the solution, after which 270 µL H_2O:THF (1:1) was added to bring the total reaction volume to 300 µL. N_2 was bubbled for 2 minutes using an 18G x 1 ½" needle and balloon. Finally, $Pd(OAc)_2$ (0.07 mg, 0.32 µmol, 20 mol%) was added in 10 µL from a freshly prepared stock solution (1:1 H_2O:THF). N_2 was bubbled for another 60 seconds using an 18G x 1 ½" needle and balloon, and then the vial was flushed with N_2. The reaction was left stirring for 12 hours at 60 °C. The reaction was quenched with 3-MPA (3 µL, 20 equiv.) and analyzed via **HPLC Method A**, revealing >95% conversion to the ketone product H_2N-WGDFL-(paramethoxyphenylketone) **4c** based on remaining starting material.

H_2N-WGDFL-(paramethoxyphenylketone) (4c): LCMS, *m/z* 727.3448 (calcd. $[M+H^+]$ = 727.3450). Purity: >99% (HPLC analysis at 220 nm). Retention time using **HPLC Method A:** 15.4 min.

HPLC Trace for H_2N-WGDFL-(paramethoxyphenylketone) 4c

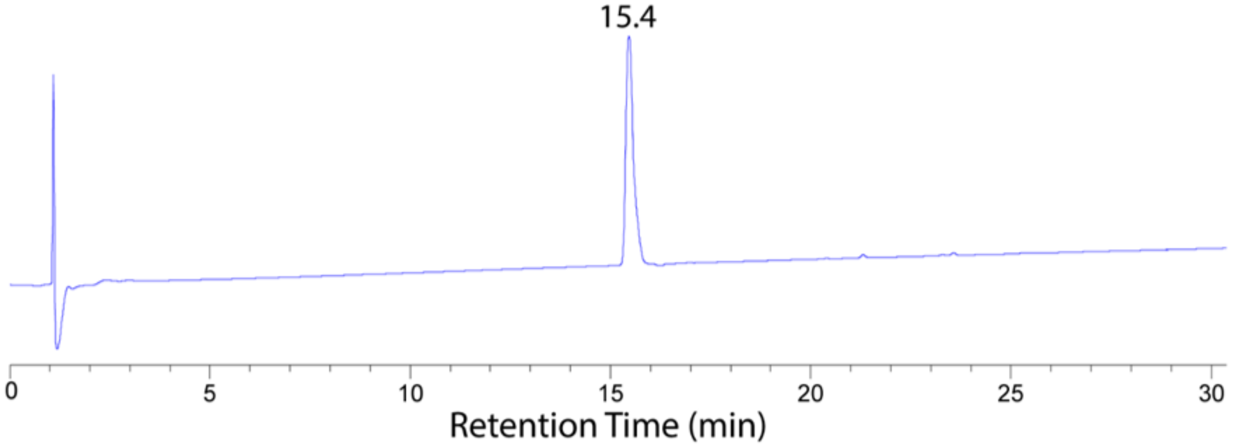

HRMS of H_2N-WGDFL-(paramethoxyphenylketone 4c

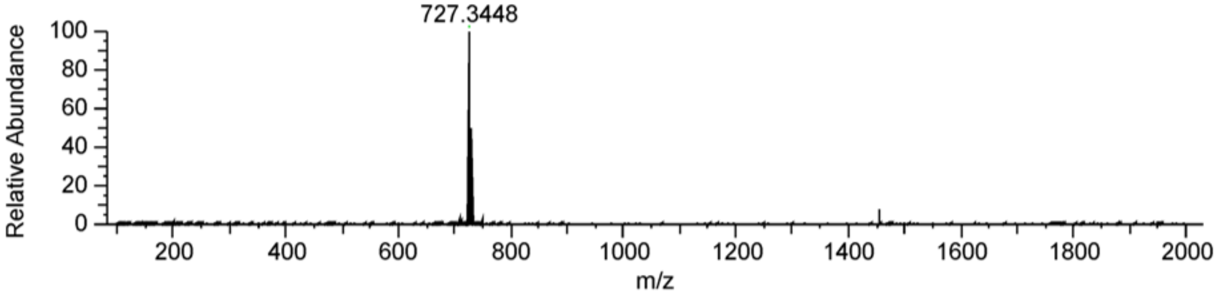

Supplementary Fig. 5b: Further Exploration of Hydroxylamine Modification of Small Molecule Nitrile

Modification of Small Molecule Nitrile with Hydroxylamine at 40 °C

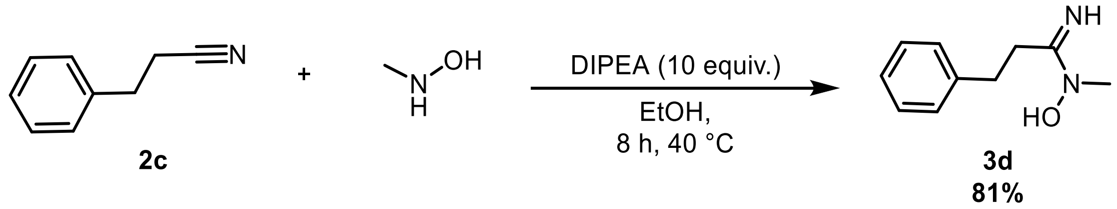

3-phenylpropionitrile **2c** (50 µL, 0.38 mmol, 1 equiv.) was dissolved in 5 mL of EtOH in a 25 mL RBF. Next, *N*-methylhydroxylamine HCl (159 mg, 1.91 mmol, 5 equiv.) was added in one portion along with DIPEA (332 µL, 1.91 µmol, 5 equiv.). The vial was stirred at 40 °C. Conversion was monitored by HPLC (see conversion table below), and after 8 h, all starting material was

consumed. The reaction mixture was purified directly using preparative HPLC, and fractions containing the product were collected and lyophilized to yield the product **3d** as a pale yellow liquid (55 mg, 81% yield). **^{1}H NMR** (400 MHz, DMSO): δ = 8.32 (s, 1H), 7.34 – 7.20 (m, 5H), 3.28 (s, 3H), 2.86 (dd, *J* = 9.6, 6.2 Hz, 2H), 2.72 (dd, *J* = 9.7, 6.2 Hz, 2H) ppm. **^{13}C NMR** (101 MHz, DMSO): δ = 165.52, 139.46, 128.45, 126.49, 40.61, 31.98, 30.43 ppm. **HRMS**: calcd. for $C_{10}H_{15}N_2O^+$ [M+H$^+$] 179.1179; found 179.1178.

**Conversion of Nitrile 2c to Product 3d at 40 °C**	
Time	Conversion
5 h	93%
8 h	>95%

Small Molecule Hydroxylamine Product (3d): Purity: >99% (HPLC analysis at 220 nm). Retention time using **HPLC Method A:** 5.2-5.3 min.

2-phenylpropionitrile (2c): Purity: >99% (HPLC analysis at 220 nm). Retention time using **HPLC Method A:** 14.6 min.

HPLC Trace for Conversion to 3d (5 h)

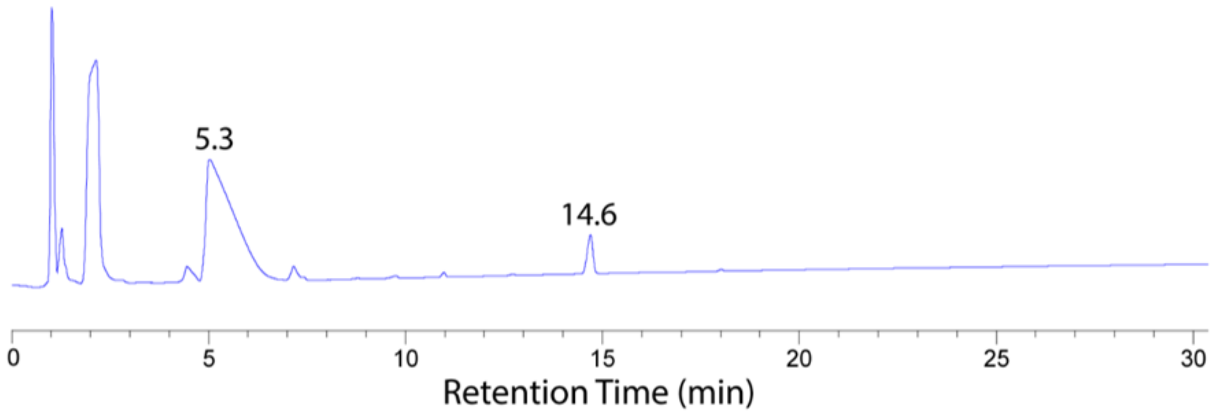

HPLC Trace for Conversion to 3d (8 h)

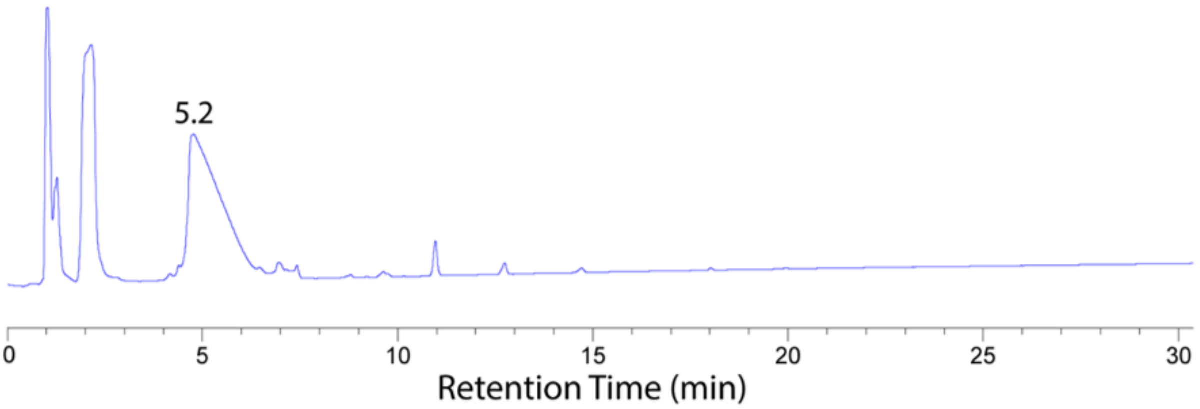

HRMS of Small Molecule Hydroxylamine Product 3d (Peak 5.2-5.3 min)

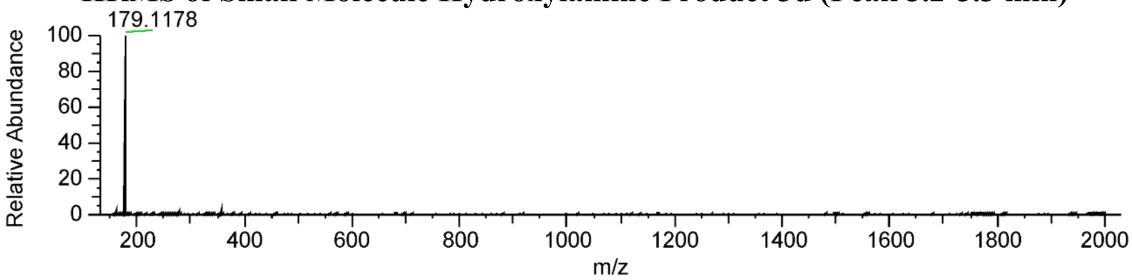

^{1}H NMR of Small Molecule Hydroxylamine Product 3d

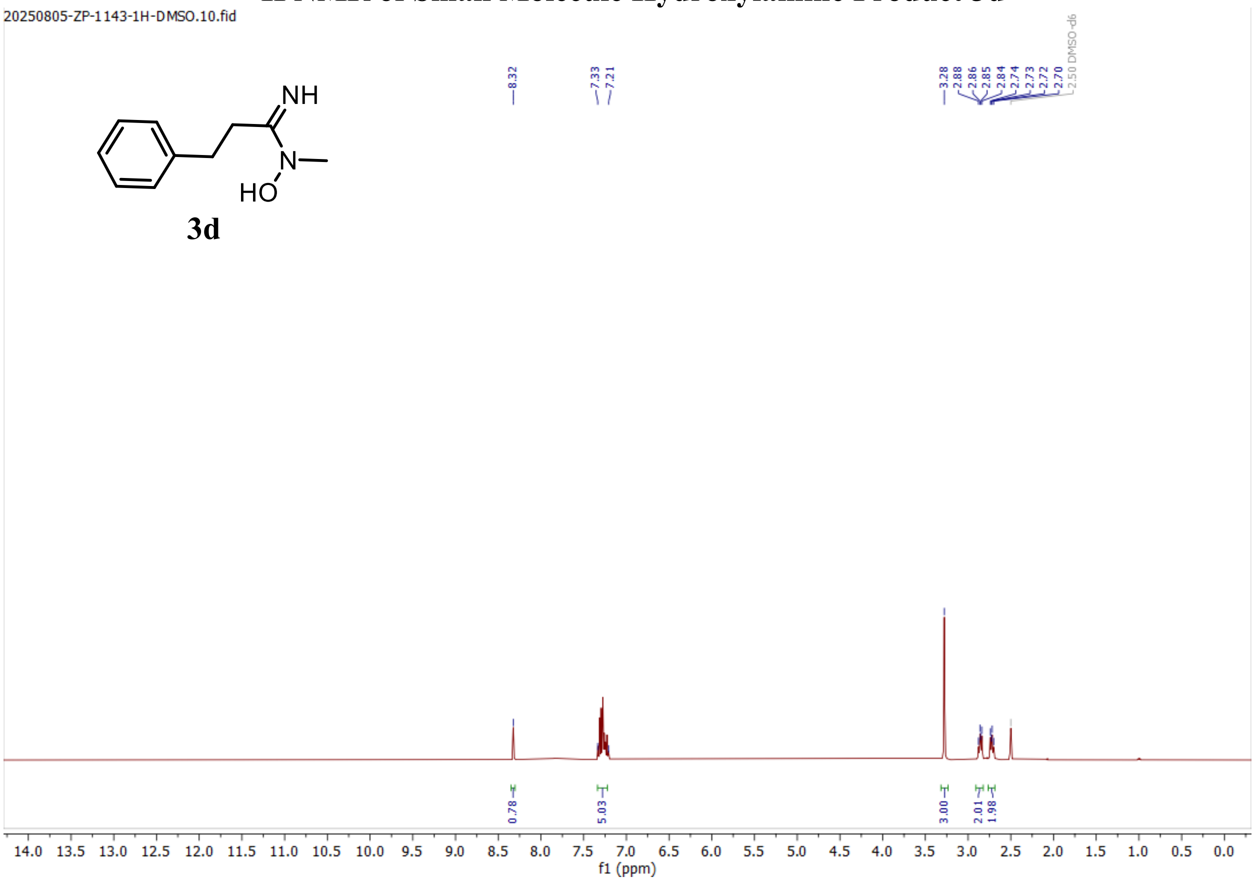

^{13}C NMR of Small Molecule Hydroxylamine Product 3d

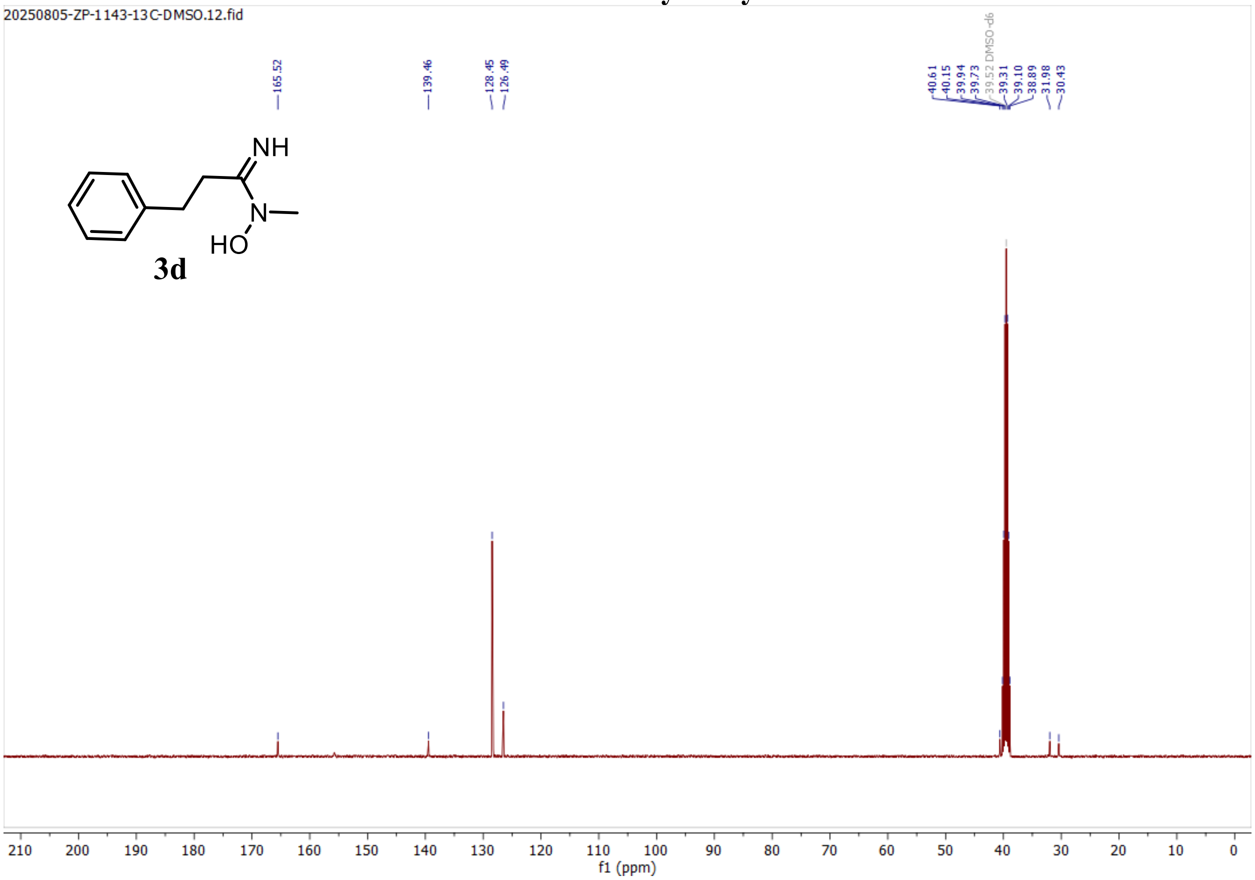

Modification of Small Molecule Nitrile with Hydroxylamine at Room Temperature

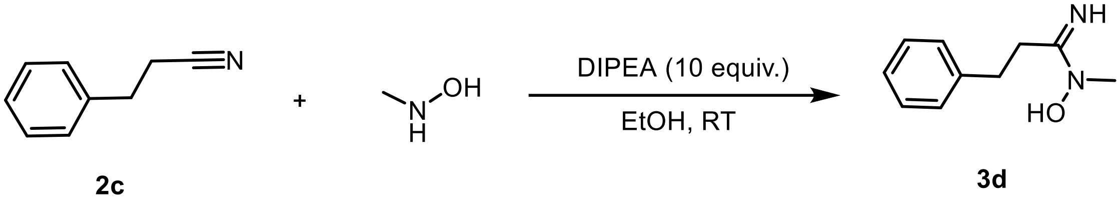

3-phenylpropionitrile **2c** (50 µL, 0.38 mmol, 1 equiv.) was dissolved in 5 mL of EtOH in a 25 mL RBF. Next, *N*-methylhydroxylamine HCl (159 mg, 1.91 mmol, 5 equiv.) was added in one portion along with DIPEA (332 µL, 1.91 µmol, 5 equiv.). The vial was stirred at room temperature. Conversion was monitored by HPLC (see conversion table below), and while conversion was noticeably slower than at 40 °C, after 24 h, conversion reached 94%.

Conversion of Nitrile 2c to Product 3d at RT	
Time	Conversion
2 h	67%
3 h	72%
5 h	80%

24 h	94%

Small Molecule Hydroxylamine Product (3d): Purity: >99% (HPLC analysis at 220 nm). Retention time using **HPLC Method A:** 5.0-5.3 min.

2-phenylpropionitrile (2c): Purity: >99% (HPLC analysis at 220 nm). Retention time using **HPLC Method A:** 14.5-14.7 min.

HPLC Trace for Conversion to 3d (2 h)

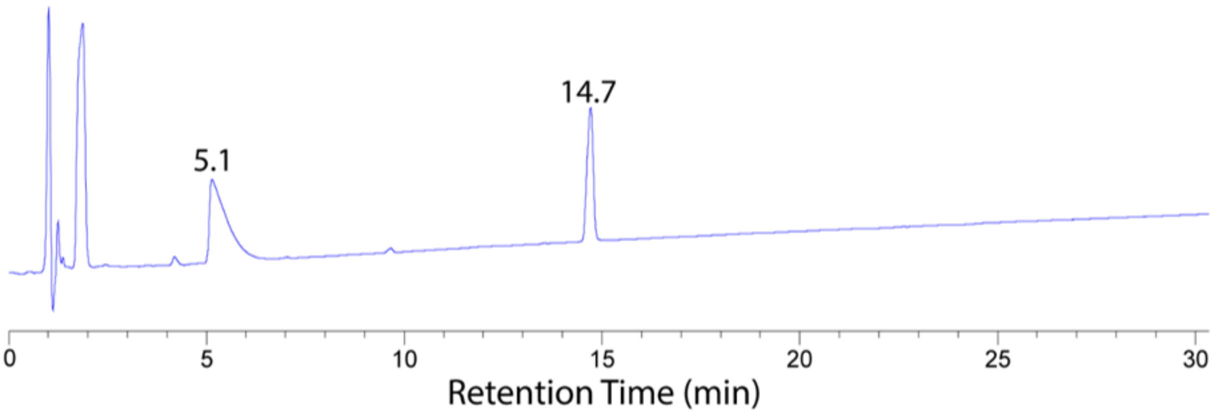

HPLC Trace for Conversion to 3d (3 h)

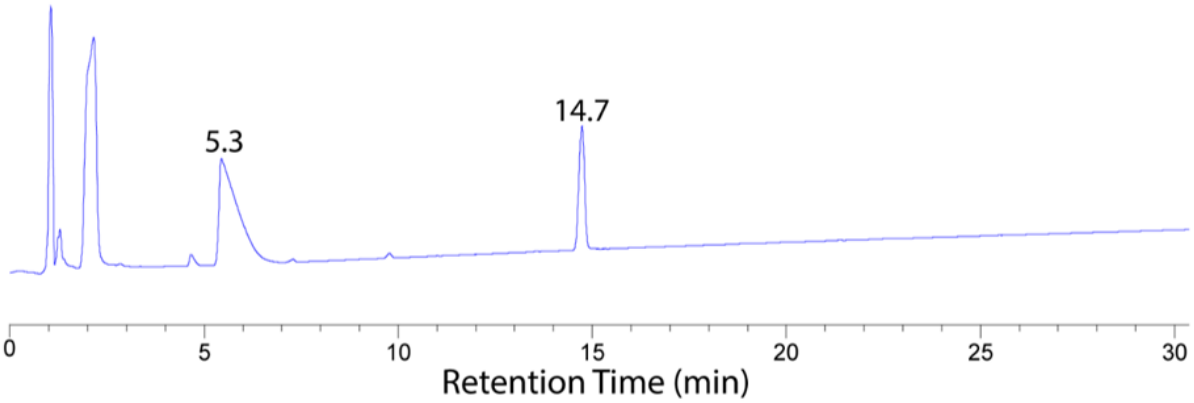

HPLC Trace for Conversion to 3d (5 h)

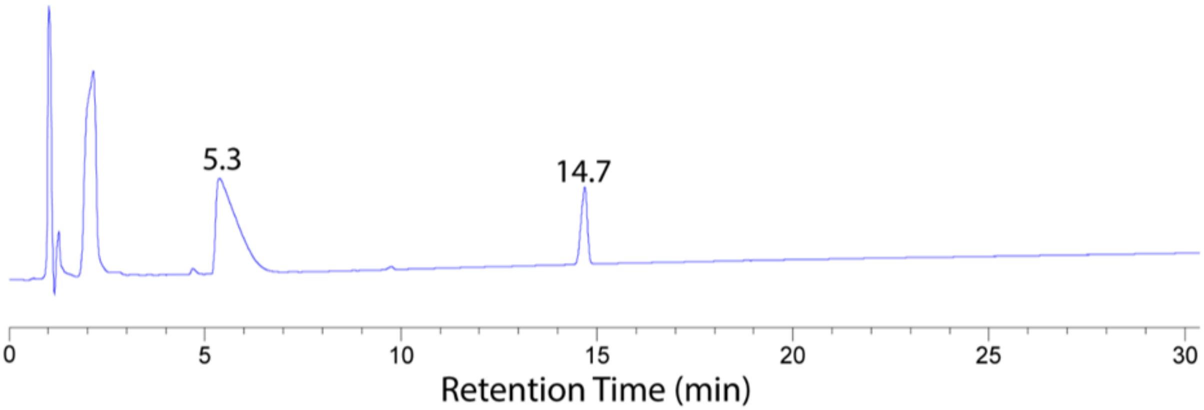

HPLC Trace for Conversion to 3d (24 h)

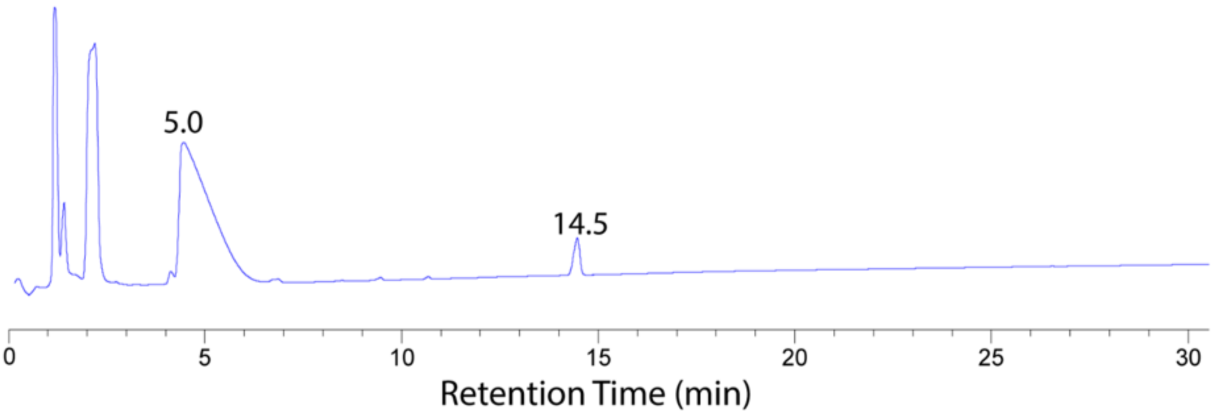

Attempted Addition of Catalysts and Ligands to Accelerate Hydroxylamine Nitrile Modification

In an attempt to accelerate the rate of nitrile modification by hydroxylamine at room temperature and establish conditions for more efficient peptide nitrile modification, we experimented with the addition of different Cu(I) and Cu(II) salts, along with *N,N'*-Dimethyl-1,2-cyclohexanediamine ligand.

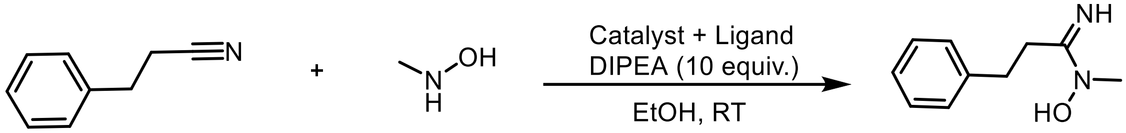

Procedure: 3-phenylpropionitrile **2c** (5 µL, 38 µmol, 1 equiv.) was dissolved in 500 µL of EtOH in a 35 mL high-pressure tube. Next, *N*-methylhydroxylamine HCl (15.9 mg, 191 µmol, 5 equiv.) was added in one portion along with DIPEA (33.2 µL, 191 µmol, 5 equiv.), *N,N'*-dimethyl-1,2-cyclohexanediamine ligand (3.0 µL, 19 µmol, 50 mol%), and Cu catalyst (7.6 µmol, 20 mol%). The vial was stirred at room temperature. After 3 h, conversion was evaluated, and for all screened conditions, moderate conversion (52-62%) was observed – ligand and catalyst addition did not accelerate reaction rate as intended.

Entry	Catalyst (20 mol%)	Ligand (50 mol%)	Conversion (3 h)
1	$Cu(OTf)_2$	None	62%
2	$Cu(OTf)_2$	*N,N'*-dimethyl-1,2-cyclohexanediamine	53%
3	CuBr	None	55%
4	CuBr	*N,N'*-dimethyl-1,2-cyclohexanediamine	58%
5	$CuSO_4$	None	62%
6	$CuSO_4$	*N,N'*-dimethyl-1,2-cyclohexanediamine	52%

Small Molecule Hydroxylamine Product (3d): Purity: >99% (HPLC analysis at 220 nm). Retention time using **HPLC Method A:** 5.7-5.8 min.

2-phenylpropionitrile (2c): Purity: >99% (HPLC analysis at 220 nm). Retention time using **HPLC Method A:** 14.7 min.

HPLC Trace for Conversion to 3d (Entry 1)

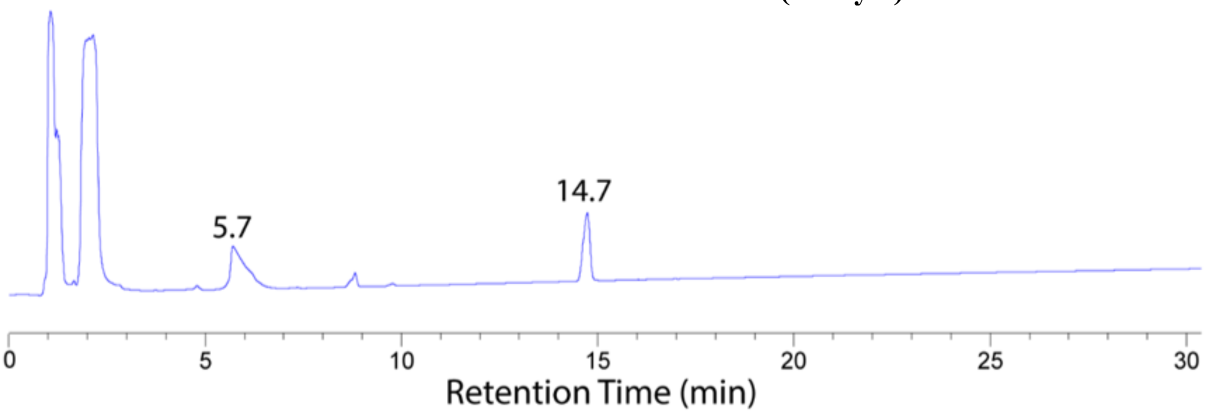

HPLC Trace for Conversion to 3d (Entry 2)

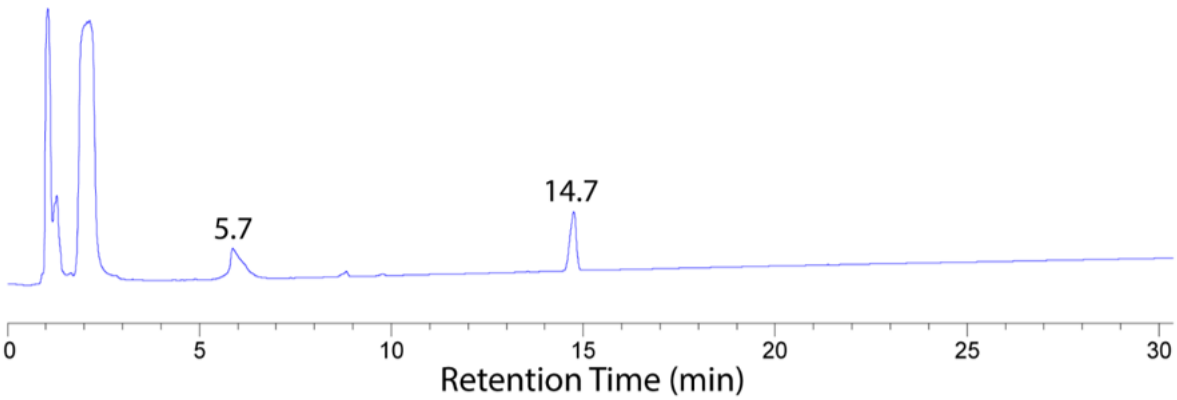

HPLC Trace for Conversion to 3d (Entry 3)

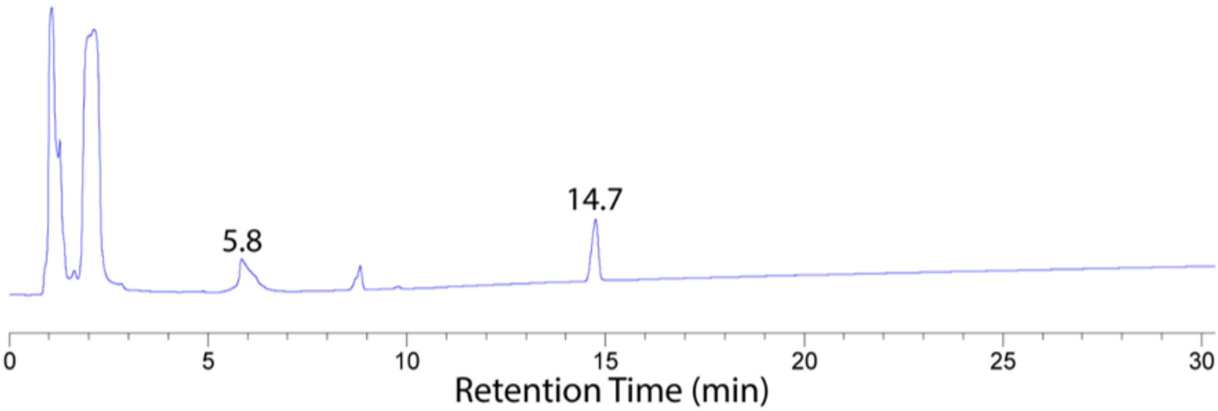

HPLC Trace for Conversion to 3d (Entry 4)

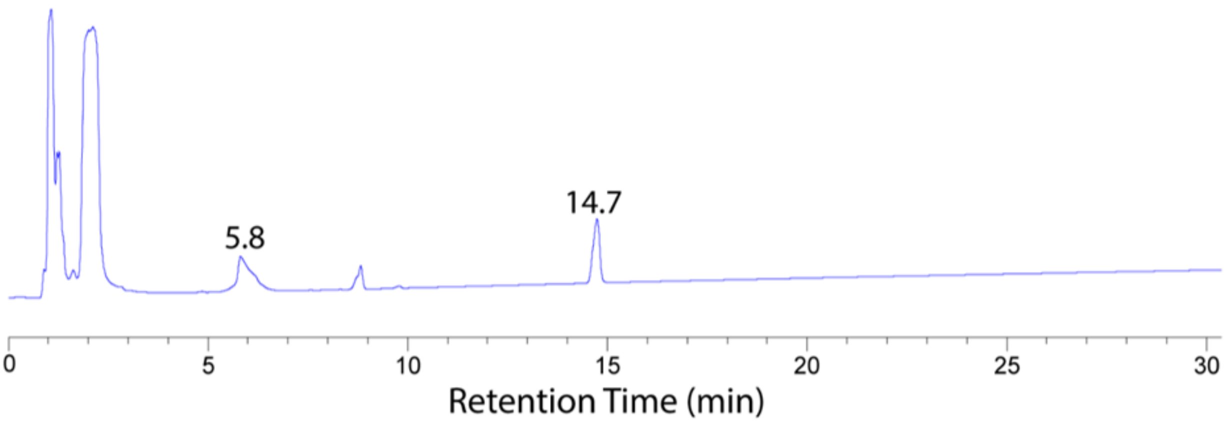

HPLC Trace for Conversion to 3d (Entry 5)

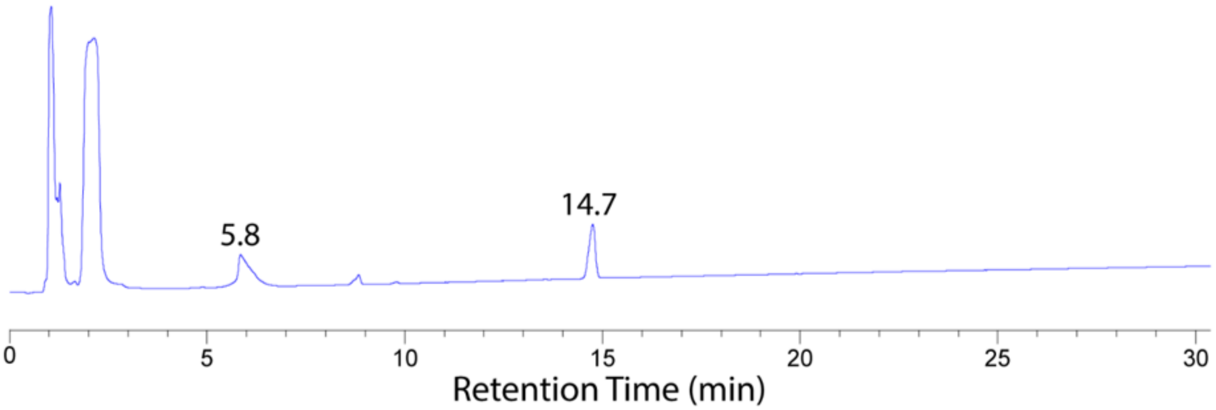

HPLC Trace for Conversion to 3d (Entry 6)

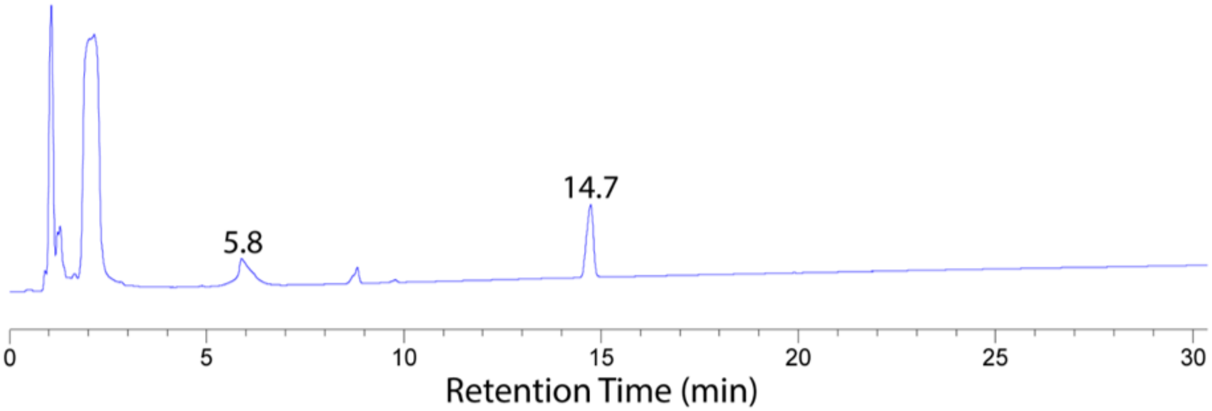

Screening O-Substituted Hydroxylamines for Small Molecule Nitrile Modification

To examine the substrate scope of hydroxylamine nitrile modification, O-substituted hydroxylamines were evaluated. However, these hydroxylamines failed to furnish the desired product even after long periods of heating. N-substituted hydroxylamines with free -OH appear to be required for efficient reaction with nitrile.

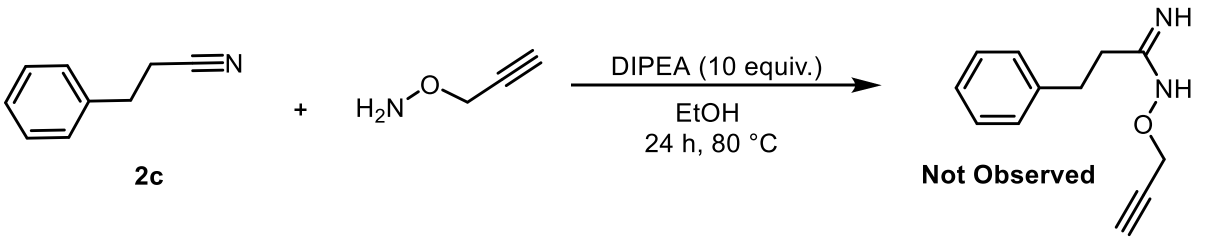

3-phenylpropionitrile **2c** (50 µL, 0.38 mmol, 1 equiv.) was dissolved in 5 mL of EtOH in a 35 mL high-pressure tube. Next, *O*-2-Propynylhydroxylamine HCl (205 mg, 1.91 mmol, 5 equiv.) was added in one portion along with DIPEA (332 µL, 1.91 µmol, 5 equiv.). The vial was stirred at 80 °C. After 24 h, no conversion to desired product was observed based on HPLC, with starting material fully intact.

2-phenylpropionitrile (2c): Purity: >99% (HPLC analysis at 220 nm). Retention time using **HPLC Method A:** 14.7 min.

HPLC Trace for Reaction of Nitrile 2c with O-2-Propynylhydroxylamine

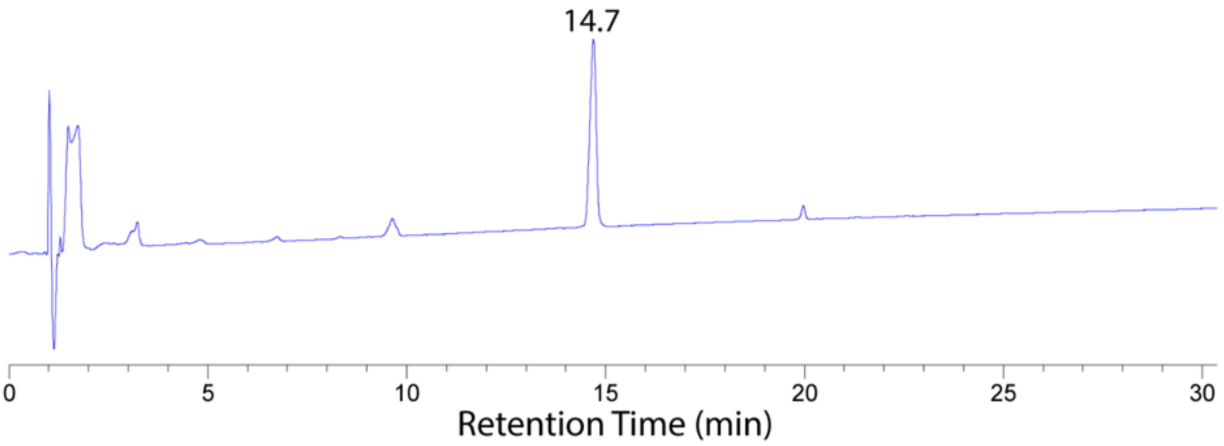

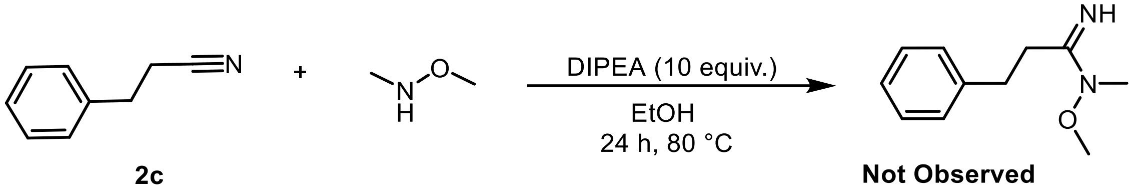

3-phenylpropionitrile **2c** (50 µL, 0.38 mmol, 1 equiv.) was dissolved in 5 mL of EtOH in a 35 mL high-pressure tube. Next, *N,O*-Dimethylhydroxylamine HCl (186 mg, 1.91 mmol, 5 equiv.) was added in one portion along with DIPEA (332 µL, 1.91 µmol, 5 equiv.). The vial was stirred at 80 °C. After 24 h, no conversion to desired product was observed based on HPLC, with starting material fully intact.

2-phenylpropionitrile (2c): Purity: >99% (HPLC analysis at 220 nm). Retention time using **HPLC Method A:** 14.7 min.

HPLC Trace for Reaction of Nitrile 2c with *N,O*-Dimethylhydroxylamine

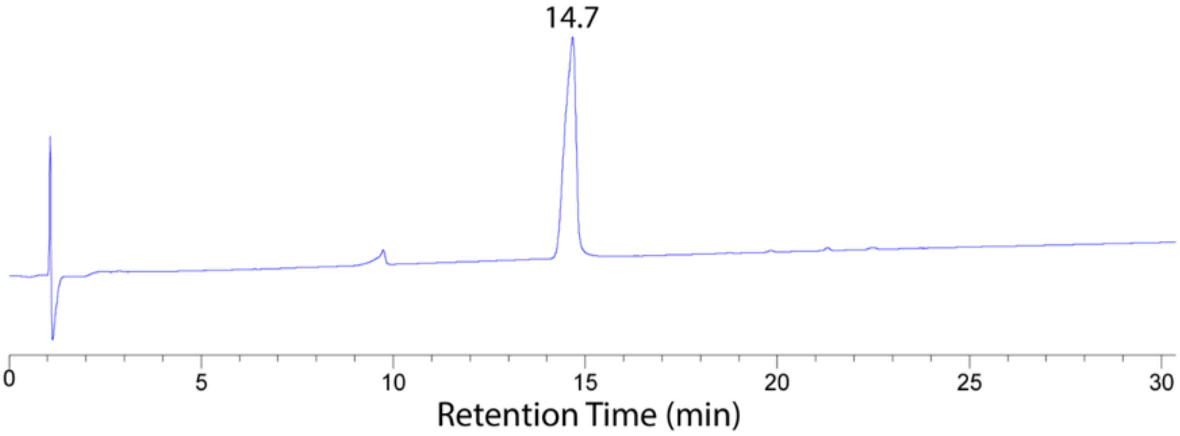

Supplementary Fig. 5c: Further Exploration of Boronic Acid Carbometallation on Small Molecule Nitrile

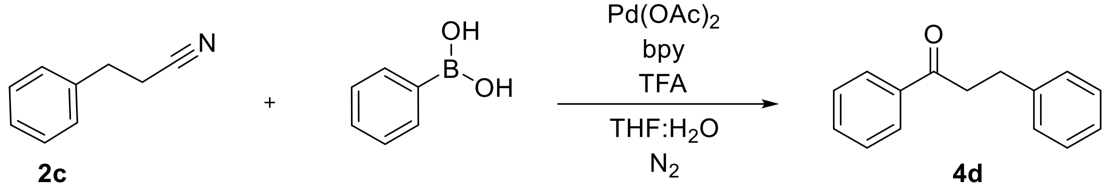

Procedure adopted from Wu and co-workers.[2] To a 25 mL RBF was added 2.4 mL of THF:H_2O and TFA (306 µL, 4.0 mmol, 10 equiv.). Next, 3-phenylpropionitrile **2c** (53 µL, 0.4 mmol, 1 equiv.), phenylboronic acid, and 2,2'-bipyridyl ligand were added to the solution. The mixture was left to stir until all compounds were dissolved, after which N_2 was bubbled for 5 minutes. Finally, $Pd(OAc)_2$ was transferred to the solution. The reaction was left under an N_2 atmosphere (balloon) at a designated temperature for the specified time. Upon completion by TLC, the reaction mixture was diluted with EtOAc (10 mL) and transferred to a 60 mL separatory funnel. The organic layer was washed with brine (3 x 15 mL), dried over Na_2SO_4, and then adsorbed onto silica. Purification by silica gel column chromatography (1:20 EtOAc:Hex eluent) yielded 1,3-diphenylpropan-1-one **4d** as a pale light brown solid (72 mg, 85% yield). Analytical TLC, 1:4 EtOAc:Hex eluent, R_f = 0.65.

^{1}H NMR (400 MHz, $CDCl_3$): δ = 7.98 (dd, *J* = 8.4, 1.4 Hz, 2H), 7.62 – 7.52 (m, 1H), 7.50 – 7.44 (m, 2H), 7.36 – 7.27 (m, 4H), 7.26 – 7.21 (m, 1H), 3.35 – 3.29 (m, 2H), 3.13 – 3.07 (m, 2H) ppm. **^{13}C NMR** (101 MHz, $CDCl_3$): δ = 199.28, 141.38, 136.94, 133.14, 128.68, 128.61, 128.51, 128.12, 126.22, 40.51, 30.21 ppm. **HRMS**: calcd. for $C_{15}H_{15}O^+$ [M+H$^+$] 211.1117; found 211.1117.

Optimization Table:

Entry	Temperature	THF:H_2O Ratio	$Pd(OAc)_2$	bpy	TFA	Phenylboronic Acid	Time	Yield
1	RT	5:1	10 mol%	20 mol%	10 equiv.	4 equiv.	36 hrs	42%
**2**	**40 °C**	**5:1**	**10 mol%**	**20 mol%**	**10 equiv.**	**4 equiv.**	**8 hrs**	**85%**

3	40 °C	5:1	20 mol%	40 mol%	10 equiv.	4 equiv.	6 hrs	83%
4	40 °C	1:1	10 mol%	20 mol%	10 equiv.	4 equiv.	8 hrs	79%
5	60 °C	5:1	10 mol%	20 mol%	10 equiv.	4 equiv.	5 hrs	83%
6	80 °C	5:1	10 mol%	20 mol%	10 equiv.	2 equiv.	5 hrs	74%
7	80 °C	5:1	10 mol%	20 mol%	10 equiv.	4 equiv.	3 hrs	76%
8	80 °C	5:1	10 mol%	20 mol%	None	4 equiv.	48 hrs	n.d.

^{1}H NMR of 4d

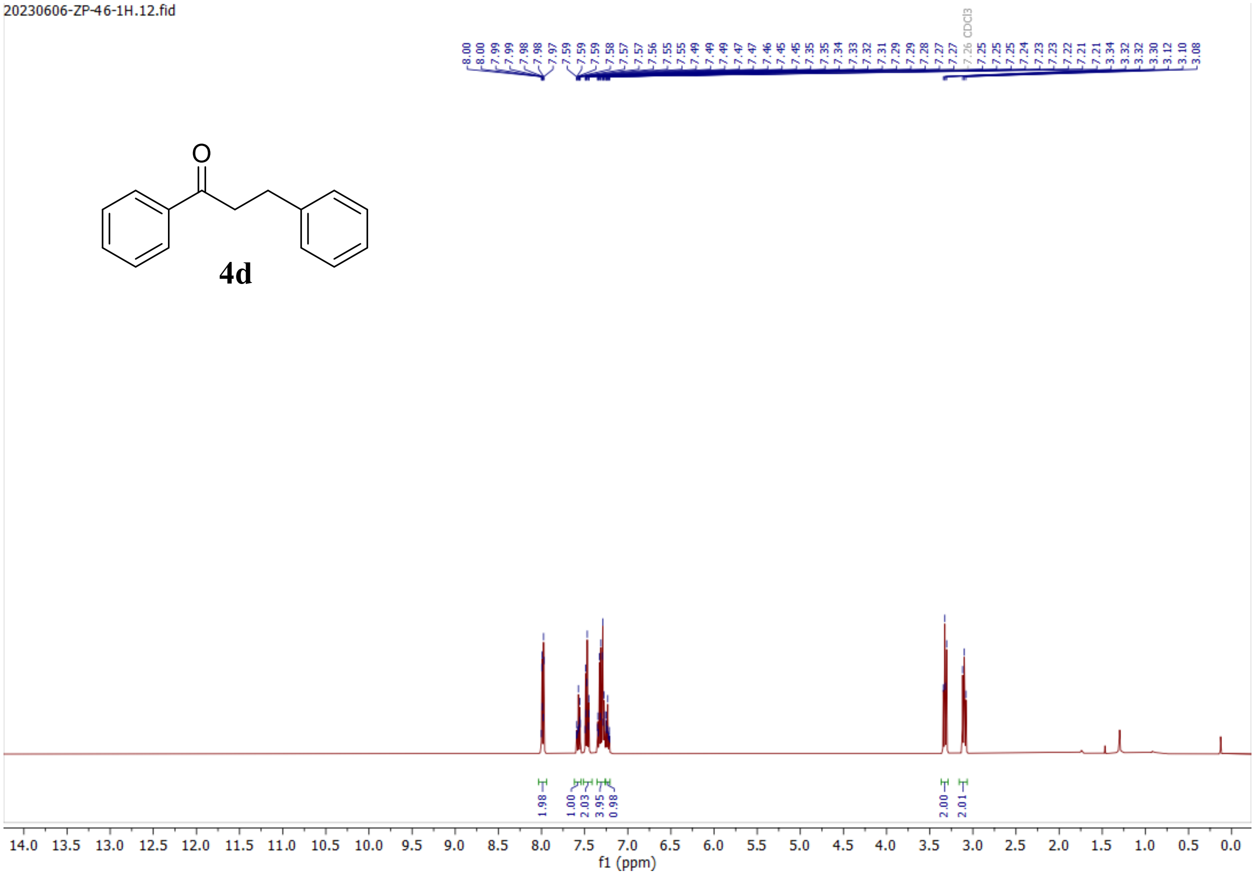

^{13}C NMR of 4d

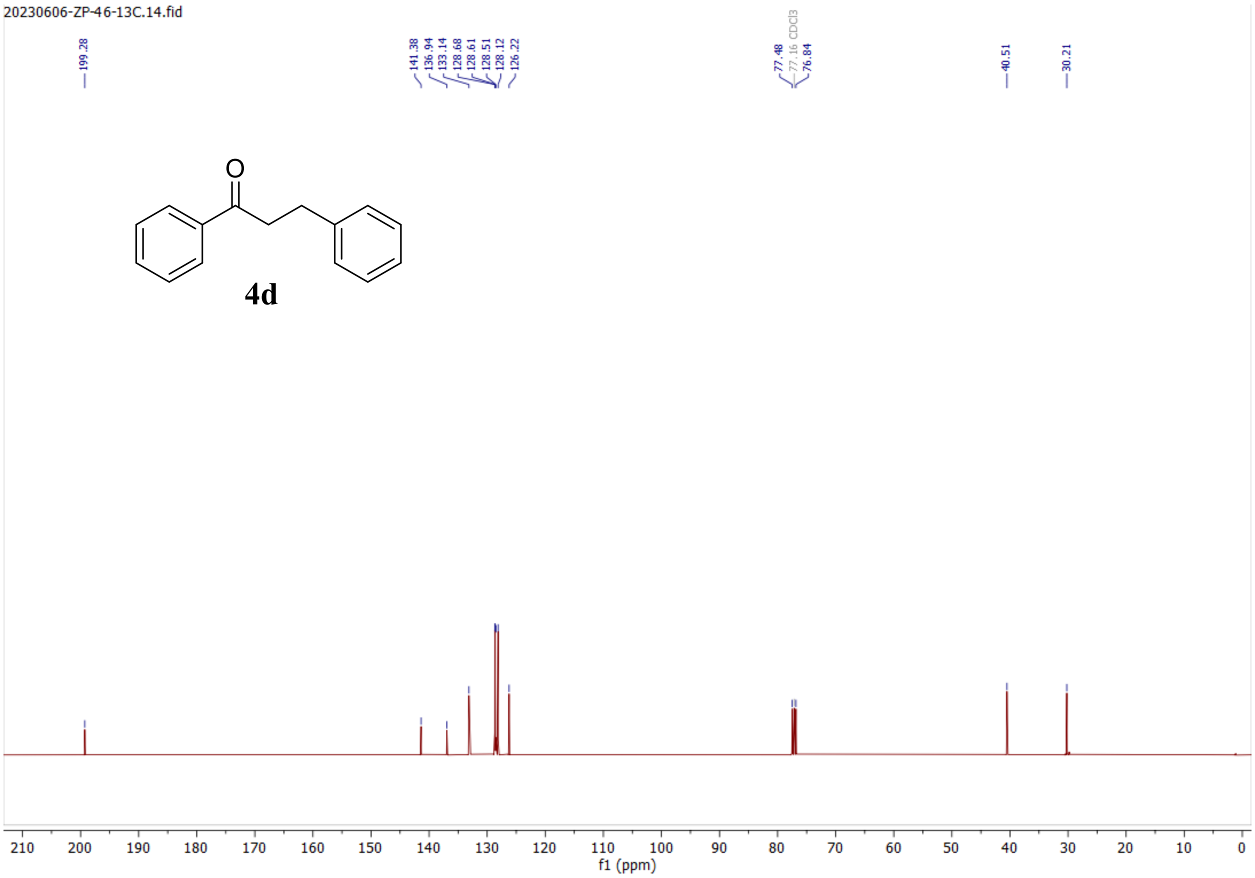

Supplementary Fig. 6: Small Molecule Boronic Acid Carbometallation Substrate Scope

General Procedure for Nitrile Boronic Acid Carbometallation Reaction on Small Molecule (GP-I):

To a 25 mL RBF was added 2.4 mL of THF:H_2O (5:1) and TFA (306 µL, 4.0 mmol, 10 equiv.). Next, 3-phenylpropionitrile **2c** (53 µL, 0.4 mmol, 1 equiv.), the appropriate boronic acid (1.6 mmol, 4 equiv.), and 2,2'-bipyridyl ligand (12.5 mg, 0.08 mmol, 20 mol%) were added to the solution. The mixture was left to stir until all compounds were dissolved, after which N_2 was bubbled for 5 minutes. Finally, $Pd(OAc)_2$ (9.0 mg, 0.04 mmol, 10 mol%) was transferred to the solution. The reaction was left for 8 hours under an N_2 atmosphere (balloon) at 40 °C. Upon completion by TLC, the reaction mixture was diluted with EtOAc (10 mL) and transferred to a 60 mL separatory funnel. The organic layer was washed with brine (3 x 15 mL), dried over Na_2SO_4, and then adsorbed onto silica. Purification by silica gel column chromatography yielded the small molecule ketone products.

General Procedure for Carbometallation Reaction Using Weakly Electron-Withdrawing Boronic Acids (GP-II):

To a 35 mL high-pressure tube was added 2.4 mL of THF:H_2O (5:1) and TFA (306 μL, 4.0 mmol, 10 equiv.). Next, 3-phenylpropionitrile **2c** (53 μL, 0.4 mmol, 1 equiv.), the appropriate boronic acid (1.6 mmol, 4 equiv.), and 2,2'-bipyridyl ligand (12.5 mg, 0.08 mmol, 20 mol%) were added to the solution. The mixture was left to stir until all compounds were dissolved, after which N_2 was bubbled for 5 minutes. Finally, $Pd(OAc)_2$ (9.0 mg, 0.04 mmol, 10 mol%) was transferred to the solution. The high-pressure tube was flushed with N_2 and the reaction was stirred for 24 hours at 80 °C. Upon completion by TLC, the reaction mixture was diluted with EtOAc (10 mL) and transferred to a 60 mL separatory funnel. The organic layer was washed with brine (3 x 15 mL), dried over Na_2SO_4, and then adsorbed onto silica. Purification by silica gel column chromatography yielded the small molecule ketone products.

General Procedure for Carbometallation Reaction Using Strongly Electron-Withdrawing Boronic Acids (GP-III):

To a 35 mL high-pressure tube was added 2.4 mL of THF:H_2O (5:1) and TFA (306 μL, 4.0 mmol, 10 equiv.). Next, 3-phenylpropionitrile **2c** (53 μL, 0.4 mmol, 1 equiv.), the appropriate boronic acid (1.6 mmol, 4 equiv.), and 2,2'-bipyridyl ligand (25.0 mg, 0.16 mmol, 40 mol%) were added to the solution. The mixture was left to stir until all compounds were dissolved, after which N_2 was bubbled for 5 minutes. Finally, $Pd(OAc)_2$ (18.0 mg, 0.08 mmol, 20 mol%) was transferred to the solution. The high-pressure tube was flushed with N_2 and the reaction was stirred for 48 hours at 80 °C. Upon completion by TLC, the reaction mixture was diluted with EtOAc (10 mL) and transferred to a 60 mL separatory funnel. The organic layer was washed with brine (3 x 15 mL), dried over Na_2SO_4, and then adsorbed onto silica. Purification by silica gel column chromatography yielded the small molecule ketone products.

Synthesis of 1-(4-methoxyphenyl)-3-phenylpropan-1-one (4e)

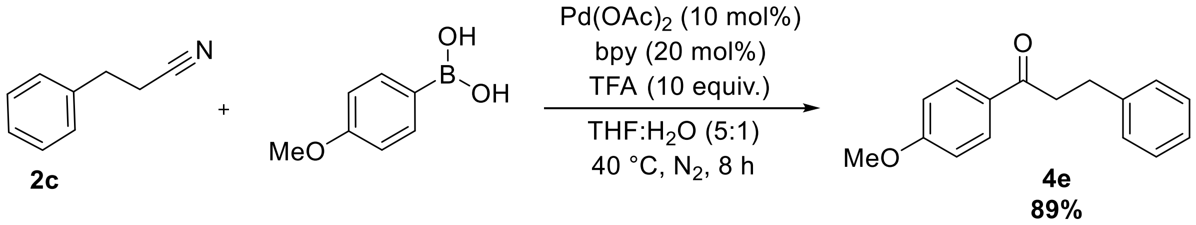

1-(4-methoxyphenyl)-3-phenylpropan-1-one **4e** was synthesized according to **GP-I**. Purification by silica gel column chromatography (1:20 EtOAc:Hex eluent) yielded the product **4e** as a pale white-yellow solid (85.5 mg, 89% yield). Analytical TLC, 1:4 EtOAc:Hex eluent, R_f = 0.60.

^{1}H NMR (400 MHz, $CDCl_3$): δ = 7.98 – 7.91 (m, 2H), 7.33 – 7.23 (m, 4H), 7.23 – 7.17 (m, 1H), 6.95 – 6.89 (m, 2H), 3.86 (s, 2H), 3.28 – 3.22 (m, 2H), 3.09 – 3.03 (m, 2H) ppm. **^{13}C NMR** (101 MHz, $CDCl_3$): δ = 197.97, 163.59, 141.61, 130.45, 130.12, 128.64, 128.56, 126.22, 113.87, 55.60, 40.25, 30.48 ppm. **HRMS**: calcd. for $C_{16}H_{17}O_2^+$ [M+H$^+$] 241.1223; found 241.1213.

^{1}H NMR of 4e

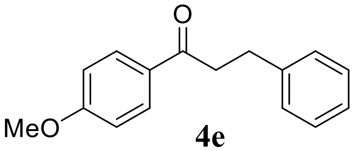

4e

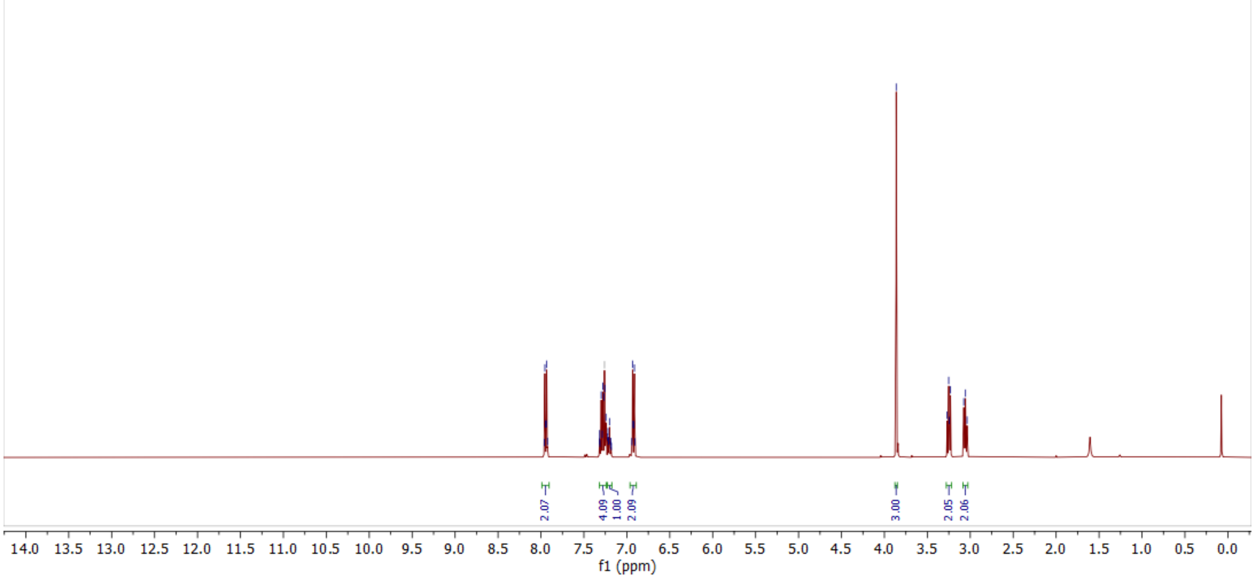

^{13}C NMR of 4e

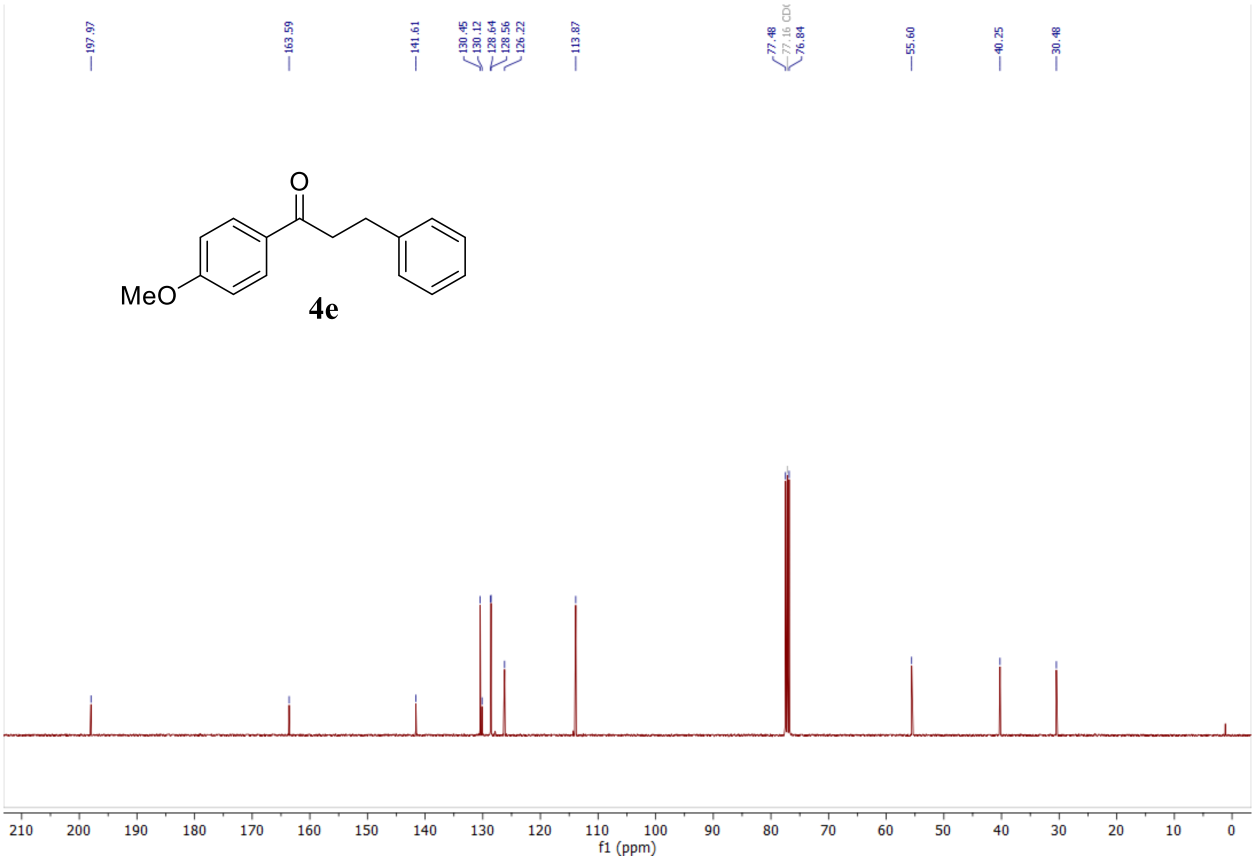

Synthesis of 1-(4-hydroxyphenyl)-3-phenylpropan-1-one (4f)

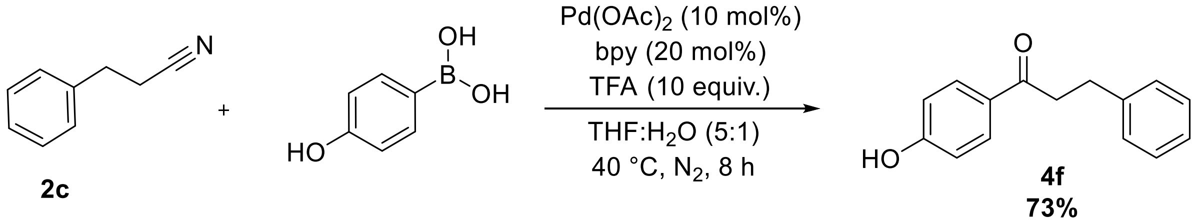

1-(4-hydroxyphenyl)-3-phenylpropan-1-one **4f** was synthesized according to **GP-I**. Purification by silica gel column chromatography (1:18 EtOAc:Hex eluent) yielded the product **4f** as a clear, crystalline solid (66.1 mg, 73% yield). Analytical TLC, 1:4 EtOAc:Hex eluent, $R_f = 0.50$.

^{1}H NMR (400 MHz, $CDCl_3$): δ = 7.93 – 7.88 (m, 2H), 7.33 – 7.27 (m, 2H), 7.26 – 7.18 (m, 3H), 6.94 – 6.89 (m, 2H), 3.30 – 3.25 (m, 2H), 3.06 (t, J = 7.8 Hz, 2H) ppm. **^{13}C NMR** (101 MHz, $CDCl_3$): δ = 199.60, 161.18, 141.23, 131.00, 129.51, 128.67, 128.50, 126.30, 115.69, 40.25, 30.64 ppm. **HRMS**: calcd. for $C_{15}H_{15}O_2^+$ [M+H$^+$] 227.1067; found 227.1057.

^{1}H NMR of 4f

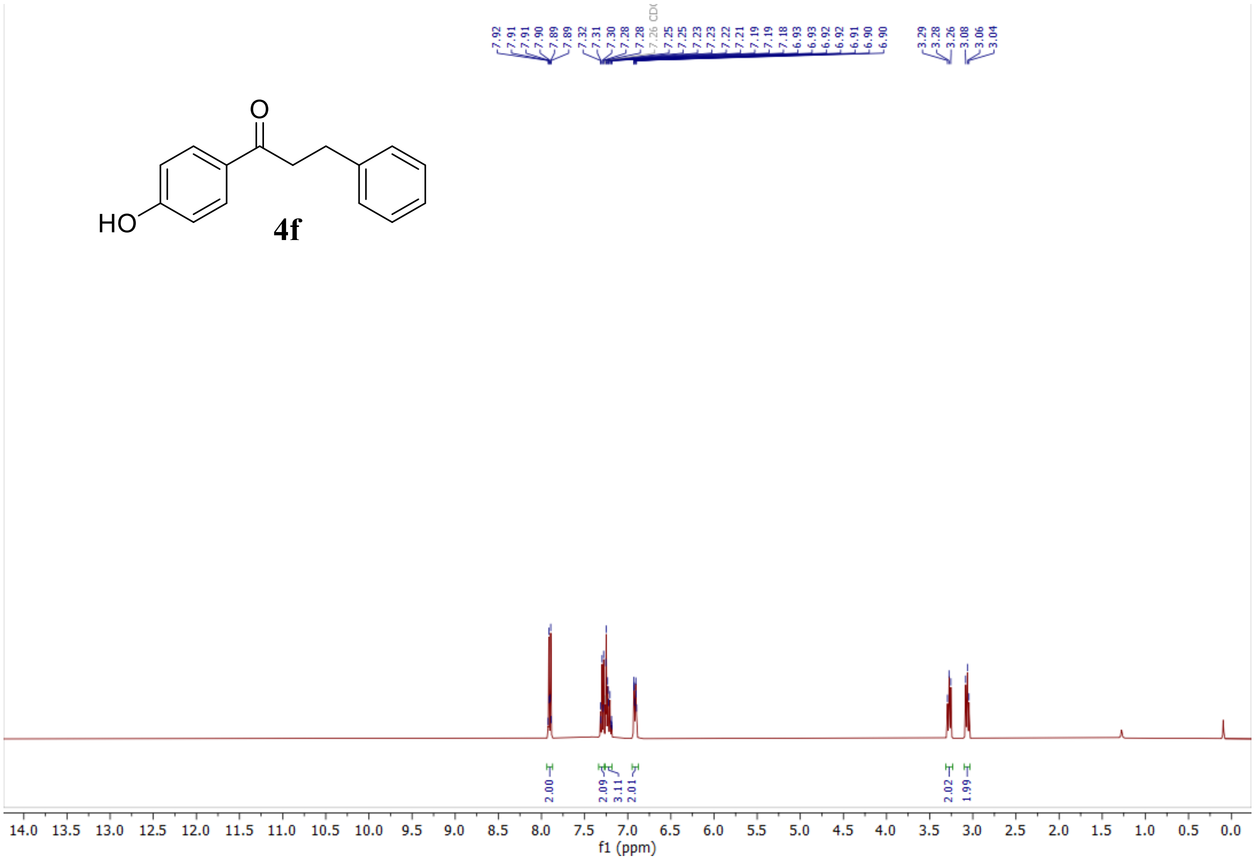

^{13}C NMR of 4f

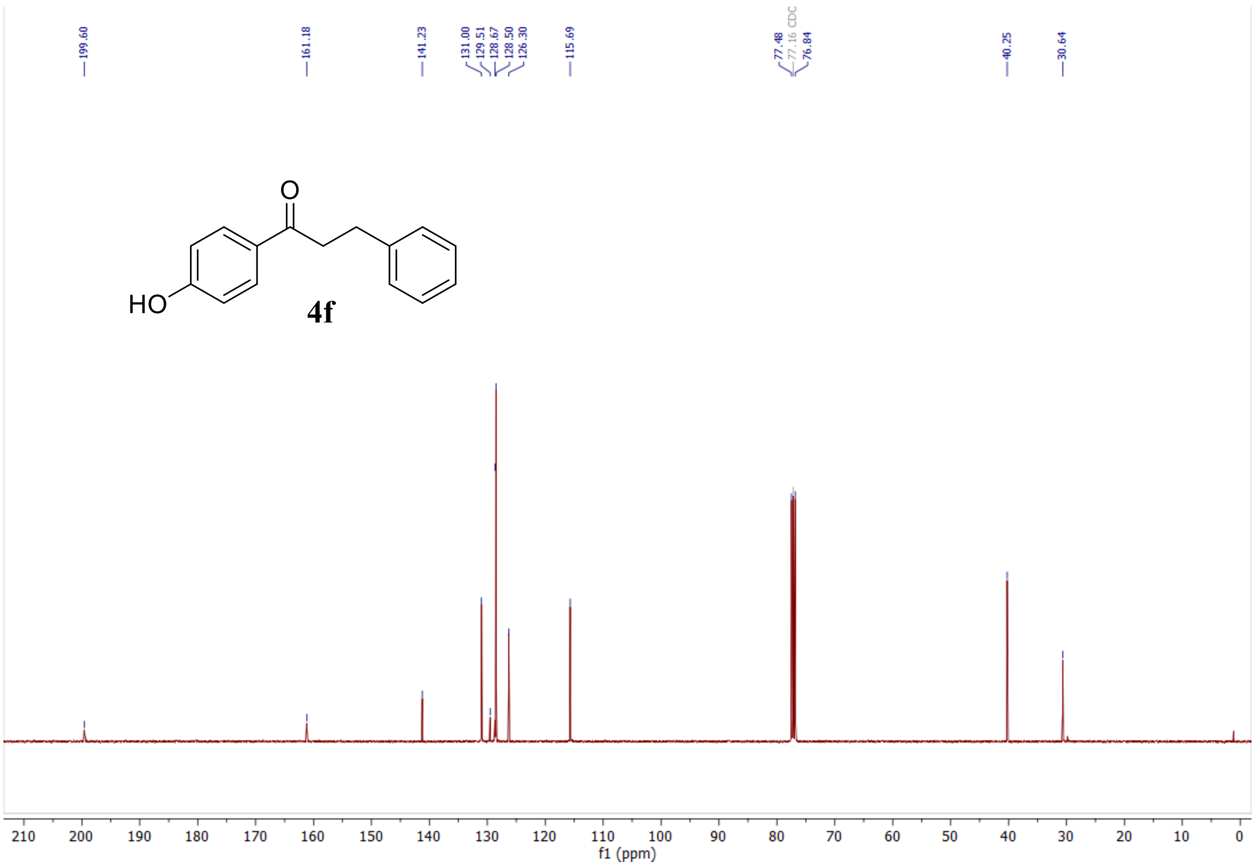

Synthesis of 1-(2,3-dihydrobenzo[*b*][1,4]dioxin-6-yl)-3-phenylpropan-1-one (4g)

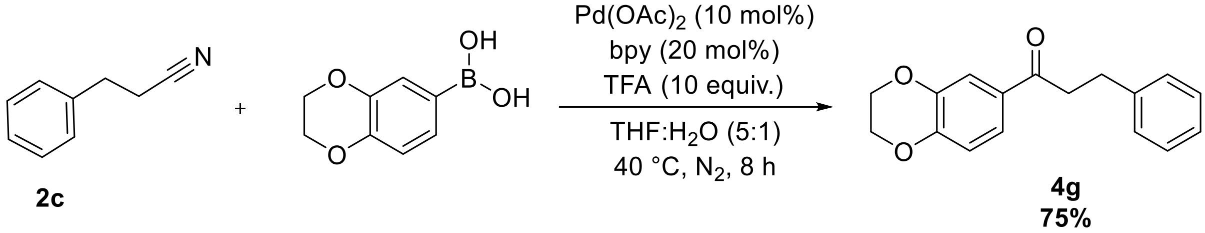

1-(2,3-dihydrobenzo[*b*][1,4]dioxin-6-yl)-3-phenylpropan-1-one **4d** was synthesized according to **GP-I**. Purification by silica gel column chromatography (1:17 EtOAc:Hex eluent) yielded the product **4g** as a dull white solid (80.5 mg, 75% yield). Analytical TLC, 1:4 EtOAc:Hex eluent, R_f = 0.45.

^{1}H NMR (400 MHz, CDCl3): δ = 7.55 – 7.47 (m, 2H), 7.34 – 7.17 (m, 5H), 6.90 (d, *J* = 8.2 Hz, 1H), 4.33 – 4.30 (m, 2H), 4.29 – 4.25 (m, 2H), 3.22 (dd, *J* = 8.9, 7.0 Hz, 2H), 3.05 (t, *J* = 7.7 Hz, 2H) ppm. **^{13}C NMR** (101 MHz, $CDCl_3$): δ = 197.83, 148.09, 143.46, 141.53, 130.89, 128.62, 128.54, 126.20, 122.27, 117.71, 117.33, 64.80, 64.24, 40.23, 30.44 ppm. **HRMS**: calcd. for $C_{17}H_{17}O_3^+$ [M+H$^+$] 269.1172; found 269.1167.

^{1}H NMR of 4g

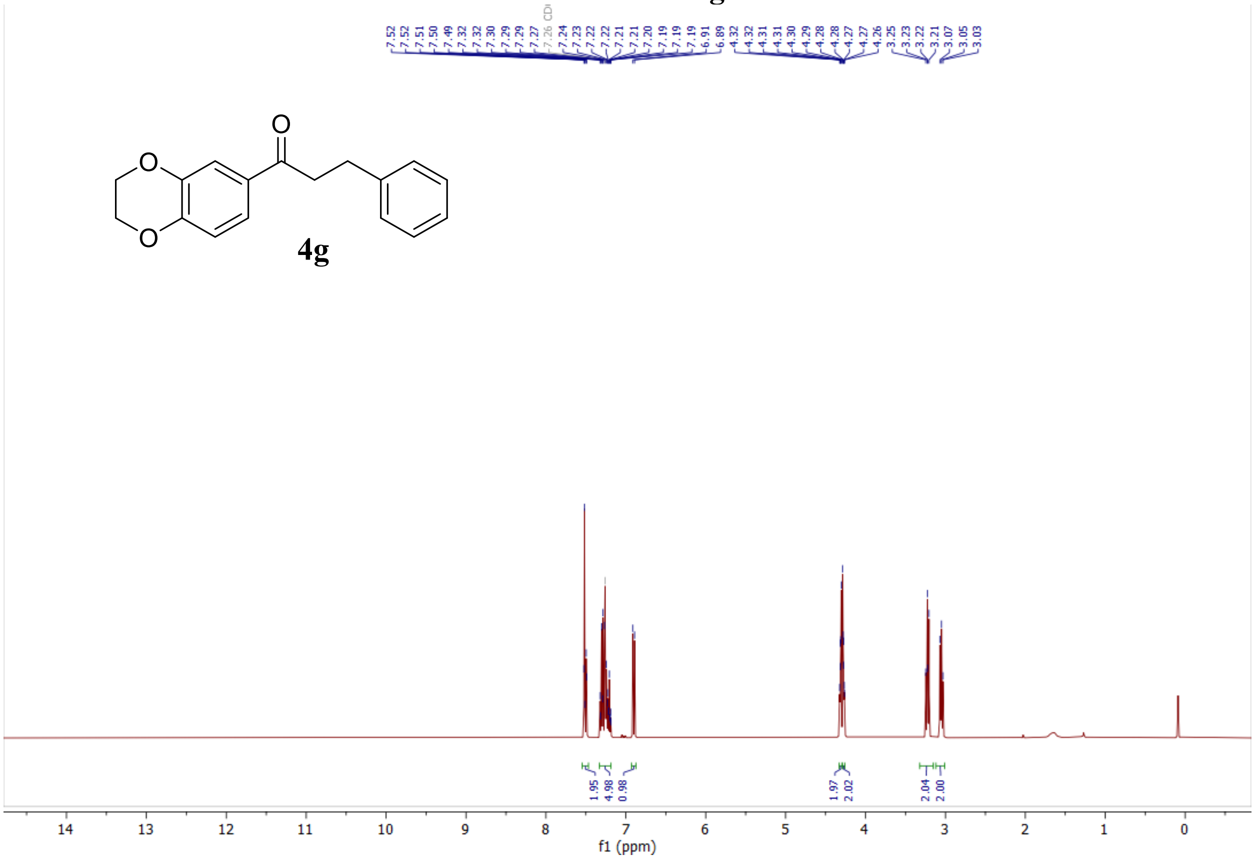

^{13}C NMR of 4g

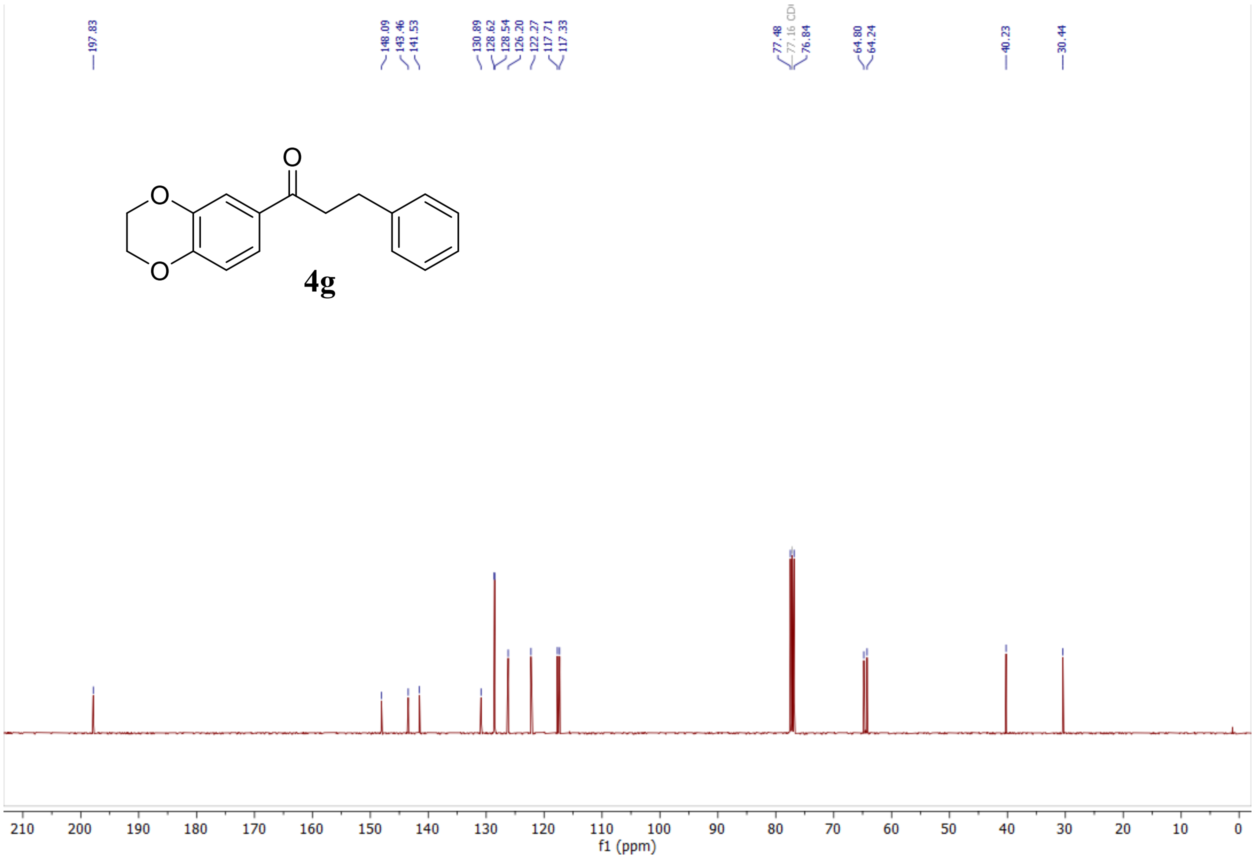

Synthesis of 1-(4-(diphenylamino)phenyl)-3-phenylpropan-1-one 4h

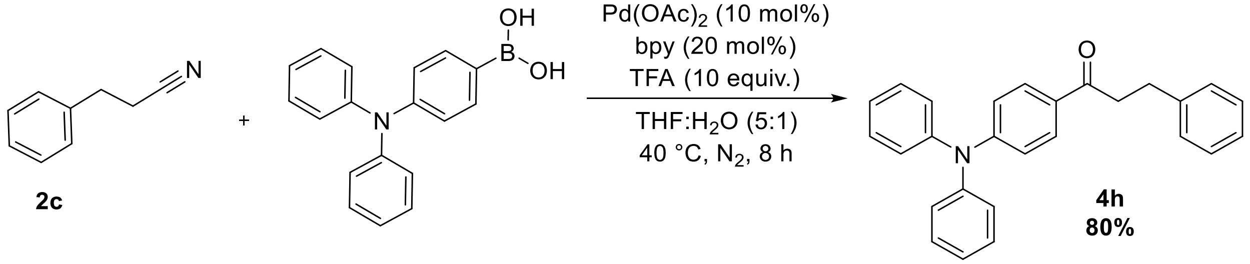

1-(4-(diphenylamino)phenyl)-3-phenylpropan-1-one **4h** was synthesized according to **GP-I**. Purification by silica gel column chromatography (1:25 EtOAc:Hex eluent) yielded the product **4h** as a dull brown-white solid (121 mg, 80% yield). Analytical TLC, 1:4 EtOAc:Hex eluent, R_f = 0.65.

^{1}H NMR (400 MHz, $CDCl_3$): δ = 7.81 (d, J = 8.9 Hz, 2H), 7.35 – 7.03 (m, 17H), 6.99 (d, J = 8.9 Hz, 2H), 3.25 – 3.k20 (m, 2H), 3.07 (t, J = 7.8 Hz, 2H) ppm. **^{13}C NMR** (101 MHz, $CDCl_3$): δ = 197.67, 152.26, 146.62, 141.70, 129.75, 129.74, 129.52, 128.61, 128.56, 126.17, 126.08, 124.76, 119.86, 40.14, 30.58 ppm. **HRMS**: calcd. for $C_{27}H_{24}NO_3^+$ [M+H$^+$] 378.1852; found 378.1840.

^{1}H NMR of 4h

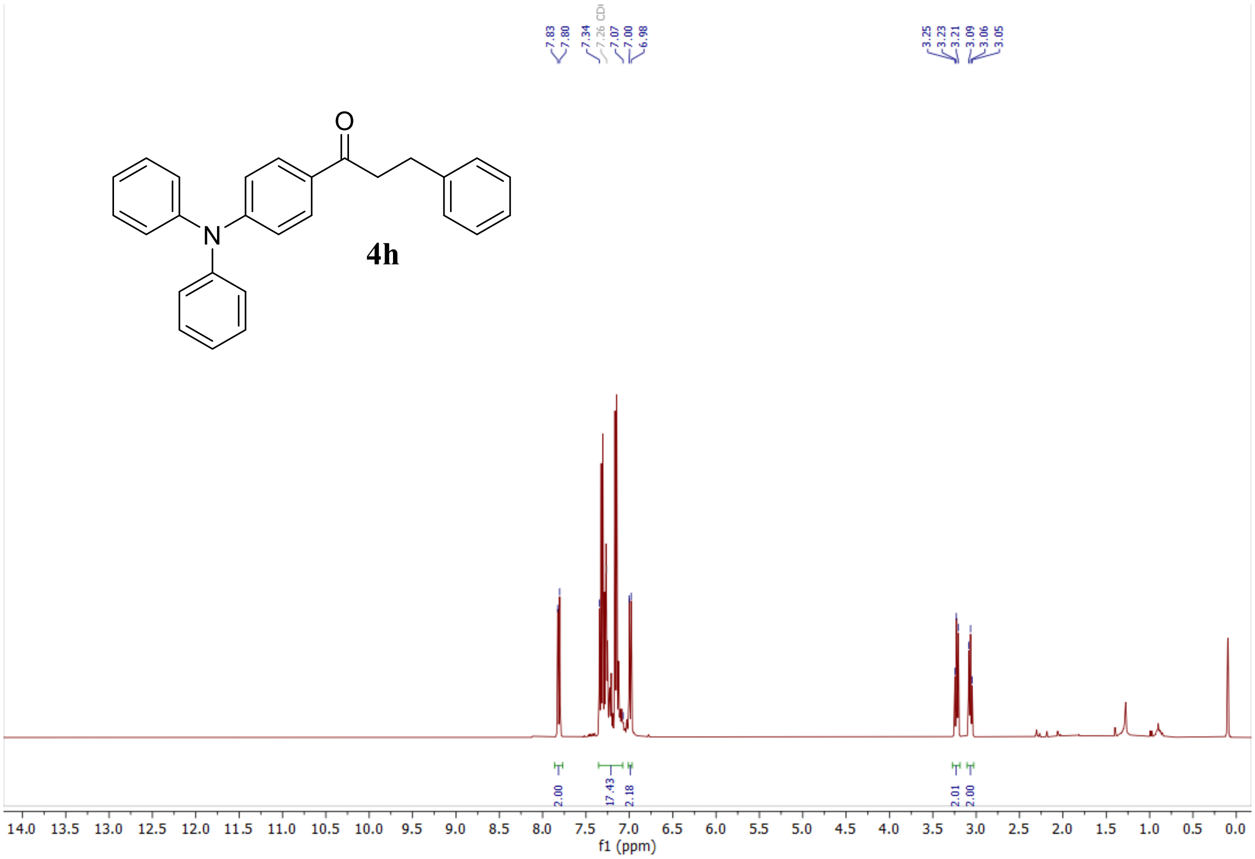

7.83
7.80
7.34
7.26 CDCl
7.07
7.00
6.98
3.25
3.23
3.21
3.09
3.06
3.05
4h
2.00
17.43
2.18
2.01
2.00
14.0 13.5 13.0 12.5 12.0 11.5 11.0 10.5 10.0 9.5 9.0 8.5 8.0 7.5 7.0 6.5 6.0 5.5 5.0 4.5 4.0 3.5 3.0 2.5 2.0 1.5 1.0 0.5 0.0
f1 (ppm)

^{13}C NMR of 4h

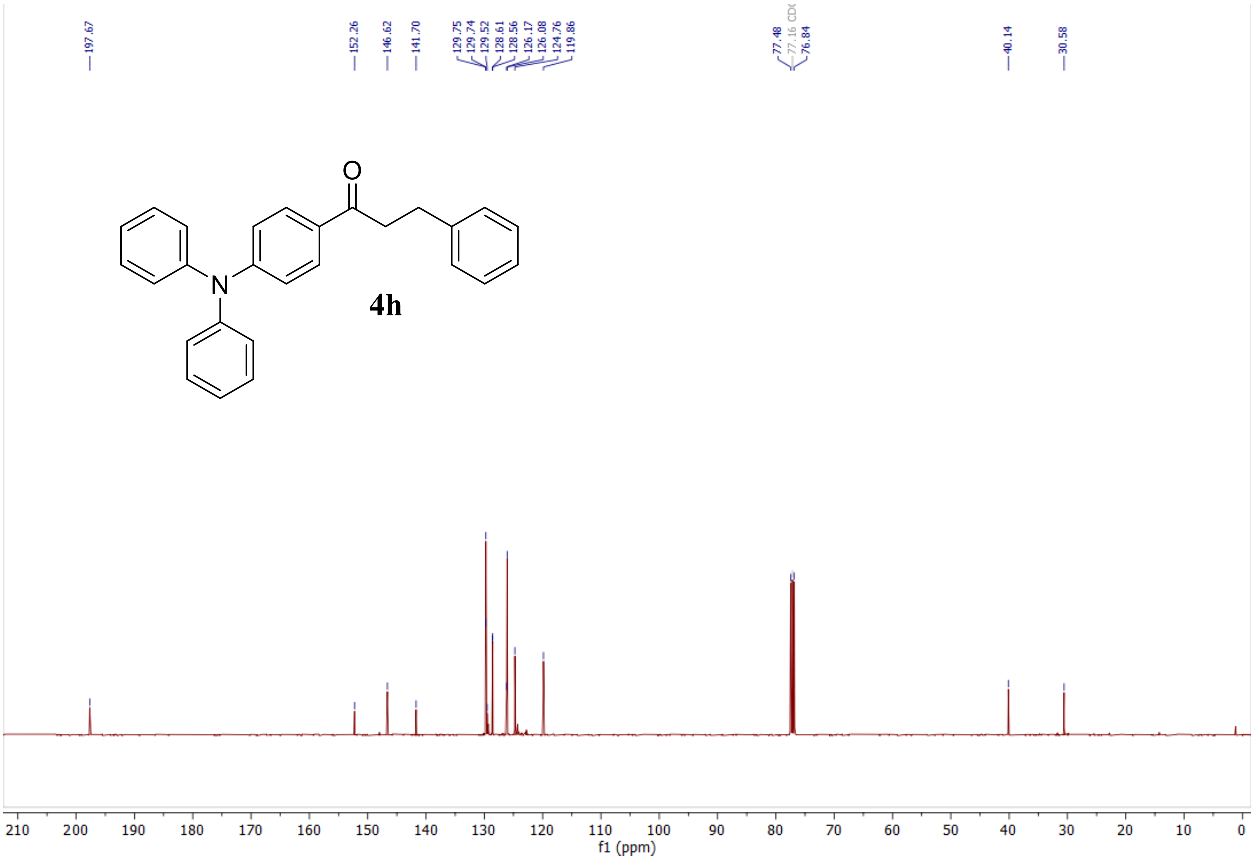

Synthesis of 1-(4-fluorophenyl)-3-phenylpropan-1-one 4i

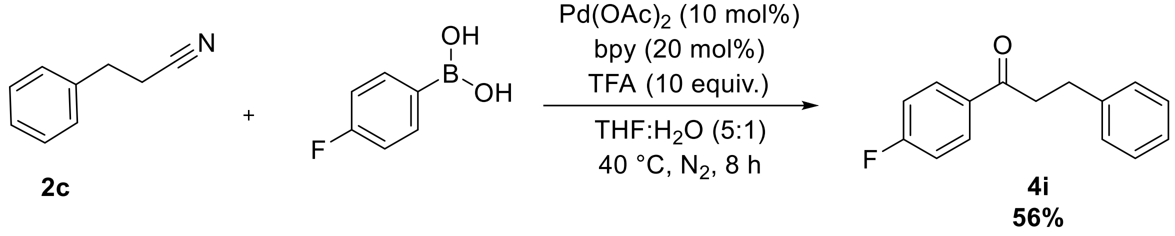

1-(4-fluorophenyl)-3-phenylpropan-1-one **4i** was synthesized according to **GP-I**. Purification by silica gel column chromatography (1:20 EtOAc:Hex eluent) yielded the product **4i** as a pale white-yellow solid (51.1 mg, 56% yield). Analytical TLC, 1:4 EtOAc:Hex eluent, $R_f = 0.60$.

^{1}H NMR (400 MHz, $CDCl_3$): δ = 8.01 – 7.95 (m, 2H), 7.33 – 7.19 (m, 5H), 7.15 – 7.09 (m, 2H), 3.30 – 3.25 (m, 2H), 3.09 – 3.04 (m, 2H) ppm. **^{13}C NMR** (101 MHz, $CDCl_3$): δ = 197.77, 167.15, 164.62, 141.28, 133.47, 133.44, 130.85, 128.71, 126.35, 115.95, 40.52, 30.26 ppm. **HRMS**: calcd. for $C_{15}H_{14}FO^+$ [M+H$^+$] 229.1023; found 229.1022.

^{1}H NMR of 4i

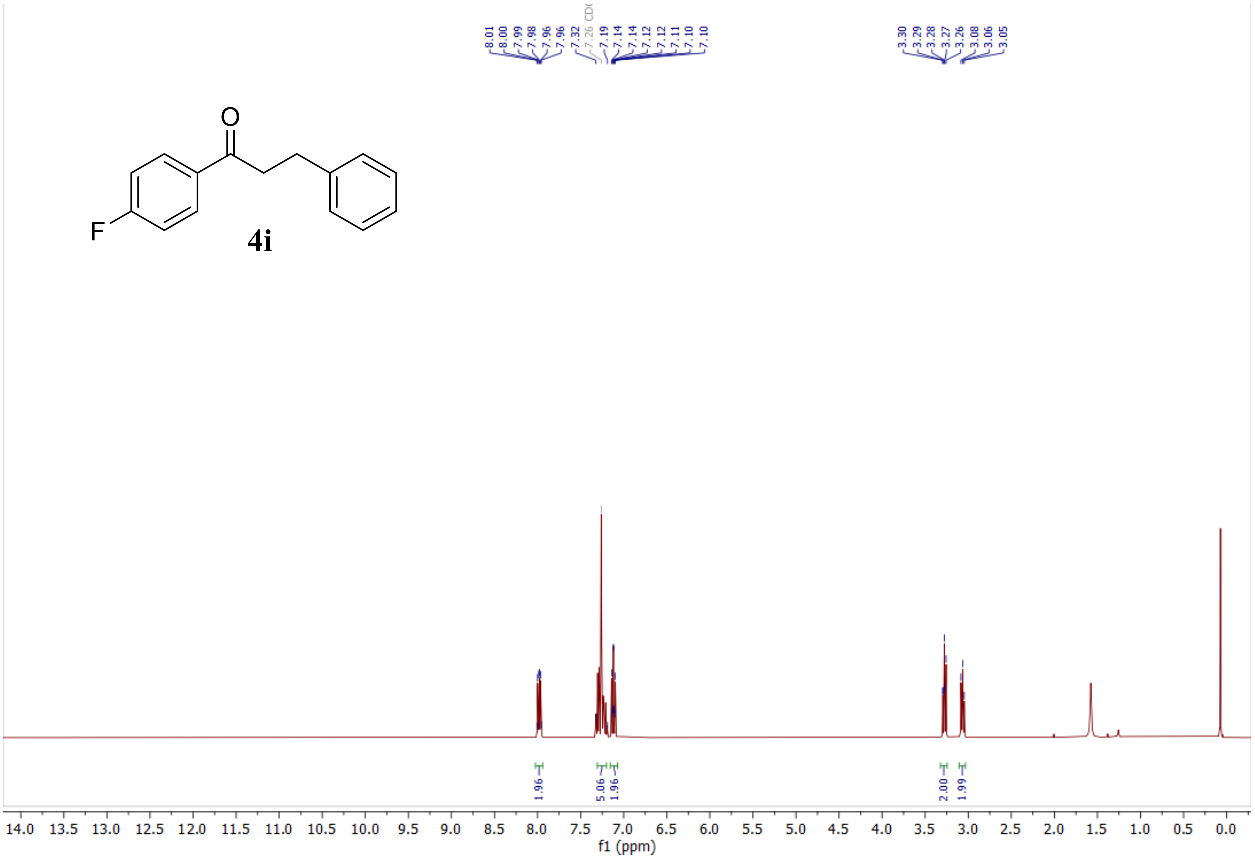

^{13}C NMR of 4i

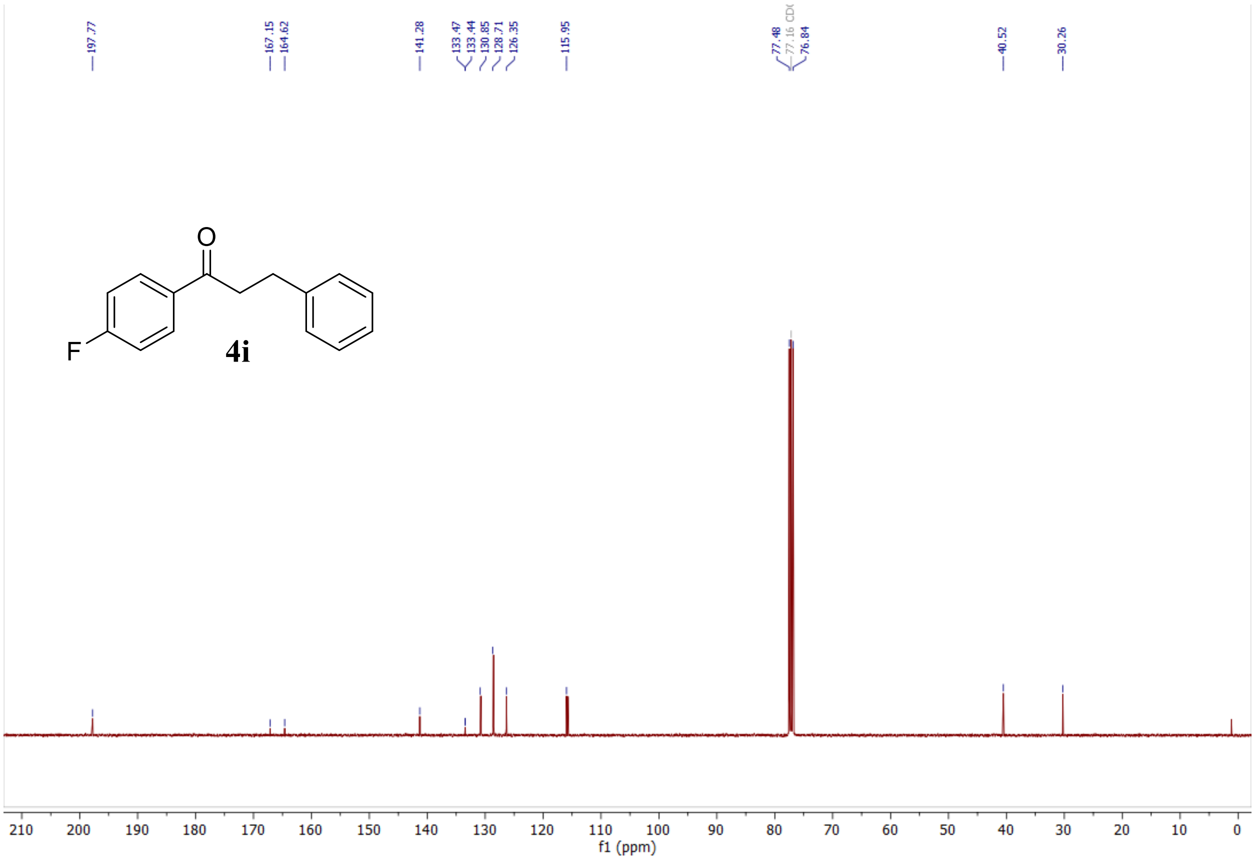

Synthesis of *N*-(3-(3-phenylpropanoyl)phenyl)methanesulfonamide 4j

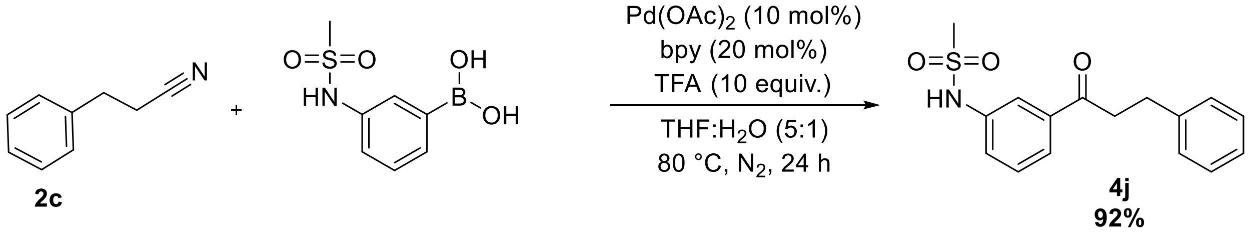

N-(3-(3-phenylpropanoyl)phenyl)methanesulfonamide **4j** was synthesized according to **GP-II**. Purification by silica gel column chromatography (1:9 EtOAc:Hex eluent) yielded the product **4j** as a flaky white solid (112 mg, 92% yield). Analytical TLC, 1:4 EtOAc:Hex eluent, $R_f = 0.35$.

^{1}**H NMR** (400 MHz, $CDCl_3$): δ = 7.82 (s, 1H), 7.76 (d, J = 7.6 Hz, 1H), 7.54 (dt, J = 8.1, 1.1 Hz, 1H), 7.44 (t, J = 7.8 Hz, 1H), 7.32 – 7.19 (m, 6H), 3.30 (dd, J = 8.3, 6.9 Hz, 1H), 3.07 (t, J = 7.6 Hz, 2H), 3.03 (s, 3H) ppm. ^{13}**C NMR** (101 MHz, $CDCl_3$): δ = 198.99, 141.07, 138.29, 130.19, 129.84, 128.68, 128.56, 126.35, 125.22, 125.01, 120.05, 40.66, 39.77, 30.15 ppm. **HRMS**: calcd. for $C_{16}H_{18}NO_3S^+$ [$M+H^+$] 304.1002; found 304.0991.

^{1}H NMR of 4j

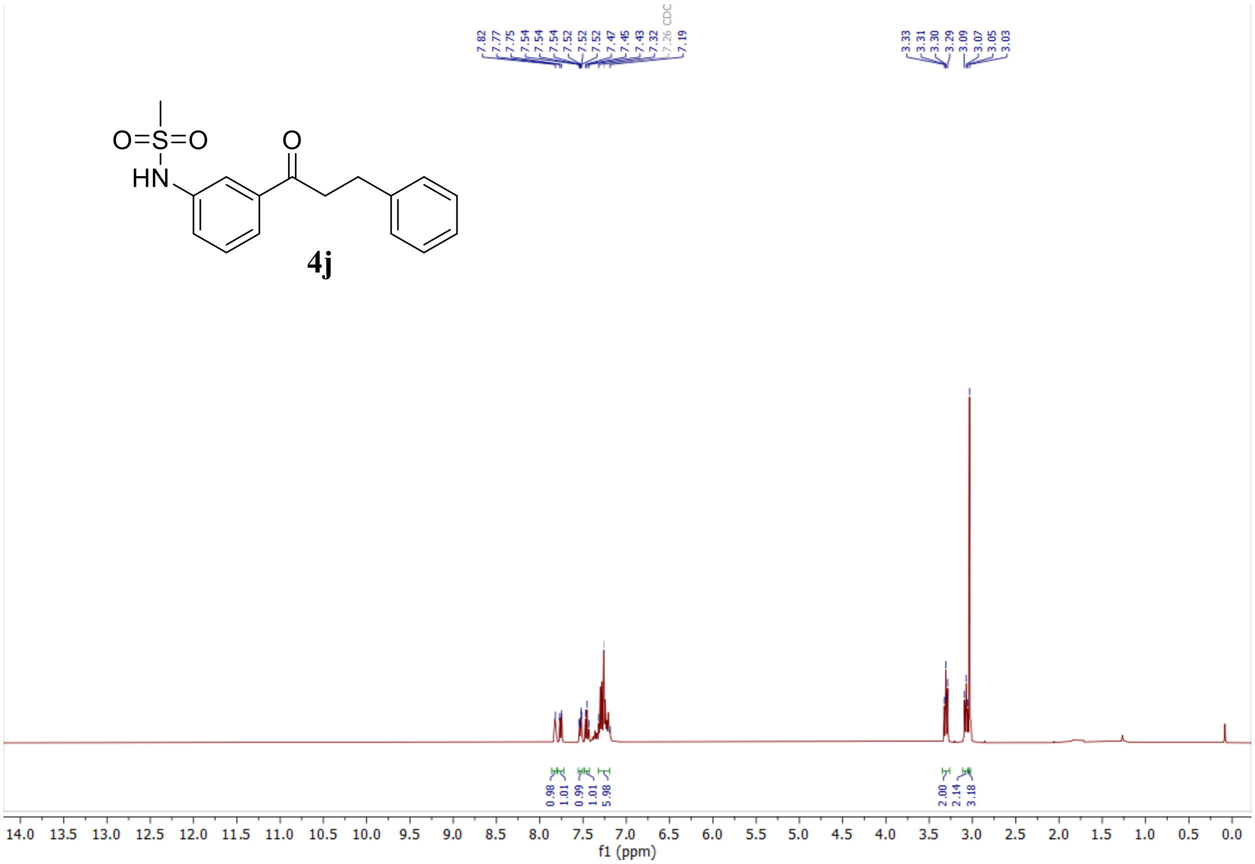

^{13}C NMR of 4j

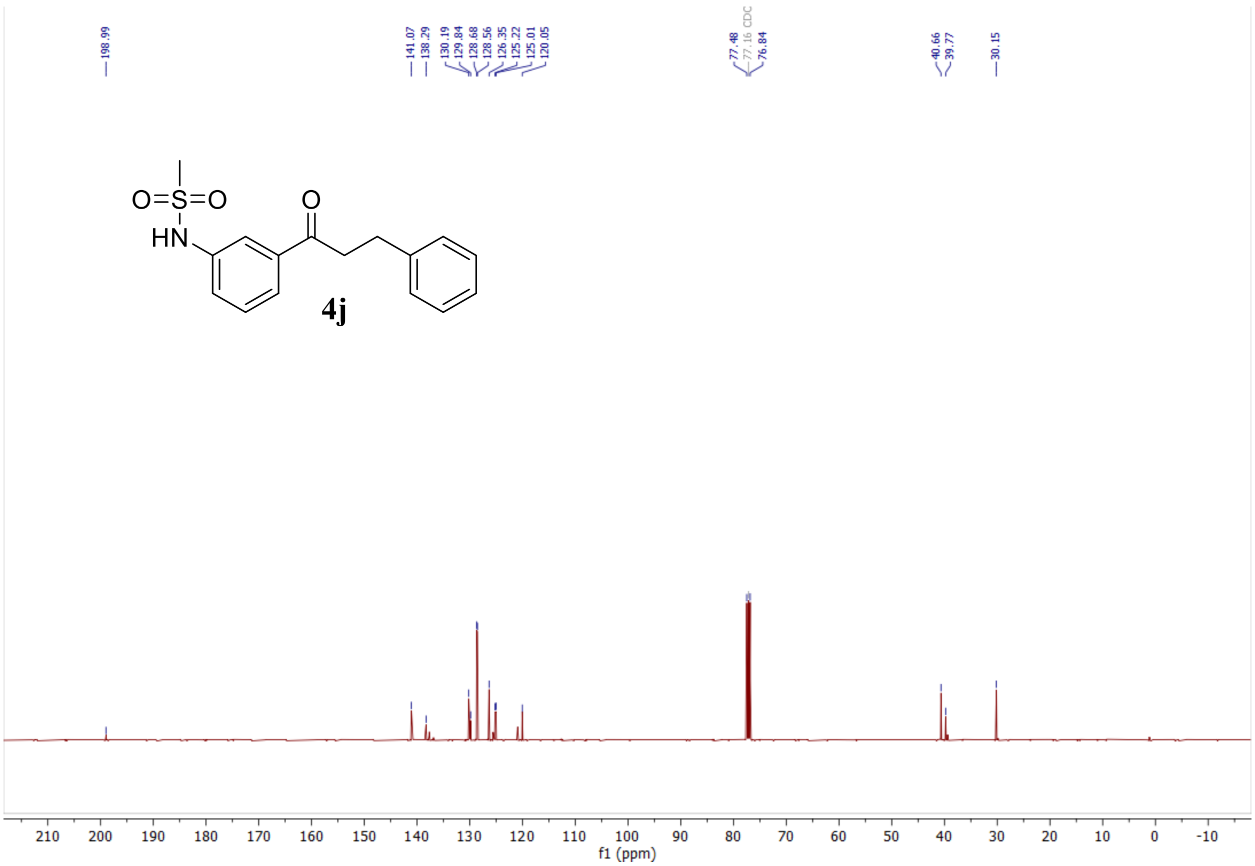

Synthesis of 1-(4-acetylphenyl)-3-phenylpropan-1-one 4k

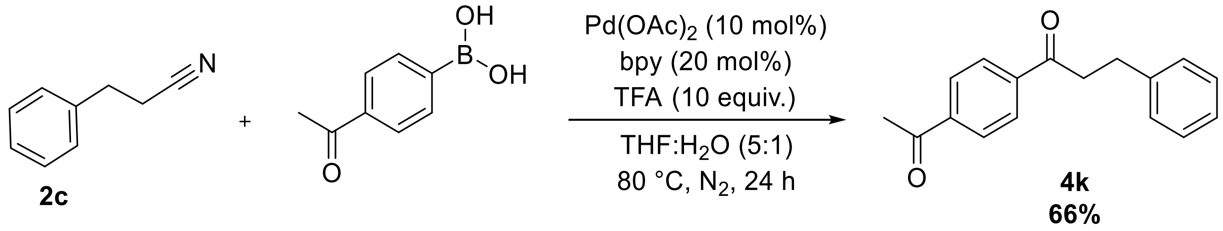

N-(3-(3-phenylpropanoyl)phenyl)methanesulfonamide **4k** was synthesized according to **GP-II**. Purification by silica gel column chromatography (1:9 EtOAc:Hex eluent) yielded the product **4k** as a white crystalline solid (66.6 mg, 66% yield). Analytical TLC, 1:4 EtOAc:Hex eluent, R_f = 0.40.

^{1}H NMR (400 MHz, $CDCl_3$): δ = 8.02 (s, 4H), 7.36 – 7.18 (m, 5H), 3.37 – 3.29 (m, 2H), 3.08 (t, *J* = 7.6 Hz, 2H), 2.64 (s, 3H) ppm. **^{13}C NMR** (101 MHz, $CDCl_3$): δ = 198.82, 197.63, 141.08, 140.27, 140.12, 128.72, 128.65, 128.55, 128.37, 126.40, 40.97, 30.12, 27.01 ppm. **HRMS**: calcd. for $C_{17}H_{17}O_2^+$ [M+H$^+$] 253.1223; found 253.1221.

^{1}H NMR of 4k

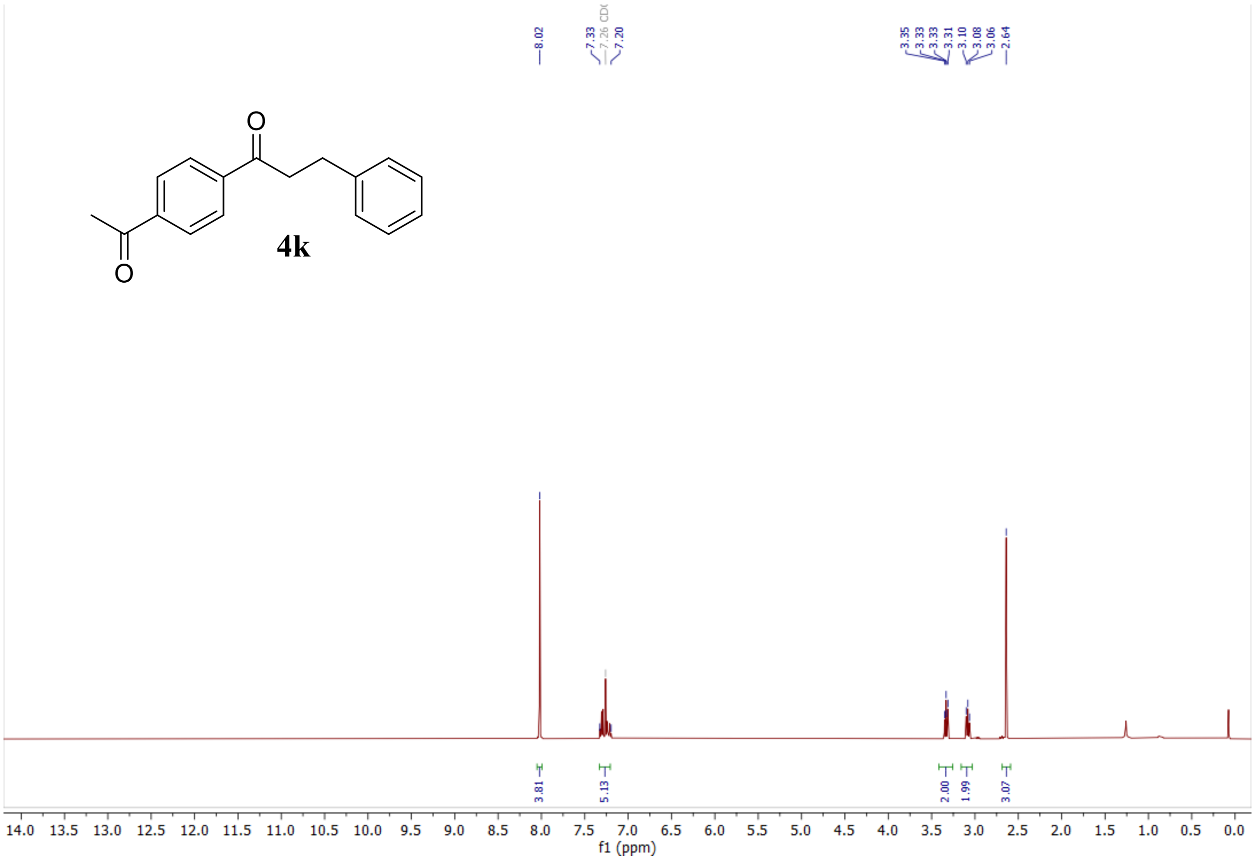

^{13}C NMR of 4k

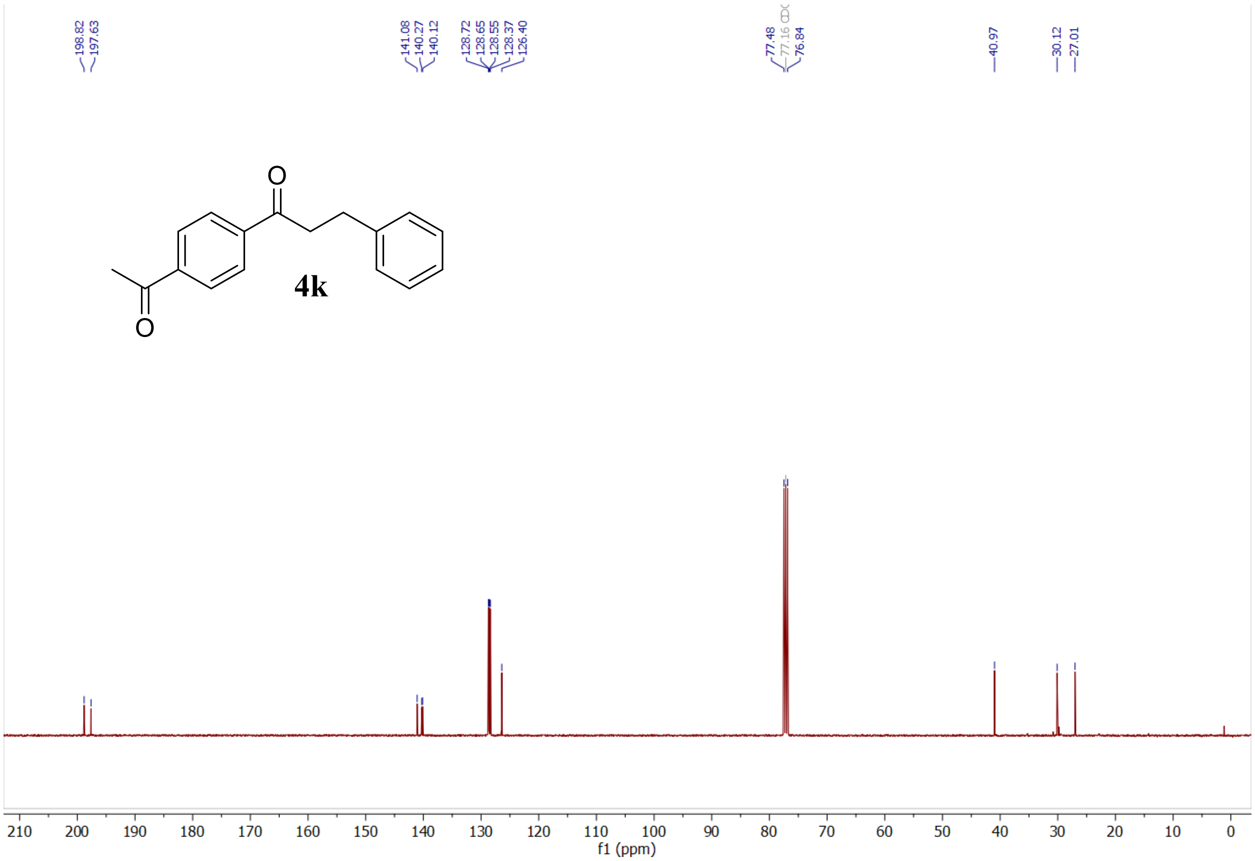
198.82
197.63
141.08
140.27
140.12
128.72
128.65
128.55
128.37
126.40
77.48
77.16 CDCl3
76.84
40.97
30.12
27.01
4k
210
200
190
180
170
160
150
140
130
120
110
100
90
80
70
60
50
40
30
20
10
0
f1 (ppm)

Synthesis of 3-phenyl-1-(2-(trifluoromethyl)phenyl)propan-1-one 4l

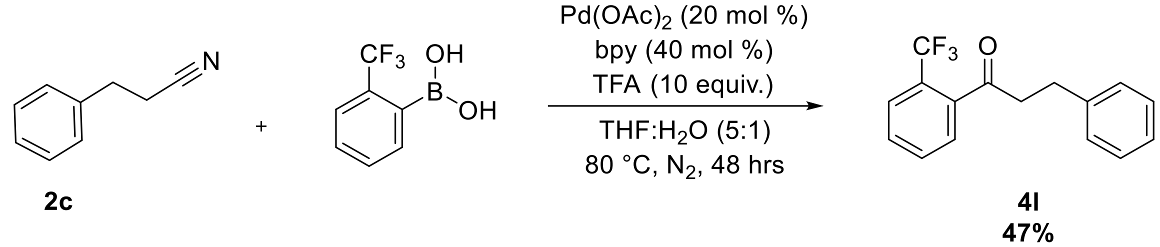

3-phenyl-1-(2-(trifluoromethyl)phenyl)propan-1-one **4l** was synthesized according to **GP-III**. Purification by silica gel column chromatography (1:14 EtOAc:Hex eluent) yielded the product **4l** as a gummy, pale yellow solid (52.3 mg, 47% yield). Analytical TLC, 1:4 EtOAc:Hex eluent, R_f = 0.55.

^{1}H NMR (400 MHz, $CDCl_3$): δ = 7.72 – 7.69 (m, 1H), 7.58 – 7.53 (m, 2H), 7.33 – 7.18 (m, 5H), 3.20 – 3.14 (m, 2H), 3.08 – 3.02 (m, 2H) ppm. **^{13}C NMR** (101 MHz, $CDCl_3$): δ = 203.58, 140.77, 131.96, 130.14, 129.04, 128.70, 128.54, 128.40, 126.98, 126.87, 126.82, 126.40, 45.05, 30.01 ppm. **HRMS**: calcd. for $C_{16}H_{14}F_3O^+$ [M+H$^+$] 279.0990; found 279.0991.

^{1}H NMR of 4l

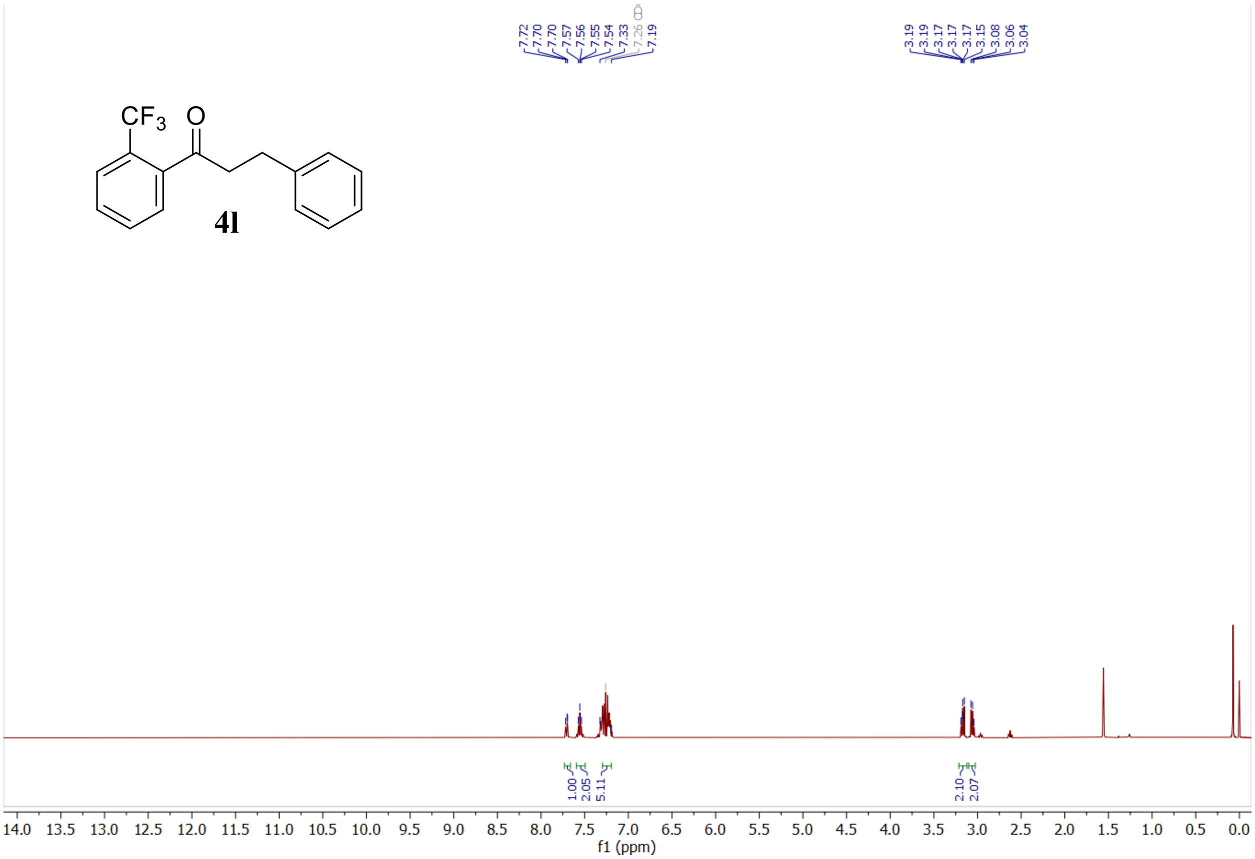

^{13}C NMR of 4l

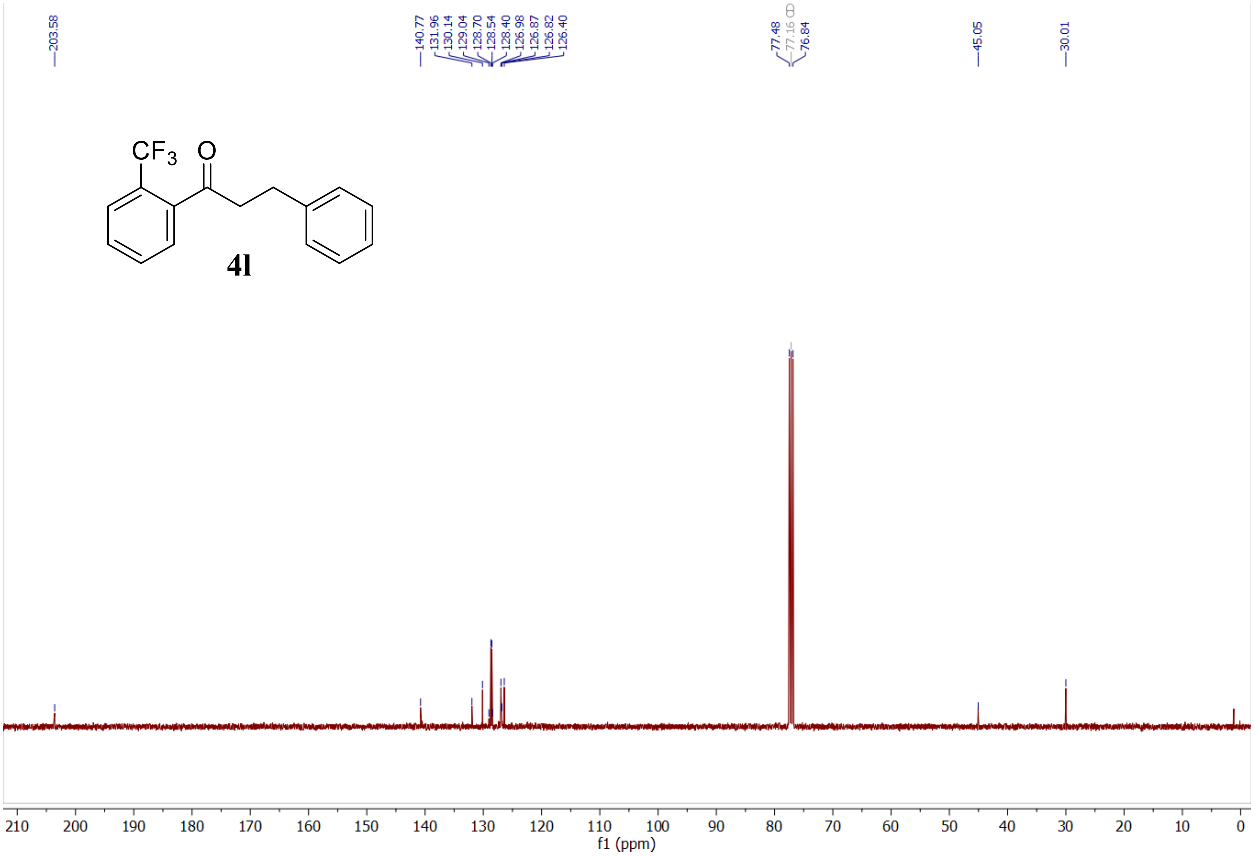

Synthesis of 1-(3-nitrophenyl)-3-phenylpropan-1-one 4m

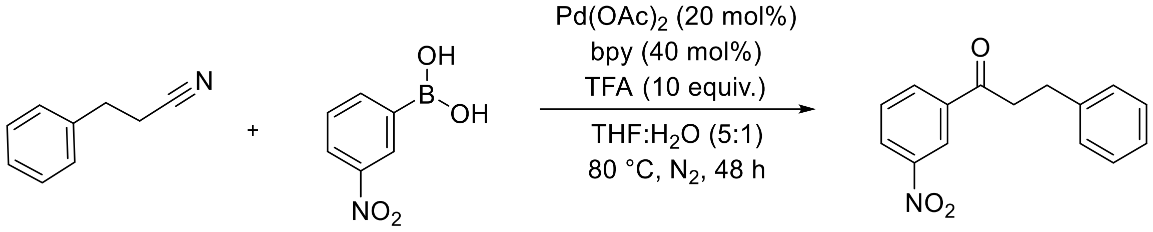

1-(3-nitrophenyl)-3-phenylpropan-1-one **4m** was synthesized according to **GP- III**. Purification by silica gel column chromatography (1:15 EtOAc:Hex eluent) yielded the product **4m** as a white-yellow solid (65.3 mg, 64% yield). Analytical TLC, 1:4 EtOAc:Hex eluent, R_f = 0.50.

^{1}H NMR (400 MHz, $CDCl_3$): δ = 8.76 (t, J = 2.0 Hz, 1H), 8.41 (ddd, J = 8.2, 2.3, 1.1 Hz, 1H), 8.28 (dt, J = 7.7, 1.3 Hz, 1H), 7.67 (t, J = 8.0 Hz, 1H), 7.27 (d, J = 55.1 Hz, 5H), 3.36 (t, J = 7.6 Hz, 2H), 3.11 (t, J = 7.5 Hz, 2H) ppm. **^{13}C NMR** (101 MHz, $CDCl_3$): δ = 197.08, 148.61, 140.75, 138.21, 133.69, 130.05, 128.80, 128.57, 127.51, 126.54, 123.11, 77.48, 76.84, 40.84, 30.00 ppm. **HRMS**: calcd. for $C_{15}H_{14}NO_3^+$ [M+H$^+$] 256.0968; found 256.0966.

^{1}H NMR of 4m

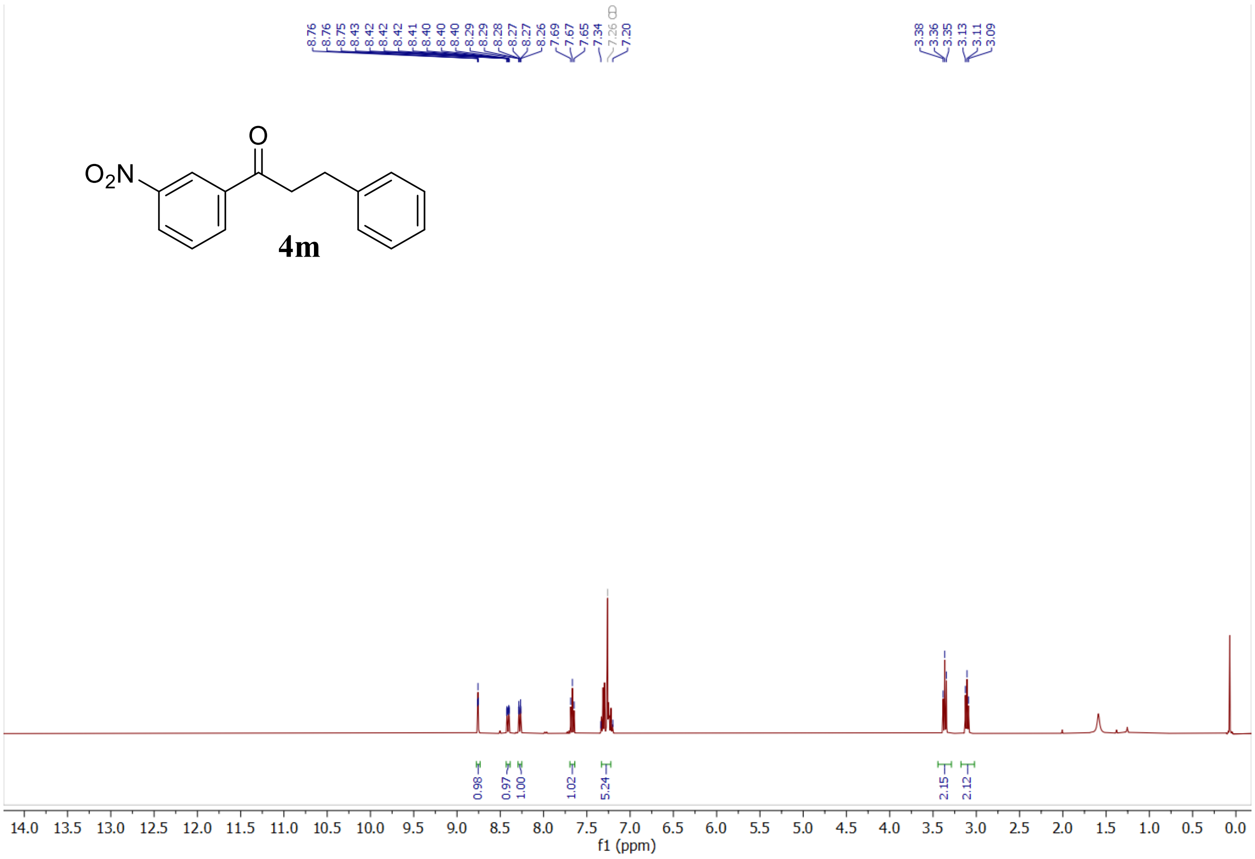

O
O_2N
4m
8.76
8.76
8.75
8.43
8.42
8.42
8.42
8.41
8.40
8.40
8.40
8.29
8.29
8.28
8.27
8.27
8.26
7.69
7.67
7.65
7.34
7.26
7.20
3.38
3.36
3.35
3.13
3.11
3.09
0.98
0.97
1.00
1.02
5.24
2.15
2.12
14.0 13.5 13.0 12.5 12.0 11.5 11.0 10.5 10.0 9.5 9.0 8.5 8.0 7.5 7.0 6.5 6.0 5.5 5.0 4.5 4.0 3.5 3.0 2.5 2.0 1.5 1.0 0.5 0.0
f1 (ppm)

^{13}C NMR of 4m

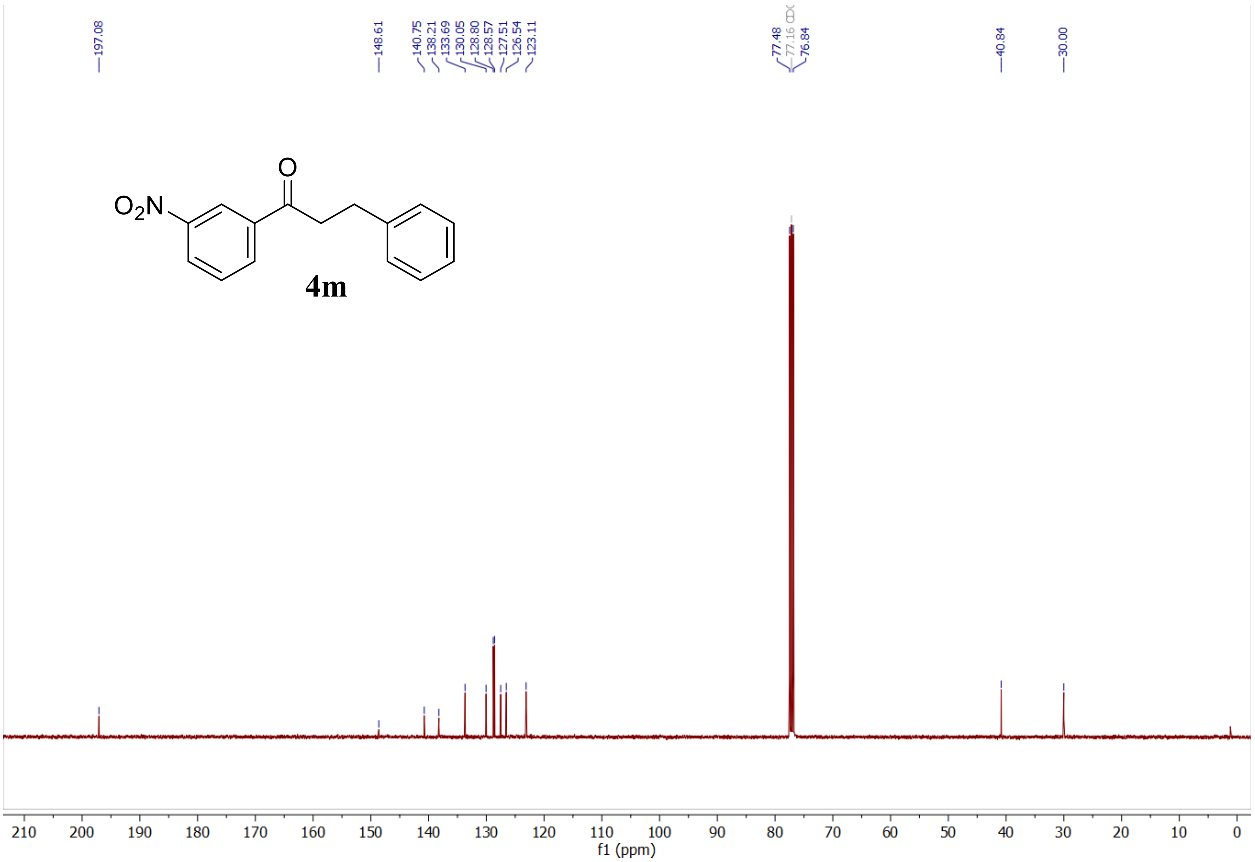

Synthesis of 3-phenyl-1-(thiophen-3-yl)propan-1-one 4n

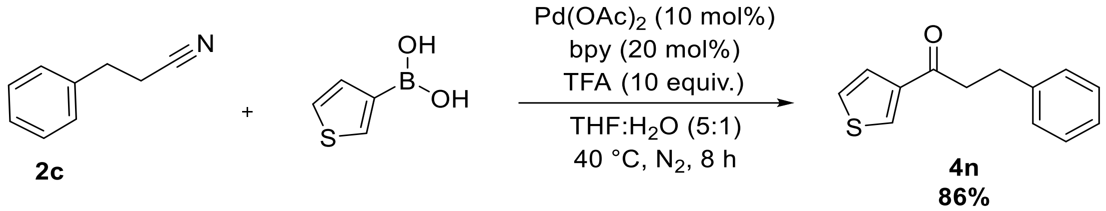

3-phenyl-1-(thiophen-3-yl)propan-1-one **4n** was synthesized according to **GP-I**. Purification by silica gel column chromatography (1:25 EtOAc:Hex eluent) yielded the product **4n** as a pale white solid (74.4 mg, 86% yield). Analytical TLC, 1:4 EtOAc:Hex eluent, $R_f = 0.70$.

^{1}H NMR (400 MHz, $CDCl_3$): δ = 8.03 (dd, *J* = 2.9, 1.3 Hz, 1H), 7.55 (dd, *J* = 5.1, 1.3 Hz, 1H), 7.33 – 7.18 (m, 6H), 3.24 – 3.19 (m, 2H), 3.06 (t, *J* = 7.7 Hz, 2H) ppm. **^{13}C NMR** (101 MHz, $CDCl_3$): δ = 193.71, 142.32, 141.33, 131.96, 128.67, 128.54, 127.04, 126.50, 126.29, 41.80, 30.22 ppm. **HRMS**: calcd. for $C_{13}H_{13}OS^+$ [M+H$^+$] 217.0682; found 217.0677.

¹H NMR of 4n

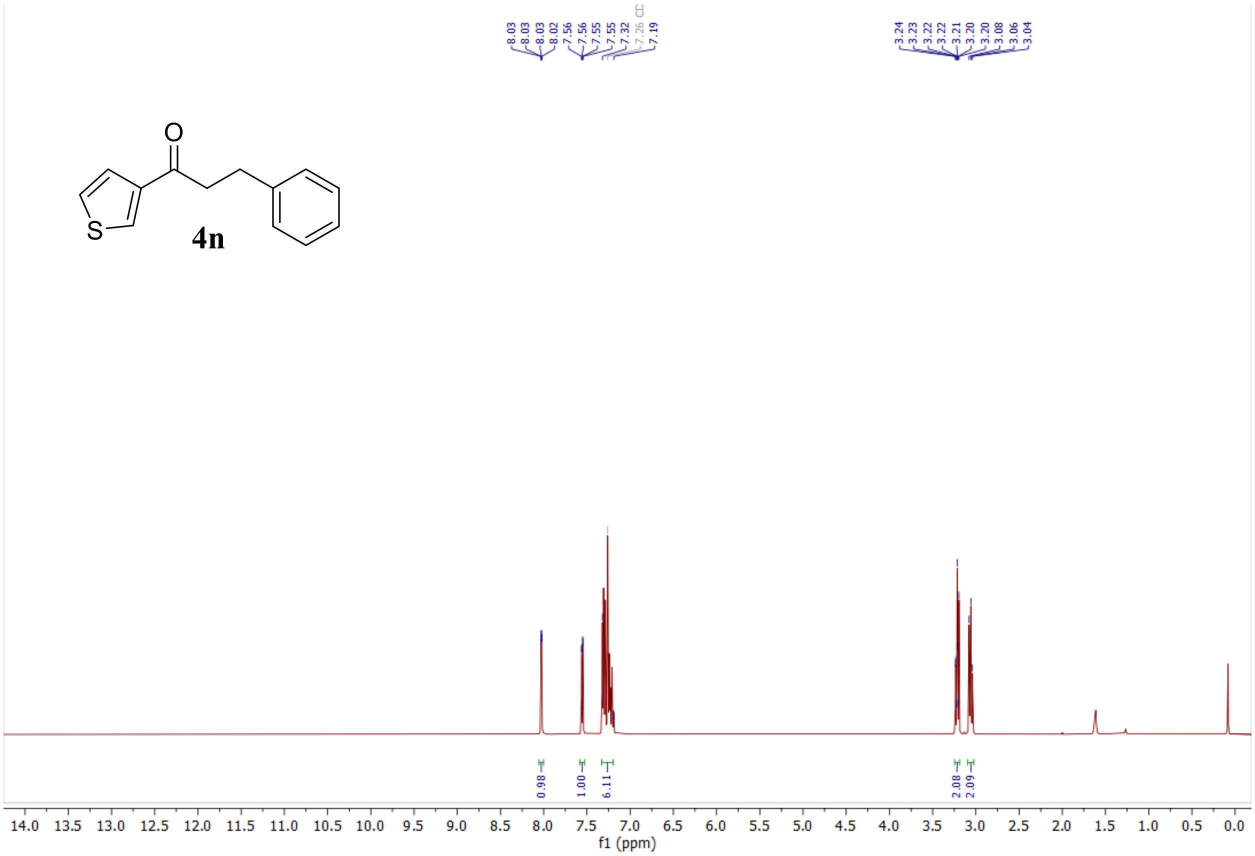
O
S
4n
8.03
8.03
8.03
8.02
7.56
7.55
7.55
7.32
7.26 CDCl3
7.19
3.24
3.23
3.22
3.22
3.21
3.20
3.20
3.08
3.06
3.04
0.98
1.00
6.11
2.08
2.09
14.0 13.5 13.0 12.5 12.0 11.5 11.0 10.5 10.0 9.5 9.0 8.5 8.0 7.5 7.0 6.5 6.0 5.5 5.0 4.5 4.0 3.5 3.0 2.5 2.0 1.5 1.0 0.5 0.0
f1 (ppm)

^{13}C NMR of 4n

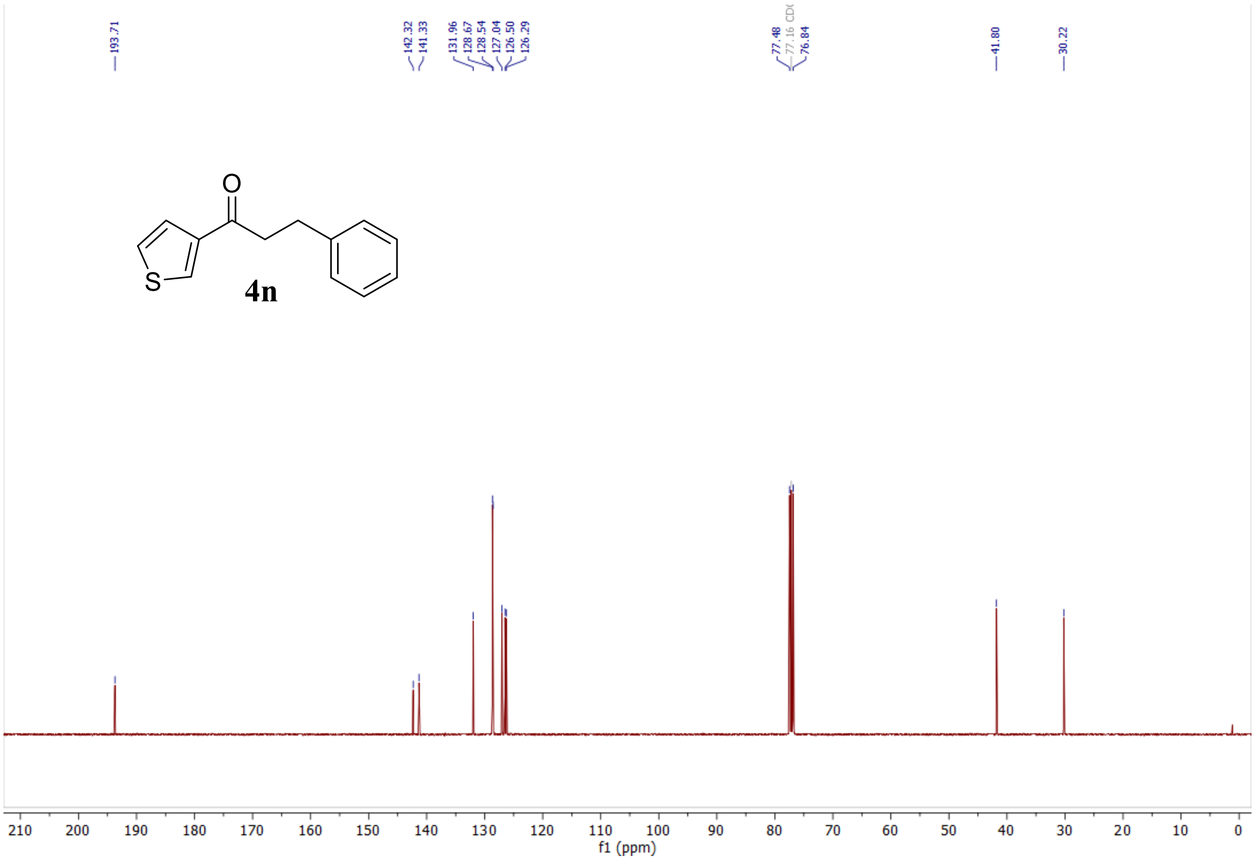

Synthesis of 1-(furan-3-yl)-3-phenylpropan-1-one 4o

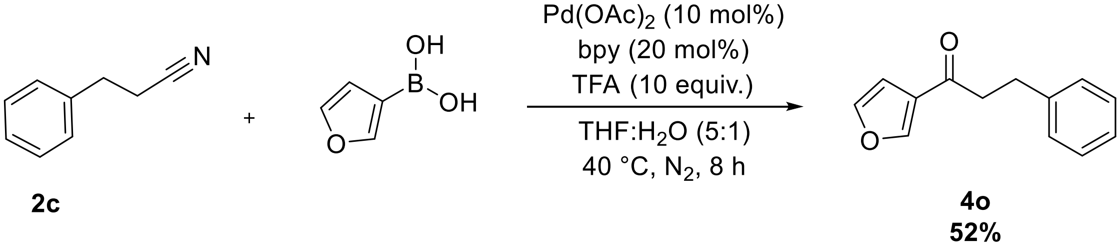

1-(furan-3-yl)-3-phenylpropan-1-one **4o** was synthesized according to **GP-I**. Purification by silica gel column chromatography (1:15 EtOAc:Hex eluent) yielded the product **4o** as a bright yellow solid (41.6 mg, 52% yield). Analytical TLC, 1:4 EtOAc:Hex eluent, $R_f = 0.55$.

^{1}H NMR (400 MHz, $CDCl_3$): δ = 8.01 – 7.96 (m, 1H), 7.43 (t, *J* = 1.7 Hz, 1H), 7.33 – 7.18 (m, 6H), 6.77 (dd, *J* = 2.0, 0.9 Hz, 1H), 3.10 – 3.02 (m, 4H) ppm. **^{13}C NMR** (101 MHz, $CDCl_3$): δ = 194.13, 147.23, 144.34, 141.18, 128.68, 128.52, 127.78, 126.33, 108.73, 42.30, 30.16 ppm. **HRMS**: calcd. for $C_{13}H_{13}O_2^+$ [M+H$^+$] 201.0910; found 201.0907.

^{1}H NMR of 4o

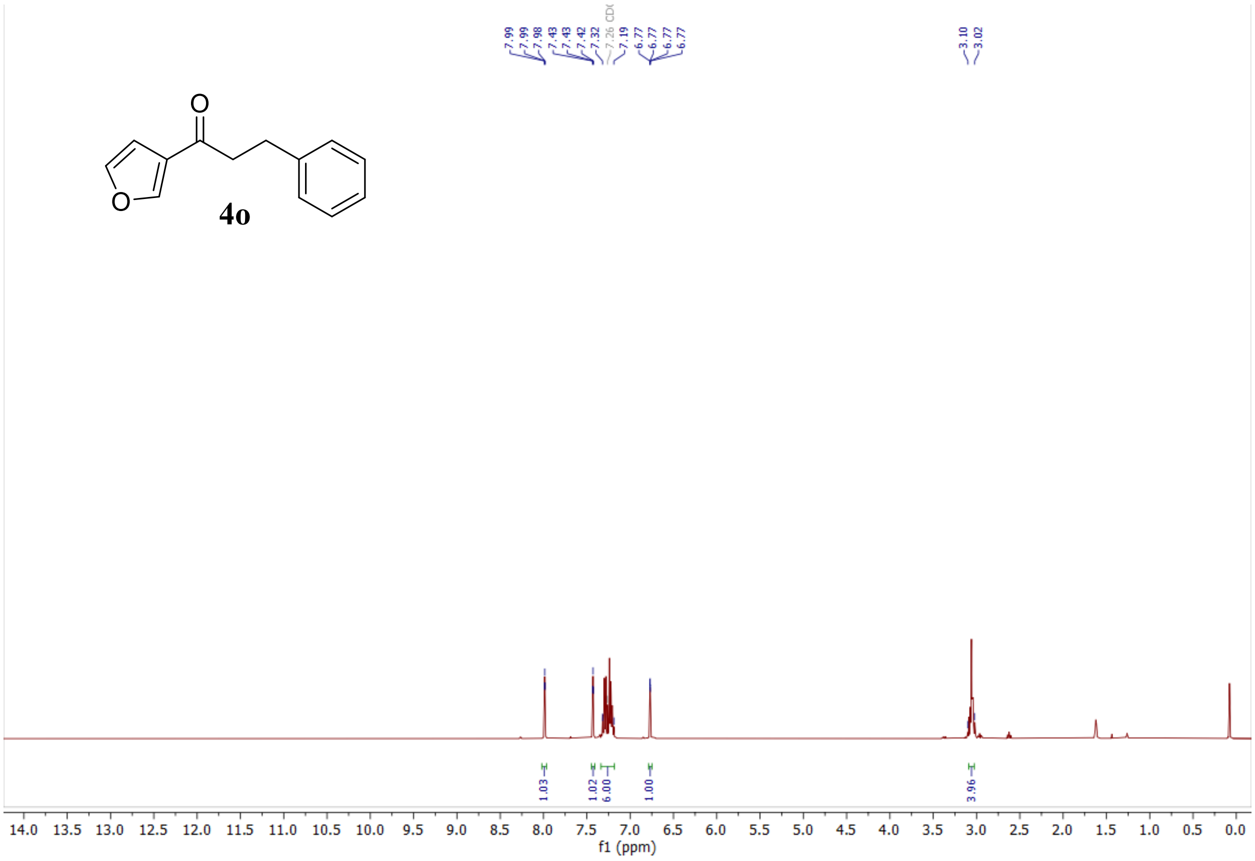

4o
7.99
7.99
7.98
7.43
7.43
7.42
7.32
7.26 CDC
7.19
6.77
6.77
6.77
6.77
3.10
3.02
1.03
1.02
6.00
1.00
3.96
14.0 13.5 13.0 12.5 12.0 11.5 11.0 10.5 10.0 9.5 9.0 8.5 8.0 7.5 7.0 6.5 6.0 5.5 5.0 4.5 4.0 3.5 3.0 2.5 2.0 1.5 1.0 0.5 0.0
f1 (ppm)

^{13}C NMR of 4o

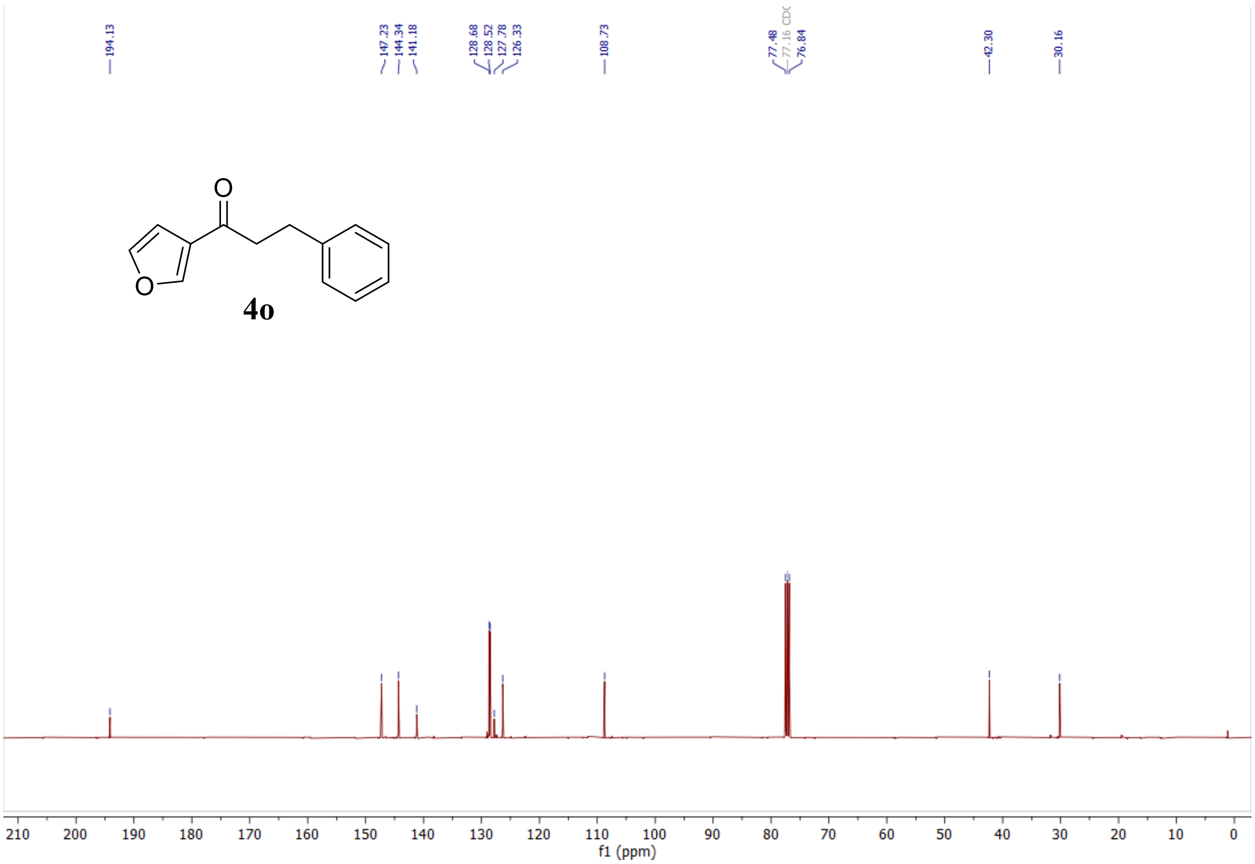

Synthesis of 1-(1*H*-indol-5-yl)-3-phenylpropan-1-one 4p

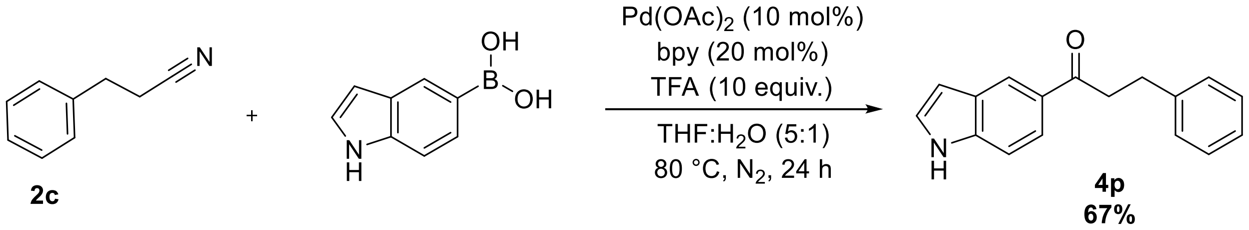

1-(1*H*-indol-5-yl)-3-phenylpropan-1-one **4p** was synthesized according to **GP-II**. Purification by silica gel column chromatography (1:8 EtOAc:Hex eluent) yielded the product **4p** as a bright red solid (66.8 mg, 67% yield). Analytical TLC, 1:4 EtOAc:Hex eluent, R_f = 0.40.

^{1}H NMR (400 MHz, $CDCl_3$): δ = 8.46 (s, 1H), 8.36 – 8.31 (m, 1H), 7.90 (dd, *J* = 8.6, 1.7 Hz, 1H), 7.42 (dt, *J* = 8.6, 0.9 Hz, 1H), 7.34 – 7.27 (m, 5H), 7.24 – 7.19 (m, 1H), 6.66 (ddd, *J* = 3.1, 2.0, 1.0 Hz, 1H), 3.42 – 3.37 (m, 2H), 3.14 – 3.09 (m, 2H) ppm. **^{13}C NMR** (101 MHz, $CDCl_3$): δ = 199.54, 141.82, 138.57, 129.80, 128.63, 128.60, 127.58, 126.17, 125.80, 122.76, 122.36, 111.16, 104.48, 40.49, 30.75 ppm. **HRMS**: calcd. for $C_{17}H_{16}NO^+$ [M+H$^+$] 250.1226; found 250.1221.

^{1}H NMR of 4p

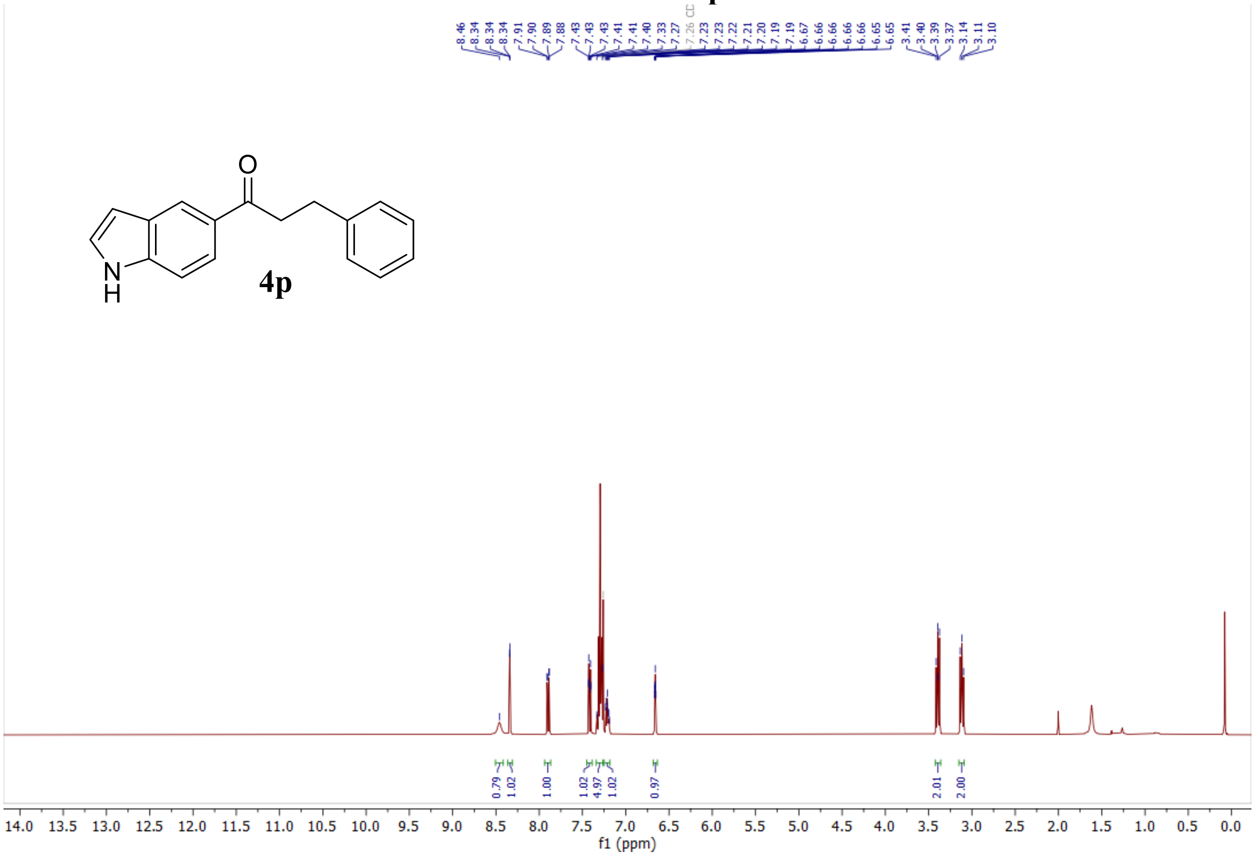

^{13}C NMR of 4p

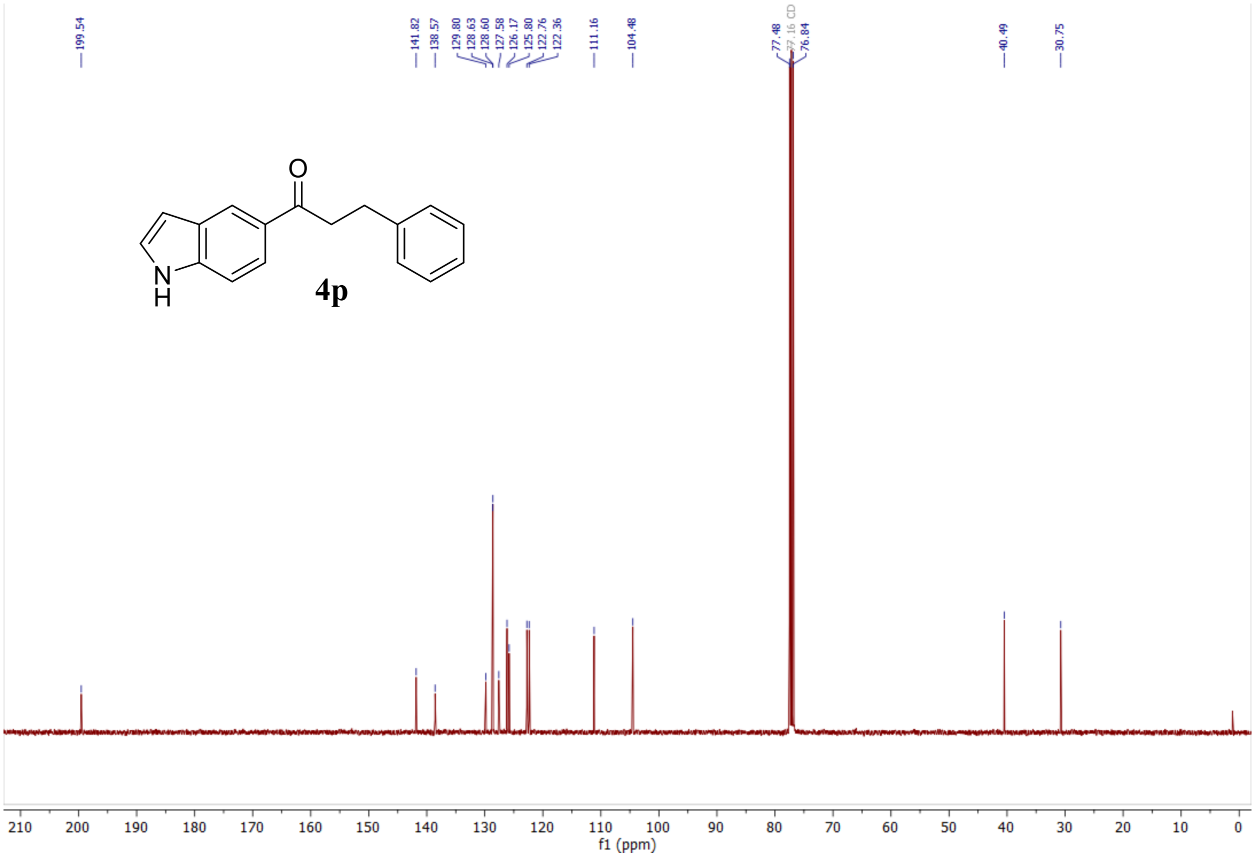

Synthesis of 1-(4-(butylthio)phenyl)-3-phenylpropan-1-one 4q

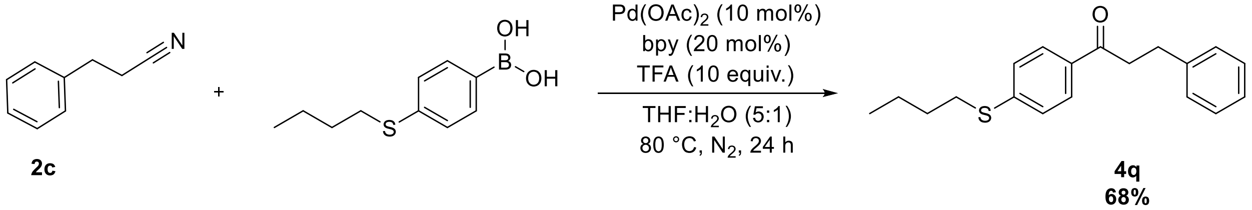

1-(4-(butylthio)phenyl)-3-phenylpropan-1-one **4q** was synthesized according to **GP-II**. Purification by silica gel column chromatography (1:50 EtOAc:Hex eluent) yielded the product **4q** as a powdery white solid (81.2 mg, 68% yield). Analytical TLC, 1:4 EtOAc:Hex eluent, R_f = 0.70.

^{1}H NMR (400 MHz, $CDCl_3$): δ = 7.87 – 7.84 (m, 2H), 7.32 – 7.19 (m, 7H), 3.31 – 3.25 (m, 2H), 3.08 – 3.04 (m, 2H), 3.01 – 2.97 (m, 2H), 1.69 (tt, J = 8.2, 6.8 Hz, 2H), 1.48 (dq, J = 14.3, 7.3 Hz, 2H), 0.95 (t, J = 7.3 Hz, 3H) ppm. **^{13}C NMR** (101 MHz, $CDCl_3$): δ = 198.41, 145.08, 141.46, 133.51, 128.66, 128.60, 128.56, 126.43, 126.26, 40.38, 31.79, 30.93, 30.35, 22.16, 13.76 ppm. **HRMS**: calcd. for $C_{19}H_{23}OS^+$ [M+H$^+$] 299.1464; found 299.1459.

^{1}H NMR of 4q

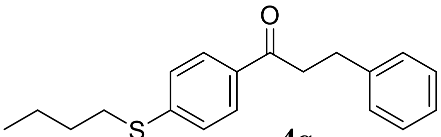

4q

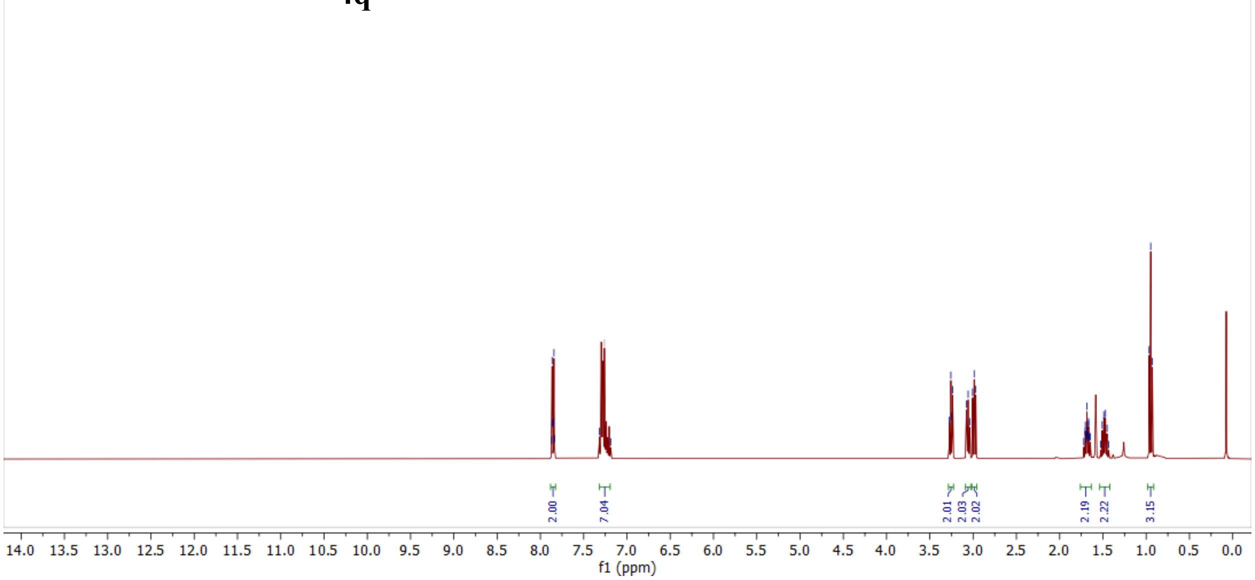

^{13}C NMR of 4q

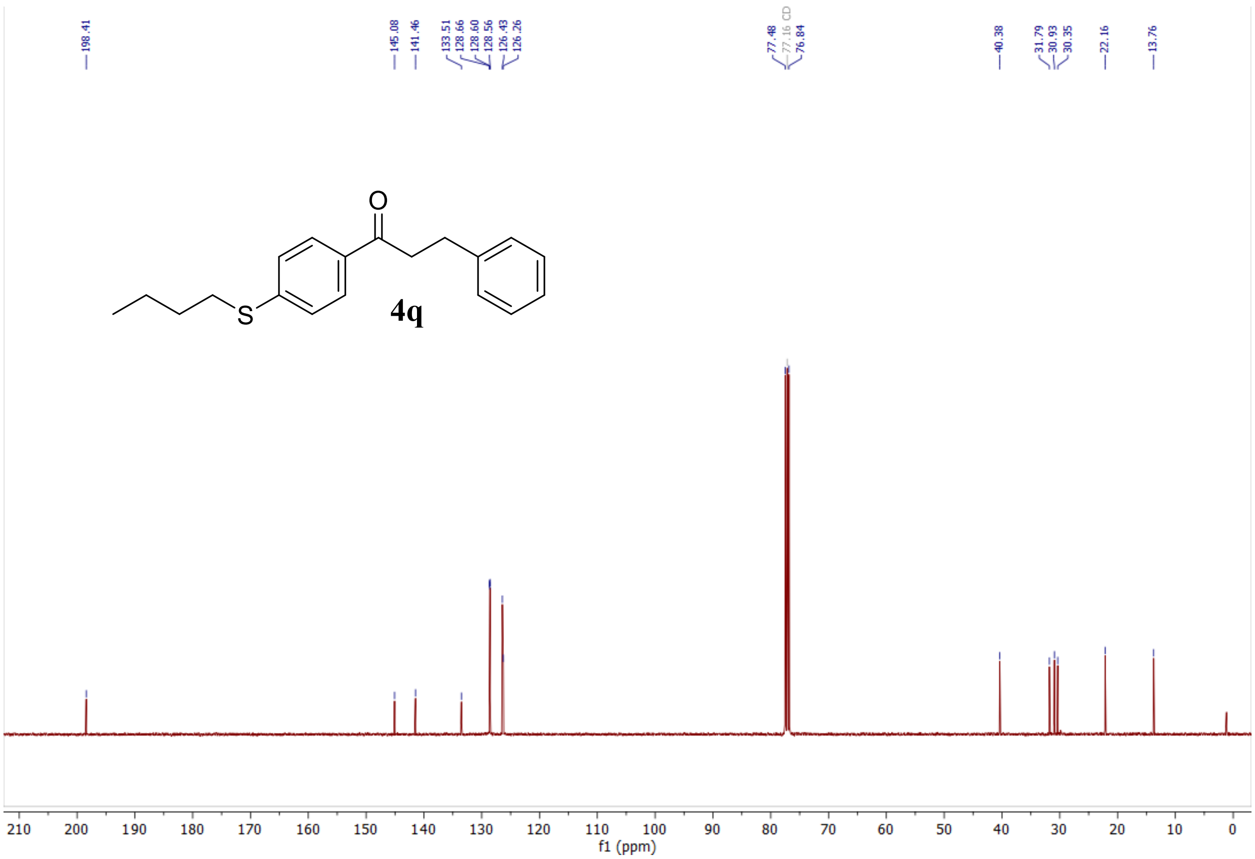

Synthesis of 1-(9*H*-fluoren-2-yl)-3-phenylpropan-1-one 4r

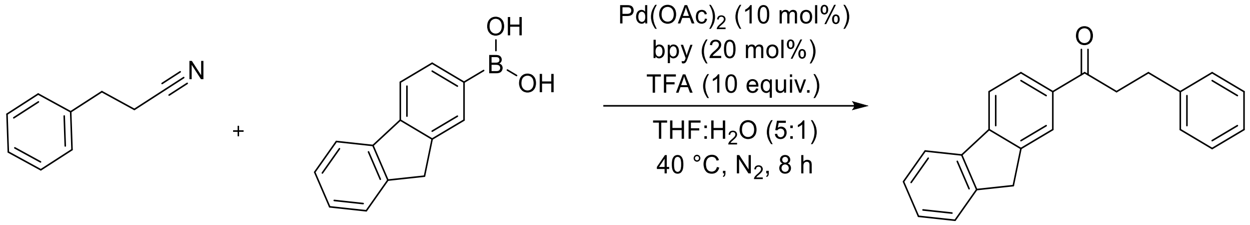

2c

4r
65%

1-(9*H*-fluoren-2-yl)-3-phenylpropan-1-one **4r** was synthesized according to **GP-I**. Purification by silica gel column chromatography (1:20 EtOAc:Hex eluent) yielded the product **4r** as a gummy, clear white solid (77.6 mg, 65% yield). Analytical TLC, 1:4 EtOAc:Hex eluent, $R_f = 0.55$.

^{1}H NMR (400 MHz, $CDCl_3$): δ = 8.15 (dd, *J* = 1.7, 0.8 Hz, 1H), 8.03 – 8.00 (m, 1H), 7.87 – 7.81 (m, 2H), 7.59 (dt, *J* = 7.1, 1.1 Hz, 1H), 7.45 – 7.37 (m, 2H), 7.35 – 7.27 (m, 4H), 7.25 – 7.20 (m, 1H), 3.95 (s, 2H), 3.40 – 3.32 (m, 2H), 3.15 – 3.07 (m, 2H) ppm. **^{13}C NMR** (101 MHz, $CDCl_3$): δ = 199.20, 146.54, 144.64, 143.47, 141.58, 140.64, 135.46, 128.67, 128.60, 128.16, 127.55, 127.21, 126.26, 125.40, 124.88, 121.01, 119.84, 40.75, 37.04, 30.48 ppm. **HRMS**: calcd. for $C_{22}H_{19}O^+$ [M+H$^+$] 299.1430; found 299.1429.

^{1}H NMR of 4r

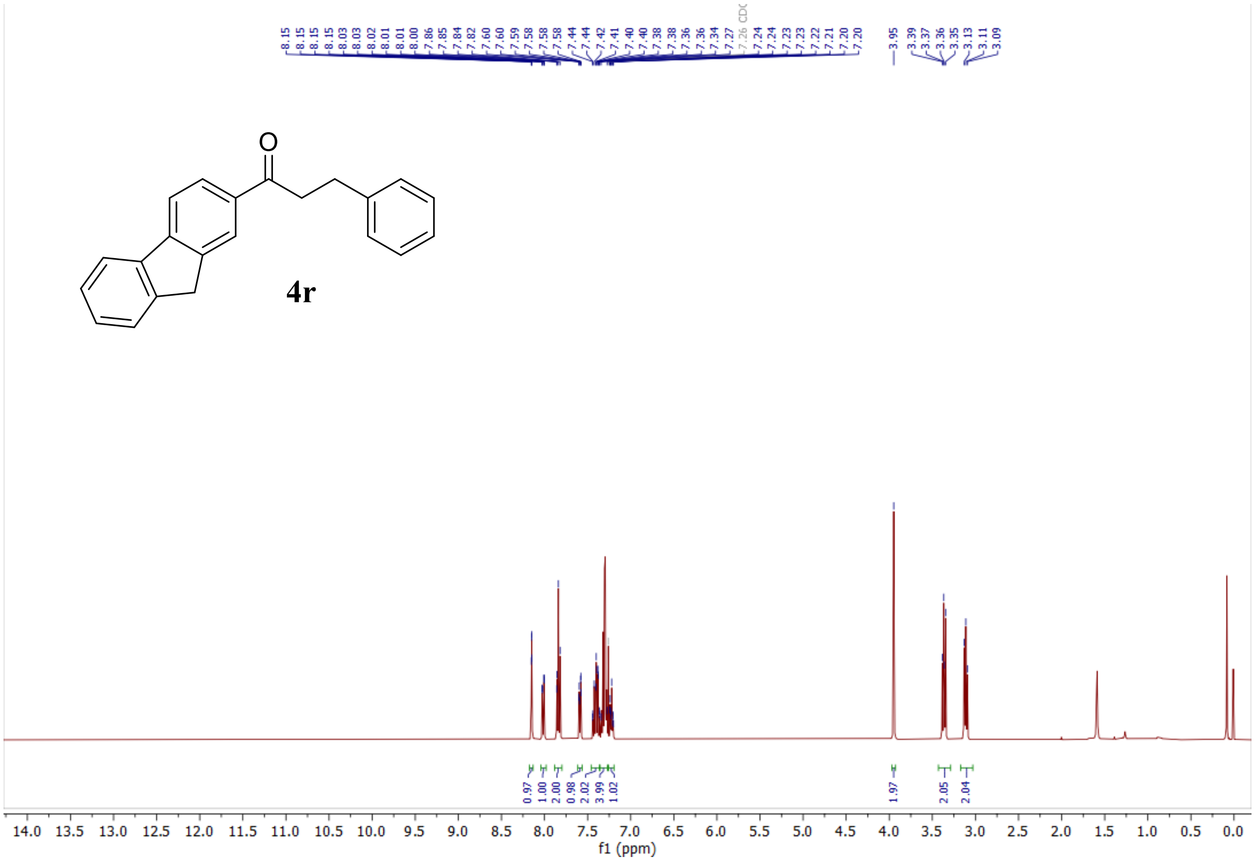

4r

^{13}C NMR of 4r

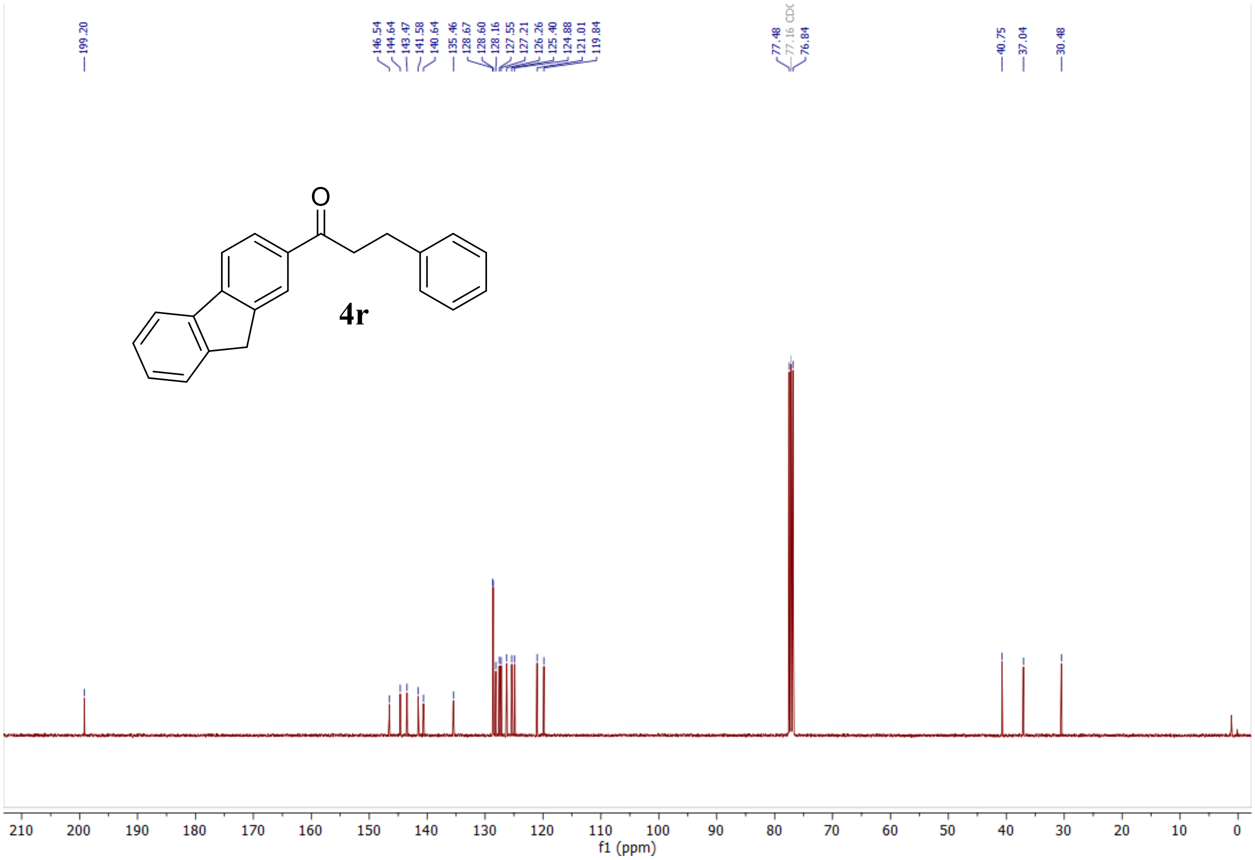

Supplementary Fig. 7: Exploration of the Reactivity of Alkyl and Vinyl Boronic Acids

After successfully synthesizing a scope of diverse small molecules using various aryl boronic acids, we sought to explore whether this reaction would also be compatible with alkyl or vinyl boronic acids. Unfortunately, despite leaving the reactions at high temperature (80 °C) and for extended reaction time, model alkyl and vinyl substrates did not show any conversion to product; unreacted 3-phenylpropionitrile **2c** starting material remained intact.

Attempted Carbometallation Reaction Using Butylboronic Acid

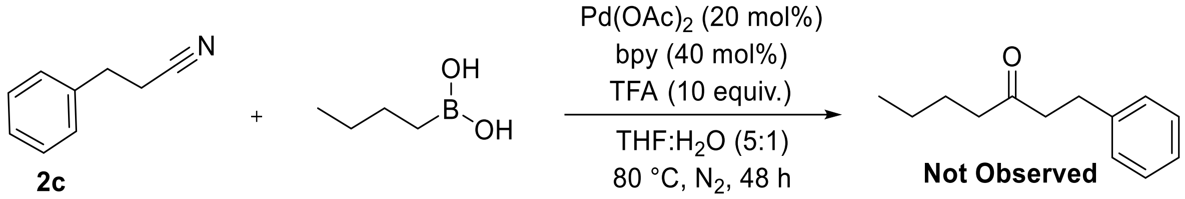

The above reaction was attempted using butylboronic acid as a substrate with conditions according to **GP-III**. After the 48 hour reaction period, starting material **2c** remained fully unconverted with no product formation, as monitored by TLC.

Attempted Carbometallation Reaction Using 1-Pentenylboronic Acid

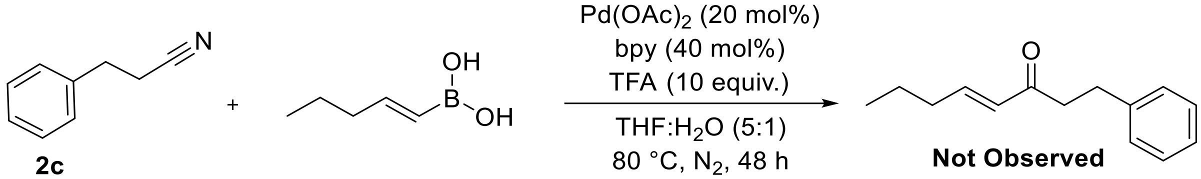

The above reaction was attempted using 1-pentenylboronic acid as a substrate with conditions according to **GP-III**. After the 48 hour reaction period, starting material **2c** remained fully unconverted with no product formation, as monitored by TLC.

Supplementary Fig. 8: Synthesis of Unnatural Nitrile Amino Acids

Fmoc-protected asparagine and glutamine were first converted to nitrile analogues through the isohypsic dehydration reaction prior to further modification with diverse boronic acids.

General Procedure for Isohypsic Dehydration of Fmoc-Asn and Fmoc-Gln (GP-IV):

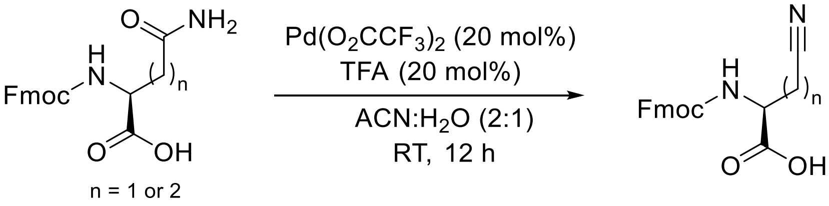

5 **6'**

To a solution of Fmoc-Gln-OH or Fmoc-Asn-OH **5a-5b** (7.6 mmol, 1 equiv.) in ACN:H_2O (2:1) (200 mL:100 mL), Pd(CF_3CO_2)$_2$ (505 mg, 1.5 mmol, 20 mol%) and TFA (116 µL, 1.5 mmol, 20 mol%) were added. The reaction mixture was stirred at room temperature for 12 hours and monitored by TLC, DCM:MeOH (20:1) eluent. Once the reaction was complete, the solvent was evaporated, and the crude product was washed with water and extracted with DCM (3 x 100 mL). The organic layer was dried and concentrated to obtain the crude product. The crude product was purified by flash column chromatography (1:20 MeOH:DCM eluent).

Synthesis of Fmoc-Asn(Nitrile)-CO_2H 6a′

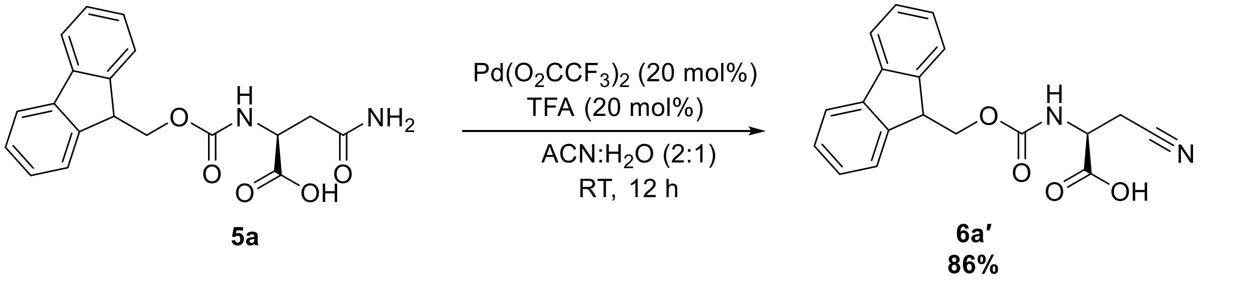

Fmoc-Asn(Nitrile)-CO_2H **6a′** was synthesized according to **GP-IV**, furnishing **6a′** as a pale-yellow semi-liquid (2.20 g, 86% yield). Analytical TLC, 1:20 MeOH:DCM eluent, R_f = 0.50.

^{1}H NMR (400 MHz, DMSO): δ = 8.08 (d, *J* = 8.4 Hz, 1H), 7.89 (d, *J* = 7.5 Hz, 2H), 7.72 (d, *J* = 7.5 Hz, 2H), 7.42 (t, *J* = 7.5 Hz, 2H), 7.33 (t, *J* = 7.4 Hz, 2H), 4.40 – 4.31 (m, 3H), 4.25 (t, *J* = 7.1 Hz, 1H), 2.99 (dd, *J* = 17.0, 5.1 Hz, 1H), 2.88 (dd, *J* = 16.9, 9.3 Hz, 1H) ppm. **^{13}C NMR** (101 MHz, DMSO): δ = 170.88, 155.88, 143.76, 140.76, 127.72, 127.16, 125.26, 120.17, 118.25, 65.96, 50.20, 46.60, 21.09 ppm. **HRMS**: calcd. for $C_{19}H_{18}N_2NaO_5^+$ [M + H_2O + Na^+] 377.1108; found 377.1105.

^{1}H NMR of 6a′

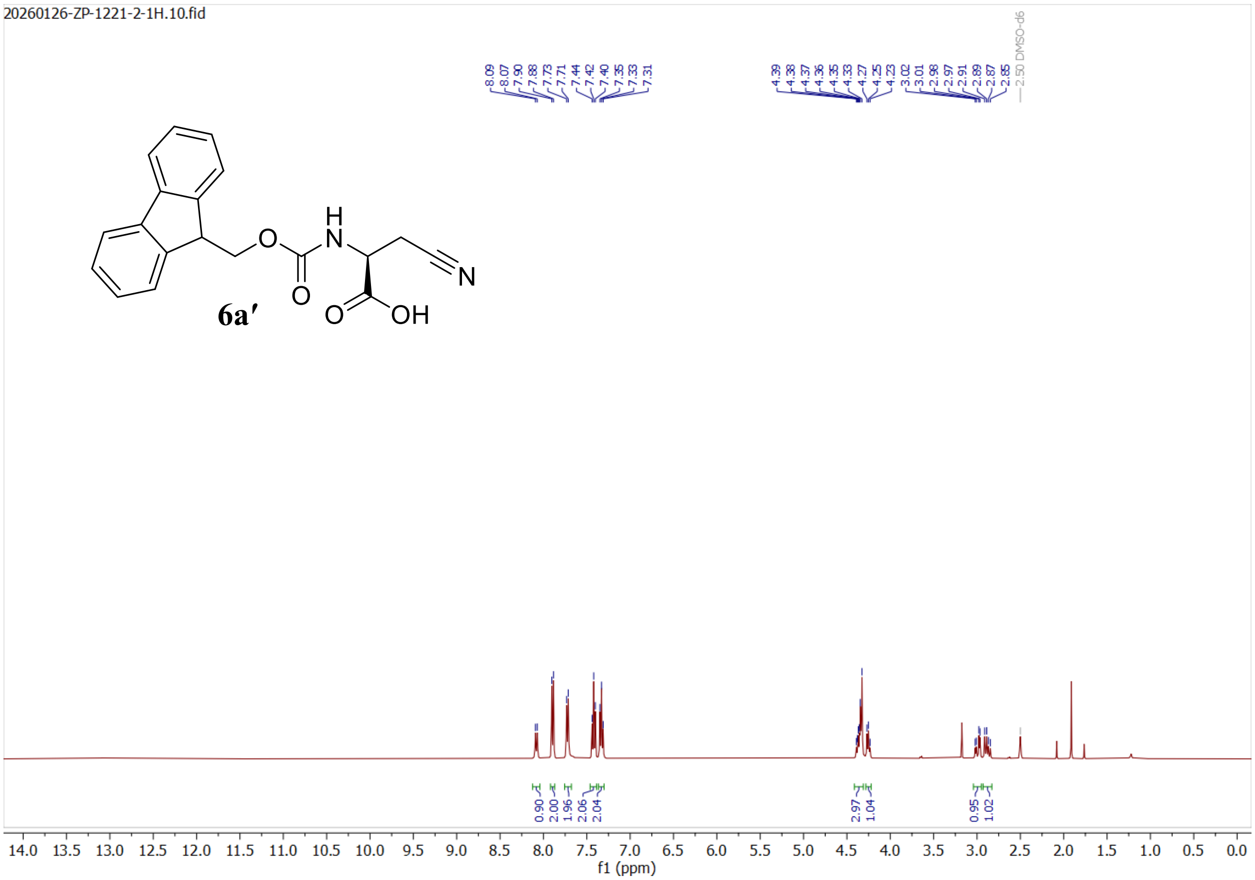

^{13}C NMR of 6a′

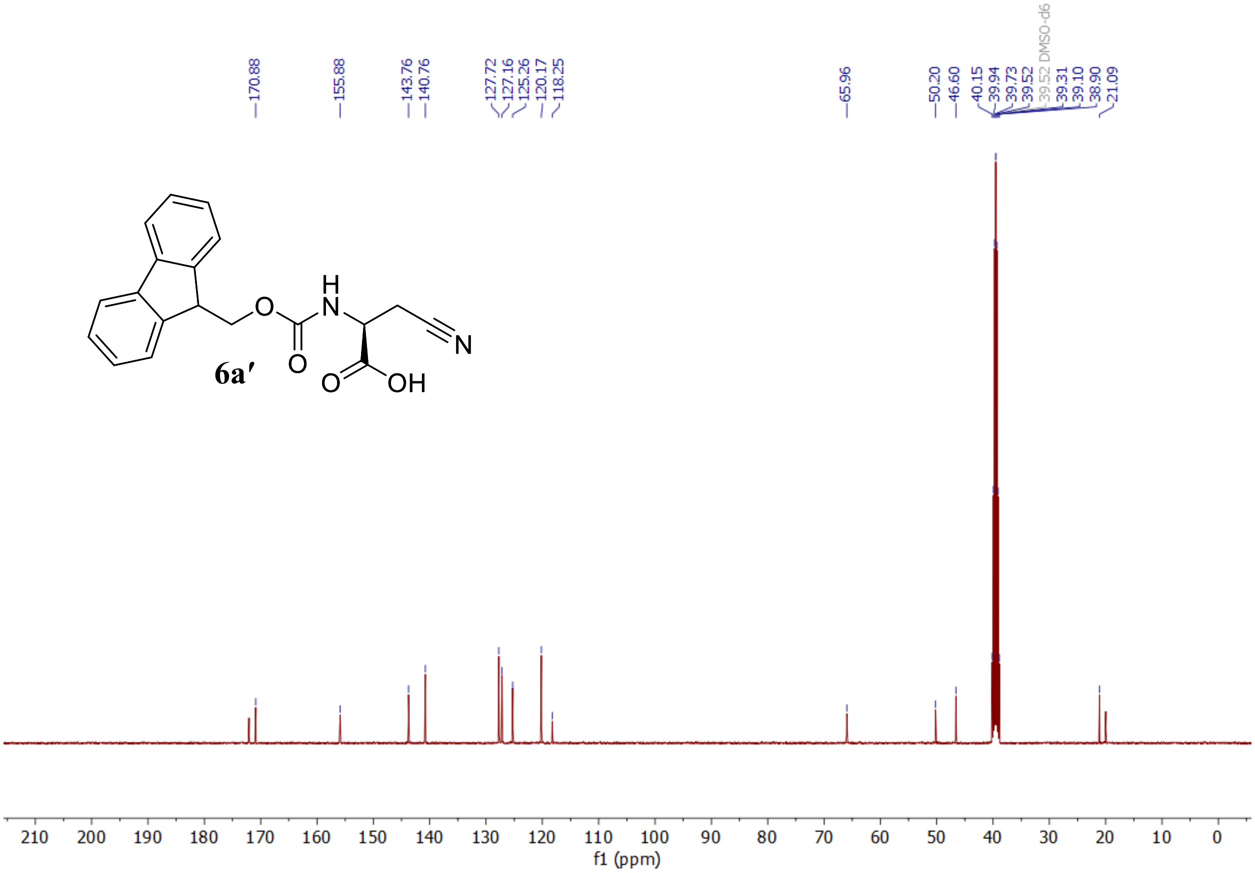

Synthesis of Fmoc-Gln(Nitrile)-CO2H 6b′

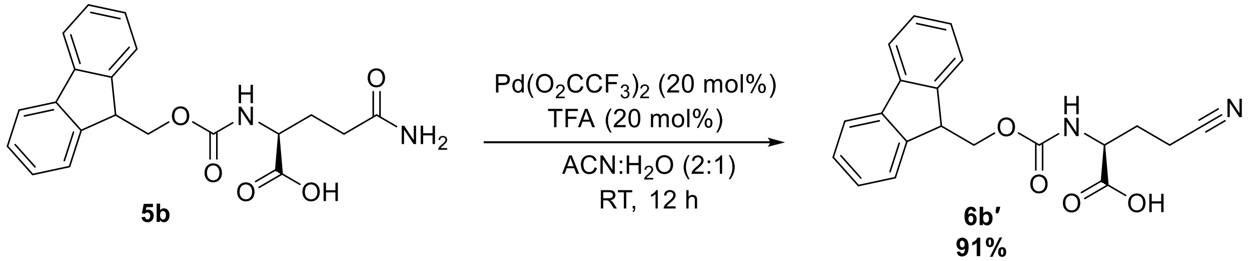

Fmoc-Gln(Nitrile)-CO2H **6b′** was synthesized according to **GP-IV**, furnishing **6b'** as a pale-yellow semi-liquid (2.42 g, 91% yield). Analytical TLC, 1:20 MeOH:DCM eluent, R_f = 0.50.

^{1}H NMR (400 MHz, DMSO): δ = 7.89 (d, J = 7.2 Hz, 2H), 7.71 (d, J = 7.4 Hz, 2H), 7.42 (t, J = 9.3 Hz, 2H), 7.33 (t, J = 8.7 Hz, 2H), 4.36-4.23 (m, 2H), 3.97 (d, J = 3.5 Hz, 1H), 2.98 (dd, J = 17.0, 5.8 Hz, 1H), 2.84 (dd, J = 17.5, 6.3 Hz, 1H) ppm. **^{1}H NMR** (400 MHz, DMSO): δ = 7.89 (d, *J* = 7.5 Hz, 2H), 7.71 (t, *J* = 8.8 Hz, 3H), 7.42 (t, *J* = 7.4 Hz, 2H), 7.33 (t, *J* = 7.4 Hz, 2H), 4.33 (d, *J* = 7.0 Hz, 2H), 4.24 (t, *J* = 6.9 Hz, 1H), 4.07 – 3.96 (m, 1H), 2.54 (t, *J* = 7.3 Hz, 2H), 2.12 – 1.83 (m, 2H) ppm. **^{13}C NMR** (101 MHz, DMSO): δ = 172.78, 156.19, 143.81, 143.78, 140.76, 127.67,

127.10, 125.27, 125.24, 120.16, 120.14, 119.92, 65.67, 52.72, 46.68, 26.61, 13.70 ppm. **HRMS**: calcd. for $C_{20}H_{20}N_2NaO_5^+$ [M + H_2O + Na^+] 391.1264; found 391.1269.

^{1}H NMR of 6b′

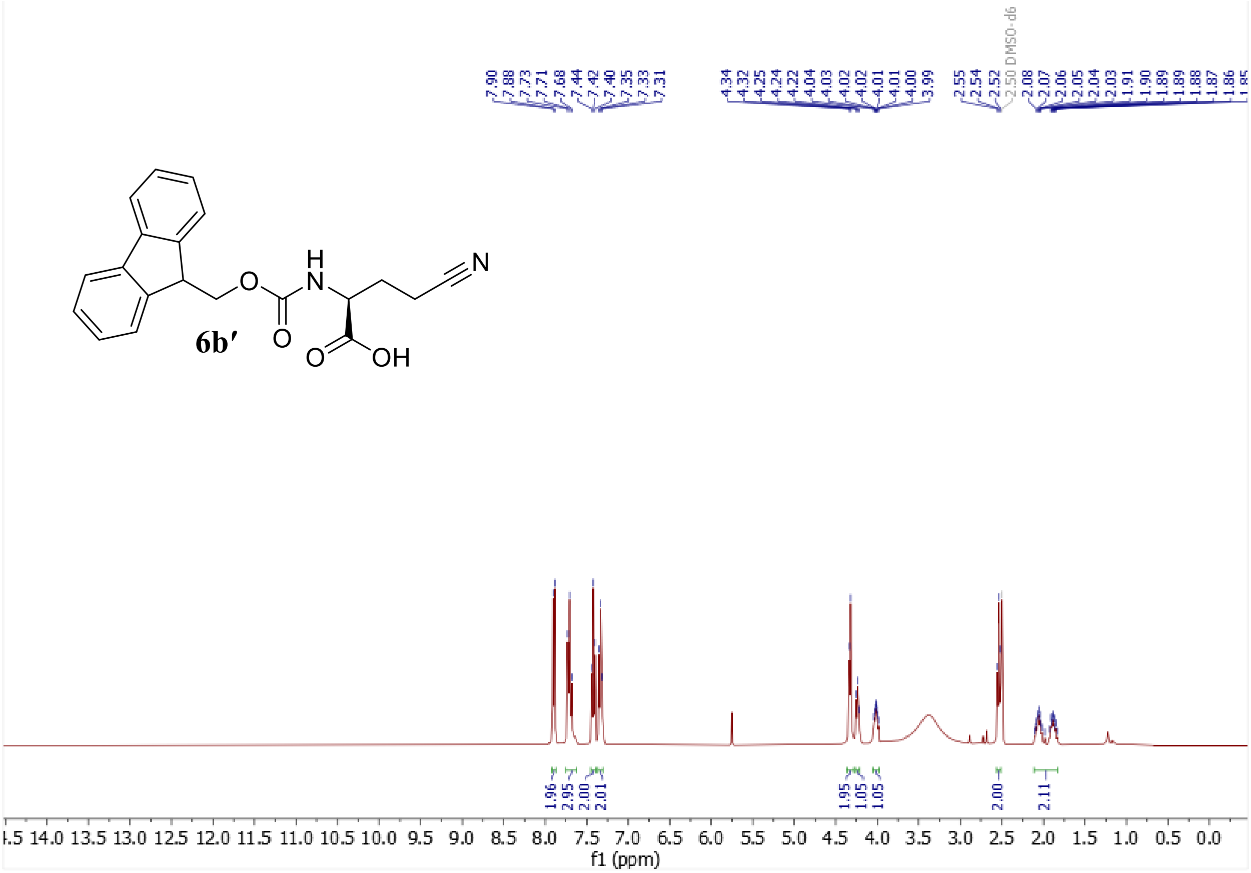

^{13}C NMR of 6b′

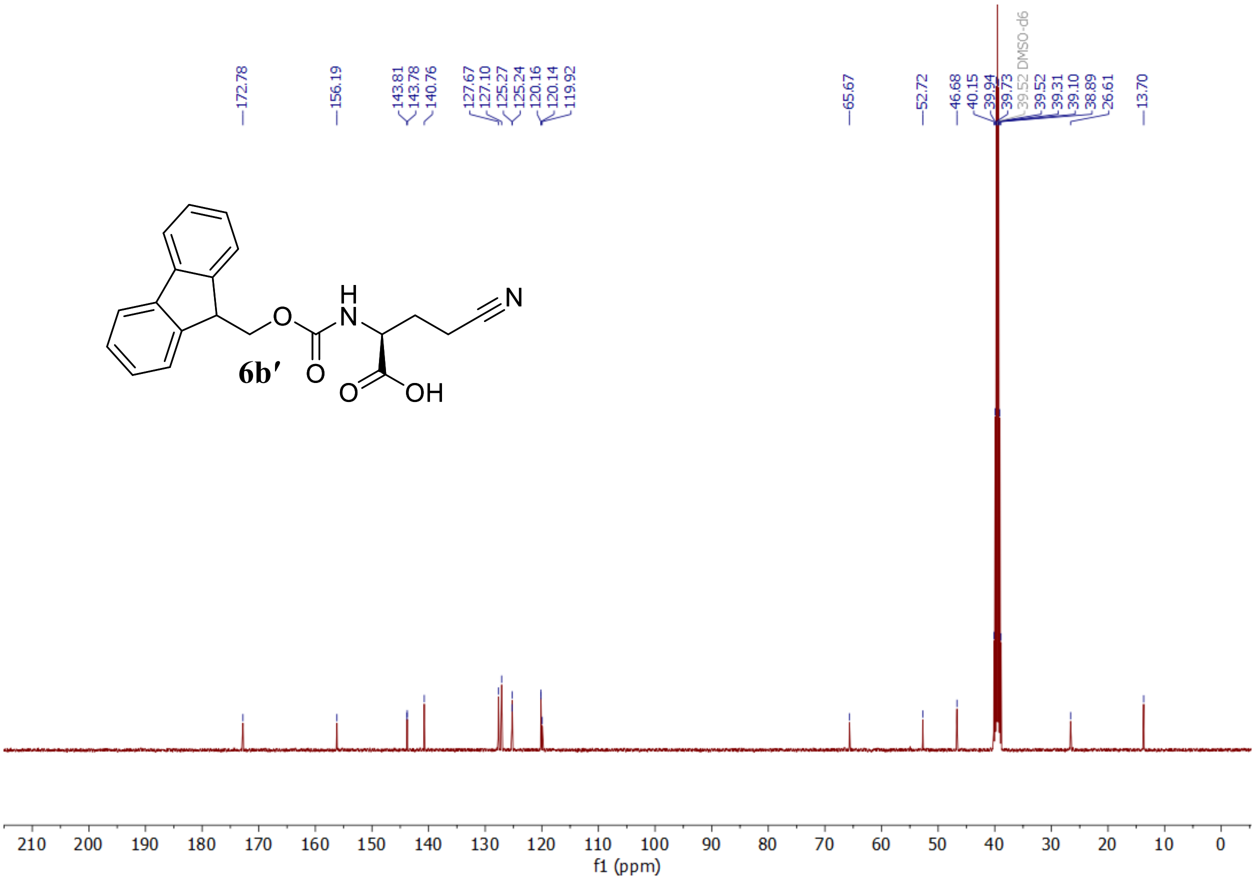

General Procedure for Amino Acid Esterification (GP-V):

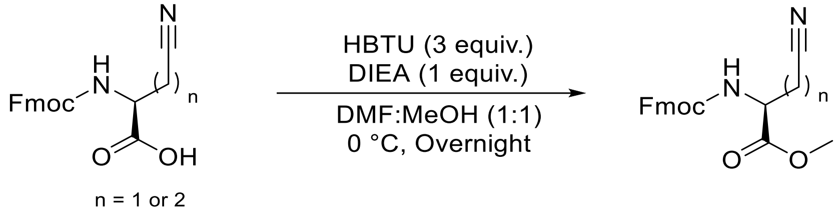

To a solution of Fmoc-Asn(Nitrile)-OH **6a′** or Fmoc-Gln(Nitrile)-OH **6b′** (7.6 mmol, 1 equiv.) in DMF (30 mL), HBTU (8.64 g, 22.8 mmol, 3 equiv.) and DIEA (1.32 mL, 7.6 mmol, 1 equiv.) were added. The reaction mixture was stirred at 0 °C for 30 minutes, then MeOH (30 mL) was added. The reaction progress was monitored by TLC, EtOAc:Hex (1:3) eluent. Upon completion, the solvent was evaporated. The crude product was washed with water and extracted with DCM (3 x 100 mL). The organic layer was dried and concentrated to yield the crude product. This crude product was purified by flash column chromatography EtOAc:Hex (1:3) eluent.

Synthesis of Fmoc-Asn(Nitrile)-CO_2CH_3 6a

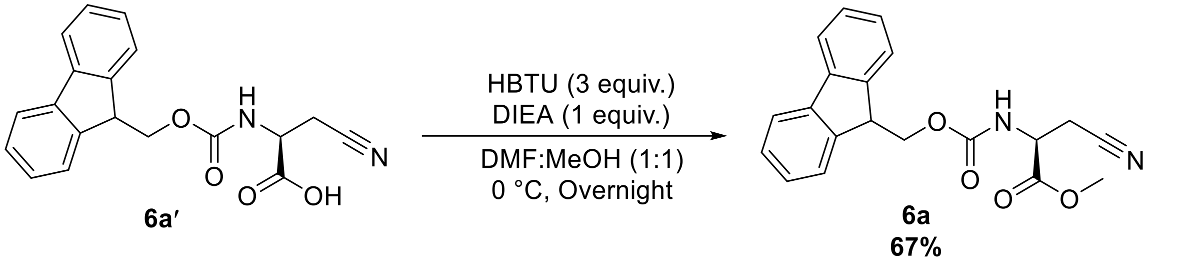

Fmoc-Asn(Nitrile)-CO_2CH_3 **6a** was synthesized according to **GP-V**, furnishing **6a** as a pale yellow solid (1.78 g, 67% yield). Analytical TLC, 3:7 EtOAc:Hex eluent, $R_f = 0.50$.

^{1}H NMR (400 MHz, DMSO): δ = 7.89 (d, J = 7.5 Hz, 2H), 7.71 (d, J = 7.5 Hz, 2H), 7.42 (t, J = 7.5 Hz, 2H), 7.34 (t, J = 7.4 Hz, 2H), 4.49 (dd, J = 9.3, 5.0 Hz, 1H), 4.37 (d, J = 7.0 Hz, 2H), 4.26 (t, J = 6.8 Hz, 1H), 3.66 (s, 3H), 3.05 – 2.87 (m, 2H) ppm. **^{13}C NMR** (101 MHz, DMSO): δ = 169.85, 155.72, 143.68, 140.75, 127.67, 127.09, 125.16, 120.14, 117.91, 65.91, 52.53, 49.96, 46.56, 19.83 ppm. **HRMS**: calcd. for $C_{20}H_{18}N_2NaO_4^+$ [M + Na$^+$] 373.1159; found 373.1158.

^{1}H NMR of 6a

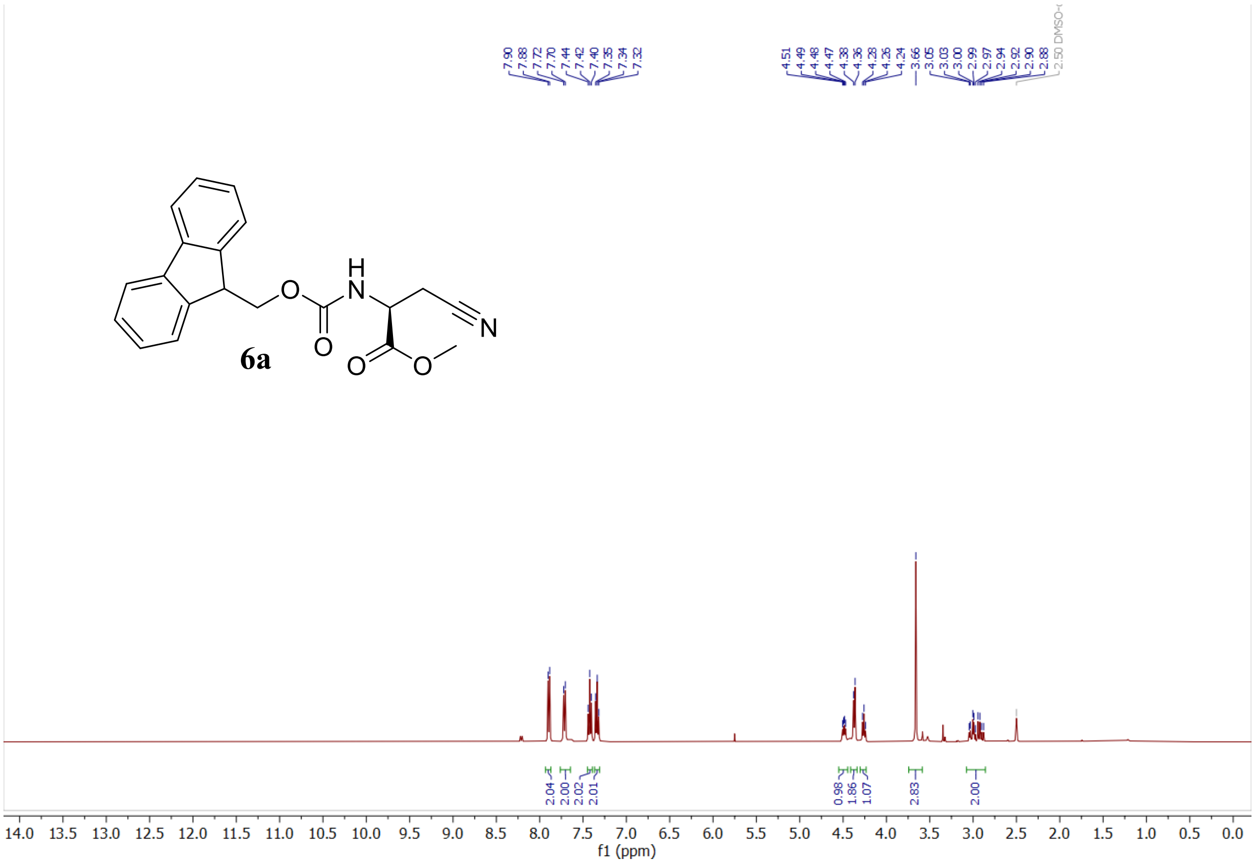

^{13}C NMR of 6a

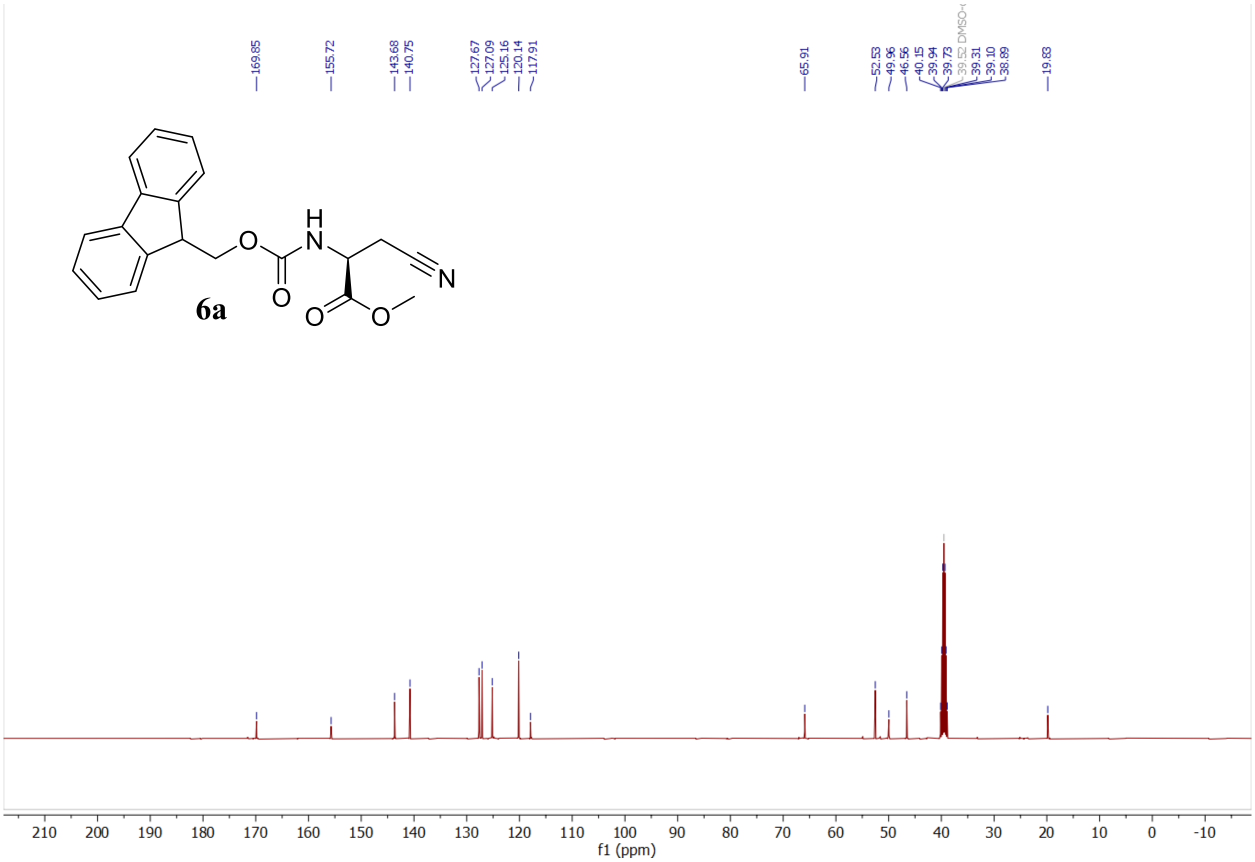

Synthesis of Fmoc-Gln(Nitrile)-CO_2CH_3 6b

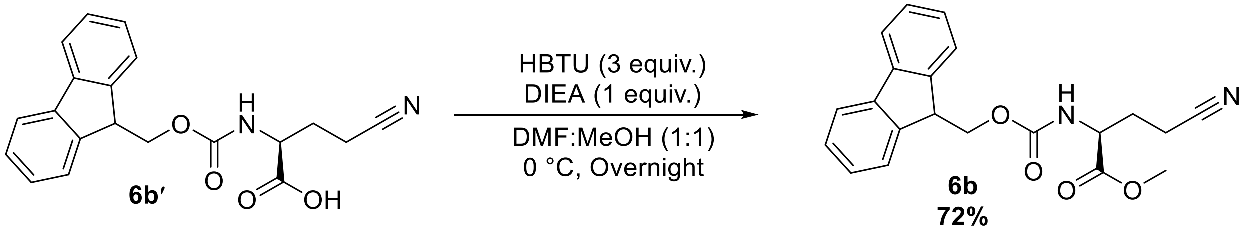

Fmoc-Gln(Nitrile)-$COOCH_3$ **6b** was synthesized according to **GP-V**, furnishing **6b** as a pale yellow solid (1.99 g, 72% yield). Analytical TLC, 3:7 EtOAc:Hex eluent, R_f = 0.50.

^{1}H NMR (400 MHz, DMSO): δ = 7.90 (d, *J* = 7.5 Hz, 2H), 7.84 (d, *J* = 8.0 Hz, 1H), 7.73 – 7.69 (m, 2H), 7.42 (t, *J* = 7.5 Hz, 2H), 7.34 (t, *J* = 7.4 Hz, 2H), 4.38 – 4.32 (m, 2H), 4.24 (t, *J* = 6.8 Hz, 1H), 4.15 – 4.08 (m, 1H), 3.65 (s, 3H), 2.55 (t, *J* = 7.3 Hz, 2H), 2.09 – 2.00 (m, 1H), 1.96 – 1.85 (m, 1H) ppm. **^{13}C NMR** (101 MHz, DMSO): δ = 171.77, 156.11, 143.77, 143.74, 140.78, 127.68, 127.10, 125.20, 120.17, 119.84, 65.67, 52.69, 52.20, 46.67, 26.52, 13.52 ppm. **HRMS**: calcd. for $C_{21}H_{20}N_2NaO_4^+$ [M + H_2O + Na^+] 387.1315; found 387.1314.

^{1}H NMR of 6b

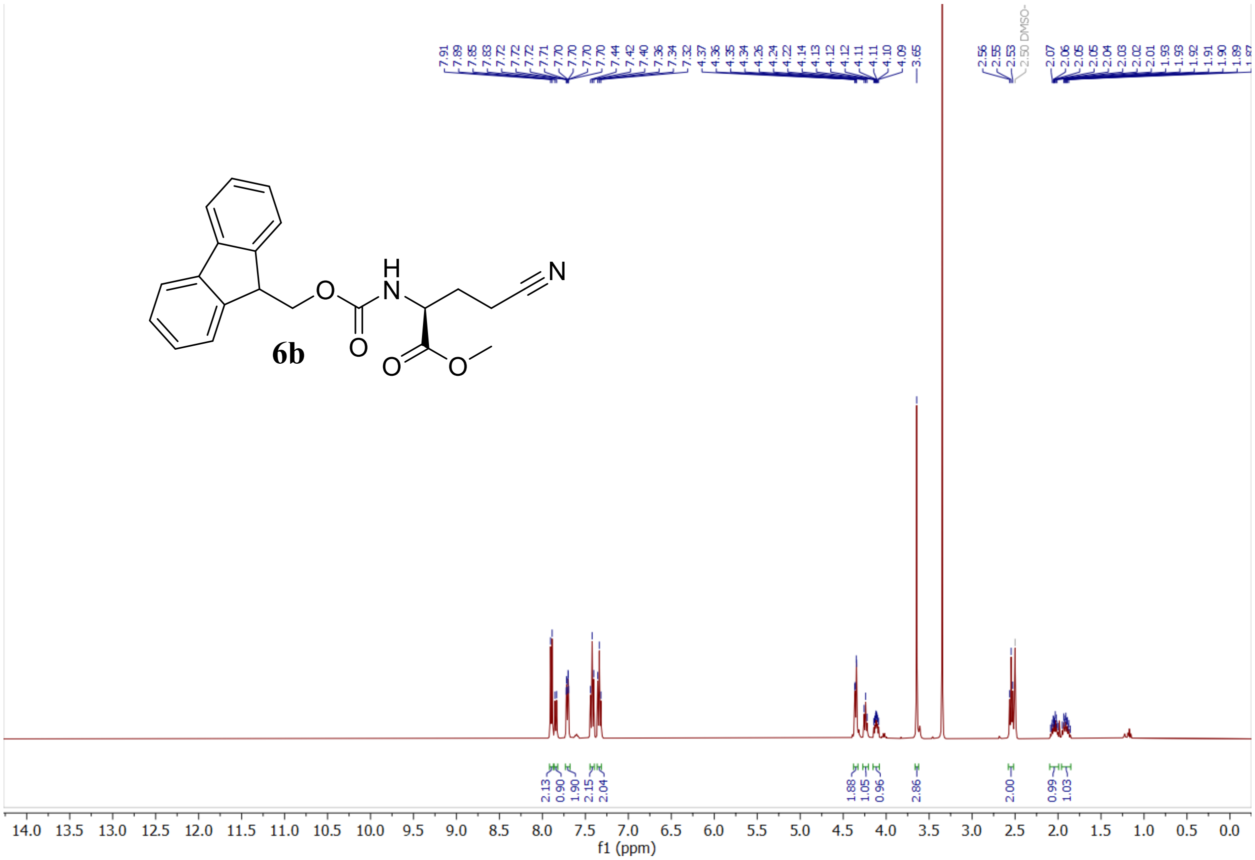

^{13}C NMR of 6b

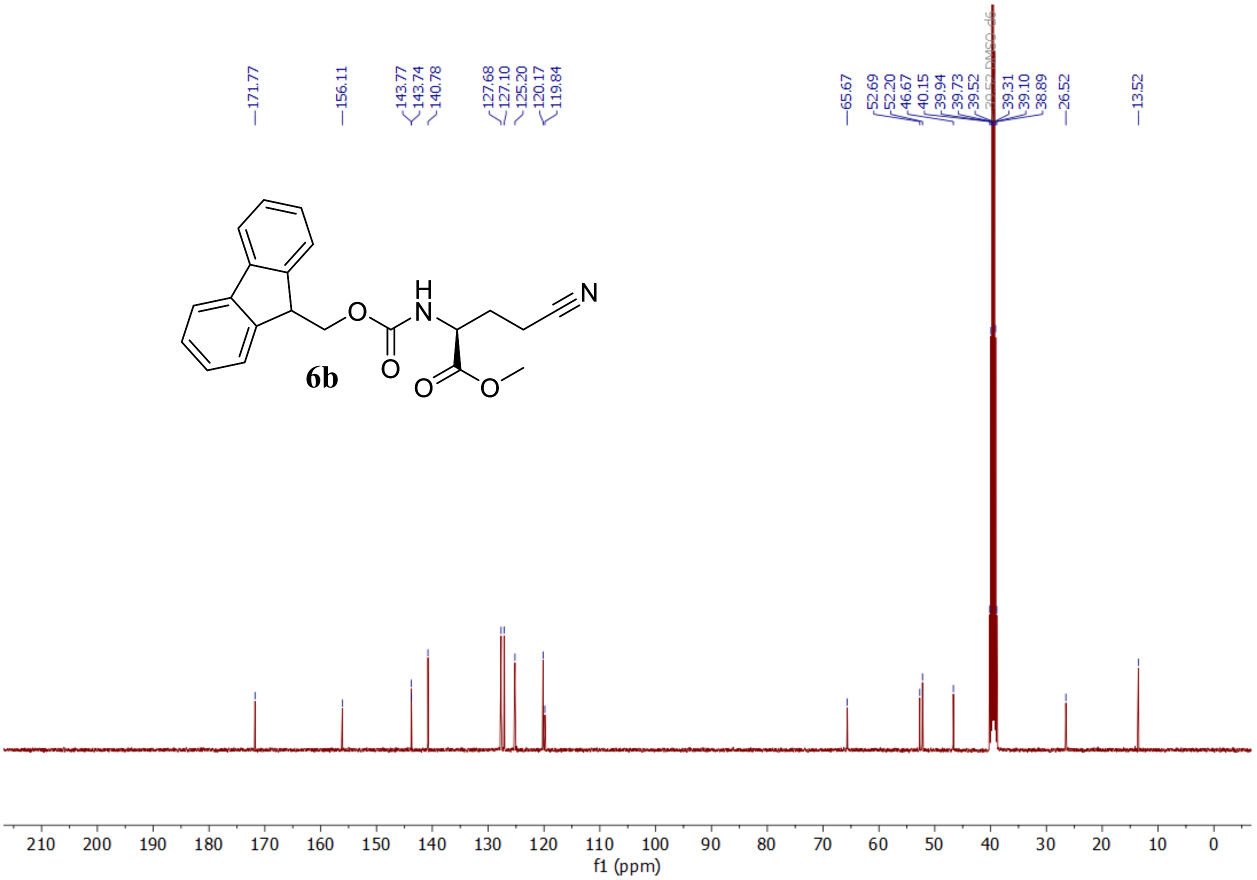

Supplementary Fig. 9: Unnatural Aryl-Ketone Amino Acid Synthesis and Application

Supplementary Fig. 9a: Carbometallation for Generation of Unnatural Aryl-Ketone Amino Acids

General Procedure for Nitrile Boronic Acid Carbometallation Reaction on Amino Acid (GP-VI):

To a 35 mL high-pressure tube was added 3 mL of THF:H_2O (5:1) and TFA (230 µL, 3.0 mmol, 10 equiv.). Next, Fmoc-Asn(Nitrile)-$COOCH_3$ **6a** or Fmoc-Gln(nitrile)-$COOCH_3$ **6b** (0.3 mmol, 1 equiv.), the appropriate boronic acid (1.2 mmol, 4 equiv.), and 2,2'-bipyridyl ligand (9.4 mg, 0.06 mmol, 20 mol%) were added to the solution. The mixture was left to stir until all compounds were dissolved, after which N_2 was bubbled for 5 minutes. Finally, $Pd(OAc)_2$ (6.7 mg, 0.03 mmol, 10 mol%) was transferred to the solution. The high-pressure tube was flushed with N_2, and the reaction was stirred for 12 hours at 80 °C. Upon completion by TLC, the reaction mixture was diluted with EtOAc (15 mL) and transferred to a 60 mL separatory funnel. The organic layer was

washed with brine (3 x 20 mL), dried over Na_2SO_4, and then adsorbed onto silica. Purification by silica gel column chromatography yielded the small molecule ketone products.

The acidic and high temperature conditions of the reaction hydrolyzed the ester of the amino acid back to carboxylic acid. As the ester hydrolysis largely proceeded slower than the carbometallation reaction, two of the reactions were stopped after 3 hours to furnish the ester product; all other amino acid products were left for 12 hours and contain a C-terminal carboxylic acid.

Asparagine Ketone Unnatural Amino Acids:

Synthesis of Fmoc-Asn(Triphenylamine)-CO_2CH_3 7a

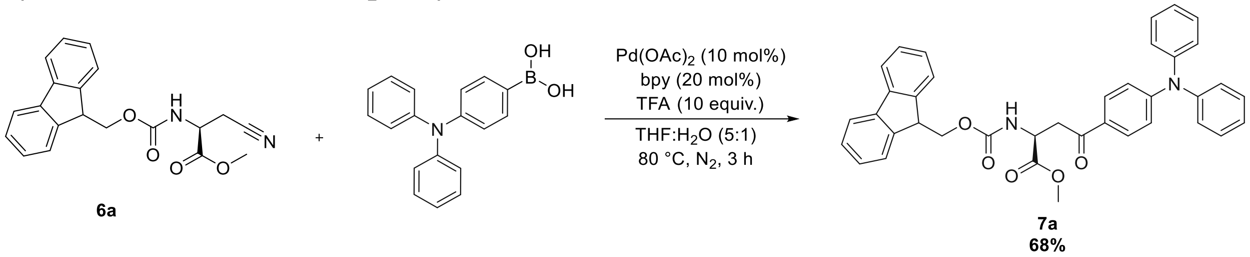

Fmoc-Asn(Triphenylamine)-CO_2CH_3 **7a** was synthesized according to **GP-VI** with a reaction time of 3 hrs. Purification by silica gel column chromatography (1:1 EtOAc:Hex eluent) yielded the product **7a** as a sticky yellow-white solid (122 mg, 68% yield). Analytical TLC, 1:19 MeOH:DCM eluent (with 0.1% AcOH), R_f = 0.55.

^{1}H NMR (400 MHz, DMSO): δ = 7.88 (d, *J* = 7.4 Hz, 2H), 7.80 (dd, *J* = 19.3, 8.4 Hz, 3H), 7.68 (dd, *J* = 7.6, 3.9 Hz, 2H), 7.40 (q, *J* = 7.2 Hz, 6H), 7.30 (q, *J* = 8.7, 8.1 Hz, 3H), 7.20 (t, *J* = 6.8 Hz, 2H), 7.14 (d, *J* = 7.2 Hz, 4H), 7.06 – 6.96 (m, 1H), 6.86 (d, *J* = 8.9 Hz, 2H), 4.35 – 4.18 (m, 3H), 3.62 (s, 3H), 3.45 – 3.29 (m, 2H) ppm. **^{13}C NMR** (101 MHz, DMSO): δ = 194.12, 172.25, 155.80, 151.85, 145.81, 143.76, 140.72, 129.96, 129.50, 128.34, 127.63, 127.06, 126.04, 125.11, 123.71, 120.12, 118.50, 65.69, 52.09, 50.08, 49.77, 46.60 ppm. **HRMS**: calcd. for $C_{38}H_{33}N_2O_5^+$ [M+H$^+$] 597.2384; found 597.2386.

^{1}H NMR of 7a

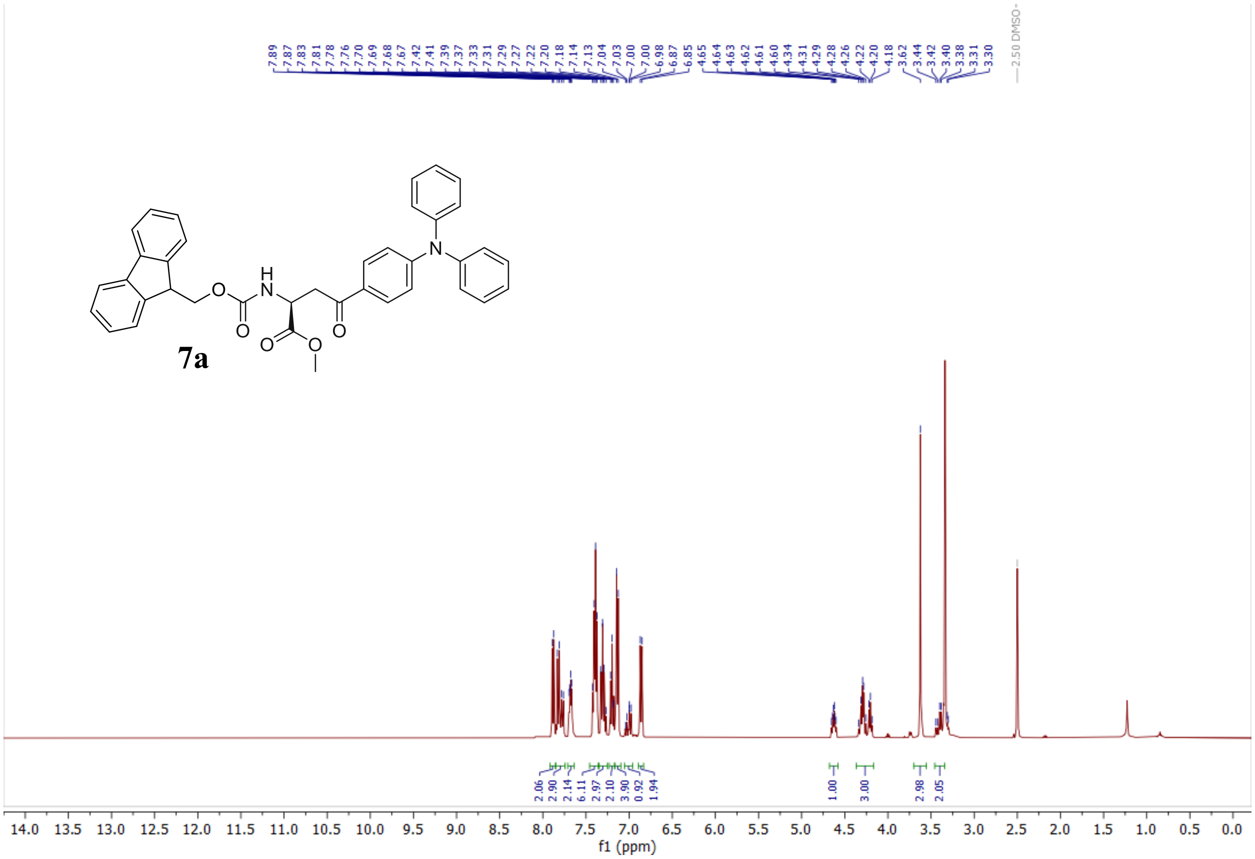

^{13}C NMR of 7a

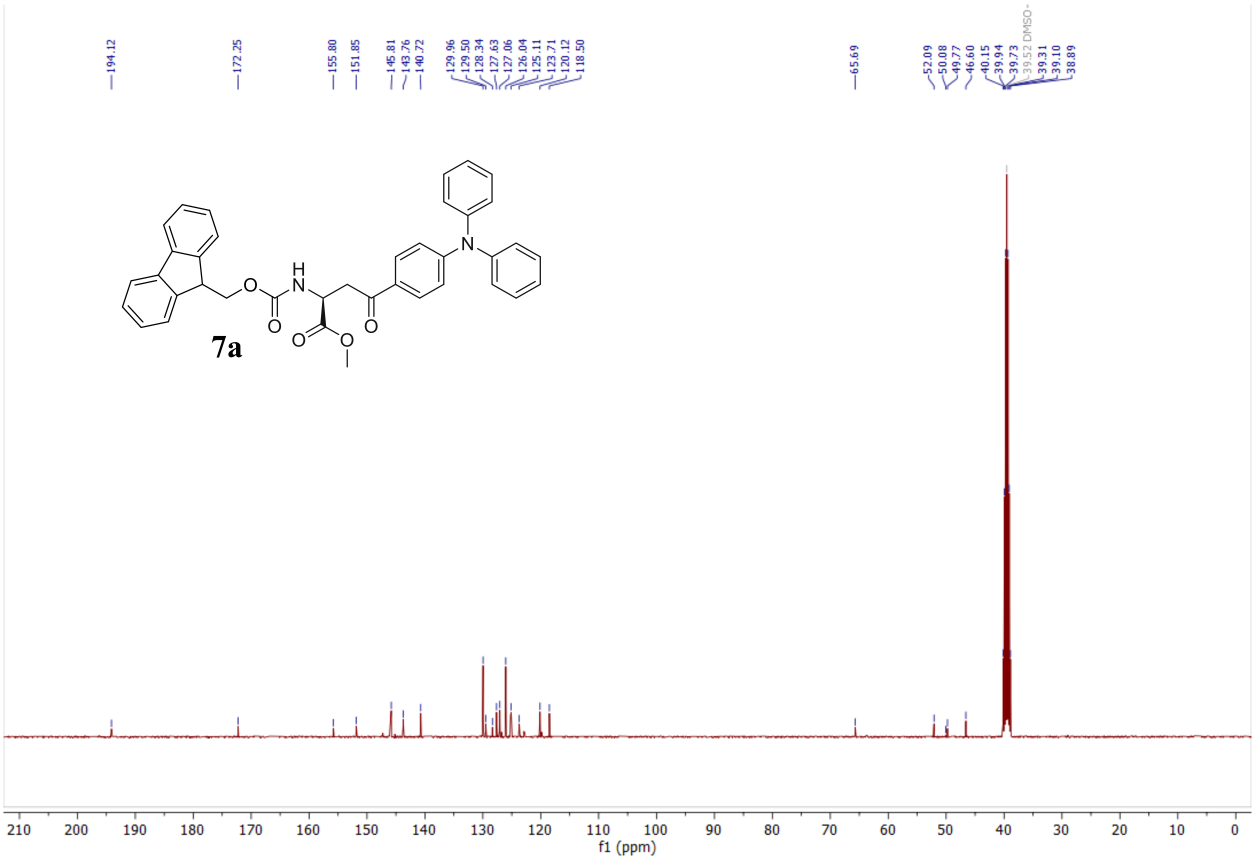

Synthesis of Fmoc-Asn(Triphenylamine)-CO_2H 7b

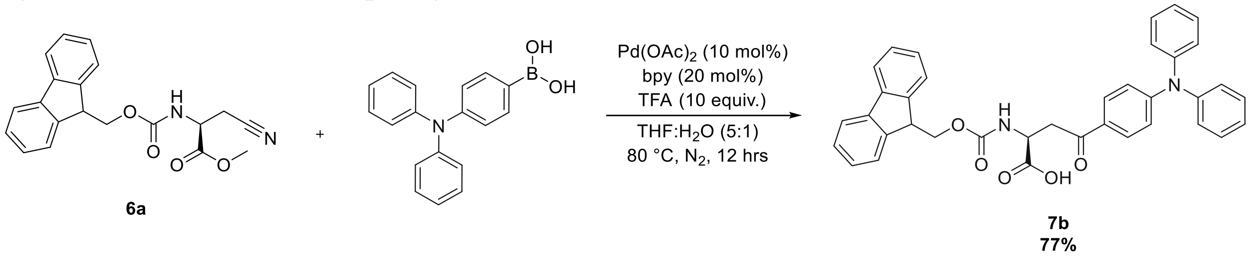

Fmoc-Asn(Triphenylamine)-CO_2H **7b** was synthesized according to **GP-VI** with a reaction time of 12 hrs. Purification by silica gel column chromatography (2:1 EtOAc:Hex eluent) yielded the product **7b** as a granule yellow solid (135 mg, 77% yield). Analytical TLC, 1:19 MeOH:DCM eluent (with 0.1% AcOH), $R_f = 0.45$.

^{1}H NMR (400 MHz, DMSO): δ = 7.88 (d, J = 7.4 Hz, 2H), 7.83 (d, J = 9.0 Hz, 2H), 7.71 – 7.62 (m, 3H), 7.43 – 7.36 (m, 6H), 7.30 (t, J = 7.5 Hz, 2H), 7.19 (t, J = 7.4 Hz, 2H), 7.13 (d, J = 7.5 Hz, 4H), 6.86 (d, J = 9.0 Hz, 2H), 4.62 – 4.52 (m, 1H), 4.32 – 4.16 (m, 3H), 3.42 – 3.27 (m, 2H) ppm. **^{13}C NMR** (101 MHz, DMSO): δ = 194.42, 173.31, 155.86, 151.80, 145.85, 143.81, 140.72, 129.96, 129.50, 128.55, 127.65, 127.09, 126.02, 125.28, 125.08, 120.12, 118.59, 65.70, 52.19, 49.84, 46.63 ppm. **HRMS**: calcd. for $C_{37}H_{31}N_2O_5^+$ [M+H$^+$] 583.2227; found 583.2224.

^{1}H NMR of Fmoc-Asn(Triphenylamine)-CO_2H 7b

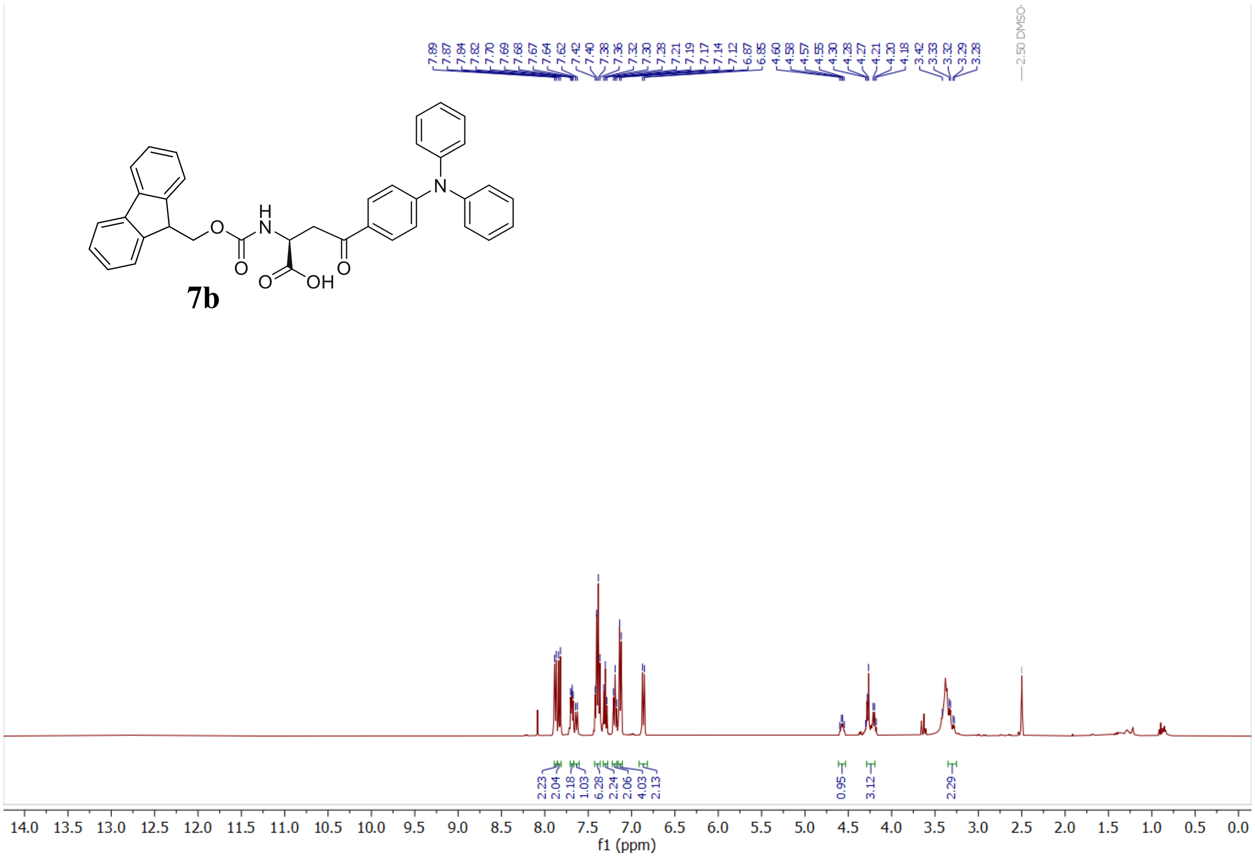

^{13}C NMR of Fmoc-Asn(Triphenylamine)-CO_2H 7b

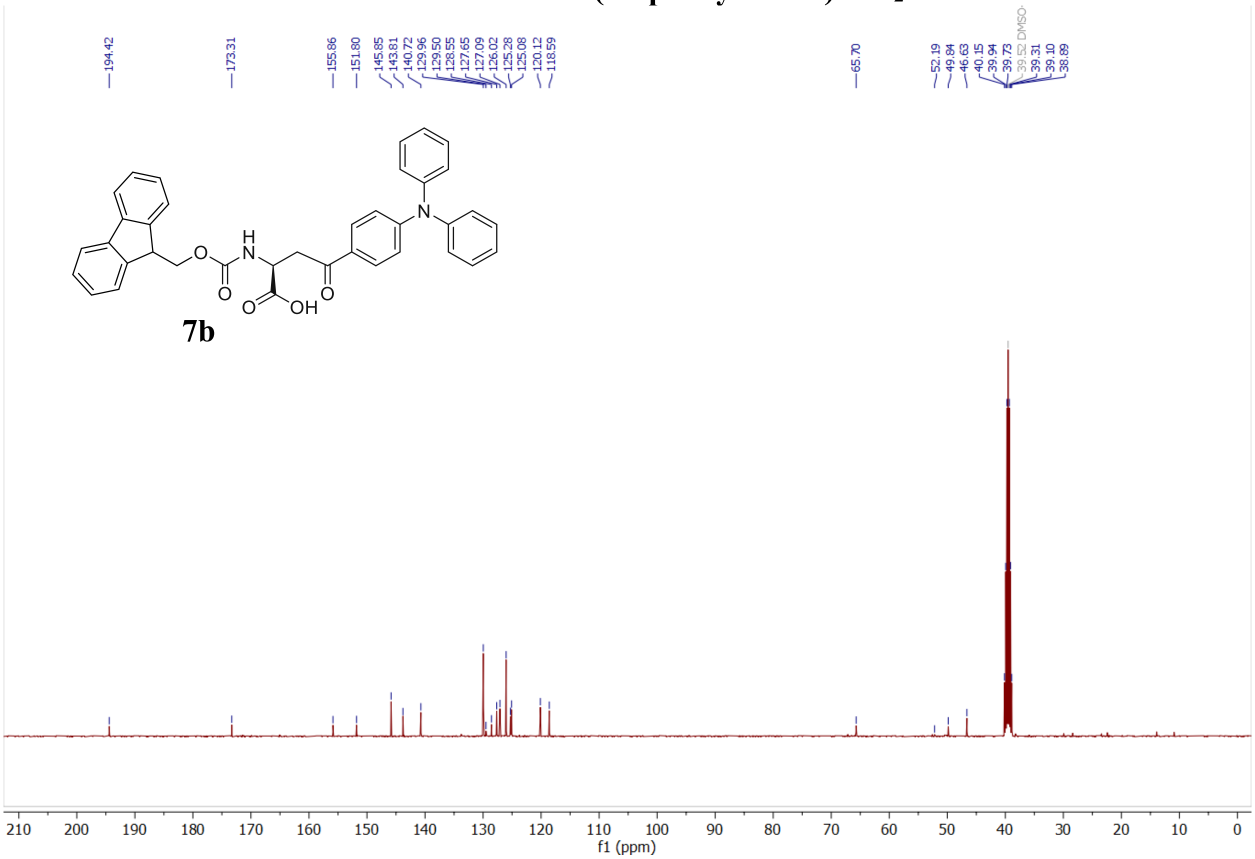

Synthesis of Fmoc-Asn(Indolyl ketone)-CO_2CH_3 7c

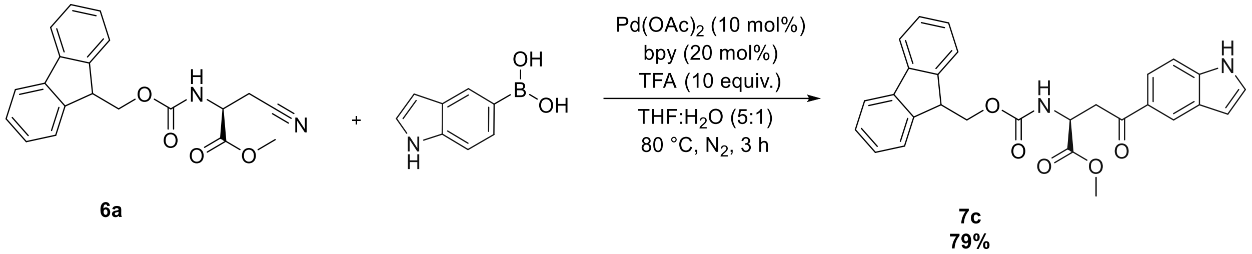

Fmoc-Asn(Indolyl ketone)-CO_2CH_3 **7c** was synthesized according to **GP-VI** with a reaction time of 3 hrs. Purification by silica gel column chromatography (3:7 EtOAc:Hex eluent) yielded the product **7c** as a granule yellow solid (111 mg, 79% yield). Analytical TLC, 1:19 MeOH:DCM eluent (with 0.1% AcOH), R_f = 0.80.

^{1}H NMR (400 MHz, DMSO): δ = 11.54 (s, 1H), 8.30 (s, 1H), 7.88 (d, *J* = 7.4 Hz, 2H), 7.79 (d, *J* = 7.9 Hz, 1H), 7.74 – 7.66 (m, 3H), 7.50 – 7.47 (m, 2H), 7.40 (t, *J* = 7.4 Hz, 2H), 7.30 (t, *J* = 7.5 Hz, 2H), 6.64 – 6.60 (m, 1H), 4.67 (td, *J* = 7.7, 5.1 Hz, 1H), 4.33 – 4.19 (m, 3H), 3.64 (s, 3H), 3.61 – 3.44 (m, 2H) ppm. **^{13}C NMR** (101 MHz, DMSO): δ = 195.76, 172.41, 155.85, 143.78, 140.72, 138.62, 128.05, 127.63, 127.30, 127.15, 127.07, 125.22, 122.37, 120.91, 120.12, 111.44, 103.03,

65.70, 52.09, 49.90, 46.61 ppm. **HRMS**: calcd. for $C_{28}H_{25}N_2O_5^+$ [M+H$^+$] 469.1758; found 469.1763.

^{1}H NMR of 7c

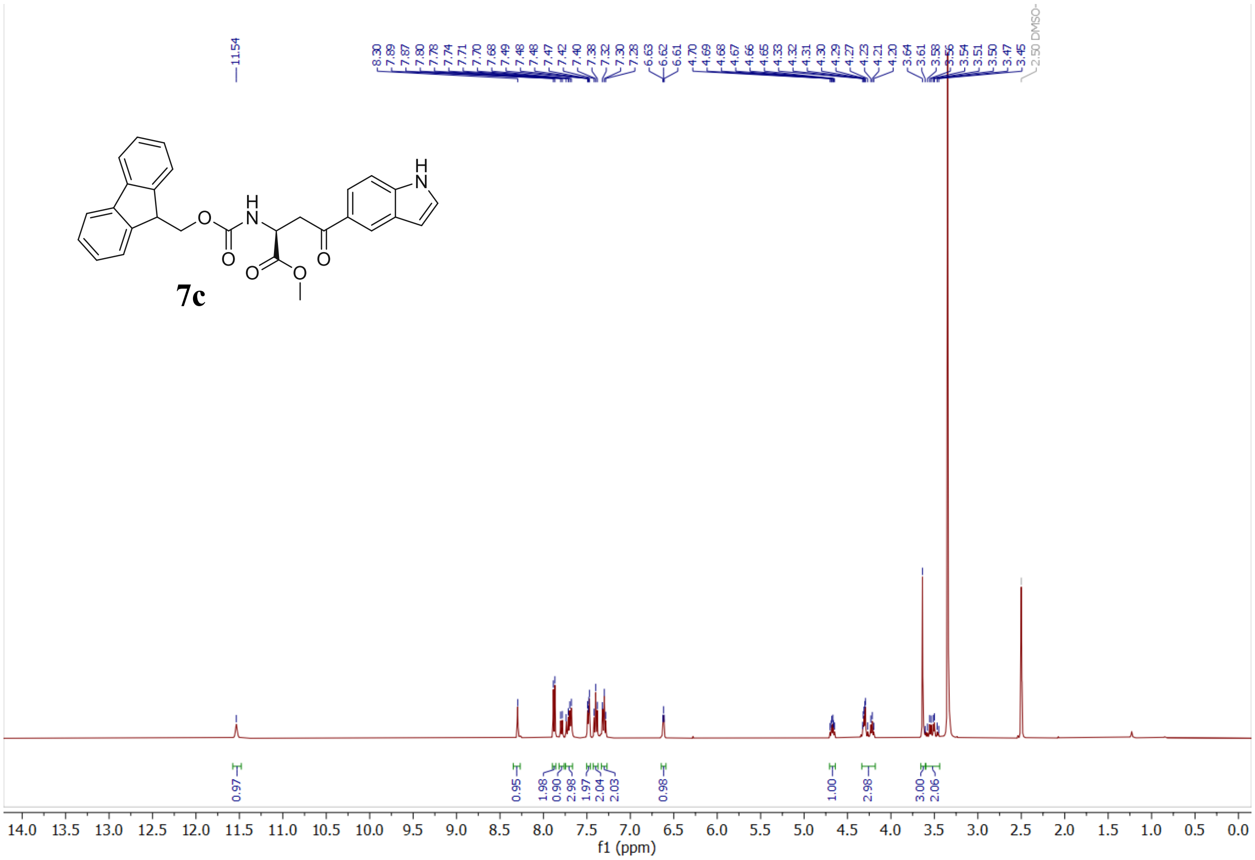

^{13}C NMR of 7c

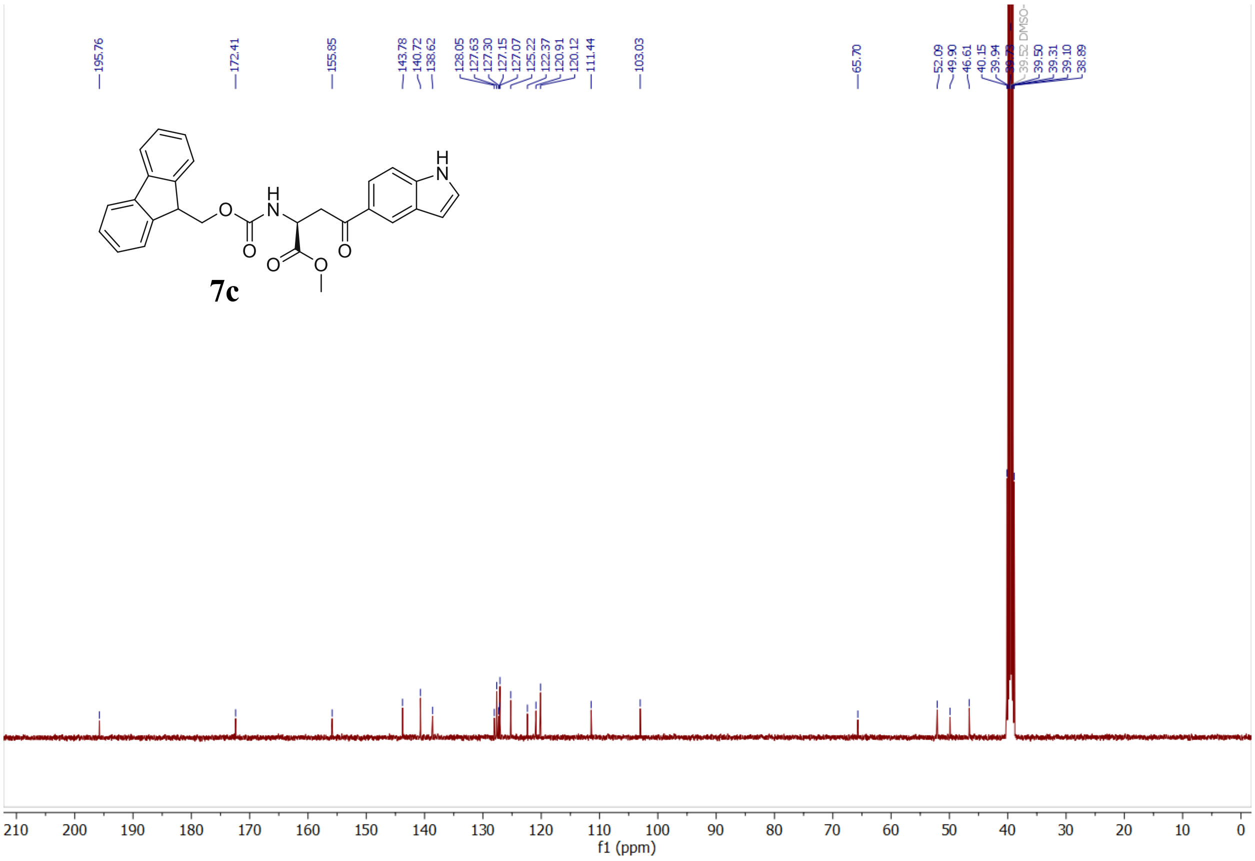

Synthesis of Fmoc-Asn(1,4-Benzodioxane ketone)-CO_2H 7d

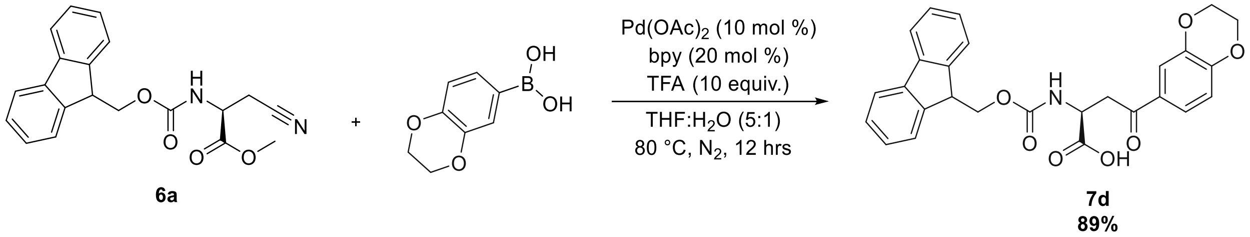

Fmoc-Asn(1,4-Benzodioxane ketone)-CO_2H **7d** was synthesized according to **GP-VI** with a reaction time of 12 hrs. Purification by silica gel column chromatography (3:2 EtOAc:Hex eluent) yielded the product **7d** as a pale white solid (126 mg, 89% yield). Analytical TLC, 1:19 MeOH:DCM eluent (with 0.1% AcOH), R_f = 0.45.

^{1}H NMR (400 MHz, DMSO): δ = 12.70 (s, 1H), 7.88 (d, *J* = 7.5 Hz, 2H), 7.69 (dd, *J* = 7.5, 3.9 Hz, 2H), 7.61 (d, *J* = 8.1 Hz, 1H), 7.49 (dd, *J* = 8.5, 2.2 Hz, 1H), 7.44 (d, *J* = 2.1 Hz, 1H), 7.41 (t, *J* = 7.5 Hz, 2H), 7.30 (td, *J* = 7.5, 1.2 Hz, 2H), 6.98 (d, *J* = 8.5 Hz, 1H), 4.53 (td, *J* = 7.8, 5.1 Hz, 1H), 4.34 – 4.26 (m, 6H), 4.23 – 4.18 (m, 1H), 3.41 – 3.28 (m, 2H) ppm. **^{13}C NMR** (101 MHz, DMSO): δ = 194.90, 173.17, 155.84, 148.04, 143.79, 143.19, 140.70, 129.96, 127.62, 127.06, 125.23, 122.07, 120.11, 117.16, 65.67, 64.56, 63.94, 49.80, 46.60 ppm. **HRMS**: calcd. for $C_{27}H_{24}NO_7^+$ [M+H$^+$] 474.1547; found 474.1549.

^{1}H NMR of Fmoc-Asn(1,4-Benzodioxane ketone)-CO_2H 7d

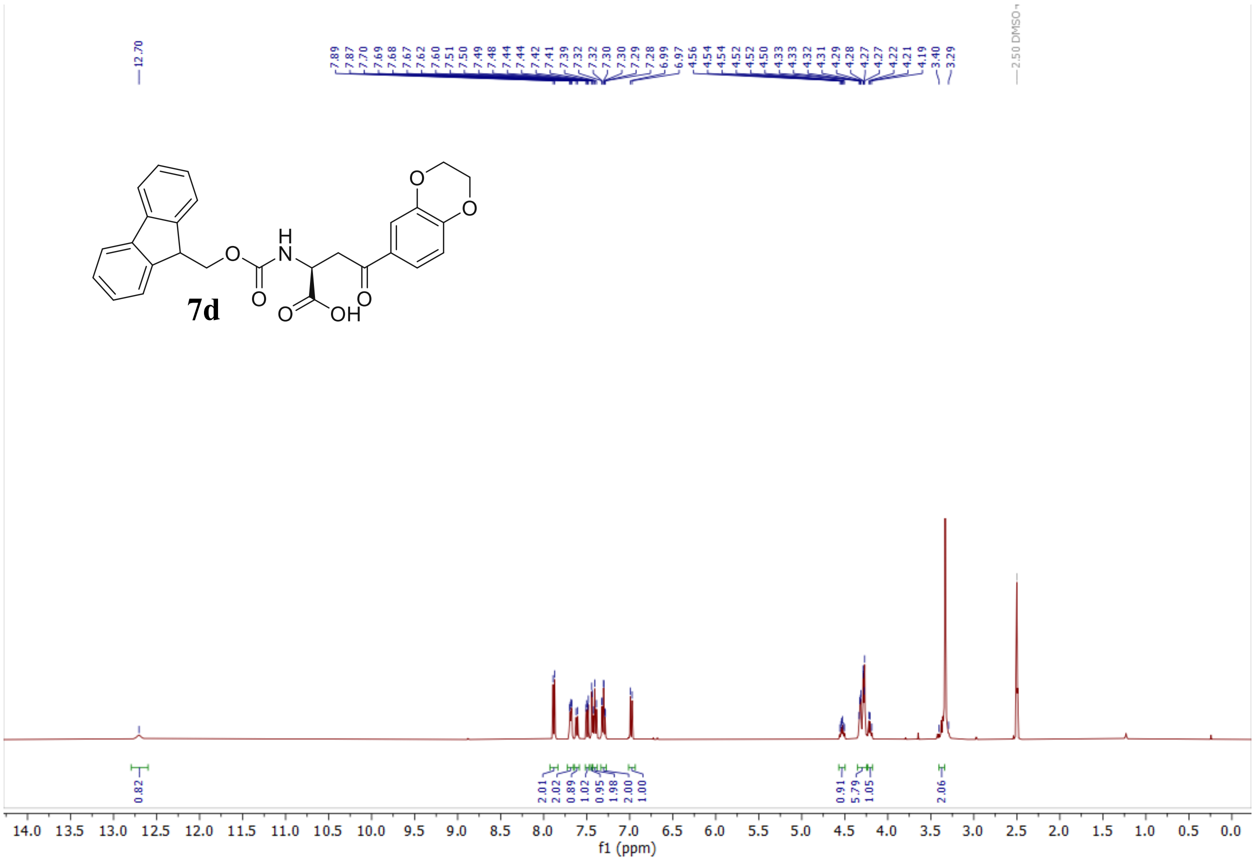

^{13}C NMR of Fmoc-Asn(1,4-Benzodioxane ketone)-CO_2H 7d

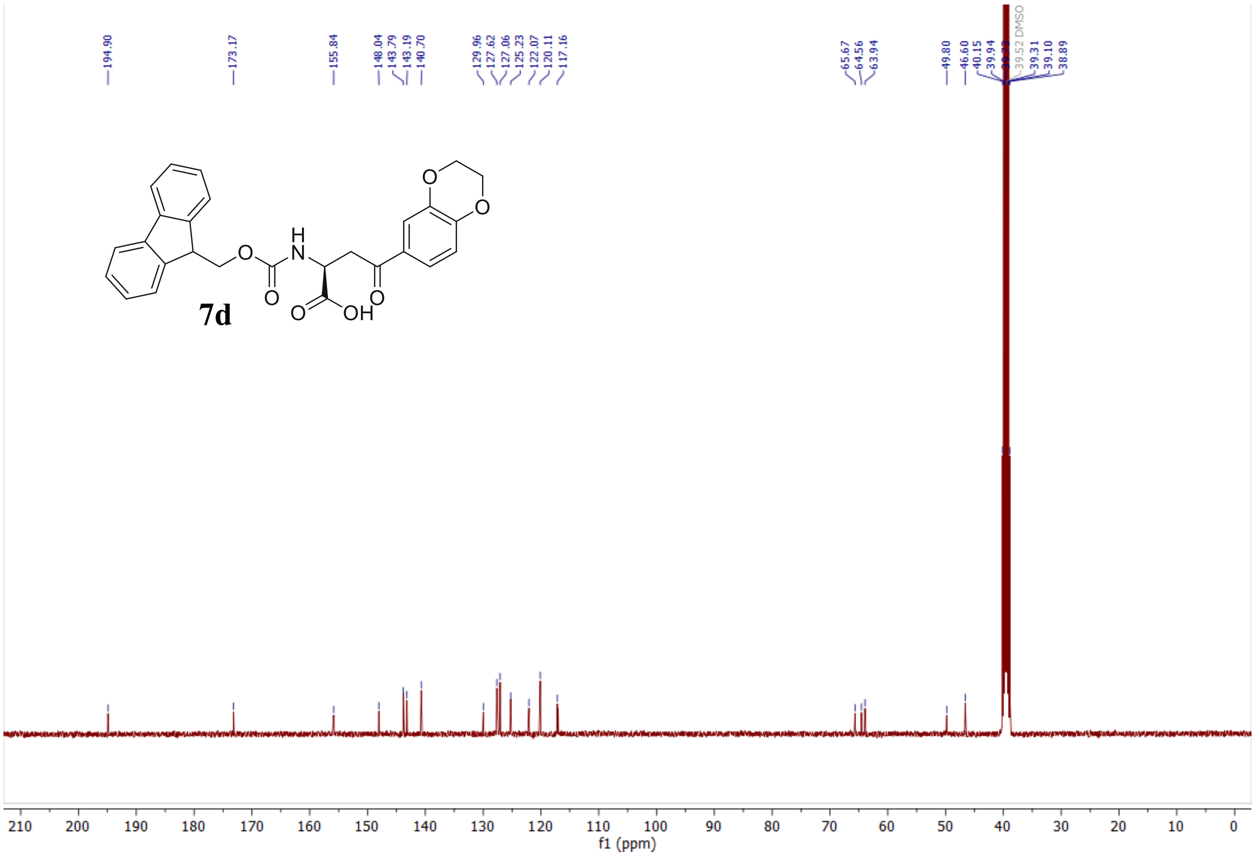

Synthesis of Fmoc-Asn(paramethoxyphenylketone)-CO_2H 7e

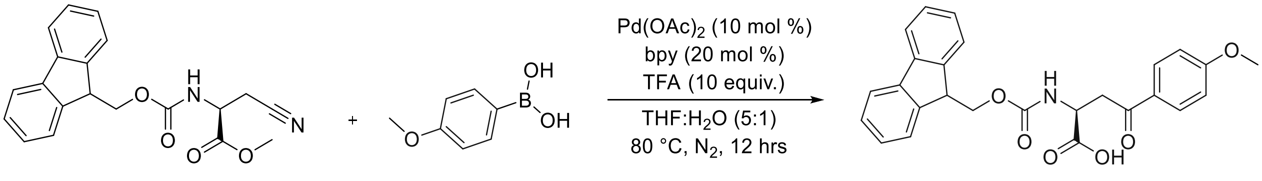

6a

7e
74%

Fmoc-Asn(paramethoxyphenylketone)-CO_2H **7e** was synthesized according to **GP-VI** with a reaction time of 12 hrs. Purification by silica gel column chromatography (4:1 EtOAc:Hex eluent) yielded the product **7e** as a pale white solid (98.8 mg, 74% yield). Analytical TLC, 1:19 MeOH:DCM eluent (with 0.1% AcOH), R_f = 0.30.

^{1}H NMR (400 MHz, DMSO): δ = 7.95 (d, J = 8.9 Hz, 2H), 7.88 (d, J = 7.5 Hz, 2H), 7.69 (dd, J = 7.6, 2.9 Hz, 2H), 7.62 (d, J = 8.1 Hz, 1H), 7.40 (t, J = 7.3 Hz, 2H), 7.30 (t, J = 7.5 Hz, 2H), 7.05 (d, J = 8.9 Hz, 2H), 4.55 (td, J = 7.8, 5.0 Hz, 1H), 4.28 (d, J = 6.4 Hz, 2H), 4.23 – 4.18 (m, 1H), 3.84 (s, 3H), 3.42 – 3.36 (m, 2H) ppm. **^{13}C NMR** (101 MHz, DMSO): δ = 194.91, 173.23, 163.28, 155.83, 143.79, 143.78, 140.69, 130.36, 129.29, 127.61, 127.05, 125.23, 120.09, 113.92, 65.66, 55.56, 49.76, 46.59 ppm. **HRMS**: calcd. for $C_{26}H_{24}NO_6^+$ [M+H$^+$] 446.1598; found 446.1871.

^{1}H NMR of Fmoc-Asn(paramethoxyphenylketone)-CO_2H 7e

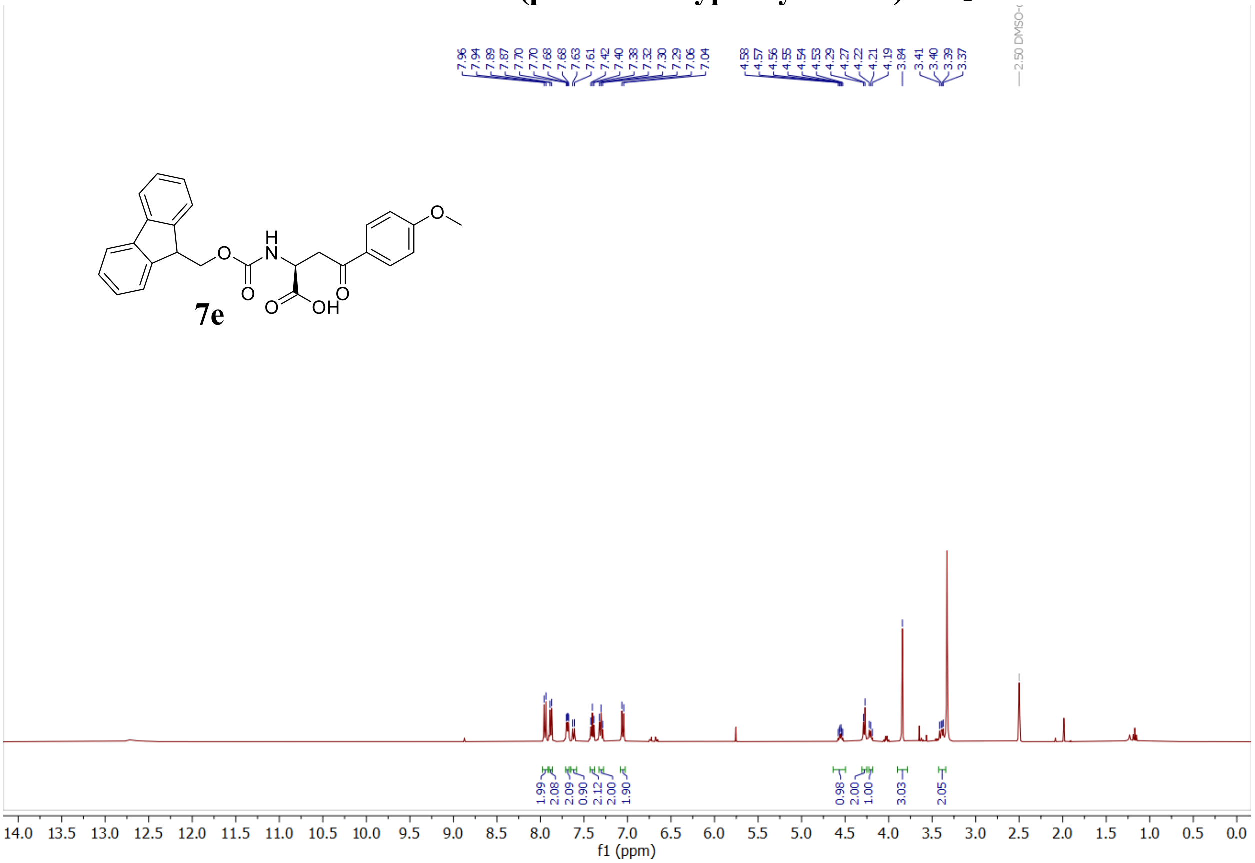

^{13}C NMR of Fmoc-Asn(paramethoxyphenylketone)-CO_2H 7e

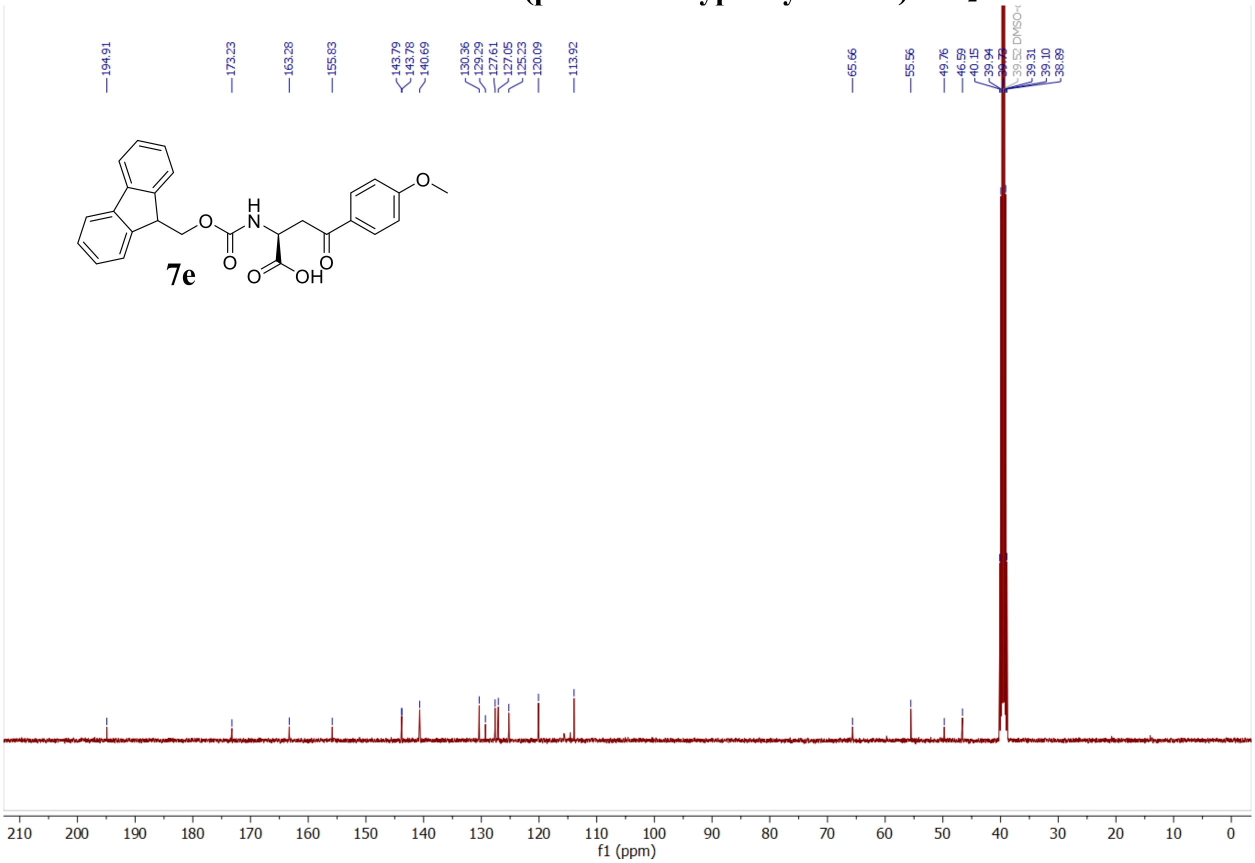

Glutamine Unnatural Ketone Amino Acids:

Synthesis of Fmoc-Gln(Butylthiophenylketone)-CO_2H 7f

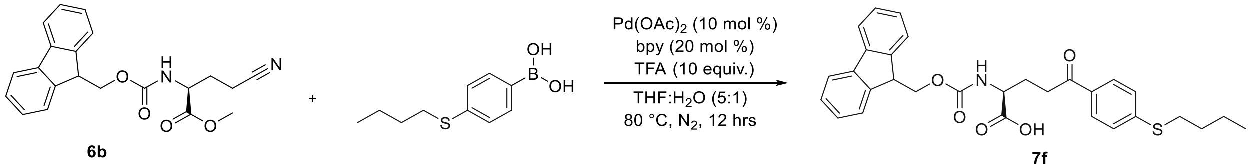

Fmoc-Gln(Butylthiophenylketone)-CO_2H **7f** was synthesized according to **GP-VI** with a reaction time of 12 hrs. Purification by silica gel column chromatography (1:1 EtOAc:Hex eluent) yielded the product **7e** as a yellow-white solid (121 mg, 78% yield). Analytical TLC, 1:19 MeOH:DCM eluent (with 0.1% AcOH), R_f = 0.50.

^{1}**H NMR** (400 MHz, DMSO): δ = 7.86 (dd, *J* = 12.8, 8.1 Hz, 4H), 7.74 – 7.70 (m, 2H), 7.42 – 7.27 (m, 6H), 4.29 – 4.18 (m, 3H), 4.08 (td, *J* = 9.6, 8.9, 4.8 Hz, 1H), 3.17 – 2.97 (m, 4H), 2.19 – 2.08 (m, 1H), 1.99 – 1.90 (m, 1H), 1.56 (p, 2H), 1.39 (h, *J* = 7.2 Hz, 2H), 0.87 (t, *J* = 7.3 Hz, 3H) ppm. ^{13}**C NMR** (101 MHz, DMSO): δ = 198.05, 173.75, 156.20, 144.19, 143.80, 140.74, 133.02, 128.42, 127.66, 127.09, 125.99, 125.29, 120.14, 65.67, 53.14, 46.68, 34.24, 30.39, 30.35, 25.45, 21.37, 13.49 ppm. **HRMS**: calcd. for $C_{30}H_{32}NO_5S^+$ [M+H$^+$] 518.1996; found 518.1982.

^{1}H NMR of Fmoc-Gln(Butylthiophenylketone)-CO_2H 7f

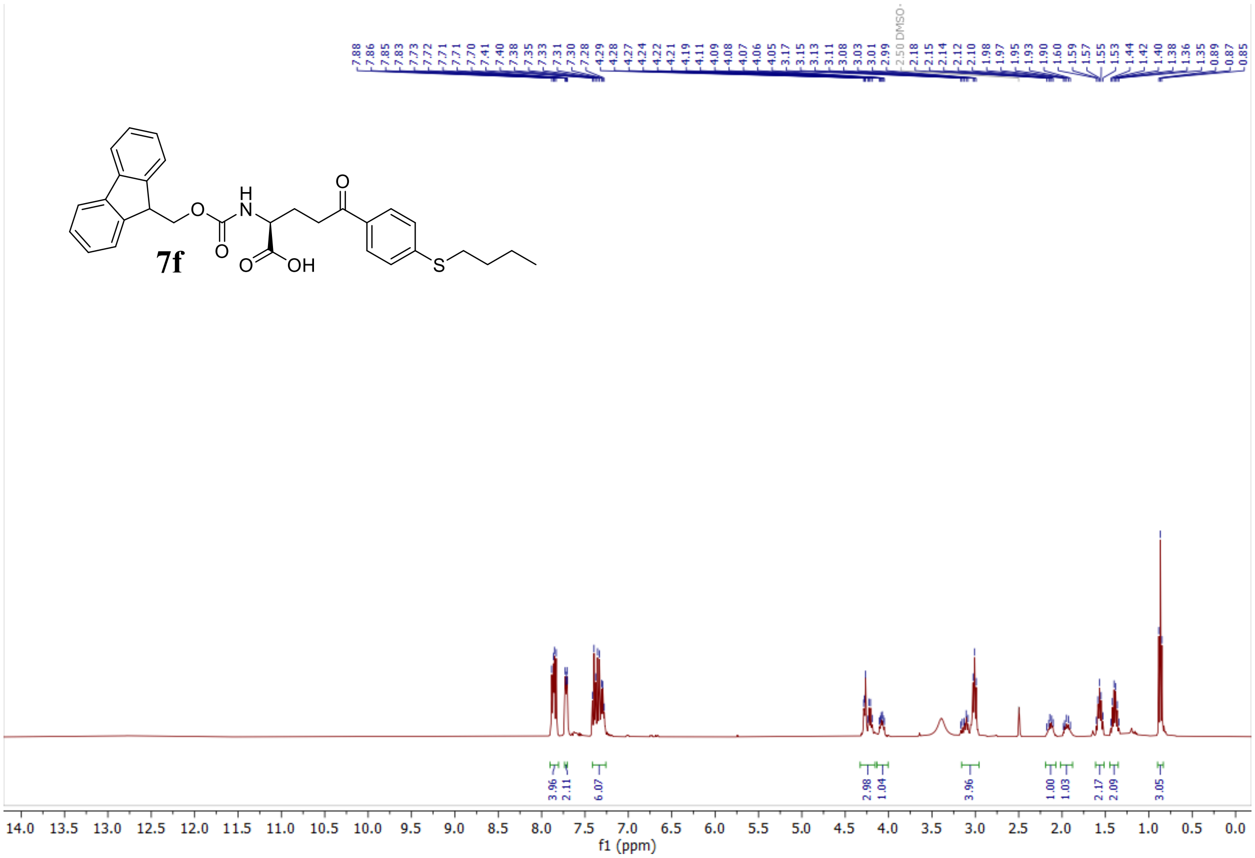

^{13}C NMR of Fmoc-Gln(Butylthiophenylketone)-CO_2H 7f

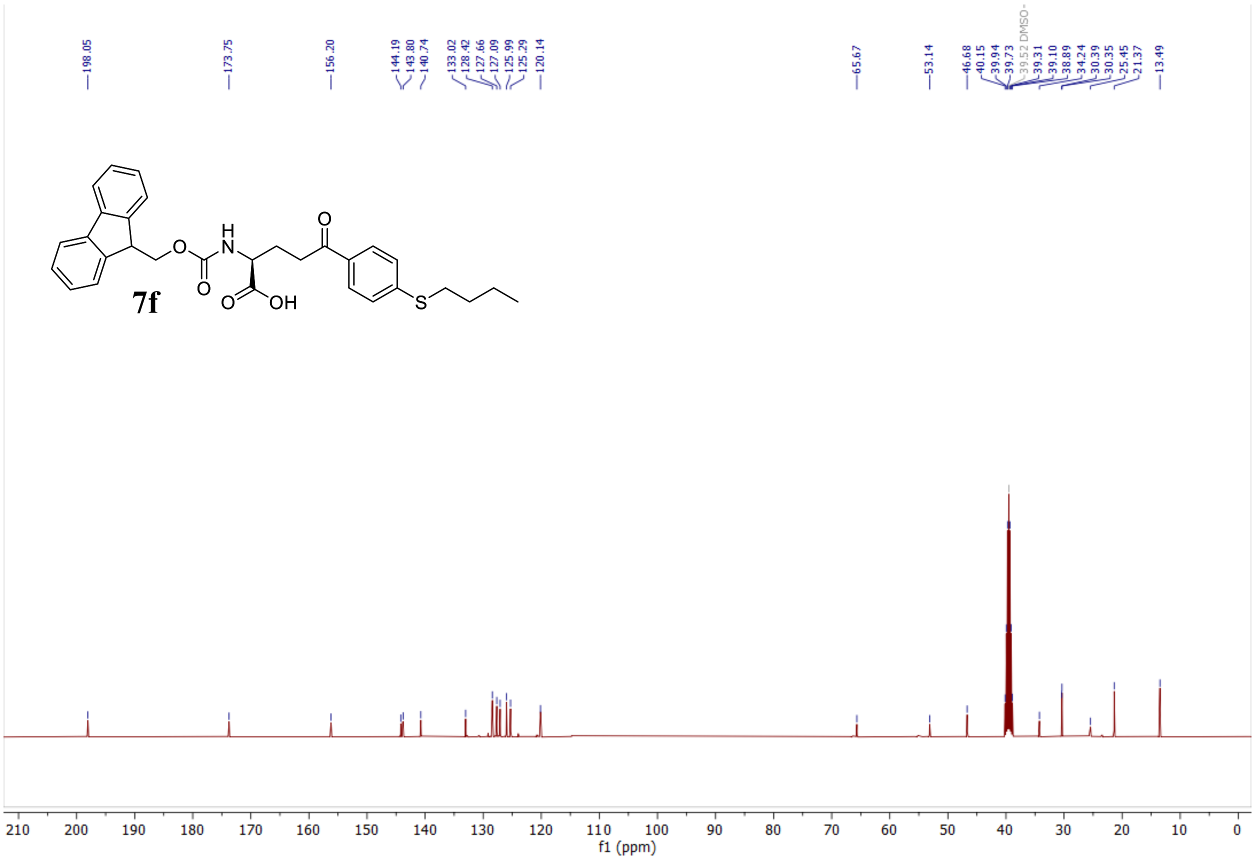

Synthesis of Fmoc-Gln(3-thiophene)-CO_2H 7g

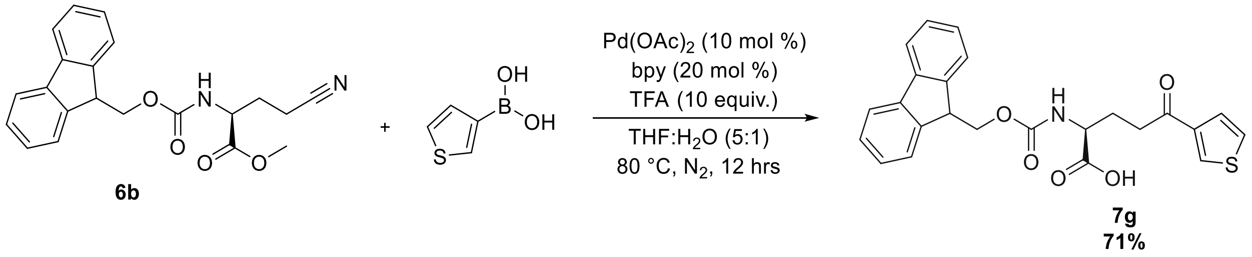

Fmoc-Gln(3-thiophene)-CO_2H **7g** was synthesized according to **GP-VI** with a reaction time of 12 hrs. Purification by silica gel column chromatography (1:1 EtOAc:Hex eluent) yielded the product **7g** as a viscous, pale white-brown solid (92.8 mg, 71% yield). Analytical TLC, 1:19 MeOH:DCM eluent (with 0.1% AcOH), $R_f = 0.40$.

^{1}H NMR (400 MHz, DMSO): δ = 8.44 (dd, J = 2.9, 1.3 Hz, 1H), 7.89 (d, J = 7.5 Hz, 2H), 7.72 (dt, J = 8.5, 4.5 Hz, 2H), 7.62 (dd, J = 5.1, 2.8 Hz, 1H), 7.48 (dd, J = 5.1, 1.3 Hz, 1H), 7.41 (t, J = 7.3 Hz, 2H), 7.31 (td, J = 7.4, 3.7 Hz, 2H), 4.35 – 4.19 (m, 3H), 4.05 (td, J = 8.9, 8.1, 4.8 Hz, 1H), 3.11 – 2.93 (m, 2H), 2.15 – 2.05 (m, 1H), 1.97 – 1.85 (m, 1H) ppm. **^{13}C NMR** (101 MHz, DMSO): δ = 193.58, 173.72, 156.19, 143.85, 141.73, 140.75, 133.49, 127.66, 127.49, 127.09, 126.48,

125.29, 120.15, 65.65, 53.16, 46.66, 35.63, 25.33 ppm. **HRMS**: calcd. for $C_{24}H_{20}NO_5S^-$ [M-H$^+$] 434.1068; found 434.1050.

^{1}H NMR of Fmoc-Gln(3-thiophene)-CO$_2$H 7g

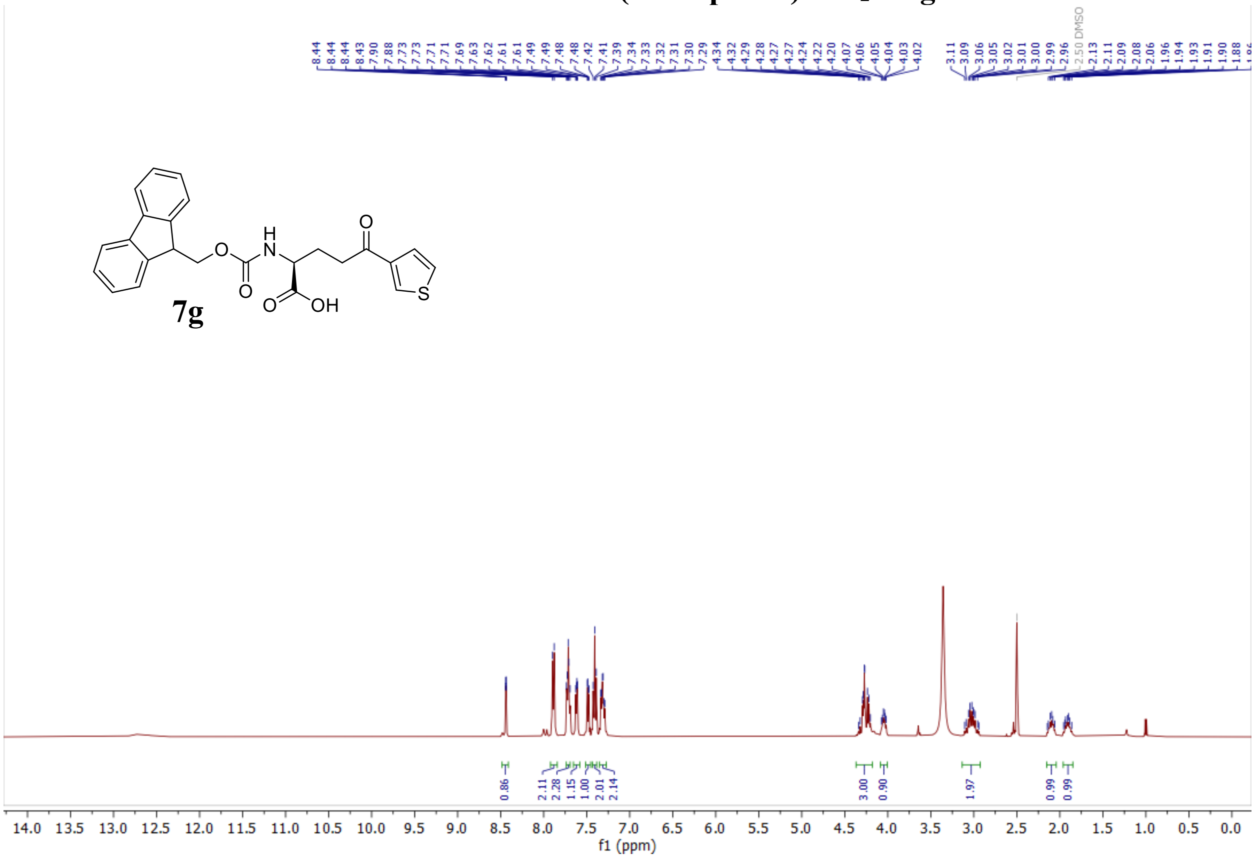

^{13}C NMR of Fmoc-Gln(3-Thiophene)-CO_2H 7g

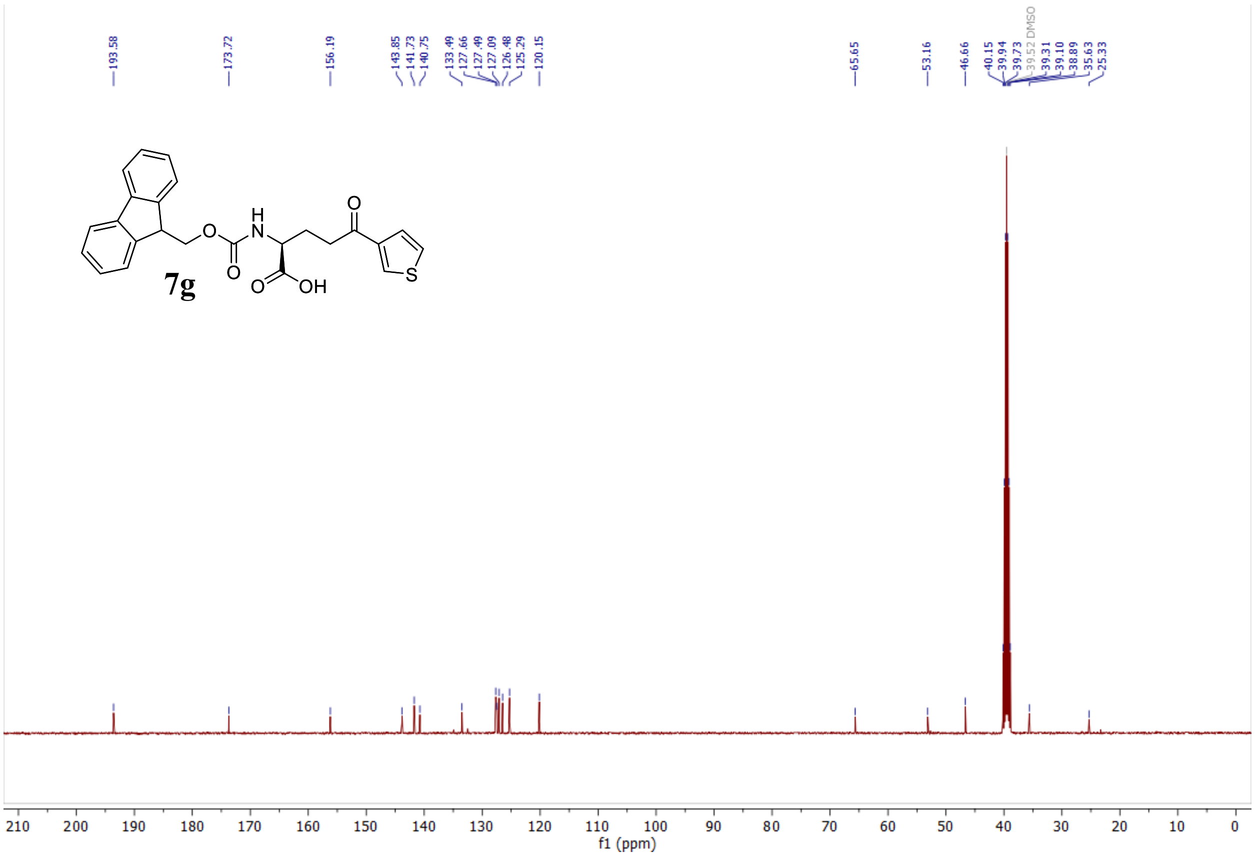

Synthesis of Fmoc-Gln(paramethoxyphenylketone)-CO_2H 7h

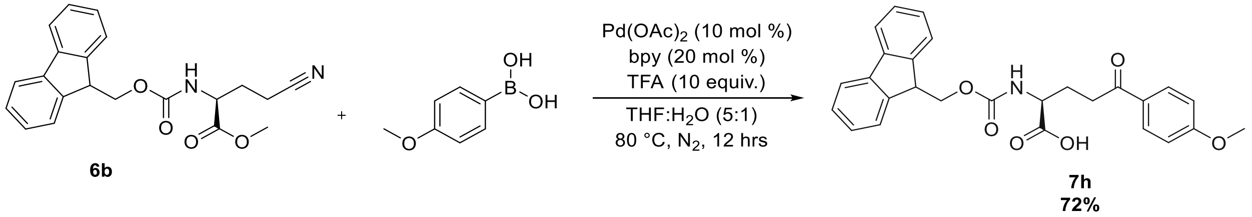

Fmoc-Gln(paramethoxyphenylketone)-CO_2H **7h** was synthesized according to **GP-VI** with a reaction time of 12 hrs. Purification by silica gel column chromatography (5:1 EtOAc:Hex eluent) yielded the product **7h** as an opaque, white gummy solid (99.3 mg, 72% yield). Analytical TLC, 1:19 MeOH:DCM eluent (with 0.1% AcOH), $R_f = 0.30$.

^{1}H NMR (400 MHz, DMSO): δ = 7.93 – 7.87 (m, 4H), 7.72 (dd, *J* = 7.8, 4.2 Hz, 2H), 7.41 (t, *J* = 7.5 Hz, 2H), 7.31 (td, *J* = 8.1, 7.4, 5.1 Hz, 2H), 7.02 (d, *J* = 9.0 Hz, 2H), 4.30 – 4.18 (m, 3H), 4.07 (td, *J* = 9.6, 4.8 Hz, 1H), 3.82 (s, 3H), 3.16 – 2.95 (m, 2H), 2.17 – 2.05 (m, 1H), 1.99 – 1.86 (m, 1H) ppm. **^{13}C NMR** (101 MHz, DMSO): δ = 197.46, 173.77, 163.11, 156.19, 143.87, 140.73, 130.15, 129.53, 127.67, 127.09, 125.29, 120.14, 113.89, 65.65, 55.53, 53.18, 46.67, 34.09, 25.52 ppm. **HRMS**: calcd. for $C_{27}H_{26}NO_6^+$ [M+H$^+$] 460.1755; found 460.1749.

^{1}H NMR of Fmoc-Gln(paramethoxyphenylketone)-CO_2H 7h

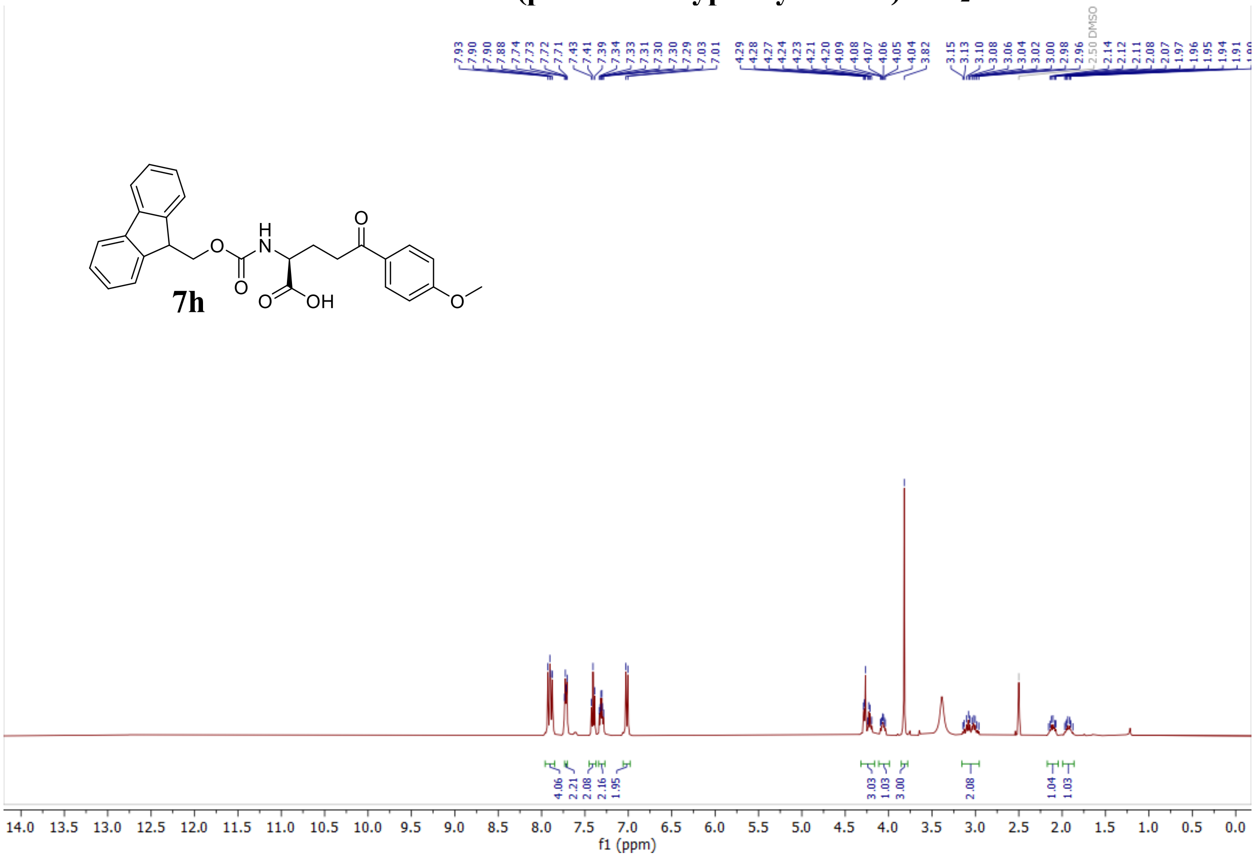

^{13}C NMR of Fmoc-Gln(paramethoxyphenylketone)-CO_2H 7h

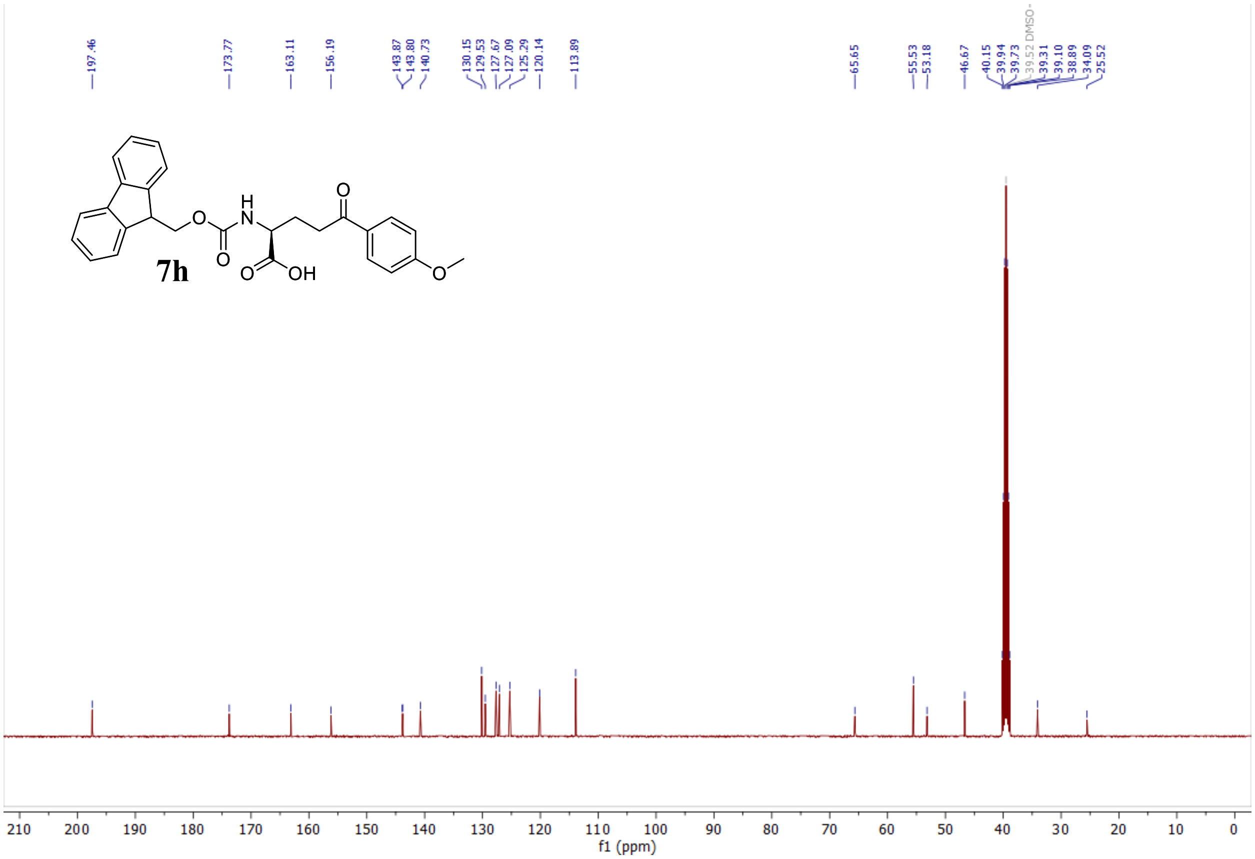

Synthesis of Fmoc-Gln(3-sulfonamide phenylketone)-CO_2H 7i

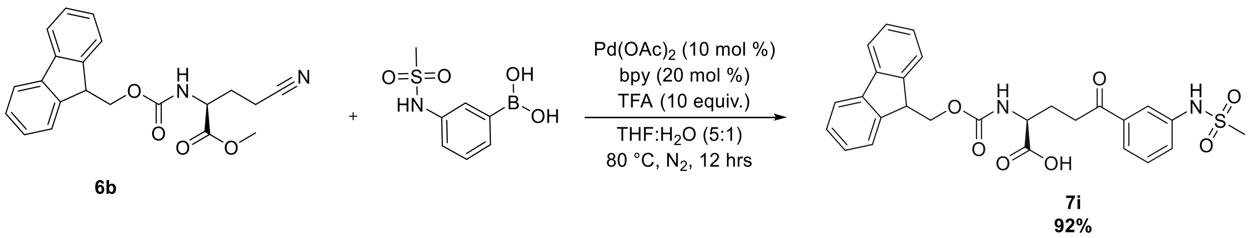

Fmoc-Gln(3-sulfonamide phenylketone)-CO_2H **7i** was synthesized according to **GP-VI** with a reaction time of 12 hrs. Purification by silica gel column chromatography (1:1 EtOAc:Hex eluent) yielded the product **7i** as a gummy white solid (144 mg, 92% yield). Analytical TLC, 1:19 MeOH:DCM eluent (with 0.1% AcOH), $R_f = 0.25$.

^{1}H NMR (400 MHz, DMSO): δ = 9.98 (s, 1H), 7.88 (d, J = 7.6 Hz, 2H), 7.78 – 7.66 (m, 4H), 7.54 – 7.45 (m, 2H), 7.40 (t, J = 6.8 Hz, 2H), 7.31 (td, J = 7.3, 4.5 Hz, 2H), 4.30 – 4.19 (m, 3H), 4.06 (td, J = 9.3, 4.8 Hz, 1H), 3.01 (s, 3H), 2.13 (dq, J = 13.6, 6.6 Hz, 1H), 1.93 (ddt, J = 13.4, 8.6, 4.3 Hz, 1H) ppm. **^{13}C NMR** (101 MHz, DMSO): δ = 198.62, 173.72, 156.20, 143.80, 140.73, 138.96, 137.58, 129.84, 129.39, 127.67, 127.09, 125.28, 120.13, 118.33, 115.24, 65.64, 53.09, 46.66, 34.60, 25.21, 23.32 ppm. **HRMS**: calcd. for $C_{27}H_{25}N_2O_7S^-$ [M-H$^+$] 521.1388; found 521.1368.

^{1}H NMR of Fmoc-Gln(3-sulfonamide phenylketone)-CO_2H 7i

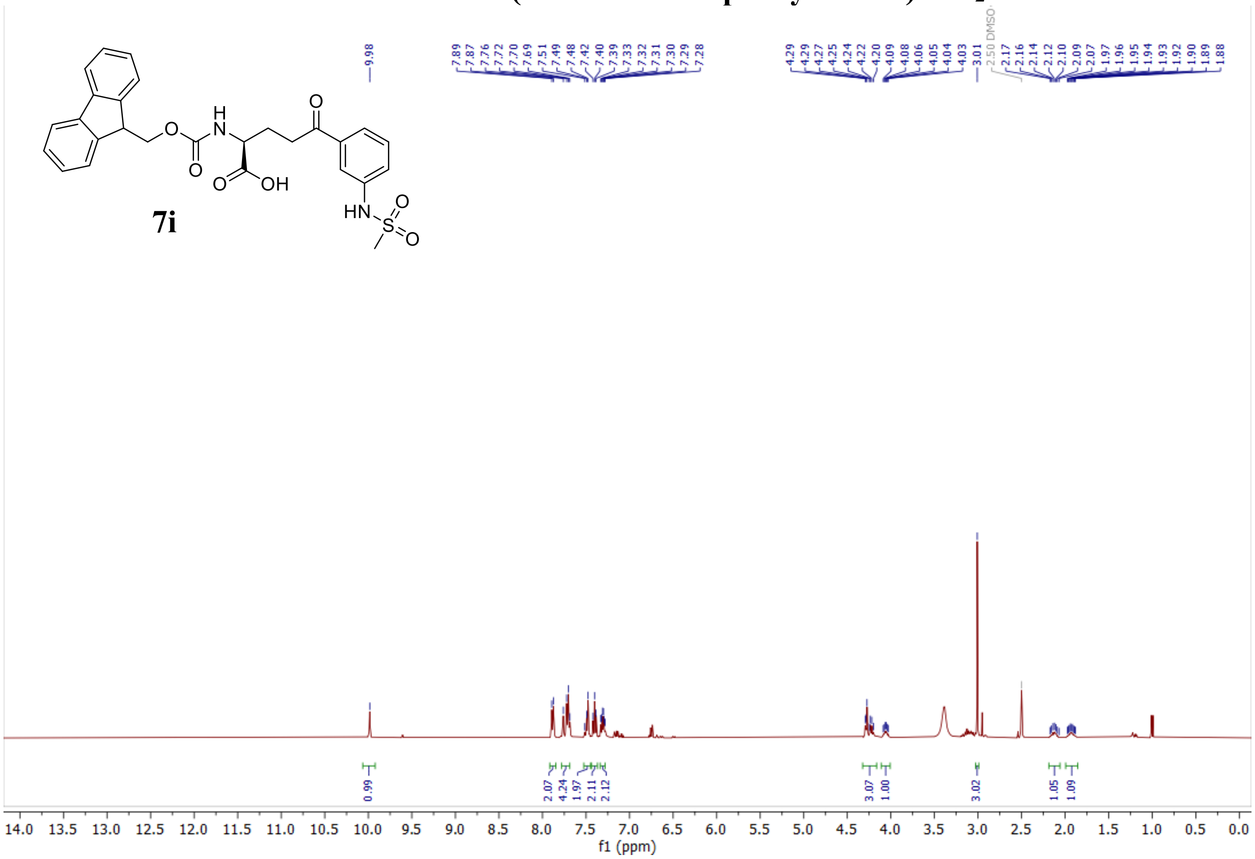

^{13}C NMR of Fmoc-Gln(3-sulfonamide phenylketone)-CO_2H 7i

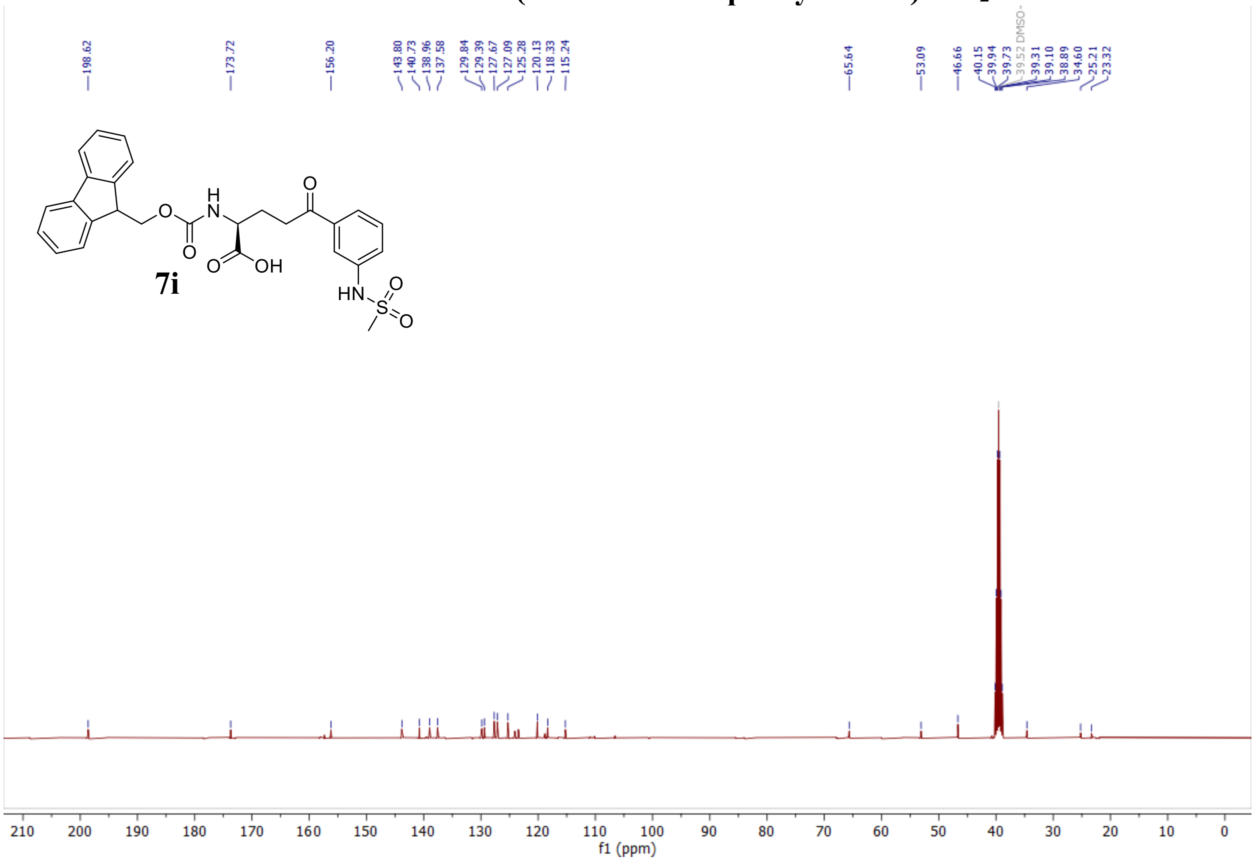

Supplementary Fig. 9b: Synthesis of Peptides Using Unnatural Amino Acid Building Blocks

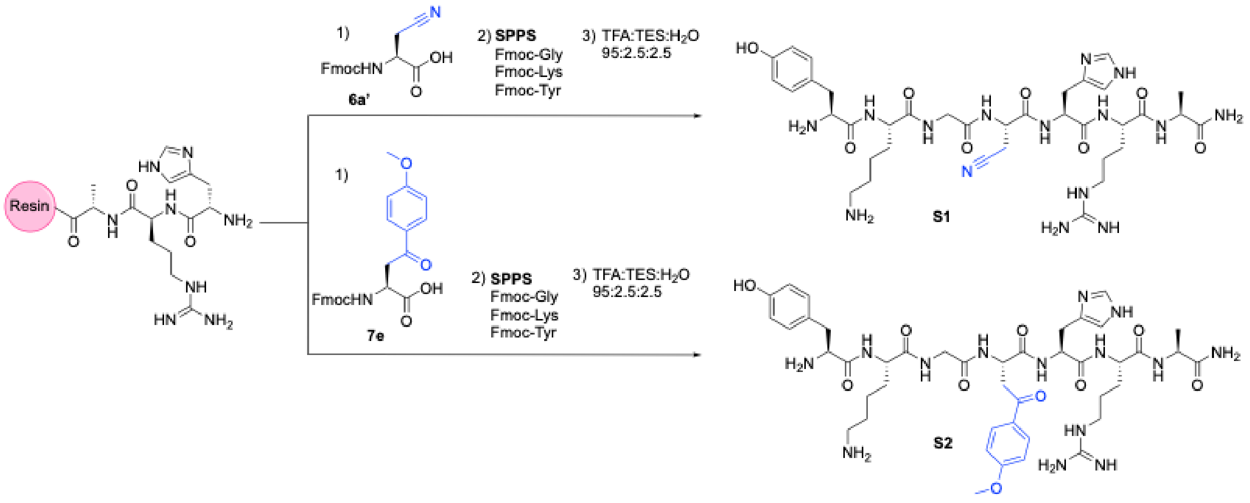

Synthesis of Nitrile Peptide H_2N-YKGN(Nitrile)HRA-$CONH_2$ (S1) using Fmoc-Asn(nitrile)-CO_2H

Peptide synthesized via standard SPPS using Fmoc-Asn(CN)-CO_2H (**6a′**). **6a′** (2 equiv.) was coupled overnight after activation with Oxyma Pure (5 equiv.) and DIEA (5 equiv.). Other couplings were done with standard HBTU/DIEA conditions as detailed in Section IV.

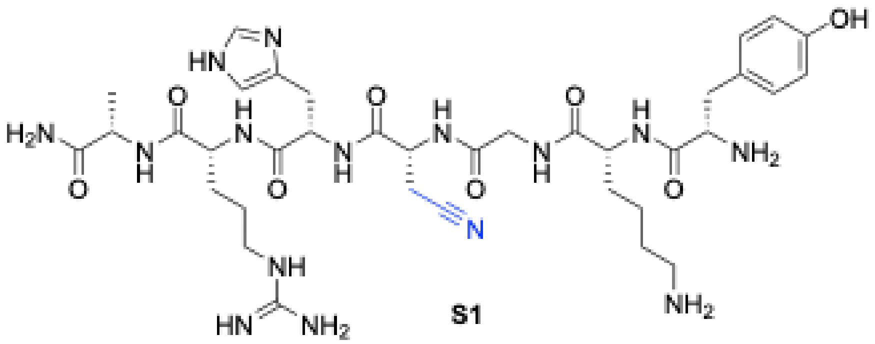

H_2N-YKGN(Nitrile)HRA-$CONH_2$ (S1): LCMS, *m/z* 826.4 (calcd. $[M+H^+]$ = 826.4431), Purity: >90% (HPLC analysis at 220 nm). Retention time using **HPLC Method A:** 14.2 min.

HPLC Trace for H_2N-YKGN(Nitrile)HRA-CO_2H S1

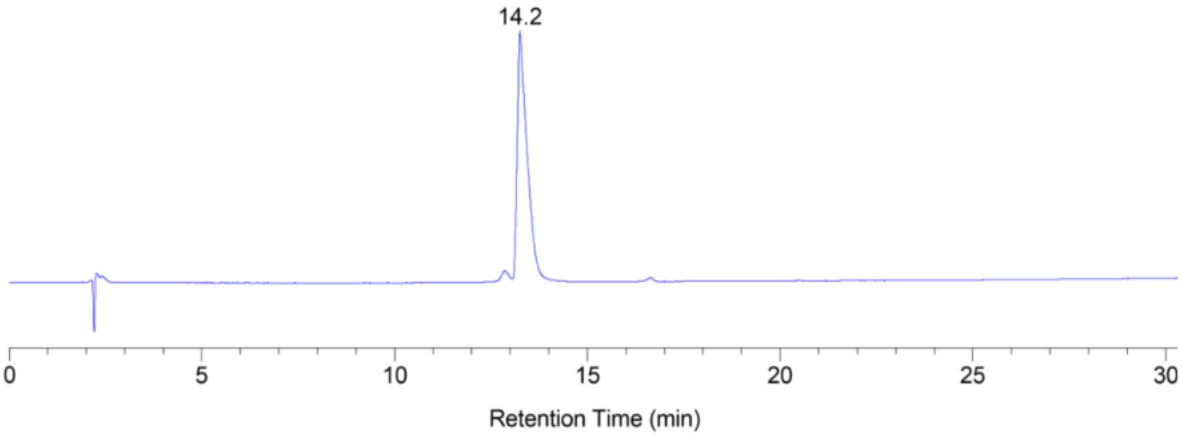

HRMS of H_2N-YKGN(Nitrile)HRA-CO_2H S1

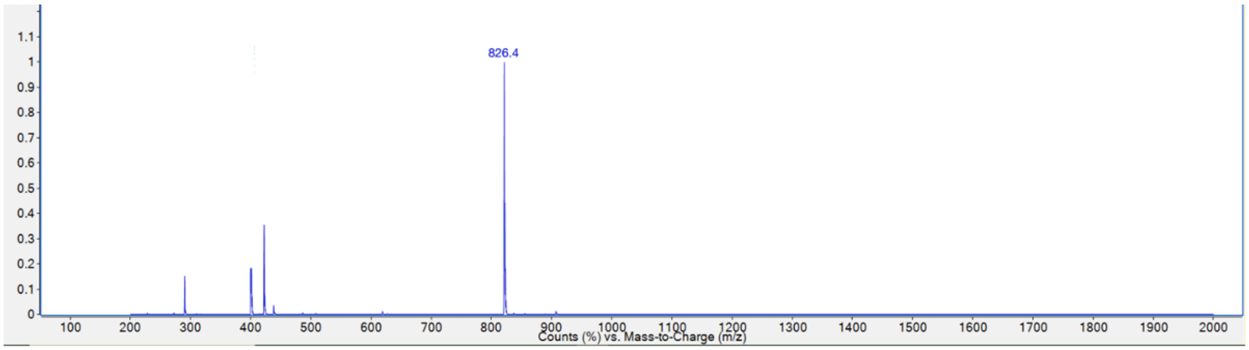

Synthesis of Ketone Peptide H_2N-YKGN(Nitrile)HRA-$CONH_2$ (S2) using Fmoc-Asn(Para-OMe)-CO_2H

Peptide synthesized via standard SPPS using Fmoc-Asn(Para-OMe)-CO_2H (**7e**). **7e** (2 equiv.) was coupled overnight after activation with Oxyma Pure (5 equiv.) and DIEA (5 equiv.). Other couplings were done with standard HBTU/DIEA conditions as detailed in Section IV.

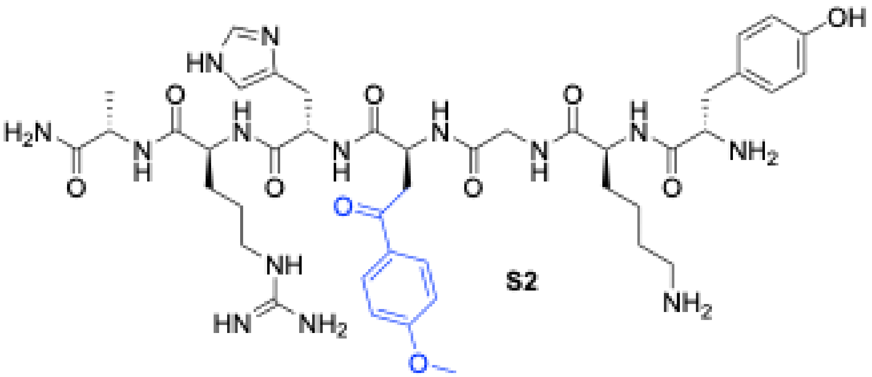

H_2N-YKGN(4-methoxyphenylketone)HRA-$CONH_2$ (S2): LCMS, *m/z* 935.5016 (calcd. $[M+H^+]$ = 935.4846), Purity: >90% (HPLC analysis at 220 nm). Retention time using **HPLC Method A:** 16.7 min.

HPLC Trace for H_2N-YKGN(4-methoxyphenylketone)HRA-CO_2H S2

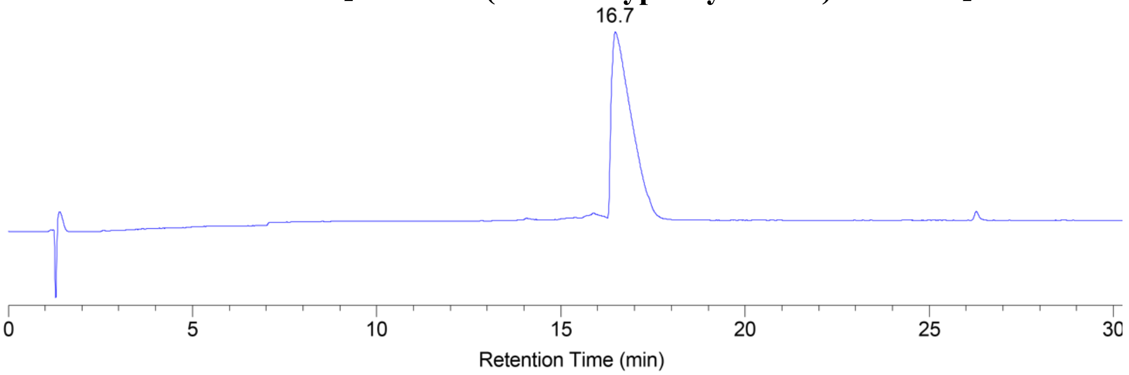

HRMS of H_2N-YKGN(4-methoxyphenylketone)HRA-CO_2H S2

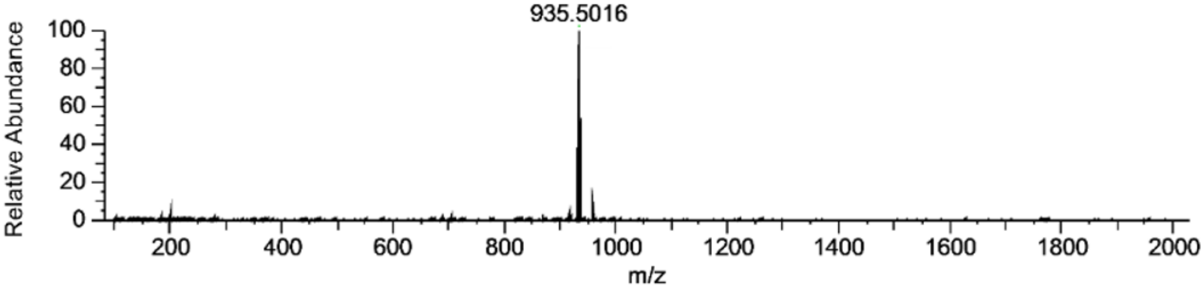

Supplementary Fig. 10: Chemoselectivity of Boronic Acid Cross-Coupling

Synthesis of H_2N-KYWCSMEHR-CO_2H S3

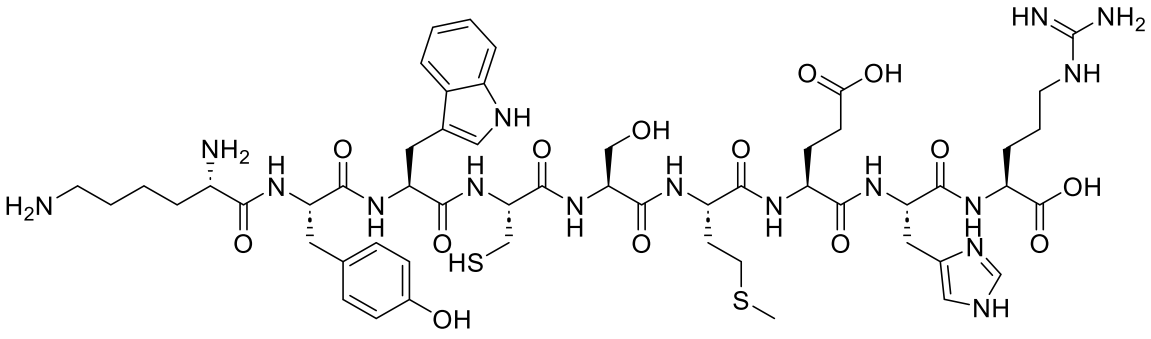

H_2N-KYWCSMEHR-CO_2H (S3): LCMS, *m/z* 1239.5383 (calcd. [$M+H^+$] = 1239.5398), *m/z* 620.2731 (calcd. [$(M+2H^+)/2$] = 620.2735), *m/z* 413.8512 (calcd. [$(M+3H^+)/3$] = 413.8514), Purity: >99% (HPLC analysis at 220 nm). Retention time using **HPLC Method B**: 9.8 min.

HPLC Trace for S3

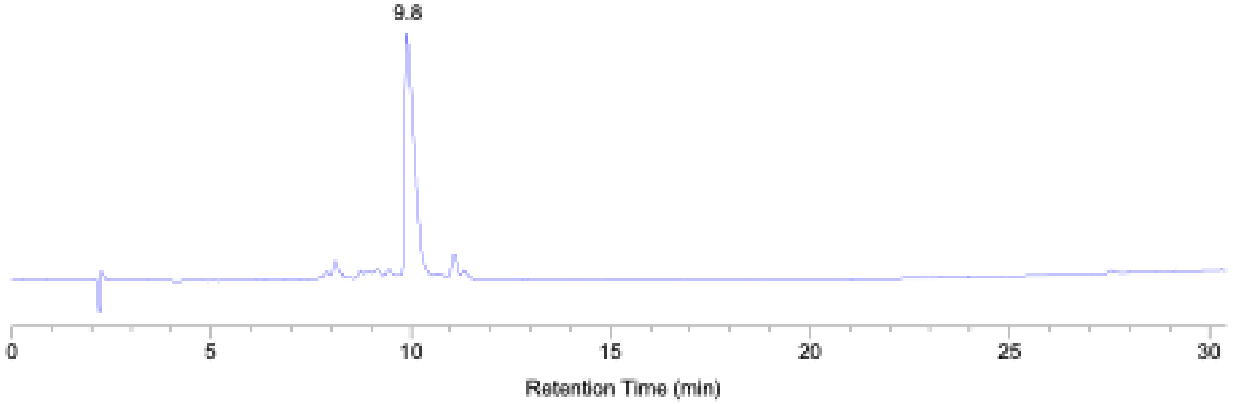

HRMS of S3

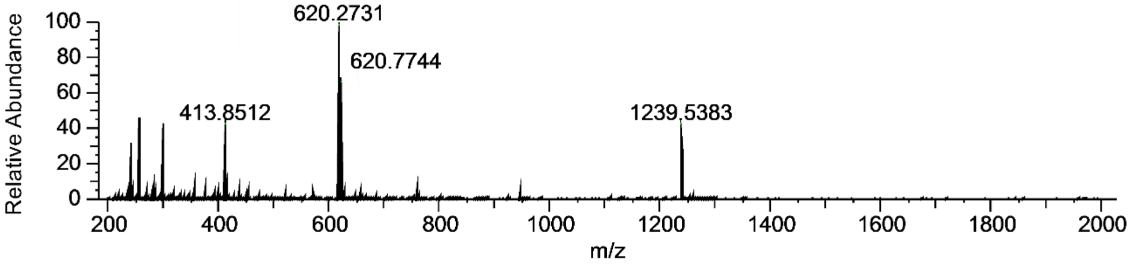

Chemoselectivity Control: Boronic Acid Cross-Coupling Conditions

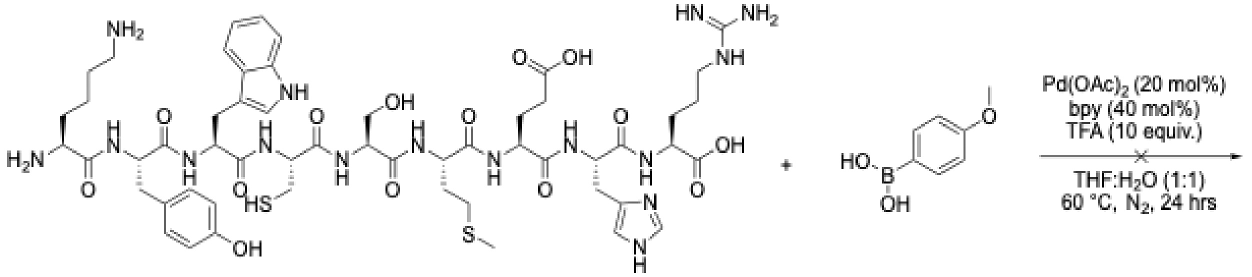

H_2N-KYWCSMEHR-CO_2H (S3) (1 mg, 0.8 µmol, 1 equiv.) was dissolved in 600 µL of 1:1 H_2O:THF in a 1/2" dram vial. Next, 4-methoxyphenyl boronic acid (500 µg, 3.2 µmol, 4 equiv.) and 2,2'-bipyridyl ligand (16 µg, 0.08 µmol, 10 mol%) from 10 mM stock solutions in 1:1 H_2O:THF were added to the vial. Then, TFA (0.8 µL, 8 µmol, 10 equiv.) was added at once via pipette. N_2 was bubbled for 2 minutes using an 18G x 1 ½" needle and balloon. Finally, $Pd(OAc)_2$ (40 µg, 0.16 µmol, 20 mol%) was added from freshly prepared 10 mM stock solution in 1:1 H_2O:THF. The vial was stirred at room temperature for 24 hours then quenched with 3-MPA (1.4 µL, 16 µmol, 20 equiv.). The reaction was analyzed via **HPLC Method B**. No conversion occurred.

Crude HPLC Trace for Boronic Acid Cross-Coupling Conditions with S3

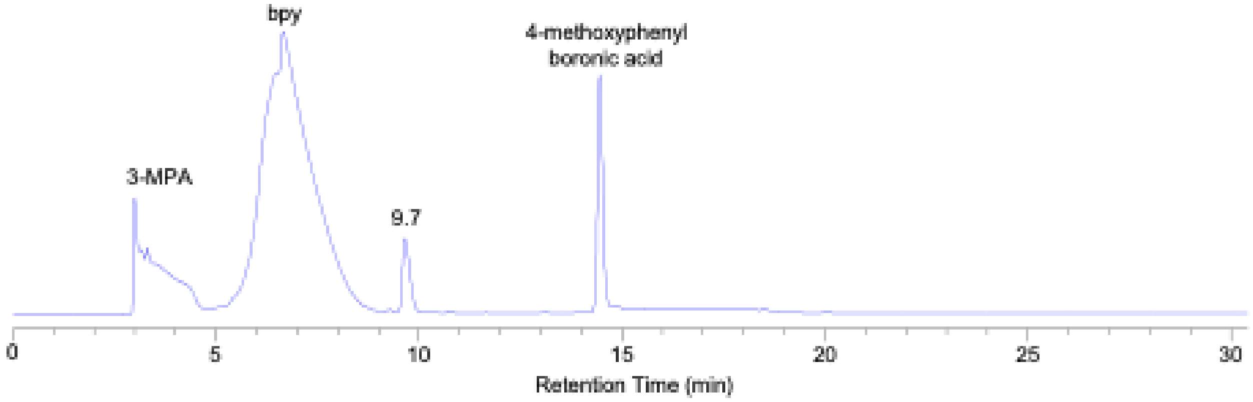

Supplementary Fig. 11: Solution Phase Peptide Diversification with Aryl Boronic Acids

Synthesis of H_2N-FRNFG-CO_2H 8

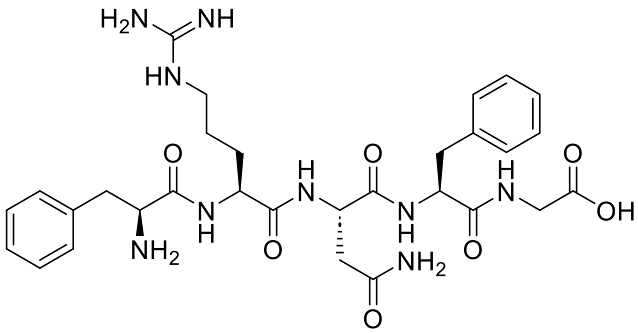

H_2N-FRNFG-CO_2H (8): LCMS, *m/z* 640.3207 (calcd. [*M*+H^+] = 640.3202), *m/z* 320.6639 (calcd. [(M+$2H^+$)/2] = 320.6637), Purity: >99% (HPLC analysis at 220 nm). Retention time using **HPLC Method A**: 5.7 min.

HPLC Trace for H_2N-FRNFG-CO_2H 8

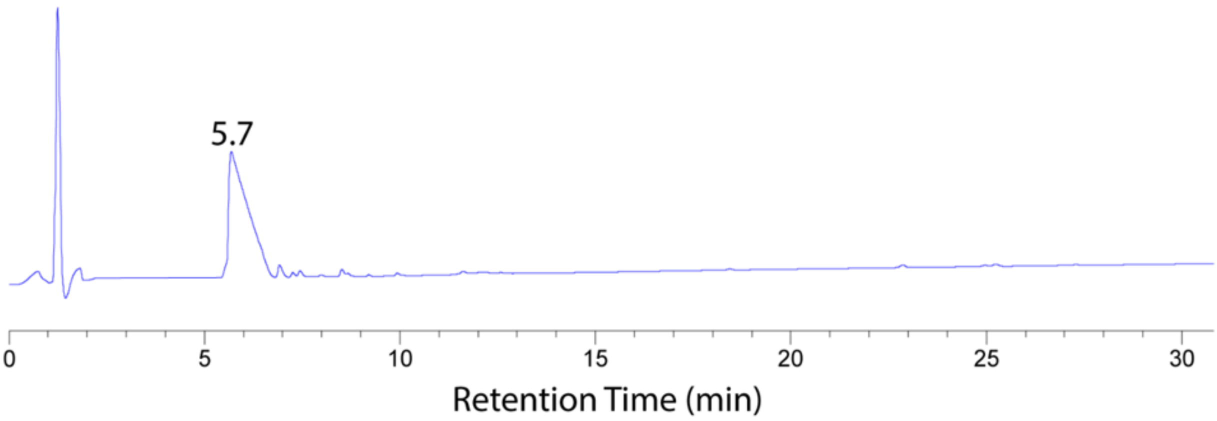

HRMS of H_2N-FRNFG-CO_2H 8

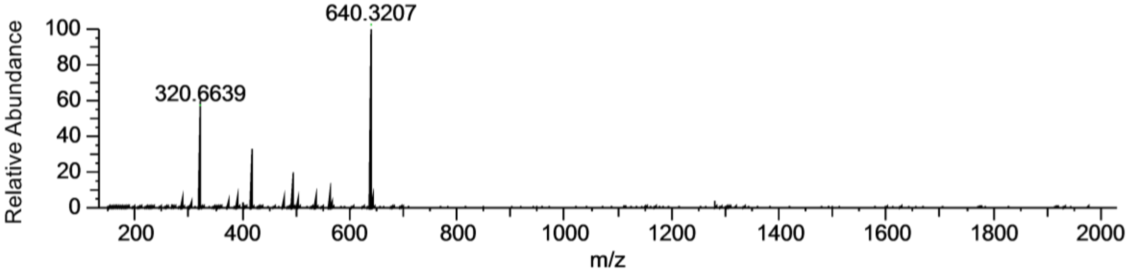

General Procedure for Large-Scale Dehydration of Asn/Gln to Nitrile on Peptide (GP-VII):

Asn/Gln-containing peptide (0.16 mmol, 1 equiv.) was dissolved in 3 mL of 1:1 H_2O:ACN in a 1/2" dram vial. Next, $Pd(O_2CCF_3)_2$ (106 mg, 0.32 mmol, 2 equiv.) was added in one portion. The concentration of peptide in the solution was 53.3 mM. The vial was stirred at room temperature for 36 hours then quenched with 3-MPA (283 µL, 20 equiv.). The reaction was analyzed via **HPLC Method A** to determine percent conversion to the nitrile isohypsic dehydration product. Fractions were then collected via preparative HPLC and lyophilized to produce pure nitrile-containing peptide.

Synthesis of H_2N-FRN(Nitrile)FG-CO_2H 9

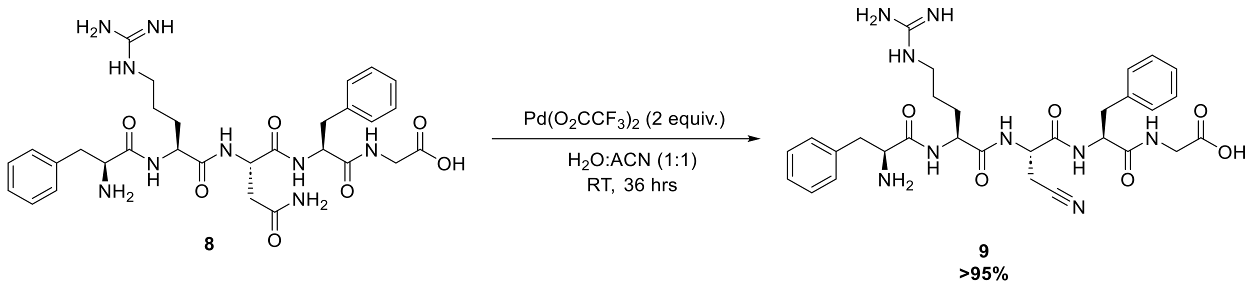

H_2N-FRN(Nitrile)FG-CO_2H **(9)** was synthesized according to **GP-VII**. Conversion was ascertained to be >95% using **HPLC Method A**. Fractions were then collected via preparative HPLC and lyophilized to produce pure **9** as a fluffy white powder (83.1 mg, 86% yield).

H_2N-FRN(Nitrile)FG-CO_2H peptide (9): LCMS, *m/z* 622.3210 (calcd. [M+H^+] = 622.3102) Purity: >99% (HPLC analysis at 220 nm). Retention time using **HPLC Method A**: 7.0 min.

HPLC Trace for H_2N-FRN(Nitrile)FG-CO_2H 9

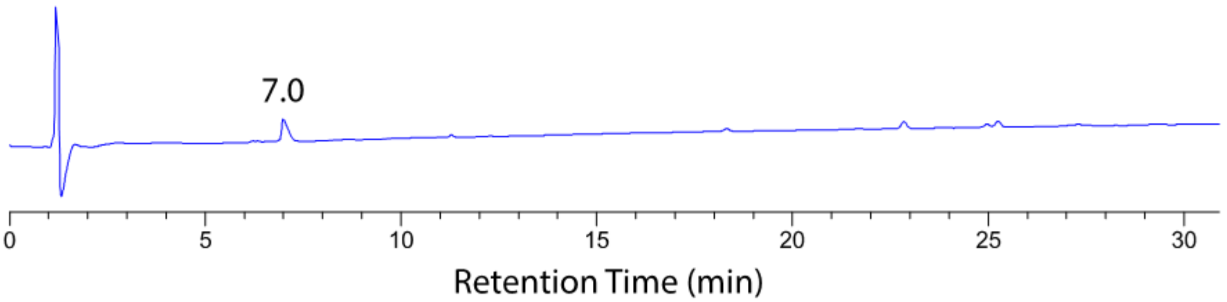

HRMS of H_2N-FRN(Nitrile)FG-CO_2H 9

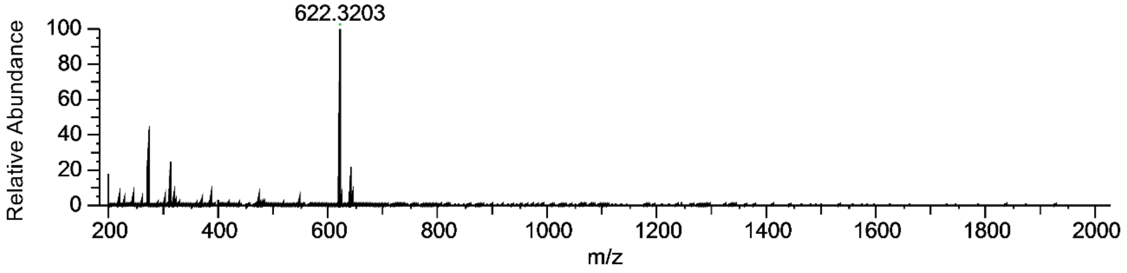

General Procedure for Nitrile Boronic Acid Carbometallation Reaction on Peptide in Solution Phase (GP-VIII):

H_2N-FRN(Nitrile)FG-CO_2H (**9**) (1.0 mg, 1.6 μmol, 1 equiv.) and the appropriate boronic acid (9.65 μmol, 6 equiv.) were added to a 1/2” dram vial. Next, 2,2’-bipyridyl ligand (0.10 mg, 0.64 μmol, 40 mol%) was added from a freshly prepared stock solution (30 μL, 1:1 H_2O:THF). TFA (~2 μL, 16.1 μmol, 10 equiv.) was then transferred to the solution, after which 270 μL H_2O:THF (1:1) was added to bring the total reaction volume to 300 μL. N_2 was bubbled for 2 minutes using

an 18G x 1 ½” needle and balloon. Finally, $Pd(OAc)_2$ (0.07 mg, 0.32 µmol, 20 mol%) was added in 10 µL from a freshly prepared stock solution (1:1 H_2O:THF). N_2 was bubbled for another 60 seconds using an 18G x 1 ½” needle and balloon, and then the vial was flushed with N_2. The reaction was left stirring for 12 hours at 60 °C. The reaction was quenched with 3-MPA (3 µL, 20 equiv.) and analyzed via **HPLC Method A** to determine conversion to the aryl ketone product based on remaining starting material.

General Procedure for Carbometallation Reaction on Peptide in Solution Phase – Strongly Electron-Withdrawing Boronic Acid Substrates (GP-IX):

H_2N-FRN(Nitrile)FG-CO_2H (**9**) (1.0 mg, 1.6 µmol, 1 equiv.) and the appropriate boronic acid (9.65 µmol, 6 equiv.) were added to a 1/2” dram vial. Next, 2,2’-bipyridyl ligand (0.25 mg, 1.6 µmol, 1 equiv.) was added from a freshly prepared stock solution (30 µL, 1:1 H_2O:THF). TFA (~2 µL, 16.1 µmol, 10 equiv.) was then transferred to the solution, after which 270 µL H_2O:THF (1:1) was added to bring the total reaction volume to 300 µL. N_2 was bubbled for 2 minutes using an 18G x 1 ½” needle and balloon. Finally, $Pd(OAc)_2$ (0.18 mg, 0.80 µmol, 50 mol%) was added in 10 µL from a freshly prepared stock solution (1:1 H_2O:THF). N_2 was bubbled for another 60 seconds using an 18G x 1 ½” needle and balloon, and then the vial was flushed with N_2. The reaction was left stirring for 48 hours at 80 °C. The reaction was quenched with 3-MPA (3 µL, 20 equiv.) and analyzed via **HPLC Method A** to determine conversion to the aryl ketone product based on remaining starting material.

Synthesis of H_2N-FRN(phenylketone)FG-CO_2H 10a

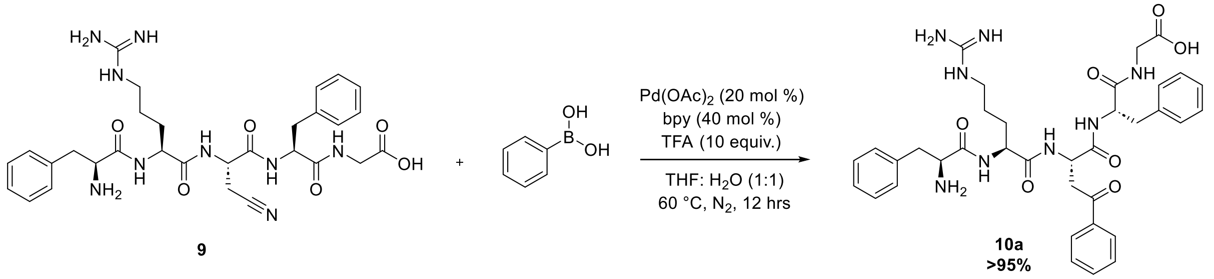

H_2N-FRN(phenylketone)FG-CO_2H **(10a)** was synthesized according to **GP-VIII**. Conversion was ascertained to be >95% using **HPLC Method A** based on remaining starting nitrile peptide.

H_2N-FRN(phenylketone)FG-CO_2H (10a): LCMS, *m/z* 701.3371 (calcd. $[M+H^+]$ = 701.3406). Purity: >99% (HPLC analysis at 220 nm). Retention time using **HPLC Method A:** 11.5 min.

Crude HPLC Trace for H_2N-FRN(phenylketone)FG-CO_2H 10a

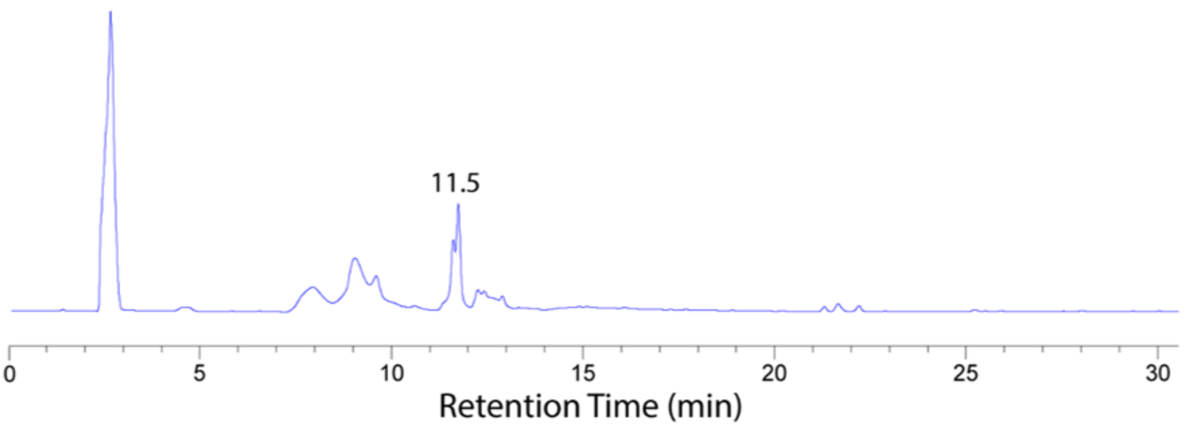

HRMS of H_2N-FRN(phenylketone)FG-CO_2H 10a

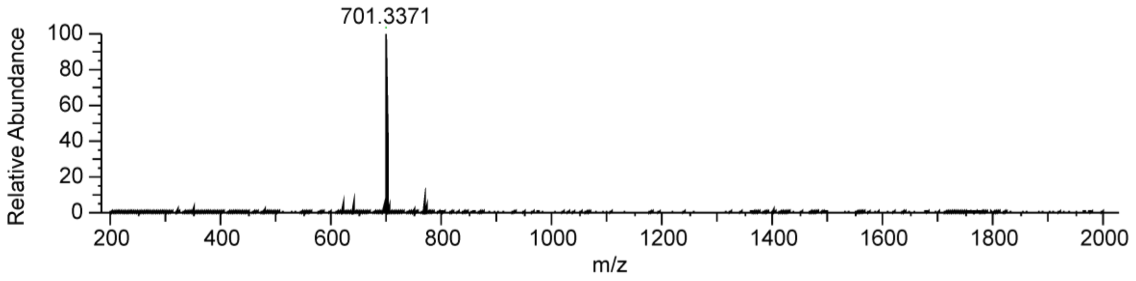

Synthesis of H_2N-FRN(paramethoxyphenylketone)FG-CO_2H 10b

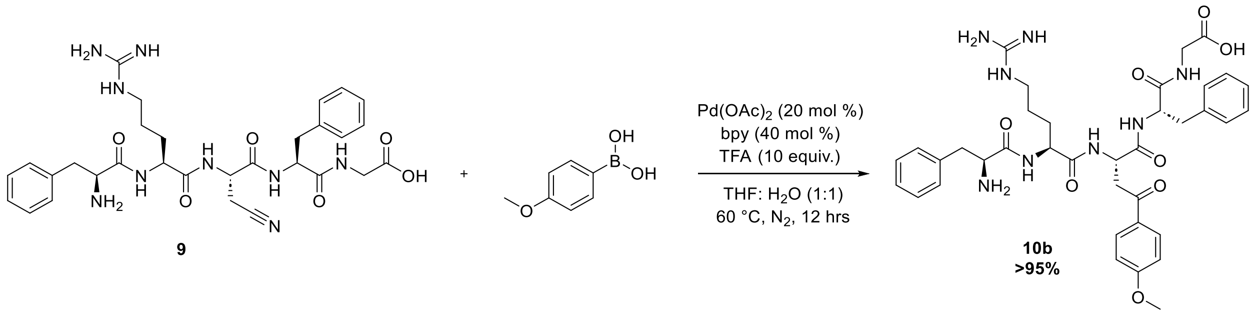

H_2N-FRN(paramethoxyphenylketone)FG-CO_2H **(10b)** was synthesized according to **GP-VIII**. Conversion was ascertained to be >95% using **HPLC Method A** based on remaining starting nitrile peptide.

H_2N-FRN(paramethoxyphenylketone)FG-CO_2H (10b): LCMS, *m/z* 731.3367 (calcd. $[M+H^+]$ = 731.3511), *m/z* 366.1720 (calcd. $[(M+2H^+)/2]$ = 366.1792). Purity: >99% (HPLC analysis at 220 nm). Retention time using **HPLC Method A:** 9.2 min.

Crude HPLC Trace for H_2N-FRN(paramethoxyphenylketone)FG-CO_2H 10b

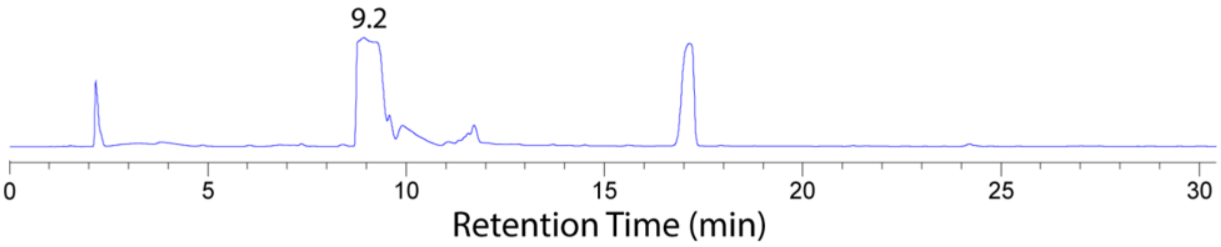

HRMS of H_2N-FRN(paramethoxyphenylketone)FG-CO_2H 10b

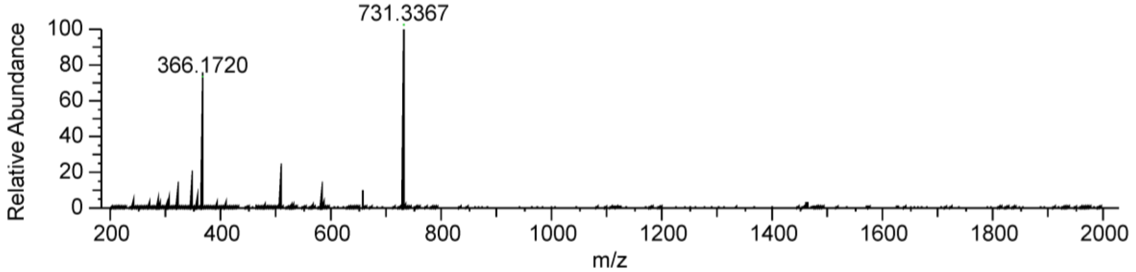

Synthesis of H_2N-FRN(Triphenylamine)FG-CO_2H 10c

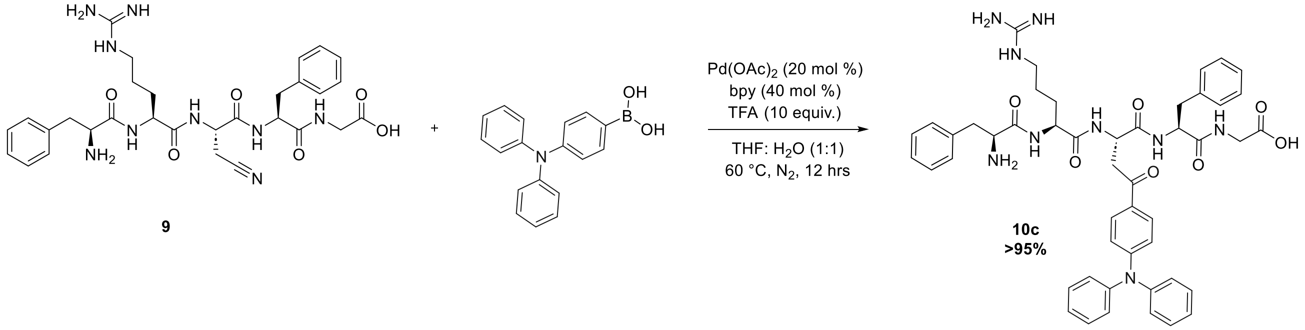

H_2N-FRN(Triphenylamine)FG-CO_2H **(10c)** was synthesized according to **GP-VIII**. Conversion was ascertained to be >95% using **HPLC Method A** based on remaining starting nitrile peptide.

H_2N-FRN(Triphenylamine)FG-CO_2H (10c): LCMS, *m/z* 868.4139 (calcd. $[M+H^+]$ = 868.4141), *m/z* 434.7105 (calcd. $[(M+2H^+)/2]$ = 434.7107). Purity: >99% (HPLC analysis at 220 nm). Retention time using **HPLC Method A:** 15.0 min.

Crude HPLC Trace for H_2N-FRN(Triphenylamine)FG-CO_2H 10c

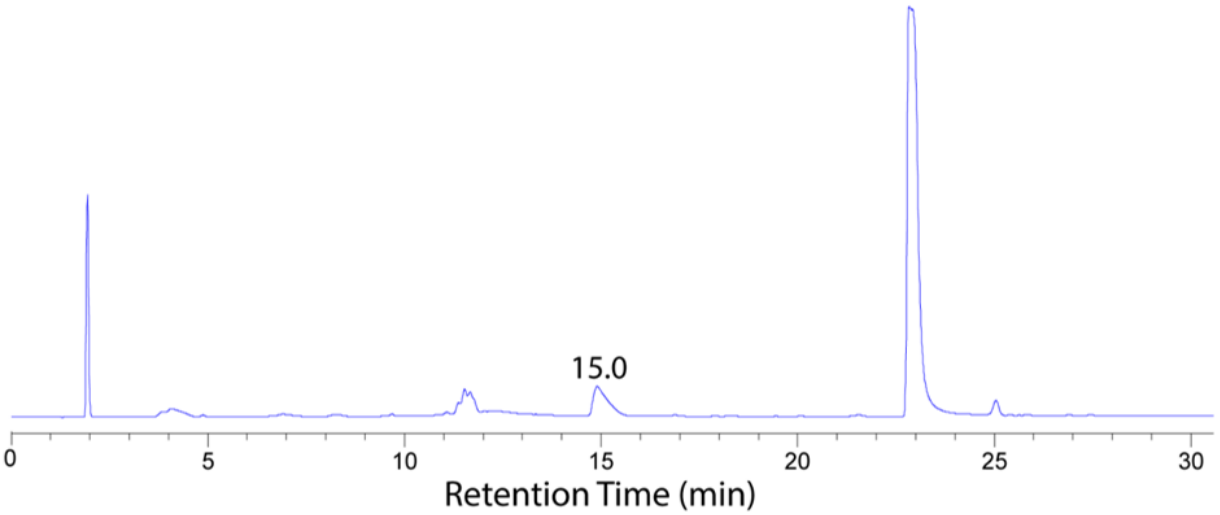

HRMS of H_2N-FRN(Triphenylamine)FG-CO_2H 10c

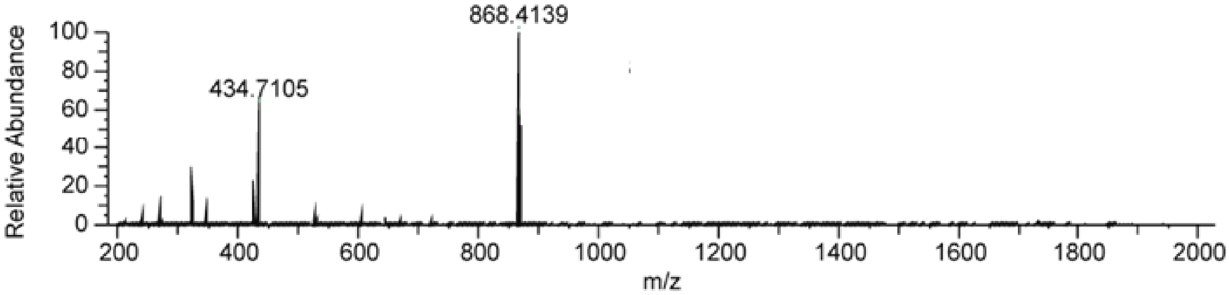

Synthesis of H_2N-FRN(Sulfonamide)FG-CO_2H 10d

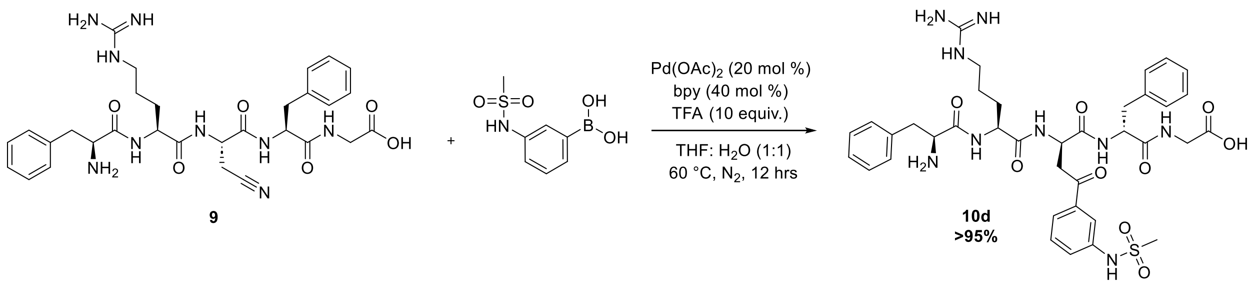

H_2N-FRN(Sulfonamide)FG-CO_2H **(10d)** was synthesized according to **GP-VIII**. Conversion was ascertained to be >95% using **HPLC Method A** based on remaining starting nitrile peptide.

H_2N-FRN(Sulfonamide)FG-CO_2H (10d): LCMS, *m/z* 794.3277 (calcd. $[M+H^+]$ = 794.3290), *m/z* 397.6675 (calcd. $[(M+2H^+)/2]$ = 397.6682). Purity: >99% (HPLC analysis at 220 nm). Retention time using **HPLC Method A:** 11.5 min.

Crude HPLC Trace for H_2N-FRN(Sulfonamide)FG-CO_2H 10d

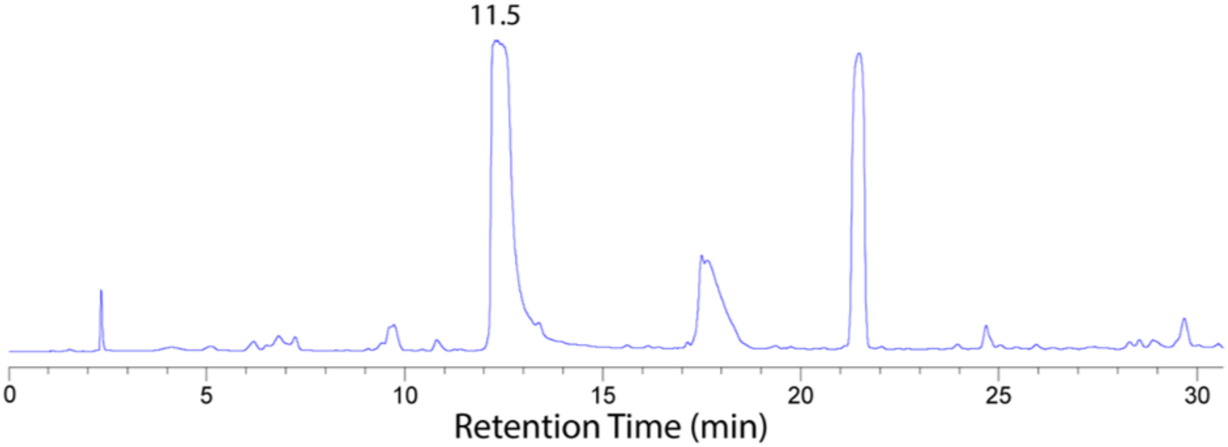

HRMS of H_2N-FRN(Sulfonamide)FG-CO_2H 10d

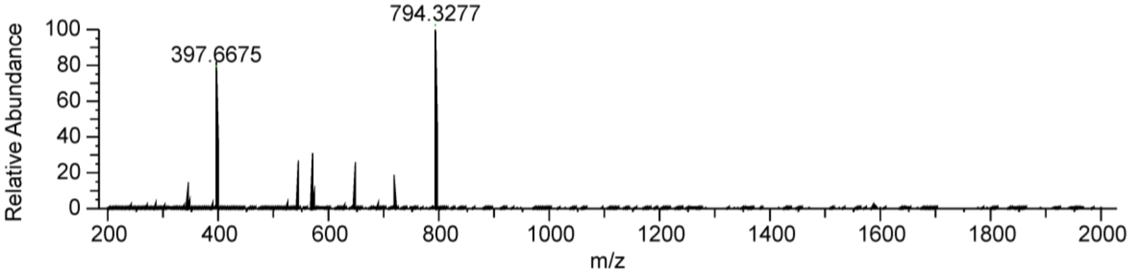

Synthesis of H_2N-FRN(3-nitrophenylketone)FG-CO_2H 10e

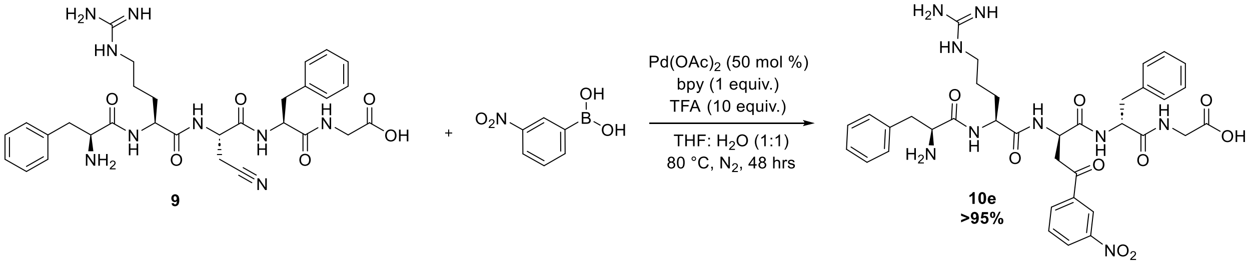

H_2N-FRN(3-nitrophenylketone)FG-CO_2H **(10e)** was synthesized according to **GP-IX**. Conversion was ascertained to be >95% using **HPLC Method A** based on remaining starting nitrile peptide.

H_2N-FRN(3-nitrophenylketone)FG-CO_2H (10e): LCMS, *m/z* 746.3134 (calcd. [M+H^+] = 746.3257), *m/z* 373.6599 (calcd. [(M+2H^+)/2] = 373.6664). Purity: >99% (HPLC analysis at 220 nm). Retention time using **HPLC Method A:** 11.3 min

Crude HPLC Trace for H_2N-FRN(3-nitrophenylketone)FG-CO_2H 10e

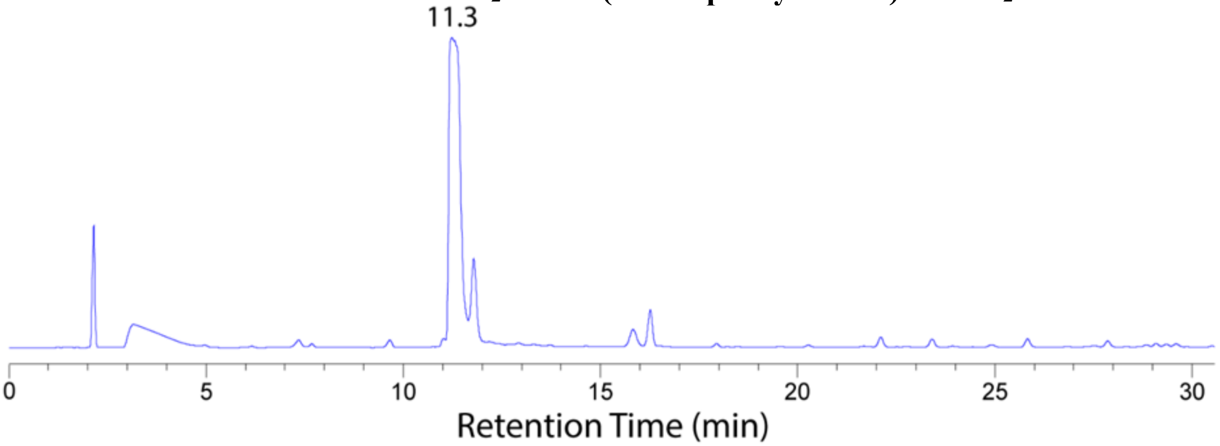

HRMS of H_2N-FRN(3-nitrophenylketone)FG-CO_2H 10e

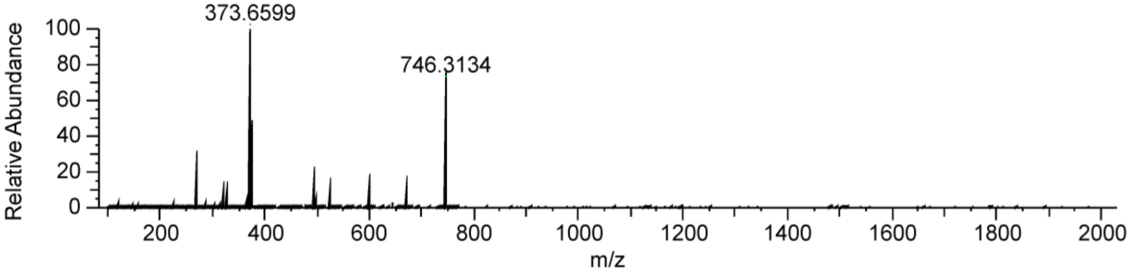

Synthesis of H_2N-FRN(4-acetylphenylketone)FG-CO_2H 10f

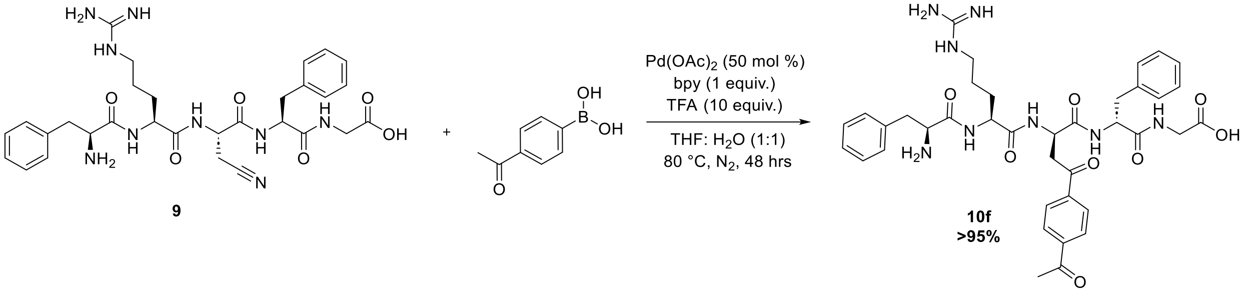

H_2N-FRN(4-acetylphenylketone)FG-CO_2H **(10f)** was synthesized according to **GP-IX**. Conversion was ascertained to be >95% using **HPLC Method A** based on remaining starting nitrile peptide.

H_2N-FRN(4-acetylphenylketone)FG-CO_2H (10f): LCMS, *m/z* 743.3508 (calcd. $[M+H^+]$ = 743.3511), *m/z* 372.1789 (calcd. $[(M+2H^+)/2]$ = 372.1792). Purity: >99% (HPLC analysis at 220 nm). Retention time using **HPLC Method A:** 11.7 min.

Crude HPLC Trace for H_2N-FRN(4-acetylphenylketone)FG-CO_2H 10f

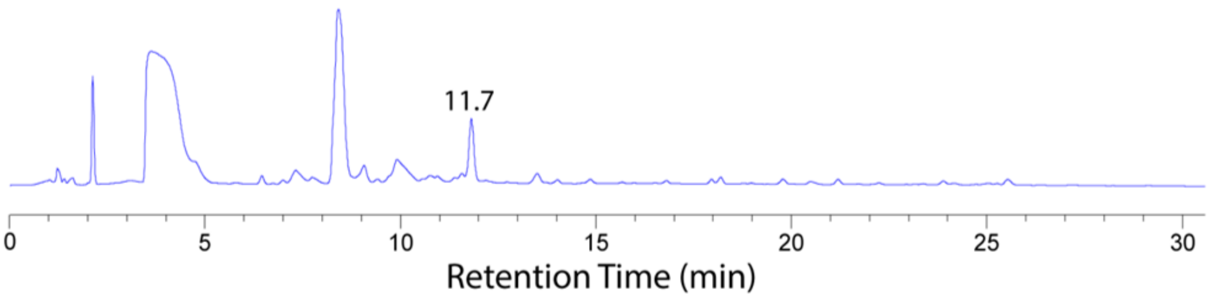

HRMS of H_2N-FRN(4-acetylphenylketone)FG-CO_2H 10f

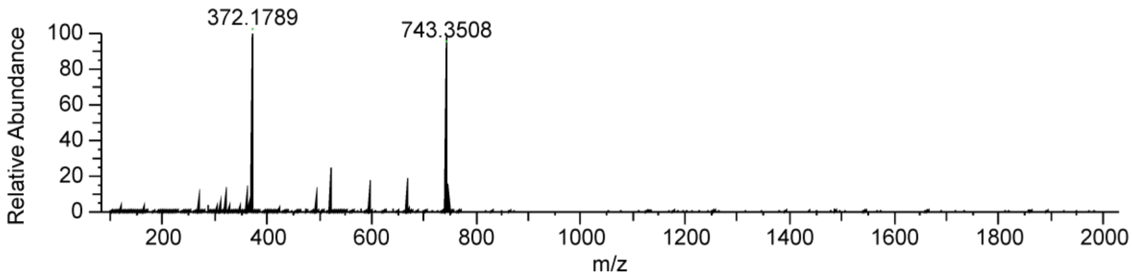

Synthesis of H_2N-FRN(2-trifluoromethylphenylketone)FG-CO_2H 10g

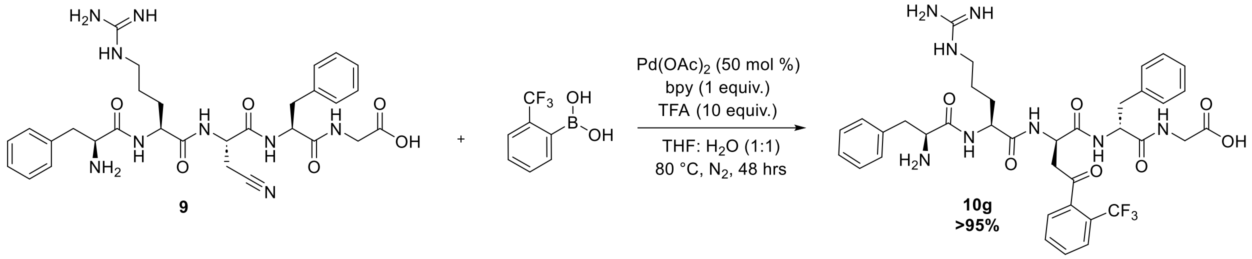

H_2N-FRN(2-trifluoromethylphenylketone)FG-CO_2H **(10g)** was synthesized according to **GP-IX**. Conversion was ascertained to be >95% using **HPLC Method A**.

H_2N-FRN(2-trifluoromethylphenylketone)FG-CO_2H (10g): LCMS, *m/z* 769.3094 (calcd. $[M+H^+]$ = 769.3280), *m/z* 385.1582 (calcd. $[(M+2H^+)/2]$ = 385.1676). Purity: >99% (HPLC analysis at 220 nm). Retention time using **HPLC Method A:** 11.0 min based on remaining starting nitrile peptide.

Crude HPLC Trace for H_2N-FRN(2-trifluoromethylphenylketone)FG-CO_2H 10g

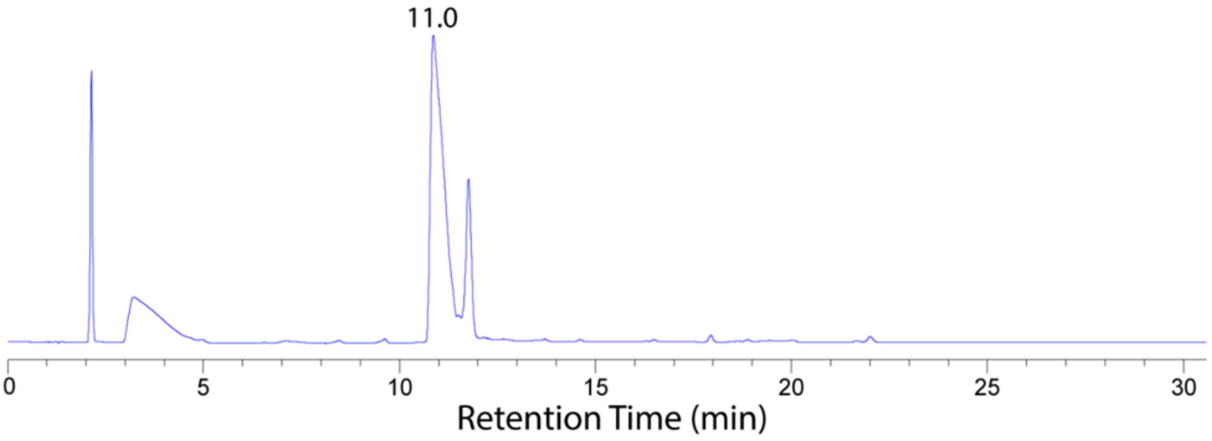

HRMS of H_2N-FRN(2-trifluoromethylphenylketone)FG-CO_2H 10g

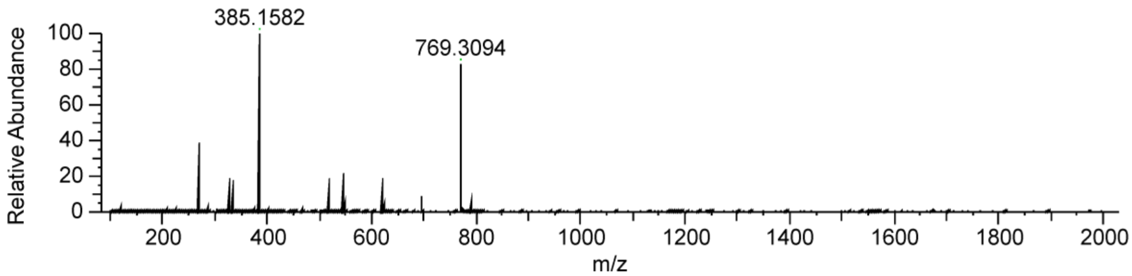

Synthesis of H_2N-FRN(3-Thiopheneketone)FG-CO_2H 10h

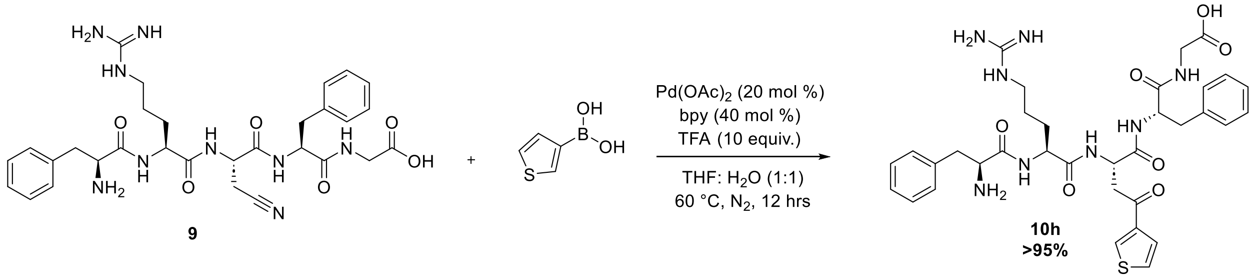

H_2N-FRN(3-Thiopheneketone)FG-CO_2H **(10h)** was synthesized according to **GP-VIII**. Conversion was ascertained to be >95% using **HPLC Method A** based on remaining starting nitrile peptide.

H_2N-FRN(3-Thiopheneketone)FG-CO_2H (10h): LCMS, *m/z* 707.2981 (calcd. $[M+H^+]$ = 707.2970), *m/z* 354.1529 (calcd. $[(M+2H^+)/2]$ = 354.1522). Purity: >99% (HPLC analysis at 220 nm). Retention time using **HPLC Method A:** 11.7 min.

Crude HPLC Trace for H_2N-FRN(3-Thiopheneketone)FG-CO_2H 10h

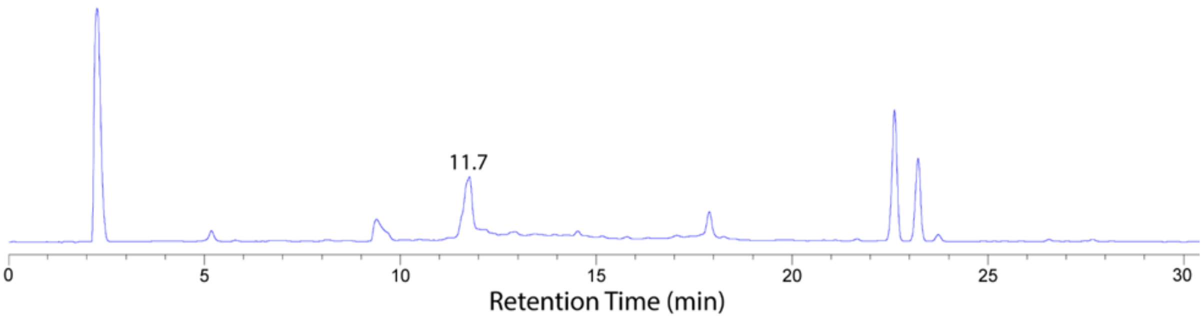

HRMS of H_2N-FRN(3-Thiopheneketone)FG-CO_2H 10h

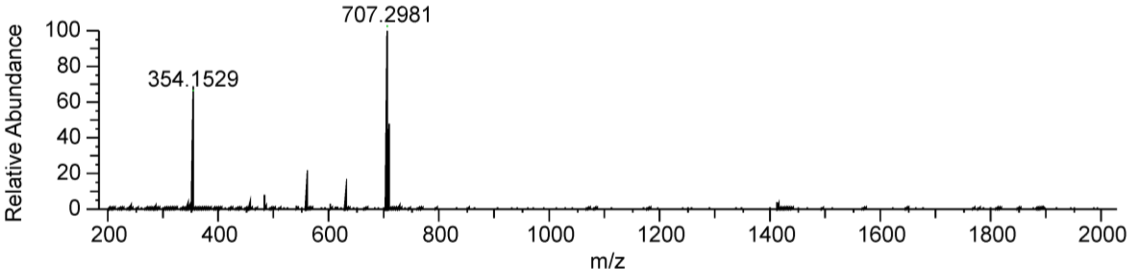

Synthesis of H_2N-FRN(Fluorenyl ketone)FG-CO_2H 10i

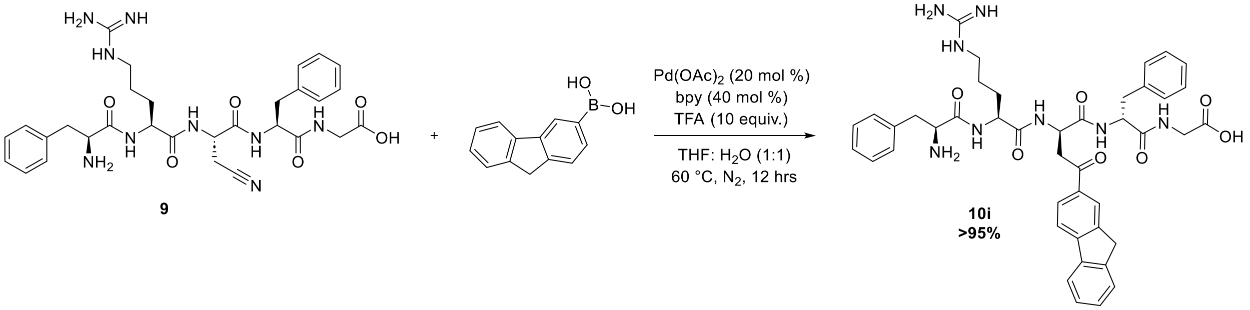

H_2N-FRN(Fluorenyl ketone)FG-CO_2H **(10i)** was synthesized according to **GP-VIII**. Conversion was ascertained to be >95% using **HPLC Method A**.

H_2N-FRN(Fluorenyl ketone)FG-CO_2H (10i): LCMS, *m/z* 789.3674 (calcd. $[M+H^+]$ = 789.3719). Purity: >99% (HPLC analysis at 220 nm). Retention time using **HPLC Method A:** 11.3 min.

Crude HPLC Trace for H2N-FRN(Fluorenyl ketone)FG-CO2H 10i

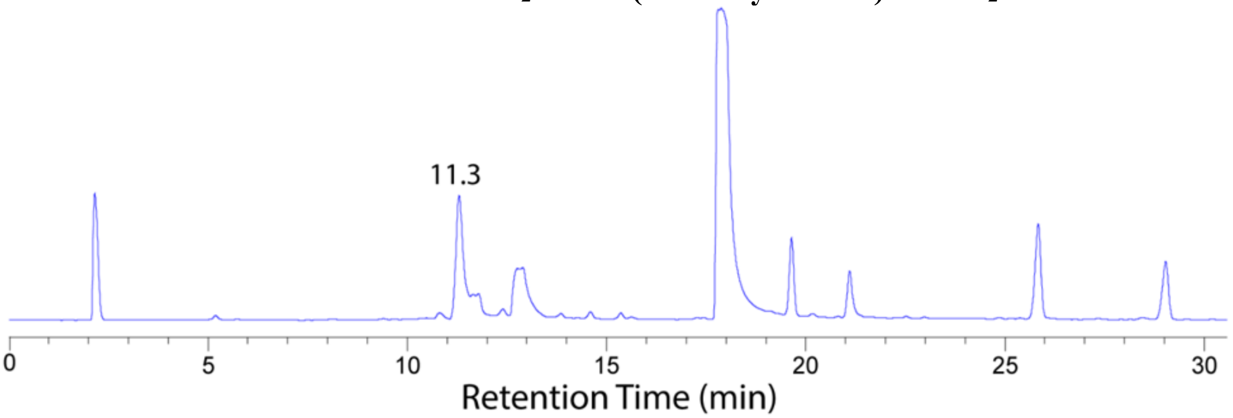

HRMS of H2N-FRN(Fluorenyl ketone)FG-CO2H 10i

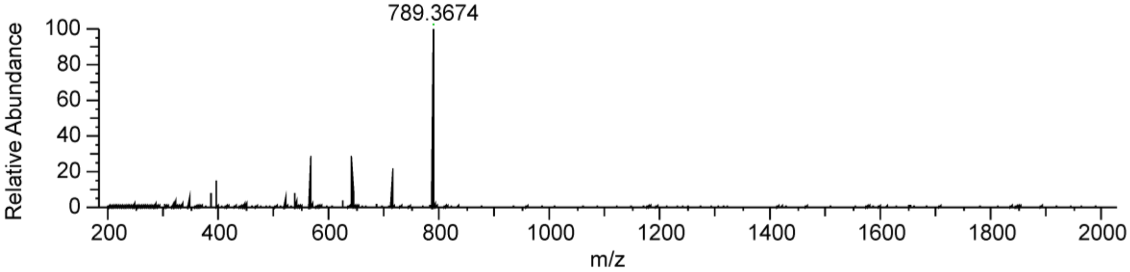

Synthesis of H2N-FRN(4-methylazide phenylketone)FG-CO2H 10j

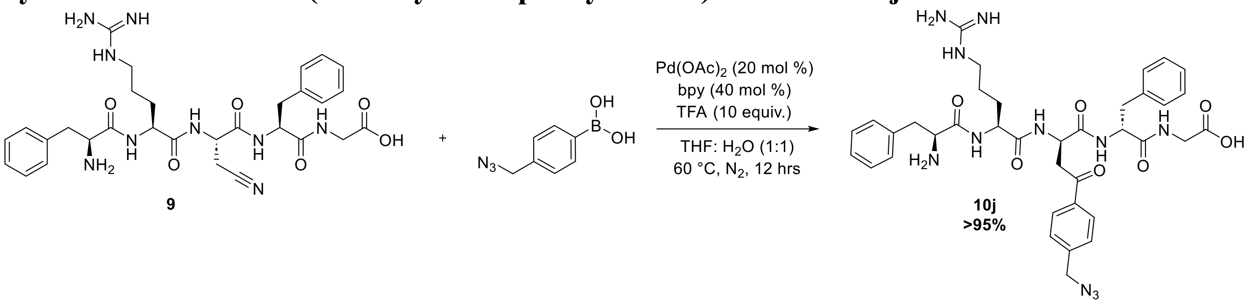

H2N-FRN(4-methylazide phenylketone)FG-CO2H **(10j)** was synthesized according to **GP-VIII**. Conversion was ascertained to be >95% using **HPLC Method A** based on remaining starting nitrile peptide.

H2N-FRN(4-methylazide phenylketone)FG-CO2H (10j): LCMS, *m/z* 756.3793 (calcd. [M+H$^+$] = 756.3576). Purity: >99% (HPLC analysis at 220 nm). Retention time using **HPLC Method A:** 12.1 min.

HPLC Trace of H_2N-FRN(4-methylazide phenylketone)FG-CO_2H 10j

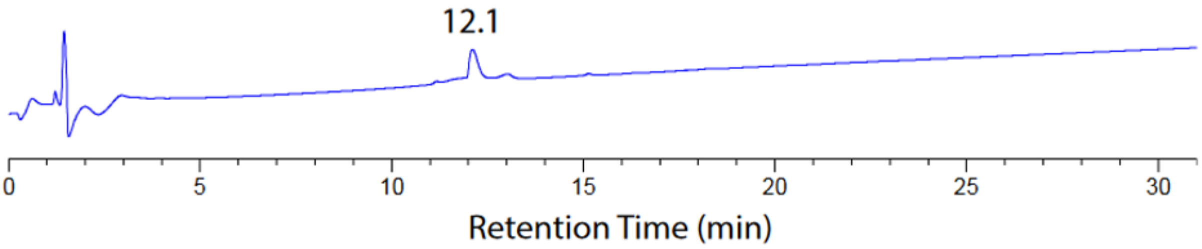

HMRS of H_2N-FRN(4-methylazide phenylketone)FG-CO_2H 10j

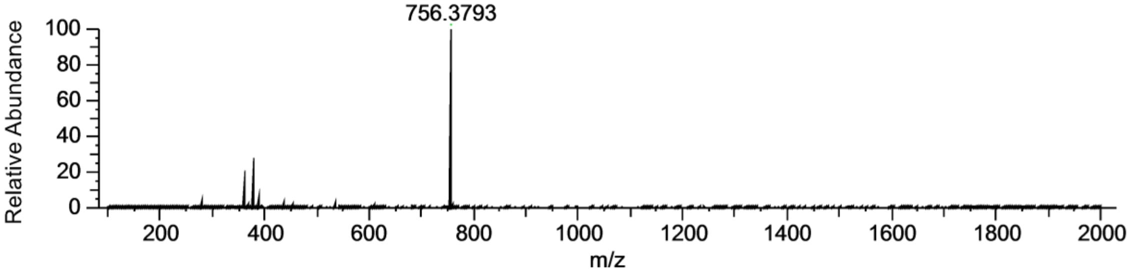

Supplementary Fig. 12: Synthesis of Azide Boronic Acid

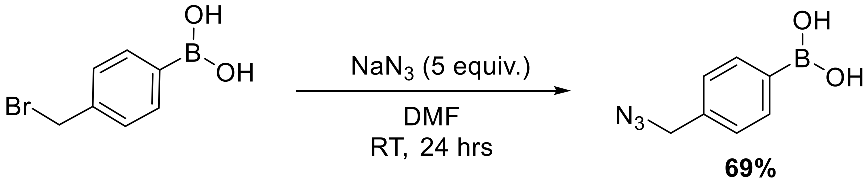

4-bromomethylphenylboronic (500 mg, 2.33 mmol, 1.0 equiv.) was dissolved in 10 mL of anhydrous DMF in a 100 mL RBF. Sodium Azide (756 mg, 11.6 mmol, 5.0 equiv.) was added to the reaction mixture, which was left to stir at room temperature for 24 hours. The reaction mixture was transferred into a separatory funnel, and the organic layer was extracted with EtOAc (30 mL) and washed with brine (3 x 50 mL). Next, the organic layer was dried over Na_2SO_4, and the solvent was evaporated to produce compound 4-azidomethylphenylboronic acid as a yellow-white solid (285 mg, 69% yield). Analytical TLC, 7:3 EtOAc:Hex eluent, $R_f = 0.55$. **^{1}H NMR** (400 MHz, $CDCl_3$): $\delta = 8.23$ (d, $J = 8.0$ Hz, 2H), 7.45 (d, $J = 8.0$ Hz, 2H), 4.42 (s, 2H) ppm. **^{13}C NMR** (101 MHz, $CDCl_3$): $\delta = 140.15$, 136.30, 127.79, 54.84 ppm.

^{1}H NMR of 4-azidomethylphenylboronic acid

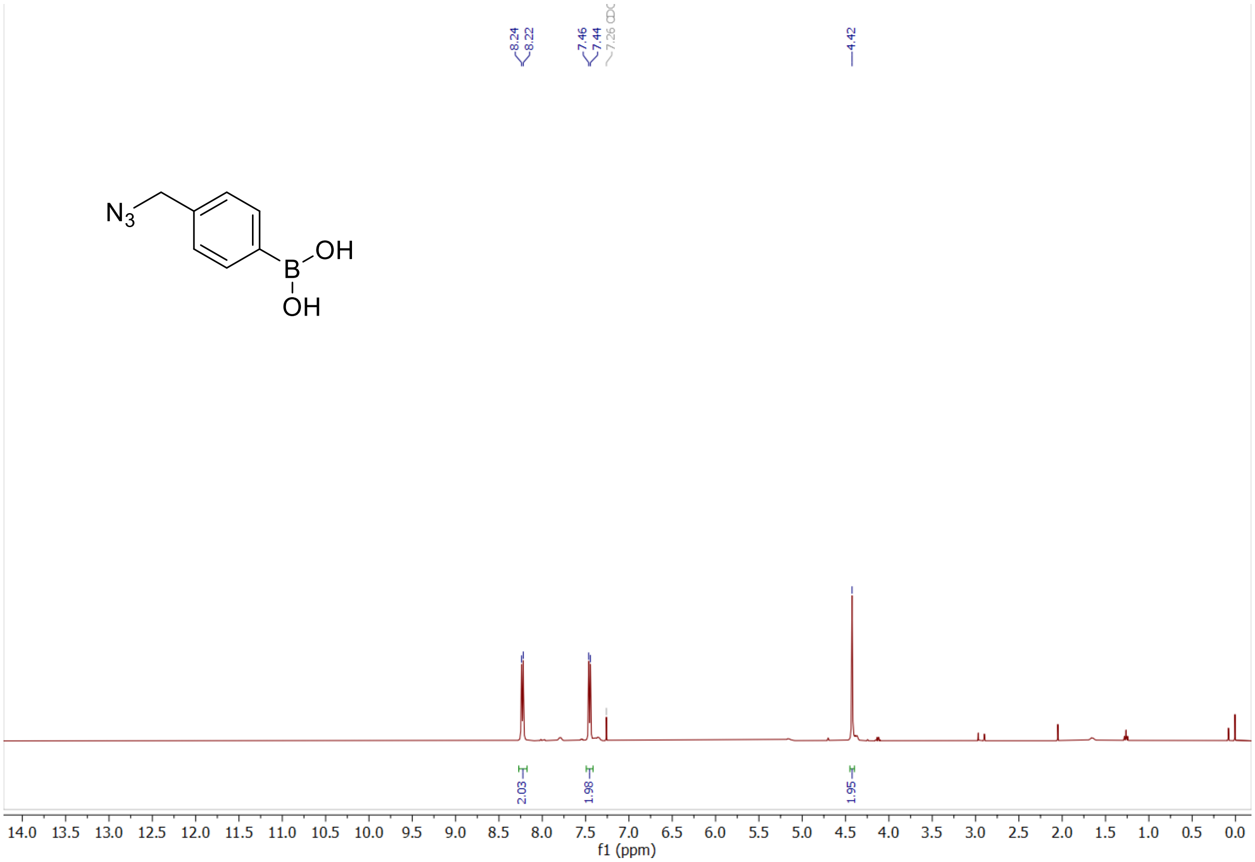

^{13}C NMR of 4-azidomethylphenylboronic acid

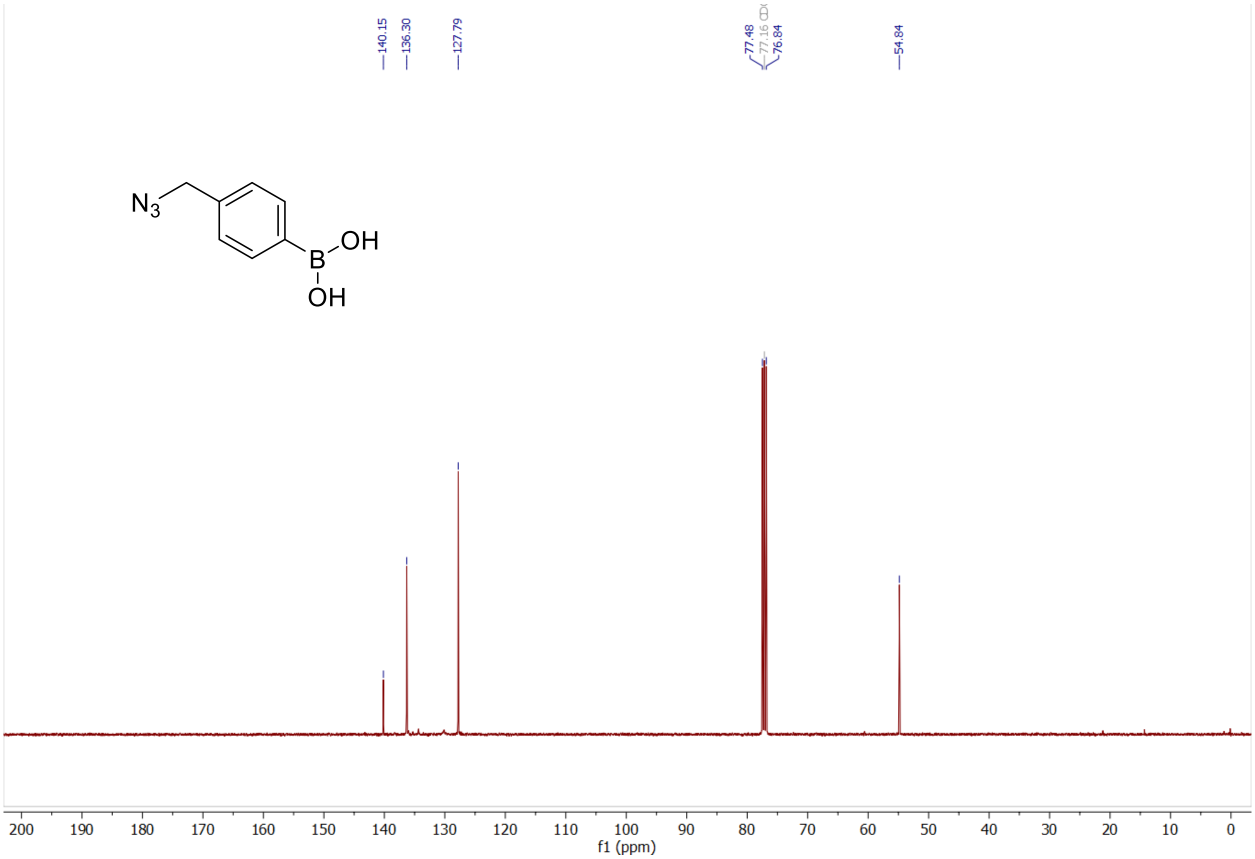

Supplementary Fig. 13: Decarbonylation During Carbometallation Reaction on Peptide

Our group previously observed selective ortho-deformylation under mild conditions during the nitrile boronic acid cross-coupling reaction.[3] We opted to explore whether these results would translate to a peptide system. Aligning with our previous exploration of this reaction, a meta-formyl boronic acid substrate retained the aldehyde moiety while two ortho-formyl boronic acid substrates underwent deformylation.

Deformylated Product Using 2-Formylphenylboronic Acid 10k

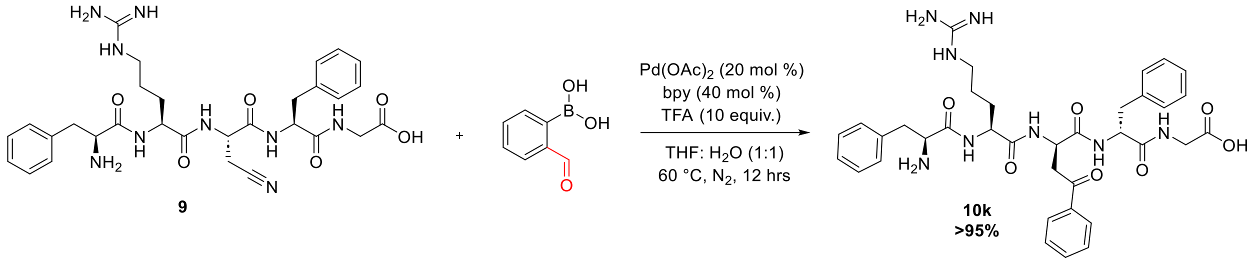

The deformylated peptide product **(10k)** was synthesized according to **GP-VIII**. Conversion was ascertained to be >95% using **HPLC Method A**.

Deformylated H_2N-FRN(phenylketone)FG-CO_2H Peptide Product (10k): LCMS, *m/z* 701.3401 (calcd. $[M+H^+]$ = 701.3406). Purity: >99% (HPLC analysis at 220 nm). Retention time using **HPLC Method A:** 11-12 min.

Crude HPLC Trace for Deformylated FRN(phenylketone)FG Peptide Product 10k

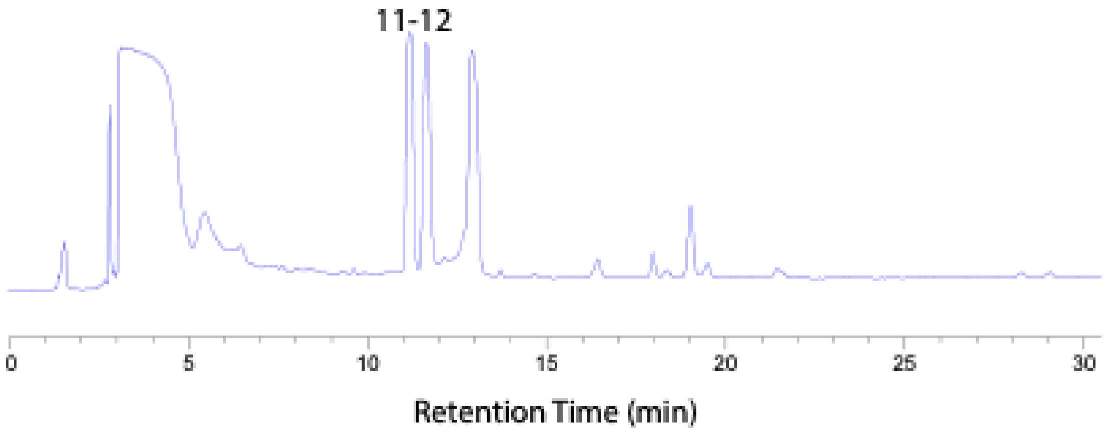

HRMS of Deformylated FRN(phenylketone)FG Peptide Product 10k

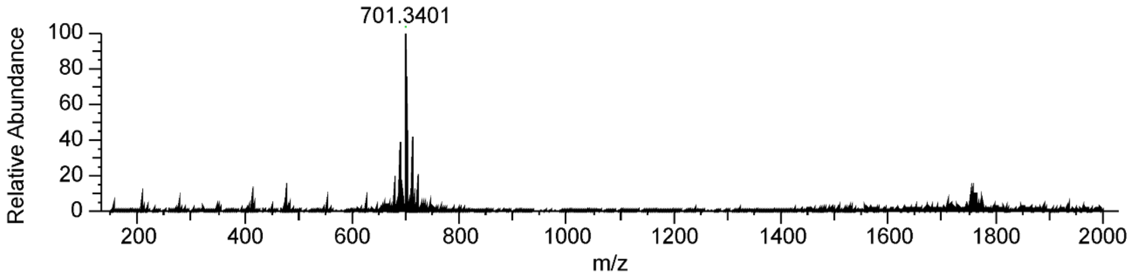

Deformylated Product Using (2-Formyl-5-methoxyphenyl)boronic acid 10l

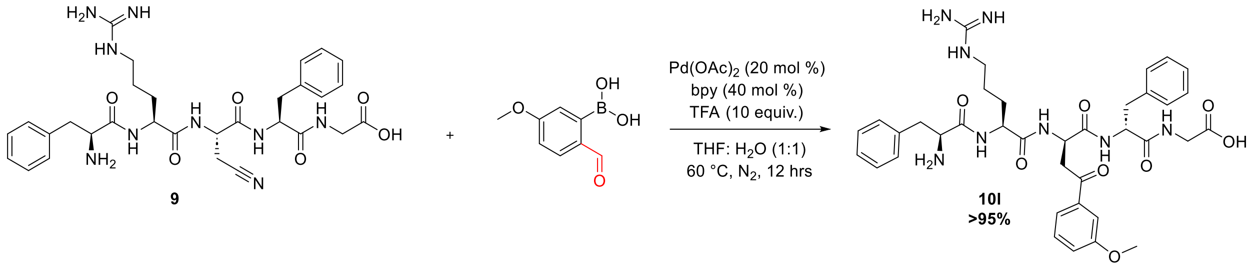

Deformylated H2N-FRN(3-methoxyphenylketone)FG-CO2H **(10l)** was synthesized according to **GP-VIII**. Conversion was ascertained to be >95% using **HPLC Method A**.

Deformylated H2N-FRN(3-methoxyphenylketone)FG-CO2H (10l): LCMS, *m/z* 731.3367 (calcd. [M+H$^+$] = 731.3511). Purity: >99% (HPLC analysis at 220 nm). Retention time using **HPLC Method A:** 11.5-12.0 min.

Crude HPLC Trace for Deformylated H2N-FRN(3-methoxyphenylketone)FG-CO2H 10l

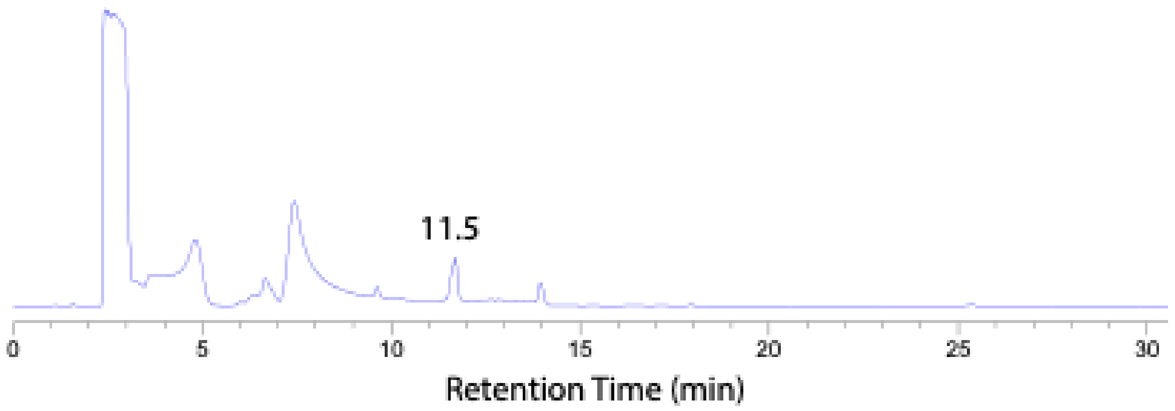

HRMS of Deformylated H2N-FRN(3-methoxyphenylketone)FG-CO2H 10l

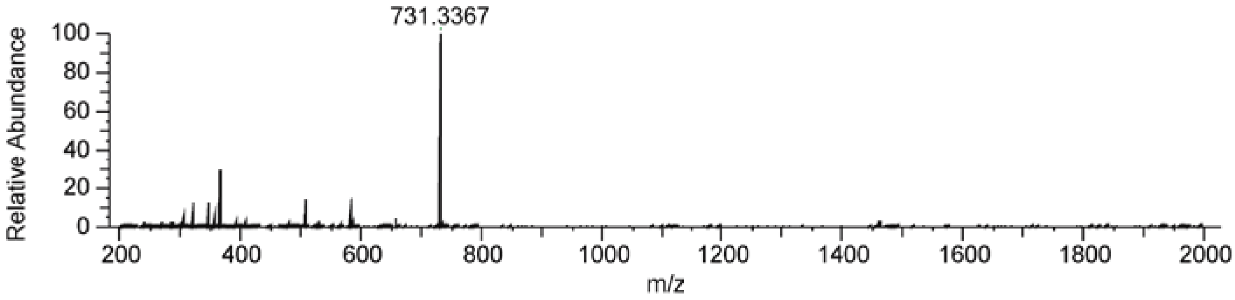

Product with Retained Aldehyde Using 3-Formylphenylboronic Acid 10m

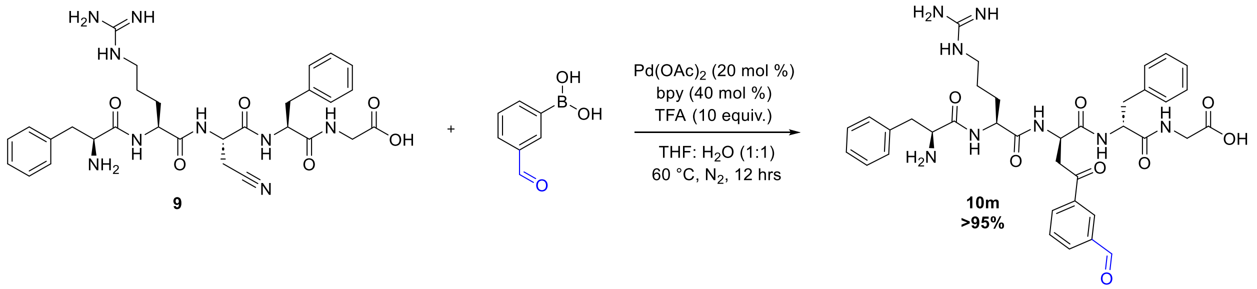

H_2N-FRN(3-formylphenylketone)FG-CO_2H **(10m)** was synthesized according to **GP-VIII**. Conversion was ascertained to be >95% using **HPLC Method A**.

H_2N-FRN(3-formylphenylketone)FG-CO_2H (10m): LCMS, *m/z* 729.3337 (calcd. $[M+H^+]$ = 729.3355). Purity: >99% (HPLC analysis at 220 nm). Retention time using **HPLC Method A:** 11.5-12.0 min.

Crude HPLC Trace for H_2N-FRN(3-formylphenylketone)FG-CO_2H 10m

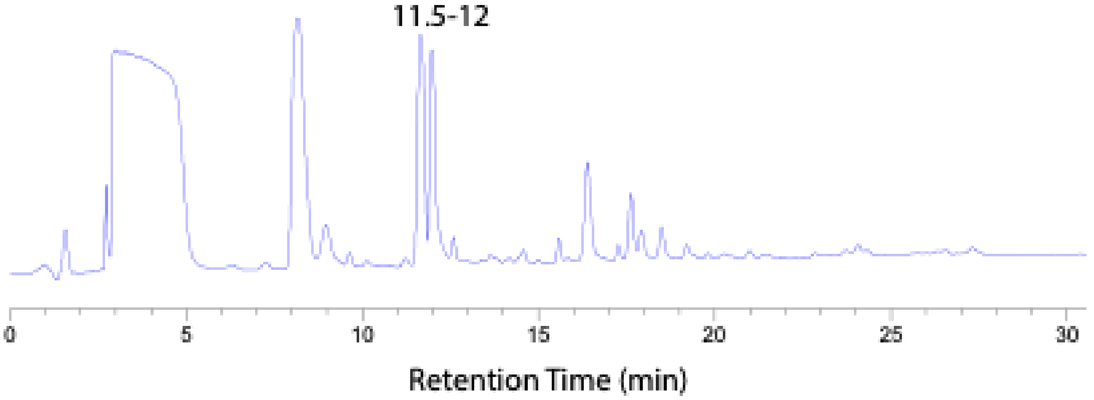

HRMS of H_2N-FRN(3-formylphenylketone)FG-CO_2H 10m

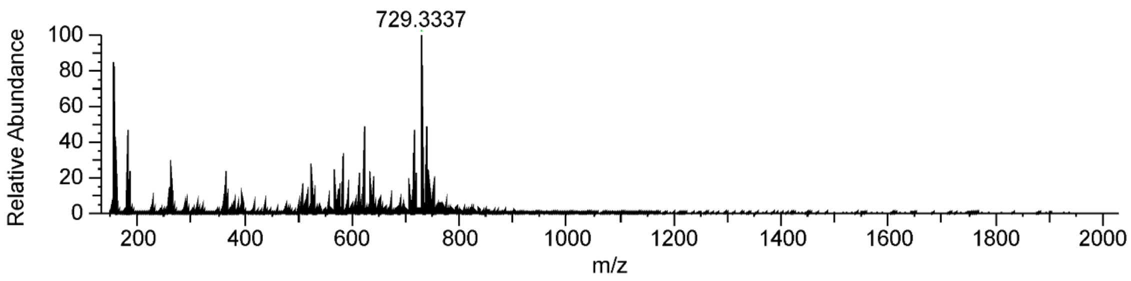

Supplementary Fig. 14: On-Resin Diversification of Asn/Gln to Nitrile and Aryl Ketones

Supplementary Fig. 14a: On-Resin Diversification Using Gln

Characterization of H_2N-AERQ-CO_2H 11

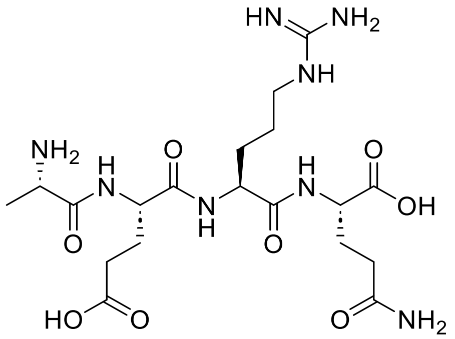

H_2N-AERQ-CO_2H (11): LCMS, *m/z* 503.2570 (calcd. $[M+H^+]$ = 503.2572), Purity: >99% (HPLC analysis at 220 nm). Retention time using **HPLC Method C**: 2.1 min.

HPLC Trace for H_2N-AERQ-CO_2H 11

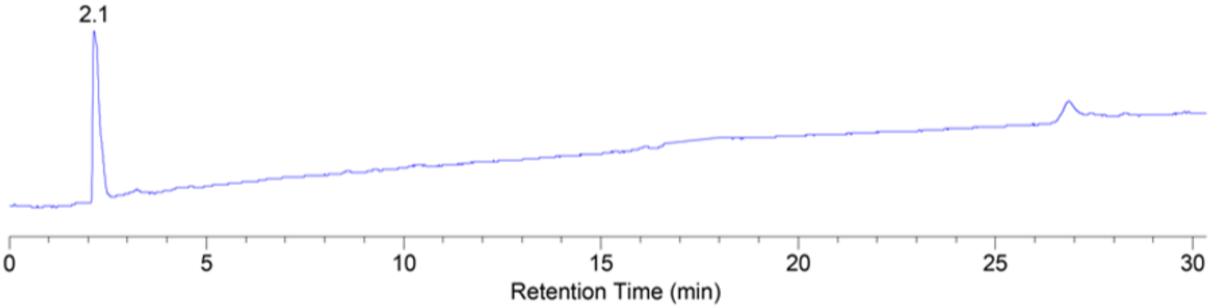

HRMS of H_2N-AERQ-CO_2H 11

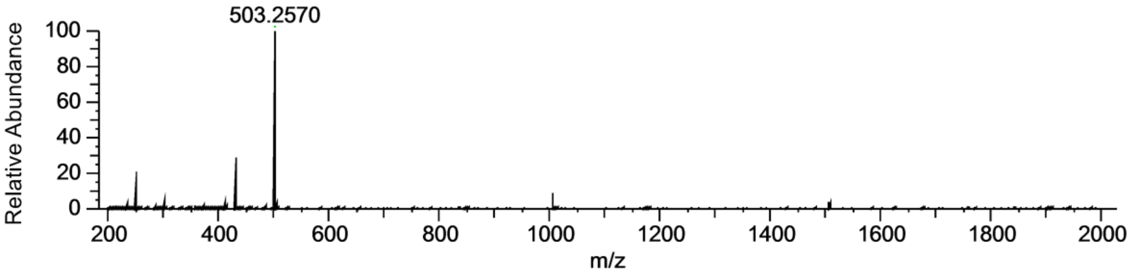

General Procedure for Dehydration of Asn/Gln on Resin (GP-X):

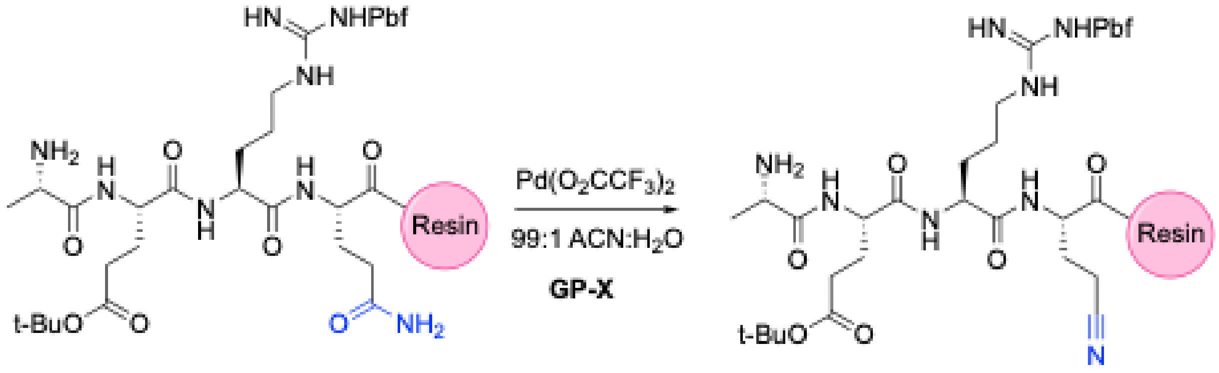

Gln-Wang preloaded resin (VWR catalog ID:101118-406) was used for peptide synthesis. Wang resin containing peptide, (1 g, 0.27 mmol) was added to a 25 mL SPPS tube. $Pd(O_2CCF_3)_2$ (89.8 mg, 0.27 mmol, 1 equiv.) was added to the tube, after which 10 mL ACN and 100 µL H_2O were added, forming a light brown solution. The tube was capped, wrapped in electrical tape, and placed on a mechanical shaker for 48 hours at RT. Following the reaction, the solvent was drained, and the resin was washed with DCM/DMF/MeOH 3x in order followed by DCM. Resin can be stored as is for subsequent reactions or cleaved using standard cleavage conditions to reveal nitrile peptide. Analysis via **HPLC Method D** was utilized to determine percent conversion to the nitrile isohypsic dehydration product based on remaining starting material.

Note: 48 hours is recommended to achieve quantitative conversion to nitrile product, allowing for easier purification as the amide (**11**) and nitrile (**12**) have similar retention times for this peptide.

Synthesis of H_2N-AERQ(Nitrile)-CO_2H 12

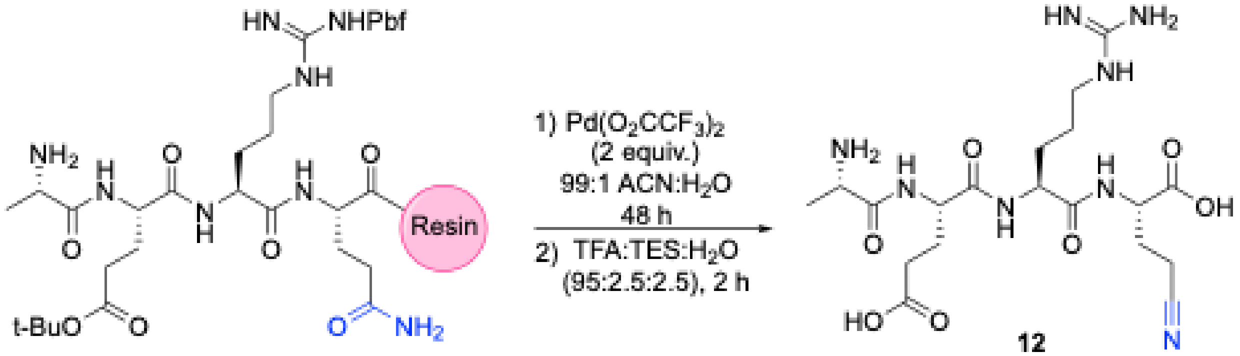

12

H_2N-AERQ(Nitrile)-CO_2H **(12)** was synthesized according to **GP-XI**. Conversion based on remaining amide was ascertained to be >95% using **HPLC Method C**.

H_2N-AERQ(Nitrile)-CO_2H (12): LCMS, *m/z* 485.2466 (calcd. $[M+H^+]$ = 485.2467), Purity: >99% (HPLC analysis at 220 nm). Retention time using **HPLC Method D:** 2.4 min.

Crude HPLC Trace for H2N-AERQ(Nitrile)-CO2H 12

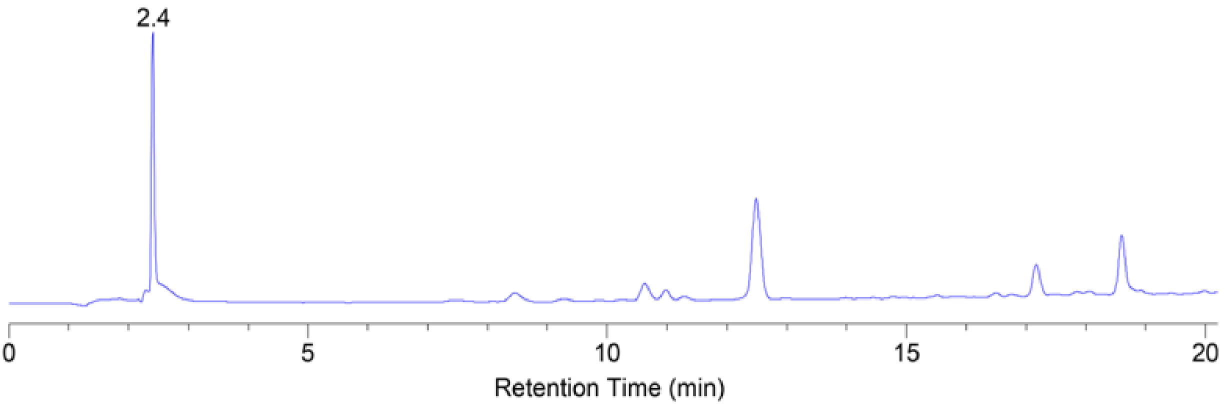

HRMS of H2N-AERQ(Nitrile)-CO2H 12

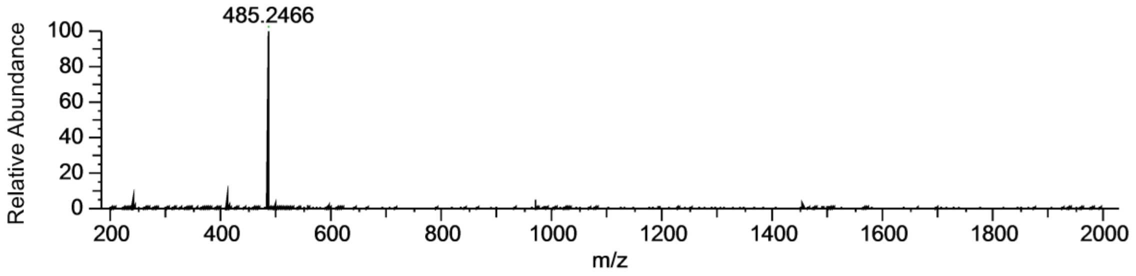

General Procedure for Nitrile Boronic Acid Carbometallation Reaction on Resin (GP-XI):

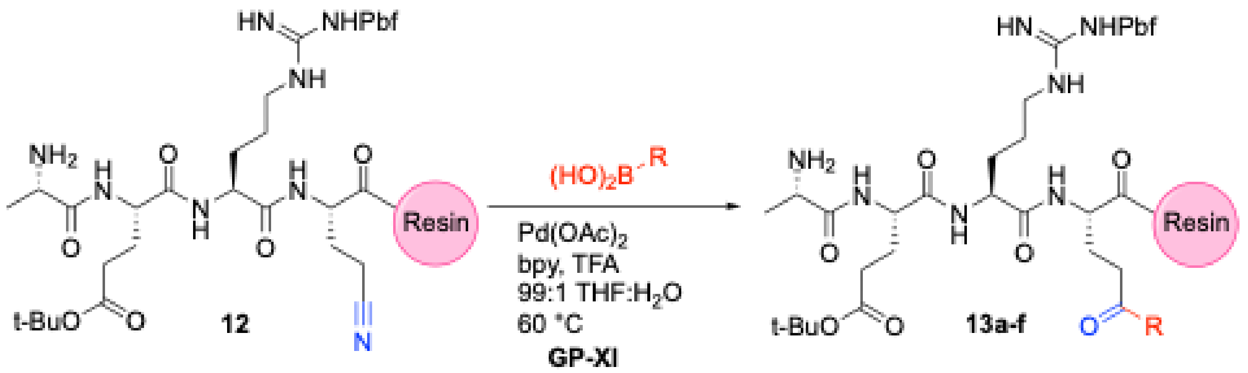

Nitrile peptide resin (100 mg, 0.027 mmol) was added to a 1" dram vial with a small stir bar. To the vial was added bipyridine ligand (9 mg, 0.054 mmol, 2 equiv.) and aryl boronic acid (0.162 mmol, 6 equiv.). Dry THF (3 mL) was added to the vial along with 20 µL H_2O and 10 µL trifluoroacetic acid (TFA). N_2 was bubbled through the solution for 5 minutes before $Pd(OAc)_2$ (12 mg, 0.054 mmol, 2 equiv.) was added. The reaction mixture was bubbled with N_2 for an additional 5 minutes, before the vial was sealed, wrapped in parafilm, and placed in an oil bath at 60 °C on low stirring for 48 hours. Resin was transferred to a 6 mL SPPS tube with a frit and washed with DCM/DMF/MeOH 3x through followed by DCM. Resin can be stored as is or cleaved using standard cleavage conditions to reveal aryl ketone peptide. Analysis via **HPLC Method B or D** was utilized to determine percent conversion to the aryl ketone peptide product

based on remaining starting nitrle peptide. **Note:** crude HPLC traces post SPPS cleavage are inherently messier from on-resin synthesis including truncated sequences, protecting group by-products, and coupling reagents as well as excess reagents for boronic acid coupling. Percent conversion is also an inherently flawed calculation in this instance due to the significant increase in molar absorptivity for aryl ketone peptides compared to the nitrile peptide.
Note: Low stirring is crucial to avoid destroying the integrity of the resin. Do not stir vigorously.

Synthesis of H_2N-AERQ(4-methoxyphenylketone)-CO_2H 13a

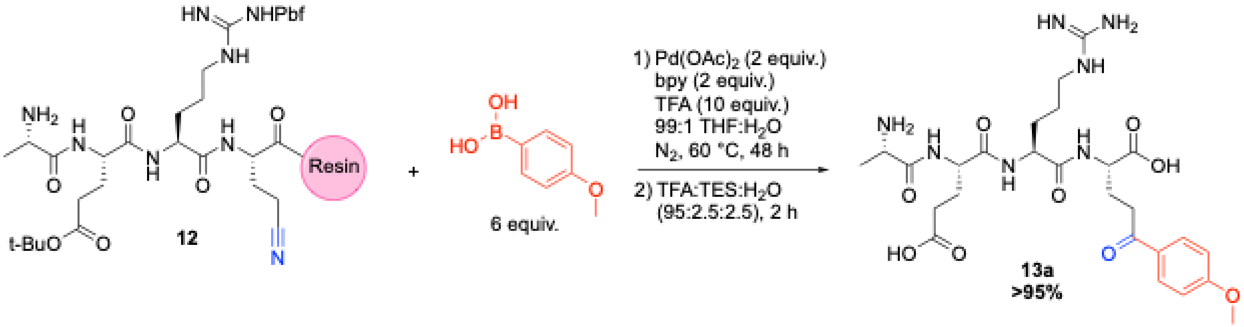

H_2N-AERQ(4-methoxyphenylketone)-CO_2H **(13a)** was synthesized according to **GP-XI**. Conversion was ascertained to be >95% using **HPLC Method D**.

H_2N-AERQ(4-methoxyphenylketone)-CO_2H (13a): LCMS, *m/z* 594.2882 (calcd. $[M+H^+]$ = 594.2882), Purity: >99% (HPLC analysis at 220 nm). Retention time using **HPLC Method D:** 9-12 min.

Crude HPLC Trace for H_2N-AERQ(4-methoxyphenylketone)-CO_2H 13a

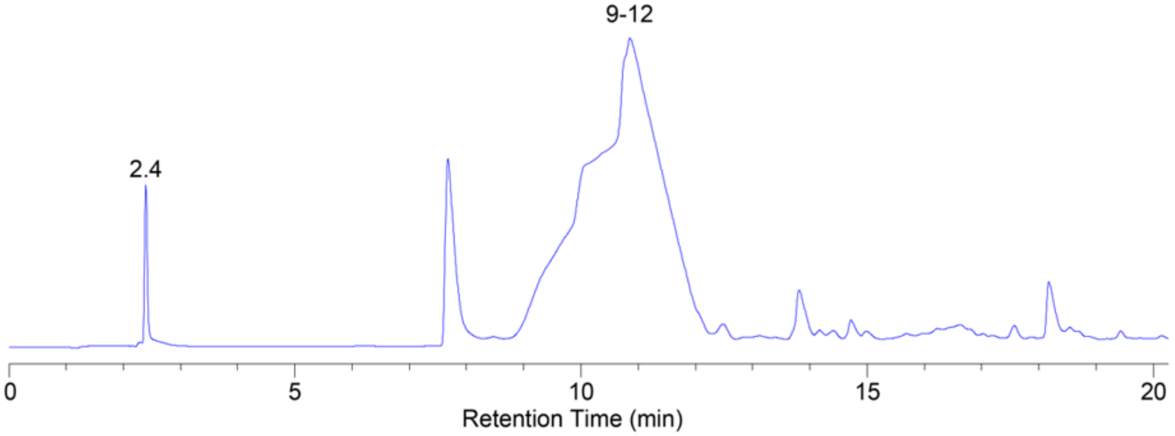

HRMS of H_2N-AERQ(4-methoxyphenylketone)-CO_2H 13a

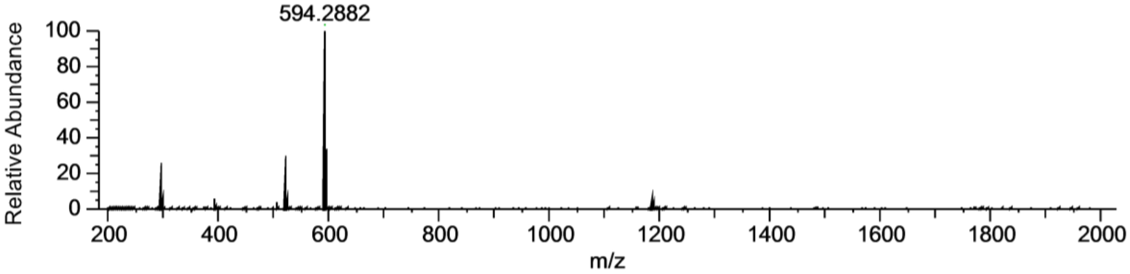

Synthesis of H_2N-AERQ(4-chlorophenylketone)-CO_2H 13b

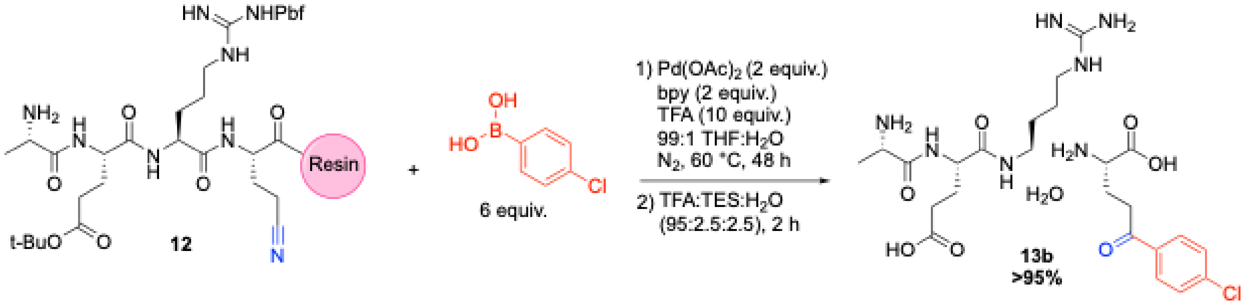

H_2N-AERQ(4-chlorophenylketone)-CO_2H **(13b)** was synthesized according to **GP-XI**. Conversion was ascertained to be >95% using **HPLC Method D**.

H_2N-AERQ(4-chlorophenylketone)-CO_2H (13b): LCMS, *m/z* 598.2386 (calcd. $[M+H^+]$ = 598.2387), Purity: >99% (HPLC analysis at 220 nm). Retention time using **HPLC Method D:** 10-14 min.

Crude HPLC Trace for H_2N-AERQ(4-chlorophenylketone)-CO_2H 13b

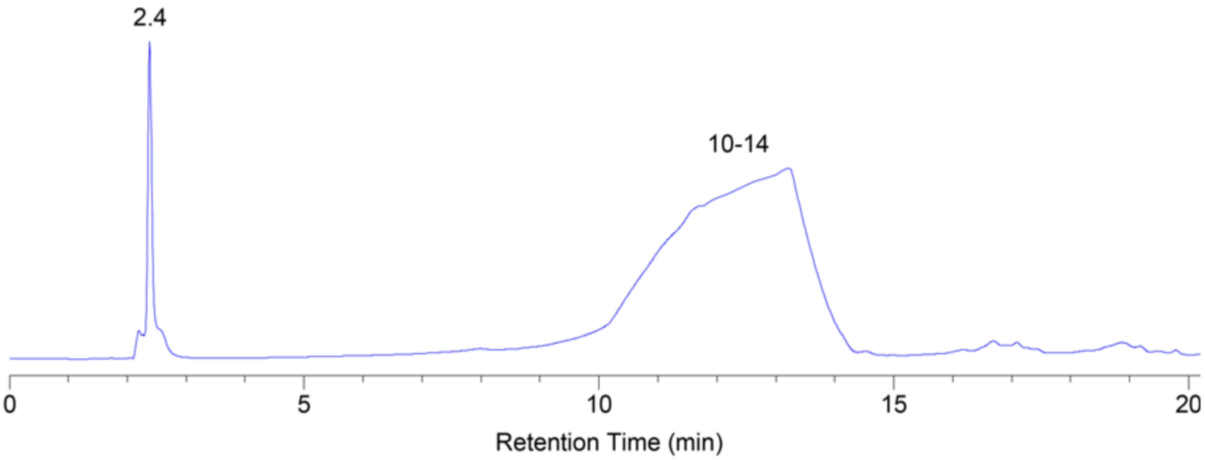

HRMS of H_2N-AERQ(4-chlorophenylketone)-CO_2H 13b

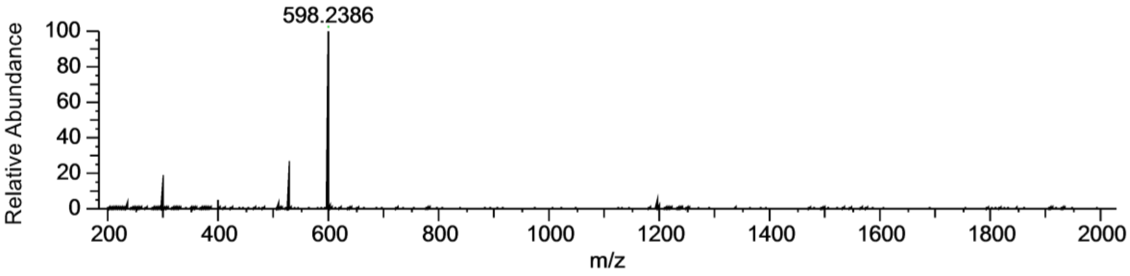

Synthesis of H_2N-AERQ(Triphenylamine)-CO_2H 13c

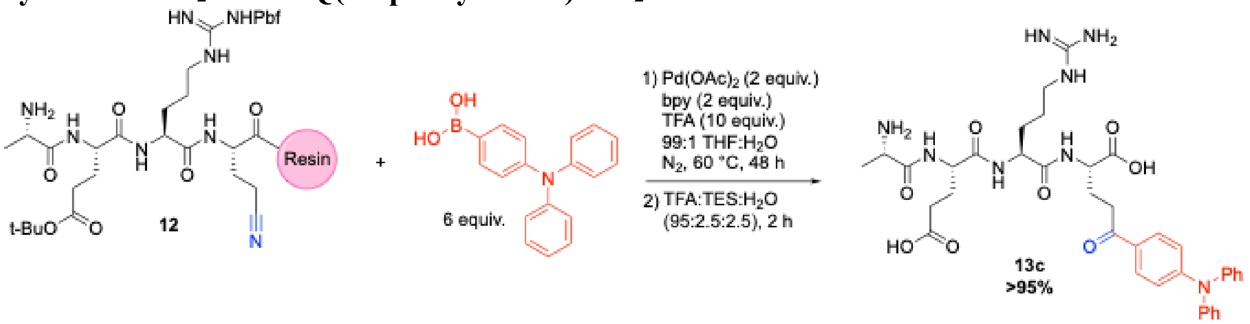

H_2N-AERQ(Triphenylamine)-CO_2H **(13c)** was synthesized according to **GP-XI**. Conversion was ascertained to be >95% using **HPLC Method A**.

H_2N-AERQ(Triphenylamine)-CO_2H (13c): LCMS, *m/z* 731.3510 (calcd. $[M+H^+]$ = 731.3511), *m/z* 366.1790 (calcd. $[(M+2H^+)/2]$ = 366.1792), Purity: >99% (HPLC analysis at 220 nm). Retention time using **HPLC Method A:** 16.8 min.

Crude HPLC Trace for H_2N-AERQ(Triphenylamine)-CO_2H 13c

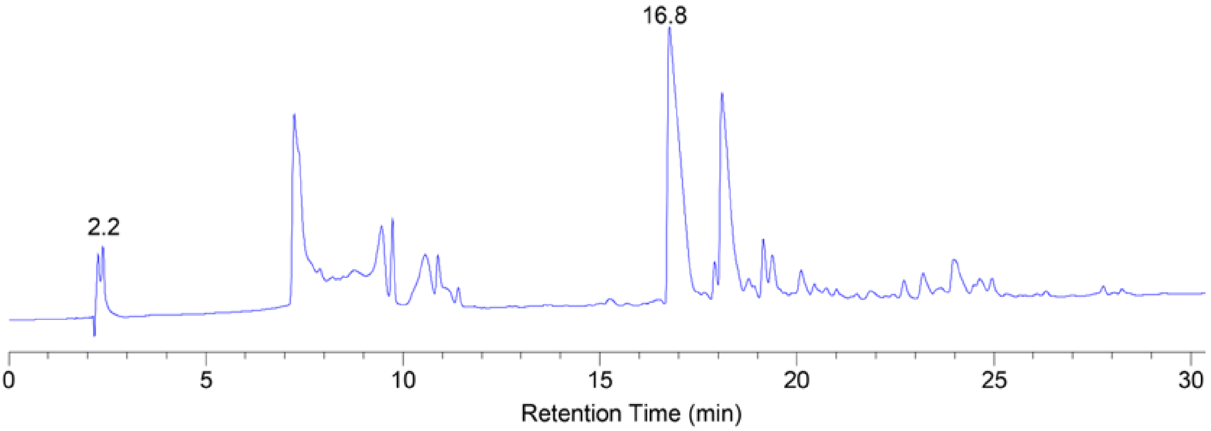

HRMS of H_2N-AERQ(Triphenylamine)-CO_2H 13c

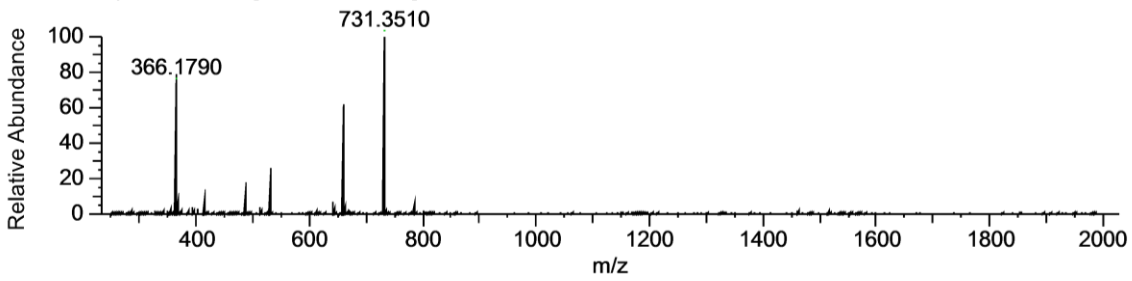

Synthesis of H_2N-AER(3-Thiophene)-CO_2H 13d

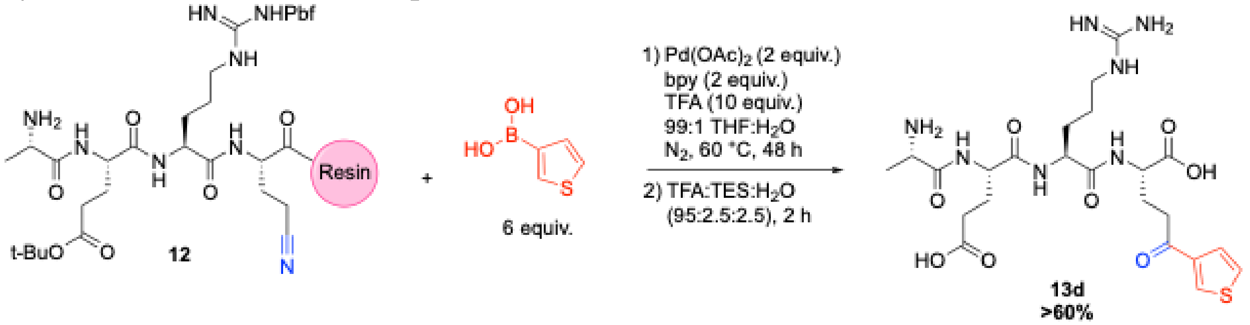

H_2N-AERQ(3-thiopheneketone)-CO_2H **(13d)** was synthesized according to **GP-XI**. Conversion was ascertained to be >60% using **HPLC Method A**.

H_2N-AERQ(3-thiopheneketone)-CO_2H (13d): LCMS, *m/z* 570.2341 (calcd. $[M+H^+]$ = 570.2341), Purity: >99% (HPLC analysis at 220 nm). Retention time using **HPLC Method A:** 8.6 min.

Crude HPLC Trace for H_2N-AERQ(3-thiopheneketone)-CO_2H 13d

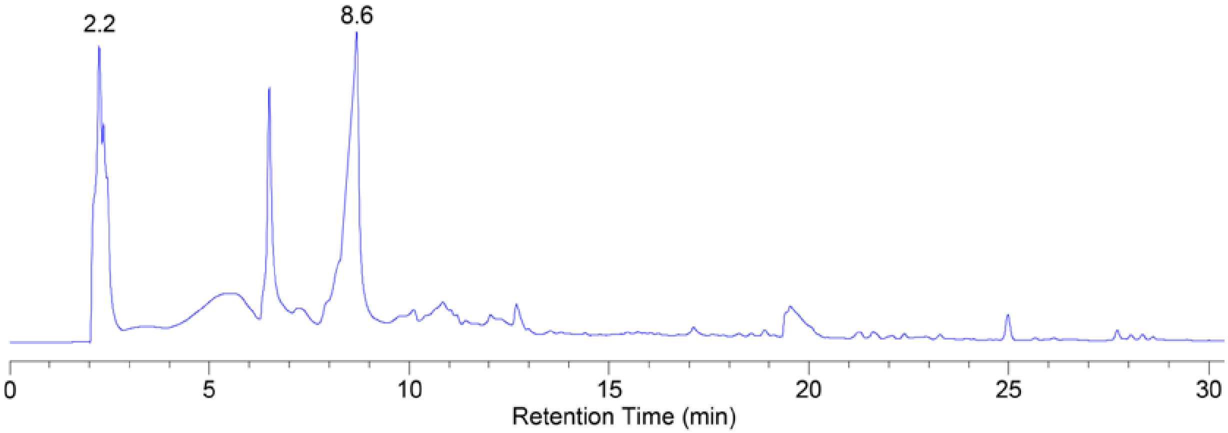

HRMS of H_2N-AERQ(3-thiopheneketone)-CO_2H 13d

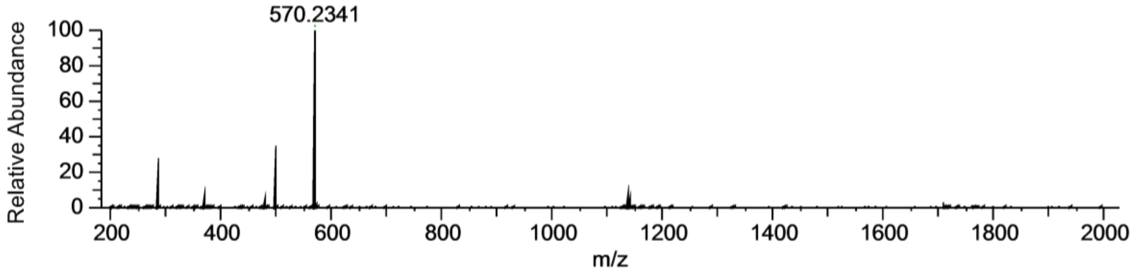

Synthesis of H_2N-AERQ(4-butylthiophenylketone)-CO_2H 13e

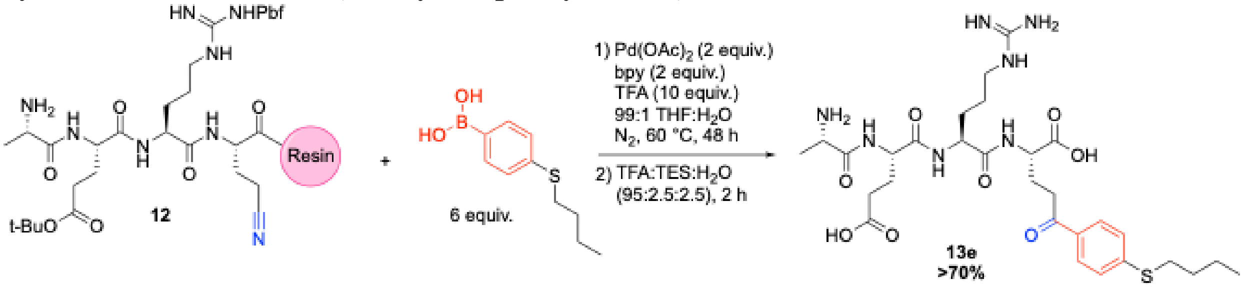

H_2N-AERQ(4-butylthiophenylketone)-CO_2H **(13e)** was synthesized according to **GP-XI**. Conversion was ascertained to be >70% using **HPLC Method D**.

H_2N-AERQ(4-butylthiophenylketone)-CO_2H (13e): LCMS, *m/z* 652.3120 (calcd. $[M+H^+]$ = 652.3123), Purity: >99% (HPLC analysis at 220 nm). Retention time using **HPLC Method D:** 15.1 min.

Crude HPLC Trace for H_2N-AERQ(4-butylthiophenylketone)-CO_2H 13e

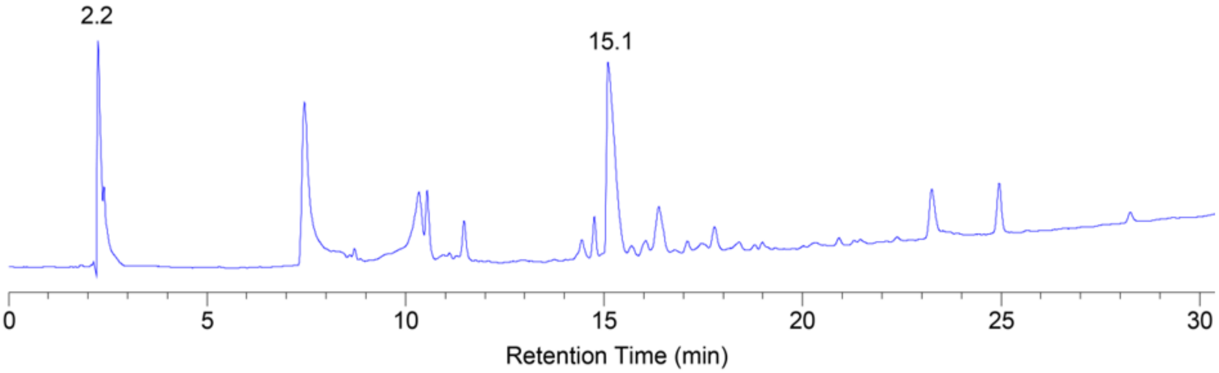

HRMS of H_2N-AERQ(4-butylthiophenylketone)-CO_2H 13e

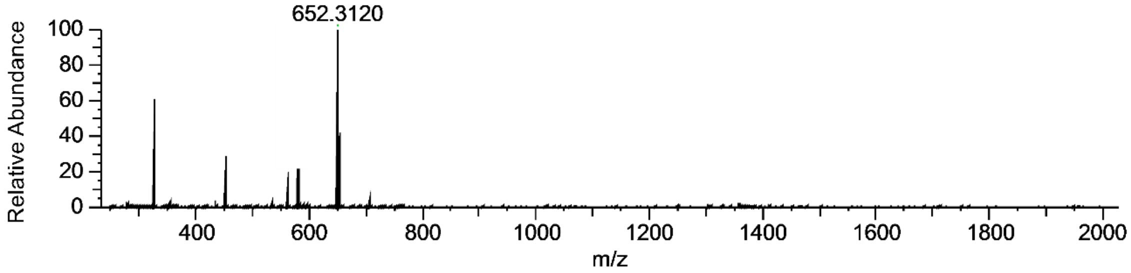

Synthesis of H_2N-AERQ(1,4-Benzodioxane ketone)-CO_2H 13f

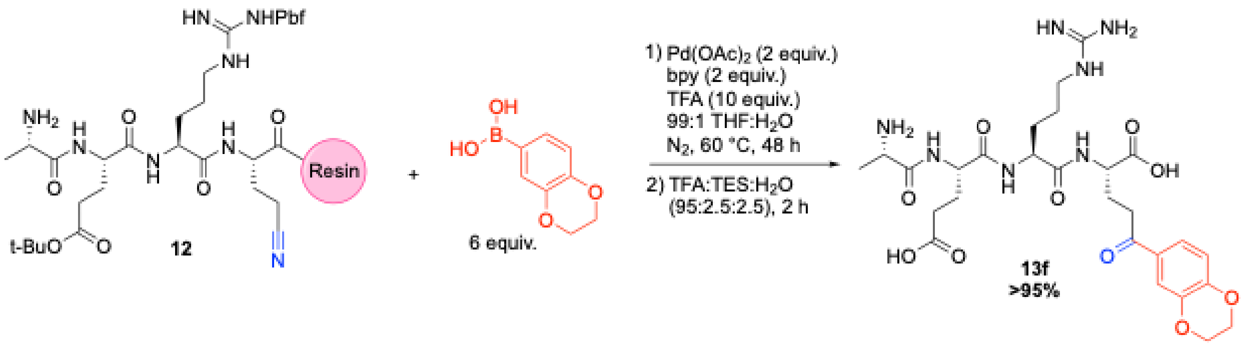

H_2N-AERQ(1,4-Benzodioxane ketone)-CO_2H **(13f)** was synthesized according to **GP-XI**. Conversion was ascertained to be >95% using **HPLC Method D**.

H_2N-AERQ(1,4-Benzodioxane)-CO_2H (13f): LCMS, *m/z* 622.2828 (calcd. $[M+H^+]$ = 622.2831), Purity: >99% (HPLC analysis at 220 nm). Retention time using **HPLC Method D:** 11.3 min.

Crude HPLC Trace for H_2N-AERQ(1,4-Benzodioxane ketone) CO_2H 13f

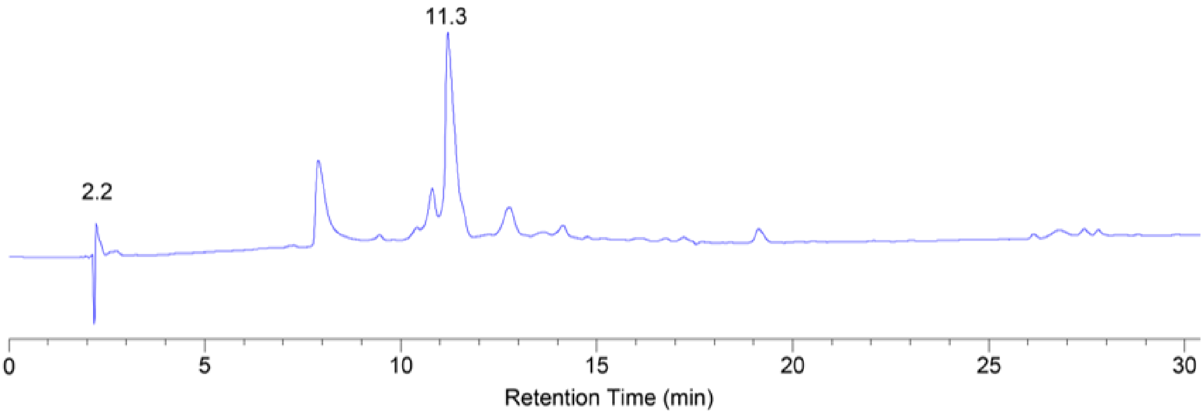

HRMS of H_2N-AERQ(1,4-Benzodioxane ketone)-CO_2H 13f

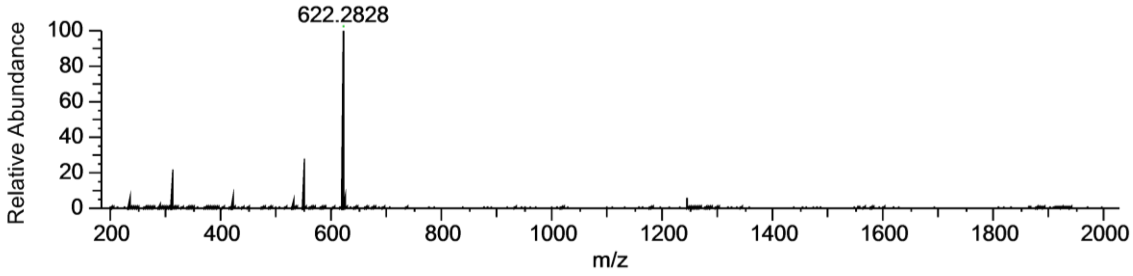

Supplementary Fig. 14b: Asn Peptide Diversification Using On-Resin Deprotection of Asn(Trt)

Procedure for On-Resin Deprotection Using Merrifield Resin:
Peptides were generated on Merrifield resin. The first amino acid coupling was achieved via the cesium salt (Gisin) method. The Fmoc-protected C-terminal amino acid was dissolved in methanol (5 mL/mmol) and water (0.5 mL/mmol). The solution was titrated to pH 7.0 with 20% aqueous cesium carbonate solution. The mixture was evaporated to dryness under reduced pressure. DMF (2.5 mL/mmol) was added and the residue was evaporated to dryness. A second portion of DMF (2.5 mL/mmol) was added and evaporated to dryness at 45 °C to ensure complete removal of water. The resulting cesium salt was dried under high vacuum overnight.

Merrifield resin was swelled in DMF (6-8 mL per gram of resin) and shaken for 24 h in an incushaker at 60 °C. The completely dry cesium salt (1.5-2.0 equivalents relative to resin chlorine substitution) was added to the swollen resin. The mixture was shaken at 60 °C for 48 hours. The resin was filtered and washed extensively with DMF (5x), 50% (v/v) aqueous DMF (5x), 50% (v/v) aqueous methanol (5x), and methanol (5x).

Fmoc deprotections were achieved with 10% piperidine (w/v) in 9:1 DMF:EtOH for 10 minutes and subsequent amino acid couplings were completed with Fmoc-protected amino acid (5 equiv.) Oxyma Pure (5 equiv.) and DIEA (5 equiv.) in DMF for 15 minutes. Resin was washed with DMF (5x) between each step.

Asn(Trt) deprotection was accomplished via treatment with 50% TFA in DCM (10 mL per gram of resin) for 1-2 hours at room temperature on a wrist shaker. The resin was drained and washed extensively with DCM (5×), 10% DIEA in DMF (2×), DMF (5×), DCM (3×), and methanol (2×). The resin was dried under vacuum. Note: Minor resin cleavage is likely under these conditions.

The cleavage cocktail was prepared by mixing TMSOTf (1.8 mL), TFA (7.0 mL), and triethylsilane (TES, 1.2 mL) per gram of resin. The mixture was cooled in an ice bath. The dried resin was added to the cocktail and stirred for 2 hours. The resin was filtered out and the filtrate was concentrated before the peptide was precipitated with ~10 volumes of cold ether. Precipitate was gathered, dried, and analyzed via LC-MS.

Synthesis of H_2N-LFANFG-CO_2H S4

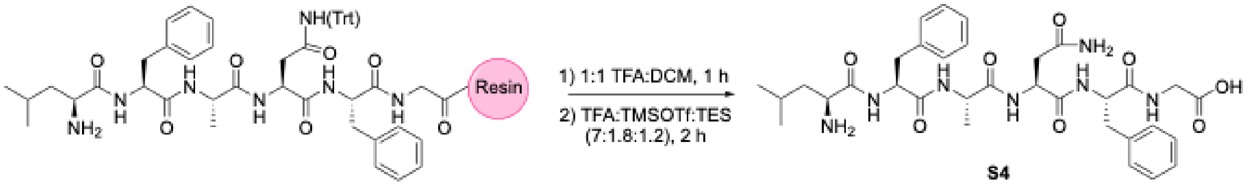

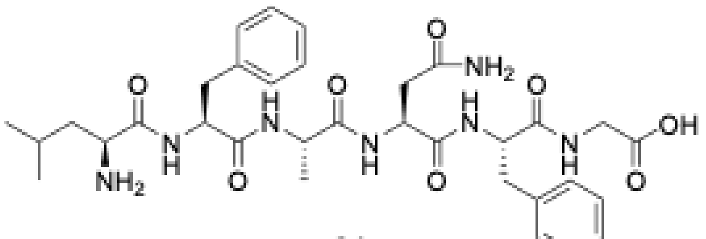

S4

H_2N-LFANFG-CO_2H (S4): LCMS, *m/z* 668.3423 (calcd. $[M+H^+]$ = 668.3402), *m/z* 334.6750 (calcd. $[(M+2H^+)/2]$ = 334.6738), Purity: >99% (HPLC analysis at 220 nm). Retention time using **HPLC Method D:** 15.9 min.

HPLC Trace for H_2N-LFANFG-CO_2H S4

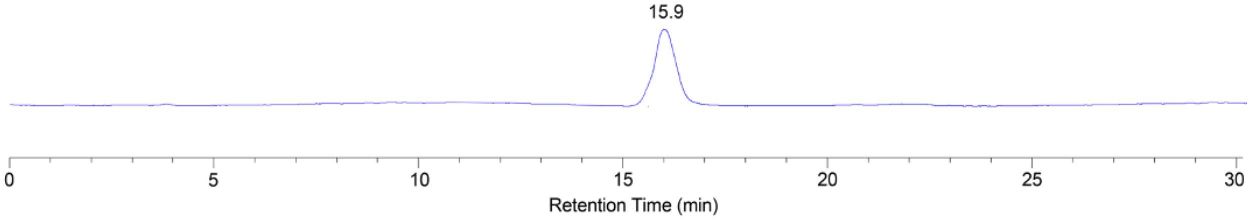

HRMS of H_2N-LFANFG-CO_2H S4

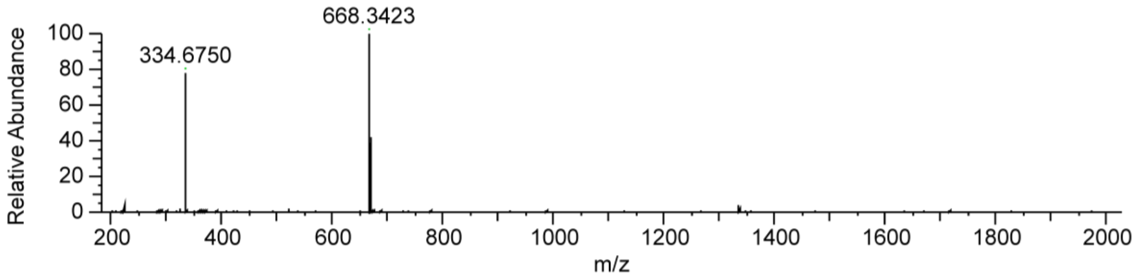

Generation of Nitrile on Resin for Peptide H_2N-LFAN(Nitrile)FG-CO_2H S5

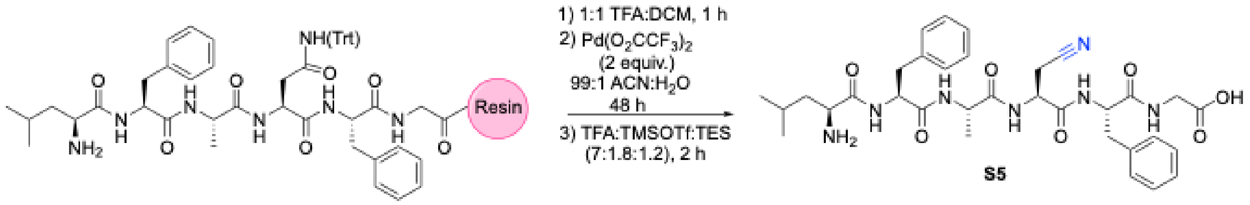

H_2N-LFAN(Nitrile)FG-CO_2H (**S5**) was synthesized according to **GP-X**. Conversion was ascertained to be >95% using **HPLC Method D**.

H_2N-LFAN(Nitrile)FG-CO_2H (S5): LCMS, *m/z* 650.3312 (calcd. $[M+H^+]$ = 650.3297), Purity: >99% (HPLC analysis at 220 nm). Retention time using **HPLC Method D:** 19.3 min.

HPLC Trace for H_2N-LFAN(Nitrile)FG-CO_2H S5

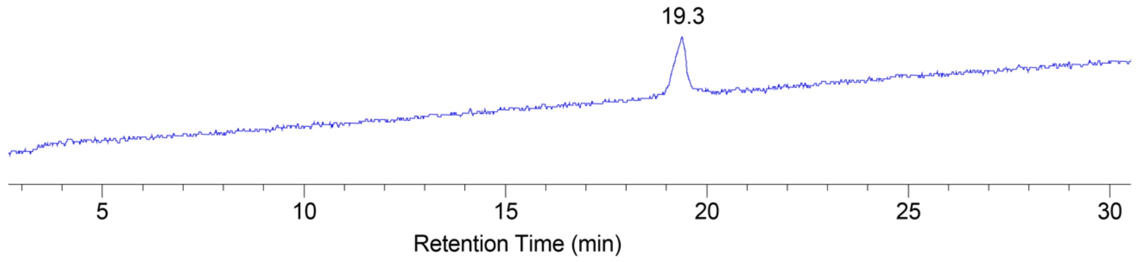

HRMS of H_2N-LFAN(Nitrile)FG-CO_2H S5

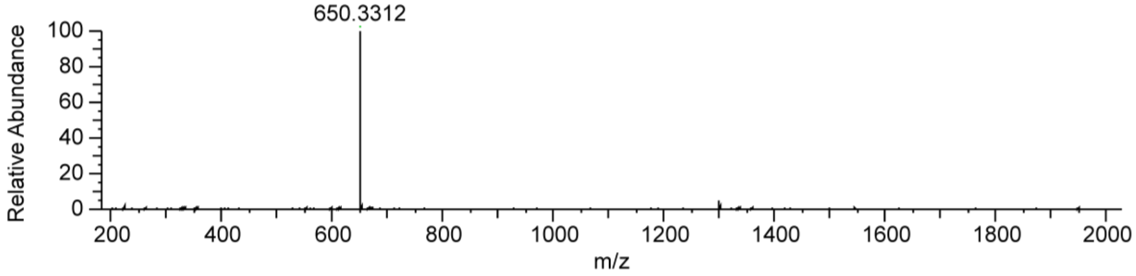

Generation of Aryl Ketone on Resin for Peptide H_2N-LFAN(4-methoxyphenylketone)FG-CO_2H S6

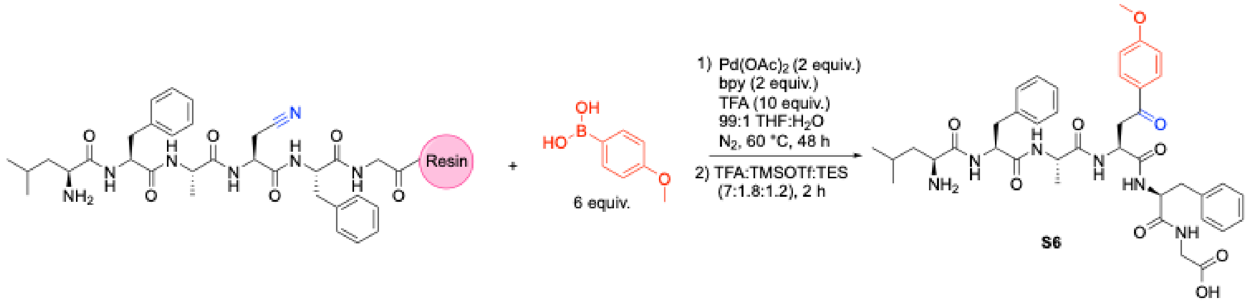

H_2N-LFAN(4-methoxyphenylketone)FG-CO_2H (**S6**) was synthesized according to **GP-XI**.

H_2N-LFAN(4-methoxyphenylketone)FG-CO_2H (S6): LCMS, *m/z* 759.3715 (calcd. $[M+H^+]$ = 759.3712), *m/z* 380.1895 (calcd. $[(M+2H^+)/2]$ = 380.1893), Purity: >99% (HPLC analysis at 220 nm). Retention time using **HPLC Method D:** 29.2 min.

HPLC Trace for H_2N-LFAN(4-methoxyphenylketone)FG-CO_2H S6

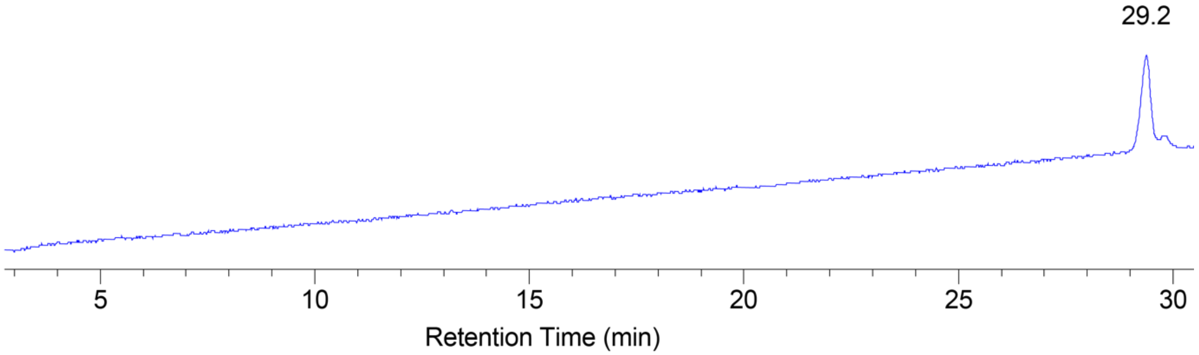

HRMS of H_2N-LFAN(4-methoxyphenylketone)FG-CO_2H S6

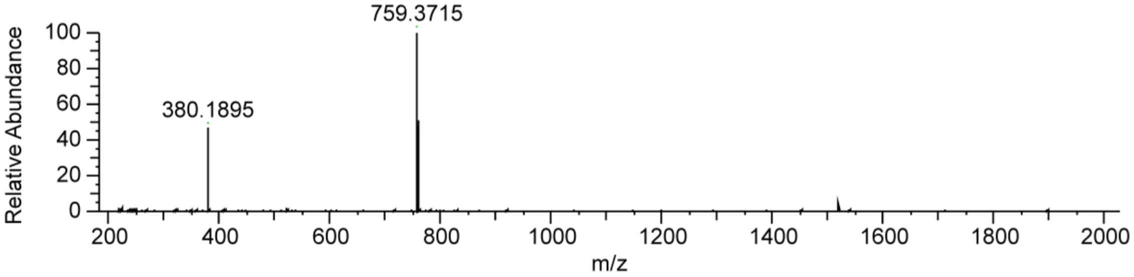

Supplementary Fig. 15: Flow Cytometry of H_2H-AERQ-CO_2H Analogs

HeLa cells were grown in 60 x 15 mm Nunclon™ dishes. Stock solutions of H_2N-AERQ-CO_2H analogs were prepared before being diluted to final desired concentration in 3 mL of media. Cells were placed in the incubator for 24 hours. Cells were then detached with trypsin and stained using Annexin V/PI following manufacturer's protocol. Annexin V (AV) conjugated to FITC was used to determined apoptosis. Propidium Iodide (PI) was used to determine necrosis within the cellular populations. Cells were analyzed via flow cytometry within 1 hour to quantify cell death. A B515/20 laser was used to detect AV-FITC while a B710/50 laser was used to detect PI. FlowJo software was used to analyze the cytometry data. Data is an average of 3 replicates analyzed on separate days using different passage numbers of cells. **Note**: Gating is based on the AV/PI controls; samples were analyzed respective to the controls on the day of analysis. Significant naïve cell death was observed due to overgrowth of cells over the 48-hour dosage period.

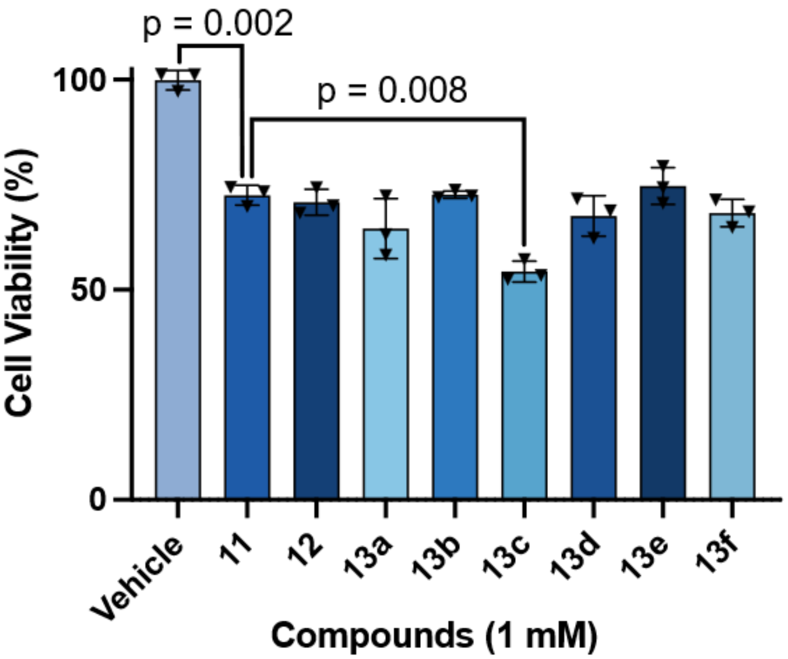

Representative Flow Cytometry for Each Compound

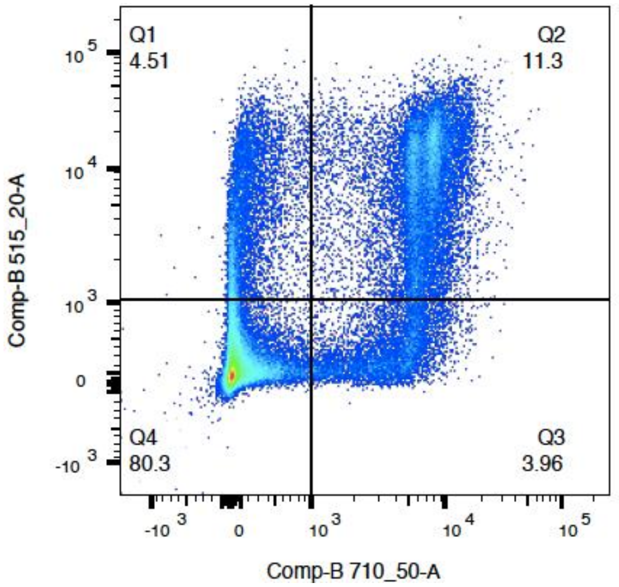

Vehicle (DMSO)

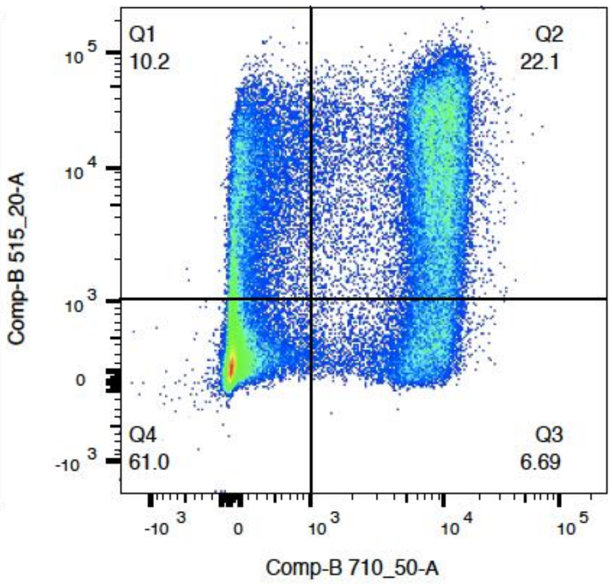

Amide (**11**)

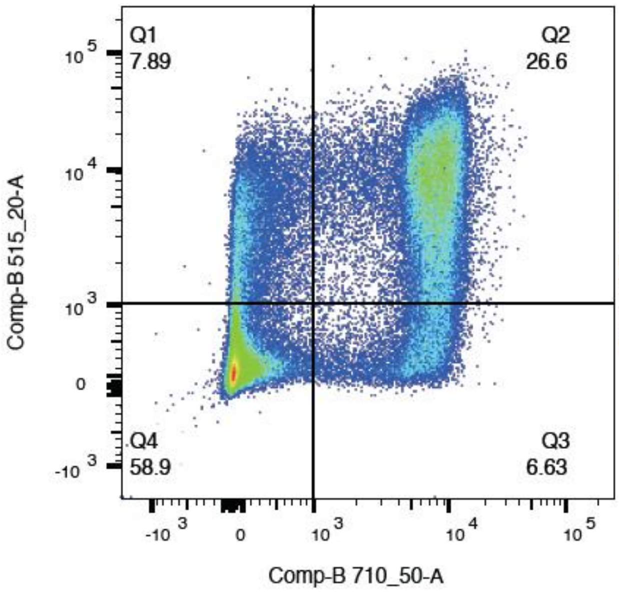

Nitrile (**12**)

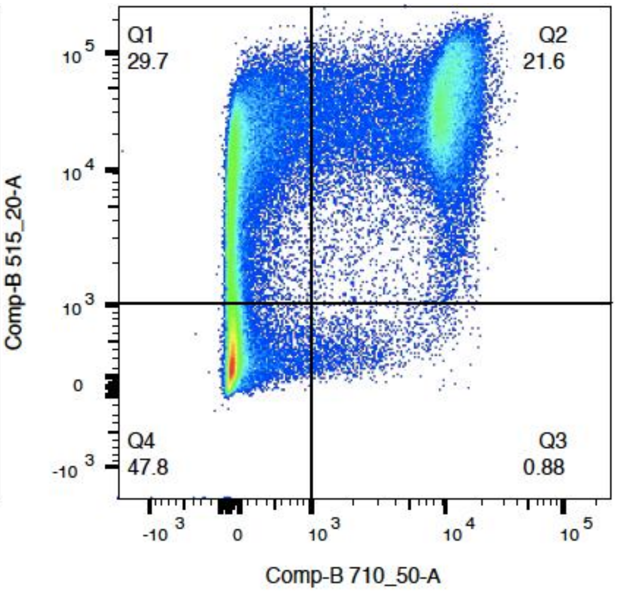

4-methoxyphenylketone (**13a**)

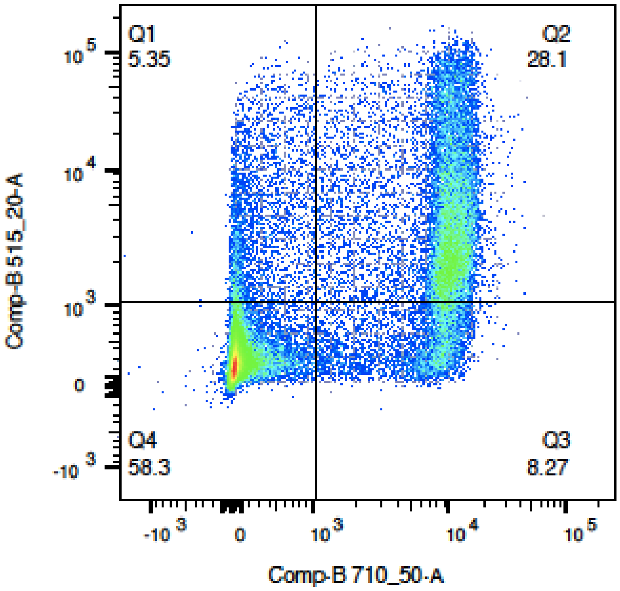

4-chlorophenylketone (**13b**)

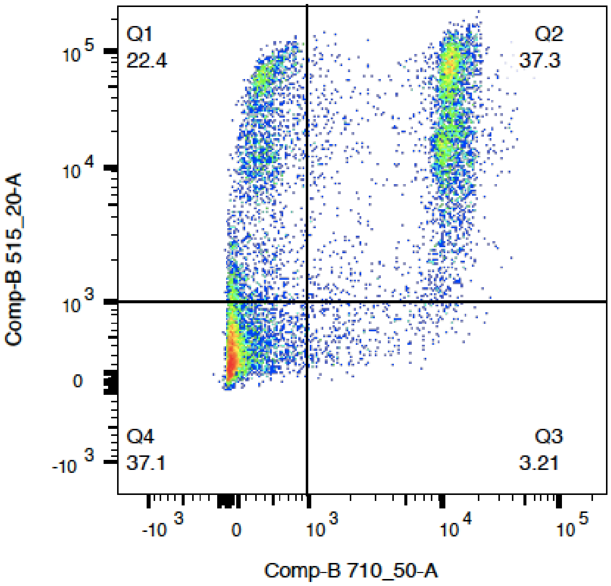

Triphenylamine (**13c**)

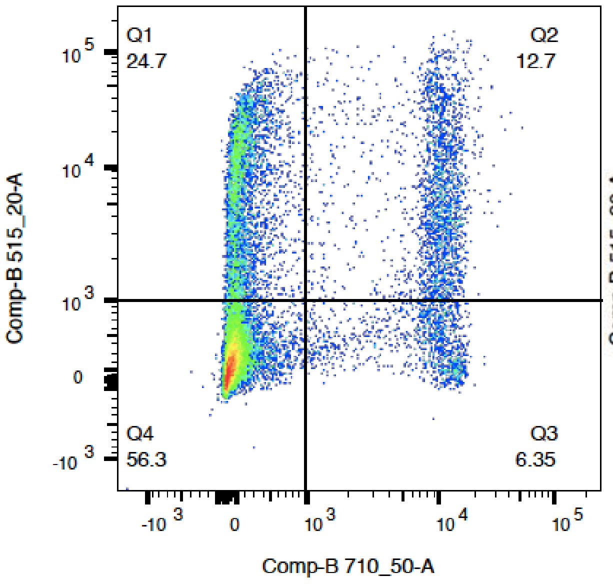

3-thiophene ketone (**13d**)

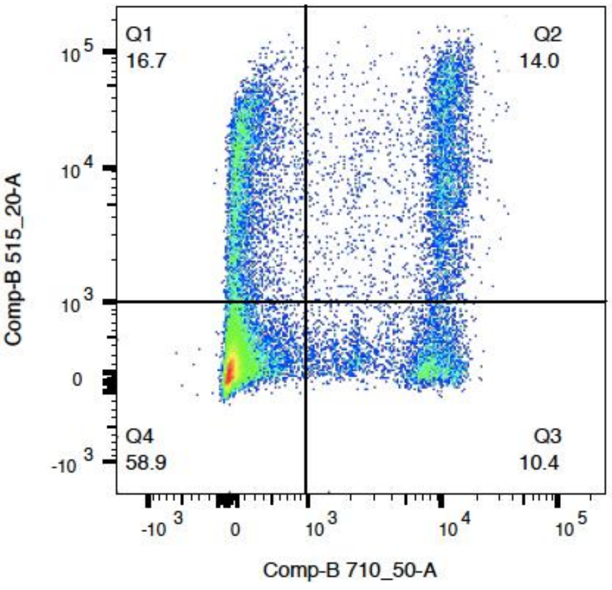

4-butylthiophenylketone (**13e**)

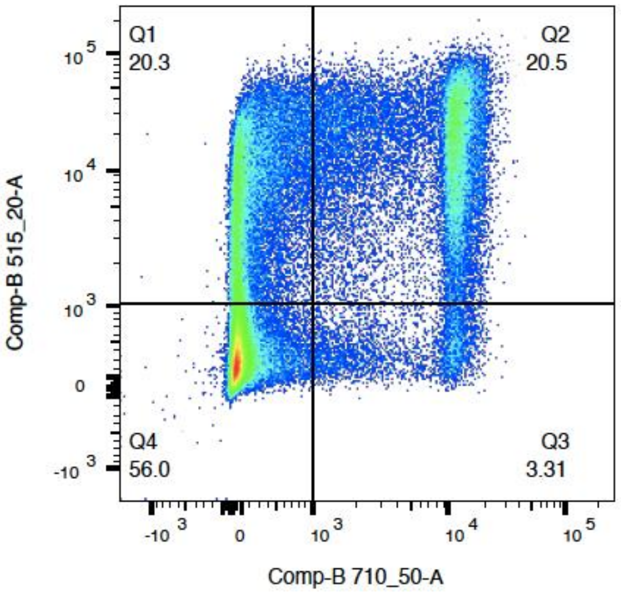

1,4-Benzodioxane ketone (**13f**)

Supplementary Fig. 16: Modification of Two Asn Residues with Boronic Acid

Looking towards boronic acid protein modification and applications such as fluorosequencing, we next sought to demonstrate the ability to convert multiple Asn/Gln residues in a peptide to nitrile with further modification by boronic acid on a large scale.

Synthesis of H_2N-WNGRNFG-CO_2H 14

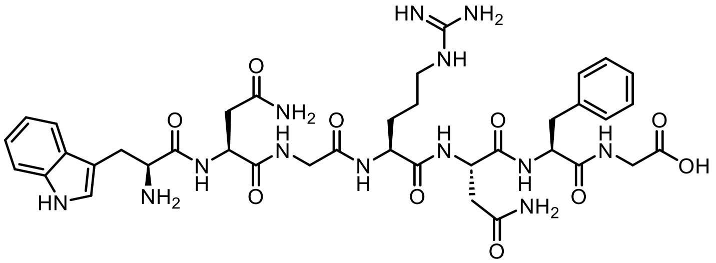

H_2N-WNGRNFG-CO_2H (14): LCMS, *m/z* 850.3937 (calcd. [M+H$^+$] = 850.3955), *m/z* 425.7003 (calcd. [(M+2H$^+$)/2] = 425.7014), Purity: >99% (HPLC analysis at 220 nm). Retention time using **HPLC Method A**: 6.6 min.

HPLC Trace for H_2N-WNGRNFG-CO_2H 14

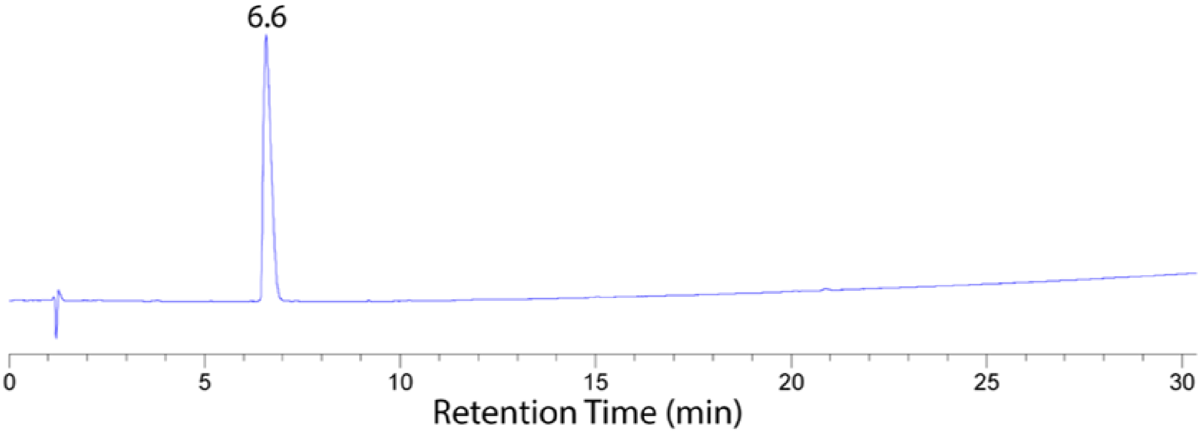

HRMS of H_2N-WNGRNFG-CO_2H 14

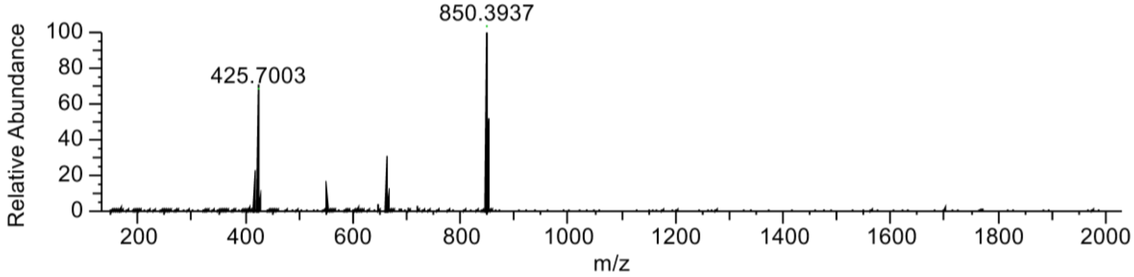

Conversion of H_2N-WNGRNFG-CO_2H to Dinitrile 15

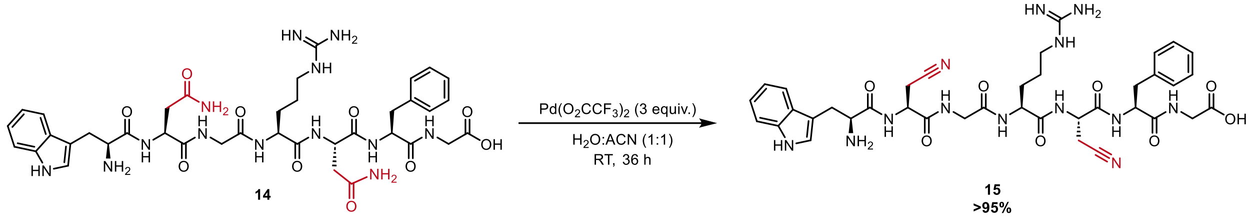

H_2N-WNGRNFG-CO_2H **14** (30 mg, 0.035 mmol, 1 equiv.) was dissolved in 2 mL of 1:1 H_2O:ACN in a 1/2” dram vial. Next, $Pd(O_2CCF_3)_2$ (35.2 mg, 0.11 mmol, 3 equiv.) was added in one portion. The concentration of peptide in the solution was 17.6 mM. The vial was stirred at room temperature for 36 hours then quenched with 3-MPA (94 µL, 20 equiv.). The reaction was analyzed via **HPLC Method A**, revealing >95% conversion to nitrile product. Fractions were then collected via preparative HPLC and lyophilized to produce pure H_2N-WNGRNFG-CO_2H di-nitrile **15** as a fluffy white powder (15.2 mg, 53% yield).

H_2N-WNGRNFG-CO_2H Dinitrile (15): LCMS, *m/z* 814.3747 (calcd. $[M+H^+]$ = 814.3743), *m/z* 407.6913 (calcd. $[(M+2H^+)/2]$ = 407.6908), Purity: >99% (HPLC analysis at 220 nm). Retention time using **HPLC Method A**: 7.2 min.

HPLC Trace for H_2N-WNGRNFG-CO_2H Dinitrile 15

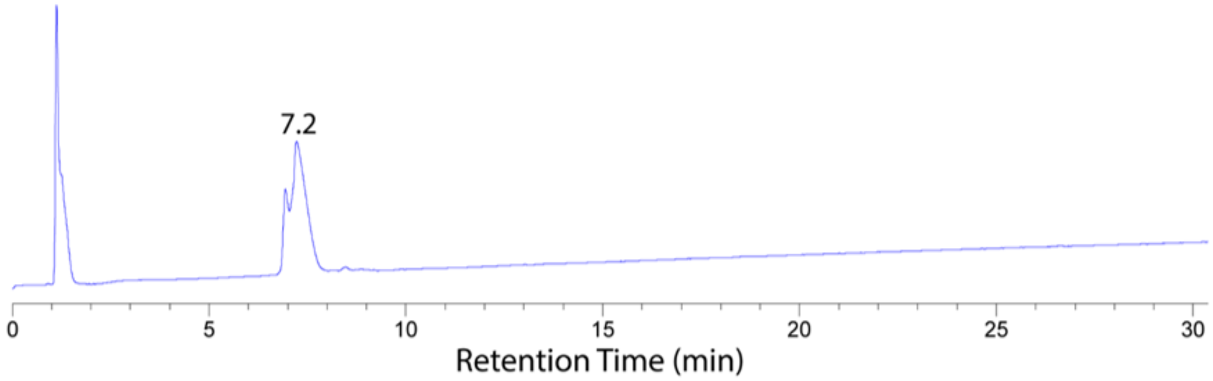

HRMS of H_2N-WNGRNFG-CO_2H Dinitrile 15

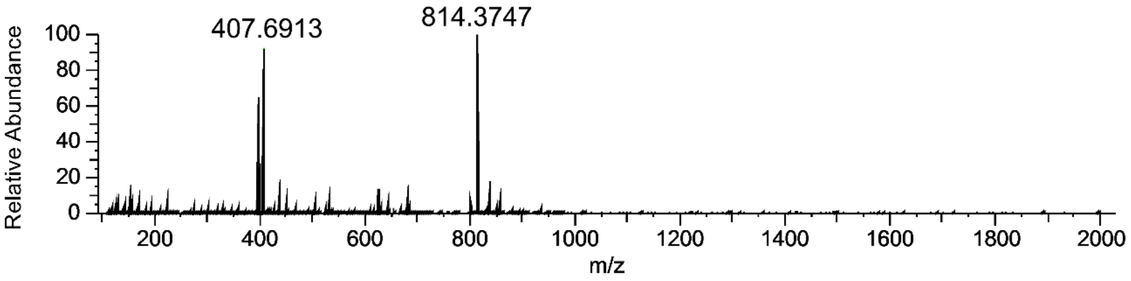

Modification of H_2N-WNGRNFG-CO_2H Dinitrile 15 with Boronic Acid

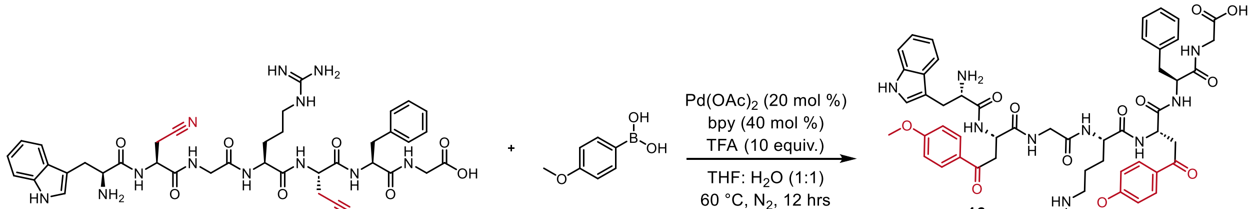

H_2N-WNGRNFG-CO_2H di-nitrile **15** (5.0 mg, 6.1 µmol, 1 equiv.) and 4-methoxyphenylboronic acid (5.6 mg, 36.9 µmol, 6 equiv.) were added to a 1" dram vial. Next, 2,2'-bipyridyl ligand (0.38 mg, 2.46 µmol, 40 mol%) was added from a freshly prepared stock solution (90 µL, 1:1 H_2O:THF). TFA (4.7 µL, 61.4 µmol, 10 equiv.) was then transferred to the solution, after which 810 µL H_2O:THF (1:1) was added to bring the total reaction volume to 900 µL. N_2 was bubbled for 2 minutes using an 18G x 1 ½" needle and balloon. Finally, $Pd(OAc)_2$ (0.28 mg, 1.23 µmol, 20 mol%) was added in 30 µL from a freshly prepared stock solution (1:1 H_2O:THF). N_2 was bubbled for another 60 seconds using an 18G x 1 ½" needle and balloon, and then the vial was flushed with N_2. The reaction was left stirring for 12 hours at 60 °C. The reaction was quenched with 3-MPA (10.7 µL, 20 equiv.) and analyzed via **HPLC Method A**, revealing >95% conversion to doubly ketone modified H_2N-W**N**GR**N**FG-CO_2H **16.** Fractions were then collected via analytical HPLC and lyophilized to produce pure **16** as a fluffy white powder (3.4 mg, 54% yield).

Doubly Modified H_2N-WNGRNFG-CO_2H (16): LCMS, *m/z* 1032.4572 (calcd. $[M+H^+]$ = 1032.4574), *m/z* 516.7318 (calcd. $[(M+2H^+)/2]$ = 516.7324). Purity: >99% (HPLC analysis at 220 nm). Retention time using **HPLC Method A:** 10.0 min.

HPLC Trace for Doubly Modified H_2N-WNGRNFG-CO_2H 16

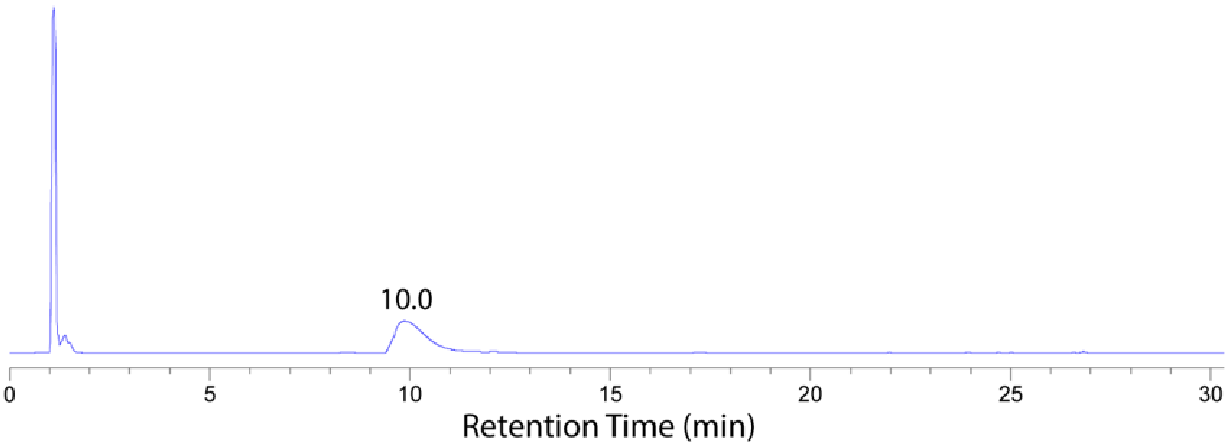

HRMS of Doubly Modified H_2N-WNGRNFG-CO_2H 16

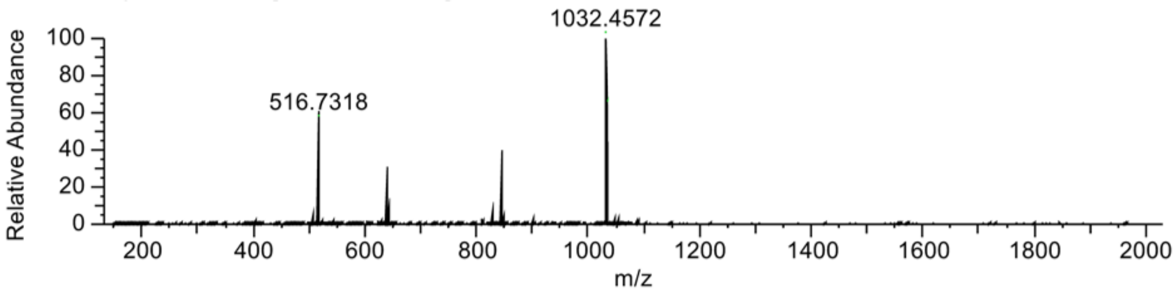

Supplementary Fig. 17: Asn/Gln Fluorosequencing

Preparation of Labeled Peptides for Fluorosequencing

General Procedure for Fluorosequencing Peptide Dehydration to Nitrile(s) (GP-XII):
Asn-containing peptides **17-18** (20 µmol, 1 equiv.) was dissolved in H_2O/ACN (1:1 v/v, 1.5 mL). $Pd(O_2CCF_3)$ (33.2 mg, 100 µmol, 5 equiv.) was added. The concentration of peptide in the solution was 13.3 mM. The mixture was stirred at room temperature for 24 h, then quenched with EDA (27 µL, 400 µmol, 20 equiv.). The product **19-20** was purified via preparative HPLC and lyophilized.

Synthesis of H_2N-AVN(Nitrile)GAYSYRA-CO_2H 19

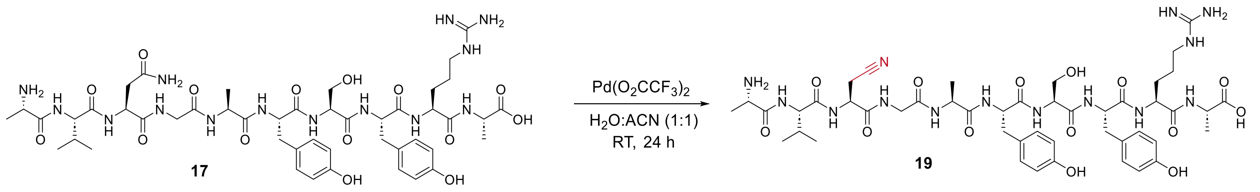

H_2N-AVN(nitrile)GAYSYRA-CO_2H **19** was synthesized from H_2N-AVNGAYSYRA-CO_2H **17** according to **GP-XII**.

H_2N-AVN(Nitrile)GAYSYRA-CO_2H (19): HRMS: *m/z* 1053.5123 (calcd. $[M+H]^+$ = 1053.5112). Retention time in LCMS: 2.5 min.

LCMS Trace of H_2N-AVN(Nitrile)GAYSYRA-CO_2H 19

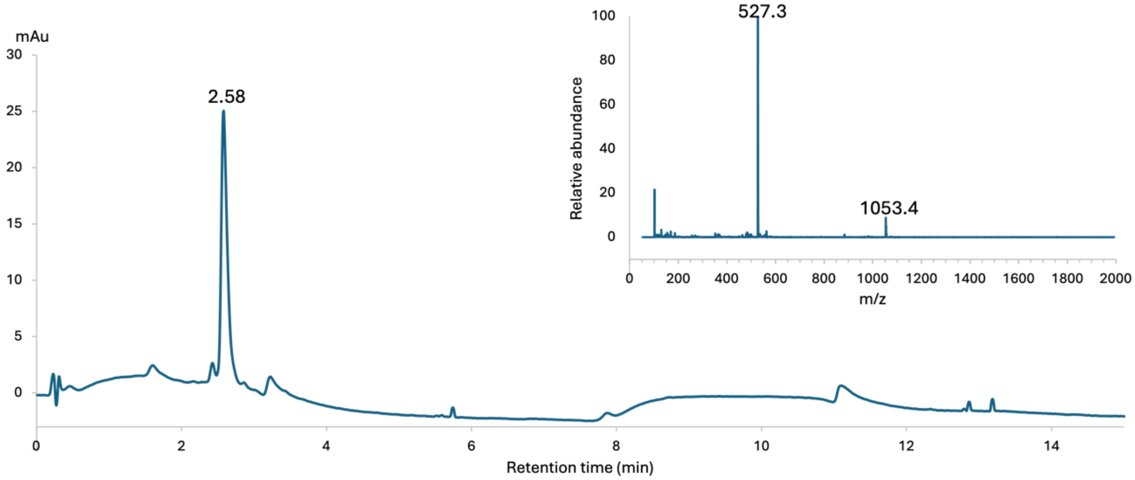

Synthesis of H_2N-GAN(Nitrile)AGN(Nitrile)AYGYR-CO_2H 20

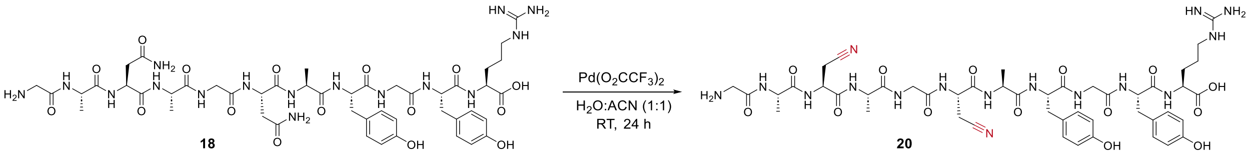

H_2N-GAN(Nitrile)AGN(Nitrile)AYGYR-CO_2H **20** was synthesized from H_2N-GANAGNAYGYR-CO_2H **18** according to **GP-XII**.

H_2N-GAN(Nitrile)AGN(Nitrile)AYGYR-CO_2H (20): LCMS: *m/z* 1077.3 (calcd. $[M+H]^+$ = 1077.4872). Retention time in LCMS: 2.2 min.

LCMS Trace of H_2N-GAN(Nitrile)AGN(Nitrile)AYGYR-CO_2H 20

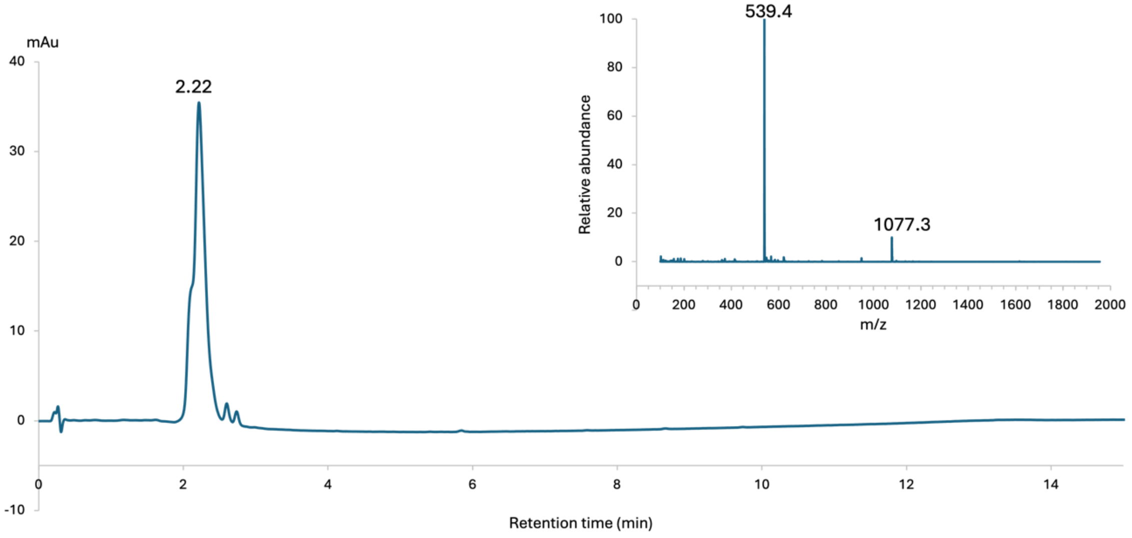

General Procedure for Fluorosequencing Peptide Modification with Azide-Functionalized Boronic acid (GP-XIII):

Nitrile-containing peptide **19-20** (9 µmol, 1 equiv.) and (4-(azidomethyl)phenyl)boronic acid (9.6 mg, 54 µmol, 6 equiv.), 2,2'-bipyridine (1.4 mg, 9 µmol, 1 equiv.) and TFA (7 µL, 90 µmol, 10 equiv.) were dissolved in H_2O/THF (2:3 v/v, 1.5 mL). The solution was degassed with N_2 for 2 minutes. A freshly prepared solution of $Pd(OAc)_2$ in THF (45 mM, 100 µL, 50 mol%) was added. The mixture was degassed for an additional 2 minutes, then heated to 60 °C for 12 hours. The reaction was quenched with 3-MPA (8 µL, 90 µmol, 10 equiv.), then peptide products **21-22** were purified via preparative HPLC and lyophilized.

Synthesis of H_2N-AVN(azide)GAYSYRA-CO_2H 21

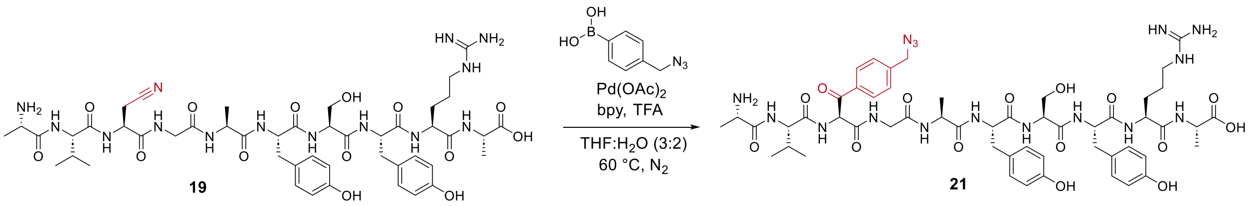

H_2N-AVN(azide)GAYSYRA-CO_2H **21** was synthesized according to **GP-XIII.**

H_2N-AVN(azide)GAYSYRA-CO_2H (21): HRMS: *m/z* 1187.5588 (calcd. $[M+H]^+$ = 1187.5592). Retention time in LCMS: 3.8 min.

LCMS Trace of H_2N-AVN(azide)GAYSYRA-CO_2H 21

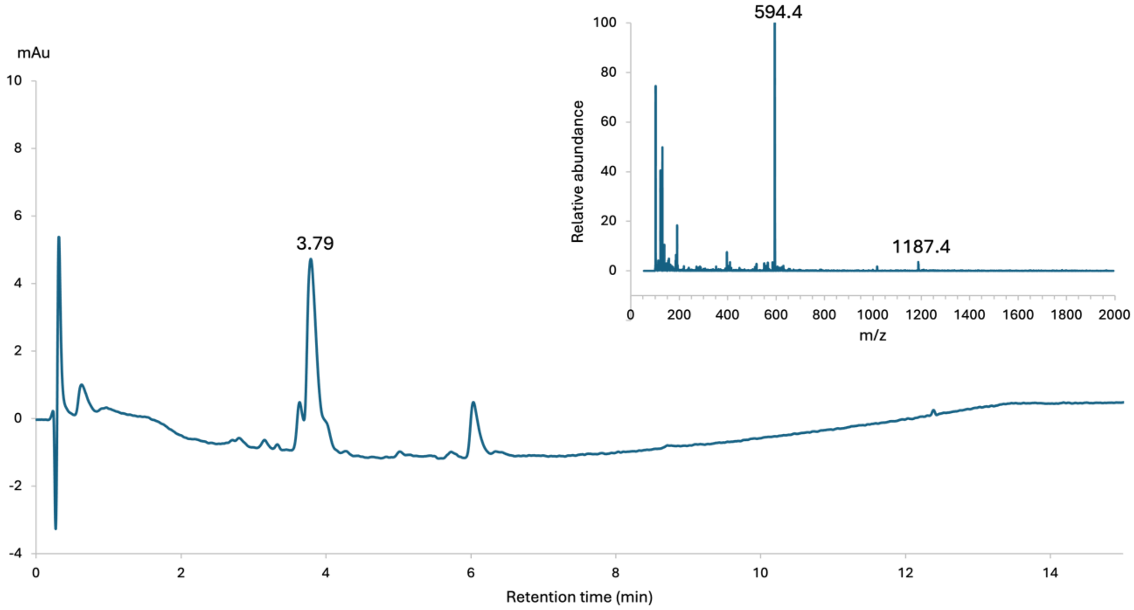

Synthesis of H_2N-GAN(azide)AGN(azide)AYGYR-CO_2H 22

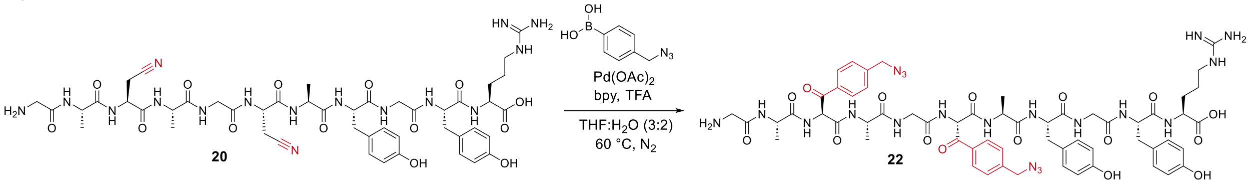

H_2N-GAN(azide)AGN(azide)AYGYR-CO_2H **22** was synthesized according to **GP-XIII** but using 10 equiv. boronic acid and increasing the reaction time to 36 hours.

H_2N-GAN(azide)AGN(azide)AYGYR-CO_2H (22): HRMS: *m/z* 1345.5828 (calcd. $[M+H]^+$ = 1345.5821). Retention time in LCMS: 4.4 min.

LCMS Trace of H_2N-GAN(azide)AGN(azide)AYGYR-CO_2H 22

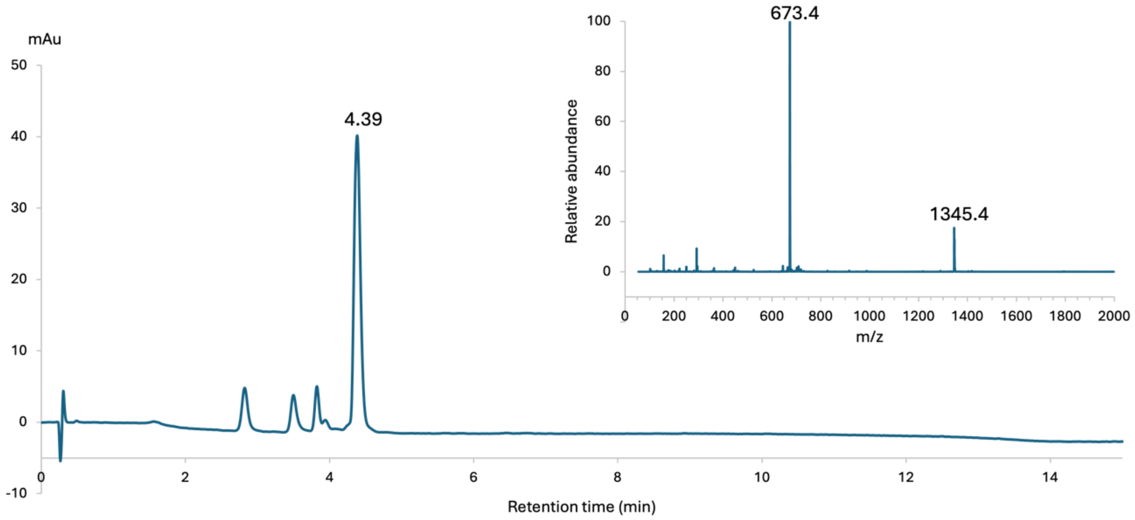

General Procedure for Addition of Fluorophore(s) and C-Terminal Alkyne (GP-XIV):
In order to add the fluorophore(s) and introduce an alkyne on the C-terminal, the azide-functionalized peptides **21-22** were captured on a PCA-resin. The fluorophore(s) were then added through copper-free Click chemistry, followed by addition of an alkyne on the C-terminal through coupling with propargylamine. Release of the peptide from the PCA-resin by heating in ACN/200 mM TRIS pH 8 afforded the fully labeled peptides **23-24**, as well as the fully labeled peptide with a transaminated N-terminal. The mixture of target peptide and transaminated peptide was used as is for fluorosequencing (see full details below).

Synthesis of PCA-resin

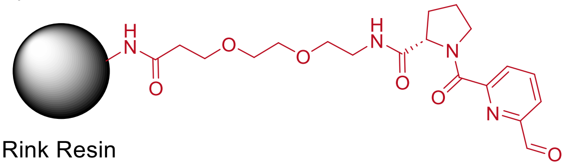

0.5 g of NovaSyn TGR R resin was transferred to a 20 mL fritted syringe and washed sequentially with DMF (3 × 10 mL), DCM (3 × 10 mL), and MeOH (3 × 10 mL). The resin was dried under vacuum (~15 min) and reswelled in DMF. A solution of Fmoc-PEG$_2$-COOH (3 eq), HBTU (3 eq), and HOBt (3 eq) in amine-free DMF (7.5 mL total) containing NMM (6 eq) was prepared and added to the resin. The mixture was gently agitated at room temperature for 4 h, then washed with DMF (3 × 10 mL) and DCM (3 × 10 mL).

The resin was treated with a solution of acetic anhydride and pyridine in amine-free DMF (7.5 mL total, 1:1:1 v/v/v) for 30 min at room temperature, washed with DMF (3 × 10 mL), DCM (3 × 10 mL), and MeOH (3 × 10 mL), and dried under vacuum. The Fmoc group was removed by treating the resin with 20% piperidine in amine-free DMF (10 mL) for 30 min, followed by washing with DMF (3 × 10 mL) and DCM (3 × 10 mL).

A coupling solution of Fmoc-Pro-COOH (3 eq), HBTU (3 eq), and HOBt (3 eq) in amine-free DMF (7.5 mL total) containing NMM (6 eq) was added to the resin, and the suspension was rocked

for 2 h at room temperature. After washing with DMF (3 × 10 mL) and DCM (3 × 10 mL), the acetylation and deprotection steps were repeated as above.

Finally, a solution of 6-formylpyridine-2-carboxylic acid (Enamine, EN300-158932, CAS# 499214-11-8) (3 eq), HBTU (3 eq), and HOBt (3 eq) in amine-free DMF (7.5 mL total) containing NMM (6 eq) was added to the resin and agitated for 1 h at room temperature. The resin was washed with DMF (3 × 10 mL), DCM (3 × 10 mL), and MeOH (3 × 10 mL), then dried under vacuum (~15 min) and stored at 4 °C until use.

Labeling of Peptides on Resin

5 mg of PCA-resin was washed with ACN/H_2O (1:1 v/v, 3 x 200 µL) and ACN/100 mM HEPES pH 8.5 (1:1, 200 µL). A solution of peptide in ACN/H_2O/100 mM HEPES pH 8.5 (1:1:2 v/v/v, 1 mM in peptide) was prepared. 100 µL of peptide solution (100 nmol) was added to the resin and the mixture was heated to 40 °C for 18 hours (1200 rpm). The solution was removed, and the resin was washed with ACN/H_2O (1:1 v/v, 3 x 200 µL) and ACN/100 mM HEPES pH 8.5 (1:1 v/v, 200 µL).

A solution of Atto643-PEG4-DBCO (ATTO-TEC, AD 643-291) in ACN/H_2O/100 mM HEPES pH 8.5 (1:1:2 v/v/v, 2 mM in fluorophore) was prepared. 50-100 µL fluorophore solution (100-200 nmol) was added to the resin and the mixture was heated to 40 °C for 18 hours (1200 rpm). The solution was removed and the resin was washed with ACN/H_2O (1:1 v/v, 2 x 200 µL) and ACN/100 mM HEPES pH 8.5 (1:1 v/v, 200 µL).

A solution of $EtONH_2 \cdot HCl$ in ACN/100 mM HEPES pH 8.5 (1:1 v/v, 100 mM in $EtONH_2 \cdot HCl$) was prepared. The pH of the solution was adjusted to 7 by addition of 1 M NaOH. 200 µL of $EtONH_2$ solution (20 µmol) was added to the resin and the mixture was heated to 40 °C for 2 hours (1200 rpm). The solution was removed and the resin was washed with ACN/H_2O (1:1 v/v, 2 x 200 µL) and DMF (3 x 200 µL).

A solution of HBTU, HOBt, NMM and propargylamine in DMF (1:1:1:1, 25 mM in each reagent) was prepared. 200 µL of activator/amine solution was added to the resin and the mixture was shaken at room temperature for 1 hour (1200 rpm). The solution was removed, and the resin was washed with DMF (2 x 200 µL) and ACN/H_2O (1:1 v/v, 3 x 200 µL).

ACN/200 mM TRIS pH 8 (1:1 v/v, 200 µL) was added to the resin. The mixture was heated to 60 °C for 20 hours (1200 rpm). The solution was collected and used as is for fluorosequencing.

Synthesis of H_2N-AVN[F]GAYSYRA-$CONCH_2CCH$ 23

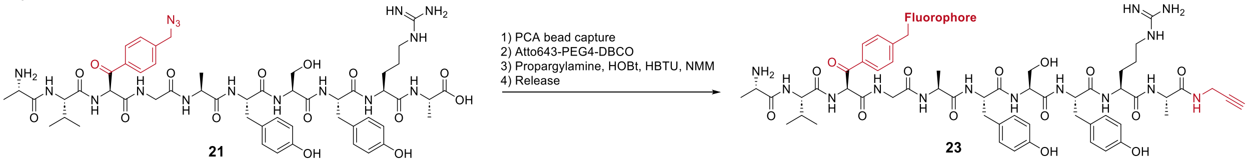

H_2N-AVN[F]GAYSYRA-$CONCH_2CCH$ **23** was synthesized according to **GP-XIV**, using 100 nmol (50 µL) of Atto643-PEG4-DBCO.

H_2N-AVN[F]GAYSYRA-$CONCH_2CCH$ (23): LCMS: *m/z* 856.1 (calcd. $[M+H]^{3+}$ = 855.7), *m/z* 1283.5 (calcd. $[M]^{2+}$ = 1283.1). Retention time in LCMS: 5.9 min.

LCMS Trace (640 nm) of H_2N-AVN[F]GAYSYRA-$CONCH_2CCH$ 23

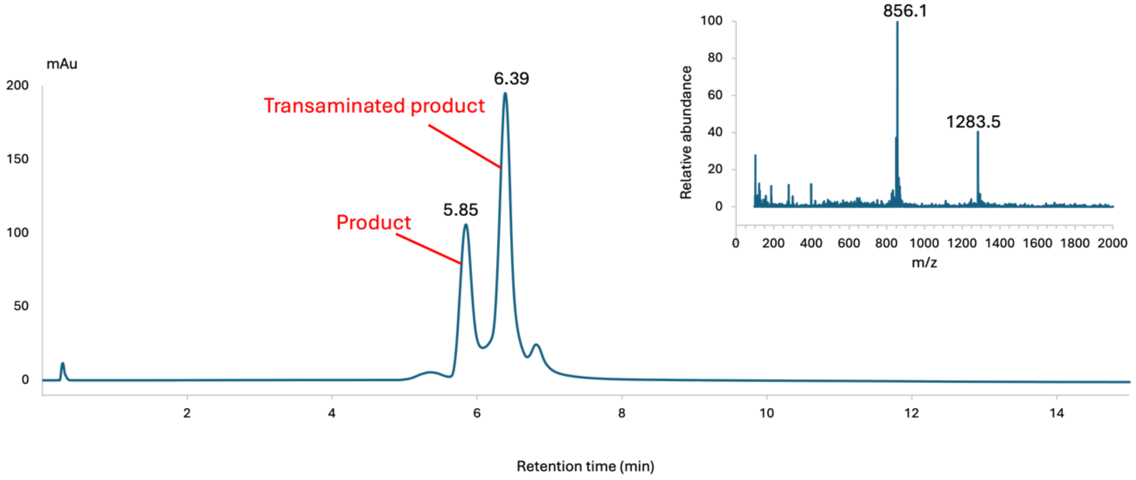

Synthesis of H_2N-GAN[F]AGN[F]AYGYR-$CONHCH_2CCH$ 24

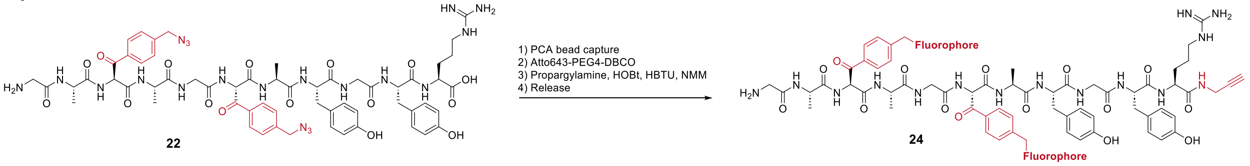

H_2N-GAN[F]AGN[F]AYGYR-$CONHCH_2CCH$ **24** was synthesized according to **GP-XIV**, using 200 nmol (100 µL) of Atto643-PEG4-DBCO.

H_2N-GAN[F]AGN[F]AYGYR-$CONHCH_2CCH$ (24): LCMS: *m/z* 1017.3 (calcd. $[M]^{4+}$ = 1016.7). Retention time in LCMS: 6.7 min.

LCMS Trace (640 nm) of H_2N-GAN[F]AGN[F]AYGYR-$CONHCH_2CCH$ 24

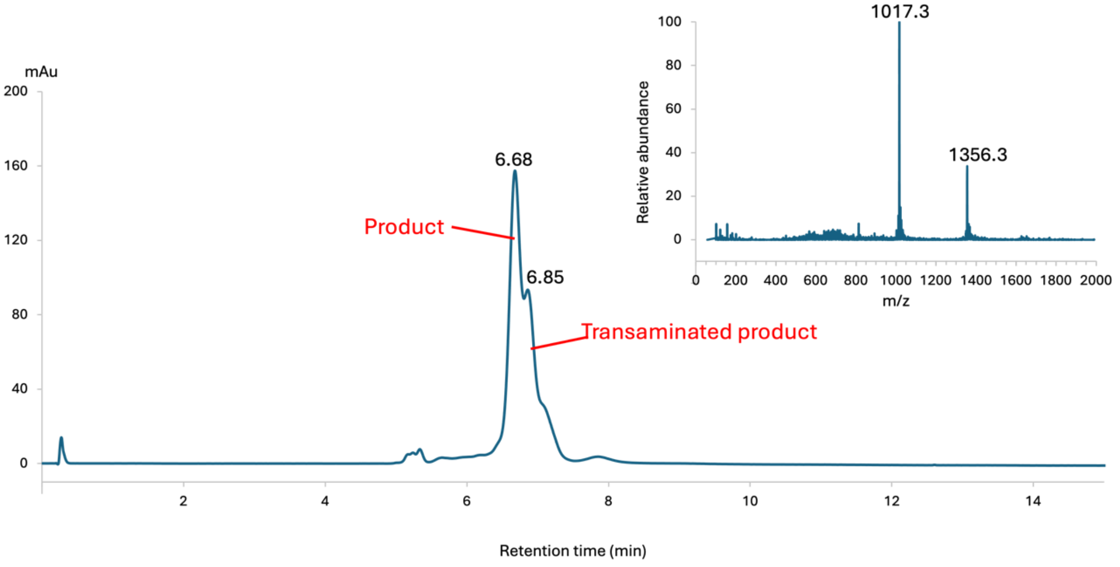

Fluorosequencing Protocol:

Silane Functionalization of Glass Slides

40 mm glass cover slides (Bioptechs, Cat 40-1313-03192) were cleaned for 10 minutes on each side using an UVO cleaner (Jelight, Model 18). After cleaning, the slides were placed vertically in a Teflon slide rack (custom-made). 100 µL of 3-azidopropyltriethoxysilane (Gelest, SIA0777, CAS# 83315-69-9) was pipetted into the lid of a Teflon Reaction Vessel (Alpha Nanotech Inc) and both the slide rack and the cap were placed in a Pyrex desiccator chamber, which had been preheated to 80 °C. The valve of the desiccator was attached to a vacuum pump and vacuum was drawn until the pump stabilized at approximately 0.08 MPa. The desiccator was placed in an 80 °C oven and allowed to sit for 16 hours. The silane-functionalized slides were stored in vacuum-sealed bags at 4 °C until use.

Peptide Immobilization

Peptides (containing alkyne) were covalently coupled to the coverslip surface via copper-catalyzed click chemistry between the alkyne-modified C-terminal AA residue and the azido silane. A fresh solution of 2 mM copper sulfate, 1 mM tris(3-hydroxypropyltriazolylmethyl)amine (Sigma, Cat # 762342), 20 mM HEPES (pH 8.0), and 5 mM sodium ascorbate with fluorescently labeled peptide was incubated for 30 min at room temperature on the coverslip, washed with water to remove unbound peptides, and dried under a nitrogen gas stream.

Total Internal Reflection Fluorescence (TIRF) Microscopy

Nikon Ti-E inverted microscope equipped with a CFI Apo 60X/1.49NA oil-immersion objective lens and a 1.5X tube lens, a motorized stage (TI2-S-HW, Nikon Inc Scientific), an 1022x1022 pixel sCMOS detector (pco.edge, PCO),and a LUNF-XL (Nikon) laser including 561 and 647 nm lasers and filter cube containing 405/488/561/638 quad dichroic and barrier filters, an emission filter wheel with band pass filters detailed below (all filters, Chroma). Each image represents a 72 µm × 72 µm square region of the sample. The different channels can now be considered as a combination of incident laser wavelength and the corresponding bandpass filter. The "640 channel" consists of excitation with the 640 nm laser (2.5 mW, 10%) and collected through quad dichroic and EM-705/72 emission filters. Laser powers measured after the objective.

Automated Fluidics for Performing Edman Sequencing Chemistry

Fluidic setup: The pumping of different solvents were atutomated using a syringe pump (Tecan Cavro, Model# 20738291) (3 way valve configuration) and a 10-port multi-position valve system (Valco Instruments, Model# EUHB), as described in the earlier publication (Swaminathan et al. 2019). The sample temperature was maintained at 40 °C (for System A) and 50 °C (for System B) by heating both the perfusion chamber and microscope objective for Edman sequencing experiments. Solvent exchanges in the fluidic device was controlled using in-house Python scripts and coordinated with image acquisition via custom macros in the Nikon Elements software package.

Descriptions of the solvents connected to the 10 port valve:

Valve Number	Name	Reagents
1	Waste	-
2	Imaging solvent:	1mM Trolox added to 5mM HEPES in Methanol. Solution is degassed
3	Base Solution:	Pyridine + N-Methylmorpholine (38:1 Pyr:NMM); Final concentration of NMM is 60 mM
4	PITC	System A: Phenyl isothiocyanate solution<br>System B: Phenyl isothiocyanate + hexafluoroisopropanol (19:1 vv PITC:HFIP)
5	Wash	20% piperidine in Acetonitrile
6	Edman reagent mixing chamber	-
7	Water	water
8	TFA	Trifluoroacetic (neat)
9	-	-
10	Flow cell	-
	Backing solvent	Acetonitrile

Steps for Performing One Cycle of Edman Degradation

Step		
	Reagent Composition	Incubation Time (mins)
Wash*	Acetonitrile	1 min
PITC mixing to base solution**	PITC	2 min
PITC coupling	20% PITC in Base solution	10 mins (x2)
Wash	Acetonitrile	-
Cleavage	100% TFA	4 mins (x2)
Wash	Acetonitrile	-
Imaging	1mM Trolox in Methanol	2 min

* Wash denotes exchanging the solvents in the flow chamber (approx. 3 minutes)
** Base solution comprises 60mM NMM in Pyridine
*** The PITC/HFIP mix is prepared at a 19:1 vv PITC:HFIP. Volumes of each are delivered to a clean, pre-rinsed glass bottle via glass serological pipettes in a fume hood. The mixing of these two reagents is endothermic. The solution is swirled several times to ensure complete mixing - the

mixing is complete when swirling the mix no longer causes it to turn opaque (~ 1 min of occasional mixing).

Signal Processing

We extracted raw sequencing reads from the time series micrographs as detailed in the Supplemental Notes and implemented in the Python package sigproc_v2, available from https://github.com/marcottelab/robust-fluorosequencing-plaster. Briefly, images capturing the same field of view were corrected for variation in signal intensity by regional illumination balancing and bandpass filtering of both background and over-saturated pixels, aligned across cycles to account for stage movement, and peaks identified by convolving with the point spread function (PSF). From radiometry on each peak, we calculated intensity parameters for each peak across Edman cycles to create a raw fluorosequencing read for each candidate peptide. Fields were filtered on the basis of image anomalies or poor alignments, and individual reads were rejected based on their fit to the PSF, with poor fits suggesting the presence of more than one molecule. A step-by-step workflow is described in our earlier publication (PMID: 37745461).

Supplementary Fig. 18: Optimization of Amide Dehydration to Nitrile on Ubiquitin

Optimization of Amide Dehydration to Nitrile on Ubiquitin

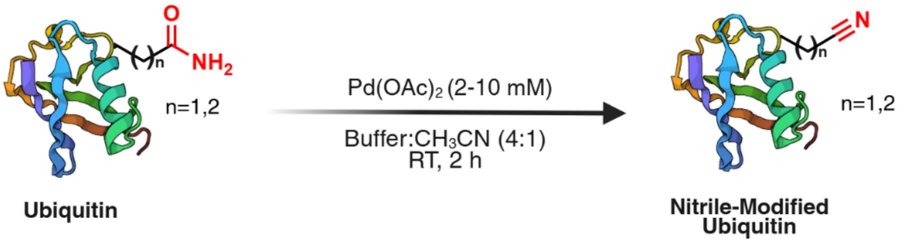

Ubiquitin (2 mg, 0.234 mM) was dissolved in 800 µL of NaP buffer (10 mM, pH 7.4) and 200 µL of $Pd(OAc)_2$ (2-10 mM) dissolved in ACN was added. The reaction was stirred at room temperature for 2 h followed by quenching with 500 µL of 1 M solution of aqueous L-cysteine and 10 µL of 1 M NaOH solution. The crude reaction mixture was passed through an Amicon™ Ultra 3 kDa centrifugal filter and washed with H_2O (7×0.5 mL) to remove the water-soluble Pd complex. The labeled protein was redissolved in 0.1% formic acid in H_2O and analyzed using LC-MS to determine conversion to nitrile (see table below). The sample modified with 5 mM $Pd(OAc)_2$ was additionally digested using SMART Digest™ Trypsin Kit by Thermo Scientific and analyzed by LC-MS/MS, with 8 sites of nitrile modification identified.

Concentration of $Pd(OAc)_2$	Unmodified Protein	1 Nitrile	2 Nitrile	≥ 3 Nitrile	Overall Conversion
2 mM (0.45 mg)	38%	50%	12%	n.d.	62%
5 mM (1.12 mg)	n.d.	33%	43%	24%	>95%
10 mM (2.24 mg)	n.d.	8%	22%	70%	>95%

MS of Unmodified Ubiquitin

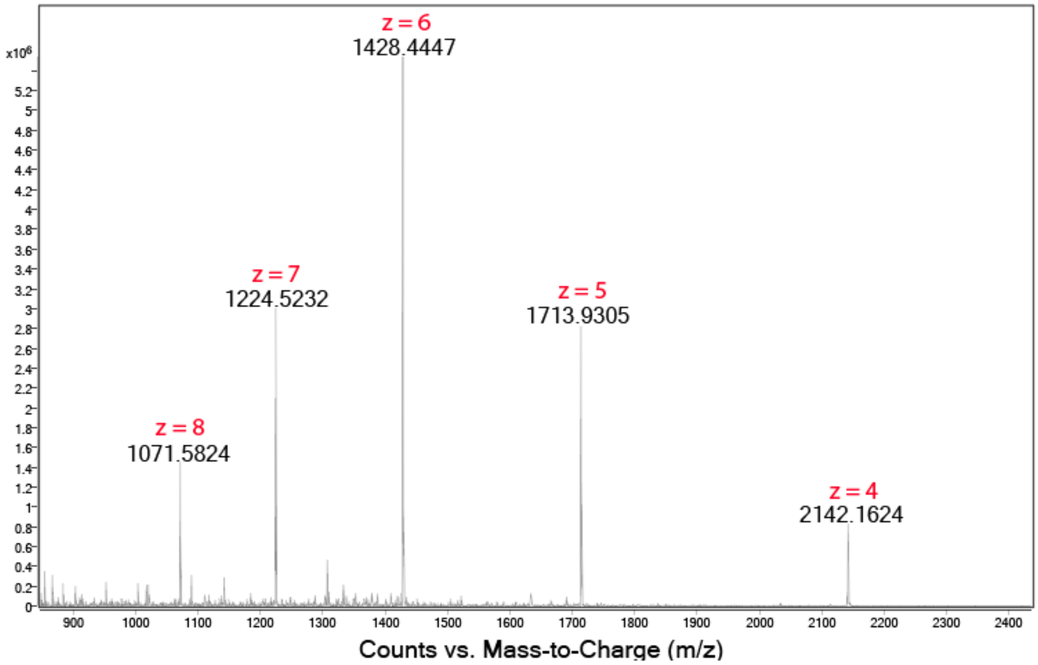

Deconvoluted MS of Unmodified Ubiquitin

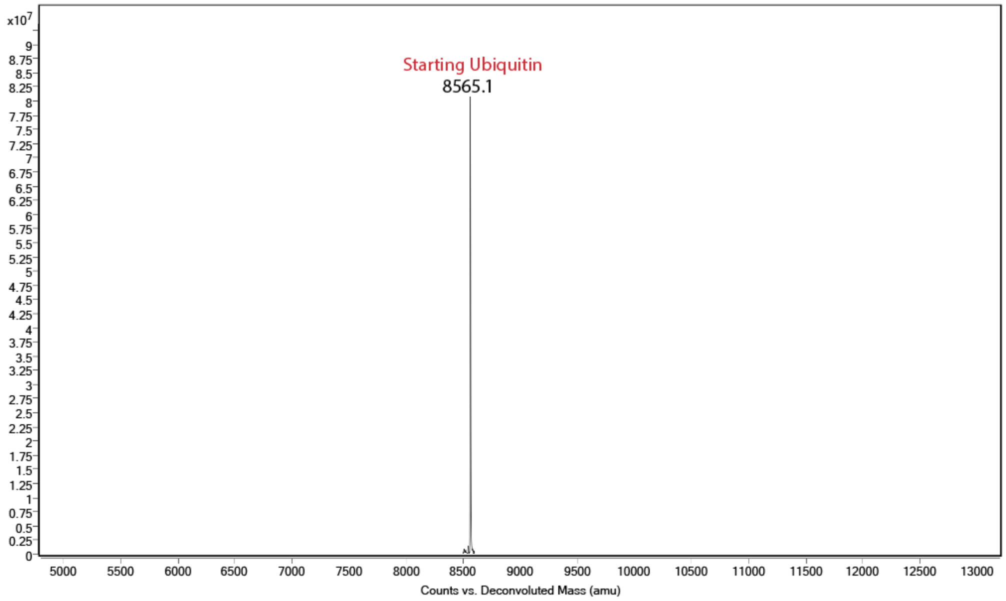

MS of Modified Ubiquitin (2 mM Pd(OAc)$_2$)

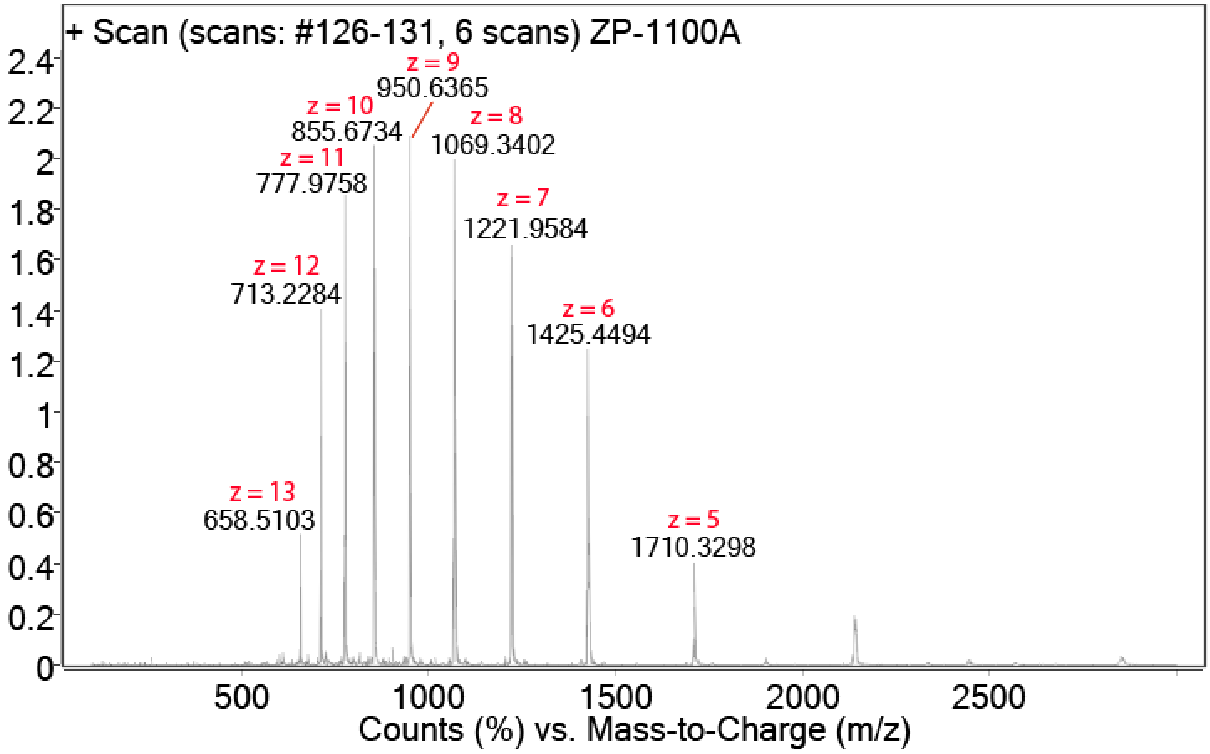

Deconvoluted MS of Modified Ubiquitin (2 mM Pd(OAc)$_2$)

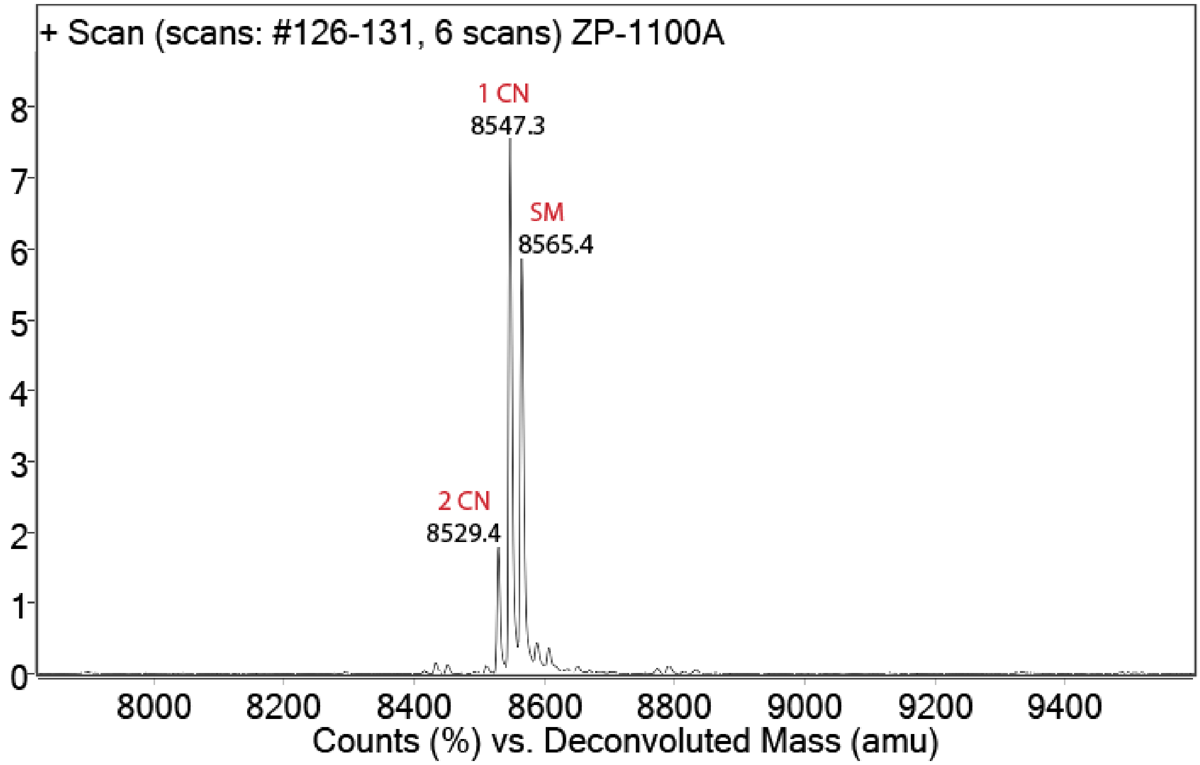

MS of Modified Ubiquitin (5 mM $Pd(OAc)_2$)

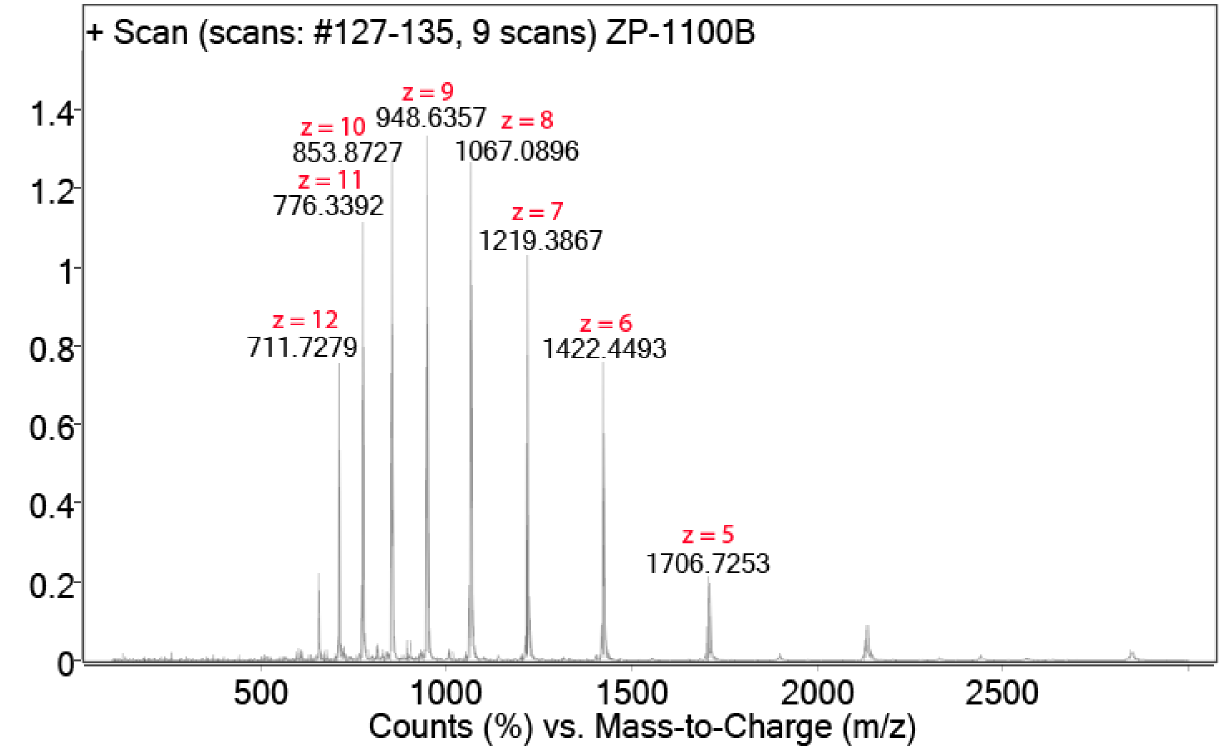

Deconvoluted MS of Modified Ubiquitin (5 mM $Pd(OAc)_2$)

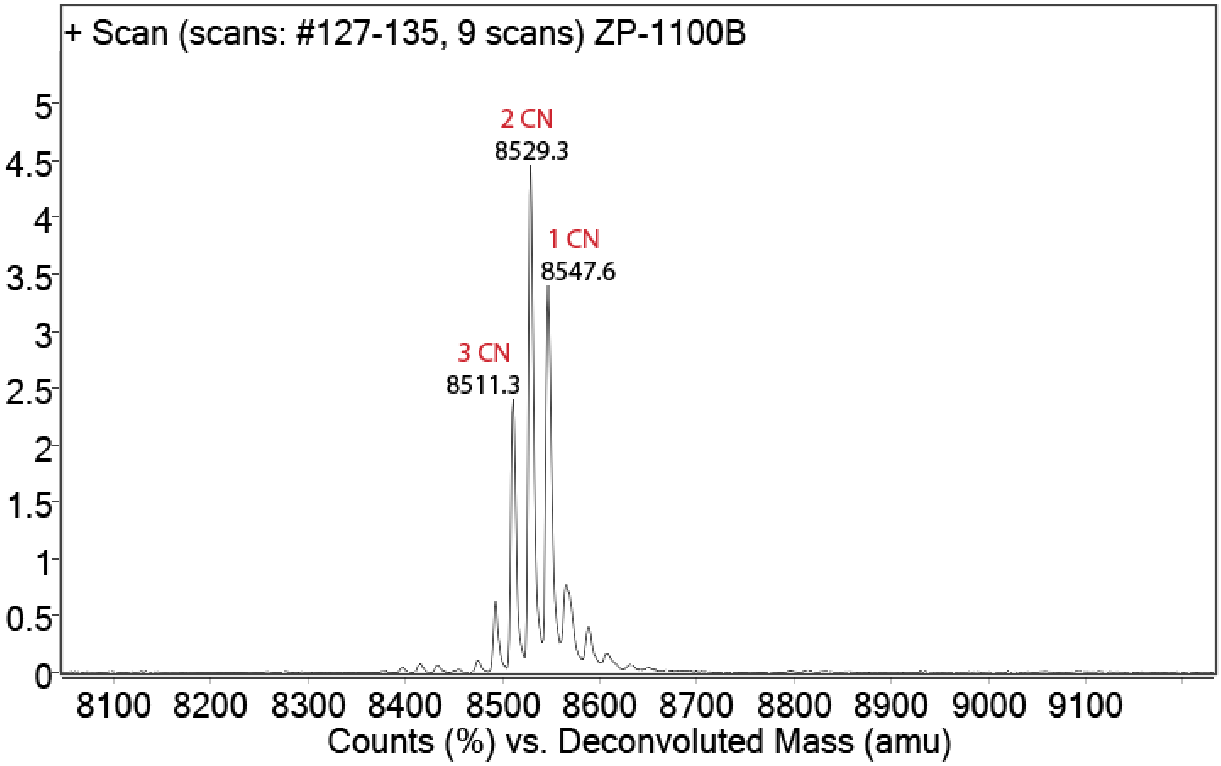

MS of Modified Ubiquitin (10 mM $Pd(OAc)_2$)

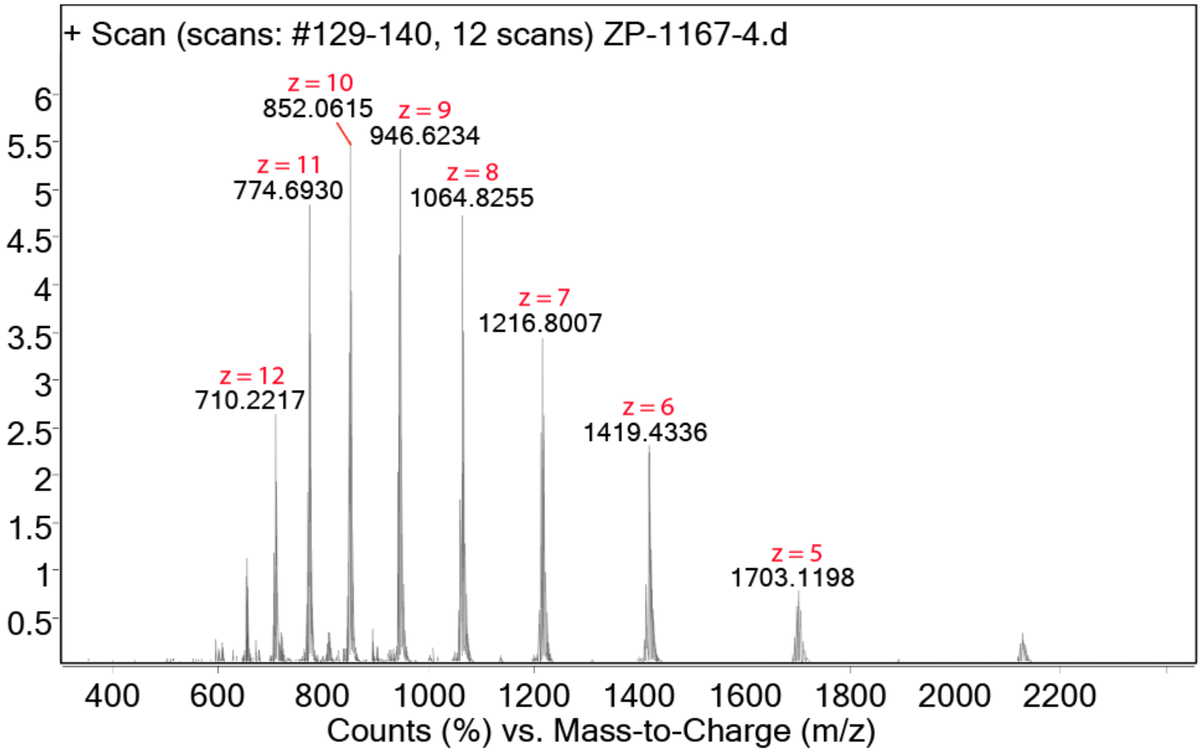

Deconvoluted MS of Modified Ubiquitin (10 mM $Pd(OAc)_2$)

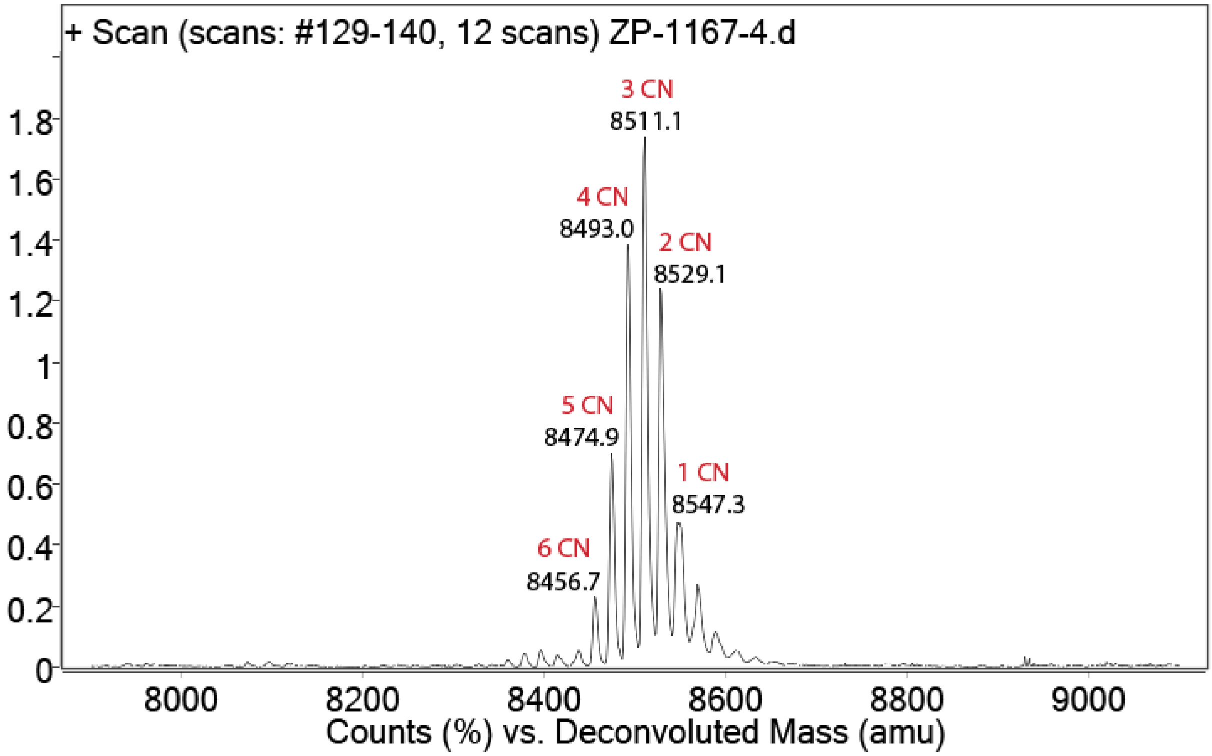

MS/MS Analysis of Digested Modified Ubiquitin

Identified peptide fragment **(1 site)**: MQIFVK (Sequence: AA 1-6, Q2)

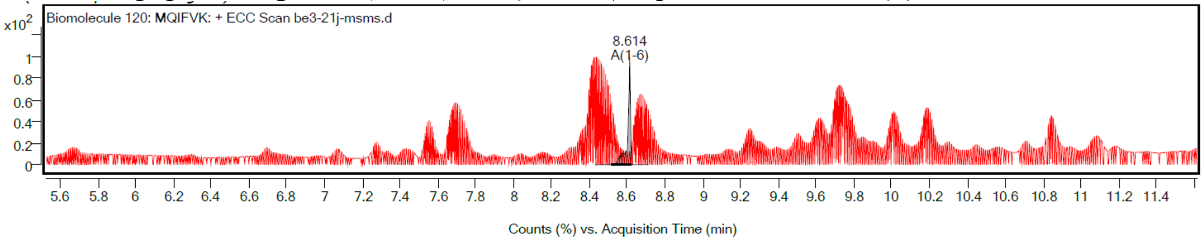

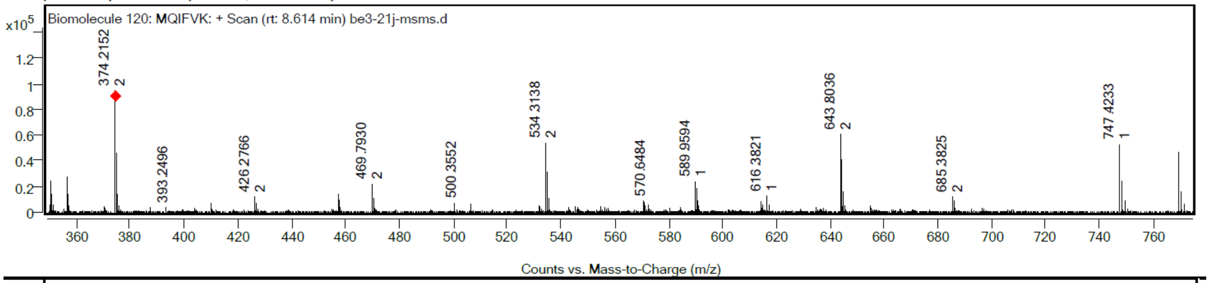

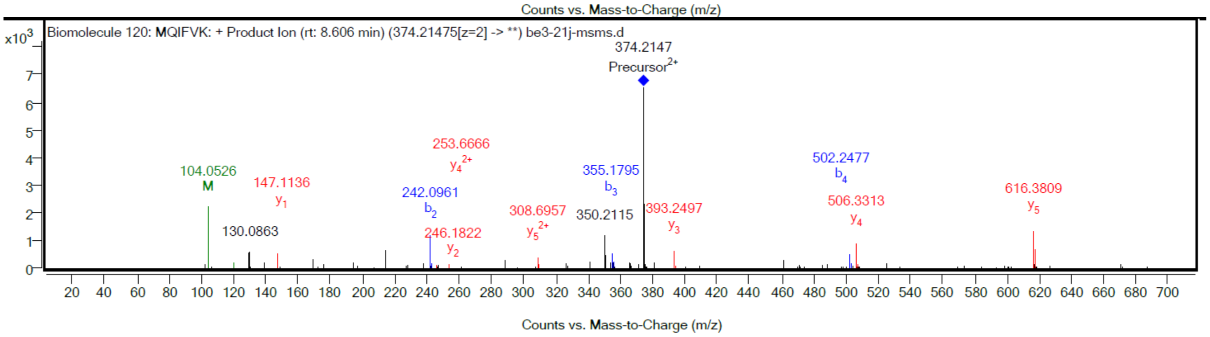

Fragment Spectrum Peaks

m/z	Diff (ppm)	Abund	Ion
147.1136	-5.45	505	y1
246.1833	-8.46	91	y2
393.2497	-0.14	588	y3
506.3313	4.67	854	y4
616.3809	1.28	1323	y5
253.6666	15.36	104	y4
308.6957	-3.81	349	y5
242.0961	-1.22	1170	b2
355.1795	0.89	509	b3
502.2477	1.12	464	b4
104.0526	2.29	2203	M
120.0804	2.71	176	F
374.2147	-0.03	6520	Precursor
374.7154	2.71	2294	Precursor
375.2134	12.55	122	Precursor

Identified peptide fragment **(2 sites)**: TITLEVEPSDTIENVKAKIQDK (Sequence: AA 12-33, [N25, Q31])

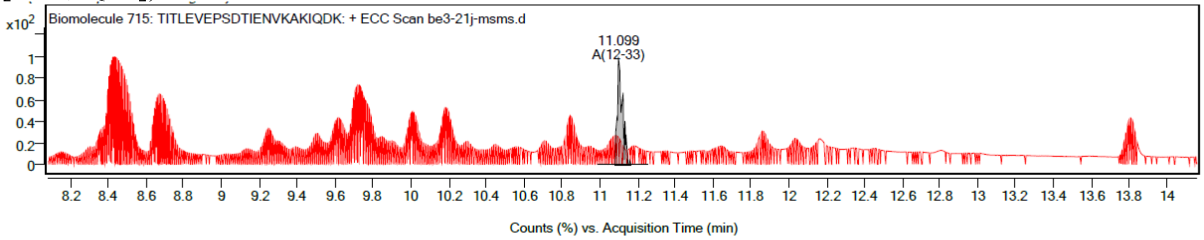

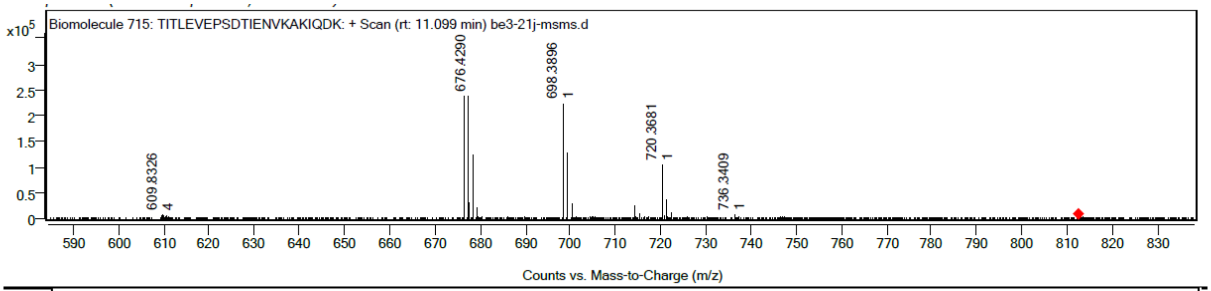

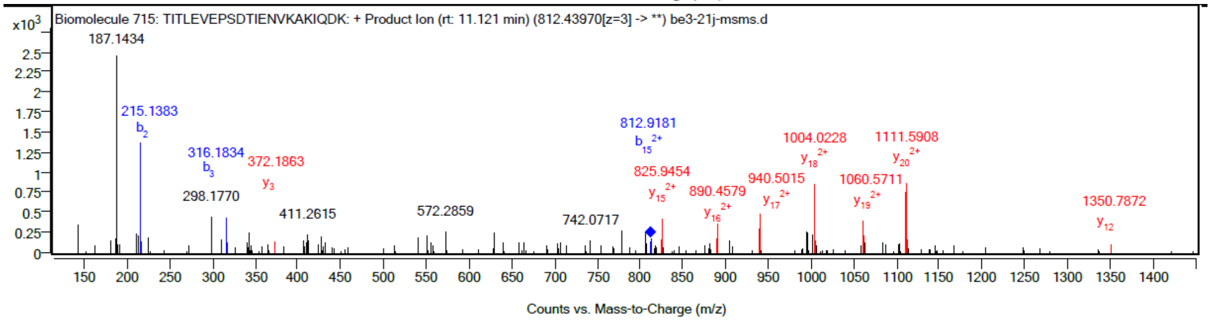

Fragment Spectrum Peaks

m/z	Diff (ppm)	Abund	Ion
372.1863	3.98	125	y3
1350.7872	-9.82	94	y12
825.9454	3.39	413	y15
890.4579	13.04	350	y16
940.5015	4.12	476	y17
1004.0228	0.45	844	y18
1060.5711	-5.42	386	y19
1111.5908	0.02	855	y20
215.1383	3.13	1362	b2
316.1834	10.37	430	b3
812.9181	-8.81	139	b15

Identified peptide fragment **(2 sites)**: EGIPPDQQR (Sequence: AA 34-42, [Q40, Q41])

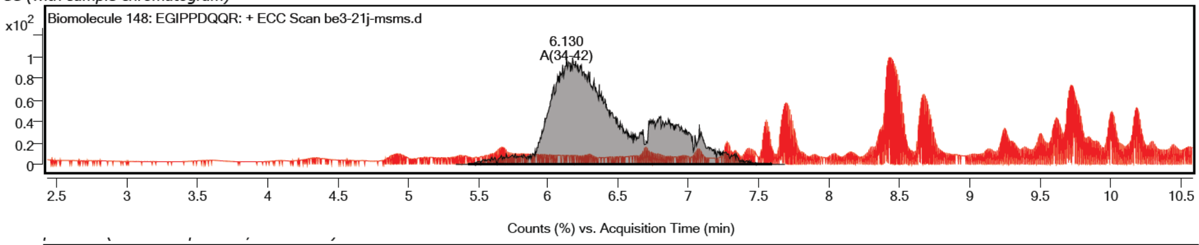

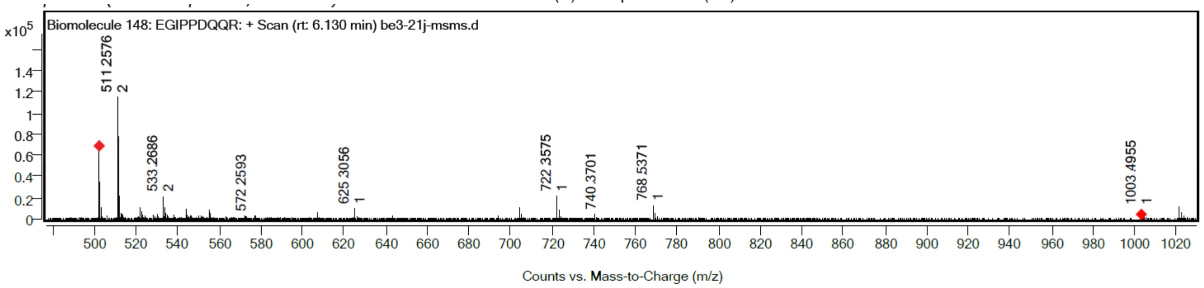

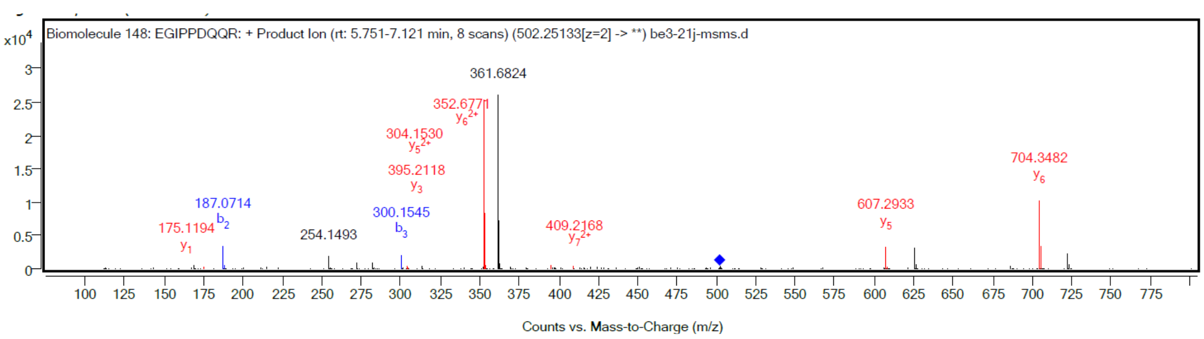

m/z	Diff (ppm)	Abund	Ion
175.1194	-2.29	364	y1
395.2118	7.93	542	y3
607.2933	2.30	3229	y5
704.3482	-1.12	10182	y6
304.1530	-6.66	464	y5
352.6771	0.74	25357	y6
409.2168	6.35	414	y7
187.0714	-0.59	3403	b2
300.1545	3.06	2064	b3

Fragment Spectrum Peaks

Identified peptide fragment **(1 site)**: QLEDGR (Sequence: AA 49-54, Q49)

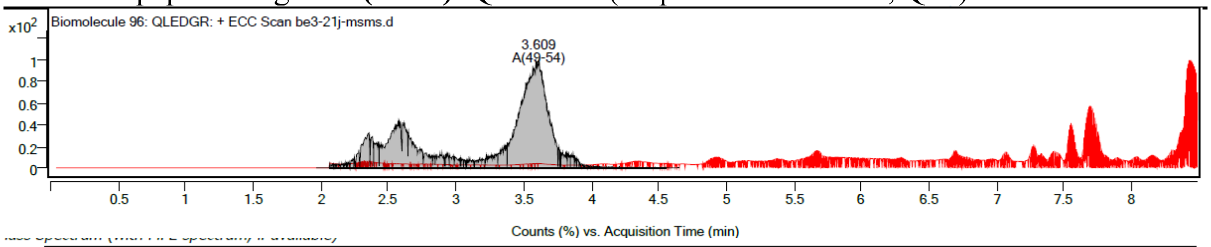

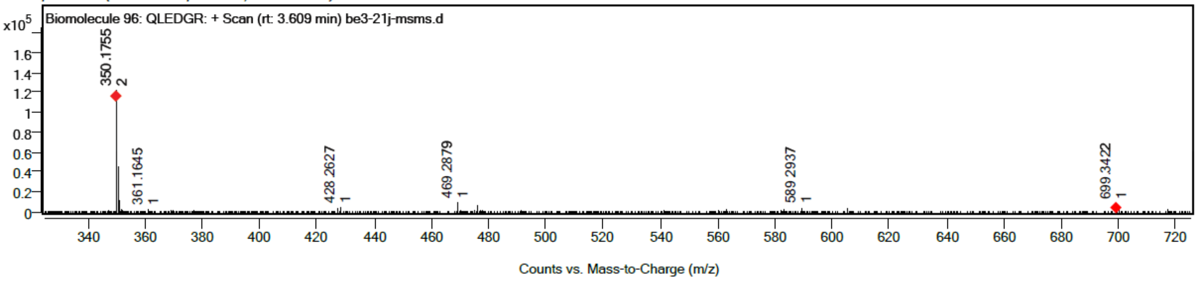

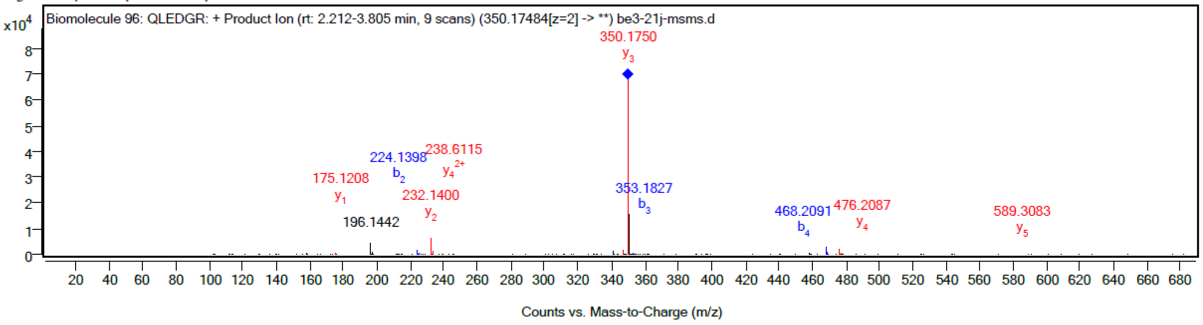

Fragment Spectrum Peaks

m/z	Diff (ppm)	Abund	Ion
175.1208	-10.75	448	y1
232.1400	1.93	6286	y2
350.1750	6.92	67858	y3
476.2087	2.74	2106	y4
589.3083	-24.16	305	y5
238.6115	-12.19	218	y4
224.1398	-1.99	1773	b2
353.1827	-2.06	592	b3
468.2091	-0.51	2726	b4
102.0535	13.91	212	E
350.6763	0.16	15451	Precursor
351.1808	-7.84	296	Precursor

Identified peptide fragment **(1 site)**: QLEDGRTLSDYNIQK (Sequence: AA 49-63, Q62)

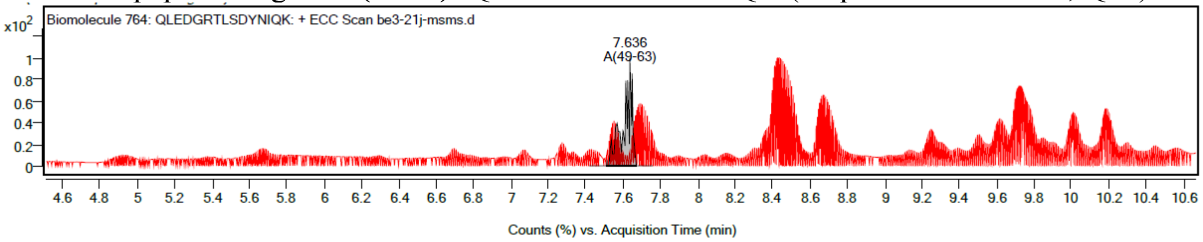

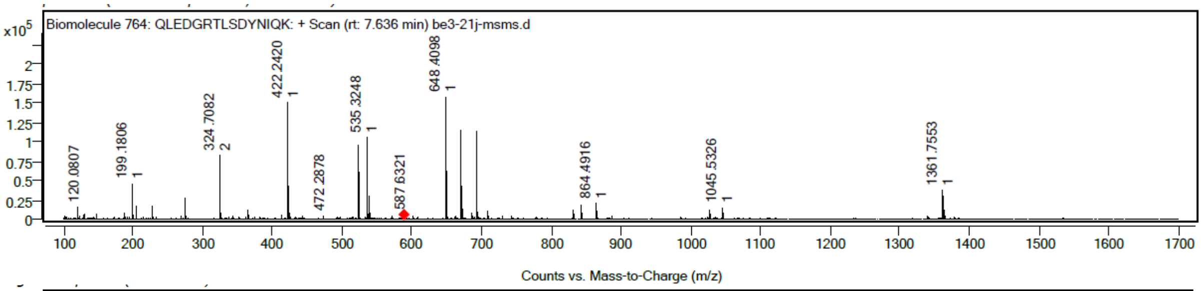

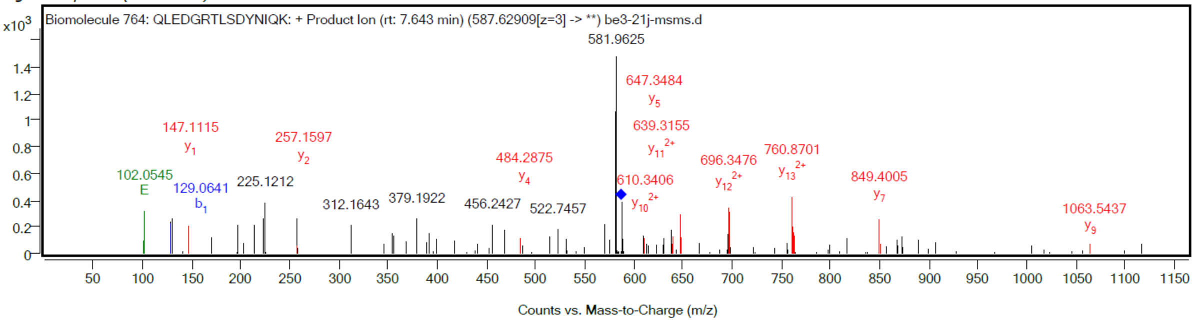

Fragment Spectrum Peaks

m/z	Diff (ppm)	Abund	Ion
147.1115	8.72	202	y1
257.1597	4.43	54	y2
484.2875	0.55	113	y4
647.3484	4.23	290	y5
849.4005	11.28	251	y7
1063.5437	-1.75	66	y9
610.3406	-25.36	114	y10
639.3155	34.40	126	y11
696.3476	2.49	342	y12
760.8701	0.70	423	y13
129.0641	13.85	225	b1
101.0709	0.74	92	Q
102.0545	4.58	316	E
587.9612	4.36	256	Precursor
588.2968	2.34	103	Precursor

Identified peptide fragment **(2 sites)**: TLSDYNIQK (Sequence: AA 55-63, [N60, Q62])

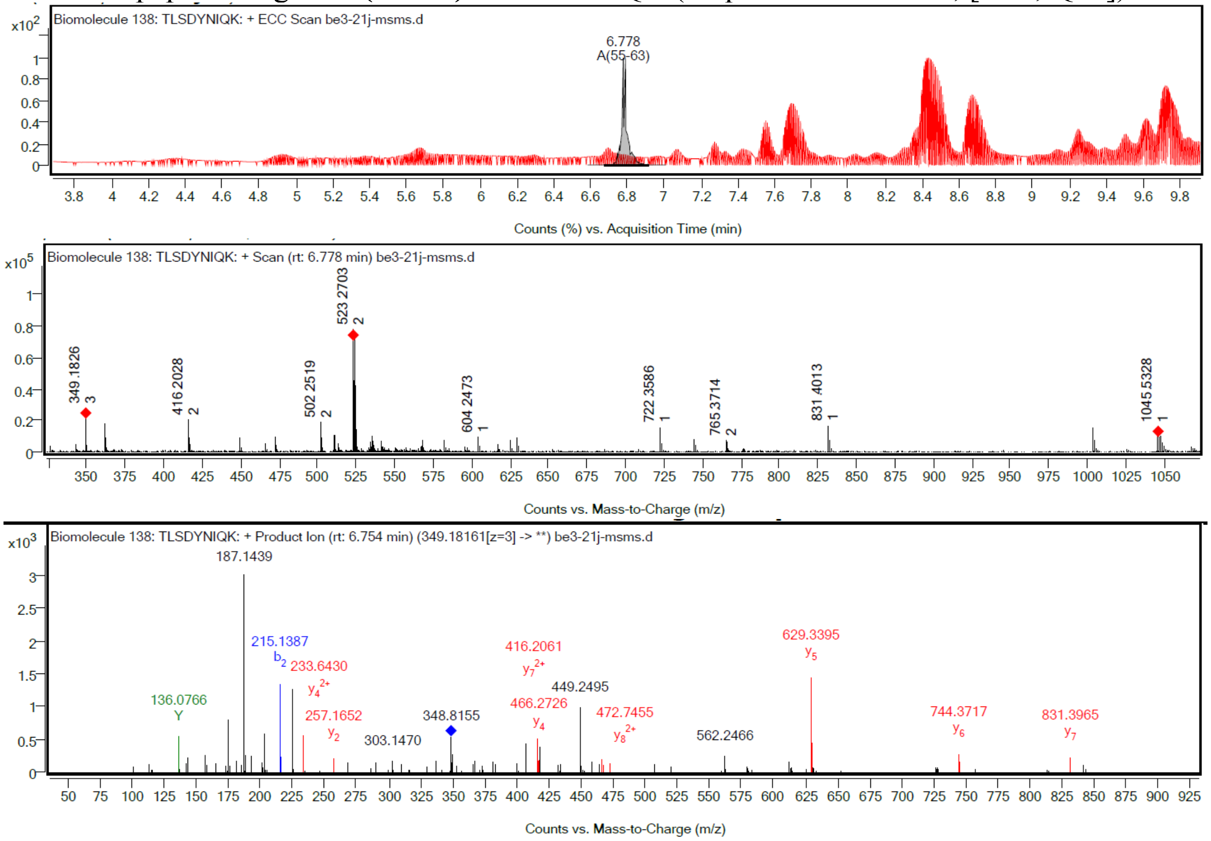

Fragment Spectrum Peaks

m/z	Diff (ppm)	Abund	Ion
257.1652	-16.89	199	y2
466.2726	10.01	192	y4
629.3395	1.69	1433	y5
744.3717	-5.67	263	y6
831.3965	3.69	211	y7
233.6430	-3.05	556	y4
416.2061	-6.40	497	y7
472.7455	-0.22	122	y8
215.1387	1.44	1334	b2
136.0766	-6.48	539	Y
349.1837	-4.96	73	Precursor
349.5176	-3.54	259	Precursor

ICP–MS Quantification of Residual Palladium

Palladium content in protein samples was quantified by inductively coupled plasma mass spectrometry (ICP–MS) using a PerkinElmer NexION 2000 instrument equipped with a standard cyclonic spray chamber and a Meinhard concentric nebulizer. The instrument was operated in standard mode, and daily autotuning was performed in accordance with the manufacturer's recommendations.

A commercial multi-element standard solution (SPEX CertiPrep, Multi-element Solution 3, Cat. No. CLMS-3; 10 μg/mL Pd in 10% HCl / 1% HNO_3) was used to prepare external calibration standards. An intermediate Pd stock (1,000 ng/mL) was generated by diluting the supplier solution 10-fold with 2% (v/v) HNO_3. Calibration standards (0, 1, 5, 10, 25, 50, and 100 ng/mL Pd) were subsequently prepared by serial dilution of the intermediate stock in 2% HNO_3. The calibration

diluent and procedural blank consisted of ultrapure Type I water acidified to 2% HNO_3 using double-distilled trace-metal–grade nitric acid.

For sample preparation, 1 mg of modified ubiquitin in 100 µL was denatured by addition of an equal volume of concentrated HNO_3 (100 µL) in 15 mL polypropylene tubes, followed by incubation at room temperature for 60 min to ensure complete release of bound Pd species. The digest was then diluted to a final volume of 5.0 mL with 2% HNO_3, resulting in an overall 25-fold dilution and a final acid concentration of ~2% HNO_3. Reagent blanks (2% HNO_3) were processed alongside all samples.

Samples, blanks, and standards were introduced via the autosampler, using a minimum volume of ~5 mL per tube to ensure consistent aspiration. Each solution was analyzed in triplicate. Mean count rates at m/z 105 were converted to concentrations using the external calibration curve ($R^2 \geq 0.999$). Blank-corrected Pd concentrations (ng/mL) in the diluted protein samples were back-calculated to the original protein samples based on the dilution factor.

Pd was quantified primarily using $^{105}Pd^+$, selected for its high natural abundance and minimal polyatomic interferences under helium KED conditions. Additional Pd isotopes ($^{106}Pd^+$, $^{108}Pd^+$, $^{104}Pd^+$, and $^{110}Pd^+$) were monitored to assess potential interferences. The final sample contained 8.78 ng/mL Pd based on $^{105}Pd^+$. The confirmation isotopes $^{106}Pd^+$ and $^{108}Pd^+$ yielded values within 3-5% of the primary isotope (8.89 and 9.20 ng/mL, respectively), indicating negligible spectral interference. Signals from $^{104}Pd^+$ and $^{110}Pd^+$ were excluded from quantitation due to known mass bias and polyatomic overlap under KED conditions.

Given a final sample volume of 5.0 mL containing 1 mg protein, the measured concentration corresponds to 43.9 ng residual Pd per 1 mg protein (43.9 ppm), or a molar Pd:protein ratio of 0.0036:1. Based on the initial reaction stoichiometry and amount of $Pd(OAc)_2$ used, this corresponds to a Pd removal efficiency exceeding 99.99% following cysteine/NaOH scavenging.

Supplementary Fig. 19: Nitrile Dehydration on Diverse Proteins

Based on optimization of nitrile formation on ubiquitin, 5 mM $Pd(OAC)_2$ was deemed the appropriate condition for application to diverse proteins, resulting in high conversion while minimizing the concentration of Pd.

Supplementary Fig. 19a: Scope of Nitrile Formation on Protein

General Procedure for Nitrile Formation on Protein (GP-XV):

The appropriate protein (2 mg) was dissolved in 800 µL of NaP buffer (10 mM, pH 7.4) and 200 µL of $Pd(OAc)_2$ (1.12 mg, 5 mM) dissolved in ACN was added. The reaction was stirred at room temperature for 2 hours followed by quenching with 500 µL of 1 M solution of aqueous L-cysteine and 10 µL of 1 M NaOH solution. The crude reaction mixture was passed through an Amicon™ Ultra 3 kDa centrifugal filter and washed with H_2O (7×0.5 mL) to remove the water-soluble Pd complex. The labeled protein was redissolved in 0.1% formic acid in H_2O and analyzed using LC-MS. Samples were additionally digested using SMART Digest™ Trypsin Kit by Thermo Scientific and analyzed by LC-MS/MS to determine the total number of nitrile-modified sites.

Nitrile Formation on Ribonuclease A

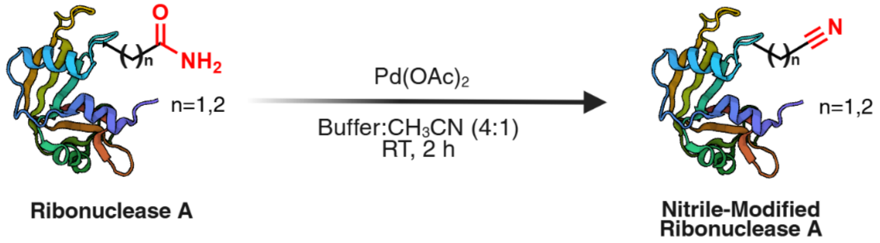

Reaction conducted according to **GP-XV**. Ribonuclease A (2 mg, 0.146 mM) was dissolved in 800 µL of NaP buffer (10 mM, pH 7.4) and 200 µL of $Pd(OAc)_2$ (1.12 mg, 5 mM) dissolved in ACN was added. The reaction was stirred at room temperature for 2 h followed by quenching with 500 µL of 1 M solution of aqueous L-cysteine and 10 µL of 1 M NaOH solution. The crude reaction mixture was passed through an Amicon™ Ultra 3 kDa centrifugal filter and washed with H_2O (7×0.5 mL) to remove the water-soluble Pd complex. The labeled protein was redissolved in 0.1% formic acid in H_2O and analyzed using LC-MS, revealing >95% conversion to nitrile (see table below). Samples were additionally digested using SMART Digest™ Trypsin Kit by Thermo Scientific and analyzed by LC-MS/MS, showing 9 nitrile-modified sites.

Modification	Mass	Conversion
Unmodified	13682.5	n.d.
1 Nitrile	13665.3 (**-18**)	24%
2 Nitrile	13646.8 (**-18**)	37%
≥3 Nitrile	13628.7 (**-18**)…	39%

MS of Unmodified Ribonuclease A

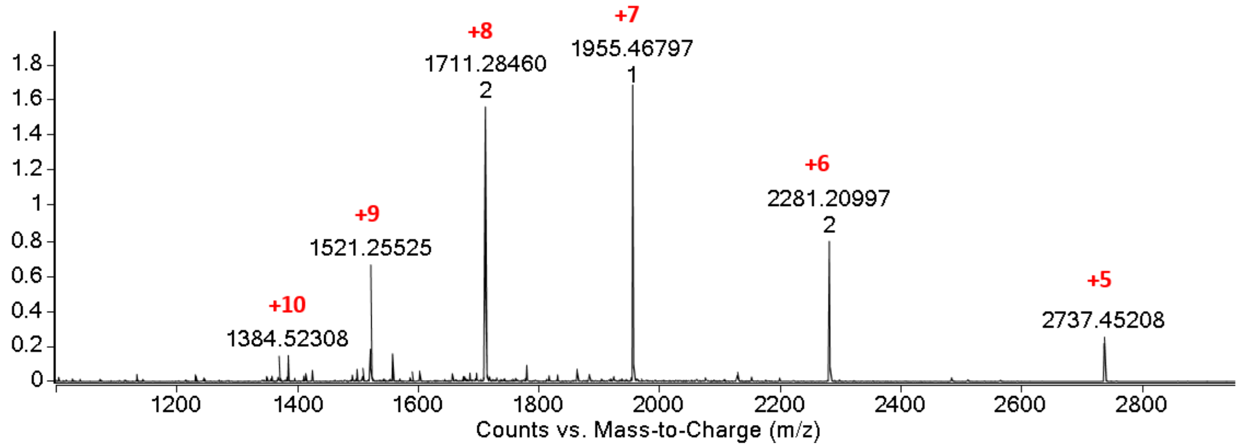

Deconvoluted MS of Unmodified Ribonuclease A

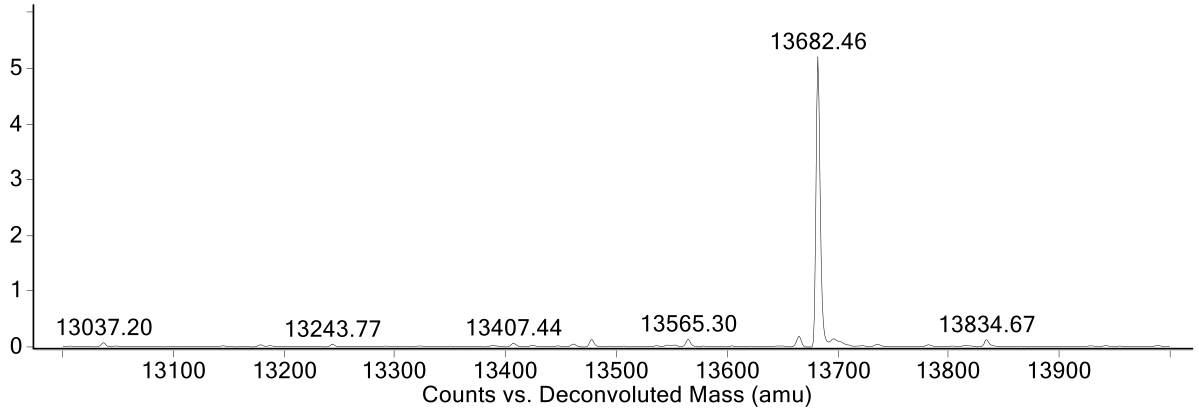

MS of Modified Ribonuclease A

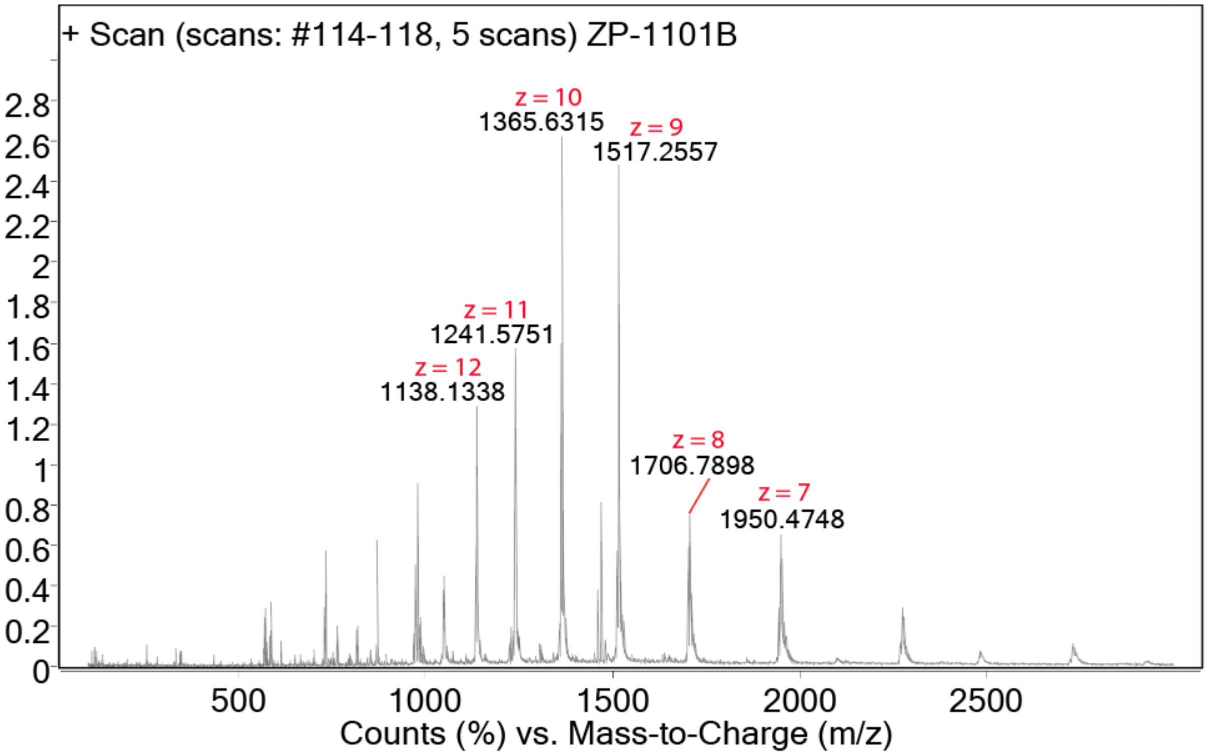

Deconvoluted MS of Modified Ribonuclease A

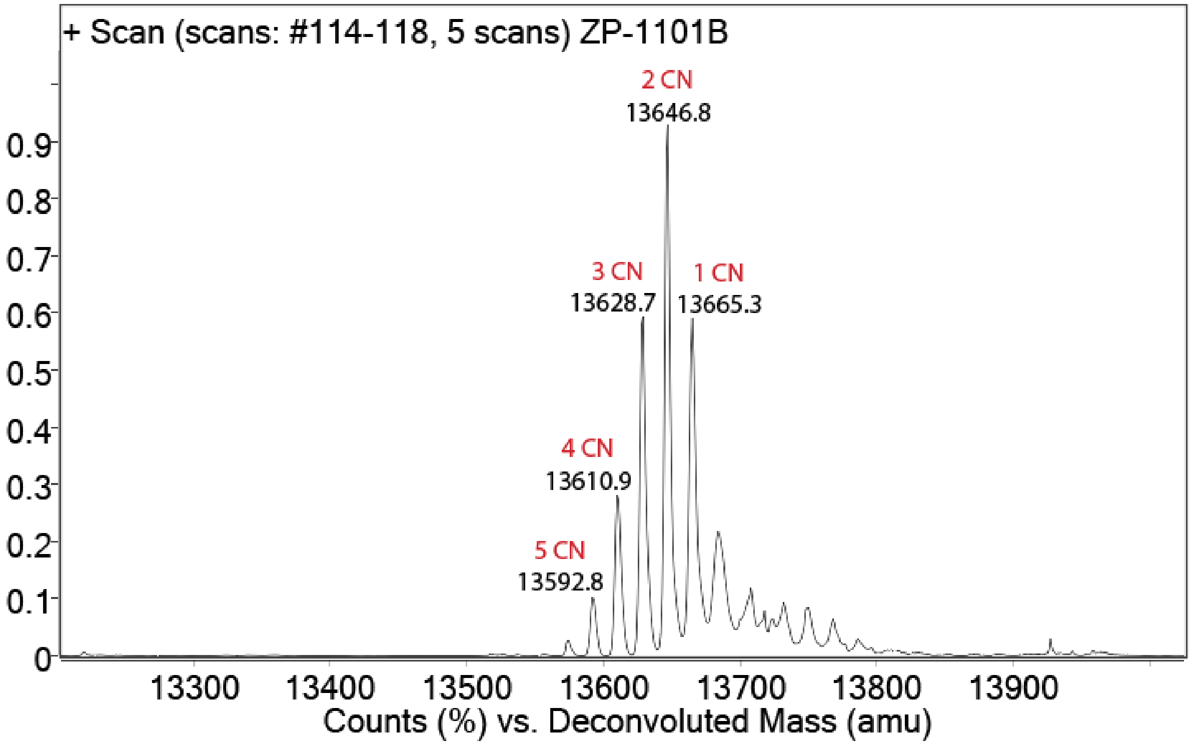

MS/MS Analysis of Digested Modified Ribonuclease A

Identified peptide fragment **(1 site)**: QHMDSSTSAASSSNYCNQMMK (Sequence: AA 11-31, Q11)

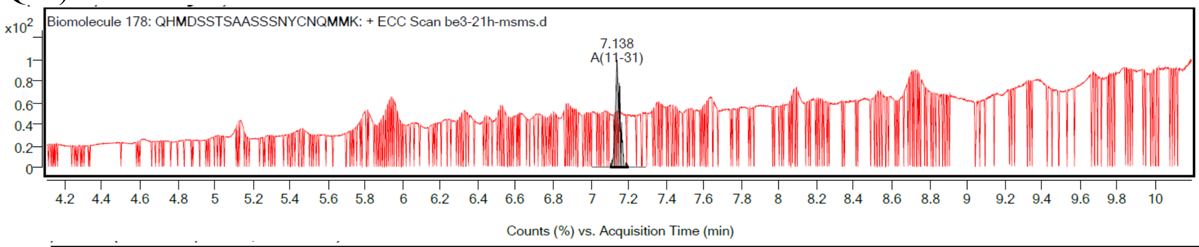

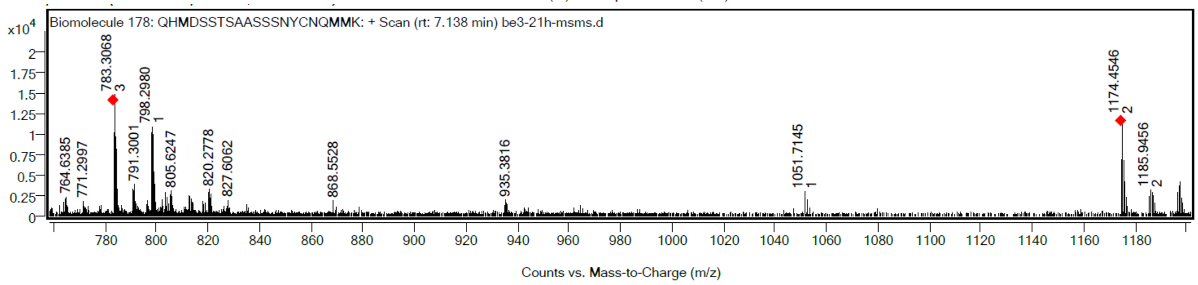

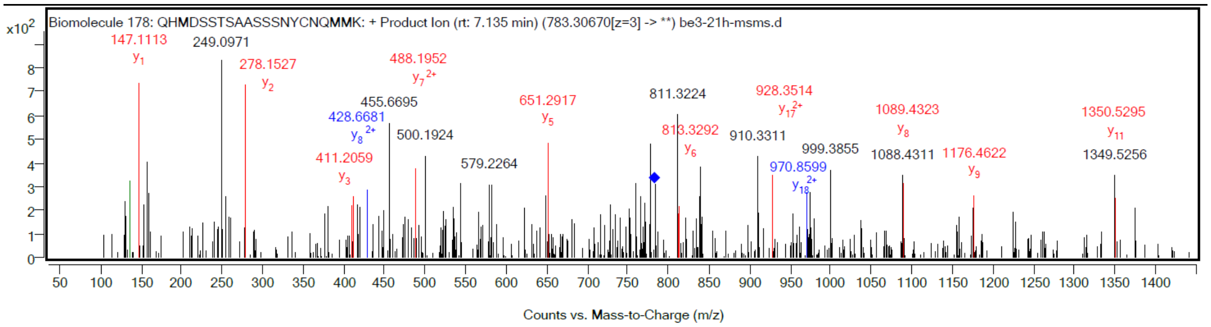

Fragment Spectrum Peaks

m/z	Diff (ppm)	Abund	Ion
147.1113	10.12	738	y1
278.1527	1.99	729	y2
411.2059	-13.11	256	y3
651.2917	5.53	483	y5
813.3292	-19.51	216	y6
1089.4323	-14.81	314	y8
1176.4622	-11.84	262	y9
1350.5295	-12.78	251	y11
488.1952	-10.01	375	y7
928.3514	20.56	348	y17
428.6681	-4.08	287	b8
970.8599	4.57	276	b18
136.0760	-2.60	324	Y
783.6483	-9.54	150	Precursor

Identified peptide fragment **(3 sites)**: QHMDSSTSAASSSNYCNQMMK (Sequence: AA 11-31, [Q11, N24, Q28])

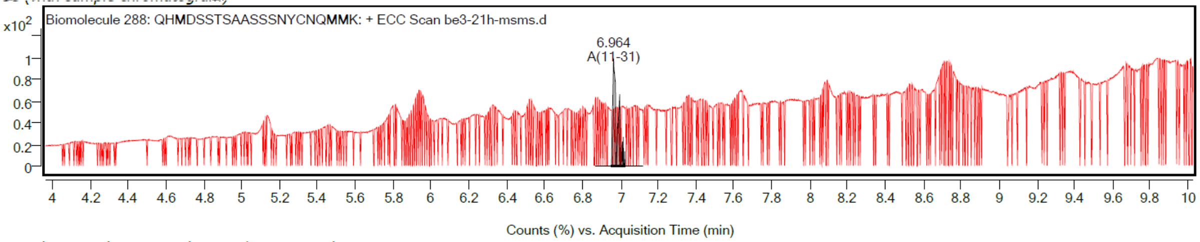

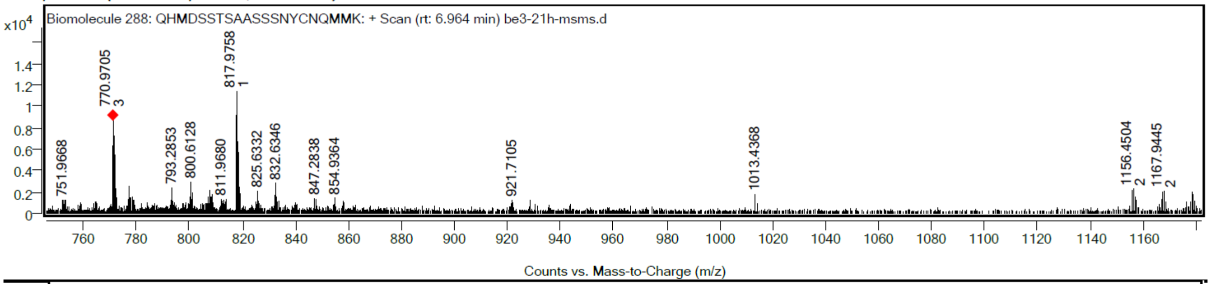

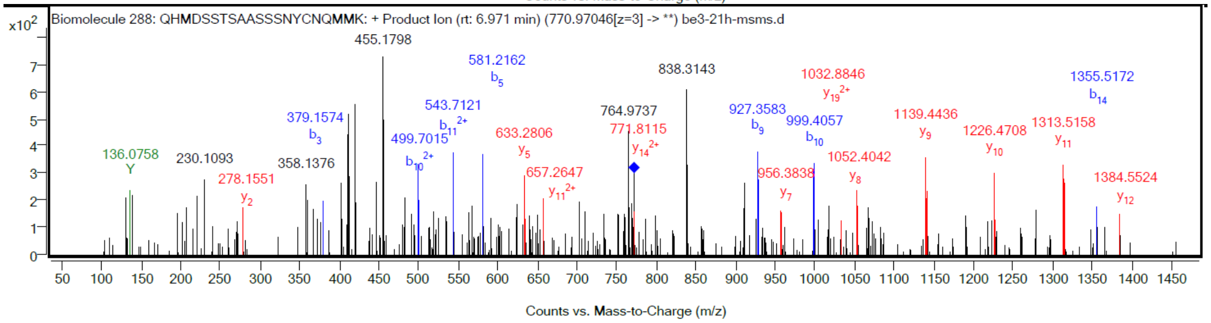

m/z	Fragment Spectrum Peaks<br>Diff (ppm)	Abund	Ion
278.1551	-6.42	171	y2
633.2806	6.54	289	y5
956.3838	-5.39	160	y7
1052.4042	6.51	235	y8
1139.4436	-0.50	357	y9
1226.4708	3.54	298	y10
1313.5158	-6.60	328	y11
1384.5524	-5.89	147	y12
657.2647	-11.34	203	y11
771.8115	-1.52	156	y14
1032.8846	16.46	124	y19
379.1574	-7.19	196	b3
581.2162	-4.29	368	b5
927.3583	4.54	377	b9
999.4057	-2.73	333	b10
1355.5172	8.05	174	b14
499.7015	3.88	331	b10
543.7121	16.60	373	b11
136.0758	-0.74	236	Y

Identified peptide fragment **(2 sites)**: CKPVNTFVHESLADVQAVCSQK (Sequence: AA 40-61, [N44, Q55])

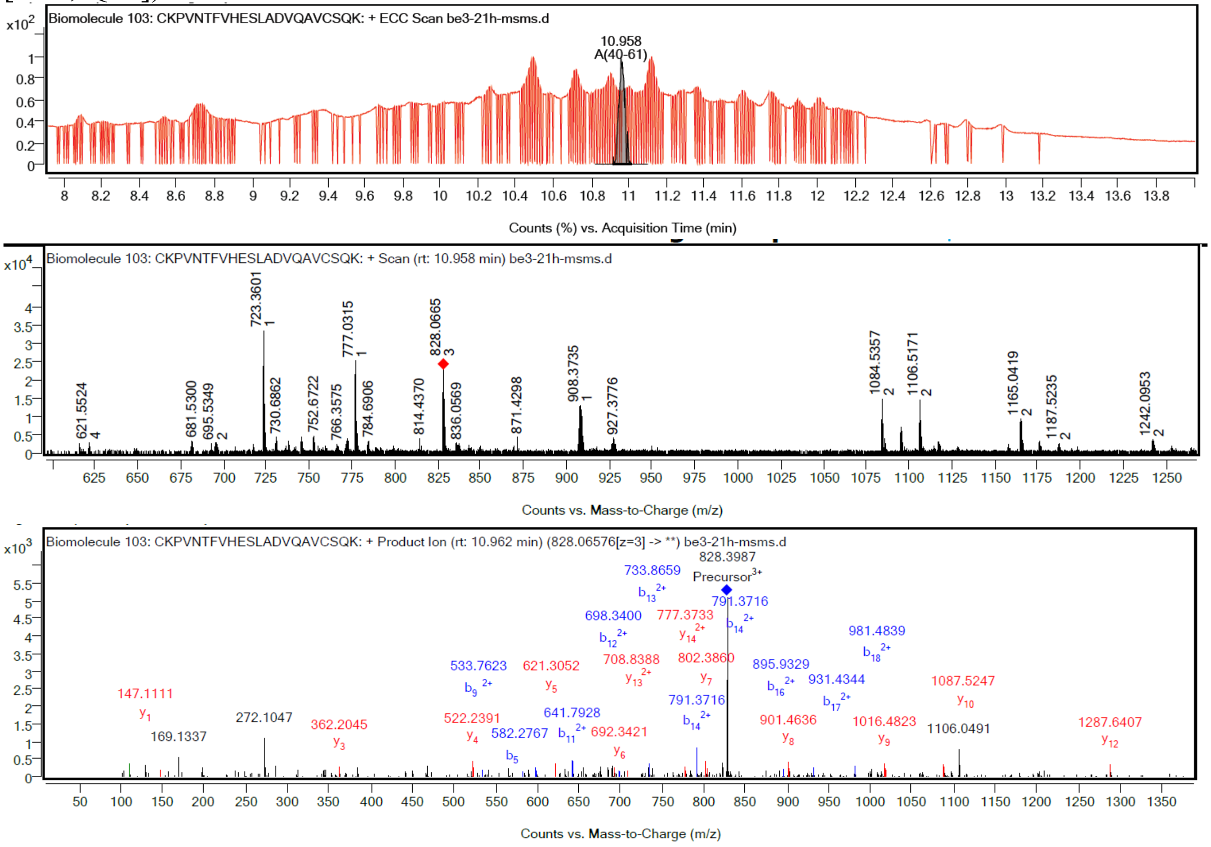

Fragment Spectrum Peaks

m/z	Diff (ppm)	Abund	Ion
147.1111	11.44	178	y1
362.2045	-3.14	266	y3
522.2391	-9.70	440	y4
621.3052	-4.35	367	y5
692.3421	-3.66	261	y6
802.3860	2.01	430	y7
901.4636	-8.39	402	y8
1016.4823	0.66	360	y9
1087.5247	-4.27	334	y10
1287.6407	-3.49	336	y12
708.8388	6.01	161	y13
777.3733	-1.05	274	y14
582.2767	-10.80	146	b5
533.7623	-3.21	180	b9
598.2817	0.33	252	b10
641.7928	7.94	466	b11
698.3400	-0.13	167	b12
733.8659	-10.18	371	b13
791.3716	0.44	826	b14
895.9329	-3.09	215	b16
931.4344	15.36	253	b17
981.4839	0.70	289	b18
110.0704	8.39	369	H
828.0650	1.96	2723	Precursor
828.3987	2.82	5080	Precursor
828.7330	3.05	4132	Precursor
829.0717	-2.02	857	Precursor

Identified peptide fragment **(2 sites)**: NGQTNCYQSYSTMSITDCR (Sequence: AA 67-85, [N71, Q74])

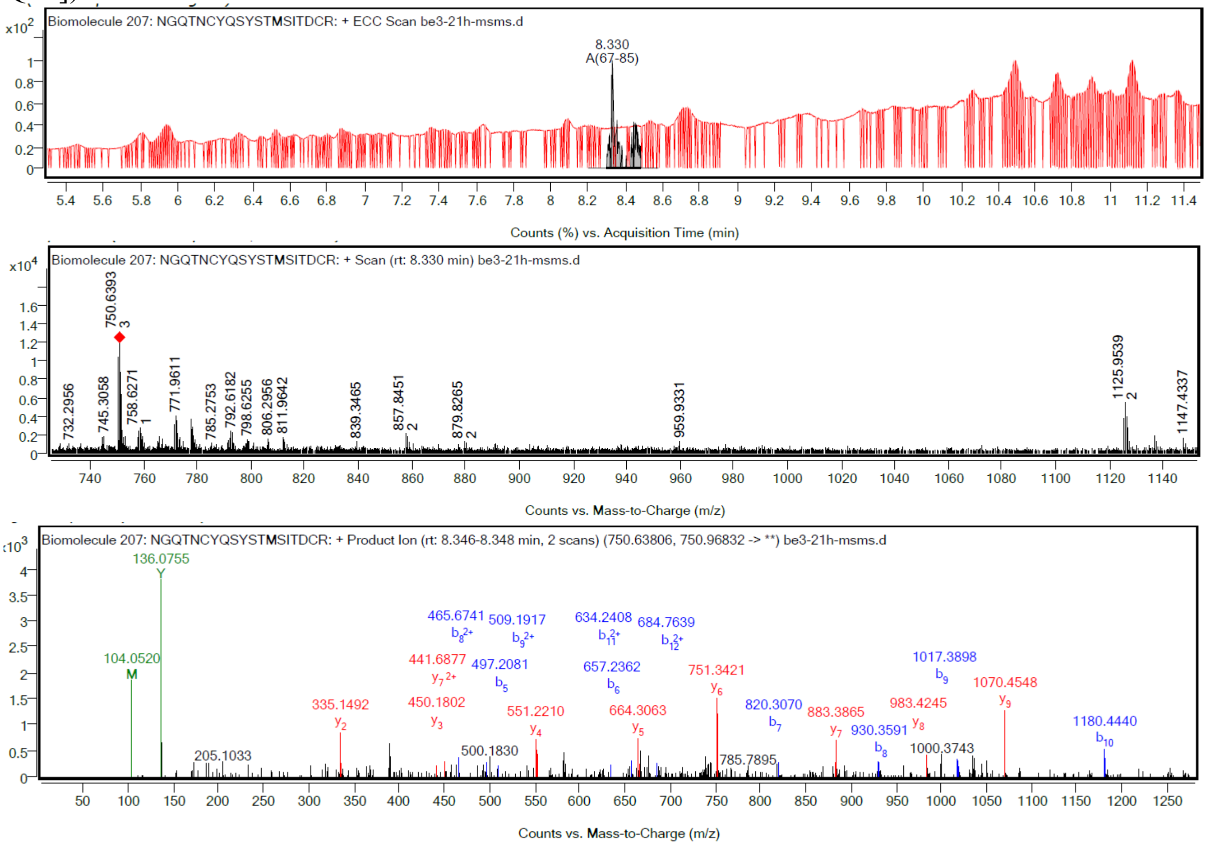

Fragment Spectrum Peaks

m/z	Diff (ppm)	Abund	Ion
335.1492	1.30	840	y2
450.1802	-8.06	283	y3
551.2210	5.94	707	y4
664.3063	3.05	734	y5
751.3421	-2.39	1511	y6
883.3865	-2.69	692	y7
983.4245	4.03	414	y8
1070.4548	5.30	1269	y9
441.6877	14.32	202	y7
497.2081	4.44	273	b5
657.2362	7.27	293	b6
820.3070	-3.39	259	b7
930.3591	-7.32	278	b8
1017.3898	-5.43	325	b9
1180.4440	3.09	518	b10
465.6741	12.16	359	b8
509.1917	8.12	200	b9
634.2408	4.29	213	b11
684.7639	4.99	247	b12
104.0520	8.34	1858	M
136.0755	1.47	3794	Y

Identified peptide fragment **(1 site)**: YPNCAYK (Sequence: AA 92-98, N94)

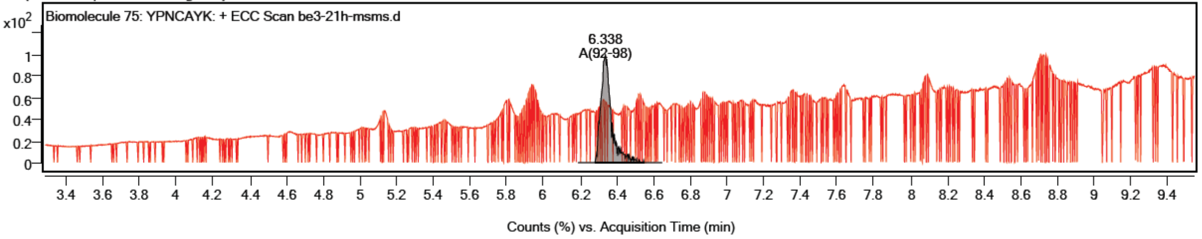

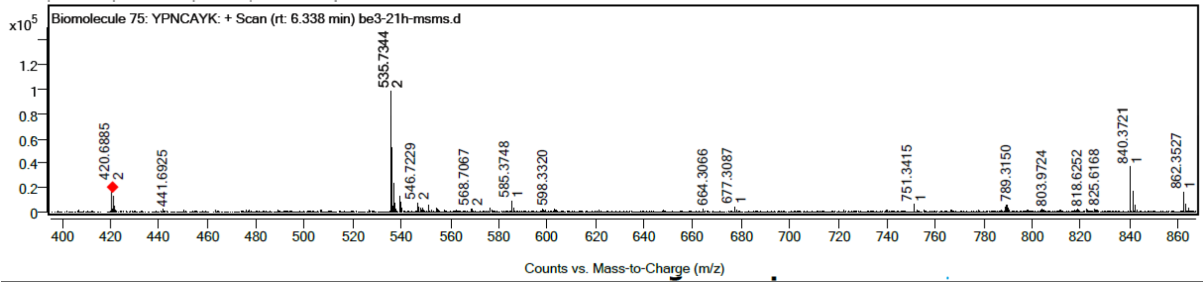

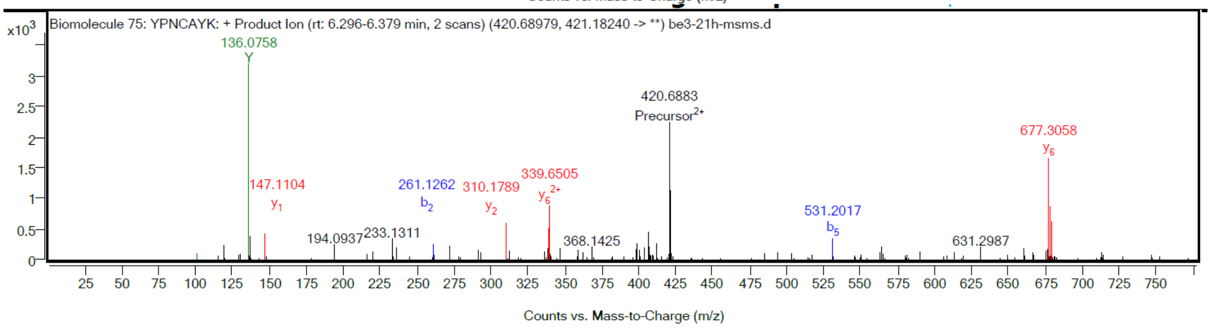

Fragment Spectrum Peaks

m/z	Diff (ppm)	Abund	Ion
147.1104	16.64	409	y1
310.1789	-8.91	594	y2
677.3058	2.61	1648	y6
339.6505	25.35	864	y6
261.1262	-10.86	233	b2
531.2017	0.66	330	b5
101.1060	13.50	94	K
136.0758	-0.51	3209	Y
420.6883	1.97	2248	Precursor
421.1859	11.43	1120	Precursor
421.6890	8.11	480	Precursor

Identified peptide fragment **(1 site)**: HIIVACEGNPYVPVHFDASV (Sequence: AA 105-124, N113)

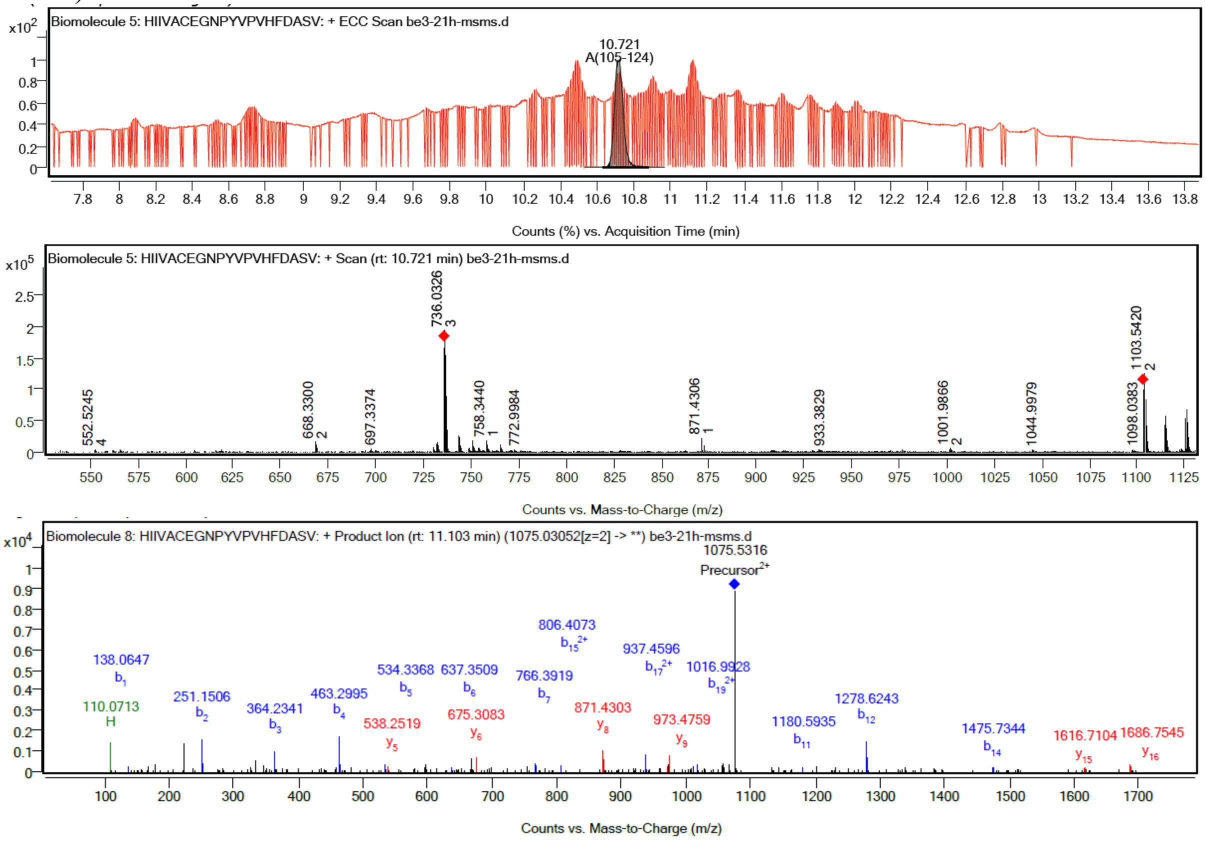

Fragment Spectrum Peaks

m/z	Diff (ppm)	Abund	Ion
538.2519	-2.18	219	y5
675.3083	2.04	644	y6
871.4303	0.64	997	y8
973.4759	34.29	749	y9
1616.7104	8.61	161	y15
1686.7545	2.11	323	y16
138.0647	11.12	231	b1
251.1506	-1.48	1568	b2
364.2341	0.64	977	b3
463.2995	6.87	1701	b4
534.3368	5.74	304	b5
637.3509	-3.00	169	b6
766.3919	-0.32	360	b7
1180.5935	-24.25	153	b11
1278.6243	4.45	1463	b12
1475.7344	13.63	194	b14
806.4073	1.69	277	b15
937.4596	-3.45	816	b17
1016.9928	-0.19	307	b19
110.0713	0.02	1411	H
1075.0289	1.37	7165	Precursor
1075.5316	0.47	8858	Precursor
1076.0310	2.58	2570	Precursor

Nitrile Formation on Lysozyme Human

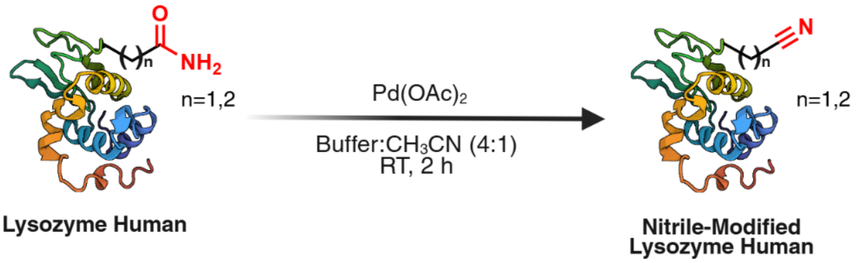

Reaction conducted according to **GP-XV**. Lysozyme Human (2 mg, 0.136 mM) was dissolved in 800 µL of NaP buffer (10 mM, pH 7.4) and 200 µL of $Pd(OAc)_2$ (1.12 mg, 5 mM) dissolved in ACN was added. The reaction was stirred at room temperature for 2 h followed by quenching with 500 µL of 1 M solution of aqueous L-cysteine and 10 µL of 1 M NaOH solution. The crude reaction mixture was passed through an Amicon™ Ultra 3 kDa centrifugal filter and washed with H_2O (7×0.5 mL) to remove the water-soluble Pd complex. The labeled protein was redissolved in 0.1% formic acid in H_2O and analyzed using LC-MS, revealing 84% conversion to nitrile (see table below). Samples were additionally digested using SMART Digest™ Trypsin Kit by Thermo Scientific and analyzed by LC-MS/MS, showing 9 nitrile-modified sites.

Modification	Mass	Conversion
Unmodified	14693.4	16%
1 Nitrile	14675.3 (**-18**)	41%
2 Nitrile	14657.3 (**-18**)	36%
3 Nitrile	14639.0 (**-18**)	7%

MS of Unmodified Lysozyme Human

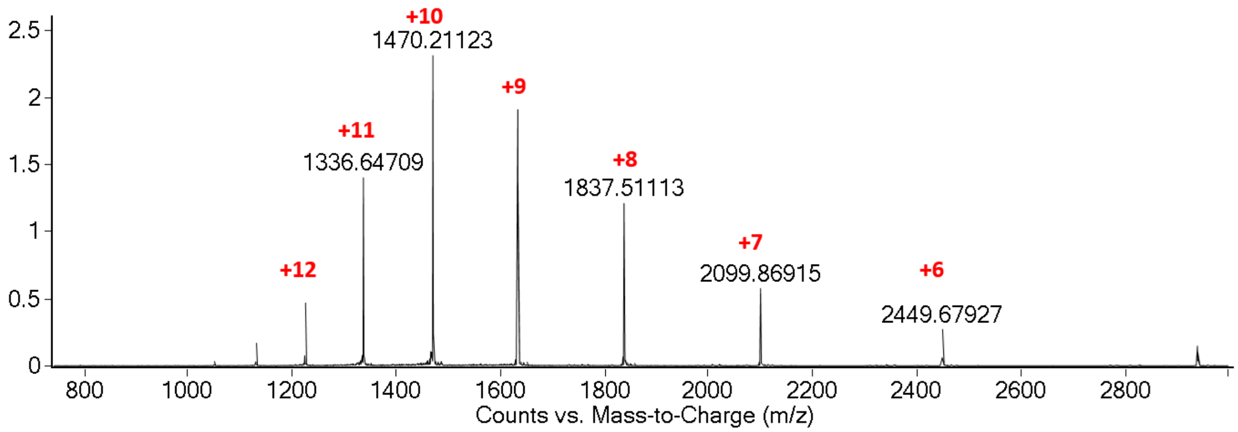

Deconvoluted MS of Unmodified Lysozyme Human

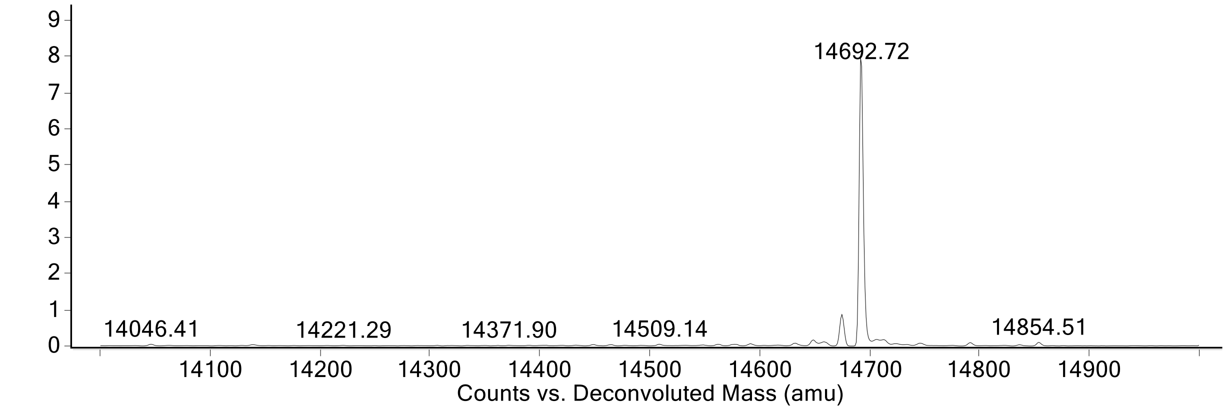

MS of Modified Lysozyme Human

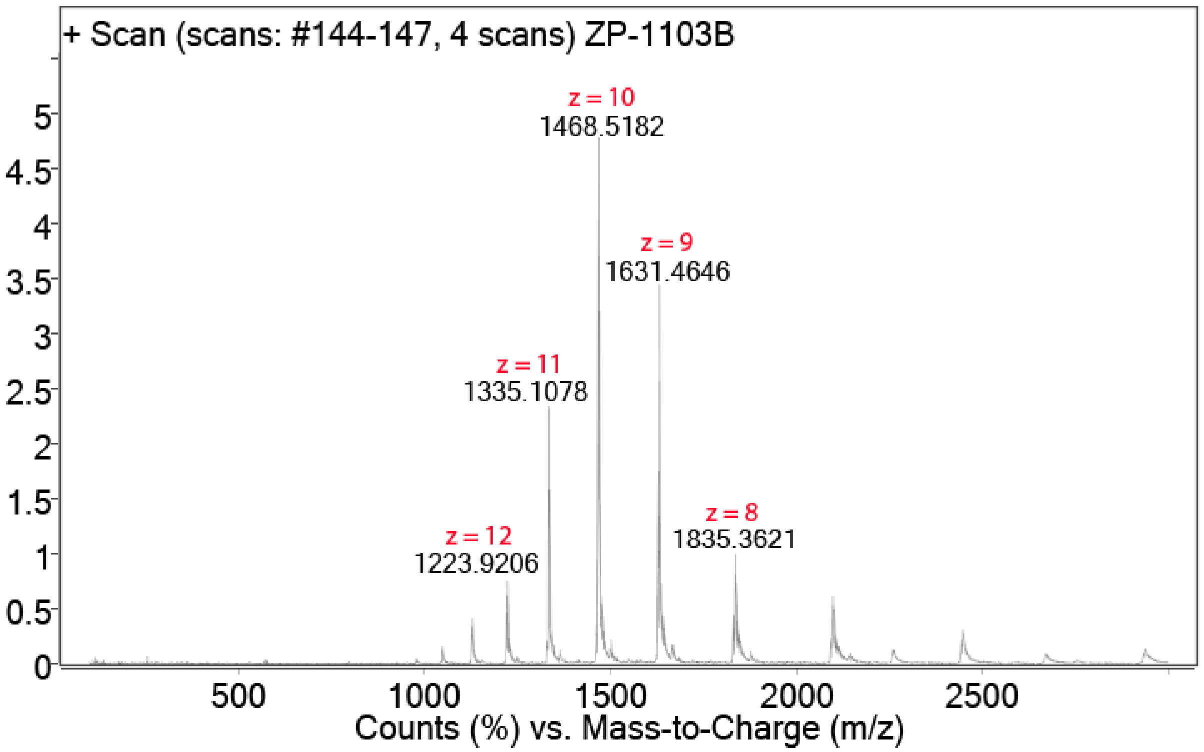

Deconvoluted MS of Modified Lysozyme Human

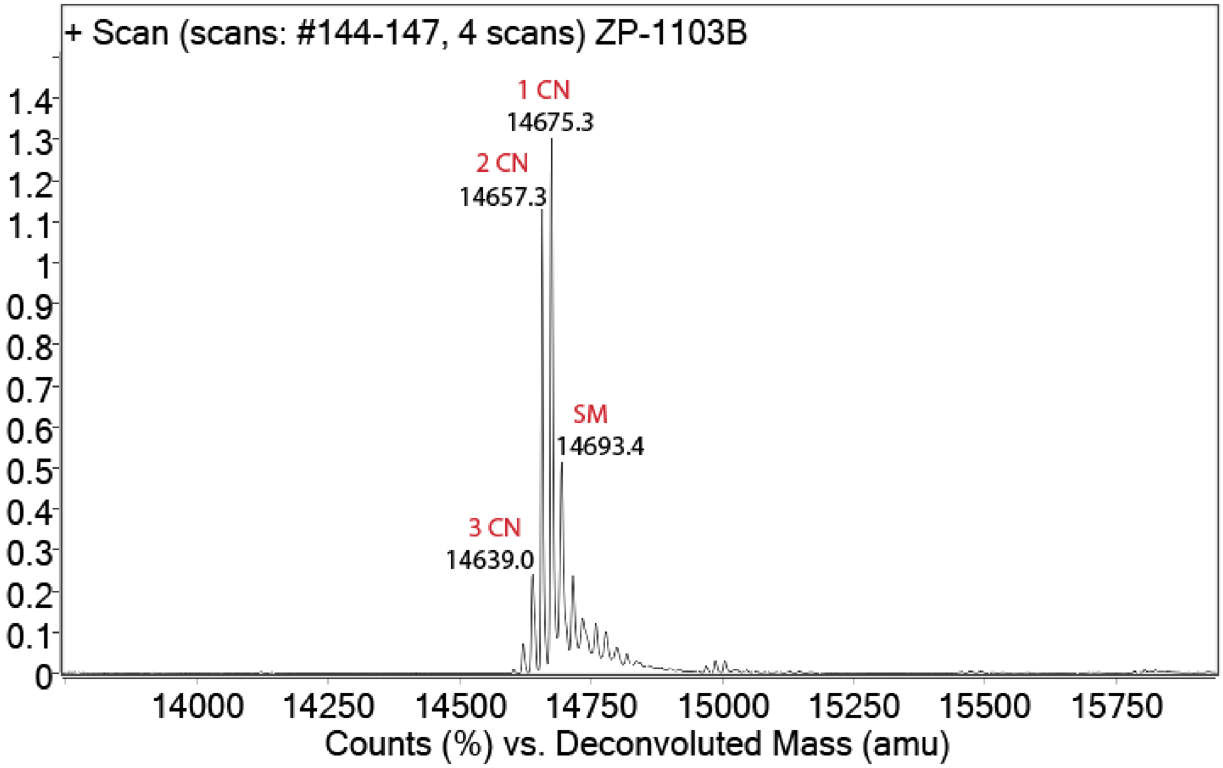

MS/MS Analysis of Digested Modified Lysozyme Human

Identified peptide fragment **(1 site)**: GISLANWMCLAK (Sequence: AA 22-33, N27)

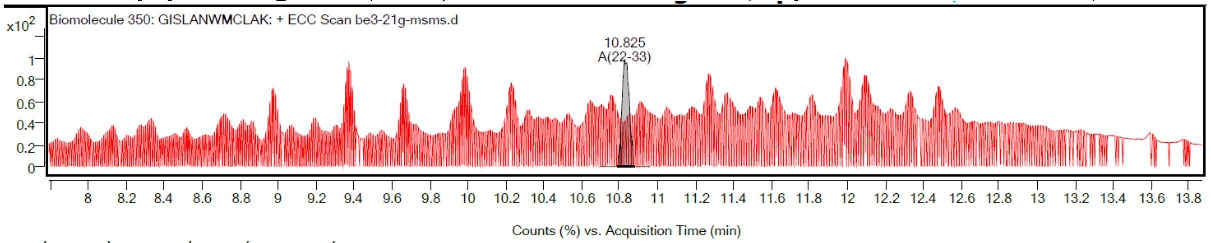

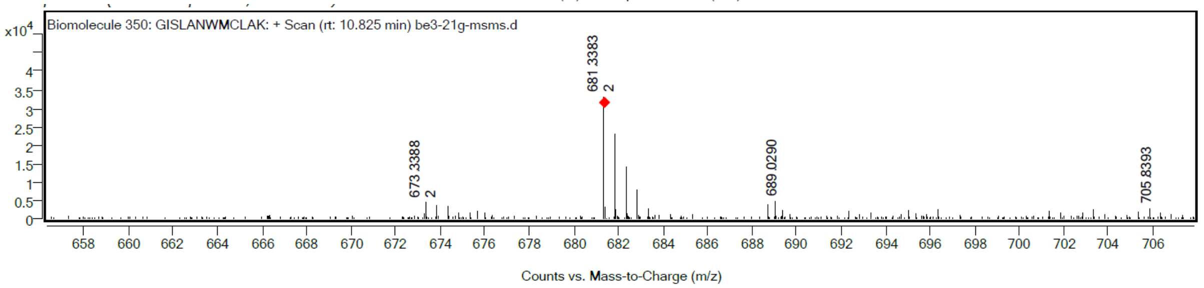

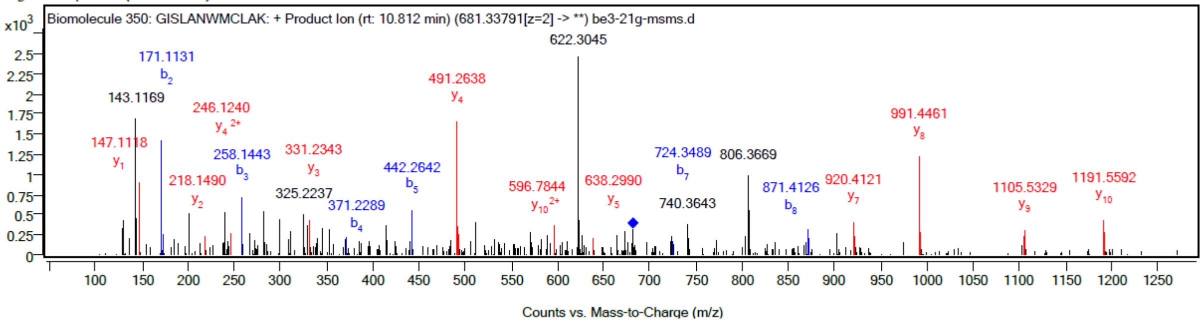

Fragment Spectrum Peaks

m/z	Diff (ppm)	Abund	Ion
147.1118	6.96	899	y1
218.1490	3.98	223	y2
331.2343	-0.87	427	y3
491.2638	1.60	1657	y4
638.2990	1.55	205	y5
920.4121	-0.47	404	y7
991.4461	2.74	1219	y8
1105.5329	3.06	307	y9
1191.5592	4.83	421	y10
246.1240	48.70	267	y4
596.7844	5.59	361	y10
171.1131	-1.53	1419	b2
258.1443	2.23	710	b3
371.2289	0.06	219	b4
442.2642	4.16	545	b5
724.3489	39.72	169	b7
871.4126	0.54	316	b8
681.3408	-2.83	309	Precursor

Identified peptide fragment **(1 site)**: ATNYNAGDR (Sequence: AA 42-50, N44)

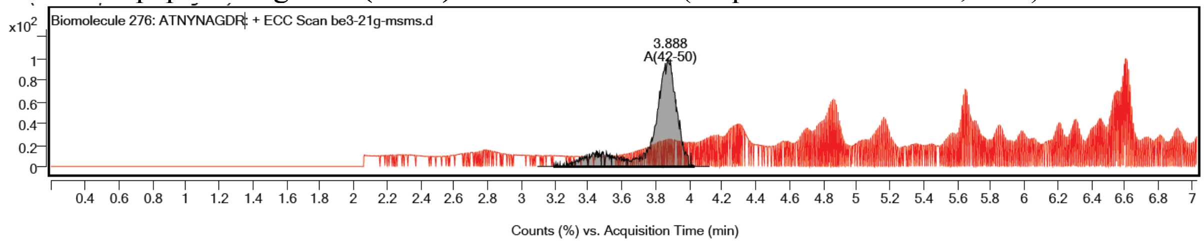

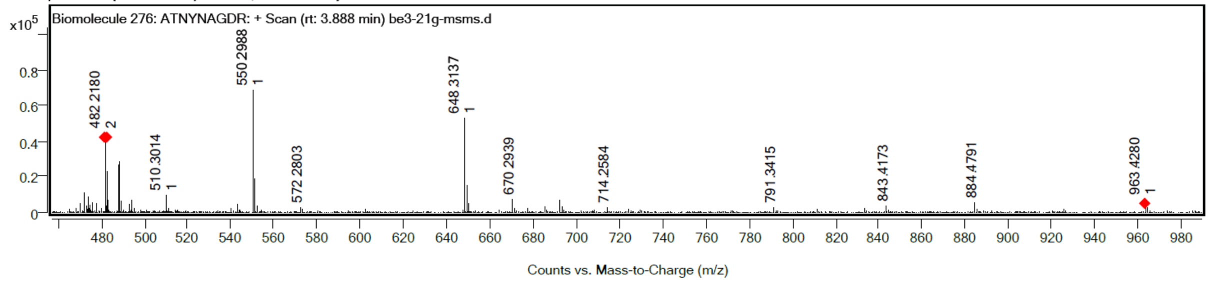

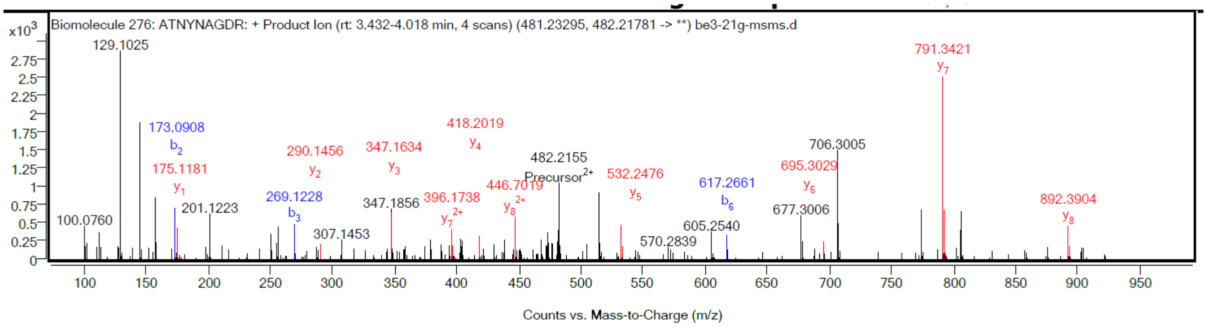

Fragment Spectrum Peaks

m/z	Diff (ppm)	Abund	Ion
175.1181	4.92	418	y1
290.1456	0.88	200	y2
347.1634	11.52	628	y3
418.2019	6.18	313	y4
532.2476	-0.28	452	y5
695.3029	11.30	231	y6
791.3421	1.28	2498	y7
892.3904	0.47	439	y8
396.1738	3.56	396	y7
446.7019	-6.34	564	y8
173.0908	7.34	689	b2
269.1228	6.20	467	b3
617.2661	2.82	318	b6
482.2155	4.22	1041	Precursor
482.7181	2.36	511	Precursor

Identified peptide fragment **(2 sites)**: STDYGIFQINSR (Sequence: AA 51-62, [Q58, N60])

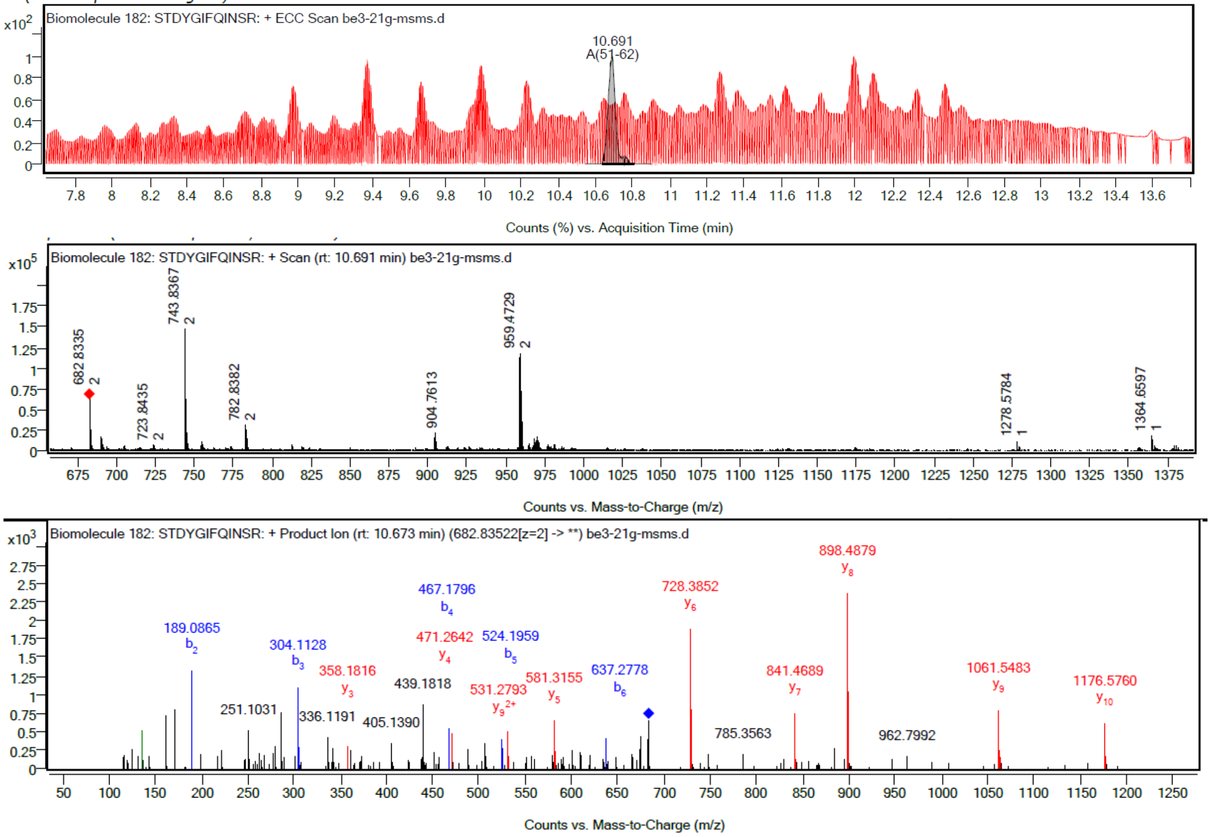

Fragment Spectrum Peaks

m/z	Diff (ppm)	Abund	Ion
358.1816	4.74	297	y3
471.2642	6.73	460	y4
581.3155	-0.12	636	y5
728.3852	-1.94	1884	y6
841.4689	-1.13	739	y7
898.4879	1.60	2362	y8
1061.5483	4.16	779	y9
1176.5760	3.12	604	y10
531.2793	1.26	487	y9
189.0865	2.32	1312	b2
304.1128	3.55	1088	b3
467.1796	-4.93	539	b4
524.1959	5.46	386	b5
637.2778	7.76	403	b6
136.0757	0.03	501	Y
682.8358	-3.59	385	Precursor
683.3316	4.91	646	Precursor

Identified peptide fragment **(1 site)**: YWCNDGK (Sequence: AA 63-69, N66)

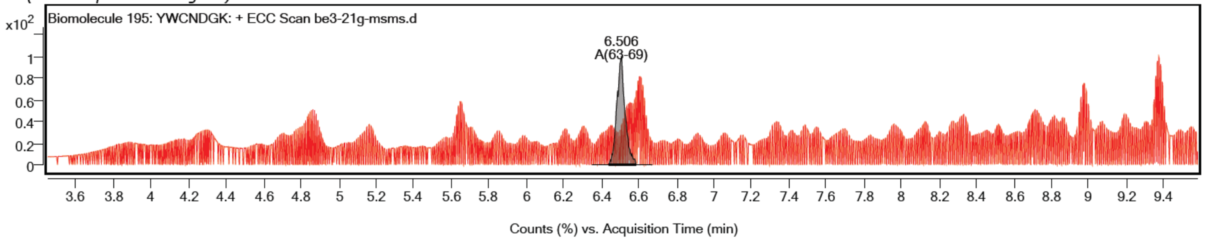

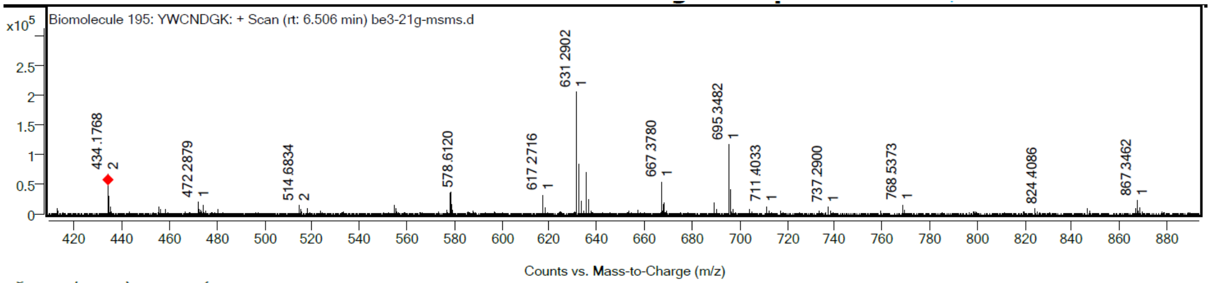

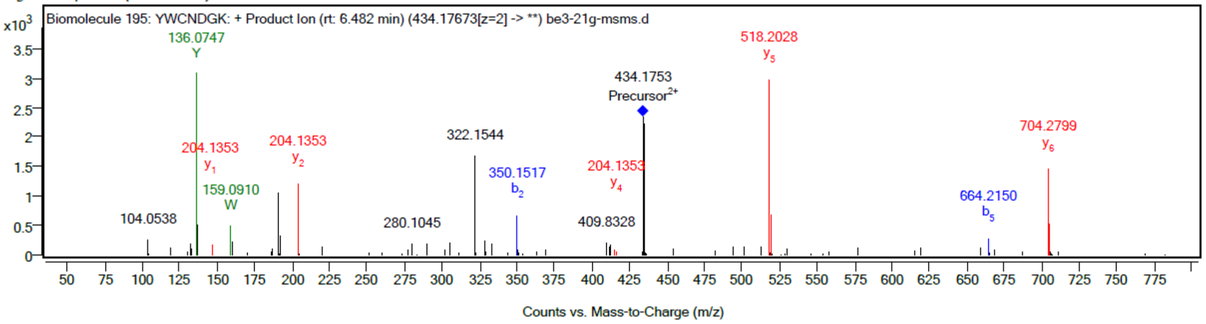

Fragment Spectrum Peaks

m/z	Diff (ppm)	Abund	Ion
147.1145	-11.67	177	y1
204.1353	-4.97	1200	y2
415.1948	-3.05	80	y4
518.2028	-0.14	2959	y5
704.2799	3.02	1448	y6
350.1517	-5.09	667	b2
664.2150	5.11	273	b5
136.0747	7.08	3081	Y
159.0910	4.09	490	W
434.1753	2.34	2328	Precursor
434.6774	1.33	2223	Precursor

Identified peptide fragment **(1 site)**: TPGAVNACHLSC (Sequence: AA 70-81, N75)

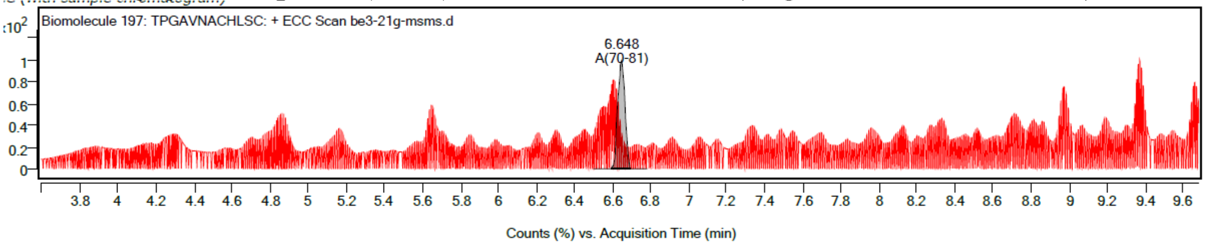

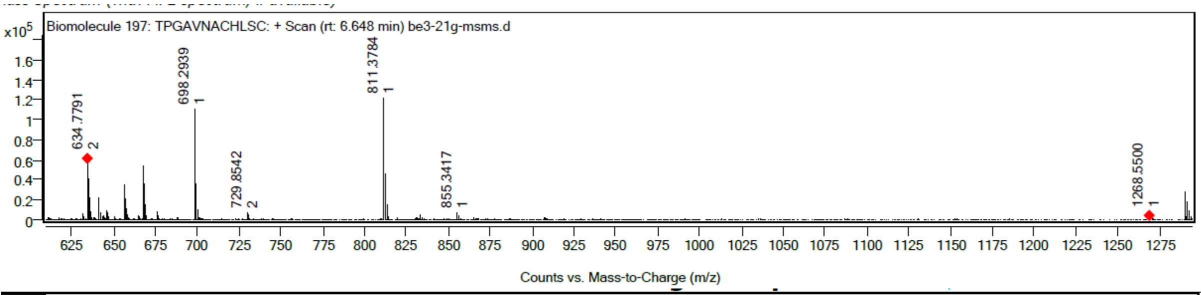

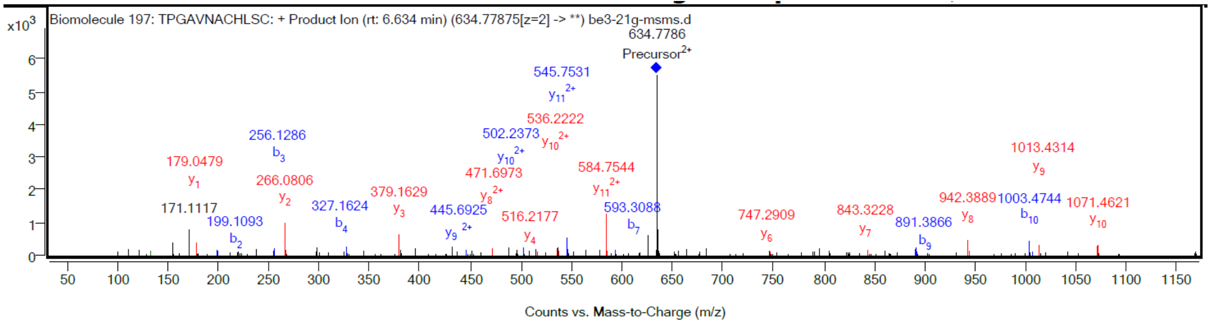

Fragment Spectrum Peaks

m/z	Diff (ppm)	Abund	Ion
179.0479	3.50	373	y1
266.0806	-0.37	978	y2
379.1629	4.54	618	y3
516.2177	11.22	87	y4
747.2909	0.52	83	y6
843.3228	0.97	149	y7
942.3889	3.36	434	y8
1013.4314	-2.20	302	y9
1071.4621	-7.56	286	y10
471.6973	4.89	181	y8
536.2222	15.64	203	y10
584.7544	4.51	1239	y11
199.1093	-8.02	133	b2
256.1286	2.44	205	b3
327.1624	11.94	251	b4
593.3088	-7.79	142	b7
891.3866	11.77	225	b9
1003.4744	3.38	417	b10
445.6925	18.08	134	b9
502.2373	10.41	210	b10
545.7531	10.07	531	b11
133.0420	7.97	129	C
634.7786	0.97	5510	Precursor
635.2795	2.05	4236	Precursor
635.7809	2.59	777	Precursor

Identified peptide fragment **(2 sites)**: GAVNACHLSCSALLQDNIADAVACAK (Sequence: AA 72-97, [Q86, N88])

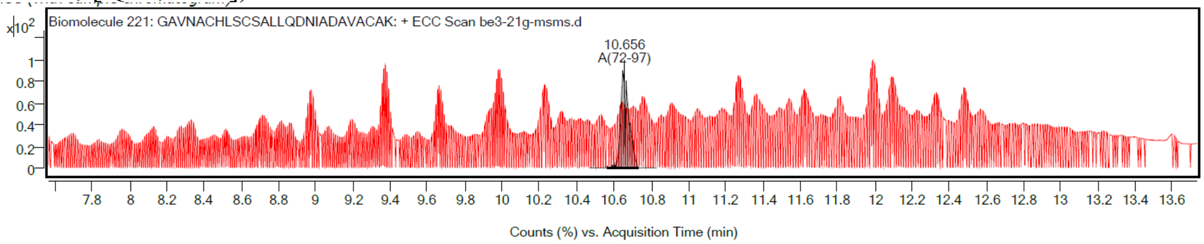

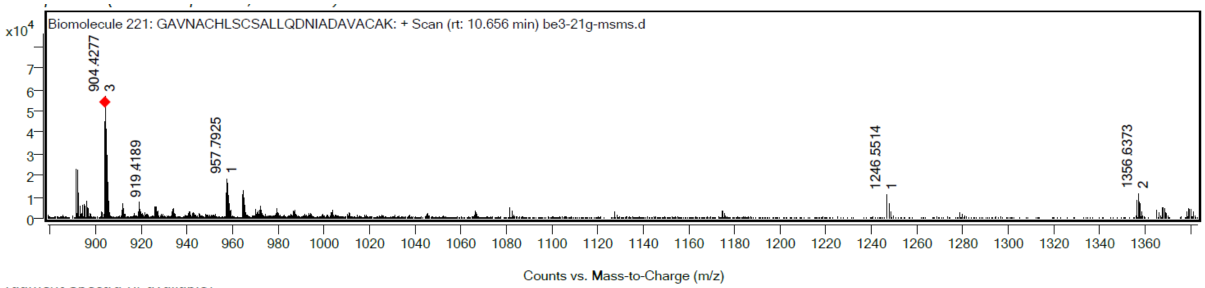

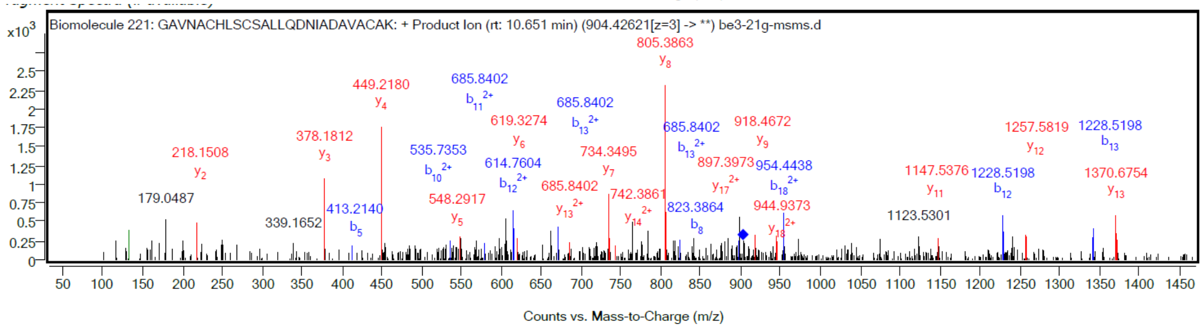

m/z	Diff (ppm)	Abund	Ion
218.1508	-4.09	489	y2
378.1812	-1.80	1068	y3
449.2180	-0.81	1756	y4
548.2917	-10.27	299	y5
619.3274	-6.85	284	y6
734.3495	0.83	862	y7
805.3863	1.16	2312	y8
918.4672	4.55	323	y9
1147.5376	3.15	277	y11
1257.5819	5.86	323	y12
1370.6754	-1.53	581	y13
685.8402	0.15	229	y13
742.3861	-5.17	184	y14
944.9373	11.55	308	y18
413.2140	0.73	181	b5
823.3864	1.81	261	b8
1228.5198	-0.01	581	b12
1342.6004	5.06	414	b13
535.7353	-11.78	250	b10
579.2611	-27.88	211	b11
614.7604	5.09	654	b12
671.3064	-1.32	430	b13
897.3973	10.23	246	b17
954.4438	6.71	622	b18
133.0439	-6.40	391	C

Identified peptide fragment **(1 site)**: VVRDPQGIR (Sequence: AA 99-107, Q104)

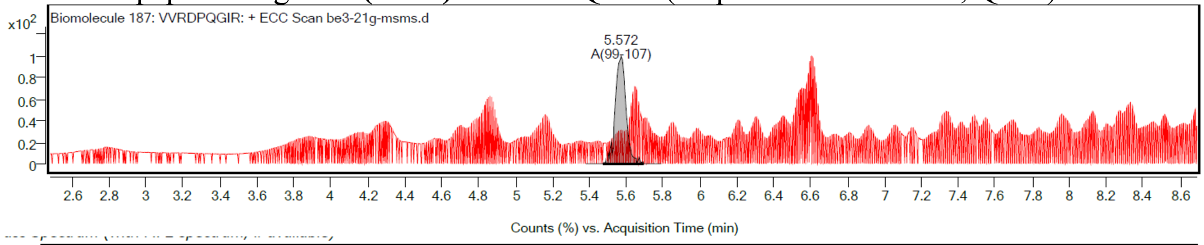

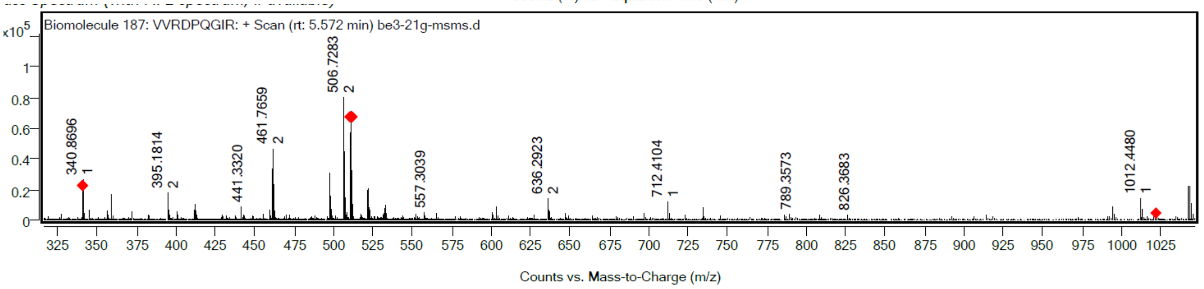

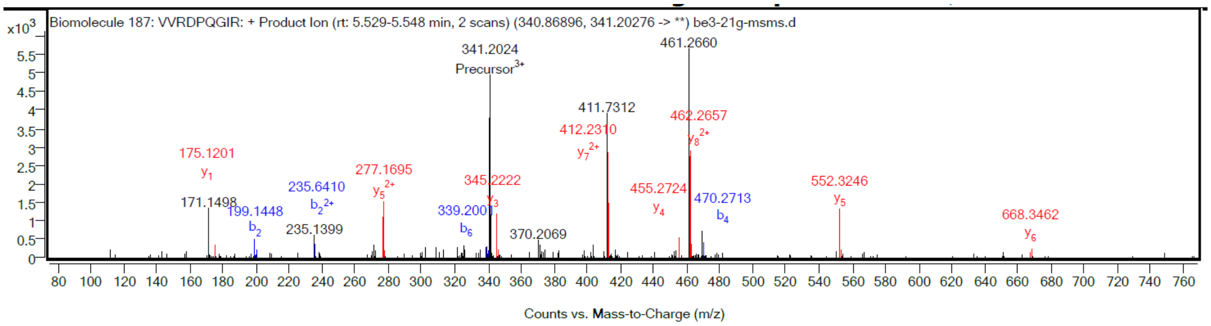

Fragment Spectrum Peaks

m/z	Diff (ppm)	Abund	Ion
175.1201	-6.71	332	y1
345.2222	6.70	1164	y3
455.2724	0.17	537	y4
552.3246	1.18	1302	y5
668.3462	14.05	205	y6
277.1695	-5.65	1507	y5
412.2310	-1.75	2835	y7
462.2657	1.00	2877	y8
199.1448	-3.49	485	b2
470.2713	1.77	385	b4
235.6410	-5.36	355	b4
339.2001	-29.50	287	b6
341.2024	-2.36	4965	Precursor
341.5373	-3.73	1149	Precursor

Identified peptide fragment **(1 site)**: QYVQGCGV (Sequence: AA 123-130, Q104)

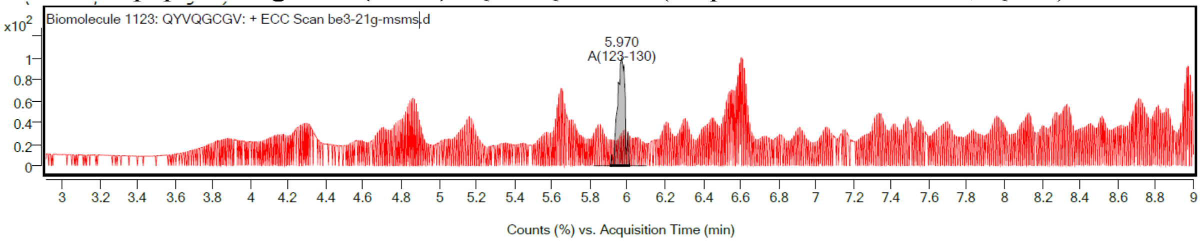

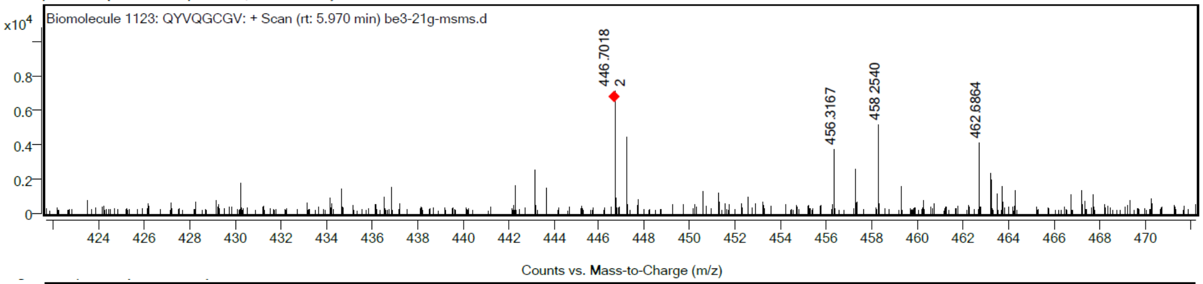

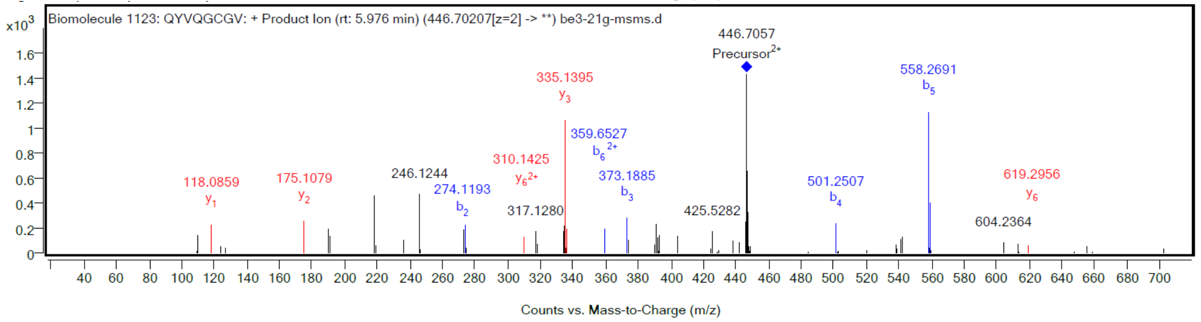

Fragment Spectrum Peaks

m/z	Diff (ppm)	Abund	Ion
118.0859	2.96	224	y1
175.1079	-0.98	254	y2
335.1395	-3.40	1062	y3
619.2956	-14.22	60	y6
310.1425	14.61	126	y6
274.1193	-2.55	222	b2
373.1885	-3.85	277	b3
501.2507	-10.08	234	b4
558.2691	-3.68	1125	b5
359.6527	-0.43	192	b6
446.7057	-6.58	1430	Precursor
447.2048	-0.85	654	Precursor

Nitrile Formation on Lysozyme Chicken

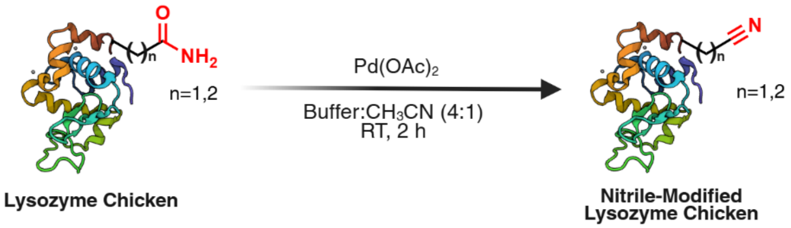

Reaction conducted according to **GP-XV**. Lysozyme Chicken (2 mg, 0.140 mM) was dissolved in 800 µL of NaP buffer (10 mM, pH 7.4) and 200 µL of $Pd(OAc)_2$ (2-10 mM) dissolved in ACN was added. The reaction was stirred at room temperature for 2 h followed by quenching with 500 µL of 1 M solution of aqueous L-cysteine and 10 µL of 1 M NaOH solution. The crude reaction mixture was passed through an Amicon™ Ultra 3 kDa centrifugal filter and washed with H_2O (7×0.5 mL) to remove the water-soluble Pd complex. The labeled protein was redissolved in 0.1% formic acid in H_2O and analyzed using LC-MS to determine conversion to nitrile (see table below). The sample treated with 5 mM $Pd(OAc)_2$ was additionally digested using SMART Digest™ Trypsin Kit by Thermo Scientific and analyzed by LC-MS/MS, showing 14 nitrile-modified sites.

Modification	Mass
Unmodified	14305.5
1 Nitrile	14287.4 (**-18**)
2 Nitrile	14269.3 (**-18**)
≥3 Nitrile	14251.2 (**-18**)…

Concentration of $Pd(OAc)_2$	Unmodified Protein	1 Nitrile	2 Nitrile	≥ 3 Nitrile	Overall Conversion
2 mM (0.45 mg)	58%	36%	6%	n.d.	42%
3 mM (0.67 mg)	47%	40%	11%	2%	53%
4 mM (0.90 mg)	24%	47%	24%	5%	76%
5 mM (1.12 mg)	12%	29%	33%	26%	88%

10 mM (2.24 mg)	5%	10%	18%	67%	95%

MS of Unmodified Lysozyme Chicken

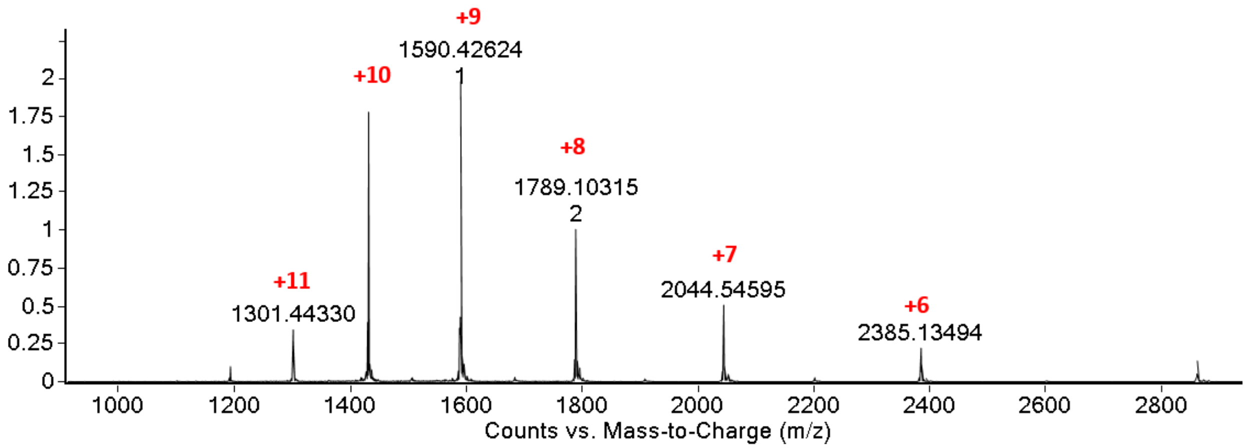

Deconvoluted MS of Unmodified Lysozyme Chicken

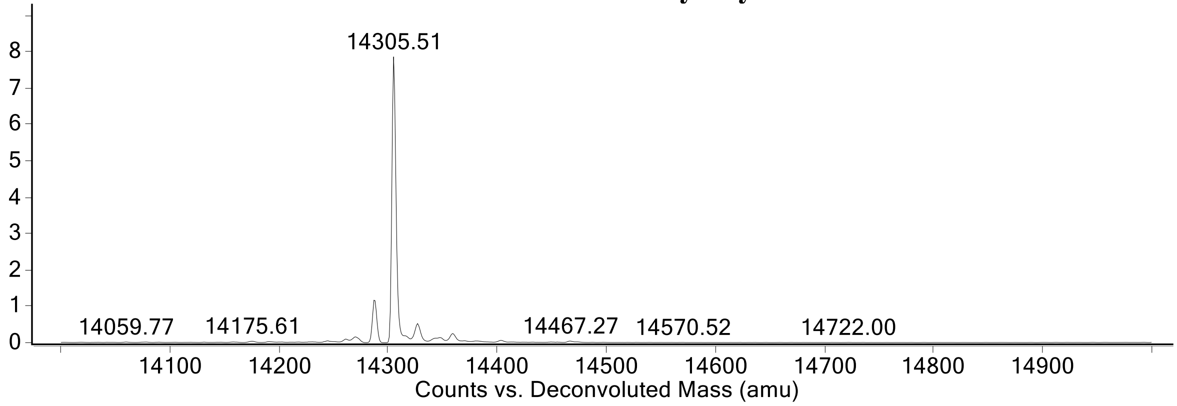

MS of Modified Lysozyme Chicken (5 mM $Pd(OAc)_2$)

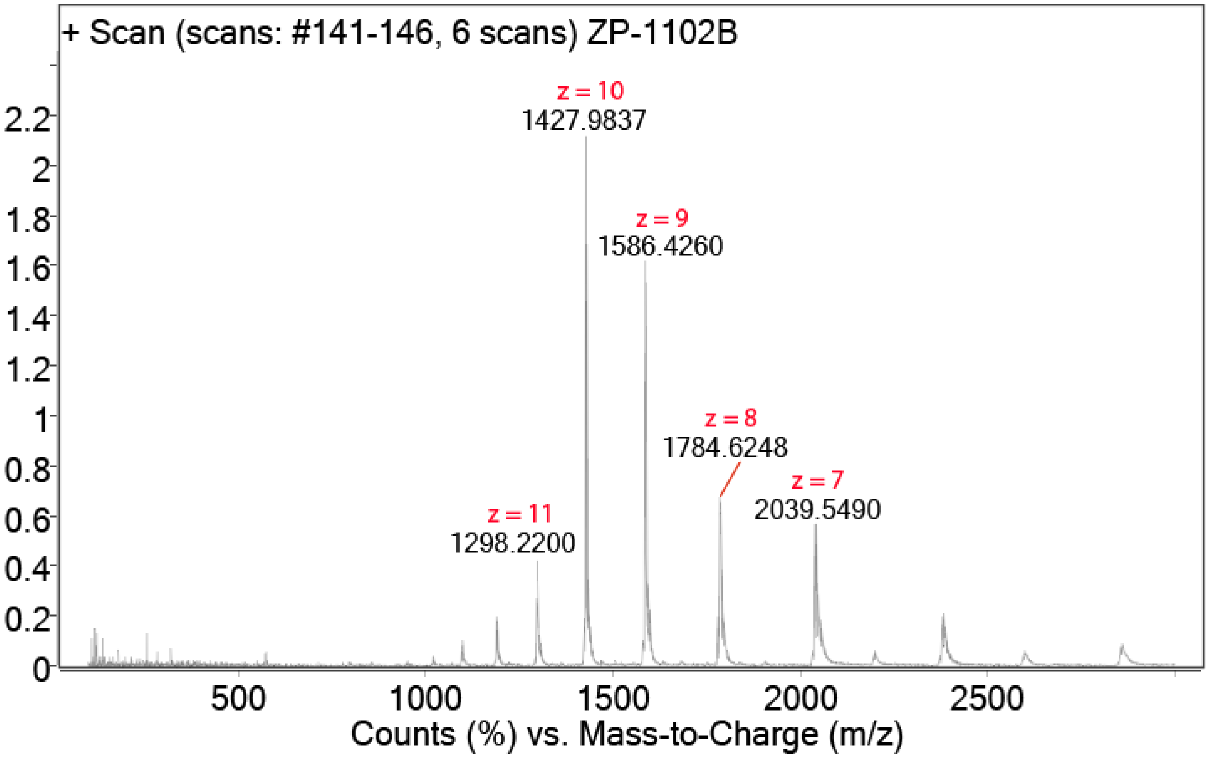

Deconvoluted MS of Modified Lysozyme Chicken (5 mM $Pd(OAc)_2$)

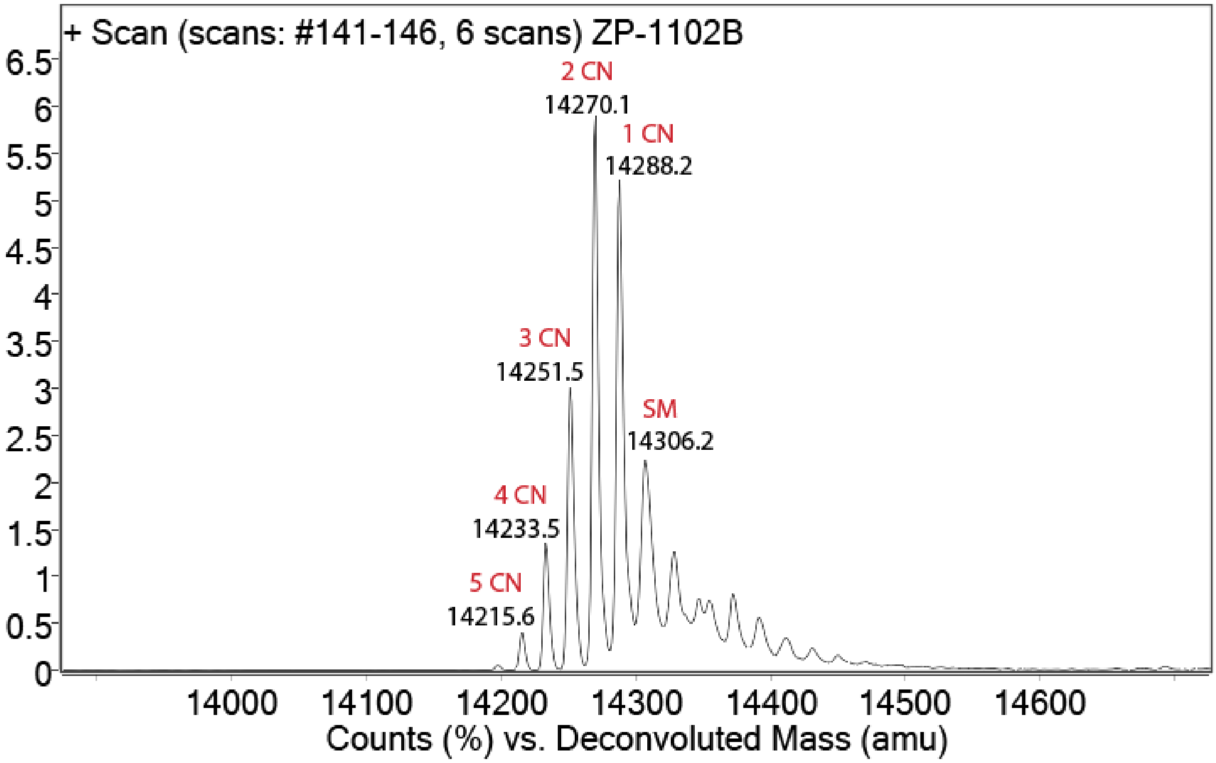

Deconvoluted MS of Nitrile-Modified Lysozyme Chicken (2 mM $Pd(OAc)_2$)

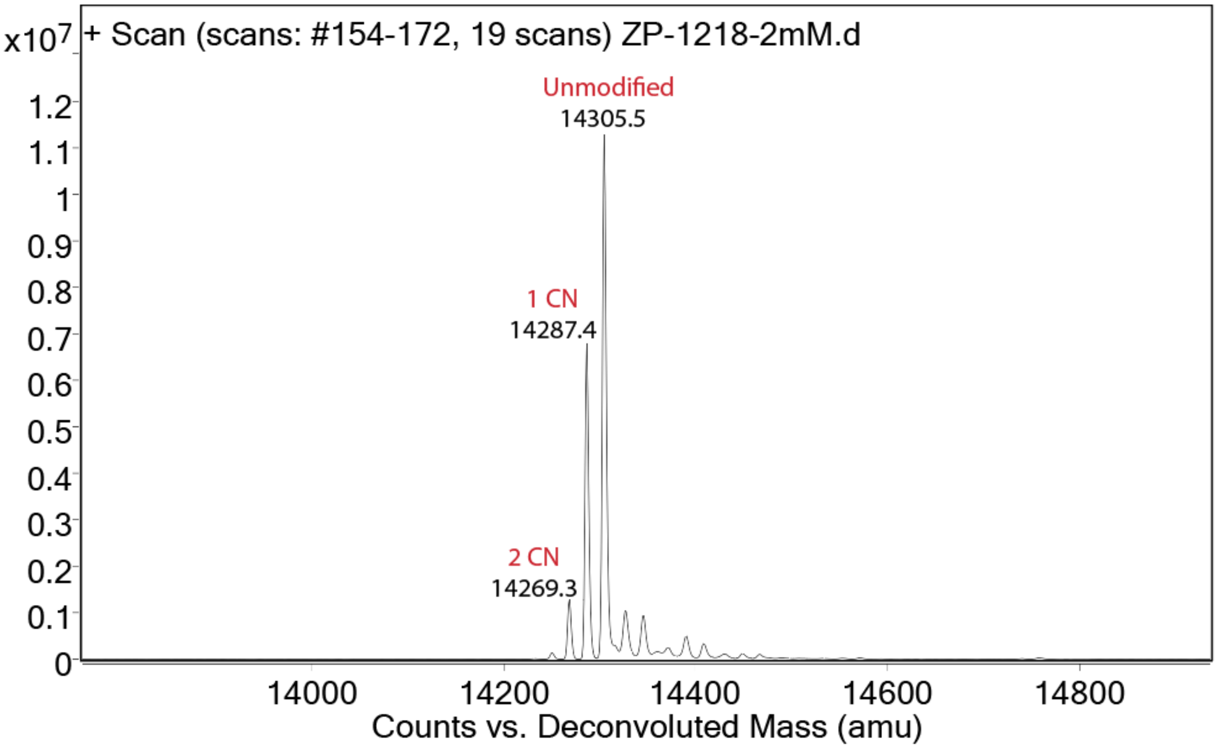

Deconvoluted MS of Nitrile-Modified Lysozyme Chicken (3 mM $Pd(OAc)_2$)

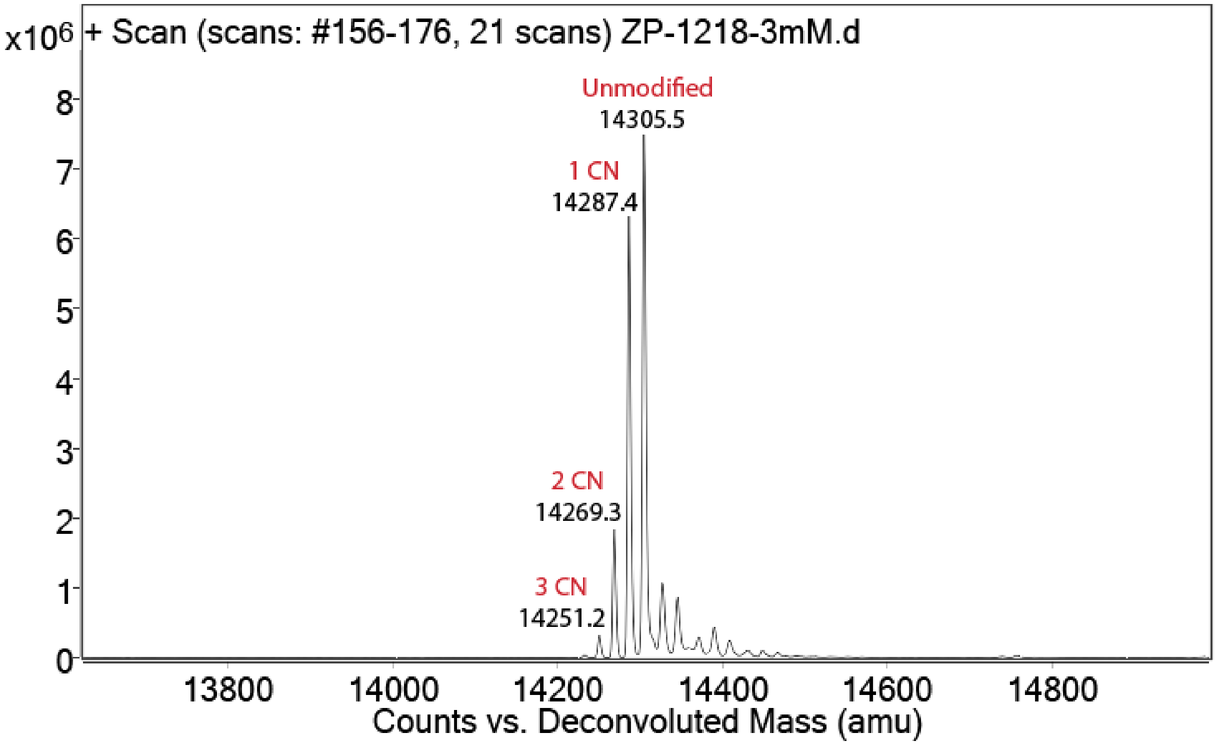

Deconvoluted MS of Nitrile-Modified Lysozyme Chicken (4 mM $Pd(OAc)_2$)

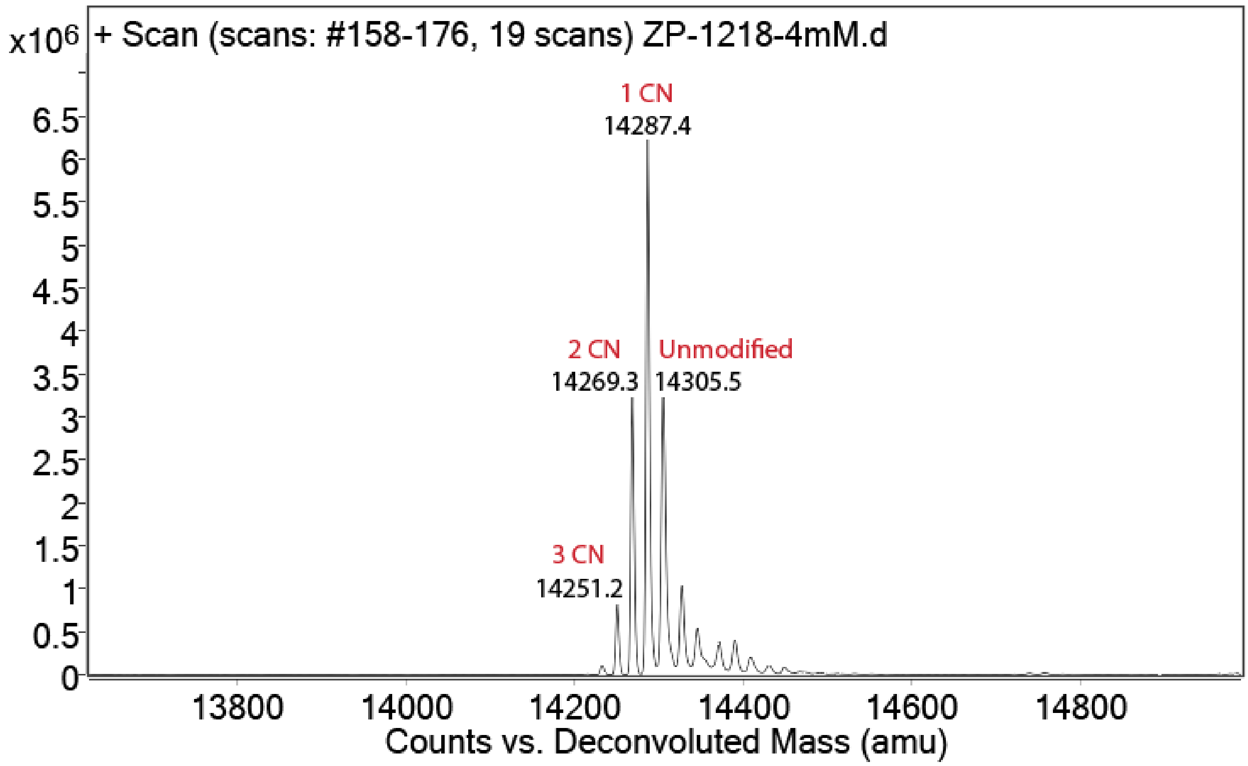

Deconvoluted MS of Nitrile-Modified Lysozyme Chicken (10 mM $Pd(OAc)_2$)

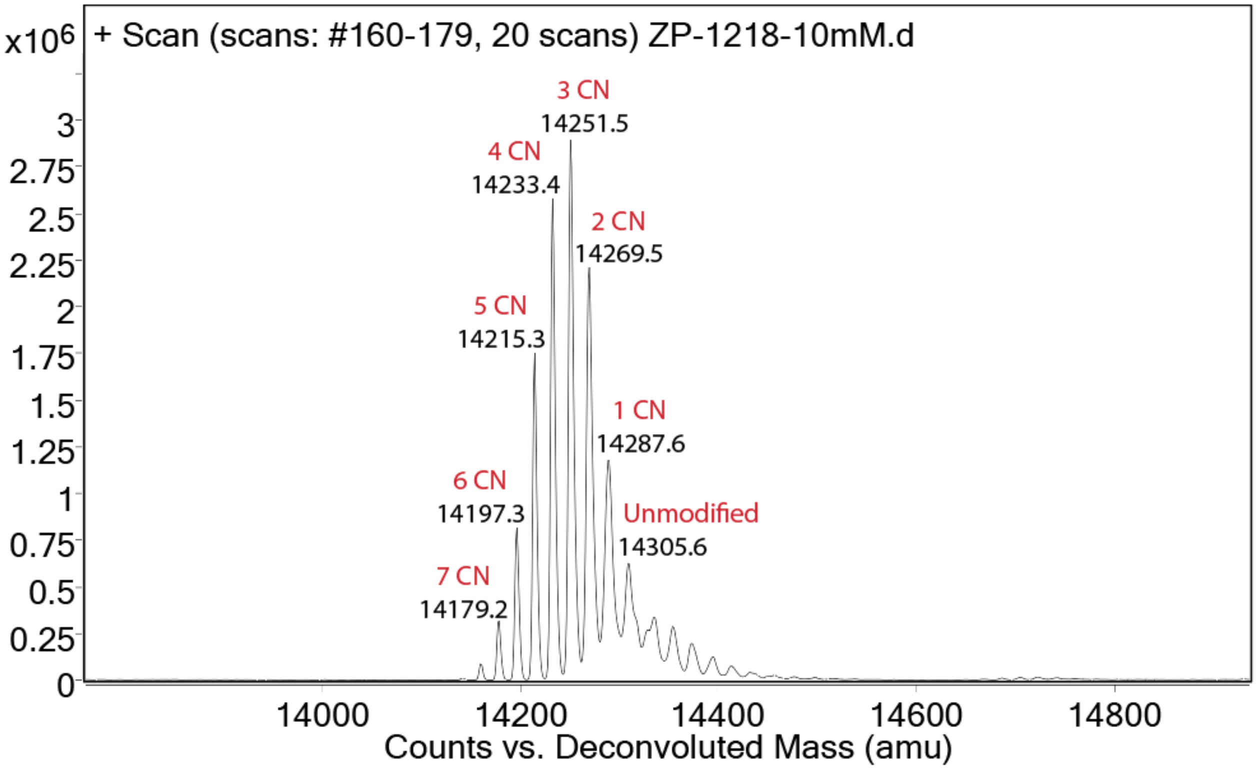

MS/MS Analysis of Digested Modified Lysozyme Chicken

Identified peptide fragment **(1 site)**: HGLDNYRGY (Sequence: AA 15-23, N19)

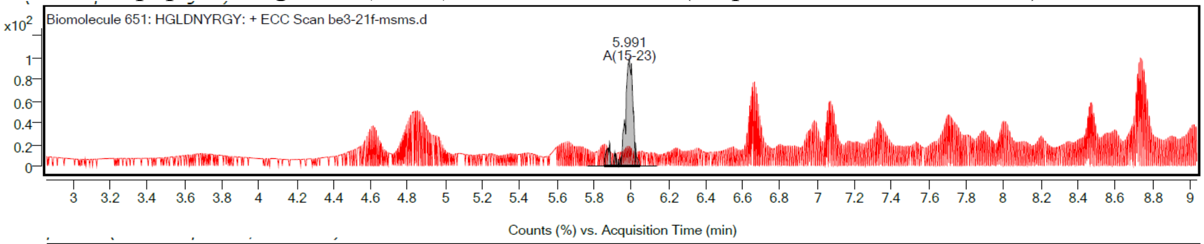

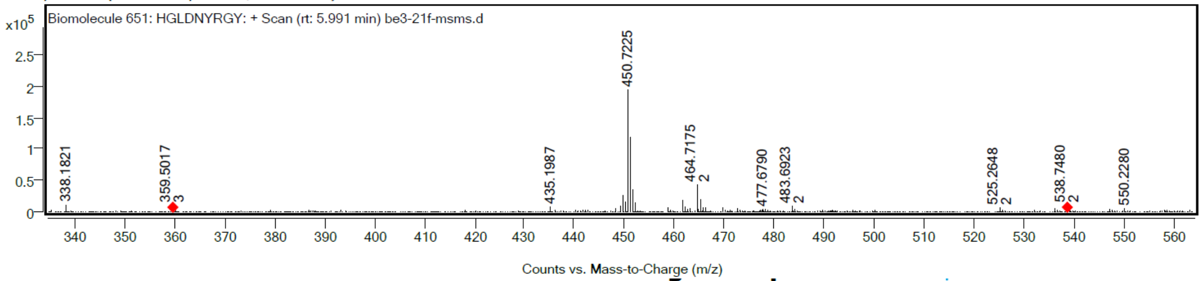

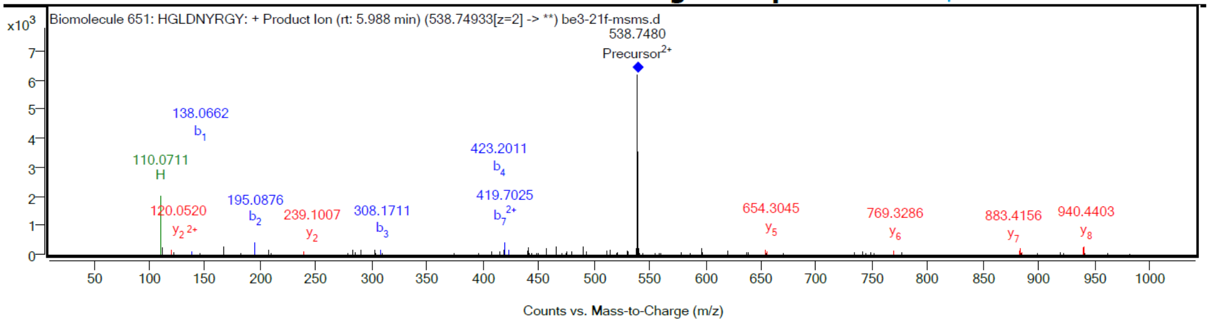

Fragment Spectrum Peaks

m/z	Diff (ppm)	Abund	Ion
239.1007	8.28	74	y2
654.3045	-7.78	134	y5
769.3286	-2.83	96	y6
883.4156	-2.06	194	y7
940.4403	-5.36	242	y8
120.0520	24.88	140	y2
138.0662	-0.27	67	b1
195.0876	0.19	375	b2
308.1711	1.93	123	b3
423.2011	-5.72	128	b4
419.7025	-2.72	395	b7
110.0711	1.42	1999	H
538.7480	1.94	6185	Precursor
539.2498	1.77	3507	Precursor
539.7565	-7.53	192	Precursor

Identified peptide fragment **(1 site)**: GYSLGN (Sequence: AA 22-27, N27)

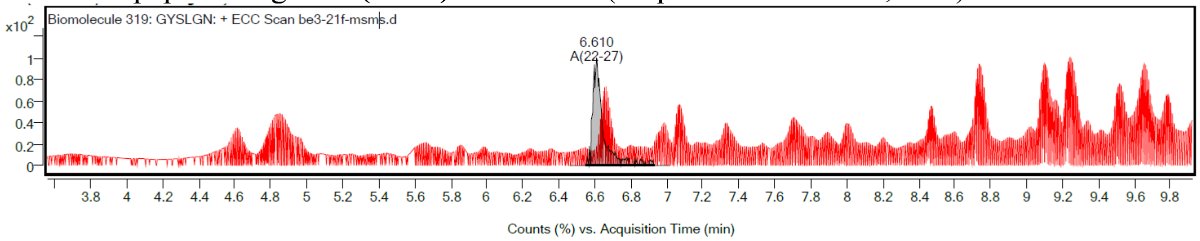

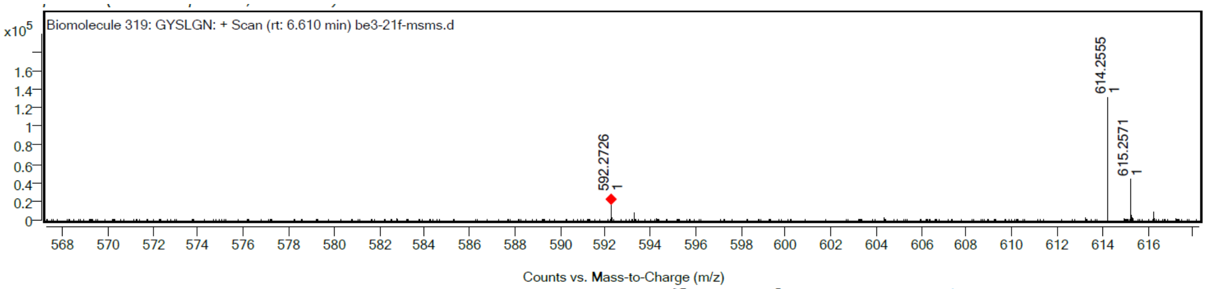

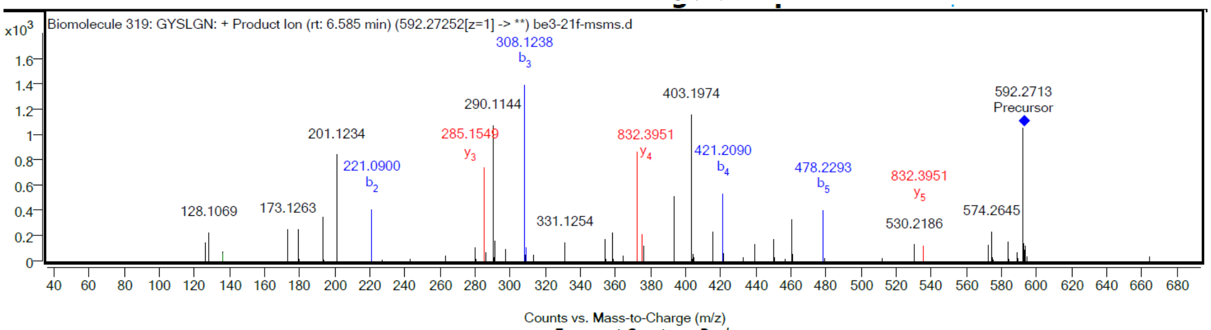

Fragment Spectrum Peaks

m/z	Diff (ppm)	Abund	Ion
285.1549	2.94	735	y3
372.1875	0.81	861	y4
535.2459	9.61	116	y5
221.0900	9.52	405	b2
308.1238	0.98	1390	b3
421.2090	-1.97	530	b4
478.2293	0.73	396	b5
136.0731	18.73	58	Y
592.2713	2.08	1048	Precursor
593.2768	-1.49	114	Precursor

Identified peptide fragment **(3 sites)**: FESNFNTQATNR (Sequence: AA 34-45, [N37, N39, Q41])

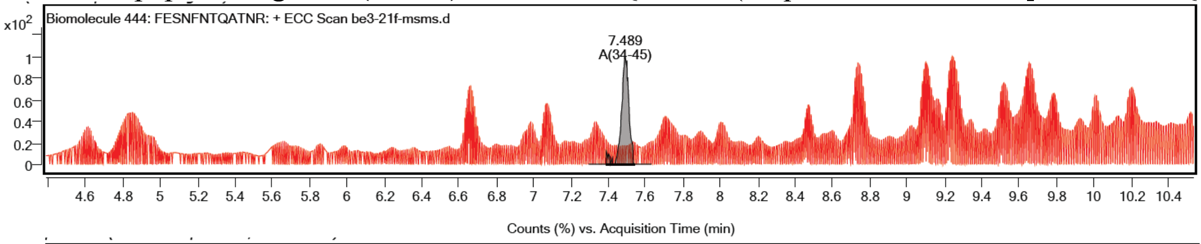

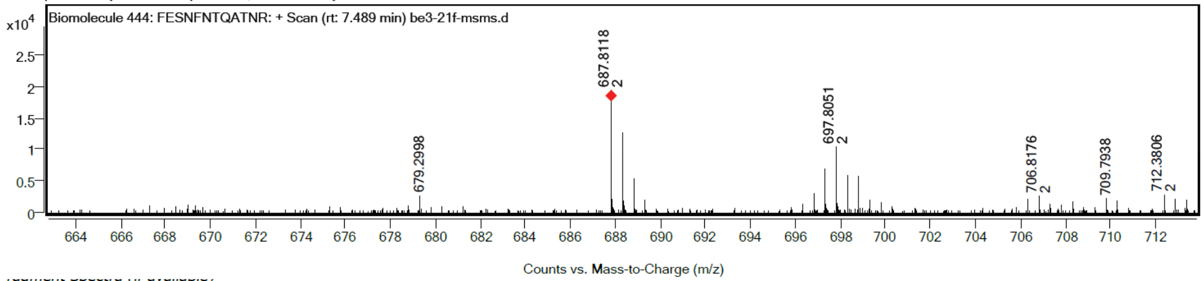

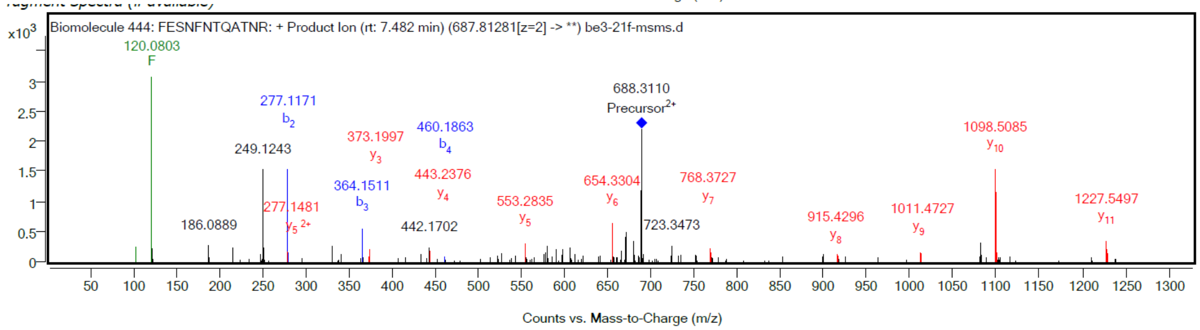

Fragment Spectrum Peaks

m/z	Diff (ppm)	Abund	Ion
373.1997	7.00	201	y3
443.2376	-3.41	171	y4
553.2835	1.14	296	y5
654.3304	2.17	628	y6
768.3727	2.62	218	y7
915.4296	14.75	114	y8
1011.4727	2.73	147	y9
1098.5085	-0.84	1531	y10
1227.5497	0.38	329	y11
277.1481	-8.65	159	y5
277.1171	4.42	1526	b2
364.1511	-2.12	543	b3
460.1863	-7.78	76	b4
102.0548	1.33	244	E
120.0803	3.80	3058	F
687.8134	-0.65	1174	Precursor
688.3110	5.16	2197	Precursor
688.8106	8.25	151	Precursor

Identified peptide fragment **(3 sites)**: NTDGSTDYGILQINSR (Sequence: AA 46-61, [N46, Q57, N59])

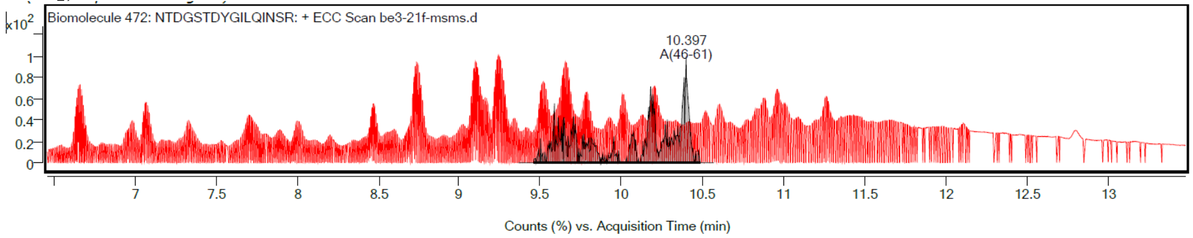

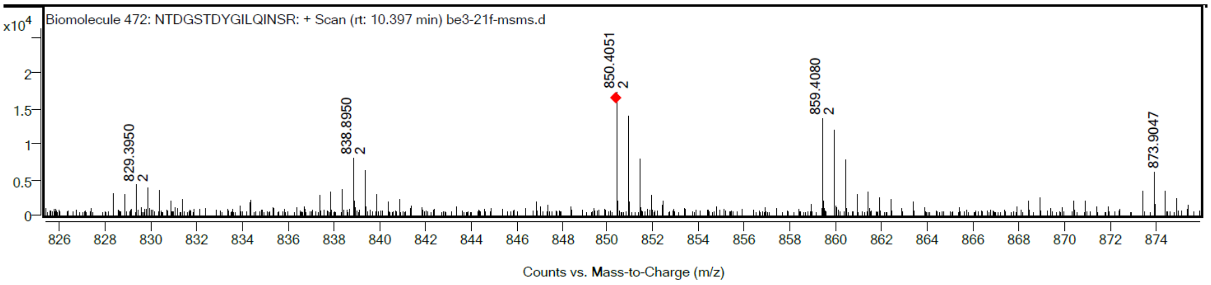

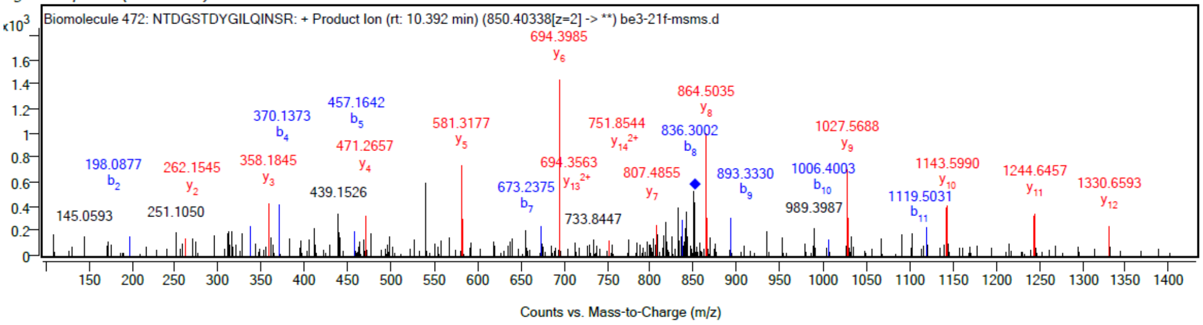

m/z	Diff (ppm)	Abund	Ion
262.1545	-13.41	132	y2
358.1845	-3.35	419	y3
471.2657	3.64	314	y4
581.3177	-3.84	727	y5
694.3985	1.38	1432	y6
807.4855	-2.37	242	y7
864.5035	1.72	989	y8
1027.5688	-0.44	698	y9
1143.5990	-0.35	398	y10
1244.6457	0.47	335	y11
1330.6593	11.81	235	y12
694.3563	-6.44	110	y13
751.8544	14.52	115	y14
198.0877	-1.92	149	b2
370.1373	-4.20	412	b4
457.1642	7.75	191	b5
673.2375	7.21	233	b7
836.3002	6.54	284	b8
893.3330	-6.48	301	b9
1006.4003	10.88	127	b10
1119.5031	-6.94	223	b11
337.1121	37.80	231	b7

Identified peptide fragment **(1 site)**: WWCNDGR (Sequence: AA 62-68, N65)

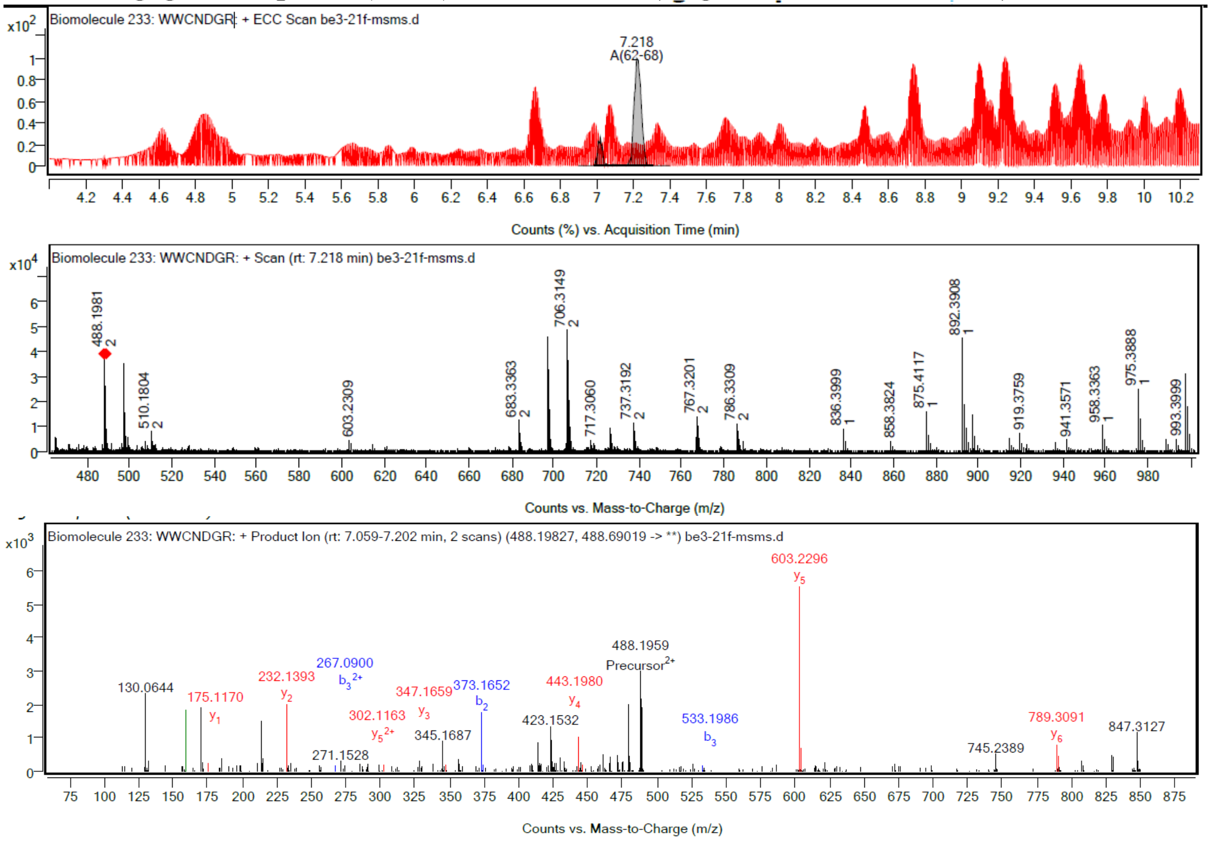

Fragment Spectrum Peaks

m/z	Diff (ppm)	Abund	Ion
175.1170	11.35	226	y1
232.1393	4.92	2002	y2
347.1659	4.19	178	y3
443.1980	3.87	1021	y4
603.2296	1.20	5535	y5
789.3091	0.73	782	y6
302.1163	8.34	191	y5
373.1652	1.80	1751	b2
533.1986	-3.82	171	b3
267.0900	44.49	172	b3
159.0911	3.74	1824	W
488.1959	4.54	3002	Precursor
488.6995	0.71	1057	Precursor

Identified peptide fragment **(2 sites)**: NLCNIPCSALLSSDITASVNCAKK (Sequence: AA 74-97, [N77, N93])

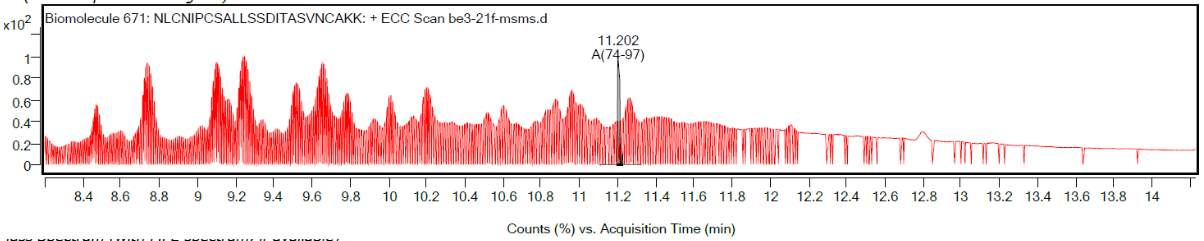

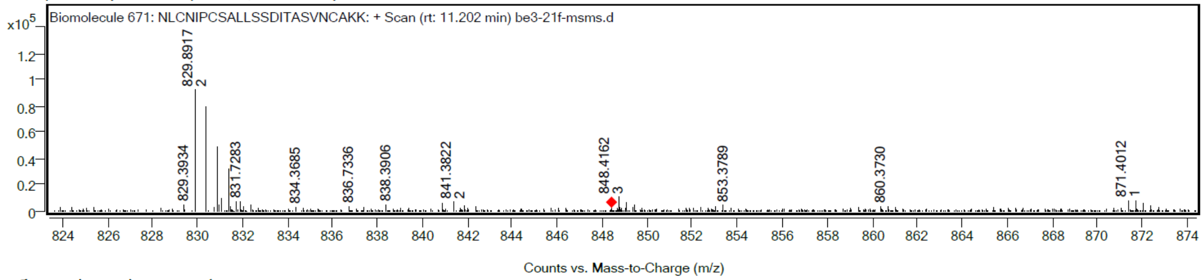

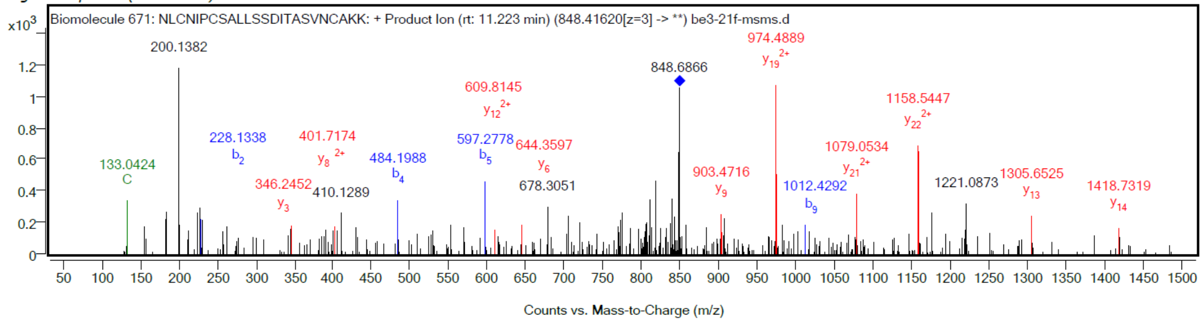

Fragment Spectrum Peaks

m/z	Diff (ppm)	Abund	Ion
346.2452	-0.95	174	y3
644.3597	-7.59	180	y6
903.4716	0.07	248	y9
1305.6525	-4.41	238	y13
1418.7319	-0.80	159	y14
401.7174	-4.30	169	y8
609.8145	-5.77	147	y12
974.4889	0.15	1067	y19
1079.0534	-5.69	376	y21
1158.5447	13.98	683	y22
228.1338	2.21	216	b2
484.1988	-3.04	337	b4
597.2778	6.00	453	b5
1012.4292	4.61	178	b9
133.0424	4.28	336	C
848.4227	-4.71	440	Precursor
848.7578	-5.53	385	Precursor
849.0825	5.98	596	Precursor
849.4236	-1.80	239	Precursor

Identified peptide fragment **(2 sites)**: IVSDGNGMNAWVAWR (Sequence: AA 98-112, [N103, N106])

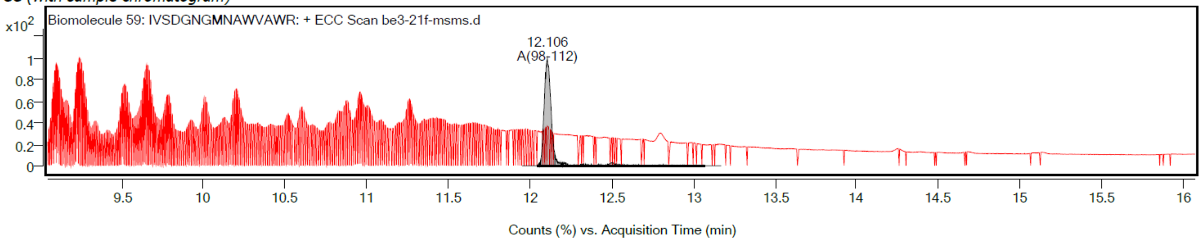

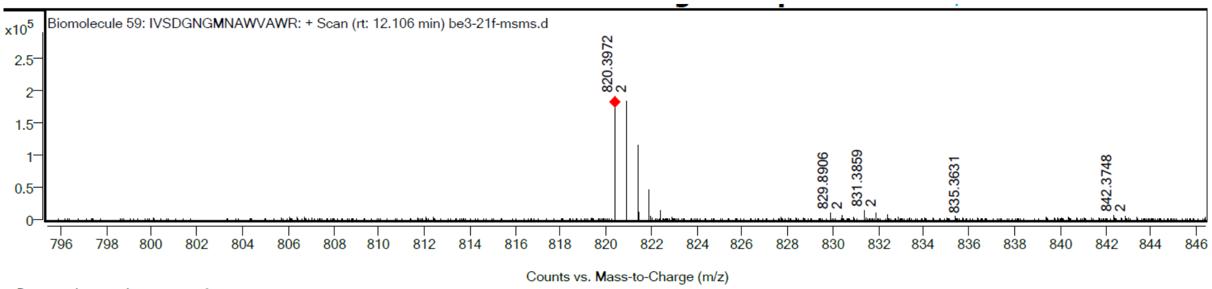

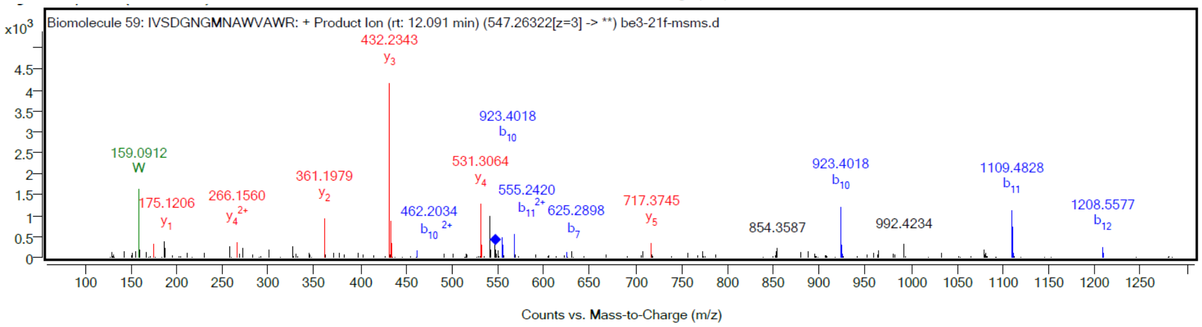

Fragment Spectrum Peaks

m/z	Diff (ppm)	Abund	Ion
175.1206	-9.42	317	y1
361.1979	1.10	924	y2
432.2343	2.46	4168	y3
531.3064	-4.99	1275	y4
717.3745	12.05	337	y5
266.1560	-1.61	356	y4
568.2753	-4.86	550	b6
625.2898	6.67	132	b7
923.4018	2.37	1213	b10
1109.4828	0.49	1122	b11
1208.5577	-4.95	239	b12
462.2034	4.82	169	b10
555.2420	5.91	477	b11
159.0912	3.25	1637	W

Identified peptide fragment **(1 site)**: TDVQAWIR (Sequence: AA 118-125, Q121)

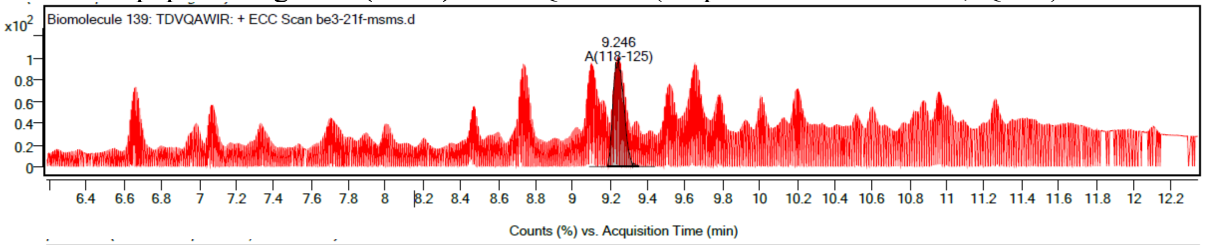

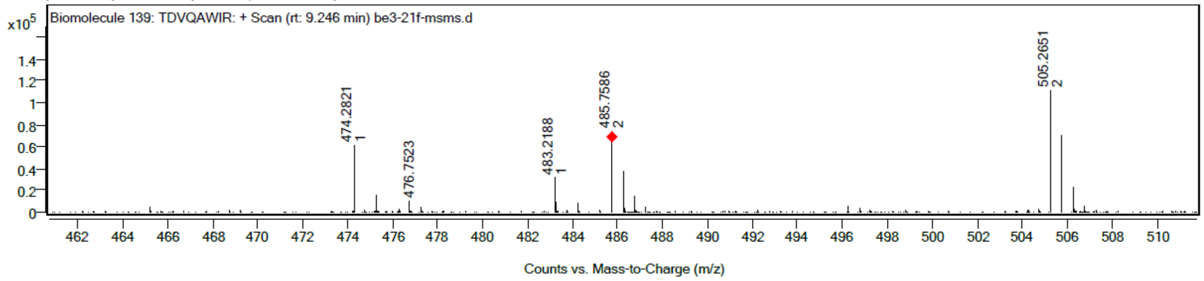

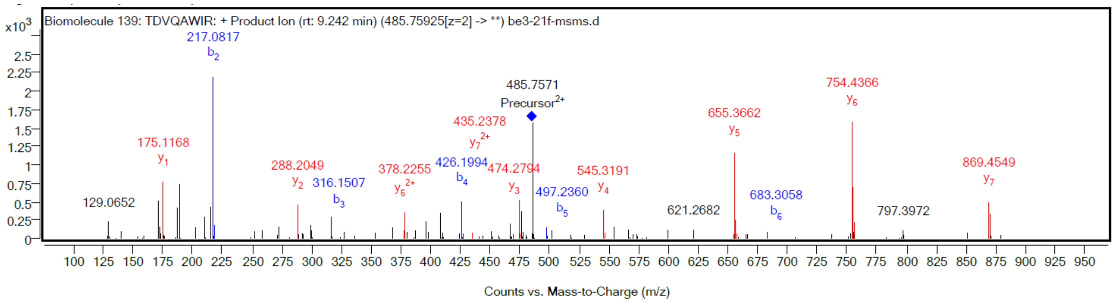

Fragment Spectrum Peaks

m/z	Diff (ppm)	Abund	Ion
175.1168	12.06	764	y1
288.2049	-6.57	458	y2
474.2794	6.08	520	y3
545.3191	0.67	386	y4
655.3662	1.85	1162	y5
754.4366	-0.92	1575	y6
869.4549	9.10	493	y7
378.2255	-5.95	351	y6
435.2378	-6.36	81	y7
217.0817	0.81	2190	b2
316.1507	-1.16	296	b3
426.1994	-2.50	494	b4
497.2360	-1.07	148	b5
683.3058	13.12	92	b6
485.7571	3.67	1572	Precursor
486.2563	8.73	1182	Precursor

Nitrile Formation on Chymotrypsinogen

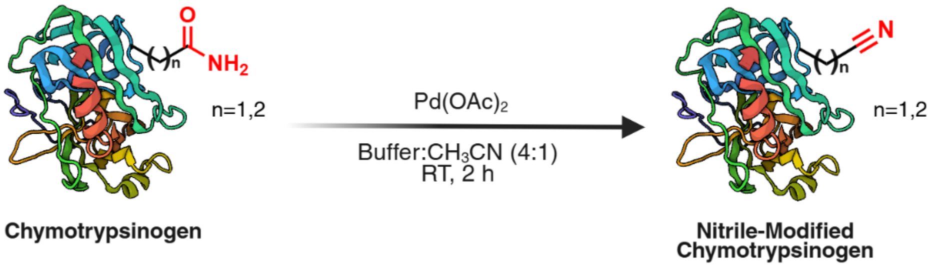

Reaction conducted according to **GP-XV**. Chymotrypsinogen (2 mg, 77.9 µM) was dissolved in 800 µL of NaP buffer (10 mM, pH 7.4) and 200 µL of $Pd(OAc)_2$ (1.12 mg, 5 mM) dissolved in ACN was added. The reaction was stirred at room temperature for 2 h followed by quenching with 500 µL of 1 M solution of aqueous L-cysteine and 10 µL of 1 M NaOH solution. The crude reaction mixture was passed through an Amicon™ Ultra 3 kDa centrifugal filter and washed with H_2O (7×0.5 mL) to remove the water-soluble Pd complex. The labeled protein was digested using SMART Digest™ Trypsin Kit by Thermo Scientific, and analyzed using LC-MS/MS. The total number of nitrile-containing sites was observed to be 15 with >95% overall conversion to nitrile.

MS of Unmodified Chymotrypsinogen

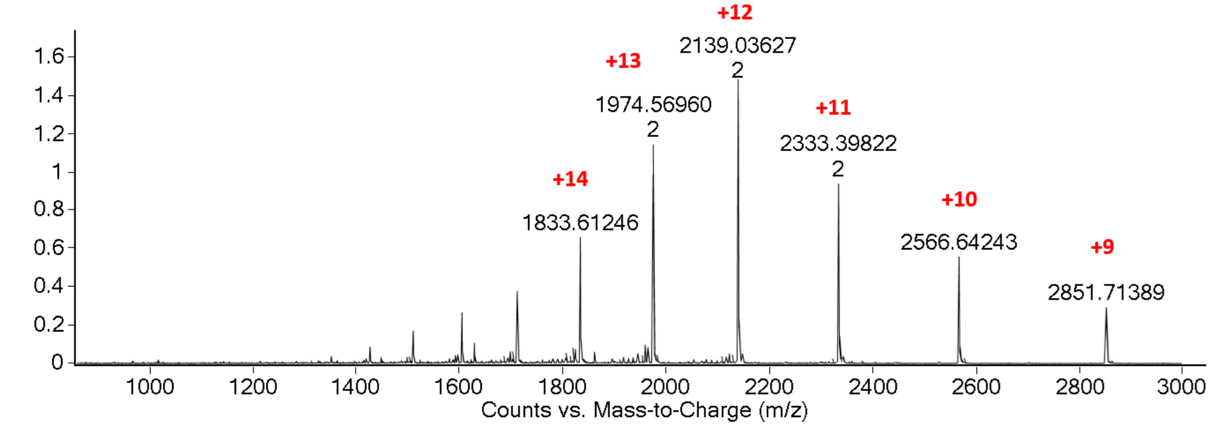

Deconvoluted MS spectra of Unmodified Chymotrypsinogen

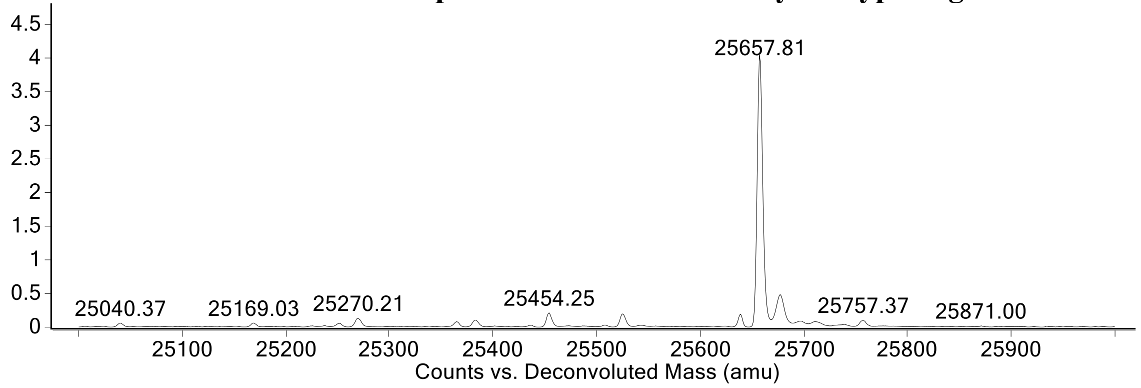

MS/MS Analysis of Digested Modified Chymotrypsinogen

Identified peptide fragment **(4 sites)**: CGVPAIQPVLSGLSRIVNGEEAVPGSWPWQVSLQDK (Sequence: AA 1-36, [Q7, N18, Q30, Q34])

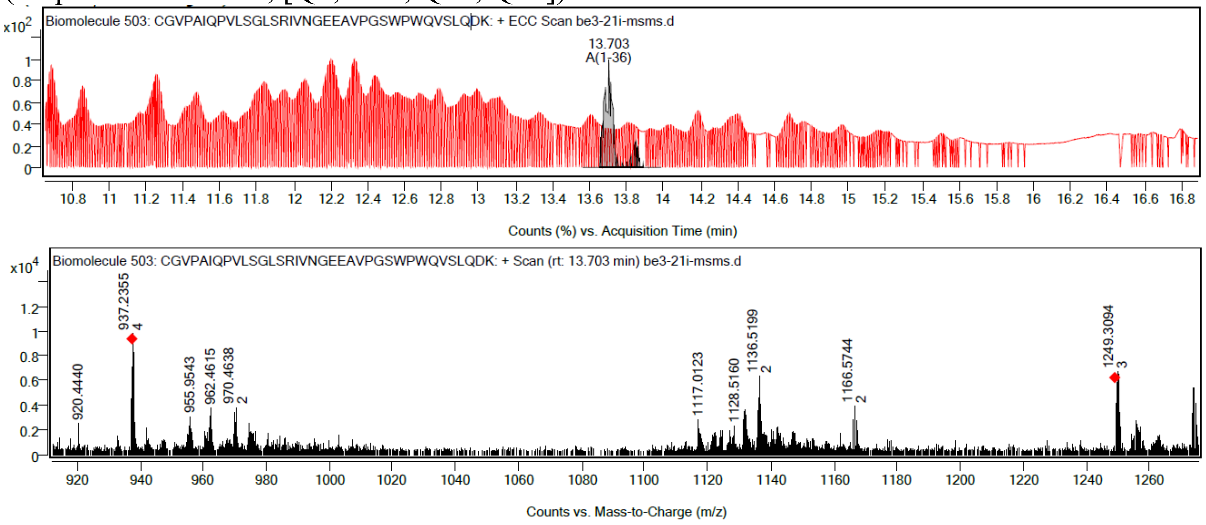

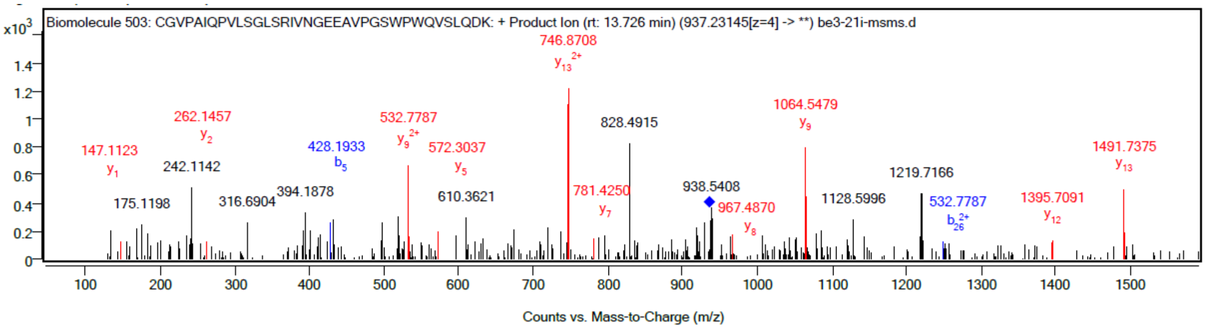

Fragment Spectrum Peaks

m/z	Diff (ppm)	Abund	Ion
147.1123	3.28	121	y1
262.1457	-22.75	123	y2
572.3037	0.21	196	y5
781.4250	-6.03	143	y7
967.4870	13.04	174	y8
1064.5479	4.20	793	y9
1395.7091	-14.71	129	y12
1491.7375	0.30	498	y13
532.7787	2.11	668	y9
746.8708	4.63	1218	y13
428.1933	6.88	262	b5
1248.6444	11.73	126	b26

Identified peptide fragment **(1 site)**: TGFHFCGGSLIN (Sequence: AA 37-48, N48)

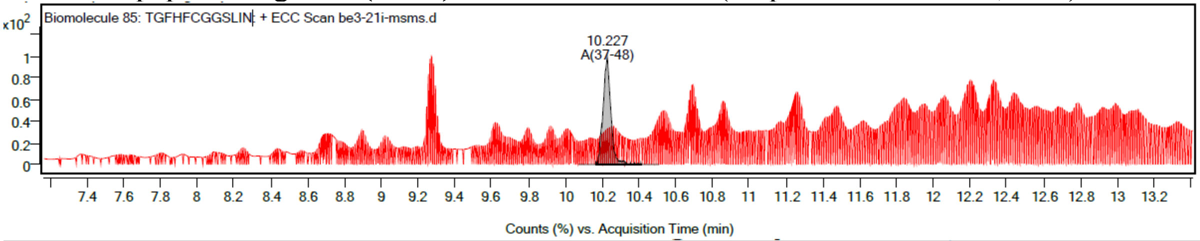

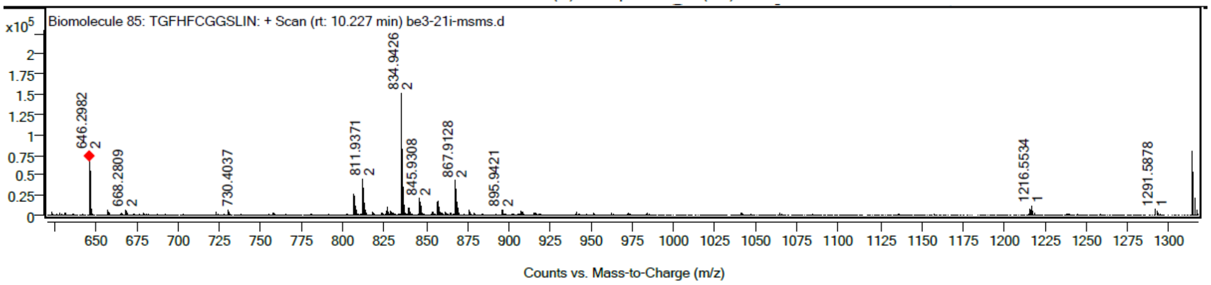

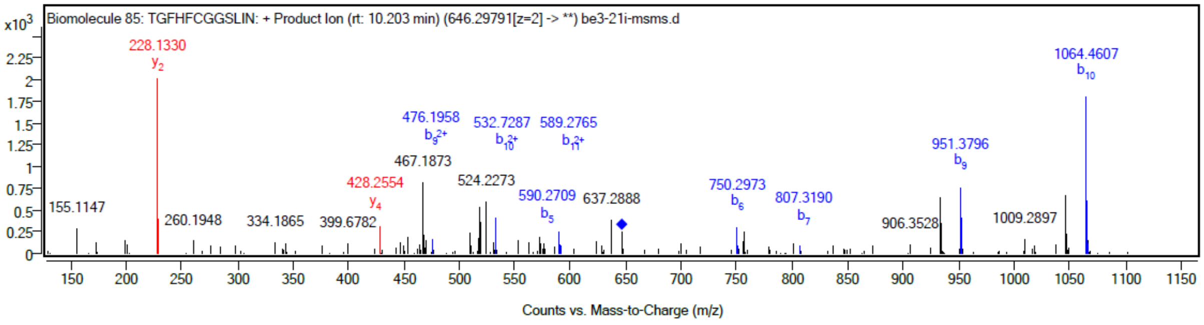

Fragment Spectrum Peaks			
m/z	Diff (ppm)	Abund	Ion
590.2709	15.27	102	b5
228.1330	5.47	2009	y2
428.2554	-11.85	313	y4
750.2973	7.31	300	b6
807.3190	6.57	90	b7
951.3796	-1.95	763	b9
1064.4607	1.07	1799	b10
476.1958	-6.92	161	b9
532.7287	11.01	410	b10
589.2765	0.14	253	b11
646.2989	-1.25	250	Precursor
646.8042	-6.93	182	Precursor

Identified peptide fragment **(1 site)**: TGFHFCGGSLINENWVVTAAH (Sequence: AA 37-57, N48)

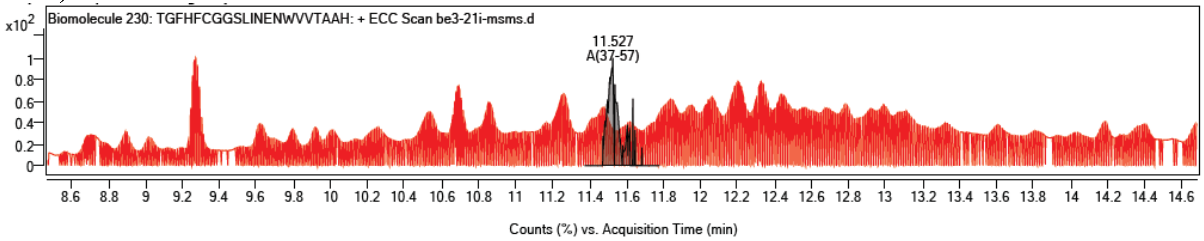

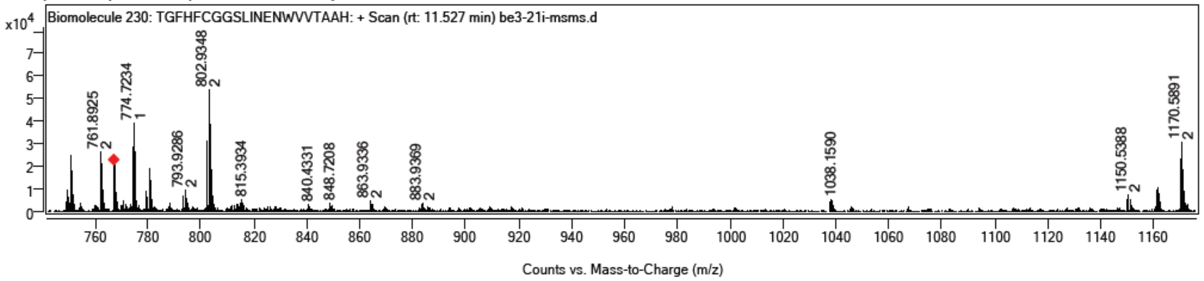

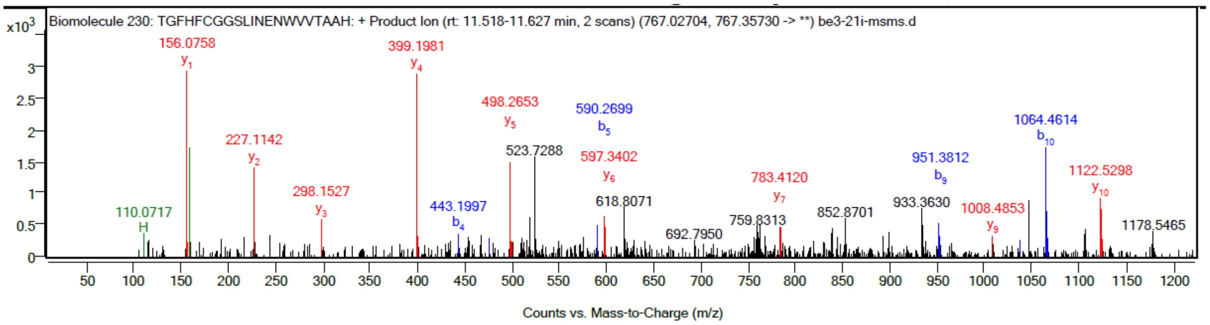

Fragment Spectrum Peaks			
m/z	Diff (ppm)	Abund	Ion
156.0758	6.07	2934	y1
227.1142	-1.42	1394	y2
298.1527	-5.69	573	y3
399.1981	1.47	2888	y4
498.2653	3.56	1482	y5
597.3402	-7.83	630	y6
783.4120	3.59	458	y7
1008.4853	4.39	316	y9
1122.5298	2.54	909	y10
443.1997	9.21	347	b4
590.2699	3.79	477	b5
951.3812	-3.58	512	b9
1064.4614	0.44	1718	b10
476.1917	1.81	280	b9
1037.4694	17.56	251	b19
110.0717	-4.15	362	H
159.0907	5.85	1721	W
767.3521	14.26	302	Precursor

Identified peptide fragment **(1 site)**: CGVTTSDVVVAGEFDQGSSSEK (Sequence: AA 58-79, Q73)

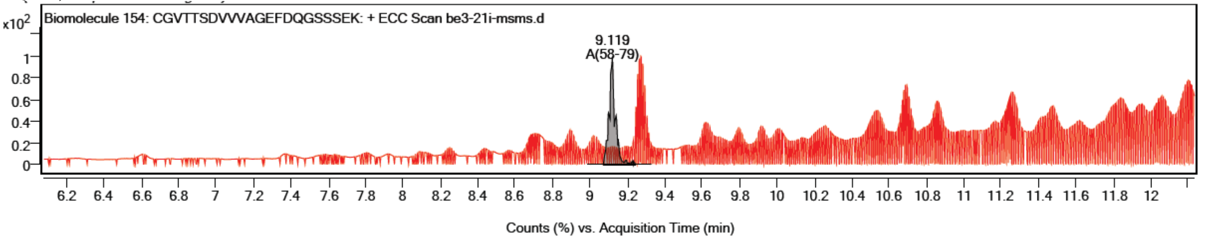

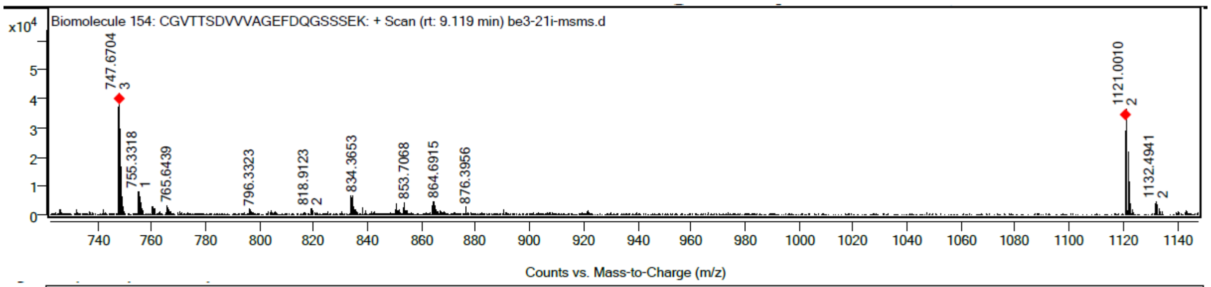

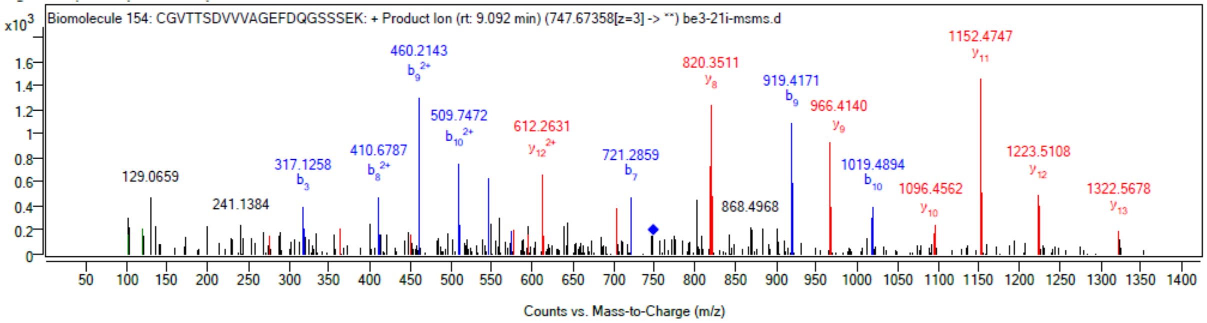

Fragment Spectrum Peaks

m/z	Diff (ppm)	Abund	Ion
276.1563	-3.38	153	y2
363.1848	7.36	209	y3
450.2197	-0.60	165	y4
594.2720	1.57	159	y6
704.3181	4.08	380	y7
820.3511	0.16	1234	y8
966.4140	2.44	924	y9
1096.4562	5.56	247	y10
1152.4747	4.88	1453	y11
1223.5108	5.48	491	y12
1322.5678	13.72	190	y13
576.7376	10.87	197	y11
612.2631	-1.13	664	y12
317.1258	6.19	389	b3
721.2859	-5.21	467	b7
919.4171	2.07	1085	b9
1019.4894	1.28	389	b10
410.6787	0.56	469	b8
460.2143	-2.50	1296	b9
509.7472	0.24	752	b10
545.2630	5.37	629	b11
573.7767	-0.13	196	b12
102.0558	-8.21	165	E
120.0804	3.43	209	F

Identified peptide fragment **(2 sites)**: YNSLTINNDITLLK (Sequence: AA 94-107, [N100, N101])

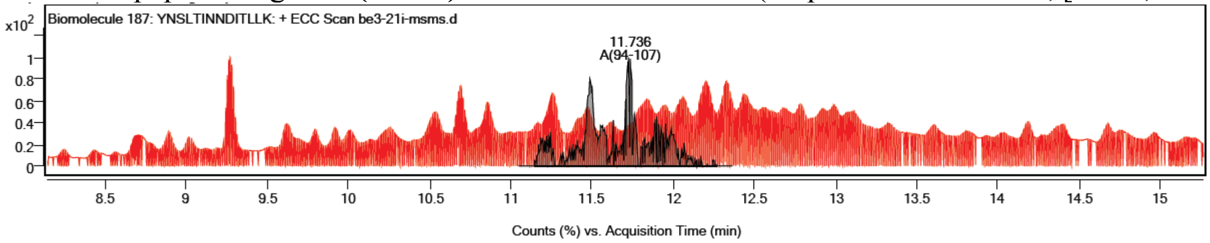

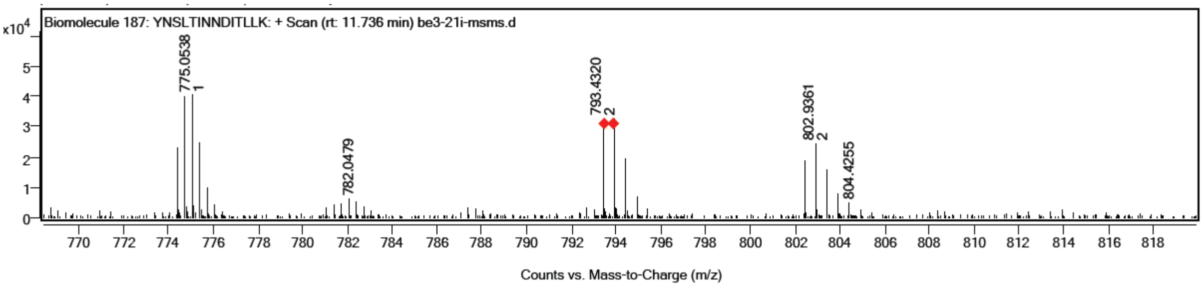

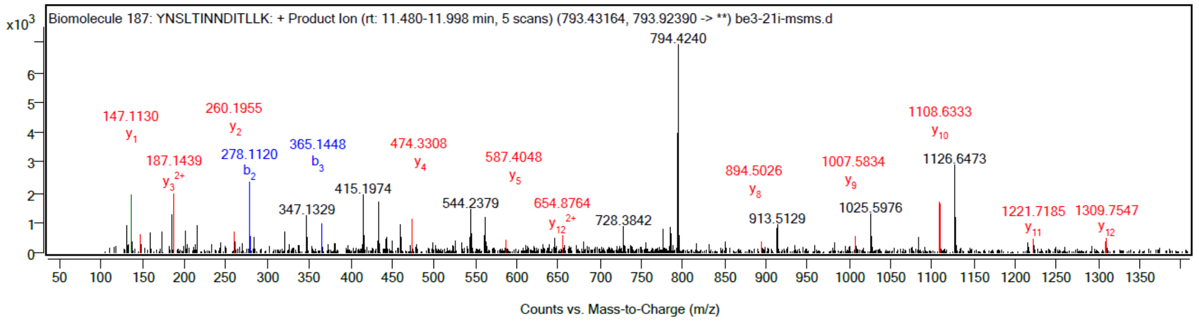

Fragment Spectrum Peaks

m/z	Diff (ppm)	Abund	Ion
147.1130	-1.17	583	y1
260.1955	5.36	673	y2
474.3308	-4.58	1098	y4
587.4048	13.36	413	y5
894.5026	1.90	357	y8
1007.5834	4.93	529	y9
1108.6333	2.49	1684	y10
1221.7185	1.34	454	y11
1309.7547	0.64	476	y12
187.1439	1.09	1968	y3
654.8764	5.02	567	y12
278.1120	5.40	2345	b2
365.1448	2.19	955	b3
136.0755	1.47	1924	Y
793.4284	5.61	3967	Precursor
793.9231	14.39	5295	Precursor

Identified peptide fragment **(1 site)**: SQTVSAVCLPSASDDFAAGTTCVTTGWGLTR (Sequence: AA 115-145, Q116)

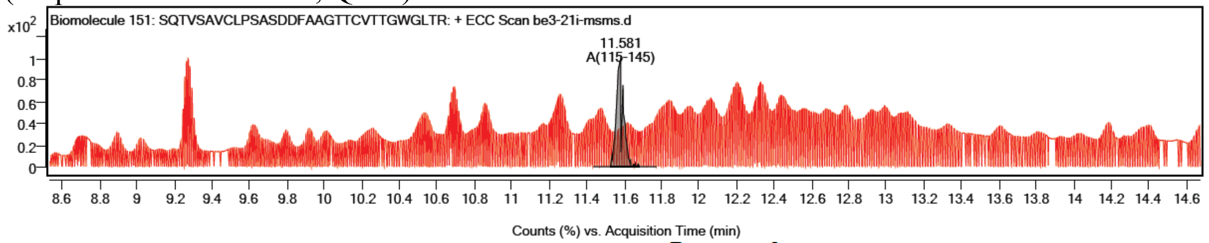

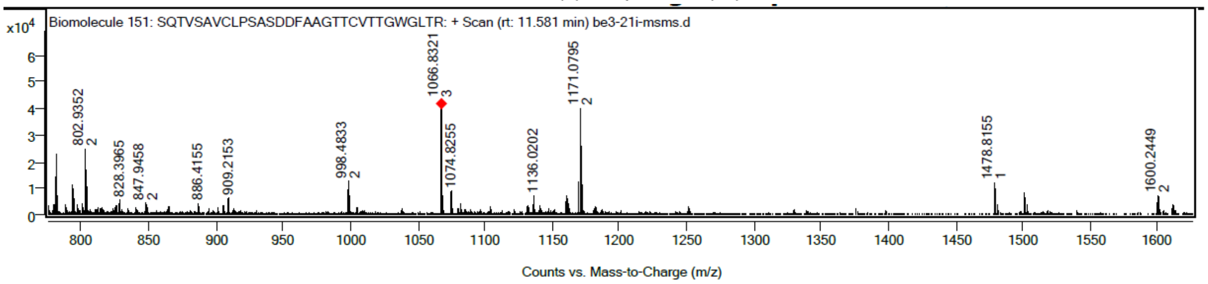

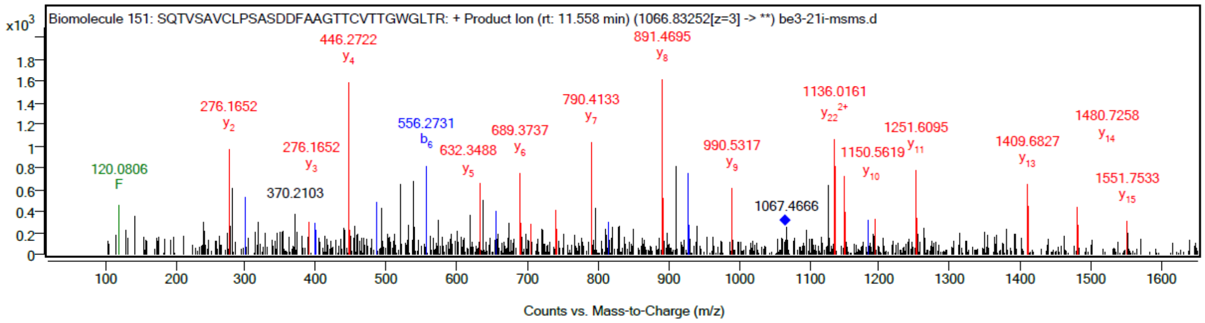

Fragment Spectrum Peaks

m/z	Diff (ppm)	Abund	Ion
276.1652	5.27	973	y2
389.2517	-2.69	293	y3
446.2722	-0.05	1591	y4
632.3488	4.28	659	y5
689.3737	-1.06	747	y6
790.4133	9.26	1037	y7
891.4695	-1.34	1616	y8
990.5317	5.10	609	y9
1150.5619	4.71	724	y10
1251.6095	4.45	773	y11
1409.6827	1.03	643	y13
1480.7258	-3.03	430	y14
1551.7533	3.28	308	y15
705.3443	2.03	275	y13
740.8650	-0.91	410	y14
1136.0161	4.30	1064	y22
1193.0639	0.63	320	y23
299.1333	5.52	529	b3
398.2045	-2.70	287	b4
485.2341	2.80	477	b5
556.2731	-0.99	816	b6
655.3432	-3.34	401	b7
815.3748	-3.87	297	b8
928.4491	7.08	745	b9
1183.5424	29.71	318	b12
120.0806	1.31	449	F

Identified peptide fragment **(1 site)**: LQQASLPLLSNTNCK (Sequence: AA 155-169, Q157)

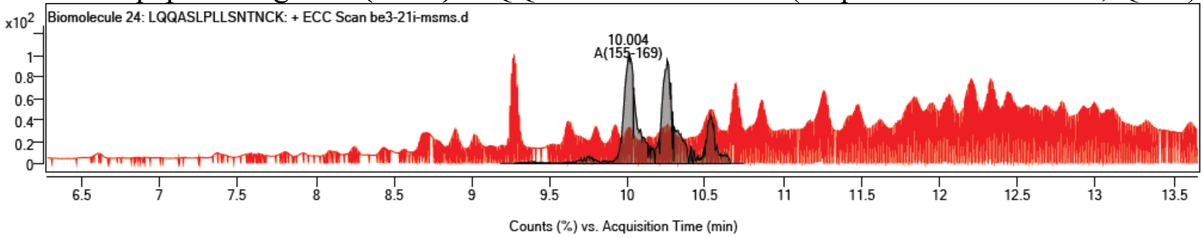

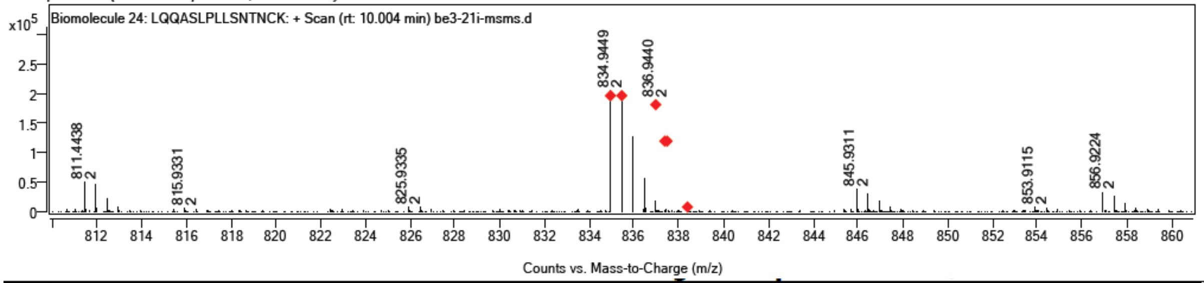

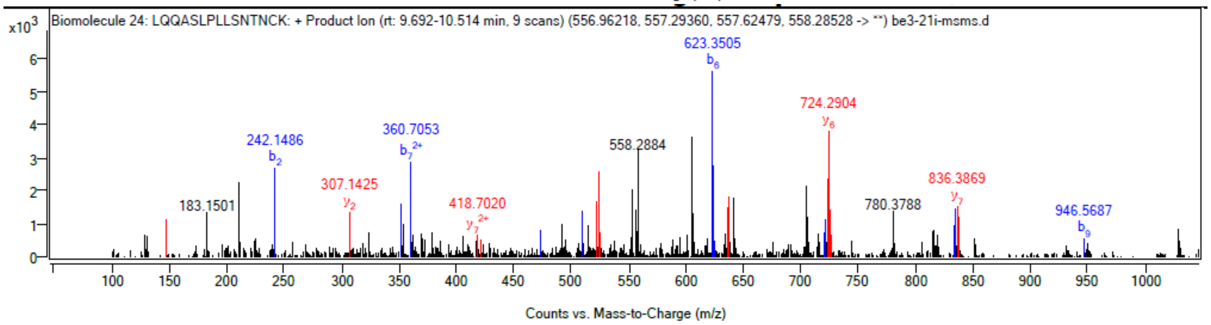

Fragment Spectrum Peaks

m/z	Diff (ppm)	Abund	Ion
147.1121	5.08	1099	y1
307.1425	3.07	1336	y2
421.1840	5.55	510	y3
524.2600	-36.77	2567	y4
637.2604	31.34	1798	y5
724.2904	30.32	3788	y6
836.3869	7.38	1499	y7
418.7020	-4.43	632	y7
242.1486	5.39	2671	b2
352.1981	-0.51	1570	b3
510.2674	-0.72	1382	b5
623.3505	1.01	5592	b6
721.4056	2.27	1124	b7
834.4885	3.38	1435	b8
946.5687	3.52	520	b9
360.7053	0.83	2842	b7
473.7832	13.71	776	b9

Identified peptide fragment **(1 site)**: NGAWTLVGIVSWGSSTCSTSTPGVYAR (Sequence: AA 204-230, N204)

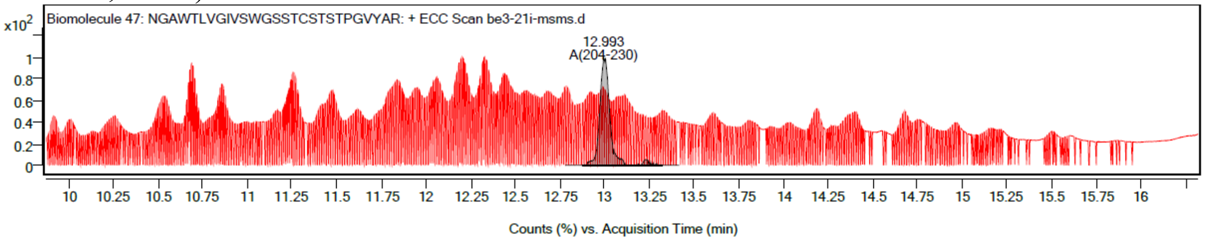

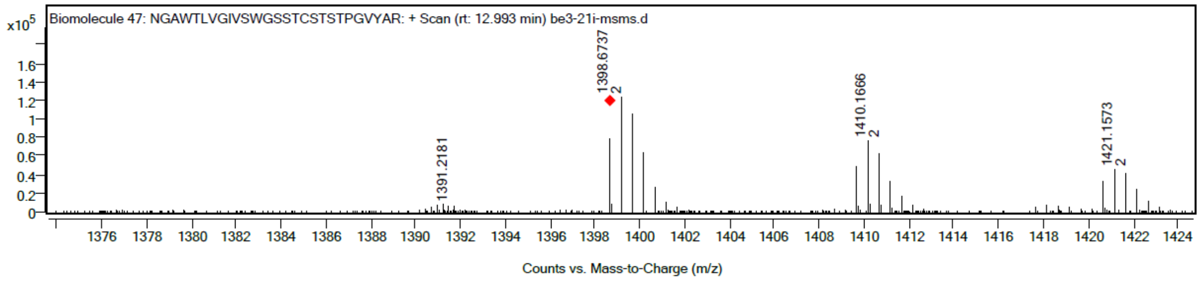

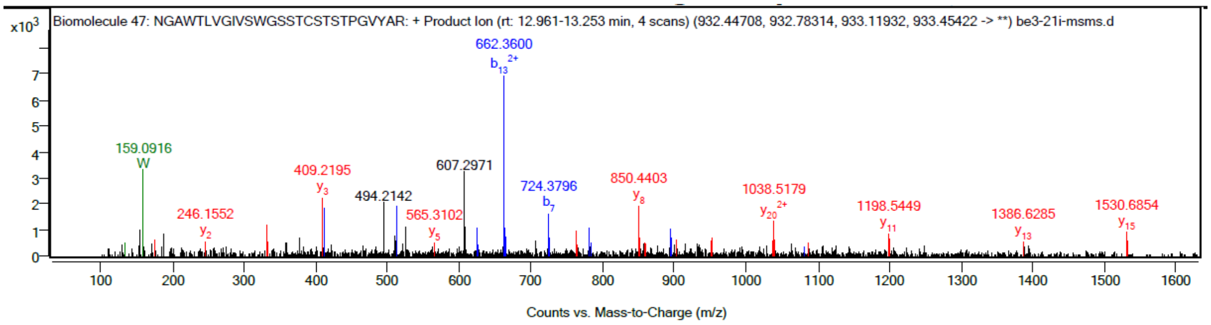

Fragment Spectrum Peaks

m/z	Diff (ppm)	Abund	Ion
175.1180	5.37	579	y1
246.1552	3.38	539	y2
409.2195	-0.21	2226	y3
565.3102	-1.71	478	y5
763.4074	3.08	949	y7
850.4403	1.68	1906	y8
952.4870	6.11	616	y9
1198.5449	5.99	837	y11
1386.6285	2.37	529	y13
1530.6854	-0.05	887	y15
331.6827	5.89	1189	y6
858.8843	1.96	486	y16
902.4000	2.20	597	y17
952.4251	13.41	675	y18
1038.5179	-23.06	1310	y20
1086.5222	0.88	481	y21
411.1783	-1.80	1841	b4
512.2246	1.25	1887	b5
625.3101	-1.26	1067	b6
724.3796	-2.63	1595	b7
781.3985	0.81	1054	b8
894.4857	-2.76	1018	b9
1080.5851	-1.36	338	b11
662.3600	-21.39	6928	b13
133.0438	-6.20	465	C
159.0916	0.66	3318	W

Identified peptide fragment **(4 sites)**: VTALVNWVQQTLAAN (Sequence: AA 231-245, [N236, Q239, Q240, N245])

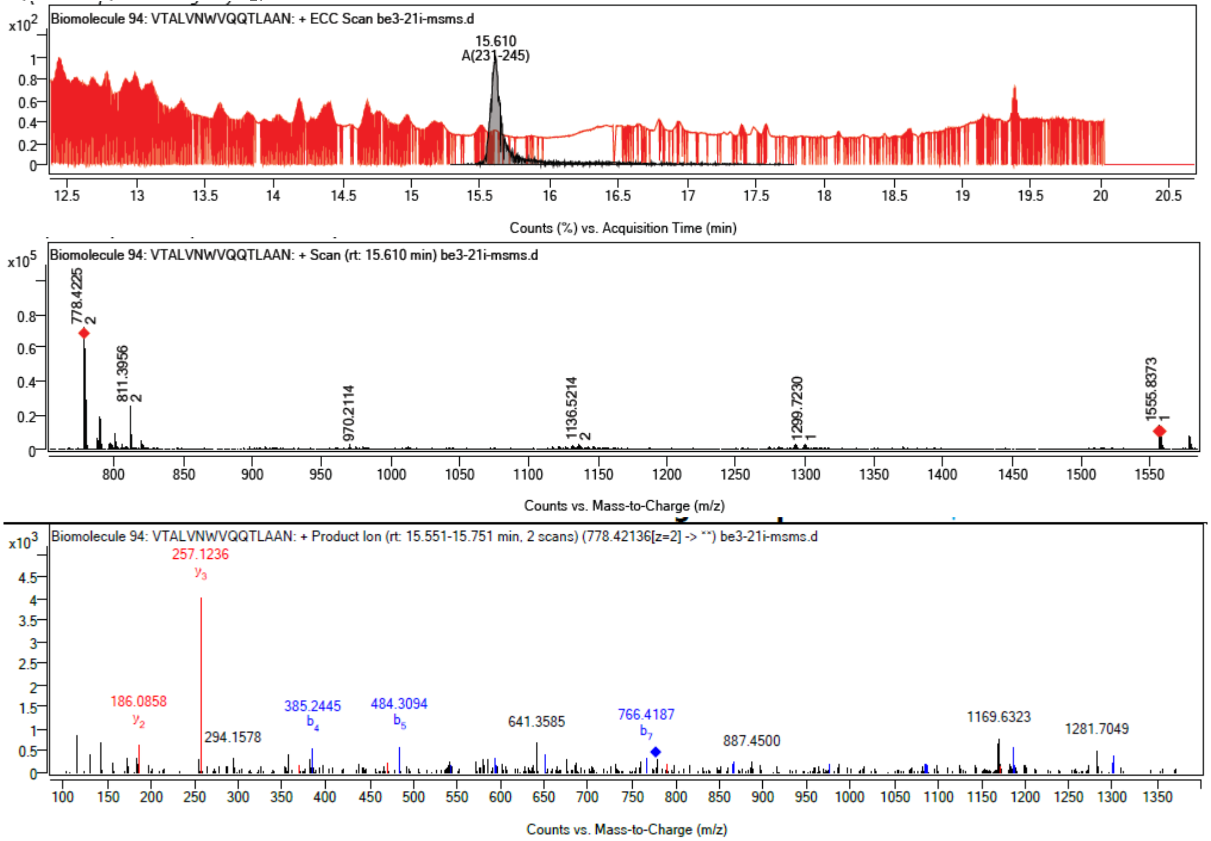

Fragment Spectrum Peaks

m/z	Diff (ppm)	Abund	Ion
186.0858	8.27	631	y2
257.1236	3.05	4021	y3
370.2137	-14.07	163	y4
471.2573	-2.28	204	y5
790.4123	10.53	185	y8
1171.6427	-35.81	148	y11
385.2445	0.03	539	b4
484.3094	7.25	582	b5
766.4187	7.70	332	b7
866.4795	19.46	232	b8
975.5407	0.42	194	b9
1085.5763	11.73	184	b10
1186.6320	3.97	571	b11
1300.7275	-2.54	376	b12
543.3022	-7.50	169	b10
593.8312	-15.44	325	b11
650.3634	1.06	409	b12

Nitrile Formation on Carbonic Anhydrase

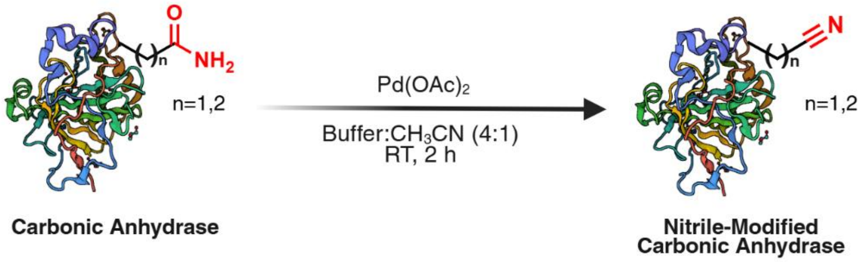

Reaction conducted according to **GP-XV**. Carbonic Anhydrase (2 mg, 68.9 μM) was dissolved in 800 μL of NaP buffer (10 mM, pH 7.4) and 200 μL of $Pd(OAc)_2$ (1.12 mg, 5 mM) dissolved in ACN was added. The reaction was stirred at room temperature for 2 h followed by quenching with

500 μL of 1 M solution of aqueous L-cysteine and 10 μL of 1 M NaOH solution. The crude reaction mixture was passed through an Amicon™ Ultra 3 kDa centrifugal filter and washed with H_2O (7×0.5 mL) to remove the water-soluble Pd complex. The labeled protein was digested using SMART Digest™ Trypsin Kit by Thermo Scientific, and analyzed using LC-MS/MS. The total number of nitrile-containing sites was observed to be 14 with >95% overall conversion to nitrile.

MS of Unmodified Carbonic Anhydrase

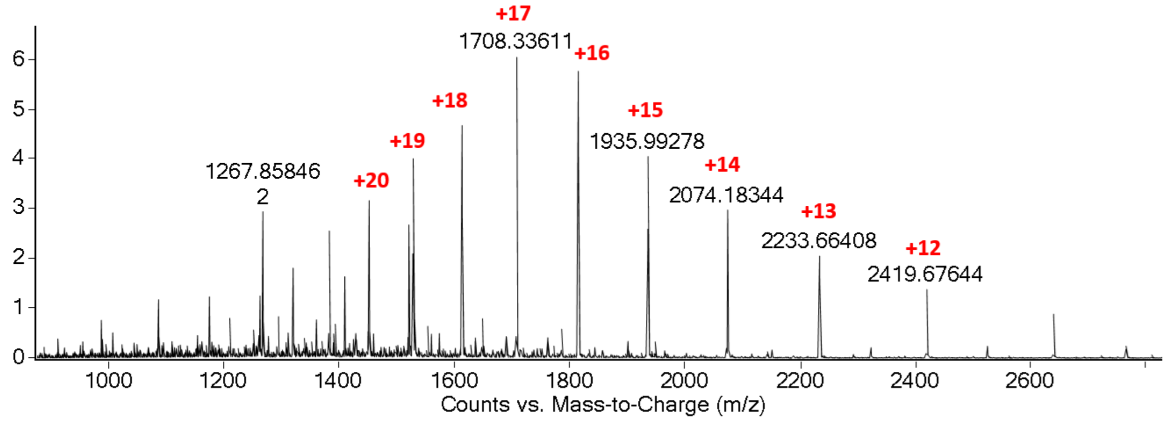

Deconvoluted MS of Unmodified Carbonic Anhydrase

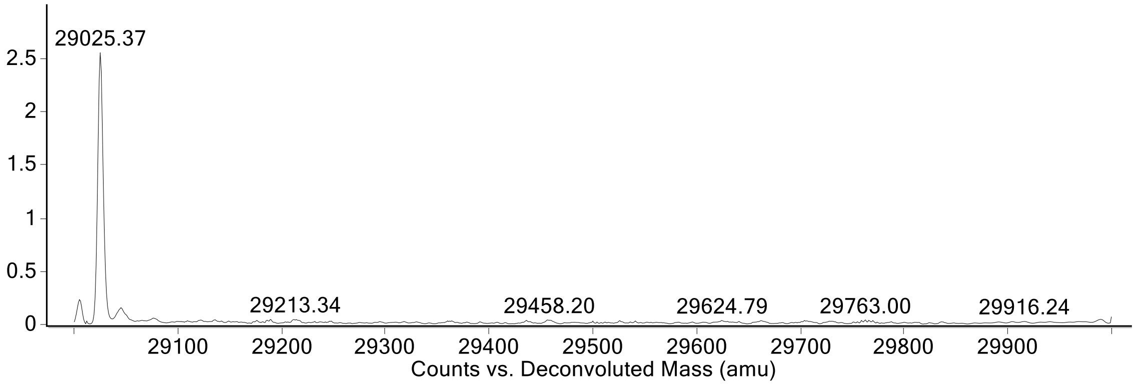

MS/MS Analysis of Digested Modified Carbonic Anhydrase

Identified peptide fragment **(1 site)**: DFPIANGER: (Sequence: AA 18-26, N23)

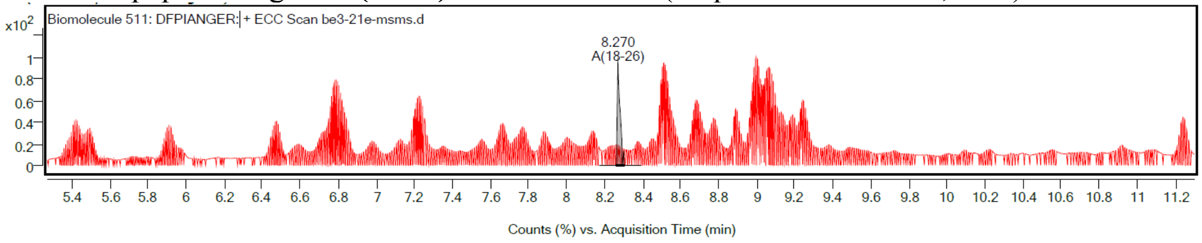

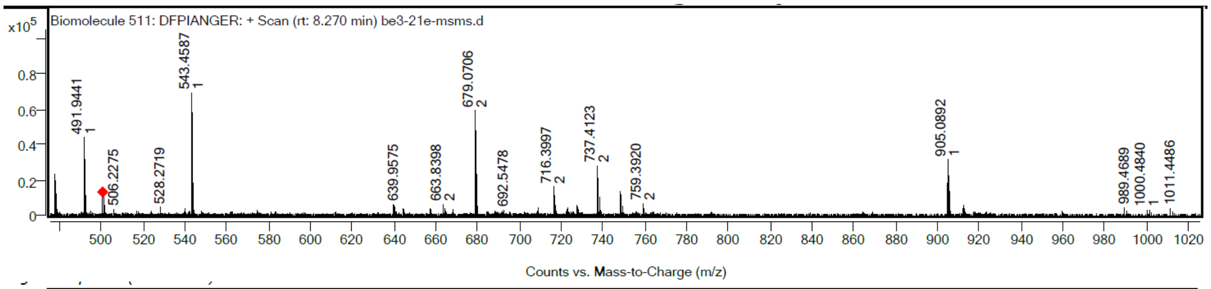

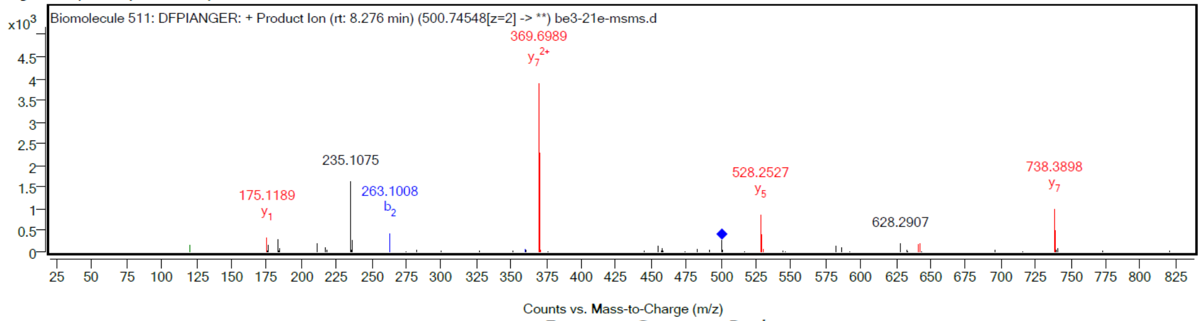

Fragment Spectrum Peaks

m/z	Diff (ppm)	Abund	Ion
175.1189	0.39	332	y1
528.2527	-0.34	859	y5
642.3217	28.36	194	y6
738.3898	-0.63	992	y7
369.6989	-1.76	3888	y7
263.1008	6.96	425	b2
360.1575	-5.86	58	b3
120.0798	8.23	156	F

Identified peptide fragment **(1 site)**: AVVQDPALKPL (Sequence: AA 36-46, Q39)

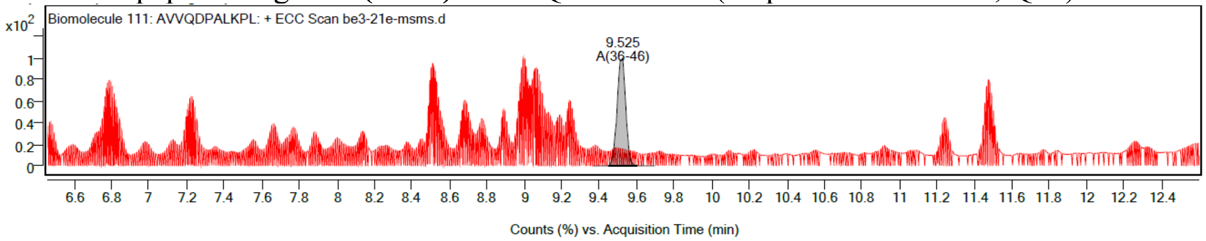

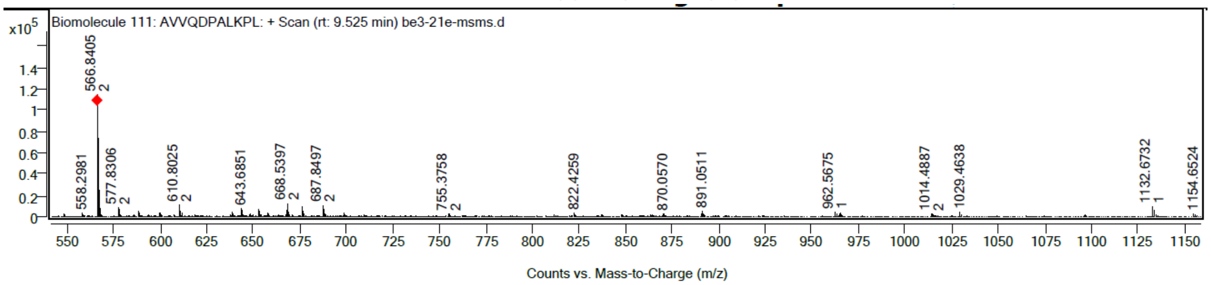

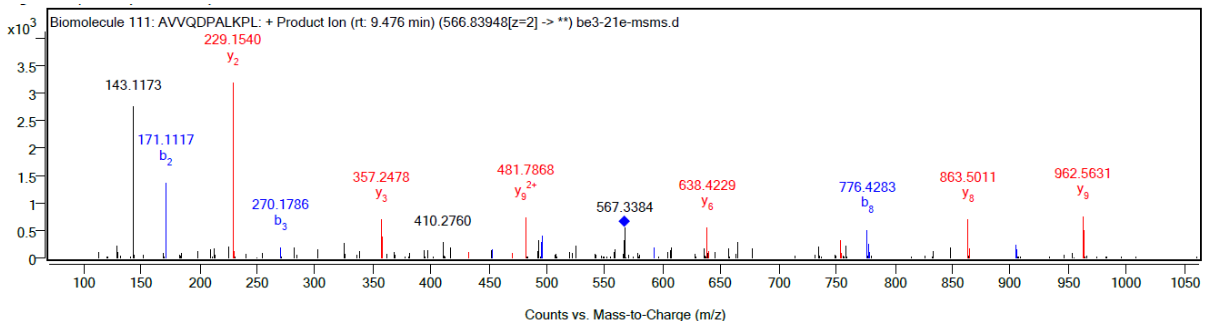

Fragment Spectrum Peaks

m/z	Diff (ppm)	Abund	Ion
229.1540	2.92	3177	y2
357.2478	5.18	690	y3
470.3375	-8.05	77	y4
638.4229	1.08	537	y6
753.4507	-0.28	307	y7
863.5011	-2.95	686	y8
962.5631	3.99	750	y9
432.2551	-5.14	93	y8
481.7868	0.74	726	y9
171.1117	6.23	1360	b2
270.1786	9.81	179	b3
496.2536	11.97	390	b5
592.3188	-16.57	182	b6
776.4283	2.30	502	b8
904.5289	-4.20	234	b9
452.7619	9.54	144	b9

Identified peptide fragment **(2 sites)**: MVNNGHSFNVEYDDSQDK (Sequence: AA 58-75, [N66, Q73)

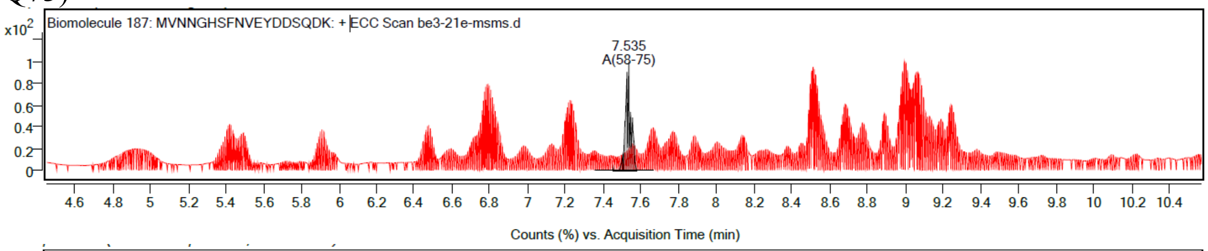

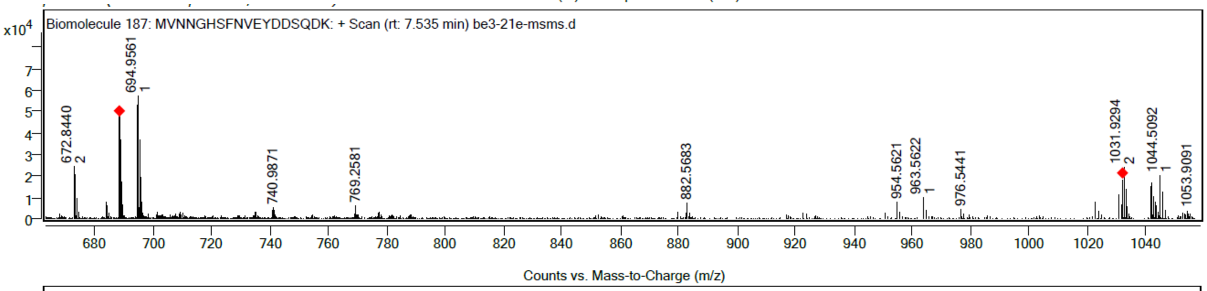

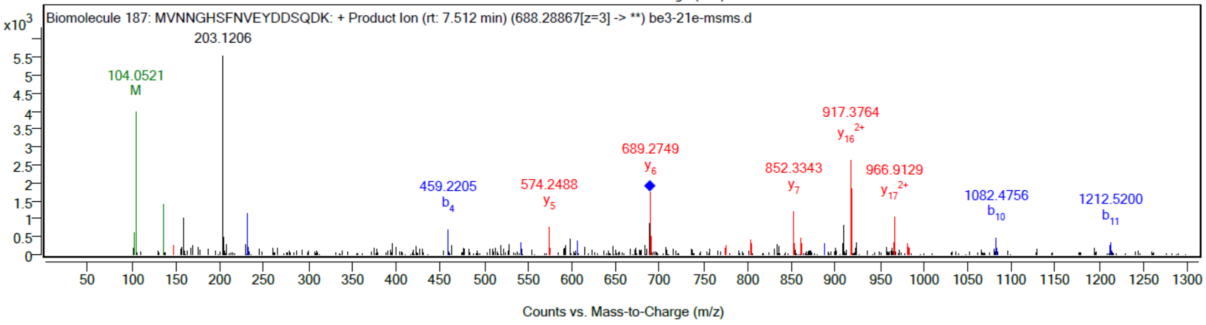

Fragment Spectrum Peaks

m/z	Diff (ppm)	Abund	Ion
147.1109	12.62	253	y1
574.2488	-3.55	758	y5
689.2749	-1.74	1739	y6
852.3343	3.15	1202	y7
981.4150	-36.11	305	y8
774.8168	10.83	248	y13
802.8337	0.70	417	y14
859.8613	-6.56	459	y15
917.3764	2.70	2636	y16
966.9129	0.16	1039	y17
231.1165	-1.23	1157	b2
459.2205	-40.13	693	b4
887.3558	30.45	306	b8
983.4019	-15.88	187	b9
1082.4756	7.41	451	b10
1212.5200	7.86	330	b11
541.7471	-2.99	323	b10
606.2655	2.08	373	b11
102.0548	1.46	625	E
104.0521	7.15	3981	M
136.0756	0.52	1415	Y
688.2909	-1.05	858	Precursor
688.6195	7.51	816	Precursor
688.9579	1.74	545	Precursor
689.2939	-0.59	370	Precursor

Identified peptide fragment **(1 site)**: LVQFHFH (Sequence: AA 89-95, Q91)

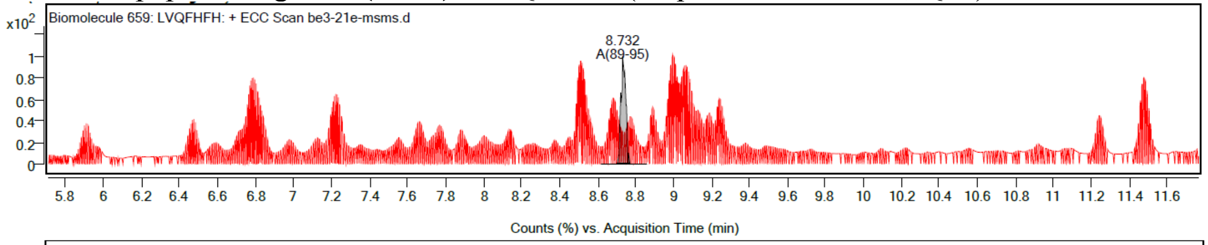

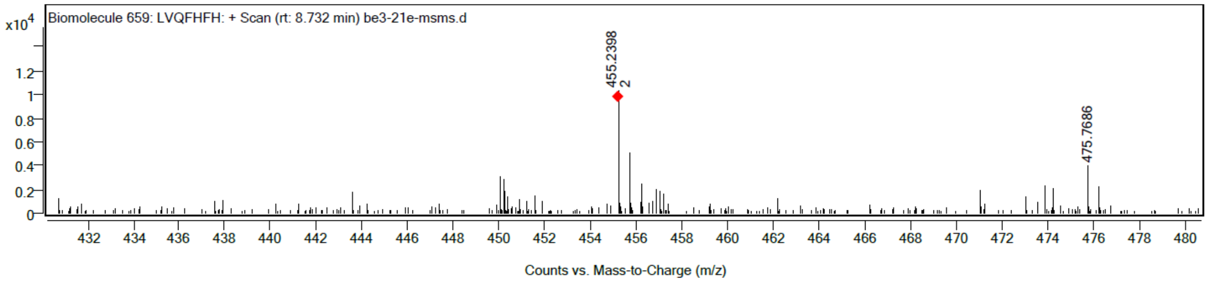

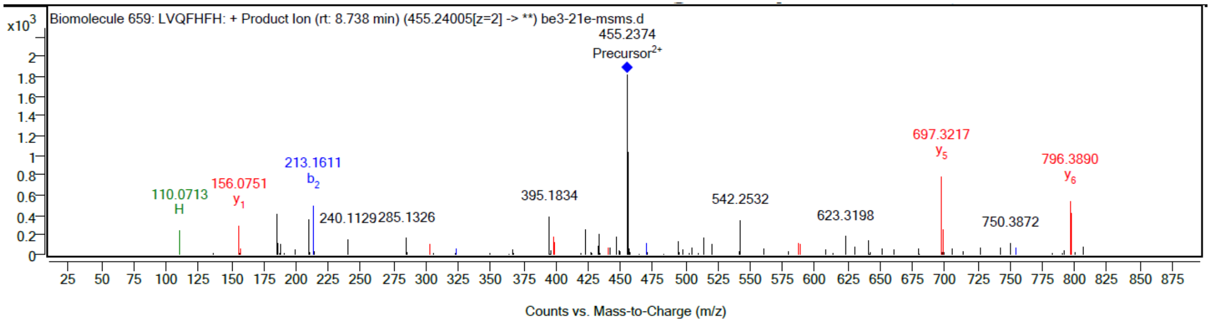

Fragment Spectrum Peaks

m/z	Diff (ppm)	Abund	Ion
156.0751	10.72	286	y1
303.1459	-2.46	102	y2
440.2003	8.65	67	y3
587.2786	-10.41	113	y4
697.3217	-1.77	780	y5
796.3890	-0.14	531	y6
398.6941	9.92	181	y6
213.1611	-6.13	493	b2
323.2029	15.11	61	b3
470.2658	22.04	108	b4
754.3992	5.77	67	b6
110.0713	-0.67	243	H
455.2374	5.90	1816	Precursor
455.7423	-1.02	1031	Precursor
456.2474	-8.65	62	Precursor

Identified peptide fragment **(2 sites)**: LVQFHFHWGSSDQGSEHTVDRK (Sequence: AA 89-110, [Q91, Q101])

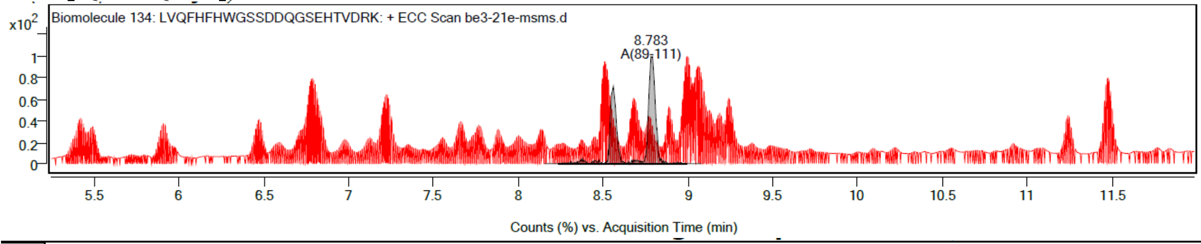

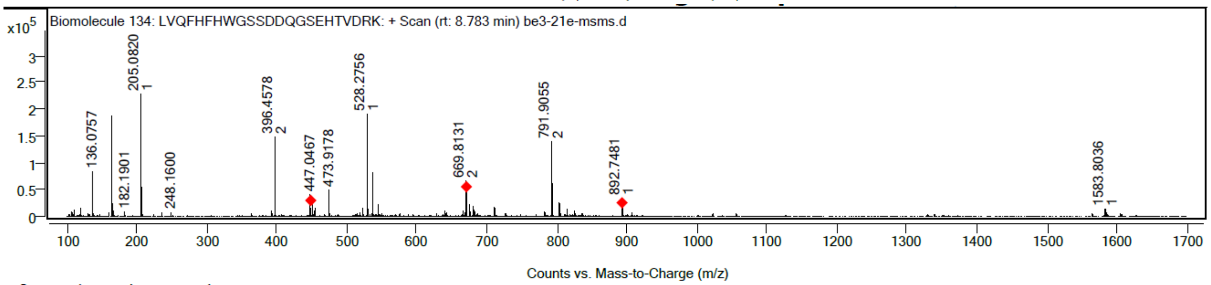

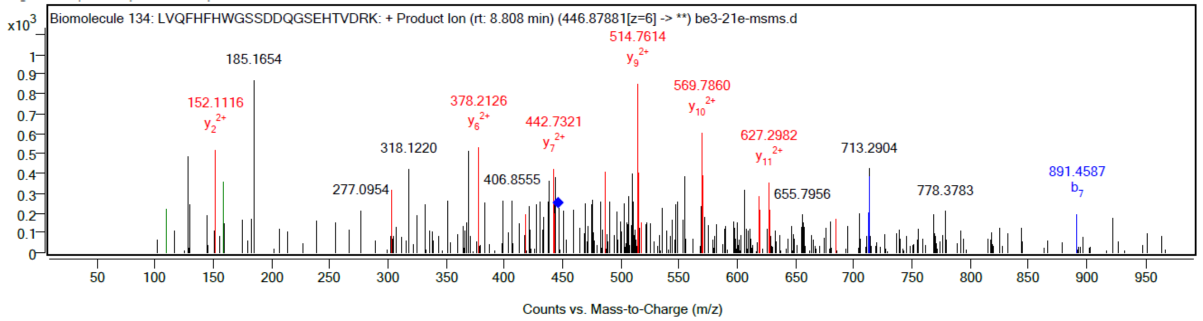

Fragment Spectrum Peaks

m/z	Diff (ppm)	Abund	Ion
303.2147	-2.64	312	y2
418.2420	-2.75	192	y3
517.3164	-13.76	150	y4
618.3569	0.12	281	y5
152.1116	-6.38	517	y2
378.2126	-2.79	527	y6
442.7321	1.79	421	y7
486.2491	-0.39	404	y8
514.7614	-3.46	850	y9
569.7860	-4.12	604	y10
627.2982	-1.83	352	y11
684.8108	-0.38	170	y12
891.4587	4.14	192	b7
713.3027	43.97	383	b12
110.0706	6.42	220	H
159.0921	-2.55	355	W

Identified peptide fragment **(1 site)**: YAAELHLVHWN (Sequence: AA 112-122, N122)

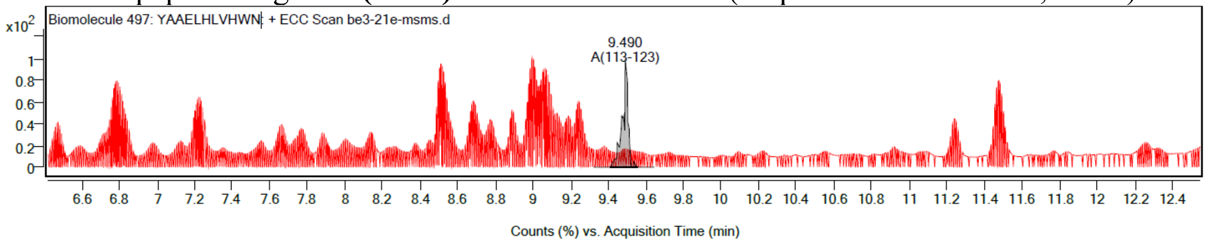

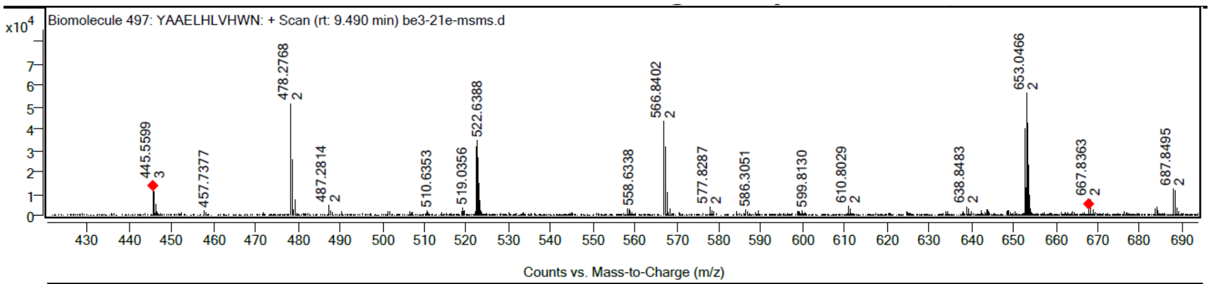

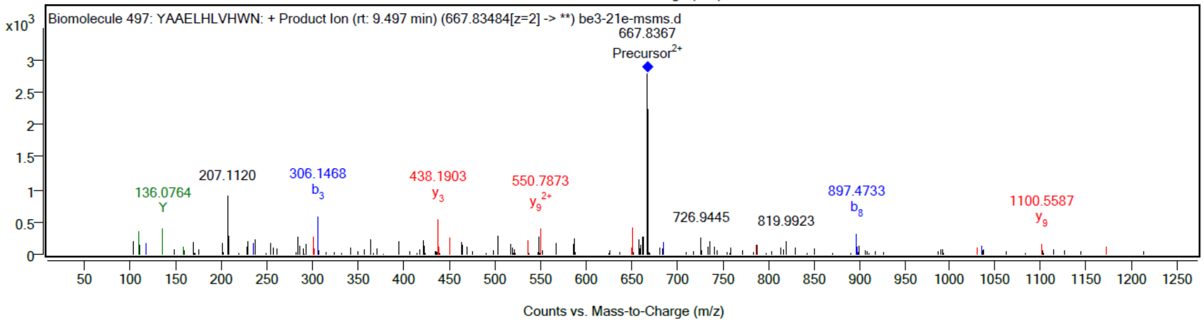

Fragment Spectrum Peaks

m/z	Diff (ppm)	Abund	Ion
301.1258	12.36	271	y2
438.1903	-4.30	532	y3
537.2600	-5.96	211	y4
651.3180	40.40	406	y5
787.4057	-7.48	141	y6
1029.5403	-13.39	100	y8
1100.5587	4.48	159	y9
1171.6048	-3.53	114	y10
450.7395	13.38	247	y7
550.7873	-3.46	391	y9
235.1075	0.96	172	b2
306.1468	-6.43	573	b3
685.3363	-8.59	181	b6
897.4733	10.69	311	b8
1034.5408	1.00	127	b9
118.0630	-46.72	172	b2
110.0706	5.74	345	H
136.0764	-5.54	391	Y
159.0939	-13.94	108	W
667.8367	-1.51	2768	Precursor
668.3348	3.85	2223	Precursor
668.8448	-8.73	310	Precursor

Identified peptide fragment **(1 site)**: YGDFGTAAQQPDGLAVVGVFLK (Sequence: AA 125-146, Q134)

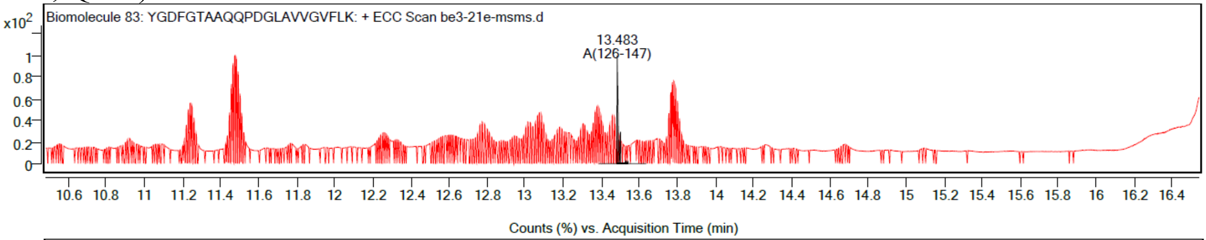

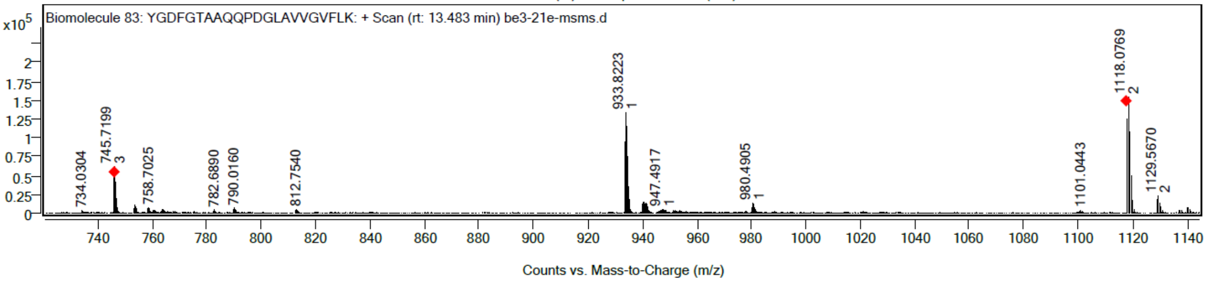

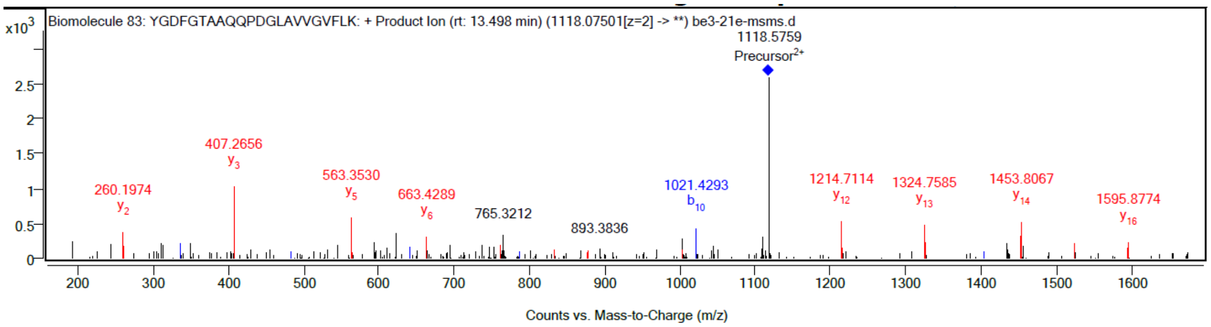

Fragment Spectrum Peaks

m/z	Diff (ppm)	Abund	Ion
260.1974	-2.08	366	y2
407.2656	-0.72	1018	y3
563.3530	3.90	576	y5
663.4289	-2.90	300	y6
761.4954	-4.49	190	y7
832.5240	6.14	116	y8
1002.6294	5.25	122	y10
1214.7114	2.45	523	y12
1324.7585	2.94	471	y13
1453.8067	12.11	520	y14
1523.8605	-1.60	216	y15
1595.8774	13.23	227	y16
877.4859	1.82	110	y18
336.1140	15.01	217	b3
483.1929	-11.29	95	b4
641.2452	17.71	162	b6
1021.4293	7.89	428	b10
1403.6134	6.58	97	b14
787.3638	4.96	96	b16
1118.0742	1.52	1365	Precursor
1118.5759	1.46	2586	Precursor
1119.0820	-2.47	612	Precursor

Identified peptide fragment **(2 sites)**: STDFPNFDPGSLLPNVLDYWTYPGSLTTPPLLESVTWIVLK (Sequence: AA 170-210, [N175, N184])

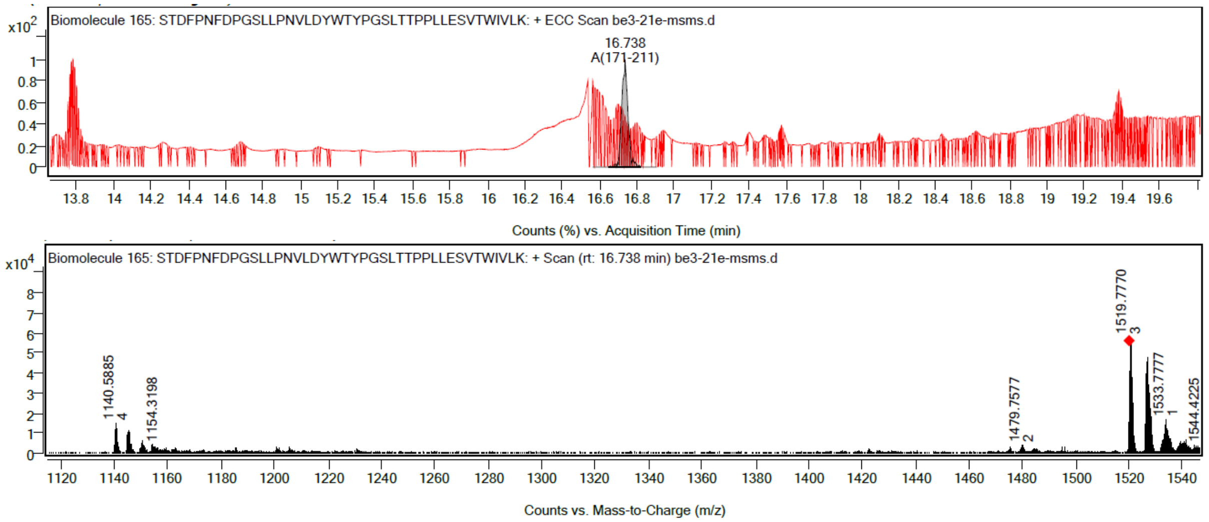

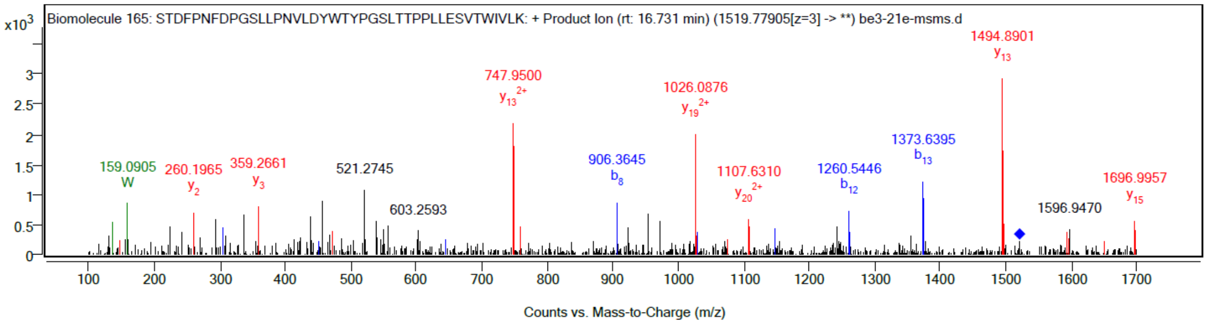

Fragment Spectrum Peaks

m/z	Diff (ppm)	Abund	Ion
147.1132	-2.91	230	y1
260.1965	1.41	695	y2
359.2661	-2.34	797	y3
472.3535	-8.79	389	y4
759.4789	-3.33	464	y6
1074.6120	6.89	248	y9
1494.8901	1.97	2916	y13
1696.9957	-4.28	556	y15
747.9500	0.22	2180	y13
1026.0876	5.28	1990	y19
1107.6310	-5.71	584	y20
1593.3239	18.76	375	y28
1649.8926	1.93	217	y29
304.1126	4.51	448	b3
451.1810	2.95	211	b4
644.2673	0.30	255	b6
906.3645	-1.90	863	b8
1147.4537	13.38	434	b11
1260.5446	6.79	714	b12
1373.6395	-1.69	1199	b13
1029.4803	7.35	366	b19
136.0748	6.52	533	Y
159.0905	7.41	849	W

Identified peptide fragment **(2 sites)**: EPISVSSQQMLK (Sequence: AA 212-223, [Q219, Q220])

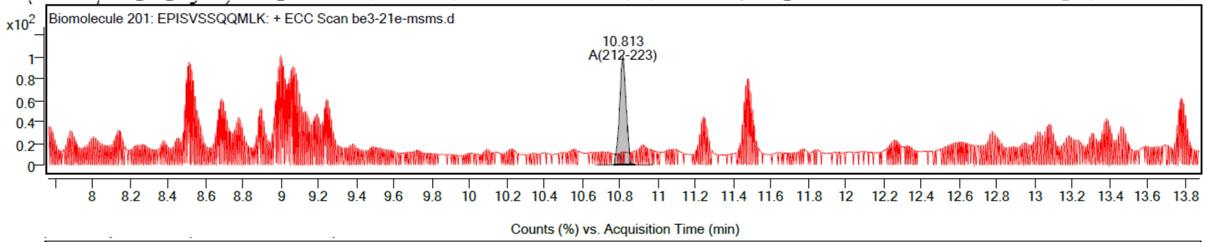

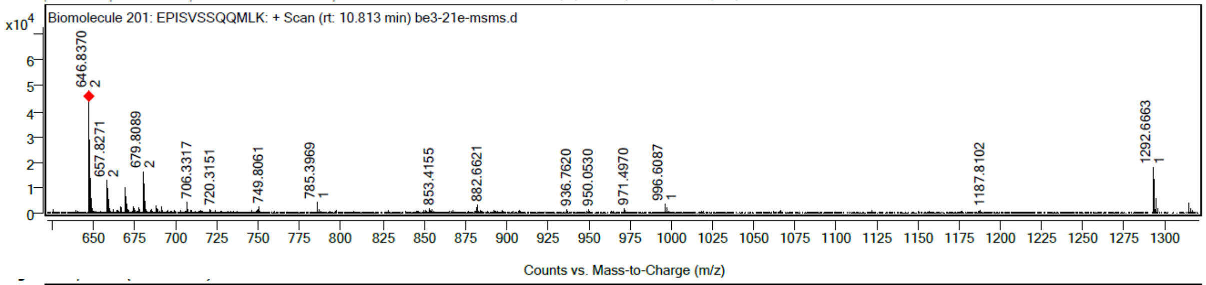

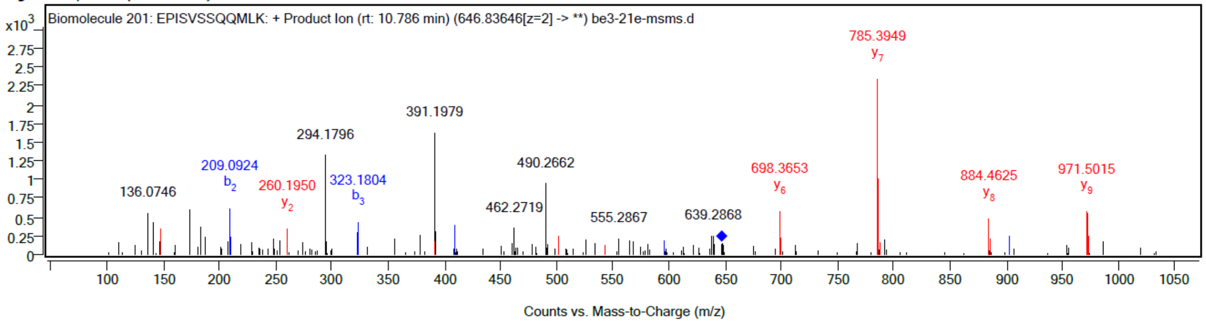

Fragment Spectrum Peaks

m/z	Diff (ppm)	Abund	Ion
147.1107	14.26	336	y1
260.1950	7.07	340	y2
391.2258	29.61	169	y3
501.2841	2.57	240	y4
698.3653	0.09	571	y6
785.3949	3.28	2338	y7
884.4625	3.83	474	y8
971.5015	-3.72	565	y9
542.7935	2.00	117	y10
209.0924	-1.49	611	b2
323.1804	-2.92	422	b3
409.2072	2.34	385	b4
595.3069	2.81	182	b6
902.4351	1.75	238	b9

Identified peptide fragment **(2 sites)**: TLNFNAEGEPELLMLANWR (Sequence: AA 226-244, [N228, N242])

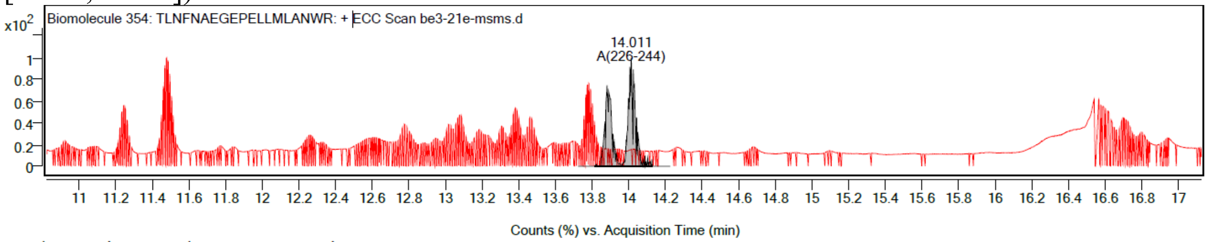

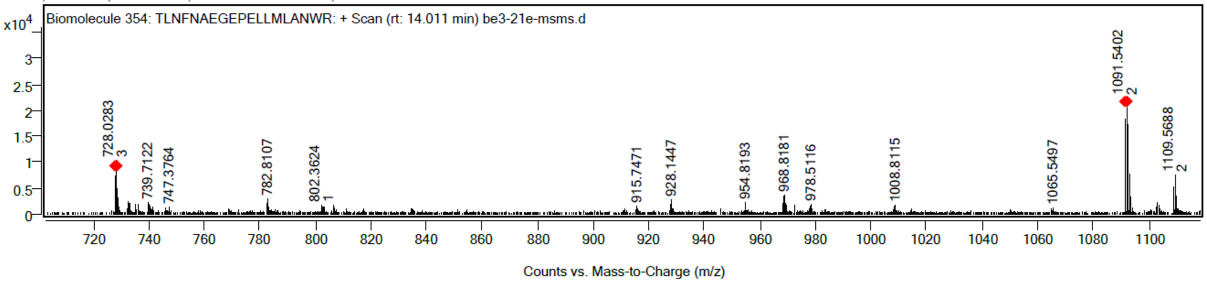

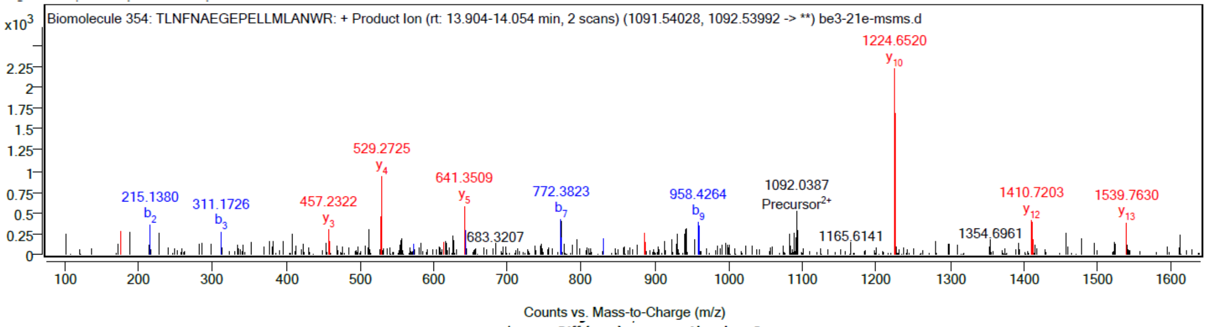

m/z	Diff (ppm)	Abund	Ion
175.1164	14.49	274	y1
457.2322	-3.51	298	y3
529.2725	-2.58	932	y4
641.3509	1.42	578	y5
885.4744	2.19	253	y7
1224.6520	3.10	2221	y10
1410.7203	-0.36	405	y12
1539.7630	-0.39	380	y13
612.8256	9.70	141	y10
215.1380	4.66	351	b2
311.1726	-3.90	268	b3
572.2822	0.89	124	b5
643.3178	3.12	285	b6
772.3823	-25.72	416	b7
830.3816	6.83	188	b8
958.4264	0.10	381	b9
1091.5369	3.89	196	Precursor
1092.0387	3.73	523	Precursor
1092.5325	10.96	356	Precursor

Nitrile Formation on Creatine Kinase

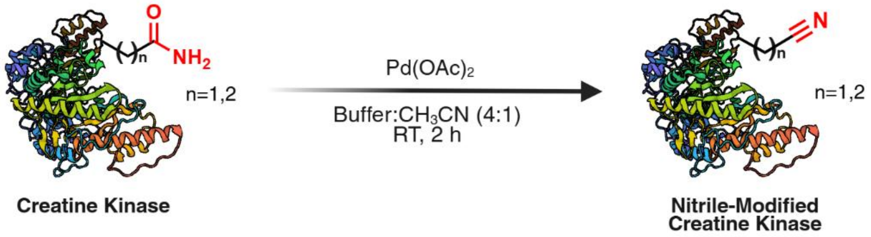

Reaction conducted according to **GP-XV**. Creatine Kinase (2 mg, 46.5 µM) was dissolved in 800 µL of NaP buffer (10 mM, pH 7.4) and 200 µL of $Pd(OAc)_2$ (1.12 mg, 5 mM) dissolved in ACN was added. The reaction was stirred at room temperature for 2 h followed by quenching with 500 µL of 1 M solution of aqueous L-cysteine and 10 µL of 1 M NaOH solution. The crude reaction mixture was passed through an Amicon™ Ultra 3 kDa centrifugal filter and washed with H_2O (7×0.5 mL) to remove the water-soluble Pd complex. The labeled protein was digested using SMART Digest™ Trypsin Kit by Thermo Scientific, and analyzed using LC-MS/MS. The total number of nitrile-containing sites was observed to be 11 with >95% overall conversion to nitrile.

MS of Unmodified Creatine Kinase

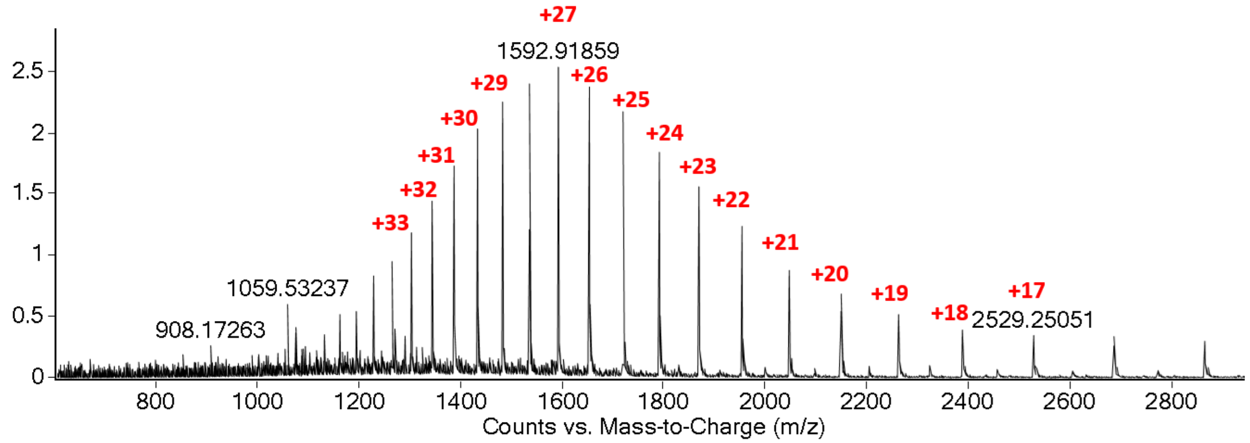

Deconvoluted MS of Unmodified Creatine Kinase

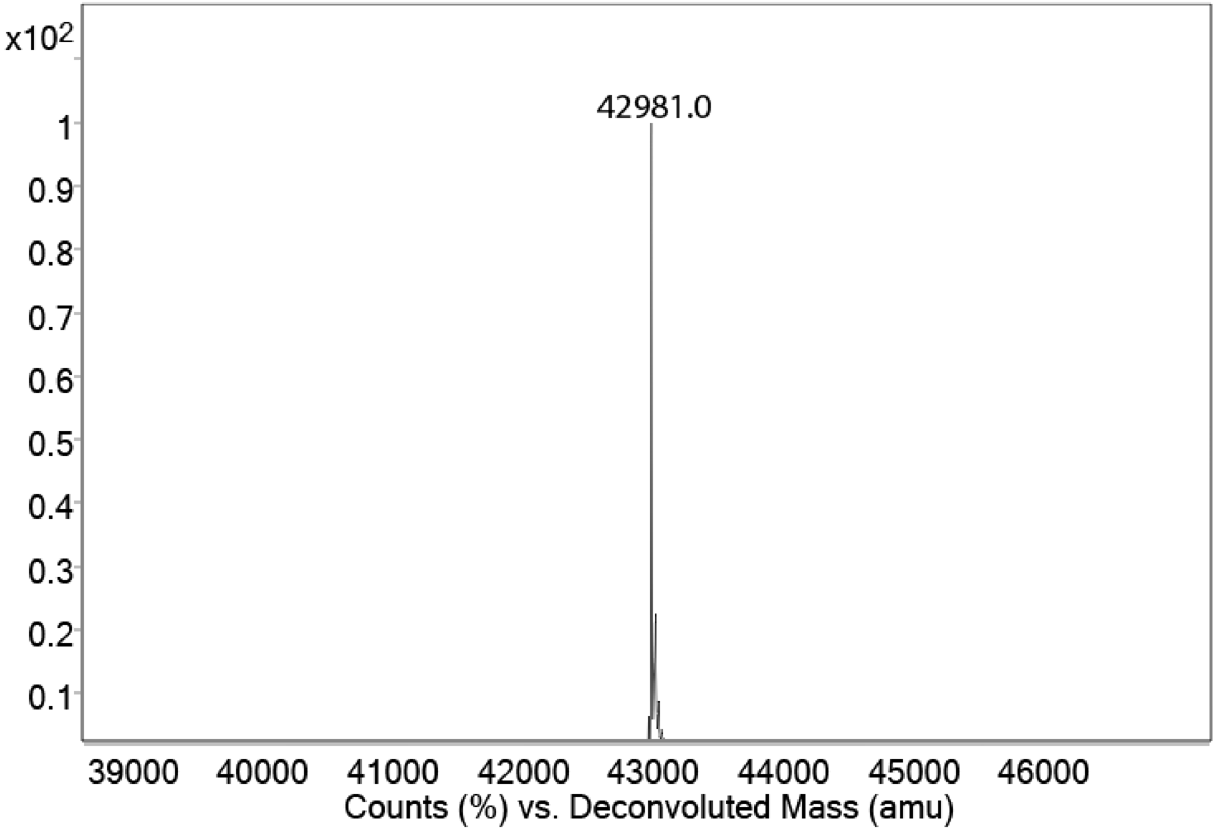

MS/MS Analysis of Digested Modified Creatine Kinase

Identified peptide fragment **(1 site)**:

ETPSGFTVDDVIQTGVDNPGHPFIMTVGCVAGDEESYEVFK (Sequence: AA 46-86, Q58)

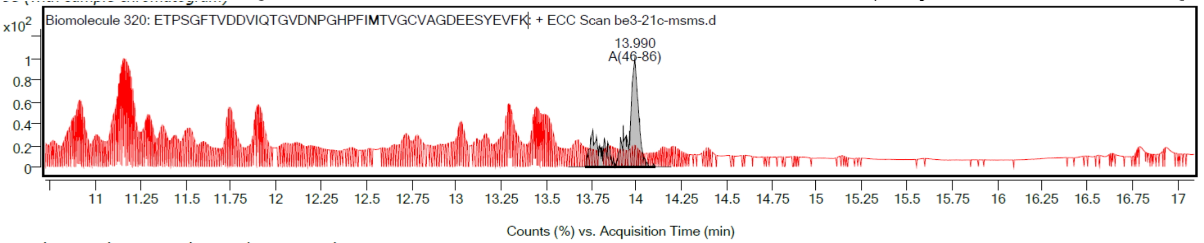

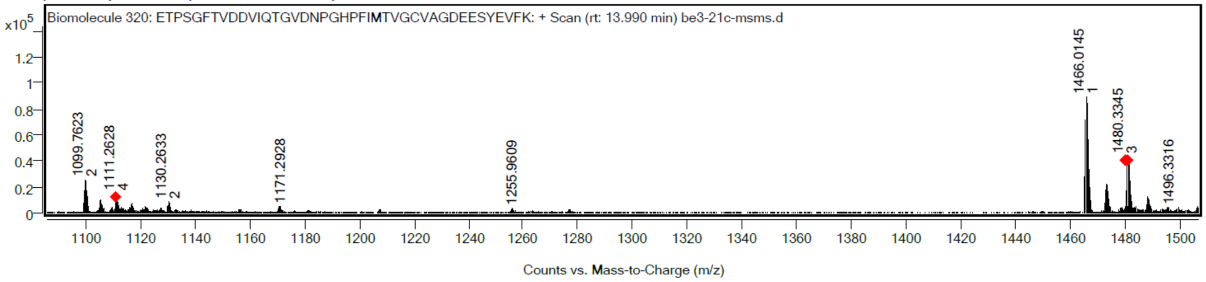

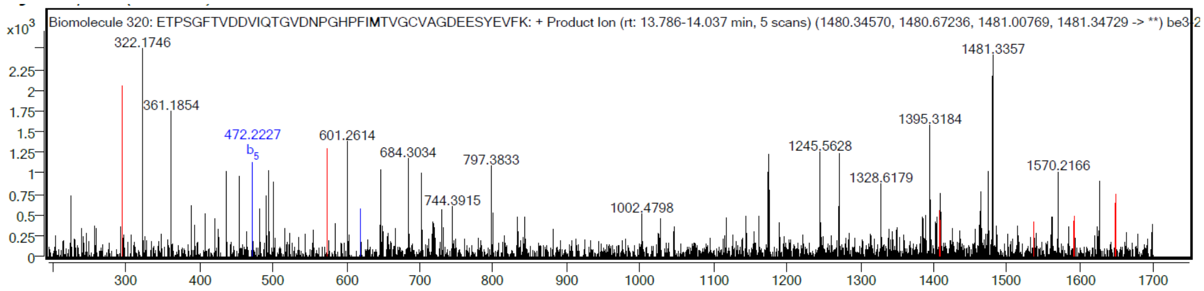

Fragment Spectrum Peaks

m/z	Diff (ppm)	Abund	Ion
294.1830	-6.14	2043	y2
573.2636	-17.71	1292	y9
1408.1134	7.96	551	y25
1536.6826	7.01	411	y28
1592.1964	14.21	483	y29
1648.7532	4.76	741	y30
472.2227	-39.93	1129	b5
619.2706	2.68	568	b6

Identified peptide fragment **(1 site)**: TDLNHENLK (Sequence: AA 108-116, N114)

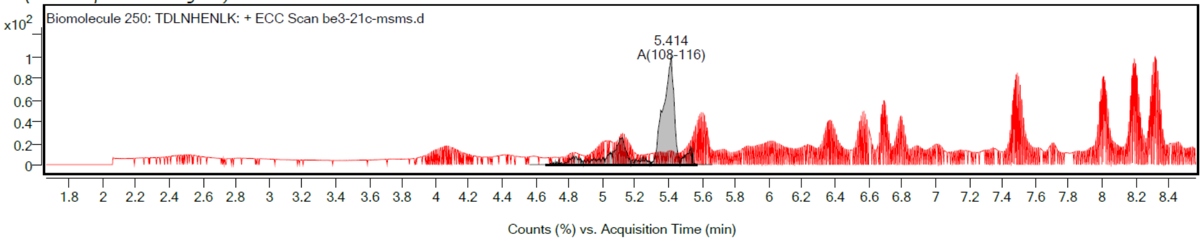

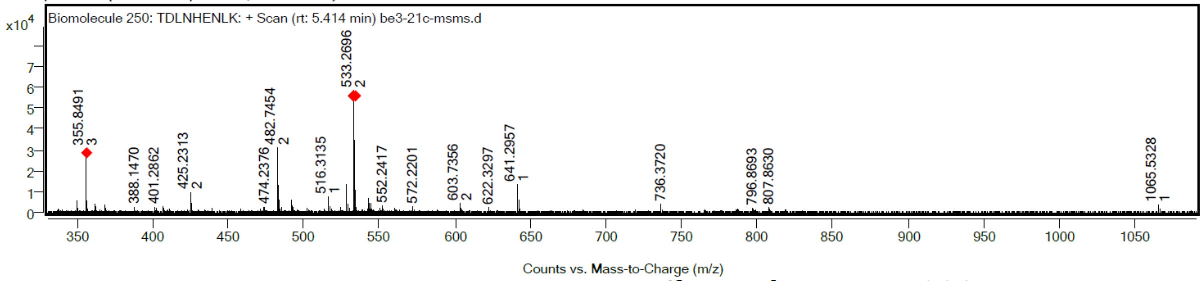

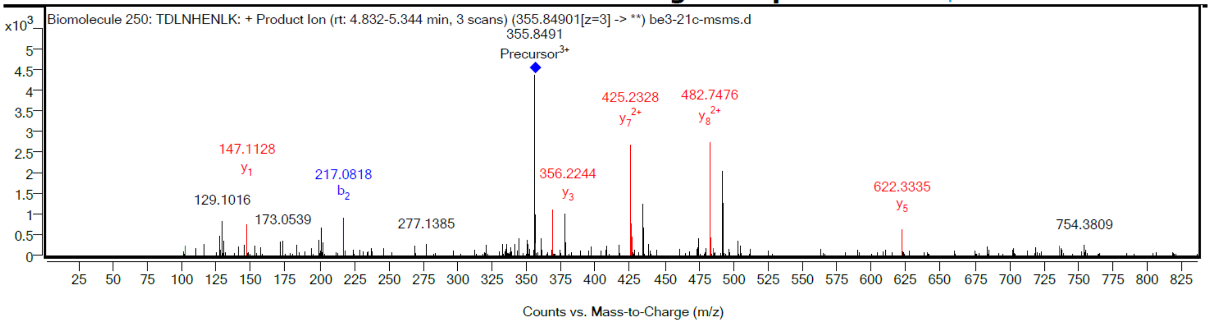

Fragment Spectrum Peaks

m/z	Diff (ppm)	Abund	Ion
147.1128	-0.24	742	y1
356.2244	13.53	277	y3
485.2691	5.67	152	y4
622.3335	-4.46	607	y5
736.3664	9.84	207	y6
368.6895	2.58	1106	y6
425.2328	-0.79	2689	y7
482.7476	-3.30	2737	y8
217.0818	0.43	903	b2
102.0523	26.29	218	E
355.8491	-0.44	4392	Precursor
356.1800	9.72	590	Precursor

Identified peptide fragment **(1 site)**: LSVEALNSLTGEFK (Sequence: AA 157-170, N163)

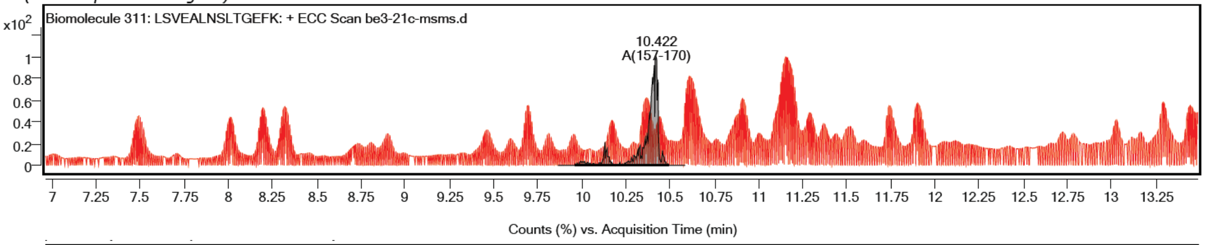

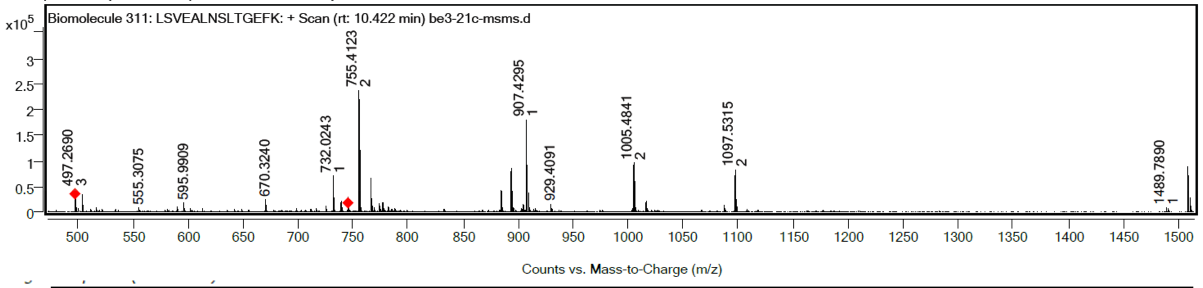

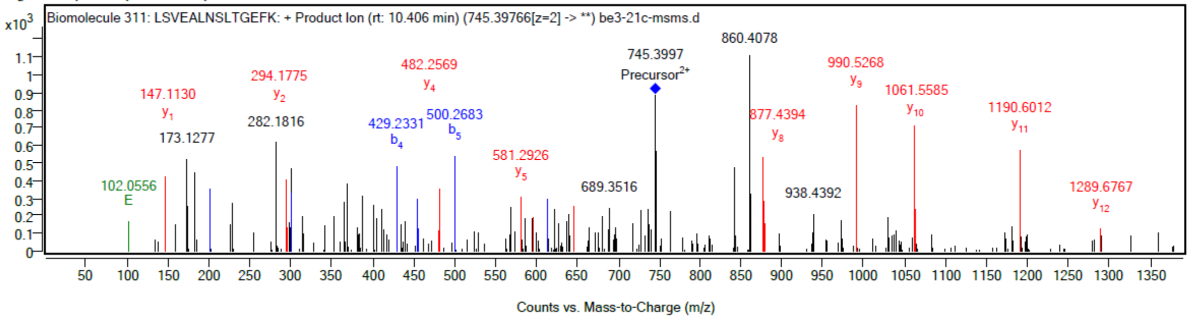

Fragment Spectrum Peaks

m/z	Diff (ppm)	Abund	Ion
147.1130	-1.27	419	y1
294.1775	12.50	402	y2
482.2569	-10.20	352	y4
581.2926	0.63	304	y5
877.4394	2.33	530	y8
990.5268	-1.37	825	y9
1061.5585	3.86	708	y10
1190.6012	3.36	571	y11
1289.6767	-2.41	125	y12
595.8005	9.59	190	y11
645.8410	1.78	251	y12
201.1215	9.51	352	b2
300.1908	3.31	329	b3
429.2331	2.87	478	b4
500.2683	6.37	534	b5
613.3517	6.33	292	b6
455.2434	26.94	294	b9
102.0556	-6.26	168	E
745.3997	-1.58	884	Precursor
745.8932	9.30	564	Precursor
746.4013	0.77	122	Precursor

Identified peptide fragment **(1 site)**: GIWHNDNKSFLVWVNEEDHLR (Sequence: AA 216-236, N222)

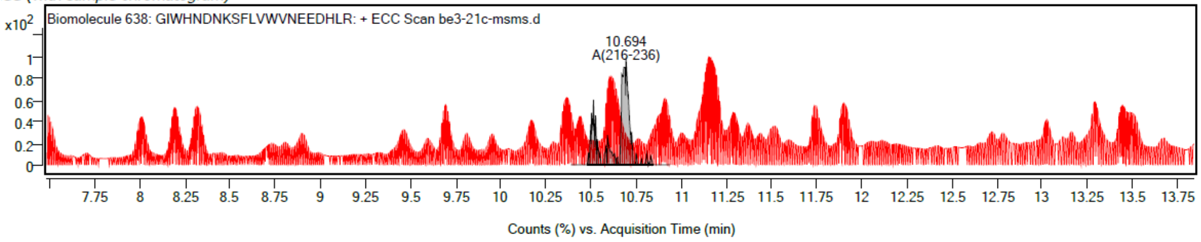

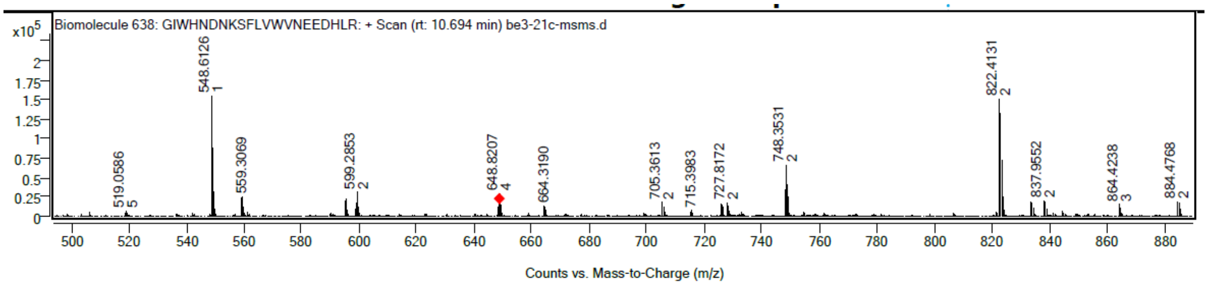

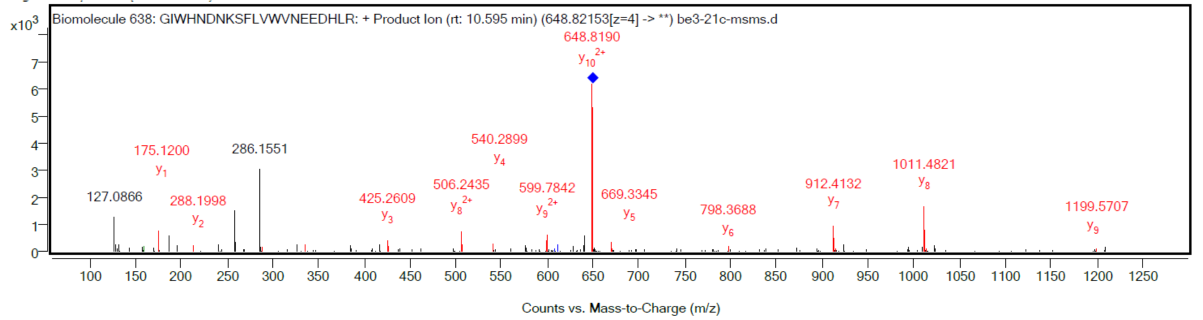

Fragment Spectrum Peaks

m/z	Diff (ppm)	Abund	Ion
175.1200	-5.96	778	y1
288.1998	11.19	153	y2
425.2609	2.53	408	y3
540.2899	-1.82	281	y4
669.3345	-4.55	350	y5
798.3688	6.54	188	y6
912.4132	4.13	949	y7
1011.4821	3.31	1671	y8
1199.5707	0.63	104	y9
213.1406	-28.16	240	y3
335.1694	0.01	257	y5
506.2435	5.58	734	y8
599.7842	5.80	624	y9
648.8190	1.88	6212	y10
611.2881	25.97	250	b5
159.0918	-0.48	193	W

Identified peptide fragment **(1 site)**: FCVGLQK (Sequence: AA 253-259, Q258)

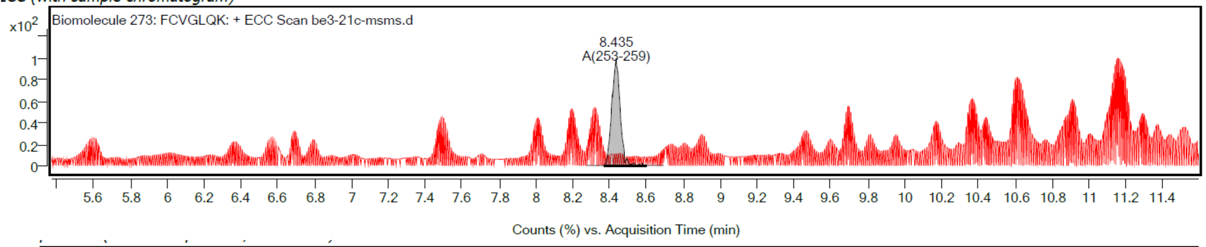

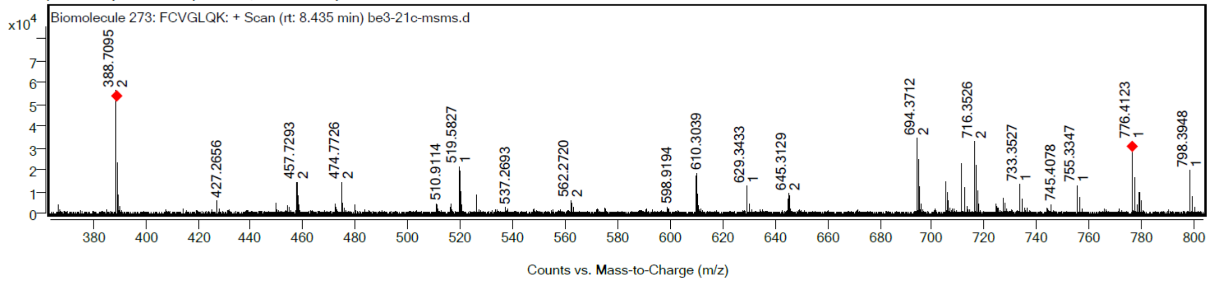

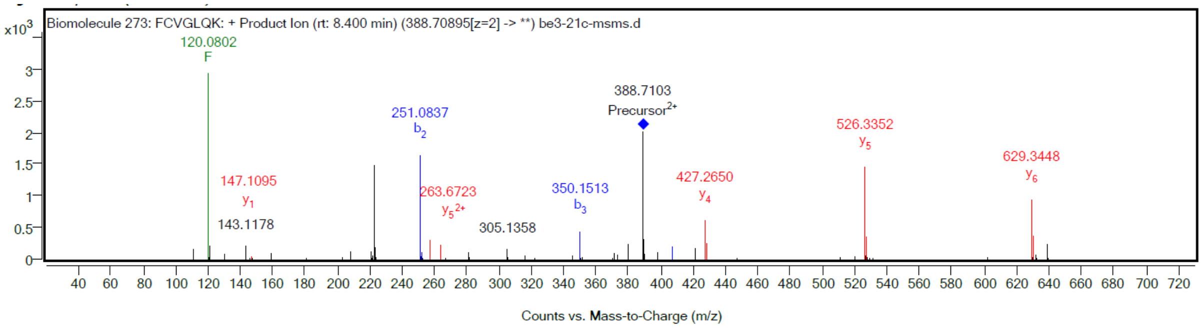

Fragment Spectrum Peaks

m/z	Diff (ppm)	Abund	Ion
147.1095	22.75	34	y1
257.1623	-5.76	296	y2
427.2650	3.19	609	y4
526.3352	-0.86	1450	y5
629.3448	-1.42	932	y6
263.6723	-4.77	221	y5
251.0837	4.74	1631	b2
350.1513	5.78	428	b3
407.1767	-4.86	184	b4
630.2780	45.77	33	b6
120.0802	4.56	2919	F
388.7103	-1.27	2006	Precursor
389.2125	-2.58	312	Precursor

Identified peptide fragment **(2 sites)**: AGHPFMWNQHLGYVLTCPSNLGTGLR (Sequence: AA 267-292, [Q275, N286])

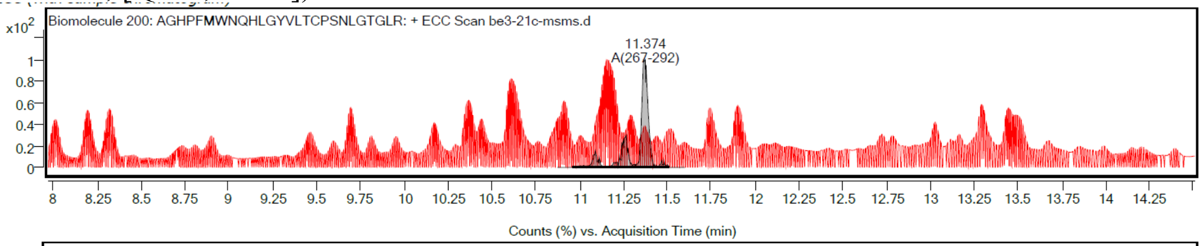

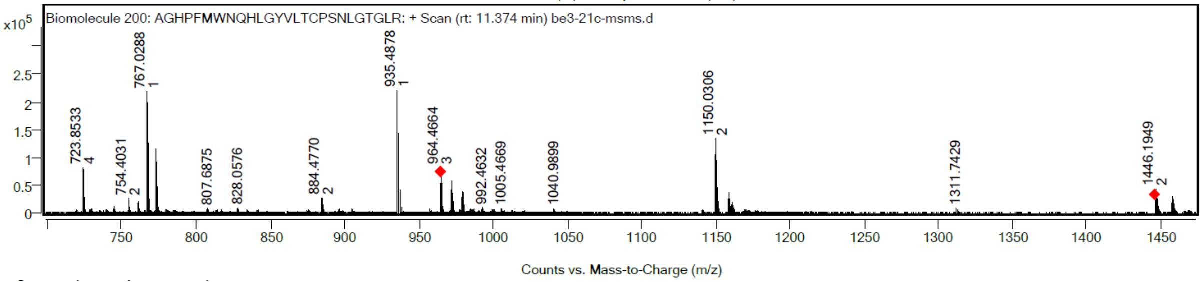

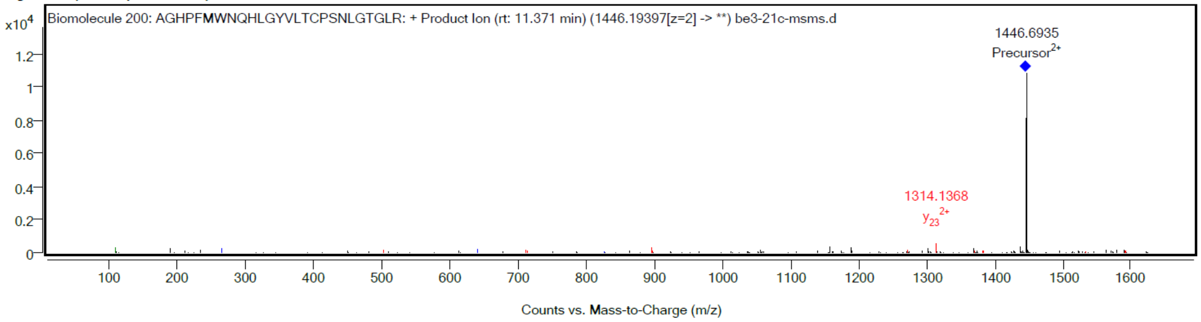

Fragment Spectrum Peaks

m/z	Diff (ppm)	Abund	Ion
503.2893	8.62	188	y5
712.4134	-4.65	150	y7
896.4924	2.70	314	y9
1271.6519	-8.37	147	y12
1370.7344	-18.02	118	y13
1533.7977	-16.10	90	y14
1591.8040	-3.88	117	y15
1314.1368	2.45	555	y23
1383.1638	5.28	110	y24
266.1214	12.65	269	b3
641.2870	-0.87	228	b6
827.3551	12.90	86	b7
110.0693	17.85	317	H
1446.1937	2.30	8126	Precursor
1446.6935	3.64	10827	Precursor
1447.1948	3.85	2931	Precursor
1447.7066	-3.13	329	Precursor

Identified peptide fragment **(2 sites)**: LGSSEVEQVQLVVDGVK (Sequence: AA 342-358, [Q349, Q351])

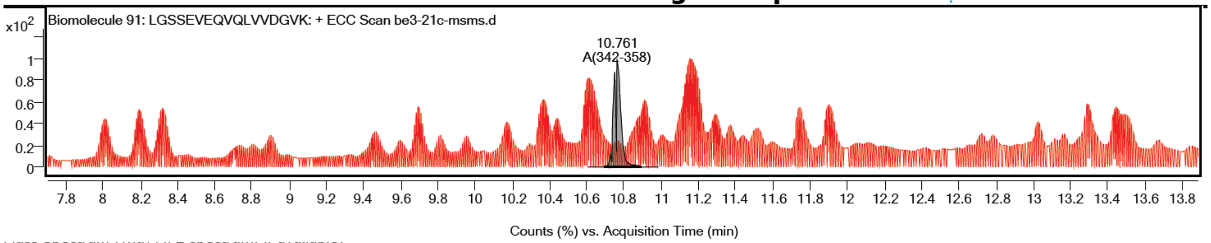

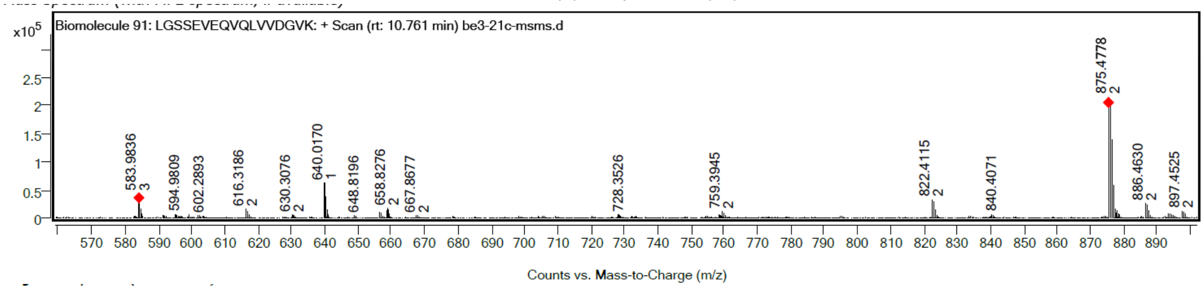

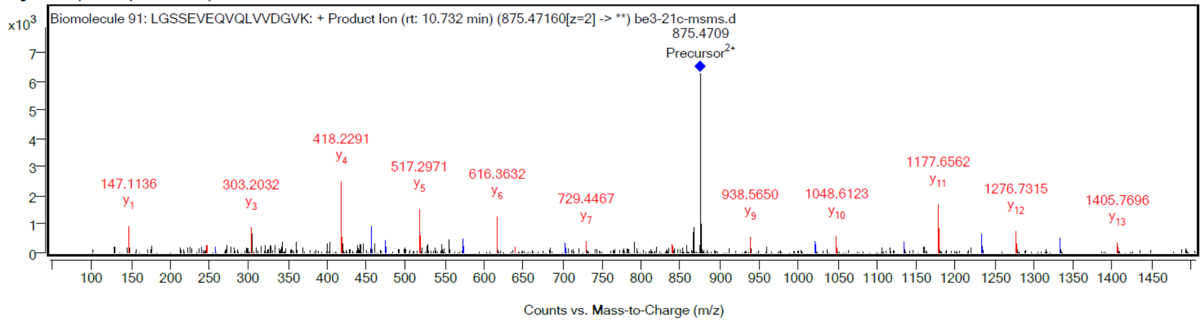

Fragment Spectrum Peaks

m/z	Diff (ppm)	Abund	Ion
147.1136	-5.27	917	y1
247.1844	0.81	271	y2
303.2032	-1.81	881	y3
418.2291	1.26	2493	y4
517.2971	1.74	1524	y5
616.3632	5.35	1267	y6
729.4467	5.29	392	y7
839.4992	-0.77	296	y8
938.5650	2.06	553	y9
1048.6123	2.56	566	y10
1177.6562	1.12	1679	y11
1276.7315	-4.35	746	y12
1405.7696	-0.70	338	y13
638.8666	0.07	216	y12
258.1437	4.24	219	b3
474.2211	-3.46	423	b5
573.2828	8.92	506	b6
702.3284	3.00	343	b7
1021.4968	-1.87	416	b10
1134.5672	10.38	379	b11
1233.6458	1.31	655	b12
1332.7118	2.97	518	b13
456.2060	46.25	909	b9
875.4709	2.08	6262	Precursor
875.9726	2.04	4818	Precursor
876.4850	-10.23	1018	Precursor

Identified peptide fragment **(2 sites)**: GQSIDDMIPAQK (Sequence: AA 370-381, [Q371, Q380])

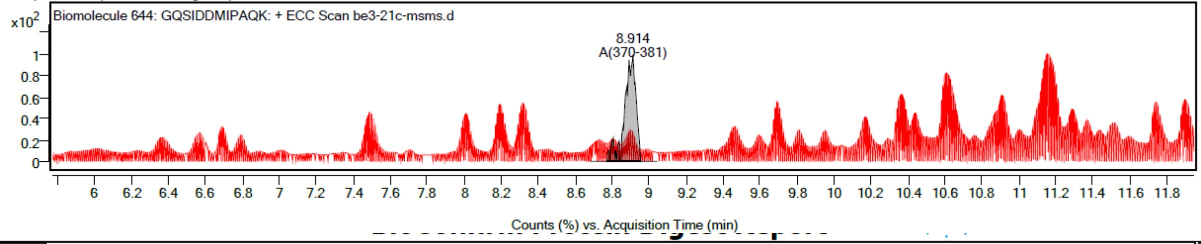

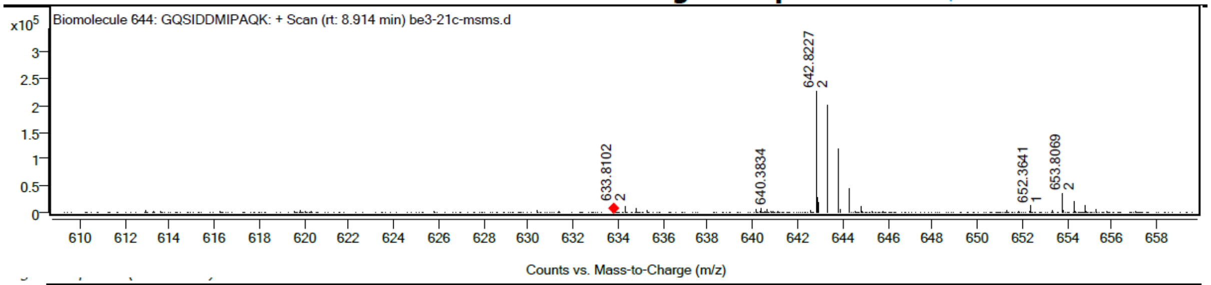

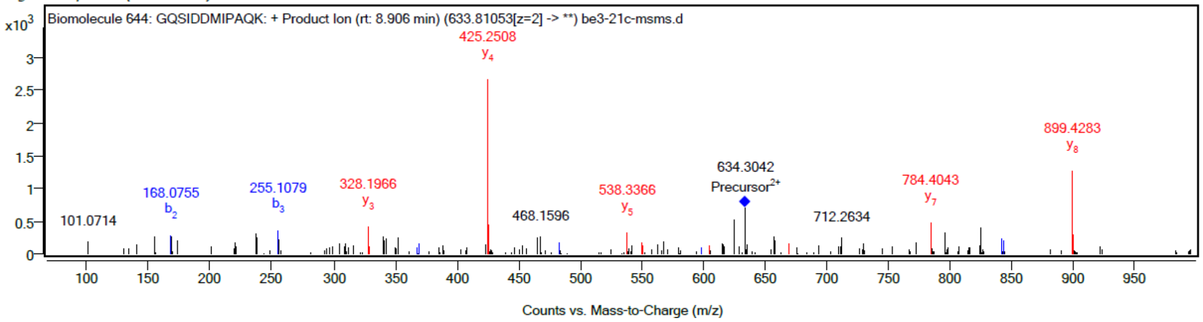

Fragment Spectrum Peaks

m/z	Diff (ppm)	Abund	Ion
328.1966	4.06	409	y3
425.2508	-0.33	2663	y4
538.3366	-3.50	316	y5
669.3743	1.39	154	y6
784.4043	-2.74	472	y7
899.4283	0.89	1269	y8
550.2683	14.54	165	y10
605.2966	6.10	120	y11
168.0755	7.28	274	b2
255.1079	3.35	356	b3
369.1850	30.44	156	b4
483.2154	9.13	175	b5
598.2445	3.66	87	b6
842.3743	-3.53	234	b8
633.8086	3.73	446	Precursor
634.3042	13.41	698	Precursor

Nitrile Formation on Bovine Serum Albumin (BSA)

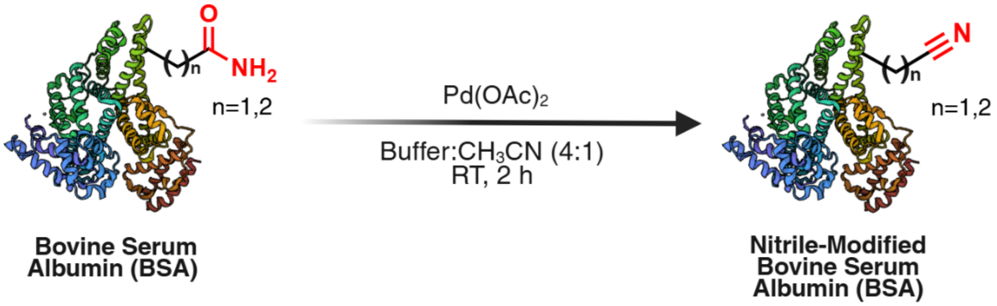

Reaction conducted according to **GP-XV**. Bovine Serum Albumin (2 mg, 30.1 µM) was dissolved in 800 µL of NaP buffer (10 mM, pH 7.4) and 200 µL of $Pd(OAc)_2$ (1.12 mg, 5 mM) dissolved in ACN was added. The reaction was stirred at room temperature for 2 h followed by quenching with 500 µL of 1 M solution of aqueous L-cysteine and 10 µL of 1 M NaOH solution. The crude reaction mixture was passed through an Amicon™ Ultra 3 kDa centrifugal filter and washed with H_2O (7×0.5 mL) to remove the water-soluble Pd complex. The labeled protein was digested using SMART Digest™ Trypsin Kit by Thermo Scientific, and analyzed using LC-MS/MS. The total number of nitrile-containing sites was observed to be 19 with >95% overall conversion to nitrile.

MS/MS Analysis of Digested Modified Bovine Serum Albumin

Identified peptide fragment (**3 sites**): GLVLIAFSQYLQQCPFDEHVK (Sequence: AA 21-41, [Q29, Q32, Q33])

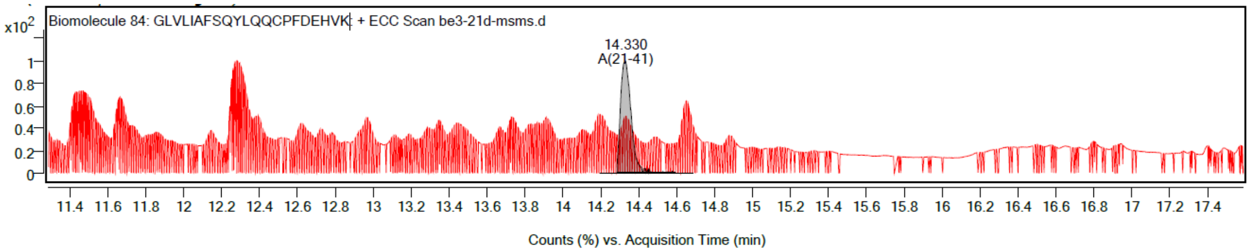

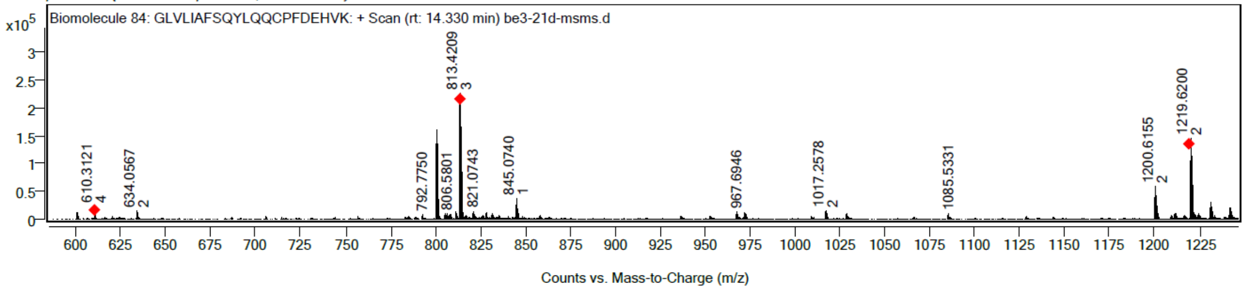

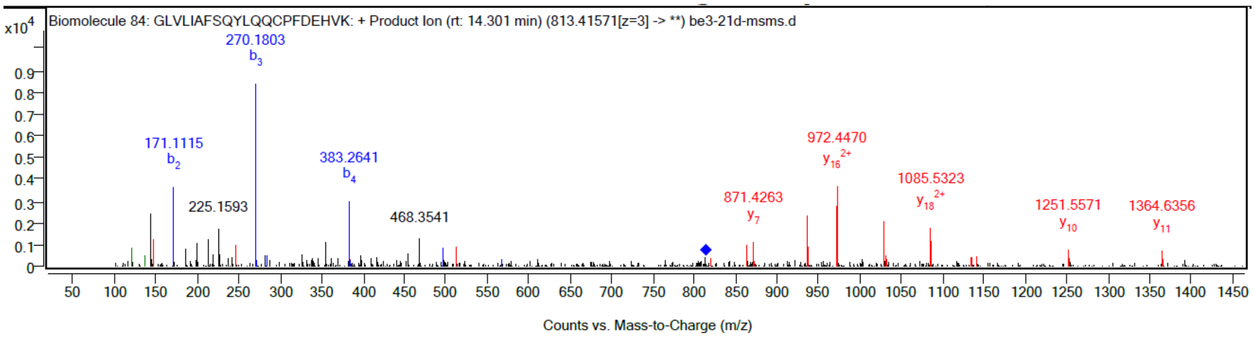

Fragment Spectrum Peaks

m/z	Diff (ppm)	Abund	Ion
147.1127	0.80	1213	y1
246.1807	2.27	947	y2
384.2388	12.12	325	y3
512.2807	3.94	898	y4
871.4263	5.17	1114	y7
1031.4644	-2.82	498	y8
1141.5084	1.00	446	y9
1251.5571	0.30	761	y10
1364.6356	4.36	702	y11
819.3838	-4.53	340	y13
863.3985	-0.85	966	y14
936.9277	4.60	2311	y15
972.4470	3.62	3651	y16
1028.4853	5.49	2037	y17
1085.5323	2.18	1767	y18
1134.5641	2.70	422	y19
171.1115	7.47	3605	b2
270.1803	3.40	8353	b3
383.2641	3.12	2964	b4
496.3520	-5.43	856	b5
567.3822	7.54	296	b6
284.1969	0.05	474	b6
120.0800	6.24	852	F
136.0754	2.06	479	Y

Identified peptide fragment (**1 site**): LVNELTEFAK (Sequence: AA 42-51, N44)

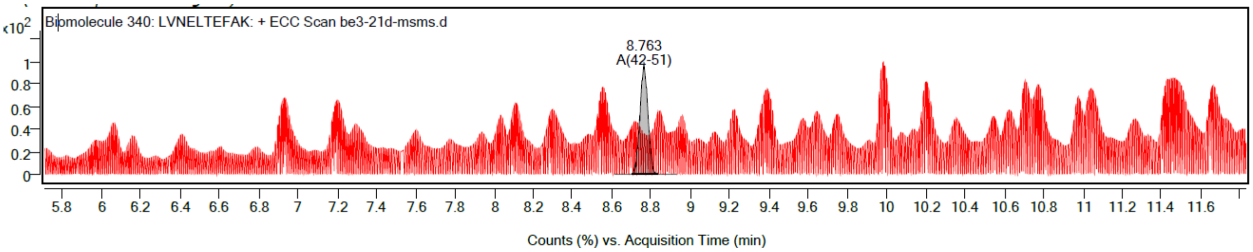

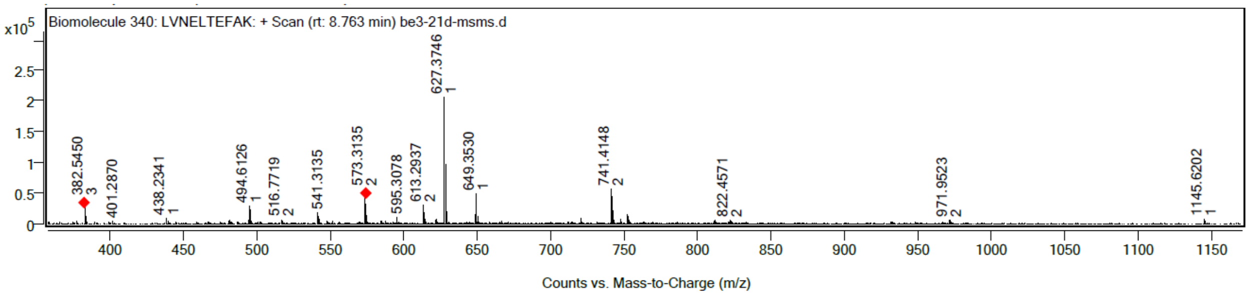

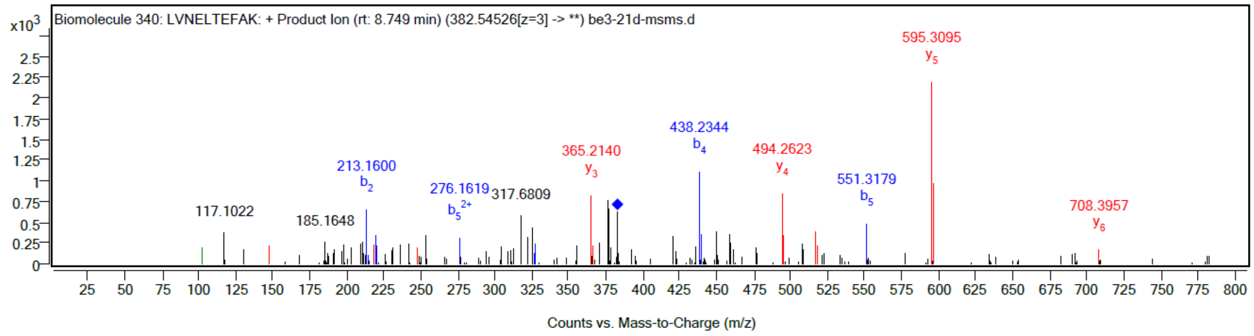

Fragment Spectrum Peaks

m/z	Diff (ppm)	Abund	Ion
147.1117	7.45	215	y1
218.1497	1.14	221	y2
365.2140	11.74	821	y3
494.2623	-2.86	840	y4
595.3095	-1.43	2191	y5
708.3957	-4.21	163	y6
247.6355	-5.79	194	y4
516.7715	0.34	380	y9
213.1600	-1.36	644	b2
438.2344	0.68	1108	b4
551.3179	1.55	476	b5
219.6207	1.56	344	b4
276.1619	4.25	306	b5
327.1875	3.10	232	b6
102.0551	-1.58	195	E
382.5444	1.36	620	Precursor
382.8765	7.42	582	Precursor

Identified peptide fragment (**1 site**): NECFLSHK (Sequence: AA 99-106, N99)

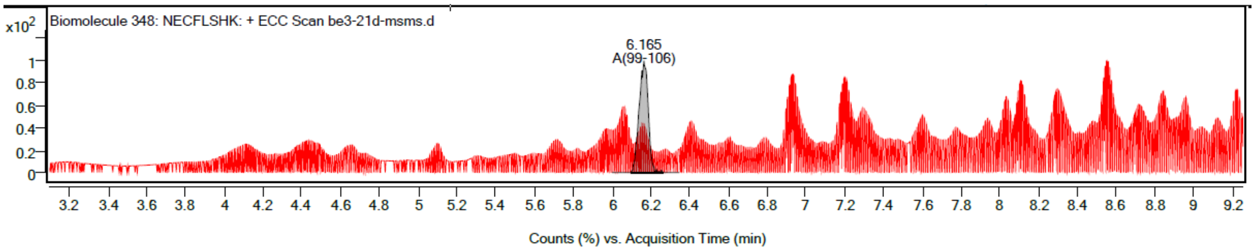

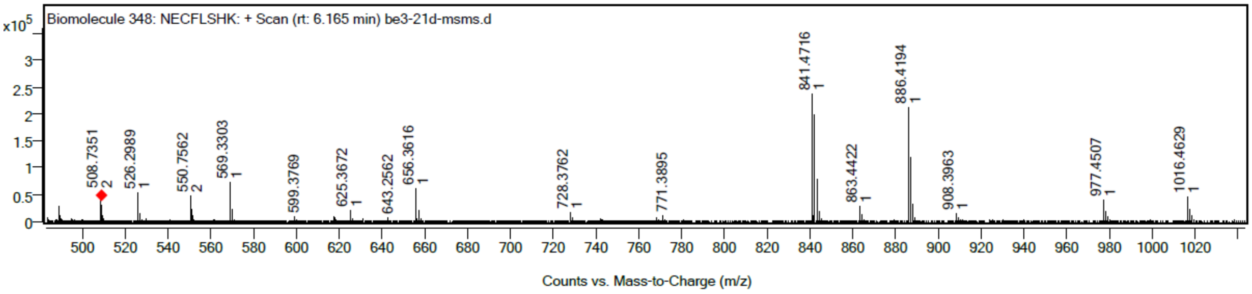

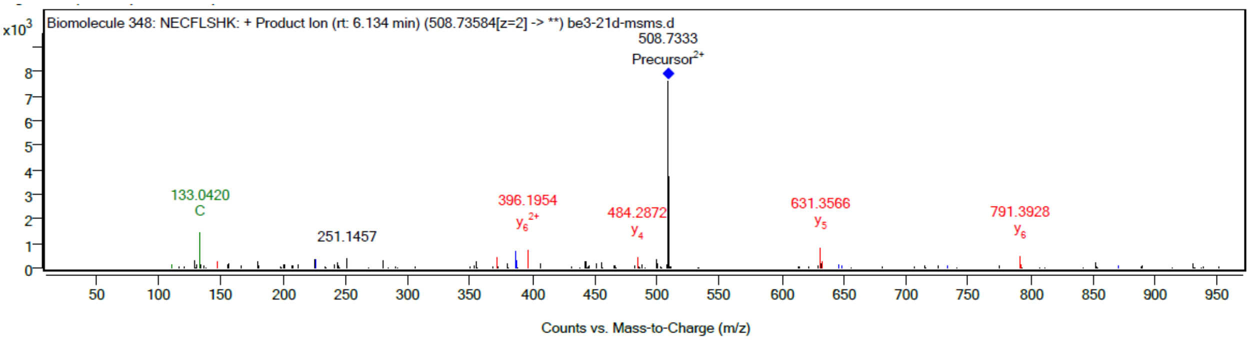

Fragment Spectrum Peaks

m/z	Diff (ppm)	Abund	Ion
147.1159	-21.17	259	y1
371.2041	-1.02	440	y3
484.2872	1.24	442	y4
631.3566	-0.60	833	y5
791.3928	-7.47	487	y6
396.1954	4.28	754	y6
226.0828	-2.39	363	b2
386.1123	1.60	697	b3
646.2657	-0.56	157	b5
733.3056	-11.13	103	b6
870.3665	-11.70	102	b7
110.0703	8.73	144	H
133.0420	7.70	1454	C
508.7333	2.55	7605	Precursor
509.2349	2.56	3745	Precursor
509.7329	9.89	672	Precursor

Identified peptide fragment (**1 site**): LKPDPNTLCDEFK (Sequence: AA 115-127, N120)

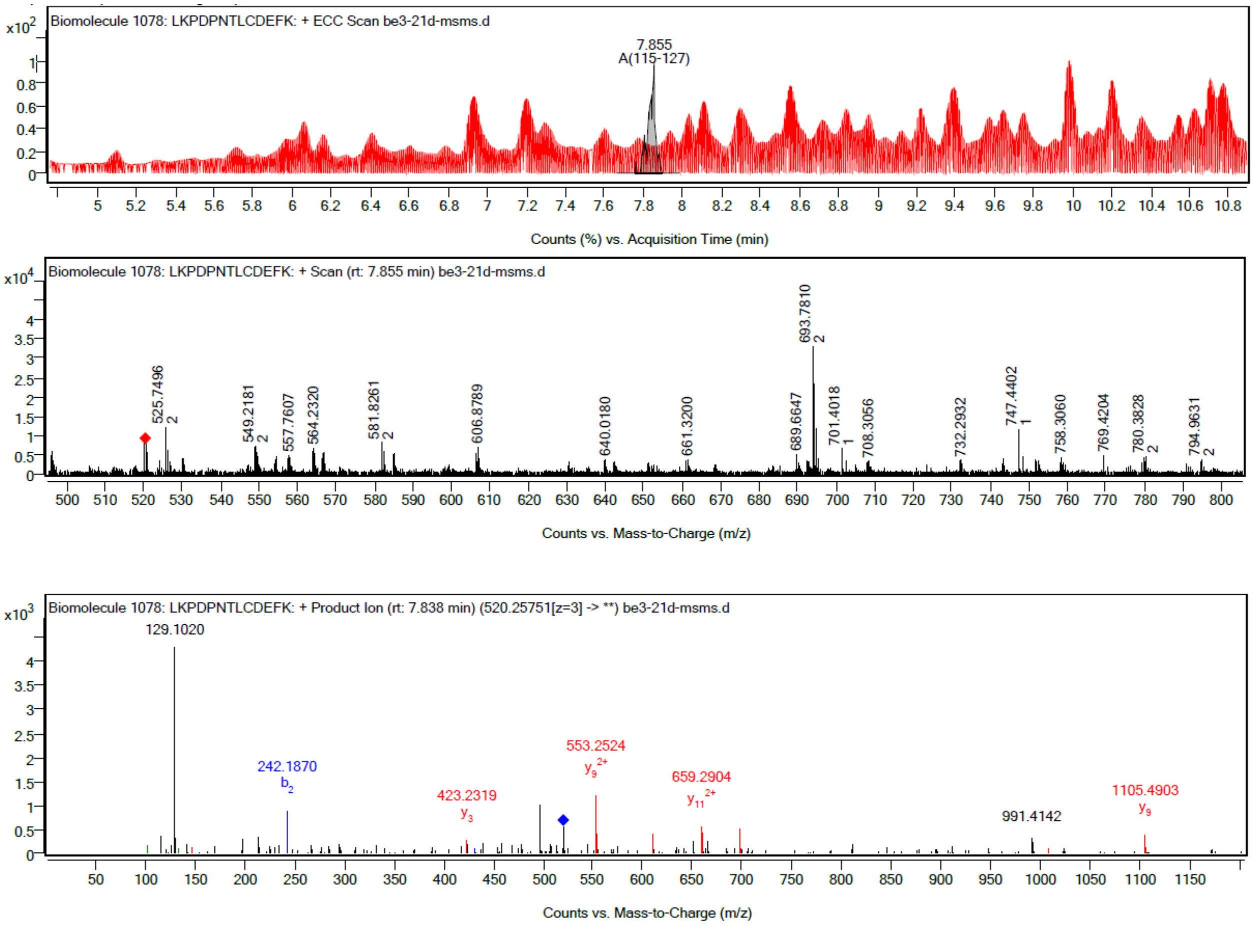

Fragment Spectrum Peaks

m/z	Diff (ppm)	Abund	Ion
147.1141	-8.74	125	y1
423.2319	-19.18	270	y3
698.2863	-6.99	508	y5
1008.4395	5.95	95	y8
1105.4903	7.23	386	y9
553.2524	0.72	1197	y9
610.7645	2.79	396	y10
659.2904	3.37	561	y11
242.1870	-2.70	867	b2
431.2462	-2.49	93	b8
102.0558	-7.79	155	E
133.0415	11.50	88	C
520.2574	-0.36	560	Precursor
520.5964	-9.19	341	Precursor
520.9270	-1.73	88	Precursor

Identified peptide fragment (**1 site**): RHPYFYAPELLYYANK (Sequence: AA 144-159, N158)

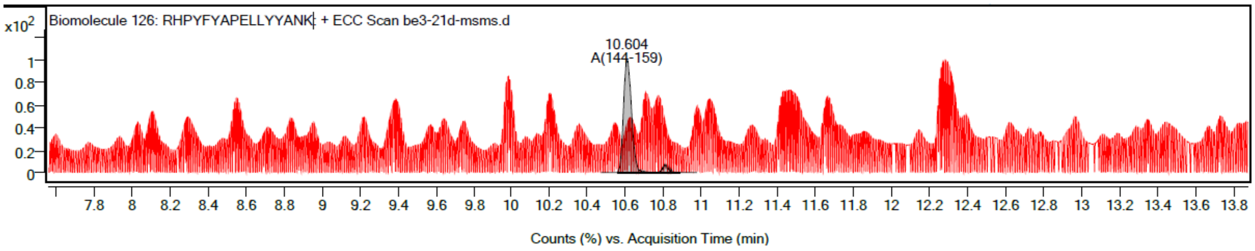

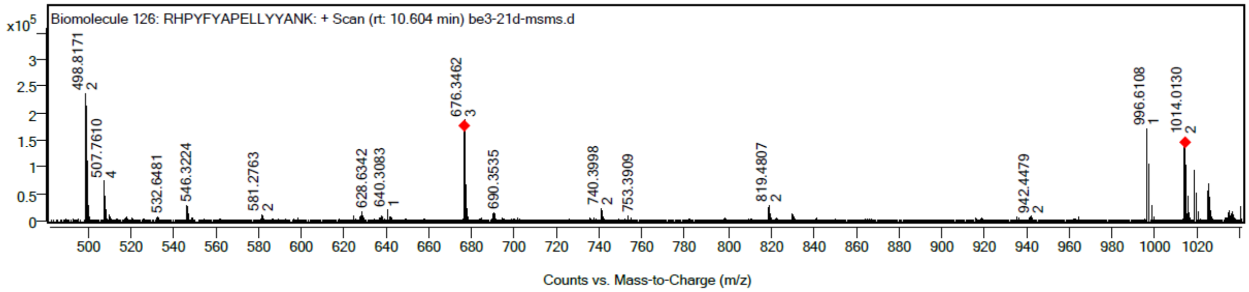

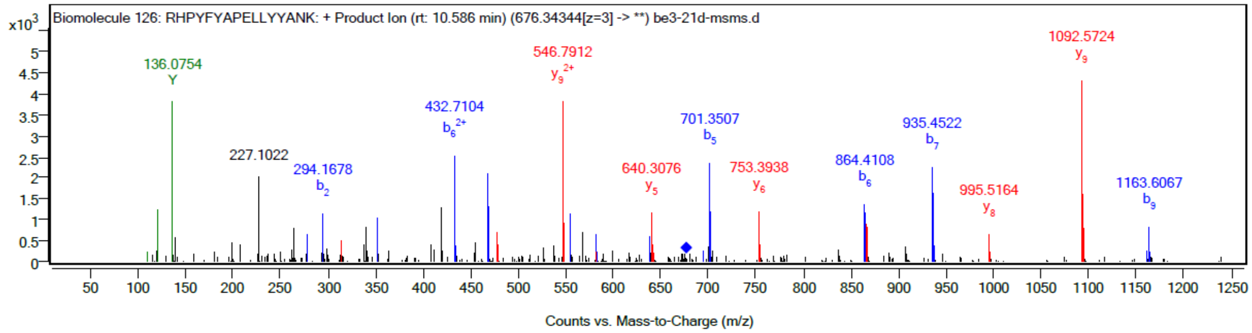

Fragment Spectrum Peaks

m/z	Diff (ppm)	Abund	Ion
936.4503	5.70	1635	y15
314.1801	7.05	508	y3
477.2446	2.11	687	y4
640.3076	2.14	1163	y5
753.3938	-1.00	1195	y6
866.4761	1.15	886	y7
995.5164	3.25	650	y8
1092.5724	0.04	4301	y9
546.7912	-2.50	3811	y9
582.3008	13.10	238	y10
294.1678	-1.70	1133	b2
554.2816	3.14	1140	b4
701.3507	1.61	2346	b5
864.4108	5.00	1360	b6
935.4522	0.09	2245	b7
1163.6067	-44.99	822	b9
277.6453	0.09	649	b4
351.1766	8.36	1051	b5
432.7104	1.87	2519	b6
468.2289	1.81	2086	b7
581.2727	8.22	647	b9
637.8209	-2.16	592	b10
694.3649	-4.85	252	b11
110.0696	15.00	232	H
120.0802	4.73	1239	F
136.0754	2.43	3818	Y

Identified peptide fragment (**1 site**): YNGVFQECCQAEDKG (Sequence: AA 160-174, Q169)

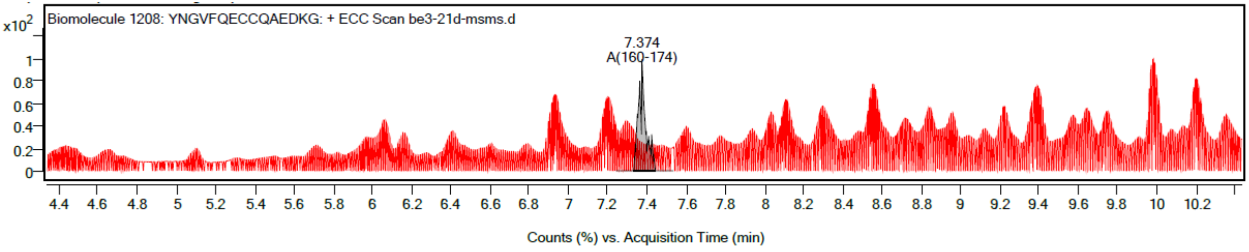

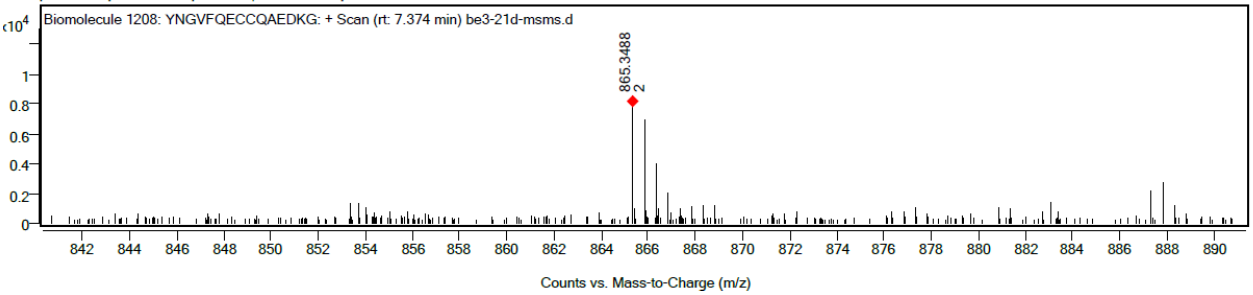

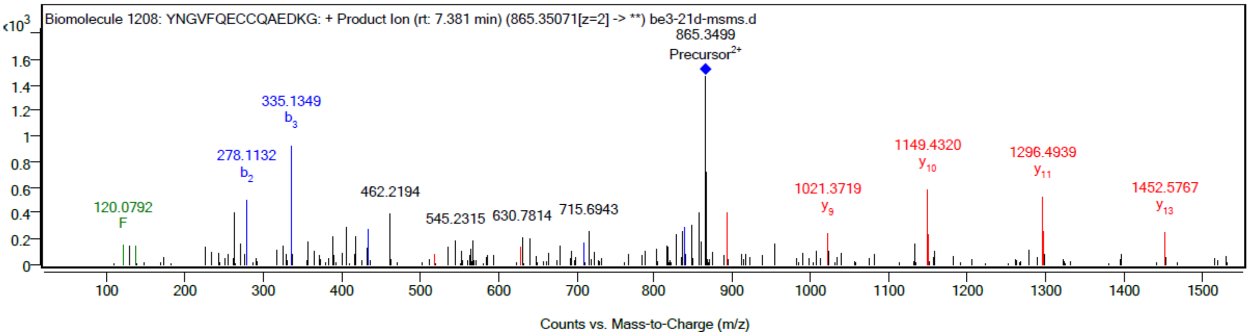

Fragment Spectrum Peaks

m/z	Diff (ppm)	Abund	Ion
629.2578	49.52	136	y6
892.3227	6.84	401	y8
1021.3719	-0.55	238	y9
1149.4320	-1.76	580	y10
1296.4939	3.42	528	y11
1452.5767	7.92	248	y13
278.1132	1.33	497	b2
335.1349	0.24	920	b3
434.2042	-1.92	276	b4
709.3391	-12.33	166	b6
838.3679	6.03	289	b7
120.0792	13.28	154	F
136.0746	8.26	144	Y
865.3499	1.17	1463	Precursor
865.8533	-0.81	721	Precursor
866.3545	-0.28	223	Precursor

Identified peptide fragment (**1 site**): YICDNQDTISSK (Sequence: AA 262-273, Q267)

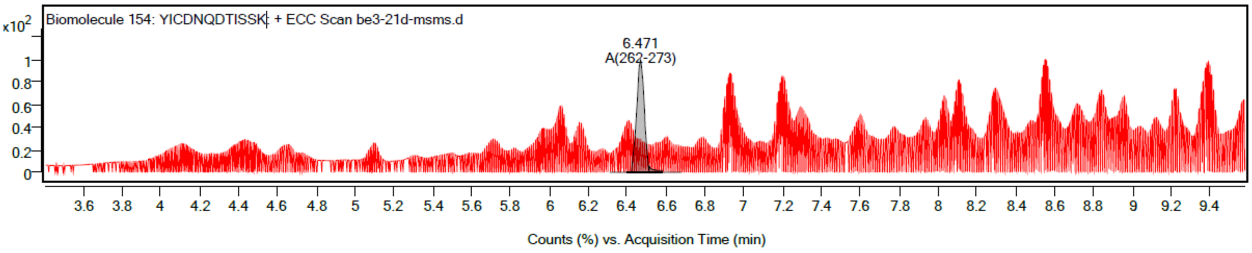

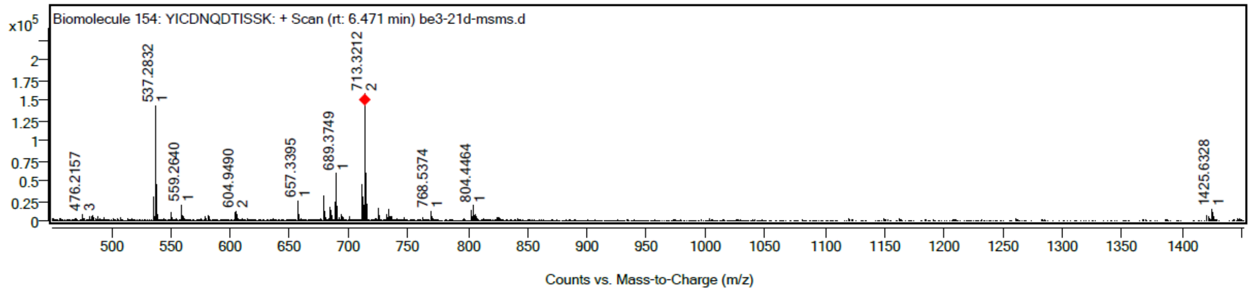

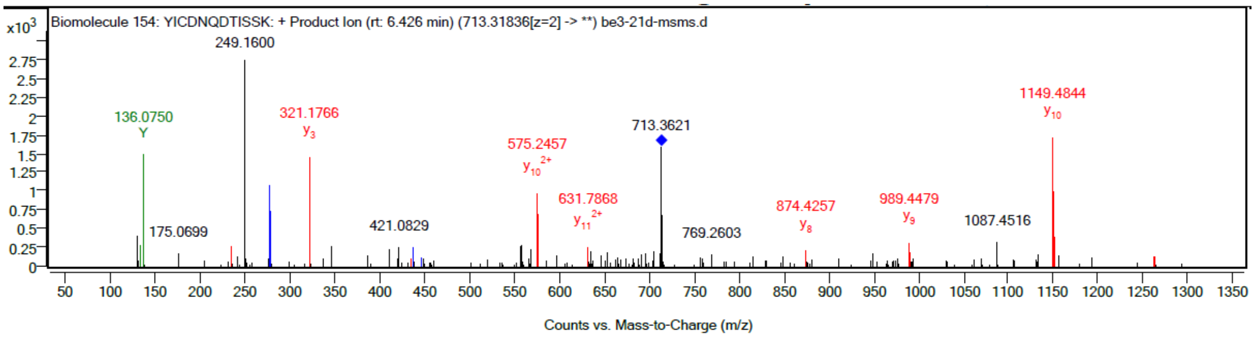

Fragment Spectrum Peaks

m/z	Diff (ppm)	Abund	Ion
234.1450	-0.75	256	y2
321.1766	0.88	1446	y3
434.2597	2.89	90	y4
874.4257	0.90	195	y8
989.4479	5.56	302	y9
1149.4844	-0.27	1706	y10
1262.5674	0.60	118	y11
575.2457	-0.07	959	y10
631.7868	1.43	237	y11
277.1546	0.43	1069	b2
437.1862	-2.05	245	b3
446.1854	-37.48	107	b7
133.0436	-4.19	271	C
136.0750	4.97	1483	Y
713.3209	-2.09	1278	Precursor
713.8228	-2.39	674	Precursor
714.3194	4.70	482	Precursor

Identified peptide fragment (**1 site**): DAIPENLPPLTADFAEDK (Sequence: AA 295-312, N300)

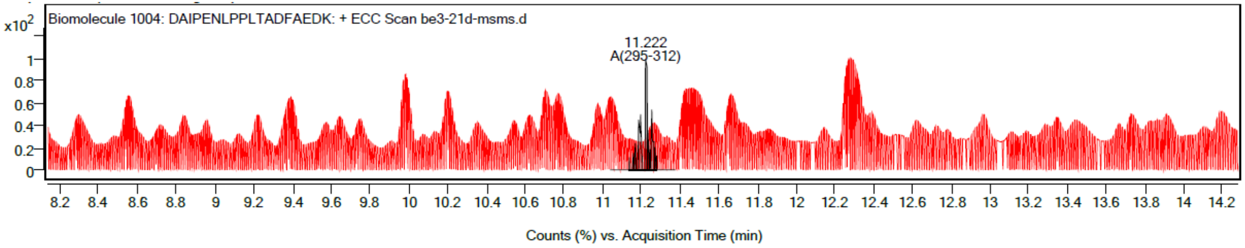

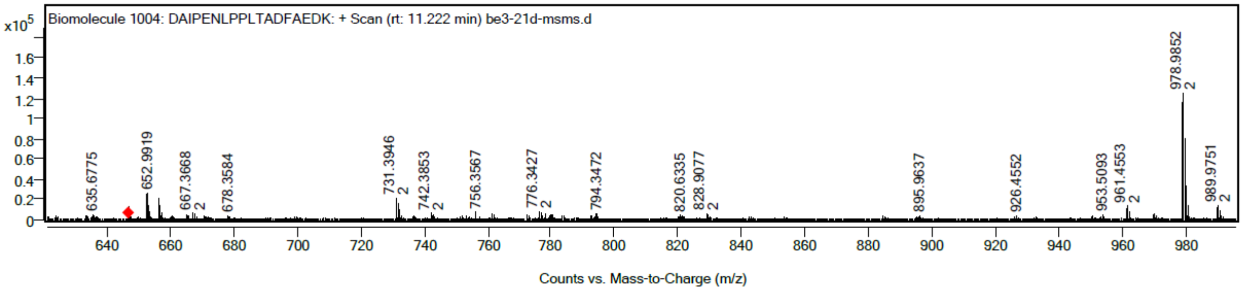

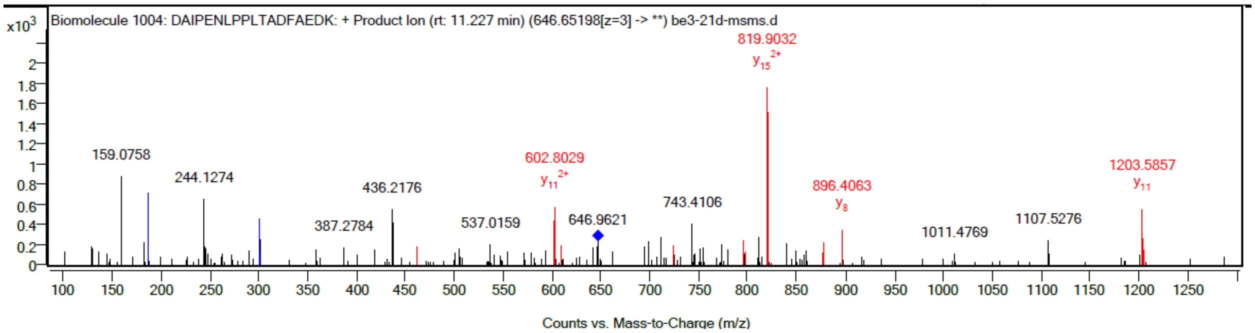

Fragment Spectrum Peaks

m/z	Diff (ppm)	Abund	Ion
462.2141	11.53	167	y4
609.2946	-10.99	178	y5
724.3110	5.26	181	y6
795.3487	4.11	227	y7
896.4063	-7.49	338	y8
1203.5857	2.89	546	y11
602.8029	-5.02	567	y11
819.9032	1.11	1755	y15
877.4531	-4.08	217	y16
187.0701	6.68	712	b2
300.1549	1.65	445	b3

Identified peptide fragment (**2 sites**): NYQEAKDAFLGSFLYEYSR (Sequence: AA 317-335, [N317, Q319])

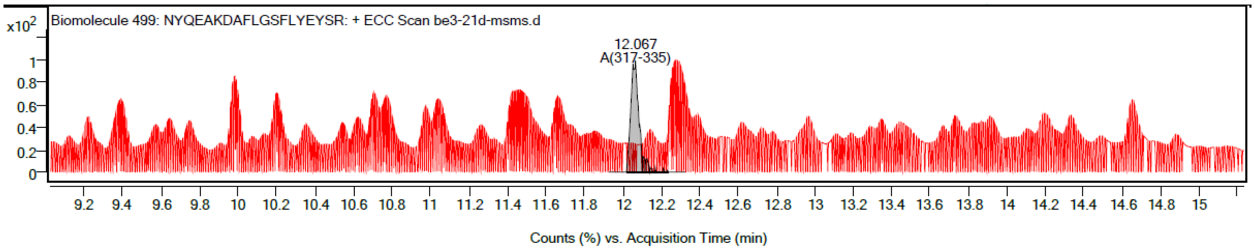

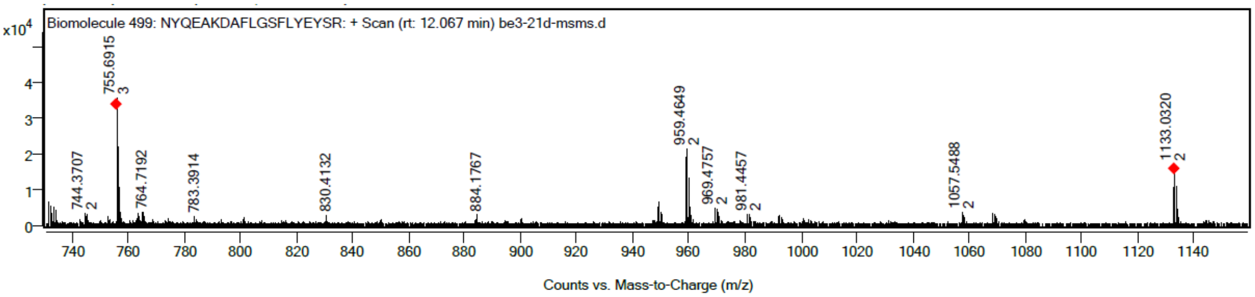

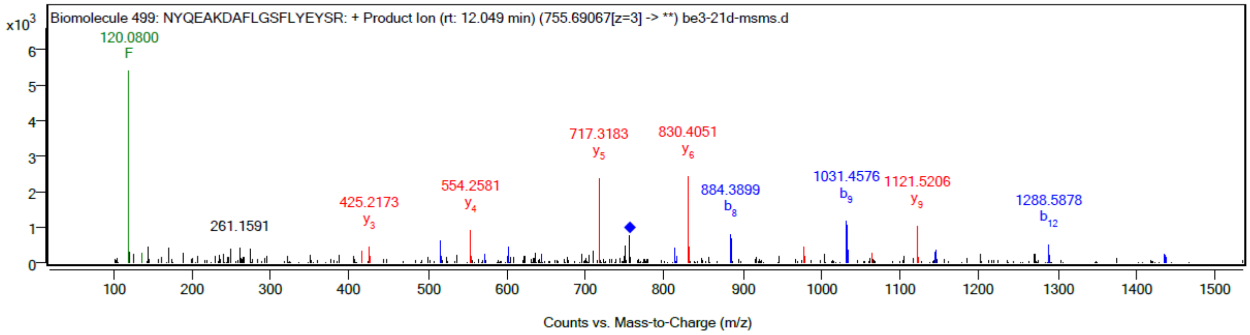

Fragment Spectrum Peaks

m/z	Diff (ppm)	Abund	Ion
425.2173	-6.92	437	y3
554.2581	-2.24	917	y4
717.3183	2.66	2379	y5
830.4051	-0.96	2425	y6
977.4790	-6.42	439	y7
1064.5072	-2.30	278	y8
1121.5206	4.95	1033	y9
415.7022	8.72	317	y6
813.3496	3.64	411	b7
884.3899	-0.19	796	b8
1031.4576	0.53	1164	b9
1145.5546	-7.88	365	b10
1288.5878	6.14	498	b12
718.3207	3.98	1346	b13
1435.6657	-1.15	254	b13
516.2333	-1.25	615	b9
572.7754	-1.12	249	b10
601.7893	-3.56	440	b11
644.8050	-5.43	250	b12
120.0800	6.37	5404	F
136.0752	3.31	269	Y

Identified peptide fragment (**1 site**): HLVDEPQNLIK (Sequence: AA 378-388, N385)

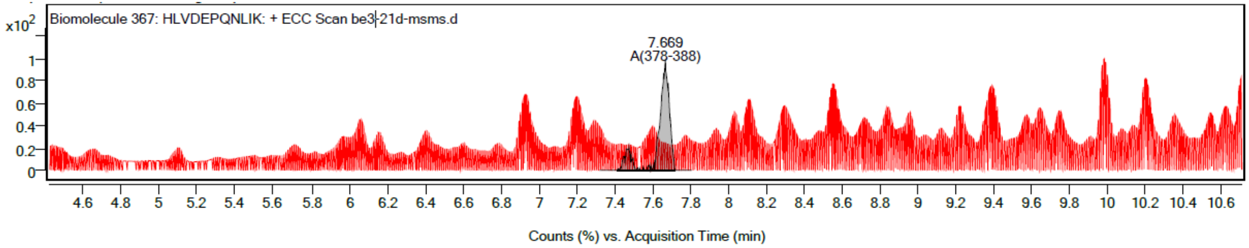

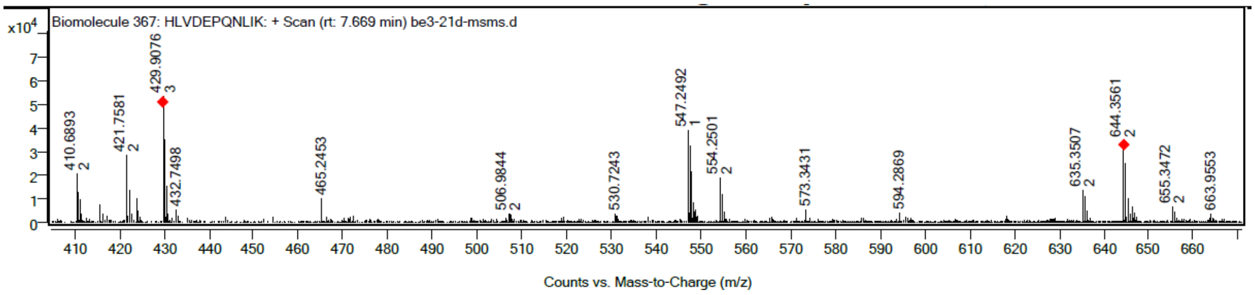

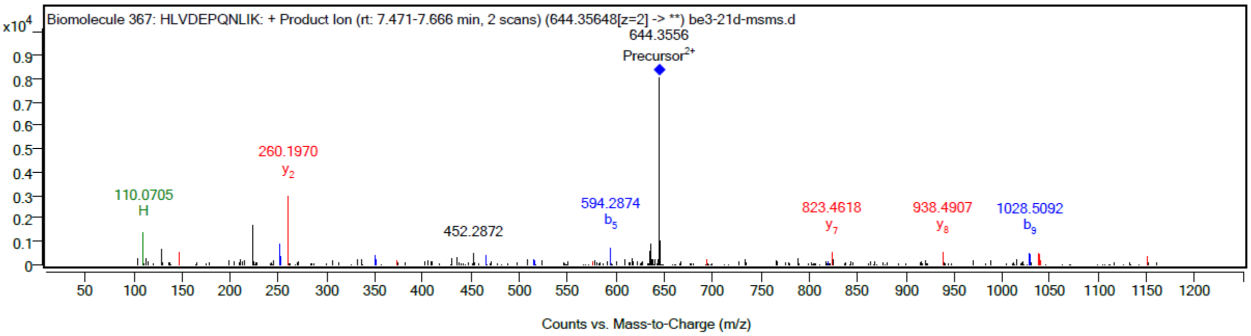

Fragment Spectrum Peaks

m/z	Diff (ppm)	Abund	Ion
147.1122	4.33	549	y1
260.1970	-0.47	2928	y2
373.2777	8.61	195	y3
694.4181	9.34	218	y6
823.4618	6.64	523	y7
938.4907	3.72	559	y8
1037.5595	2.93	511	y9
1150.6455	1.04	352	y10
575.8207	10.89	151	y10
251.1491	4.56	909	b2
350.2173	3.89	383	b3
465.2460	-0.91	396	b4
594.2874	1.36	730	b5
819.4380	-46.96	158	b7
1028.5092	6.60	516	b9
514.7608	1.62	212	b9
110.0705	6.89	1374	H
644.3556	1.22	8050	Precursor
644.8549	4.95	5485	Precursor
645.3612	-2.20	1049	Precursor

Identified peptide fragment (**2 sites**): LGEYGFQNALIVR (Sequence: AA 397-409, [Q403, N404])

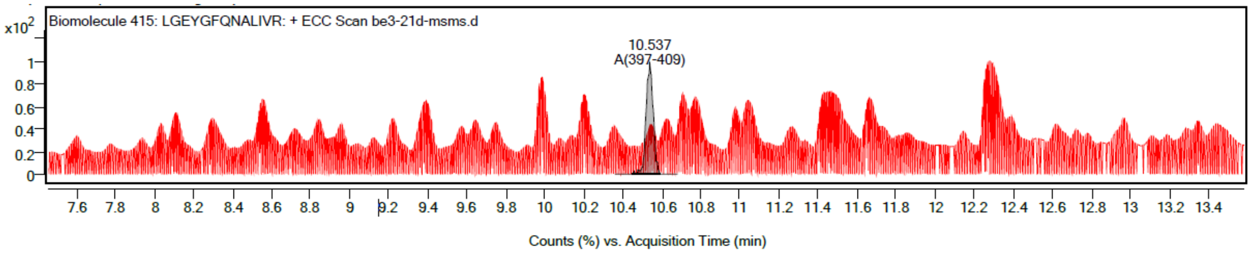

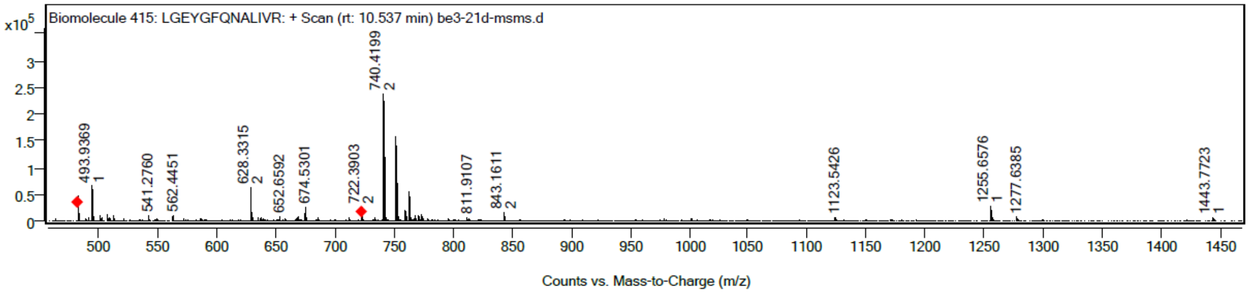

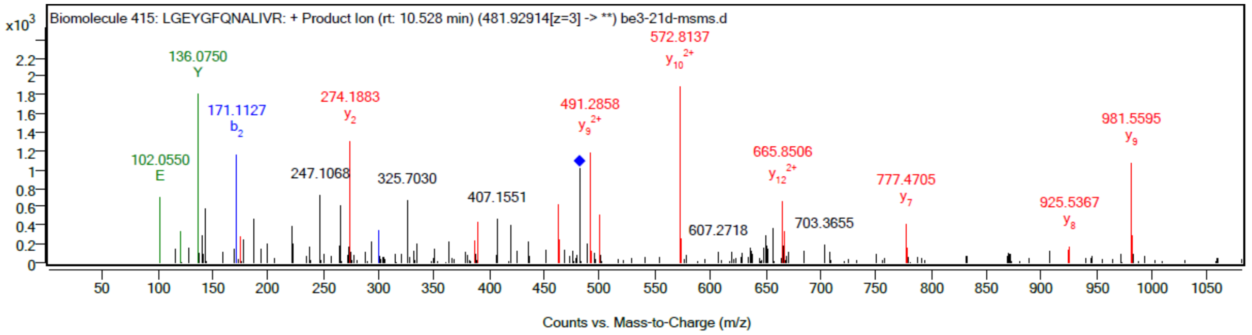

Fragment Spectrum Peaks

m/z	Diff (ppm)	Abund	Ion
175.1153	20.58	282	y1
274.1883	-3.28	1301	y2
387.2731	-4.22	232	y3
500.3554	0.17	513	y4
667.4310	-9.03	339	y6
777.4705	3.22	412	y7
925.5367	8.68	168	y8
981.5595	3.45	1065	y9
389.2406	-1.12	429	y7
462.7782	-8.37	627	y8
491.2858	-1.51	1176	y9
572.8137	5.35	1890	y10
665.8506	-2.75	657	y12
171.1127	0.67	1155	b2
300.1543	3.69	343	b3
102.0550	-0.70	703	E
120.0794	11.46	334	F
136.0750	4.73	1813	Y
481.9306	-1.93	398	Precursor
482.2656	-3.13	1017	Precursor
482.5964	4.43	181	Precursor

Identified peptide fragment (**1 site**): MPCTEDYLSLILNR (Sequence: AA 445-458, N457)

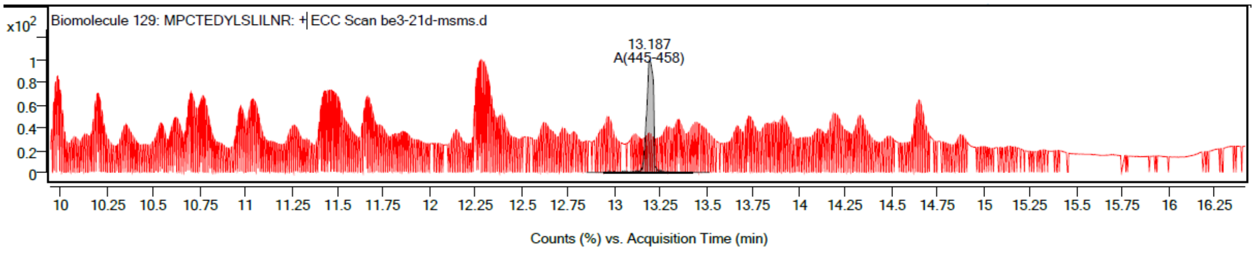

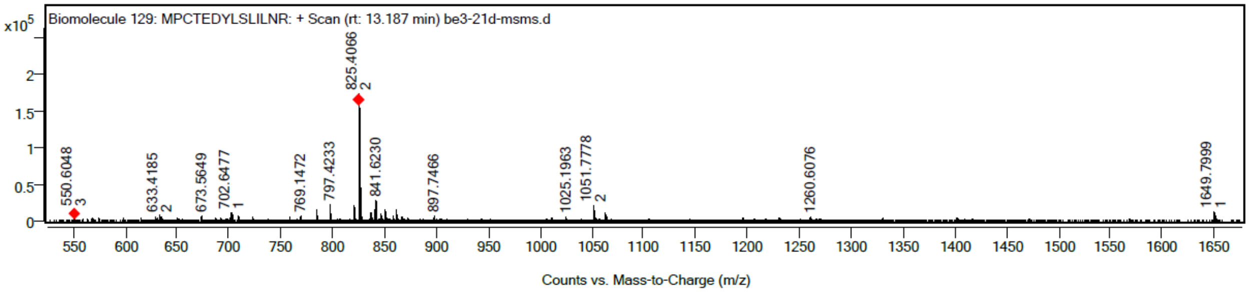

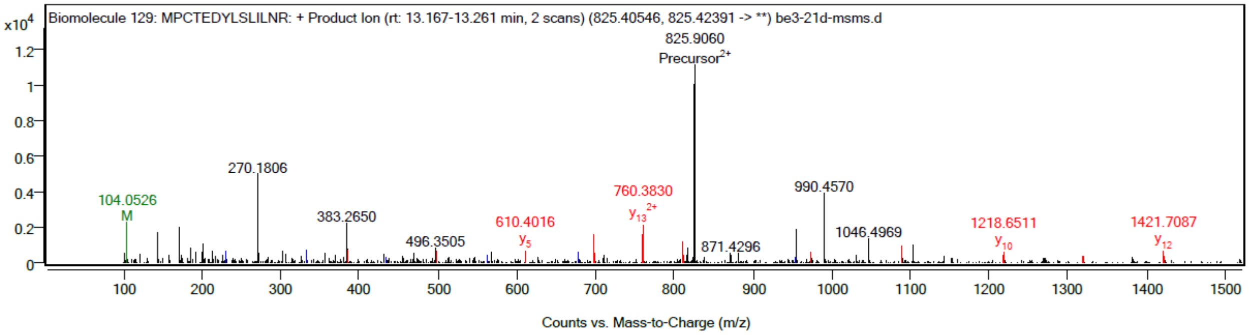

Fragment Spectrum Peaks

m/z	Diff (ppm)	Abund	Ion
384.2358	-1.15	698	y3
497.3179	3.17	667	y4
610.4016	3.17	689	y5
697.4301	7.85	1599	y6
810.5216	-2.49	1196	y7
973.5785	4.50	614	y8
1088.6106	-0.63	931	y9
1218.6511	3.91	599	y10
1318.6936	4.95	400	y11
1421.7087	0.46	670	y12
760.3830	4.36	2135	y13
229.1005	0.17	644	b2
332.1086	3.32	739	b3
433.1554	4.66	304	b4
562.1986	2.50	430	b5
677.2246	3.50	601	b6
953.3731	1.31	337	b8
104.0526	2.33	2309	M
825.4043	0.72	10011	Precursor
825.9060	0.70	11115	Precursor
826.4006	9.31	2696	Precursor

Identified peptide fragment (**1 site**): QTALVELLK (Sequence: AA 525-533, Q525)

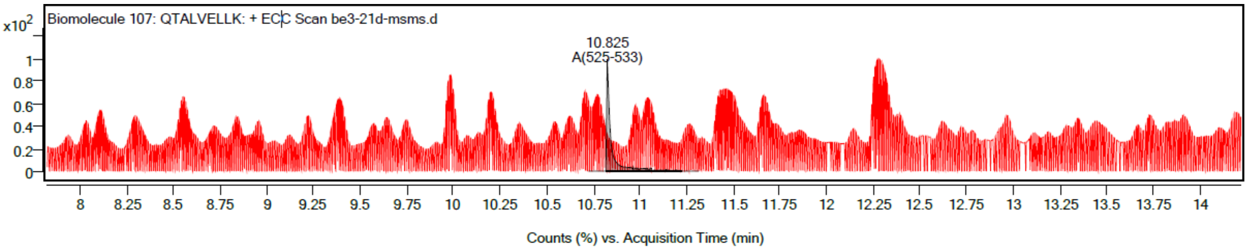

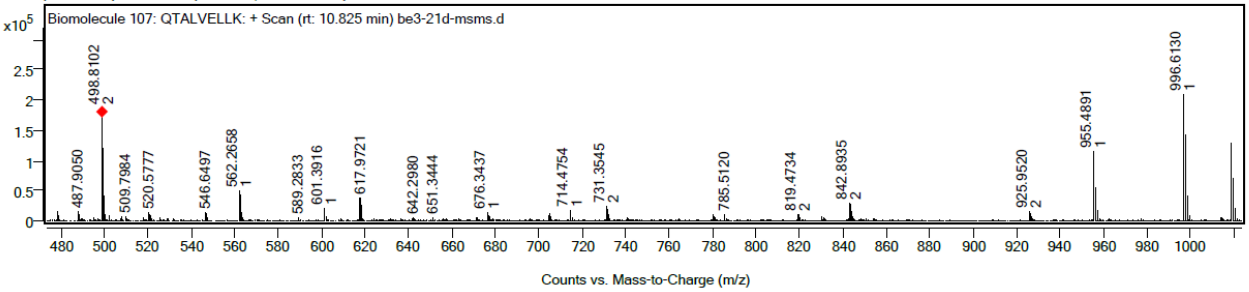

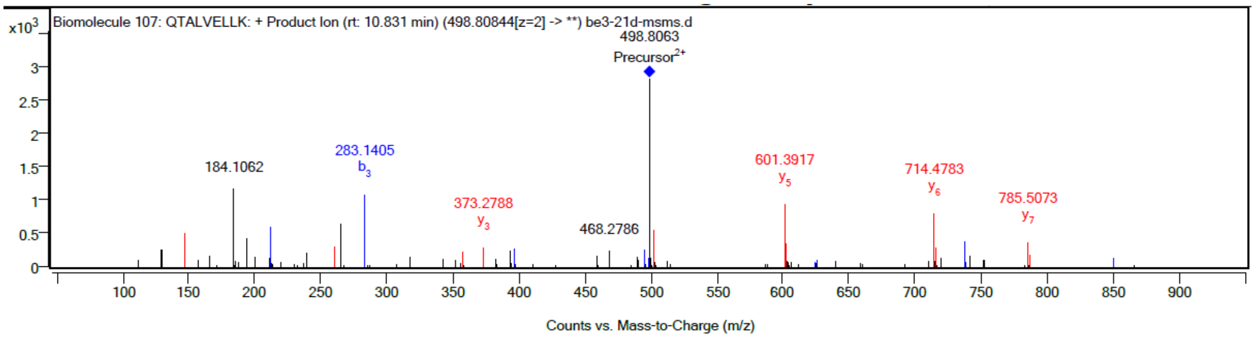

Fragment Spectrum Peaks

m/z	Diff (ppm)	Abund	Ion
147.1118	7.13	497	y1
260.1969	0.04	294	y2
373.2788	5.81	272	y3
502.3204	6.25	543	y4
601.3917	0.38	922	y5
714.4783	-3.27	778	y6
785.5073	7.35	357	y7
357.7425	-2.52	206	y6
212.1004	11.90	577	b2
283.1405	-1.38	1064	b3
396.2248	-1.55	264	b4
495.2914	2.26	239	b5
625.3344	6.58	83	b6
737.4127	8.89	371	b7
850.5016	1.98	116	b8
498.8063	3.51	2798	Precursor
499.3095	0.40	1162	Precursor
499.8078	7.26	122	Precursor

Identified peptide fragment (**1 site**): TVMENFVAFVDK (Sequence: AA 545-556, N549)

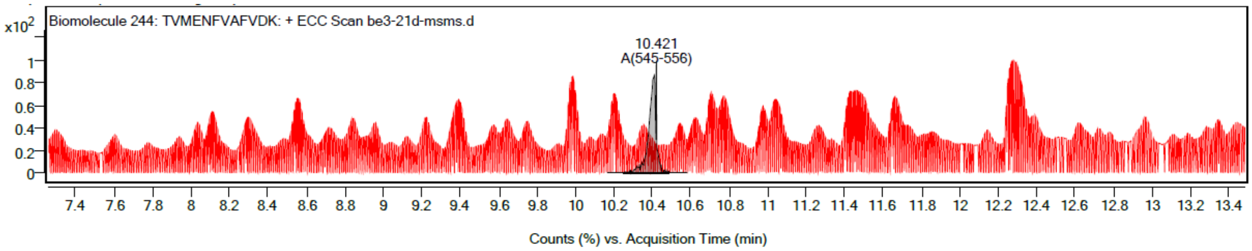

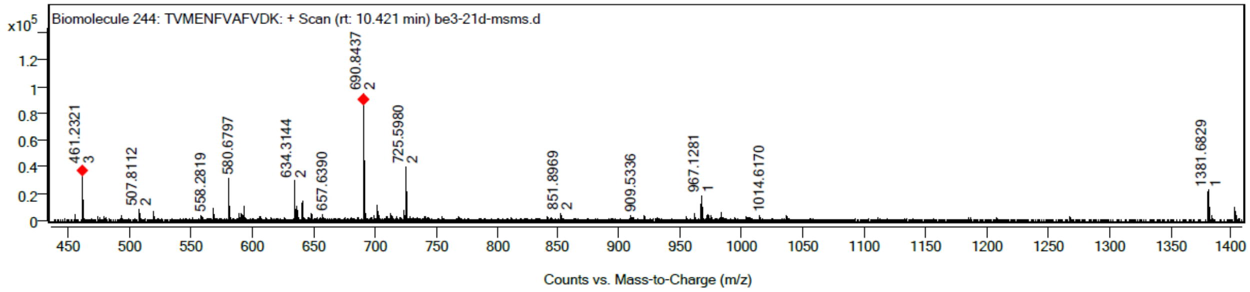

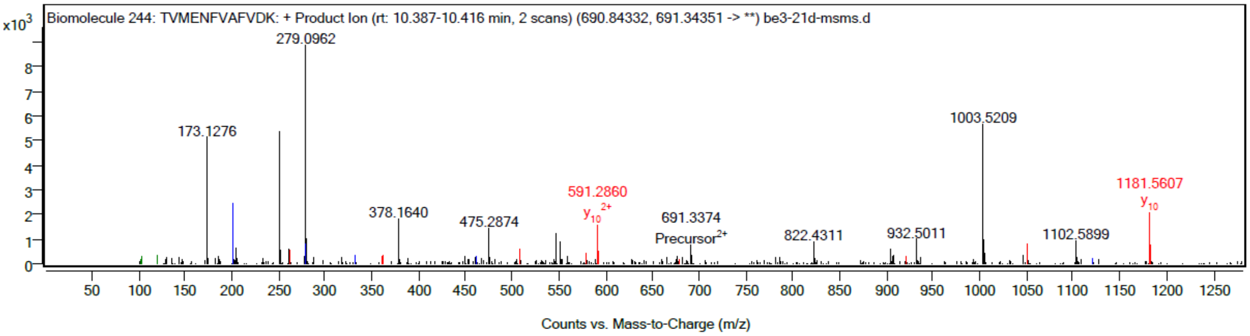

Fragment Spectrum Peaks

m/z	Diff (ppm)	Abund	Ion
262.1399	-0.70	570	y2
362.2101	3.99	367	y3
508.2730	7.12	589	y4
579.3157	-3.43	418	y5
678.3776	6.63	195	y6
921.4821	0.86	290	y8
1050.5278	-2.20	821	y9
1181.5607	4.43	2089	y10
591.2860	1.05	1574	y10
201.1237	-1.89	2472	b2
332.1593	13.66	342	b3
461.2061	0.79	314	b4
1120.5571	-6.69	223	b10
279.1286	-19.83	799	b5
102.0536	12.85	187	E
104.0527	1.01	289	M
120.0817	-7.58	362	F
691.3374	10.55	786	Precursor
691.8521	-8.31	337	Precursor

Identified peptide fragment (**1 site**): LVVSTQTAL (Sequence: AA 574-582, Q579)

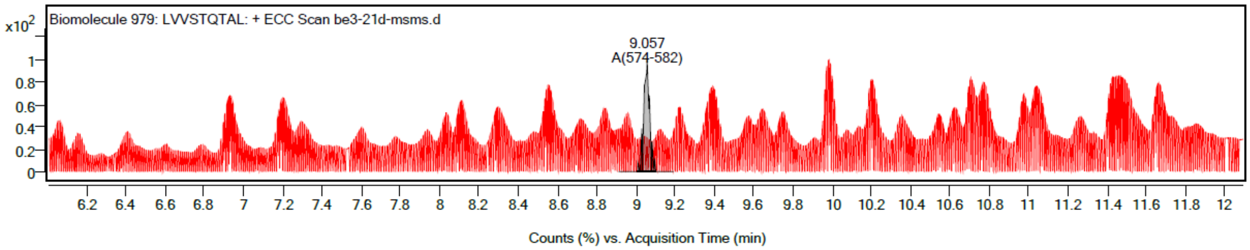

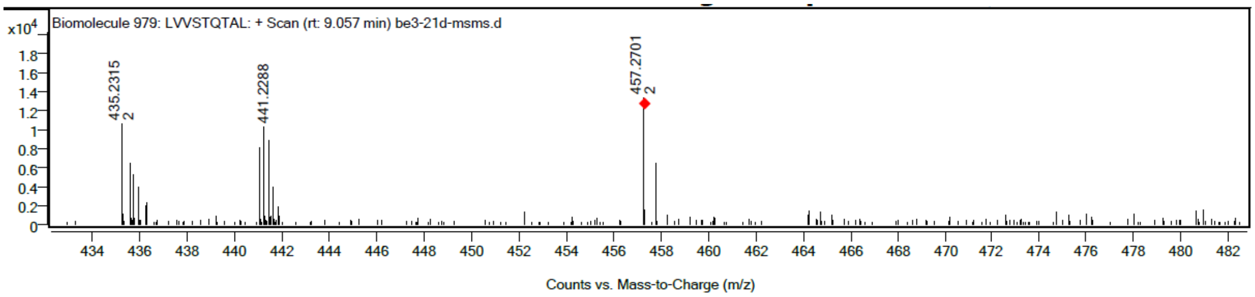

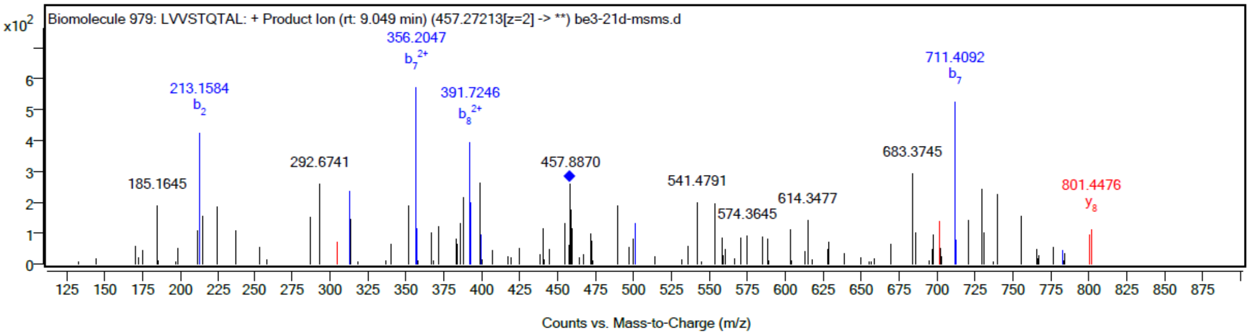

Fragment Spectrum Peaks

m/z	Diff (ppm)	Abund	Ion
304.1784	27.43	70	y3
701.3691	19.55	137	y7
801.4476	8.74	110	y8
213.1584	6.32	421	b2
312.2241	12.96	235	b3
399.2576	6.59	94	b4
500.3098	-3.91	131	b5
711.4092	-7.95	522	b7
782.4538	-16.71	43	b8
356.2047	2.01	569	b7
391.7246	-1.57	391	b8

Supplementary Fig. 19b: Analysis of Nitrile-Modified Sites

We have analyzed all modified sites across the individual proteins modified in figure 8 via Solvent Accessible Surface Area (SASA) analysis via the LCPO method.[4] Given the standard threshold cutoff of 0.2 relative solvent-accessible surface area (RSA), 66.3% of Asn-nitriles were solvent exposed compared to 73.6% of all Asn residues in those proteins. For Gln it was 63.0% for nitrile labelled and 67.6% for all Gln.

Asparagine All SASA Overview

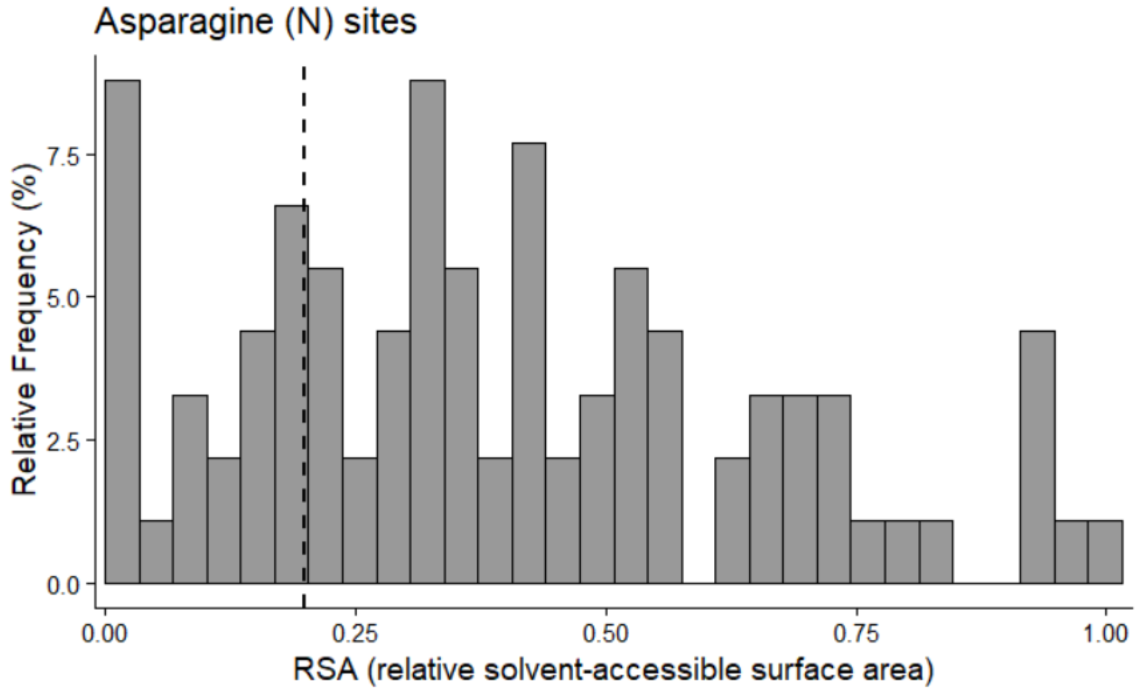


```
========================
Residue: Asparagine (N)
N sites: 91
            exposure  n  percent
1  Buried (<20% RSA) 24 26.37363
2 Exposed (≥20% RSA) 67 73.62637
      25%       50%       75%
0.1923418 0.3550633 0.5512342
lower.25% upper.75%
0.1923418 0.5512342
Percent exposed (RSA >= 0.20): 73.6 %
                    Statistic RSA_value
25%      25th percentile (Q1)     0.192
50%               Median (Q2)     0.355
75%      75th percentile (Q3)     0.551
    Interquartile range (IQR) 0.19–0.55
            Exposure  N Frequency (%)
  Buried (RSA < 20%) 24          26.4
 Exposed (RSA ≥ 20%) 67          73.6
```

Glutamine All SASA Overview

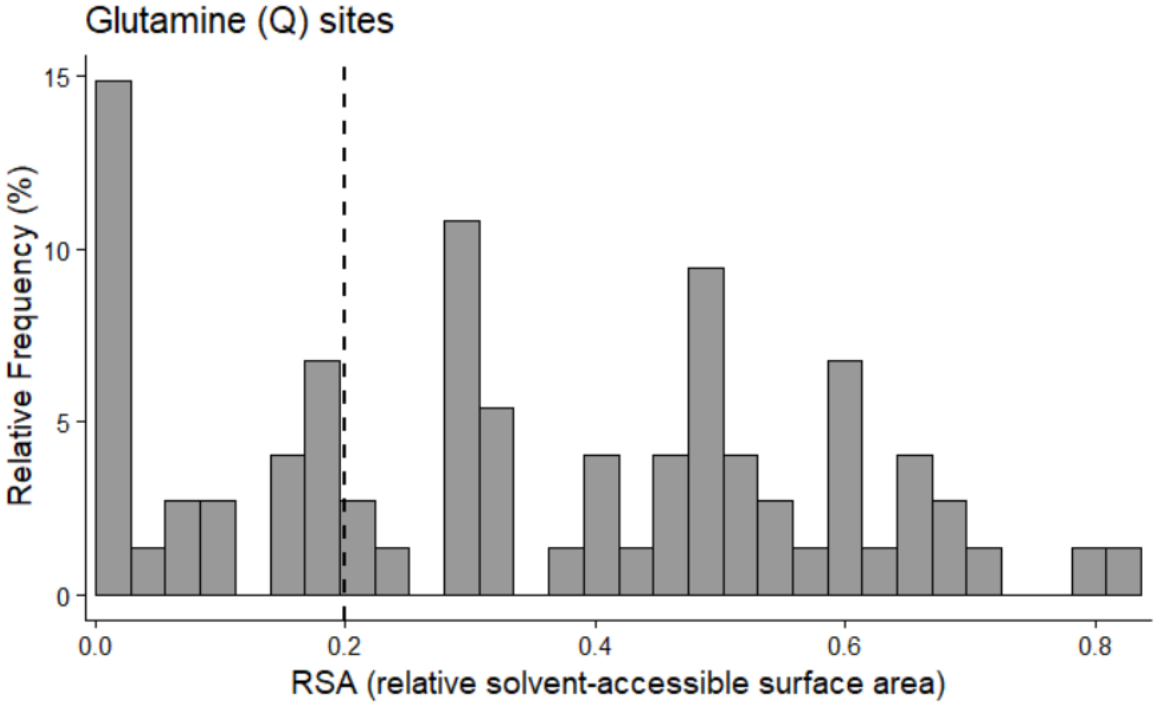


```
========================
Residue: Glutamine (Q)
N sites: 74
            exposure  n  percent
1  Buried (<20% RSA) 24 32.43243
2 Exposed (≥20% RSA) 50 67.56757
      25%       50%       75%
0.1604762 0.3219841 0.5153042
lower.25% upper.75%
0.1604762 0.5153042
Percent exposed (RSA >= 0.20): 67.6 %
                      Statistic RSA_value
25%      25th percentile (Q1)      0.16
50%              Median (Q2)     0.322
75%      75th percentile (Q3)     0.515
    Interquartile range (IQR) 0.16-0.52
            Exposure  N Frequency (%)
  Buried (RSA < 20%) 24          32.4
 Exposed (RSA ≥ 20%) 50          67.6
```

Asparagine Nitrile SASA Overview

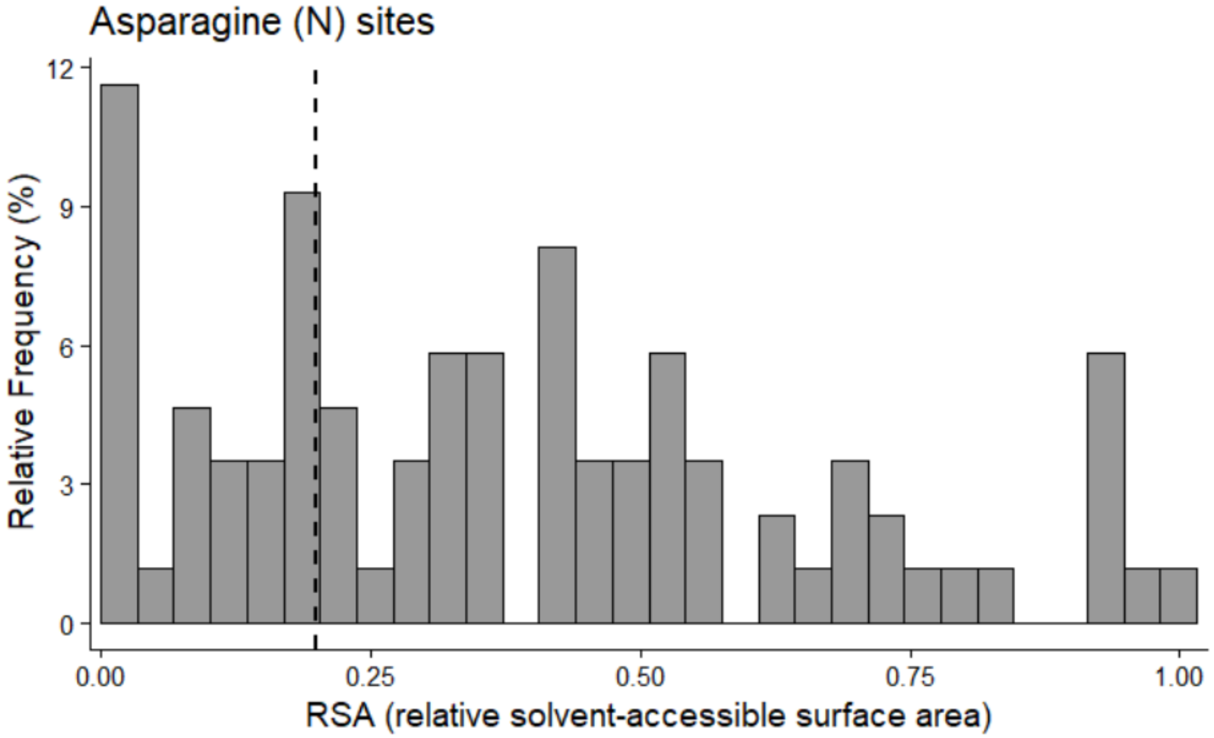


```
========================
Residue: Asparagine (N)
N sites: 86
            exposure  n  percent
1  Buried (<20% RSA) 29 33.72093
2 Exposed (≥20% RSA) 57 66.27907
      25%       50%       75%
0.1747468 0.3396519 0.5387500
lower.25% upper.75%
0.1747468 0.5387500
Percent exposed (RSA >= 0.20): 66.3 %
                    Statistic RSA_value
25%      25th percentile (Q1)     0.175
50%              Median (Q2)      0.34
75%      75th percentile (Q3)     0.539
    Interquartile range (IQR) 0.17–0.54
            Exposure  N Frequency (%)
  Buried (RSA < 20%) 29          33.7
 Exposed (RSA ≥ 20%) 57          66.3
```

Glutamine Nitrile SASA Overview

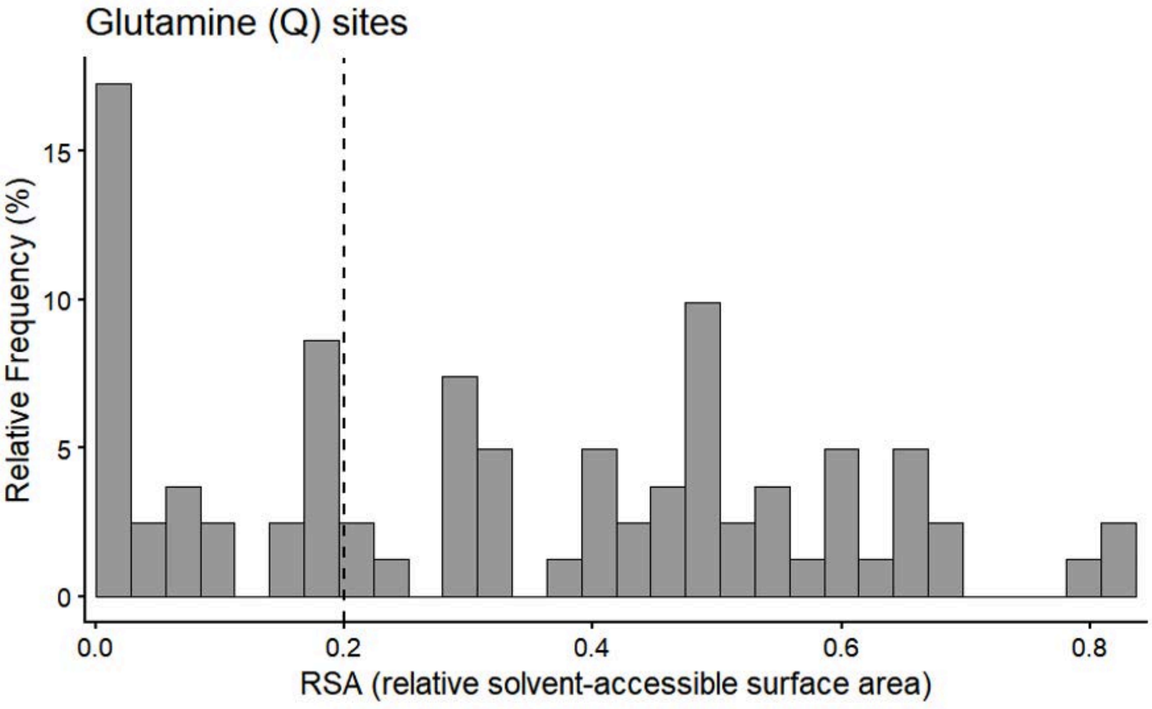


```
========================
Residue: Glutamine (Q)
N sites: 81
            exposure  n  percent
1  Buried (<20% RSA) 30 37.03704
2 Exposed (≥20% RSA) 51 62.96296
      25%       50%       75%
0.1070899 0.3167725 0.4957143
lower.25% upper.75%
0.1070899 0.4957143
Percent exposed (RSA >= 0.20): 63 %
                          Statistic RSA_value
25%      25th percentile (Q1)     0.107
50%               Median (Q2)     0.317
75%      75th percentile (Q3)     0.496
    Interquartile range (IQR)  0.11-0.5
            Exposure  N Frequency (%)
  Buried (RSA < 20%) 30            37
 Exposed (RSA ≥ 20%) 51            63
```

Supplementary Fig. 20: Modification of Ubiquitin Nitrile to Ketone Using Boronic Acid

We next sought to apply the boronic acid carbometallation reaction to nitrile-modified protein, generating orthogonal ketone handles at Asn/Gln sites. We began our studies using nitrile ubiquitin (modified with 10 mM $Pd(OAc)_2$ to generate a high number of nitrile sites) and highly reactive electron-donating 4-methoxyphenylboronic acid. We were able to obtain quantitative modification of ubiquitin nitrile to ketone, establishing conditions for further protein studies.

Conversion of Ubiquitin to Nitrile (10 mM PdOAc)2

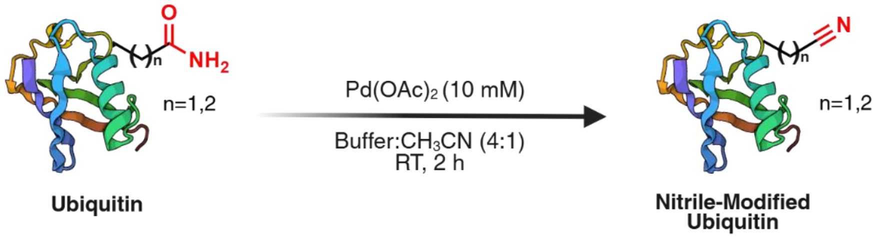

Ubiquitin (2 mg, 0.234 mM) was dissolved in 800 µL of NaP buffer (10 mM, pH 7.4) and 200 µL of $Pd(OAc)_2$ (2.24 mg, 10 mM) dissolved in ACN was added. The reaction was stirred at room temperature for 2 h followed by quenching with 500 µL of 1 M of aqueous L-cysteine solution and 10 µL of 1 M NaOH solution. The crude reaction mixture was passed through an Amicon™ Ultra 3 kDa centrifugal filter and washed with H_2O (7×0.5 mL) to remove the water-soluble Pd complex. The labeled protein was redissolved in 0.1% formic acid in H_2O and analyzed using LC-MS, revealing >95% conversion with 1-5 nitrile modifications observed (see table below).

Modification	Mass	Conversion
Unmodified	8565.1	n.d.
1 Nitrile	8547.7 (**-18**)	17%
2 Nitrile	8529.3 (**-18**)	29%
3 Nitrile	8511.1 (**-18**)	28%
4 Nitrile	8493.0 (**-18**)	18%
5 Nitrile	8474.9 (**-18**)	8%

MS of Nitrile-Modified Ubiquitin

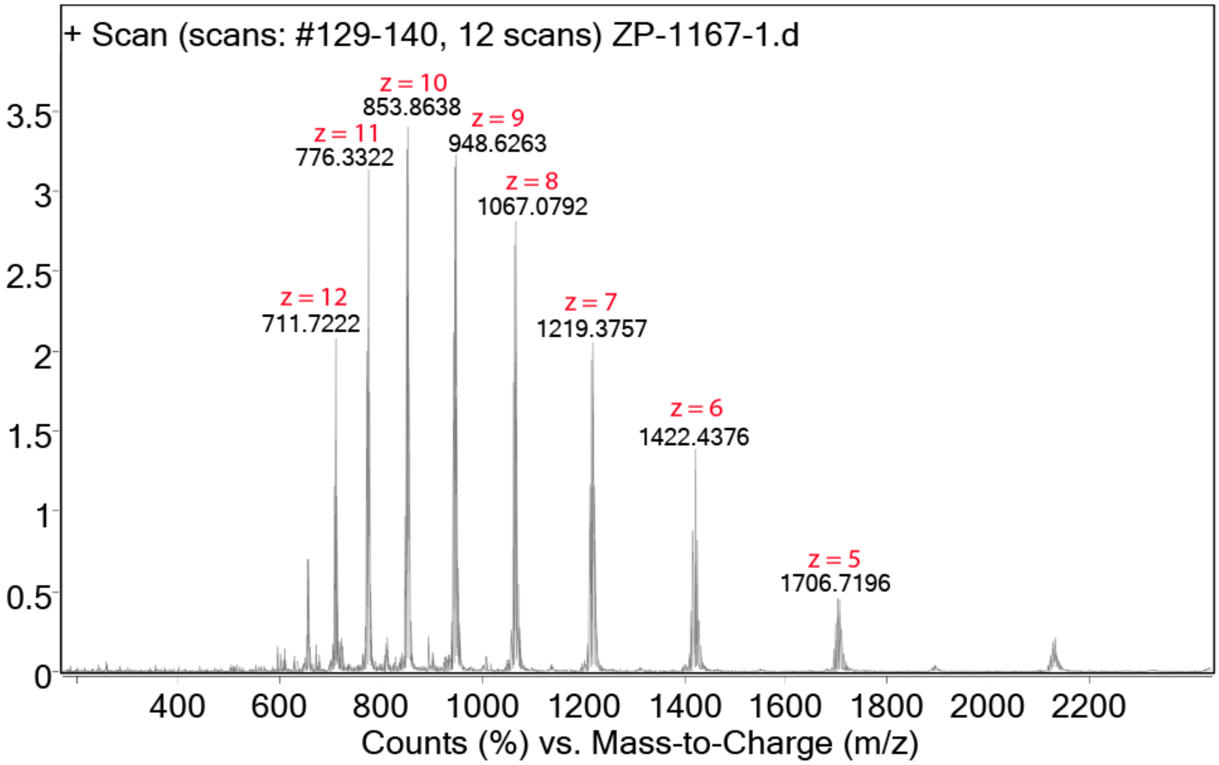

Deconvoluted MS of Nitrile-Modified Ubiquitin

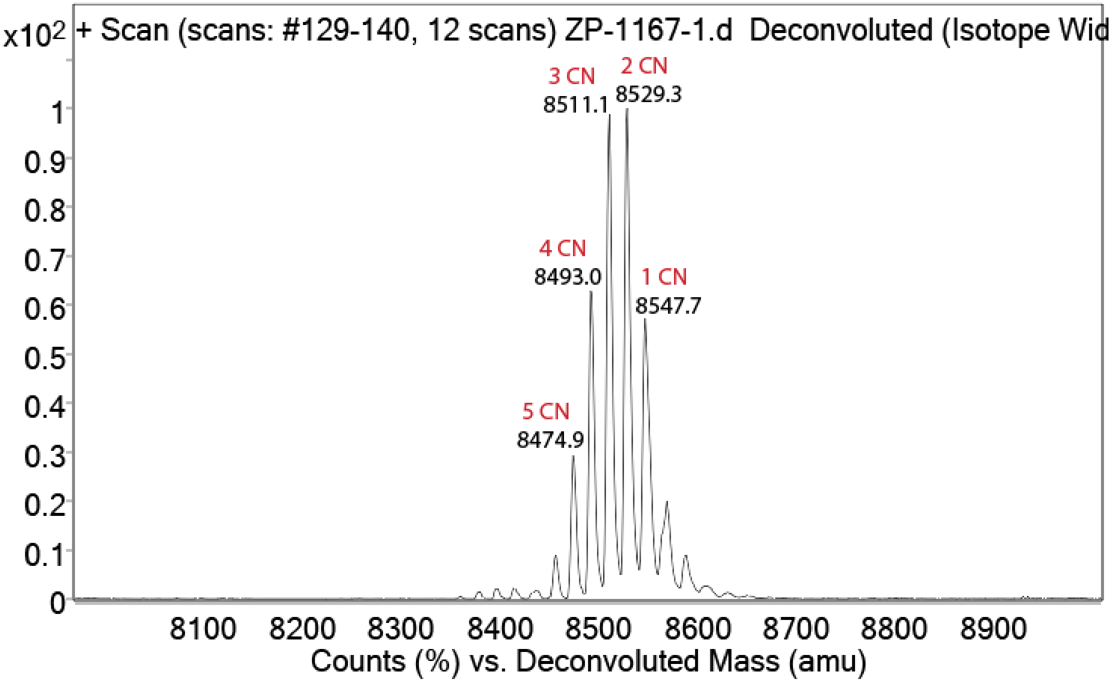

Modification of Nitrile Ubiquitin to Ketone with Boronic Acid

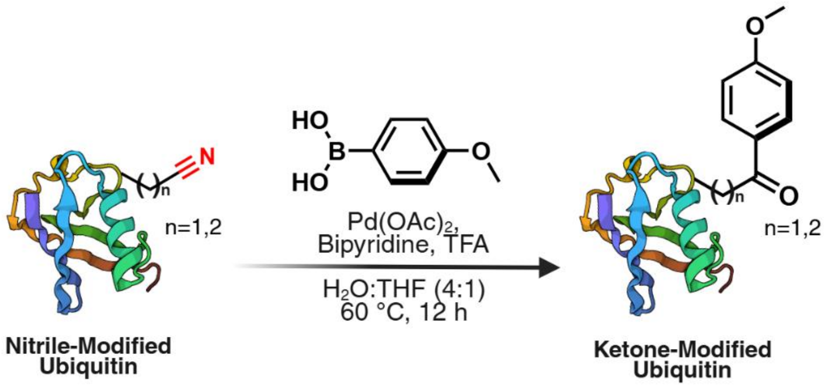

2 mg of nitrile-modified ubiquitin (0.468 mM final concentration) was dissolved in 350 µL of H_2O in a 1/2" dram vial, after which 2 µL of TFA was added. Next, 2 mg of boronic acid (~25 mM final concentration) dissolved in 50 µL THF was added to the mixture. N_2 was then bubbled into the solution for 3 minutes using an 18G x 1 ½" needle and balloon. In a separate vial, 1 mg of bipyridine (~12.8 mM final concentration) and 1 mg of $Pd(OAc)_2$ (~9 mM final concentration) were dissolved in 100 µL of 1:1 THF:H_2O and stirred for 15 minutes at room temperature to generate a pre-formed bipyridine-palladium complex. This pre-formed complex was then added to the reaction mixture, and N_2 was bubbled for an additional 2 minutes. The reaction vial was then sealed and stirred for 12 hours at 60 °C. The Pd was quenched by addition of 200 µL 1 M aqueous L-cysteine and 10 µL of 1 M NaOH. The crude reaction mixture was passed through Amicon™ Ultra 3 kDa spin-concentrator and washed with H_2O (7×0.5 mL) to remove the small molecule impurities. The modified ubiquitin was then analyzed using LC-MS, which revealed >95% conversion of nitrile to the ketone product, with 1-4 boronic acid ketone modifications observed (see table below). Note that a similar result was obtained using 10 mM NaP buffer (pH 7.4) in place of H_2O for the reaction.

Modification	Mass	Conversion
Unmodified	8565.1	n.d.
1 Boronic Acid	8656.1 (**+91**)	20%
2 Boronic Acid	8747.1 (**+91**)	45%
3 Boronic Acid	8838.2 (**+91**)	28%
4 Boronic Acid	8929.2 (**+91**)	7%

MS of Ketone-Modified Ubiquitin

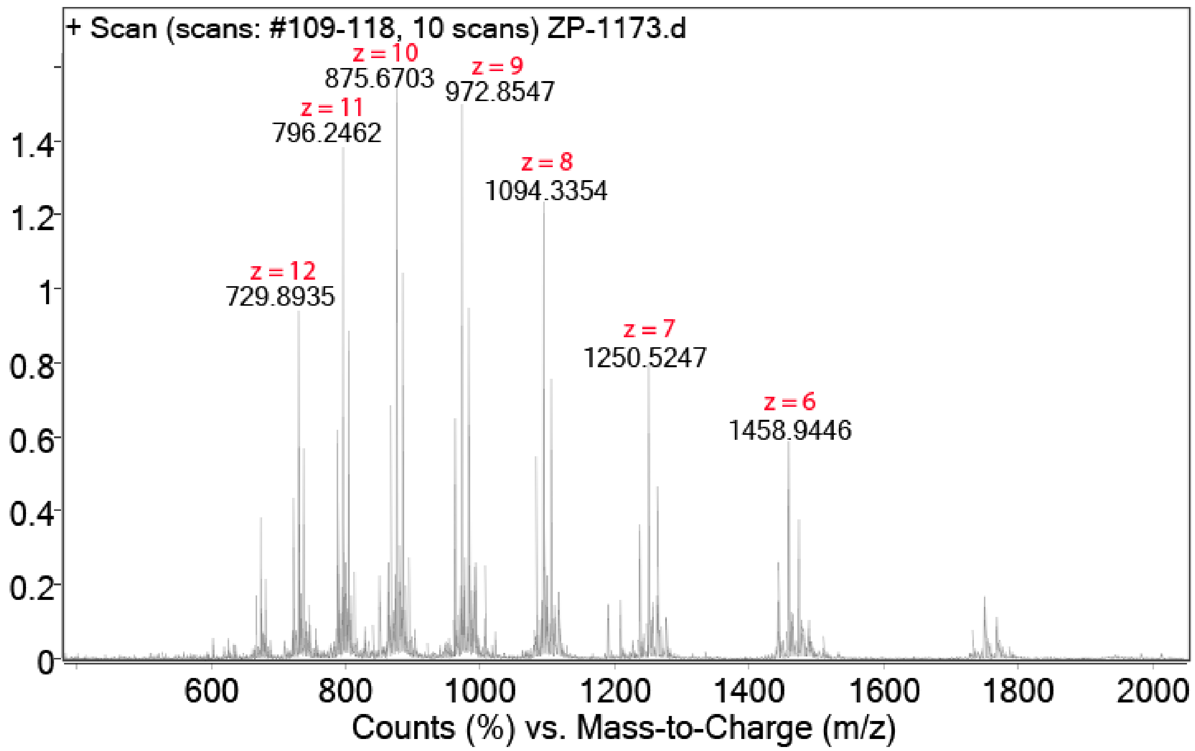

Deconvoluted MS of Ketone-Modified Ubiquitin

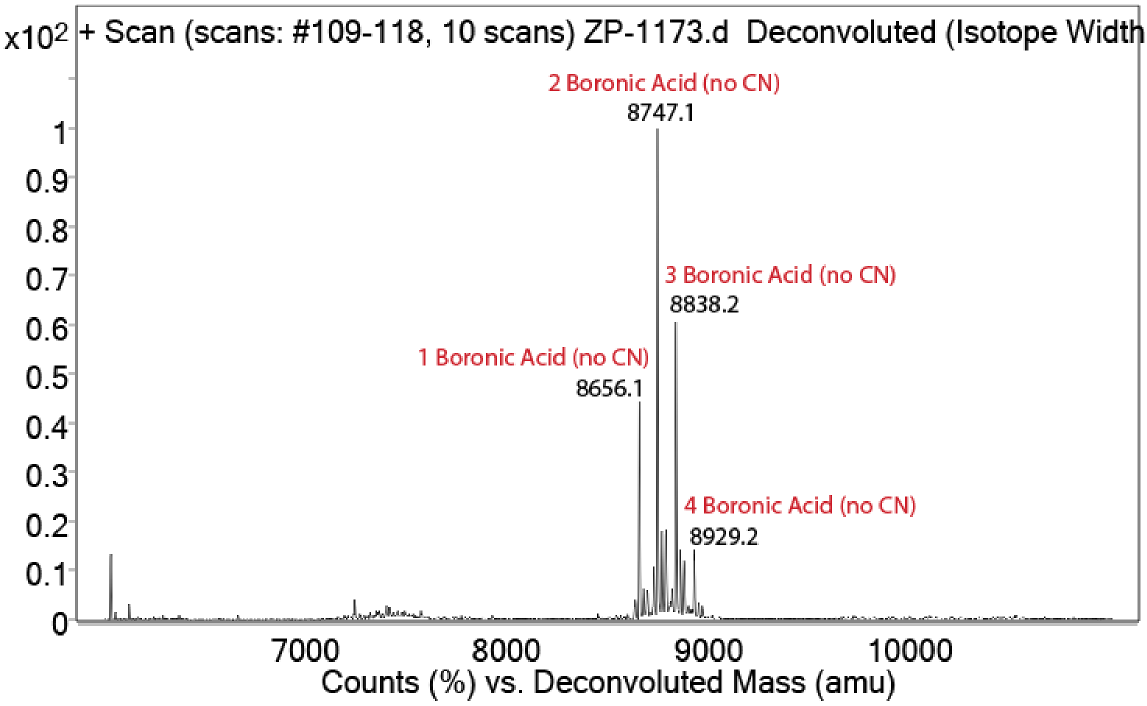

Supplementary Fig. 21: Screening of Electronic Effects for Boronic Acid Nitrile Modification on Protein

To further evaluate the electronic effects of different phenylboronic acids on the efficiency of the boronic acid carbometallation reaction on nitrile-modified protein, we screened electron withdrawing and electron donating phenylboronic acids using nitrile-modified bovine serum albumin (BSA). As expected, results showed electron-donating phenylboronic acids (NMe_2, Ome) to be the most reactive boronic acids for the carbometallation reaction on protein, aligning with the results on small molecule and peptide.

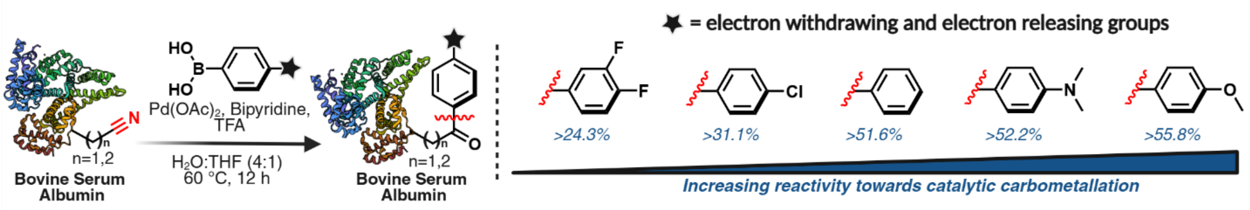

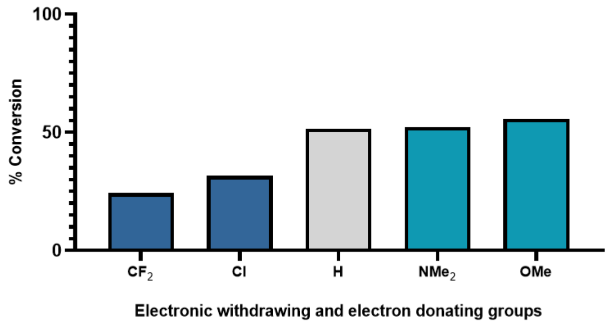

General Procedure for Nitrile Boronic Acid Carbometallation on Protein (GP-XVI):
2 mg of the appropriate nitrile-modified protein was dissolved in 350 µL of H_2O in a 1/2” dram vial, after which 2 µL of TFA was added. Next, 2 mg of boronic acid (~25 mM final concentration) dissolved in 50 µL THF was added to the mixture. N_2 was then bubbled into the solution for 3 minutes using an 18G x 1 ½” needle and balloon. In a separate vial, 1 mg of bipyridine (~12.8 mM final concentration) and 1 mg of $Pd(OAc)_2$ (~9 mM final concentration) were dissolved in 100 µL of 1:1 THF:H_2O and stirred for 15 minutes at room temperature to generate a pre-formed bipyridine-palladium complex. This pre-formed complex was then added to the reaction mixture, and N_2 was bubbled for an additional 2 minutes. The reaction vial was then sealed and stirred for 12 hours at 60 °C. The Pd was quenched by addition of 200 µL 1 M aqueous L-cysteine and 10 µL of 1 M NaOH. The crude reaction mixture was passed through Amicon™ Ultra 3 kDa spin-concentrator and washed with H_2O (7×0.5 mL) to remove the small molecule impurities. The labeled protein was lyophilized, digested using SMART Digest™ Trypsin Kit by Thermo Scientific, and then analyzed using LC-MS/MS to determine the number of modified sites and conversion of nitrile to the carbometallation product.

Modification of BSA with Phenylboronic Acid

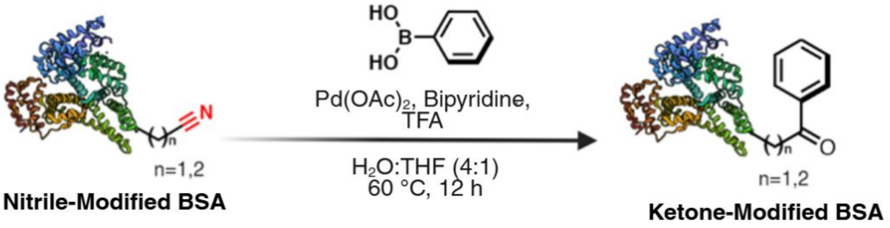

Reaction conducted according to **GP-XVI**. 2 mg of nitrile-modified bovine serum albumin (BSA) (60 µM final concentration) was dissolved in 350 µL of H_2O in a 1/2" dram vial, after which 2 µL of TFA was added. Next, 2 mg of phenylboronic acid dissolved in 50 µL THF was added to the mixture. N_2 was then bubbled into the solution for 3 minutes using an 18G x 1 ½" needle and balloon. In a separate vial, 1 mg of bipyridine and 1 mg of $Pd(OAc)_2$ were dissolved in 100 µL of 1:1 THF:H_2O and stirred for 15 minutes at room temperature to generate a pre-formed bipyridine-palladium complex. This pre-formed complex was then added to the reaction mixture, and N_2 was bubbled for an additional 2 minutes. The reaction vial was then sealed and stirred for 12 hours at 60 °C. The Pd was quenched by addition of 200 µL 1 M aqueous L-cysteine and 10 µL of 1 M NaOH. The crude reaction mixture was passed through Amicon™ Ultra 3 kDa spin-concentrator and washed with H_2O (7×0.5 mL) to remove the small molecule impurities. The labeled protein was lyophilized, digested using SMART Digest™ Trypsin Kit by Thermo Scientific, and then analyzed using LC-MS/MS. 5 modification sites were observed, with 51.6% conversion of nitrile sites to the carbometallation product.

MS/MS Analysis of Digested Modified Bovine Serum Albumin

Identified peptide fragment (**2 sites**): GLVLIAFSQYLQQCPFDEHV (Sequence: AA 21-40, [Q32, Q33])

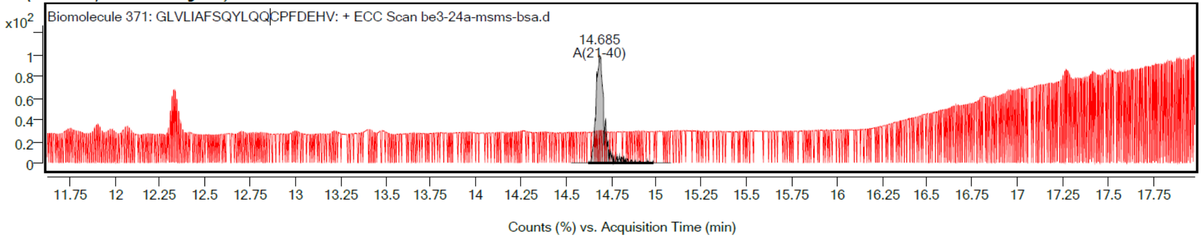

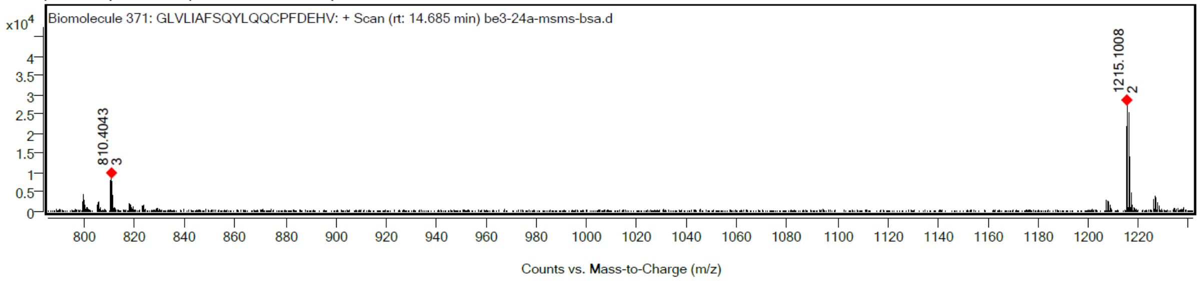

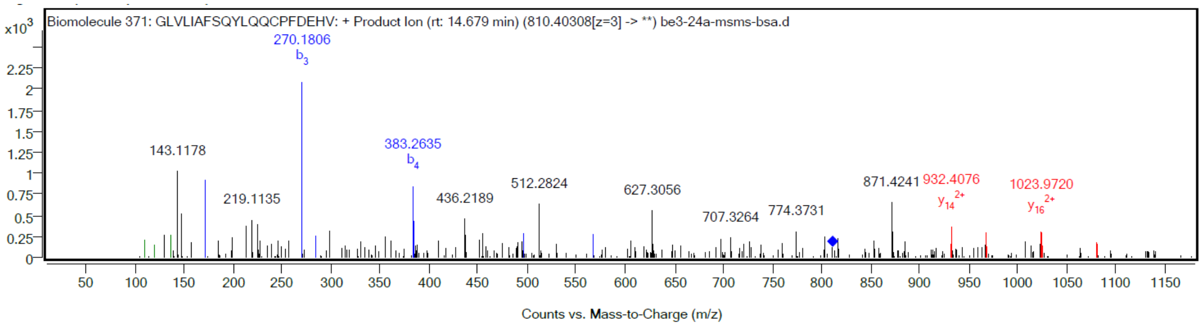

Fragment Spectrum Peaks

m/z	Diff (ppm)	Abund	Ion
932.4076	2.63	362	y14
967.9318	-3.29	295	y15
1023.9720	-2.94	306	y16
1080.5163	-4.96	176	y17
171.1132	-2.50	915	b2
270.1806	2.17	2081	b3
383.2635	4.67	836	b4
496.3494	-0.19	284	b5
567.3833	5.57	269	b6
284.1949	6.94	254	b6
110.0719	-5.69	209	H
120.0819	-9.76	143	F
136.0760	-2.28	259	Y

Identified peptide fragment (**2 sites**): NGVFQECCQAEDK (Sequence: AA 161-173, [Q165, Q169])

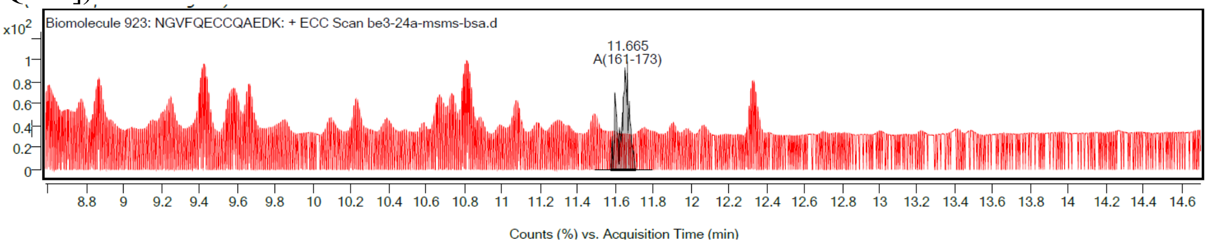

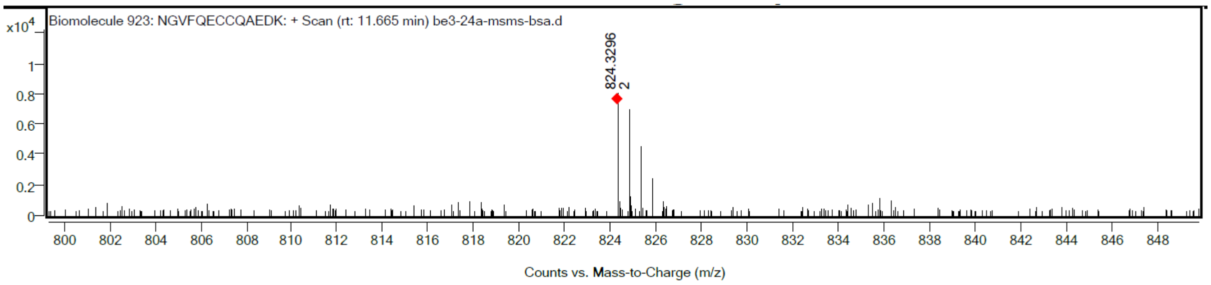

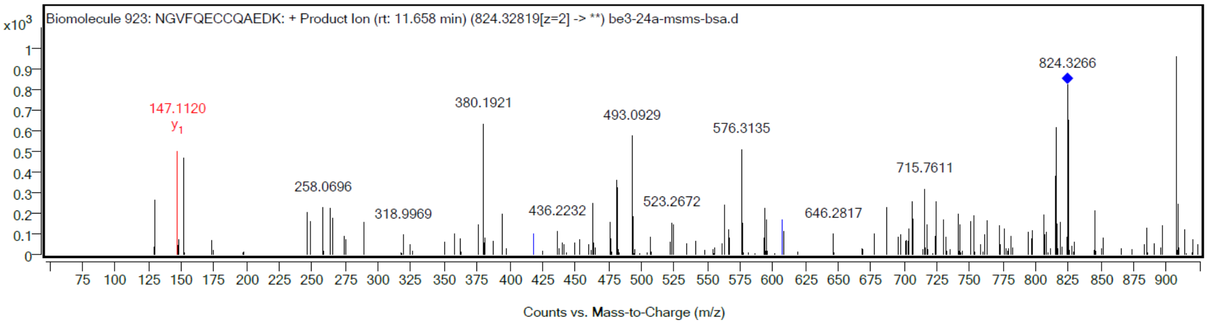

m/z	*Fragment Spectrum Peaks* Diff (ppm)	Abund	Ion
147.1120	5.69	501	y1
418.2093	-1.96	101	b4
607.2726	24.44	169	b5
825.3308	4.10	653	Precursor

Identified peptide fragment (**1 site**): DVCKNYQEAKDAFLG (Sequence: AA 313-327, Q319)

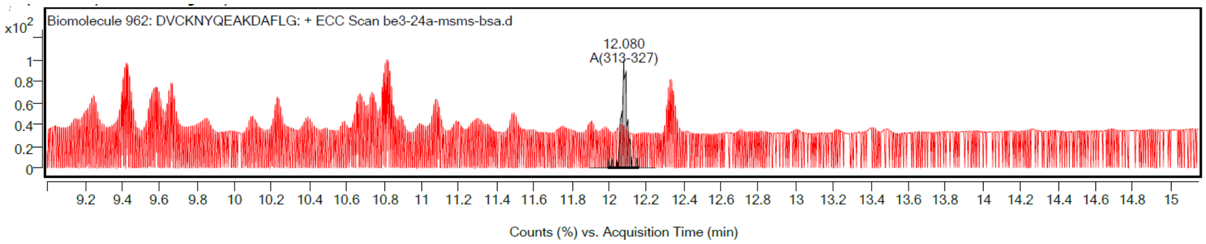

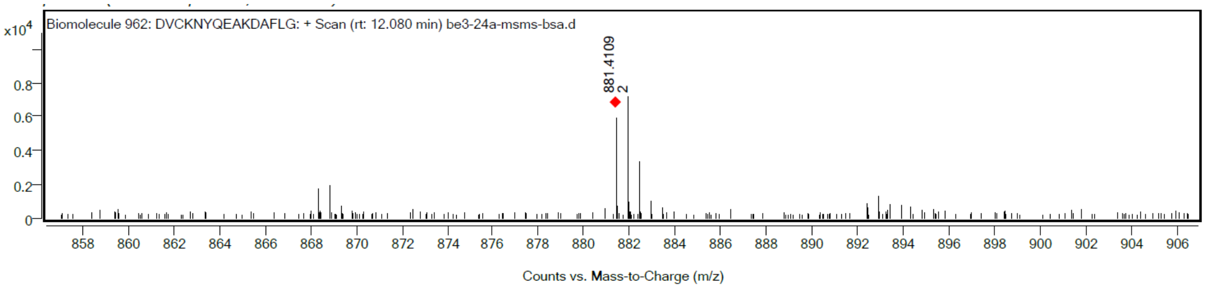

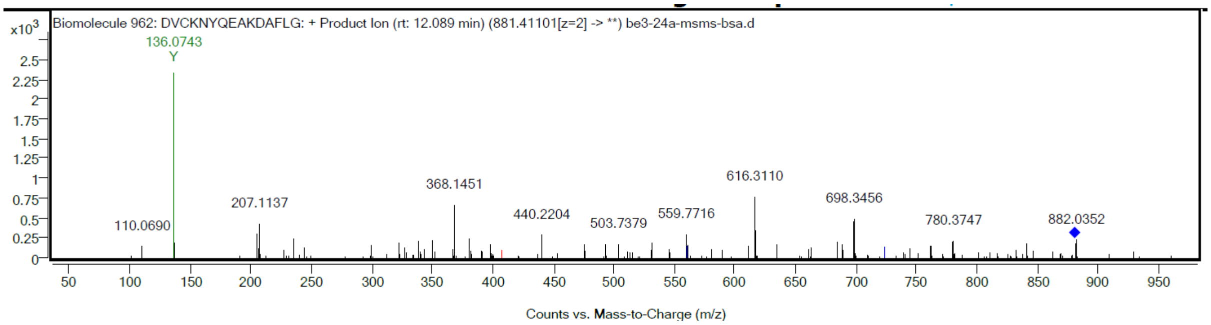

m/z	*Fragment Spectrum Peaks* Diff (ppm)	Abund	Ion
407.2308	-4.62	88	y4
560.2722	-40.11	143	b5
723.3226	-13.26	133	b6
136.0743	10.14	2324	Y
881.4157	-5.02	175	Precursor
881.9073	6.39	132	Precursor

Modification of BSA with 4-Dimethylaminophenylboronic Acid

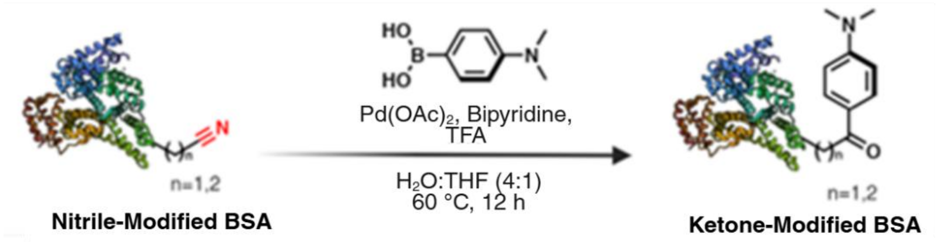

Reaction conducted according to **GP-XVI**. 2 mg of nitrile-modified bovine serum albumin (BSA) (60 µM final concentration) was dissolved in 350 µL of H_2O in a 1/2” dram vial, after which 2 µL

of TFA was added. Next, 2 mg of 4-dimethylaminophenylboronic acid dissolved in 50 µL THF was added to the mixture. N_2 was then bubbled into the solution for 3 minutes using an 18G x 1 ½" needle and balloon. In a separate vial, 1 mg of bipyridine and 1 mg of $Pd(OAc)_2$ were dissolved in 100 µL of 1:1 THF:H_2O and stirred for 15 minutes at room temperature to generate a pre-formed bipyridine-palladium complex. This pre-formed complex was then added to the reaction mixture, and N_2 was bubbled for an additional 2 minutes. The reaction vial was then sealed and stirred for 12 hours at 60 °C. The Pd was quenched by addition of 200 µL 1 M aqueous L-cysteine and 10 µL of 1 M NaOH. The crude reaction mixture was passed through Amicon™ Ultra 3 kDa spin-concentrator and washed with H_2O (7×0.5 mL) to remove the small molecule impurities. The labeled protein was lyophilized, digested using SMART Digest™ Trypsin Kit by Thermo Scientific, and then analyzed using LC-MS/MS. 4 modification sites were observed, with 52.2% conversion of nitrile sites to the carbometallation product.

MS/MS Analysis of Digested Modified Bovine Serum Albumin

Identified peptide fragment (**2 sites**): YNGVFQECCQAEDKGACLLPK (Sequence: AA 160-180, [N162, Q170])

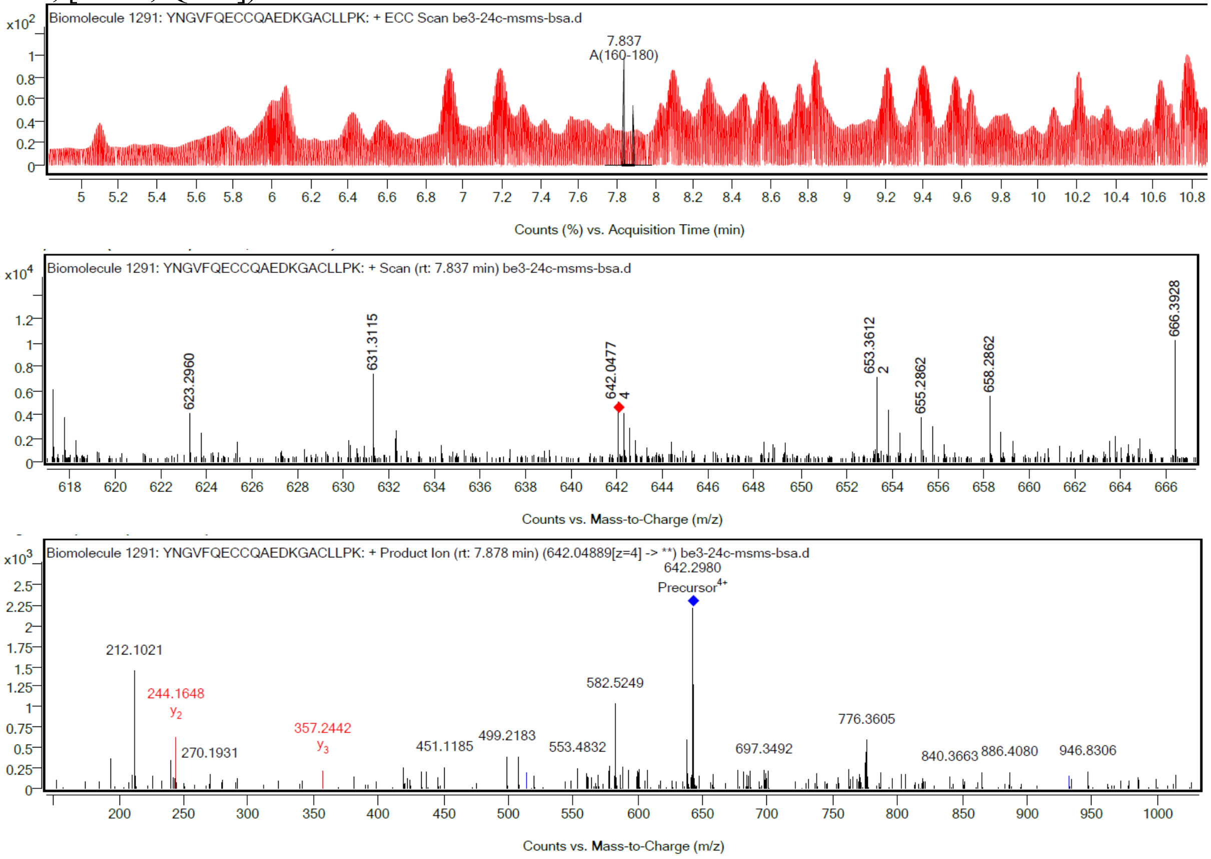

Fragment Spectrum Peaks

m/z	Diff (ppm)	Abund	Ion
357.2442	15.34	209	y3
514.2046	31.43	197	b8
932.4054	-6.76	151	b15
642.0431	7.28	1343	Precursor
642.2980	0.91	2208	Precursor
642.5485	1.48	1808	Precursor
642.7980	3.58	1273	Precursor

Identified peptide fragment (**1 site**): DNQDTISSKLK (Sequence: AA 265-275, N266)

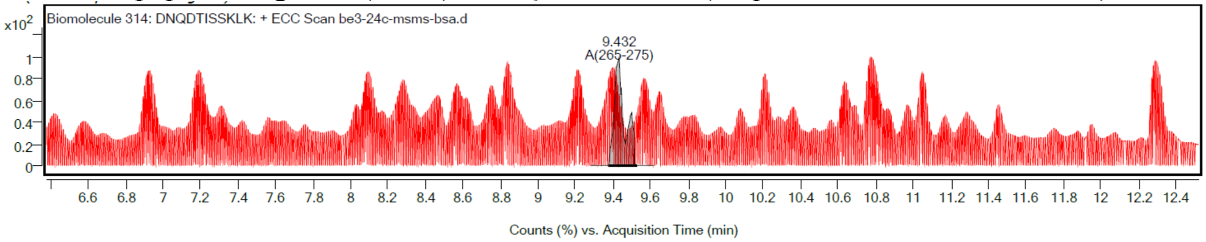

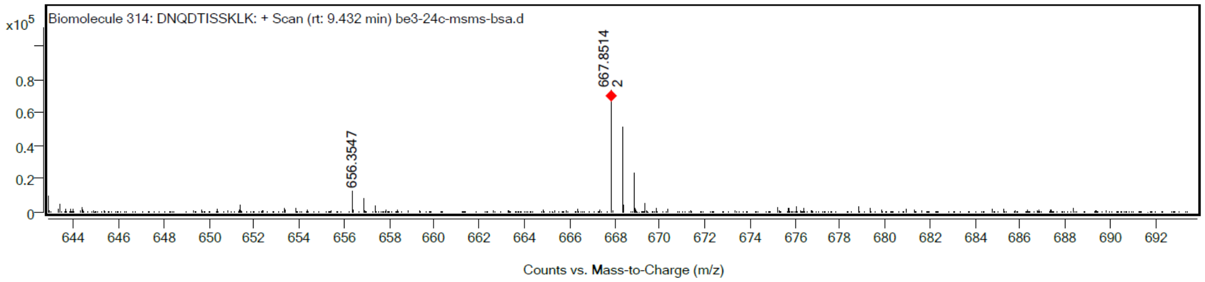

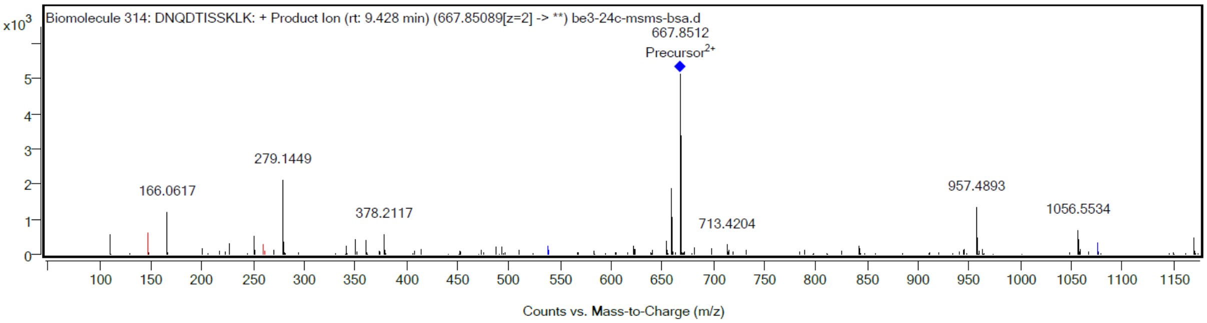

Fragment Spectrum Peaks

m/z	Diff (ppm)	Abund	Ion
147.1127	0.95	608	y1
260.2016	-18.13	285	y2
1075.5006	4.49	331	b9
538.2517	8.63	242	b9
667.8512	-0.05	5142	Precursor
668.3515	1.98	3370	Precursor
668.8472	10.95	656	Precursor

Identified peptide fragment (**1 site**): EPQNLIKQNCDQFEK (Sequence: AA 382-396, N385)

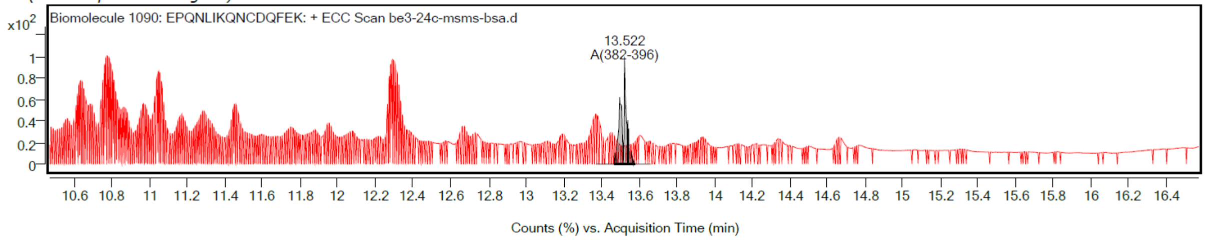

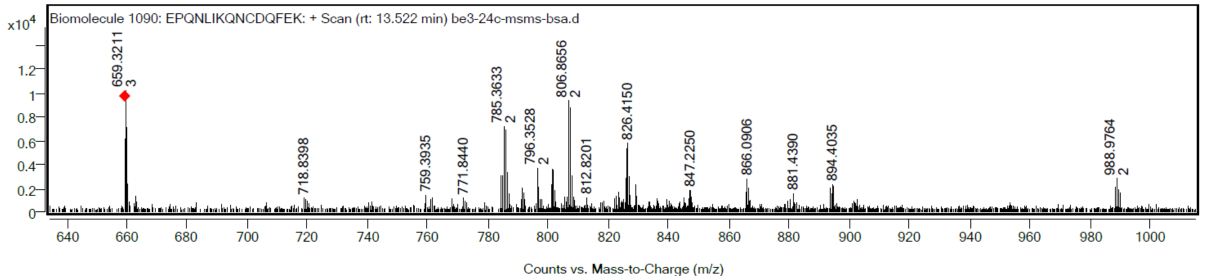

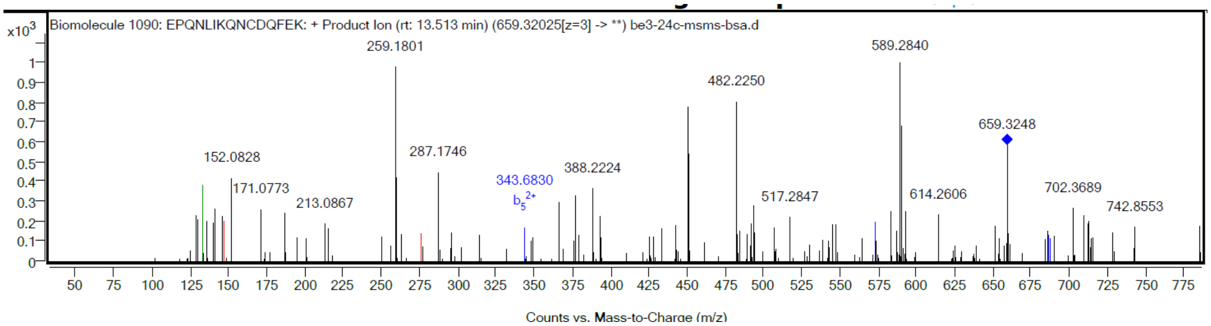

Fragment Spectrum Peaks

m/z	Diff (ppm)	Abund	Ion
147.1139	-7.37	200	y1
276.1598	-16.02	137	y2
573.2722	-9.60	195	b4
686.3633	-18.24	127	b5
343.6830	-11.46	164	b5
133.0417	9.74	382	C

Modification of BSA with 4-Methoxyphenylboronic Acid

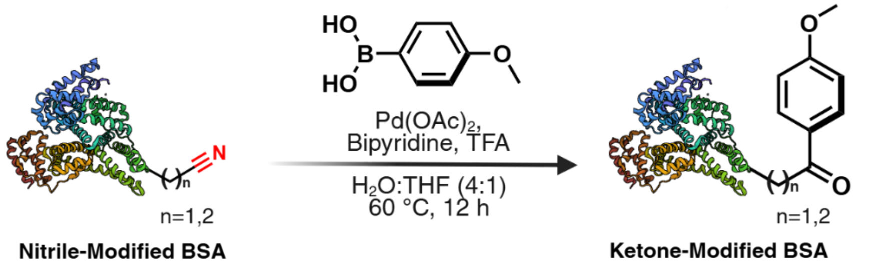

Reaction conducted according to **GP-XVI**. 2 mg of nitrile-modified bovine serum albumin (BSA) (60 µM final concentration) was dissolved in 350 µL of H_2O in a 1/2” dram vial, after which 2 µL of TFA was added. Next, 2 mg of 4-methoxyphenylboronic acid dissolved in 50 µL THF was added to the mixture. N_2 was then bubbled into the solution for 3 minutes using an 18G x 1 ½” needle and balloon. In a separate vial, 1 mg of bipyridine and 1 mg of $Pd(OAc)_2$ were dissolved in 100 µL of 1:1 THF:H_2O and stirred for 15 minutes at room temperature to generate a pre-formed bipyridine-palladium complex. This pre-formed complex was then added to the reaction mixture, and N_2 was bubbled for an additional 2 minutes. The reaction vial was then sealed and stirred for 12 hours at 60 °C. The Pd was quenched by addition of 200 µL 1 M aqueous L-cysteine and 10 µL of 1 M NaOH. The crude reaction mixture was passed through Amicon™ Ultra 3 kDa spin-concentrator and washed with H_2O (7×0.5 mL) to remove the small molecule impurities. The labeled protein was lyophilized, digested using SMART Digest™ Trypsin Kit by Thermo Scientific, and then analyzed using LC-MS/MS. 8 modification sites were observed, with 55.8% conversion of nitrile sites to the carbometallation product.

MS/MS Analysis of Digested Modified Bovine Serum Albumin

Identified peptide fragment (**2 sites**): GLVLIAFSQYLQQCPFDEHV (Sequence: AA 21-40, [Q32, Q33])

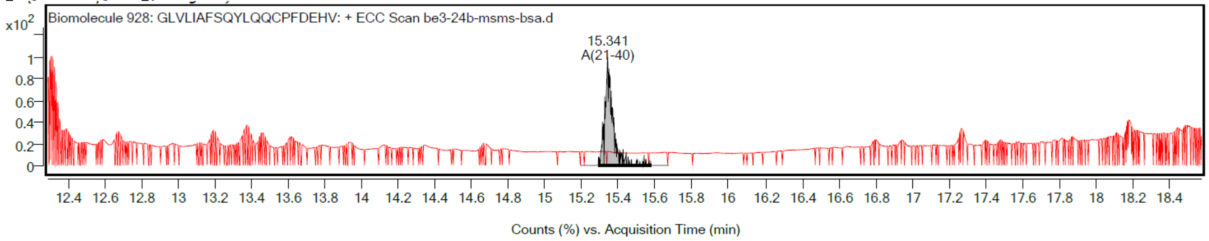

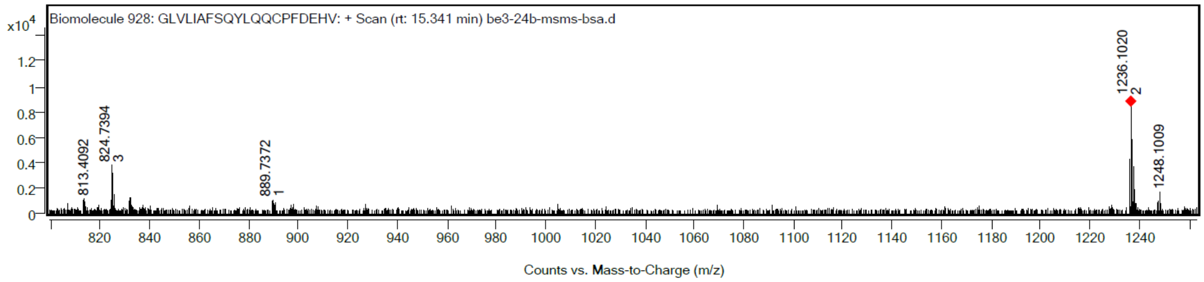

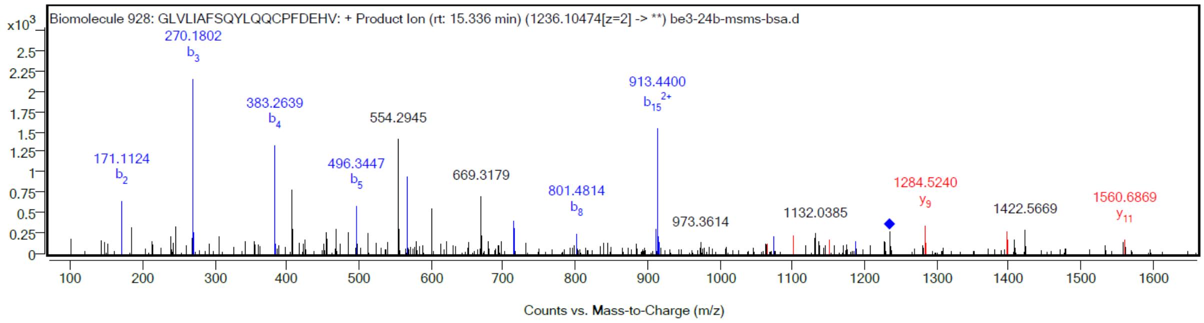

Fragment Spectrum Peaks

m/z	Diff (ppm)	Abund	Ion
1065.4380	-3.18	117	y8
1284.5240	0.10	342	y9
1397.6080	0.19	268	y10
1560.6869	-9.86	164	y11
1102.0093	7.85	212	y17
1151.5412	9.49	162	y18
171.1124	2.20	641	b2
270.1802	3.75	2146	b3
383.2639	3.72	1327	b4
496.3447	9.28	576	b5
567.3856	1.50	942	b6
714.4540	1.29	397	b7
801.4814	6.88	242	b8
911.5398	-5.35	303	b9
1074.5910	6.70	206	b10
1187.6626	16.63	145	b11
913.4400	27.70	1533	b15

Identified peptide fragment (**2 sites**): VLIAFSQYLQQCPFDEHVK (Sequence: AA 23-41, [Q29, Q32])

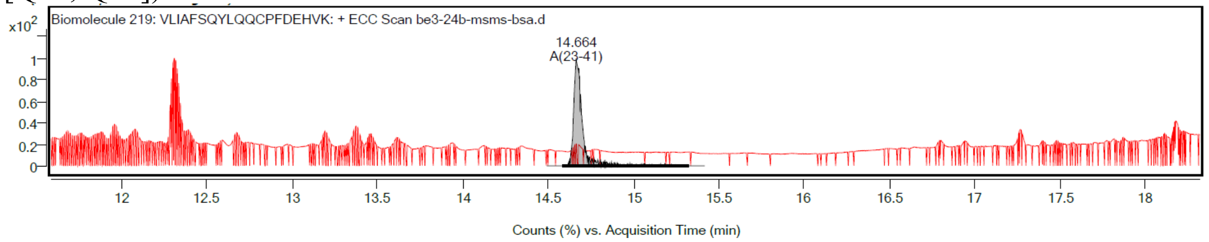

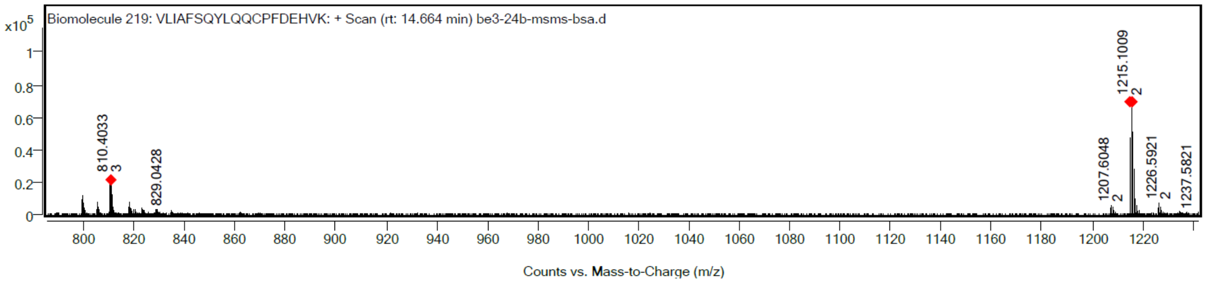

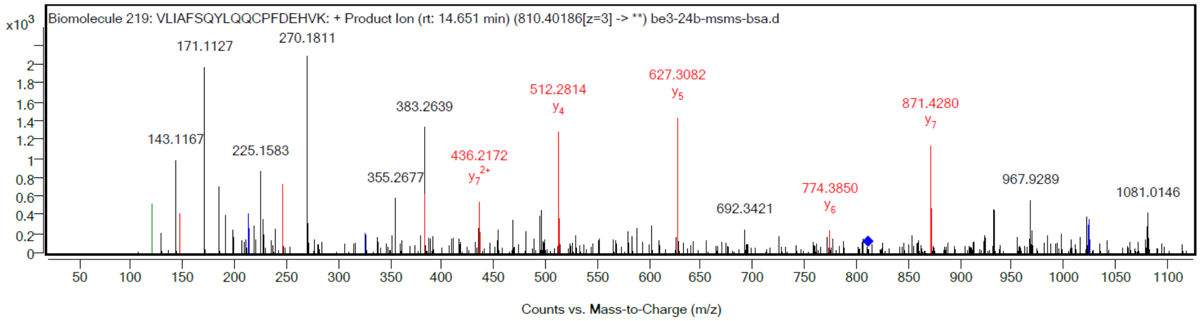

Fragment Spectrum Peaks

m/z	Diff (ppm)	Abund	Ion
147.1113	9.99	417	y1
246.1811	0.28	726	y2
383.2386	3.92	621	y3
512.2814	2.52	1275	y4
627.3082	2.39	1430	y5
774.3850	-8.87	227	y6
871.4280	3.30	1129	y7
436.2172	4.33	530	y7
213.1603	-2.49	414	b2
326.2441	-0.94	206	b3
1024.4691	13.80	352	b16
120.0808	-0.50	517	F

Identified peptide fragment (**1 site**): KDVCKNYQEAK (Sequence: AA 312-322, N317)

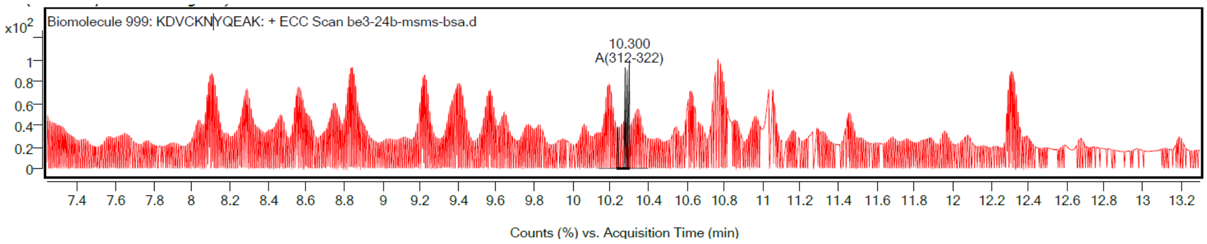

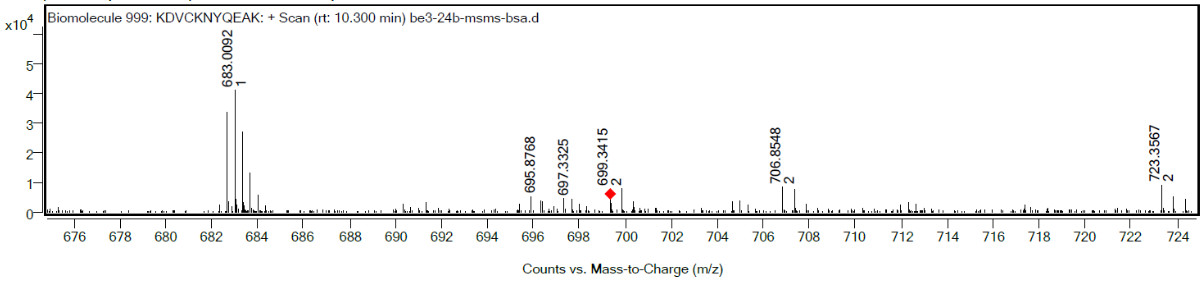

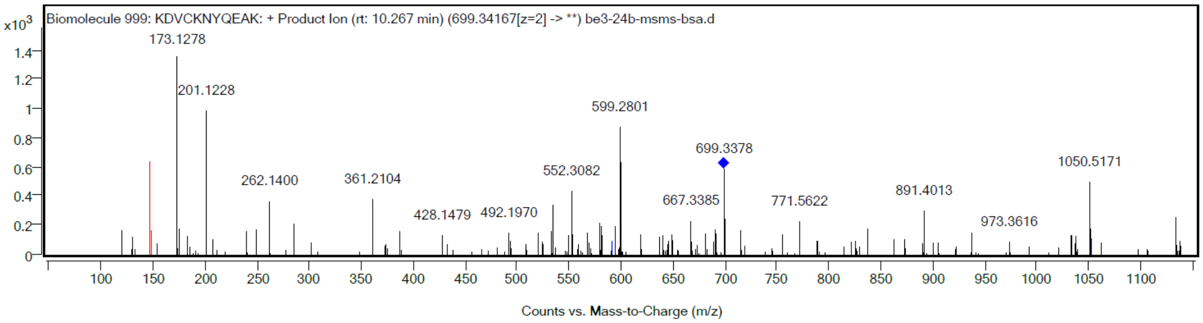

Fragment Spectrum Peaks

m/z	Diff (ppm)	Abund	Ion
147.1133	-3.55	634	y1
1052.5319	-42.68	103	b8
591.2579	17.83	87	b9

Identified peptide fragment (**4 sites**): QNLIKQNCDQFEK (Sequence: AA 384-396, [Q384, N385, Q389, N390])

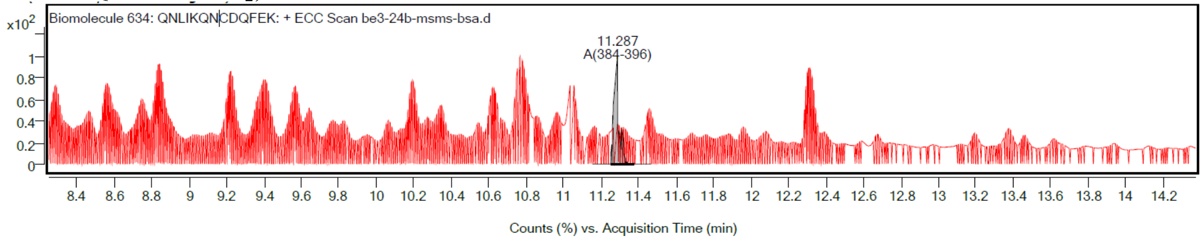

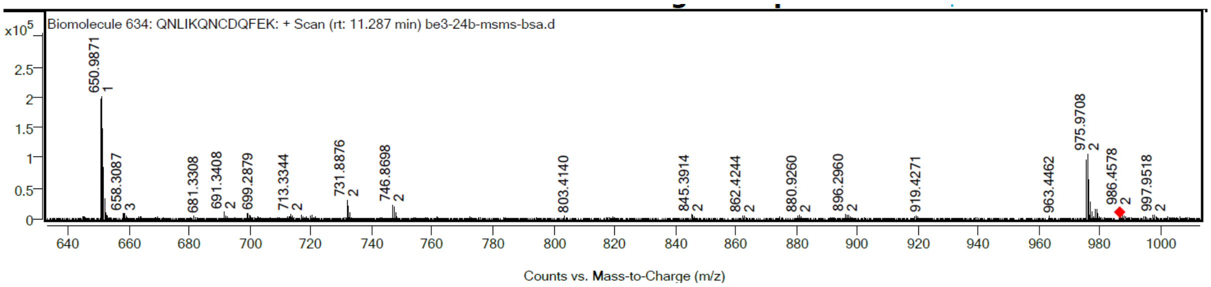

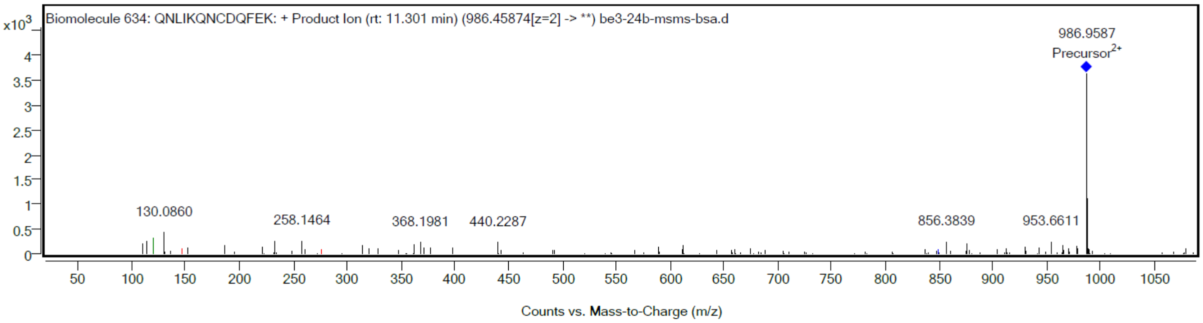

Fragment Spectrum Peaks

m/z	Diff (ppm)	Abund	Ion
147.1125	2.35	113	y1
276.1535	-31.23	93	y4
848.8833	0.66	83	b11
120.0795	10.47	316	F
986.4573	0.60	2869	Precursor
986.9587	0.89	3630	Precursor
987.4505	10.84	1121	Precursor
987.9530	10.07	110	Precursor

Modification of BSA with 4-Chlorophenylboronic Acid

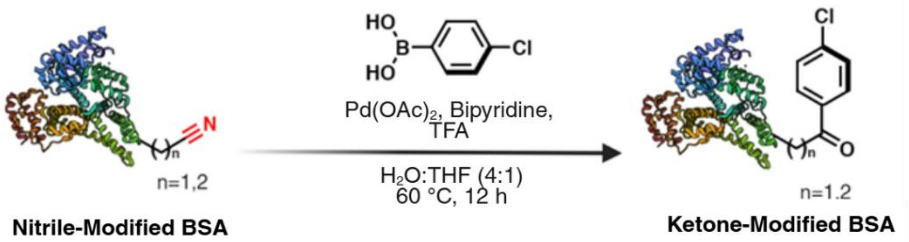

Reaction conducted according to **GP-XVI**. 2 mg of nitrile-modified bovine serum albumin (BSA) (60 µM final concentration) was dissolved in 350 µL of H_2O in a 1/2” dram vial, after which 2 µL of TFA was added. Next, 2 mg of 4-chlorophenylboronic acid dissolved in 50 µL THF was added to the mixture. N_2 was then bubbled into the solution for 3 minutes using an 18G x 1 ½” needle and balloon. In a separate vial, 1 mg of bipyridine and 1 mg of $Pd(OAc)_2$ were dissolved in 100 µL of 1:1 THF:H_2O and stirred for 15 minutes at room temperature to generate a pre-formed bipyridine-palladium complex. This pre-formed complex was then added to the reaction mixture, and N_2 was bubbled for an additional 2 minutes. The reaction vial was then sealed and stirred for 12 hours at 60 °C. The Pd was quenched by addition of 200 µL 1 M aqueous L-cysteine and 10 µL of 1 M NaOH. The crude reaction mixture was passed through Amicon™ Ultra 3 kDa spin-concentrator and washed with H_2O (7×0.5 mL) to remove the small molecule impurities. The labeled protein was lyophilized, digested using SMART Digest™ Trypsin Kit by Thermo Scientific, and then analyzed using LC-MS/MS. 3 modification sites were observed, with 31.6% conversion of nitrile sites to the carbometallation product.

MS/MS Analysis of Digested Modified Bovine Serum Albumin

Identified peptide fragment (**1 site**): LVLIAFSQYLQQCPFDEHVK (Sequence: AA 22-41, Q32)

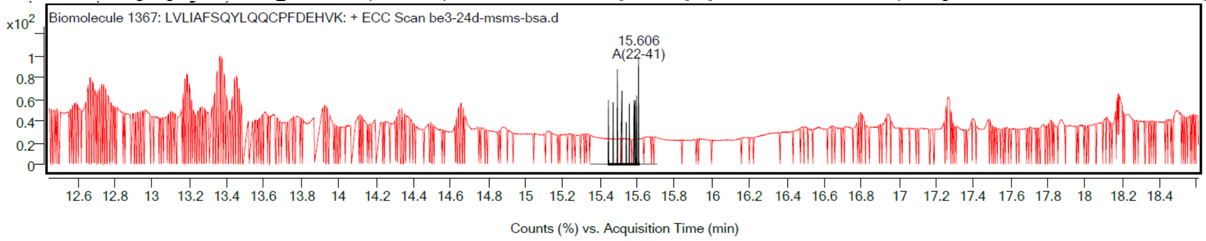

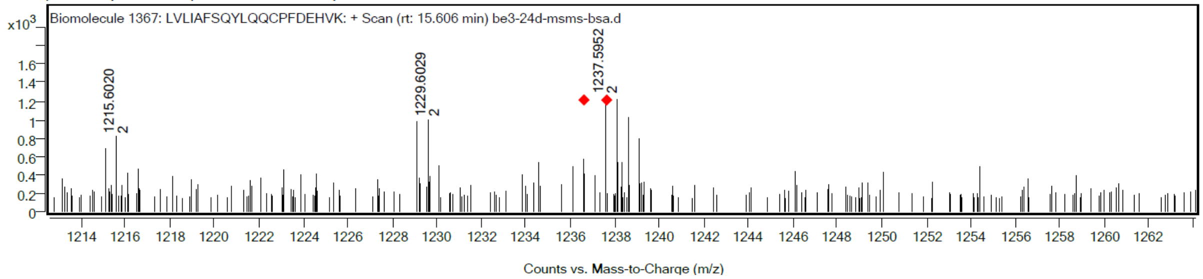

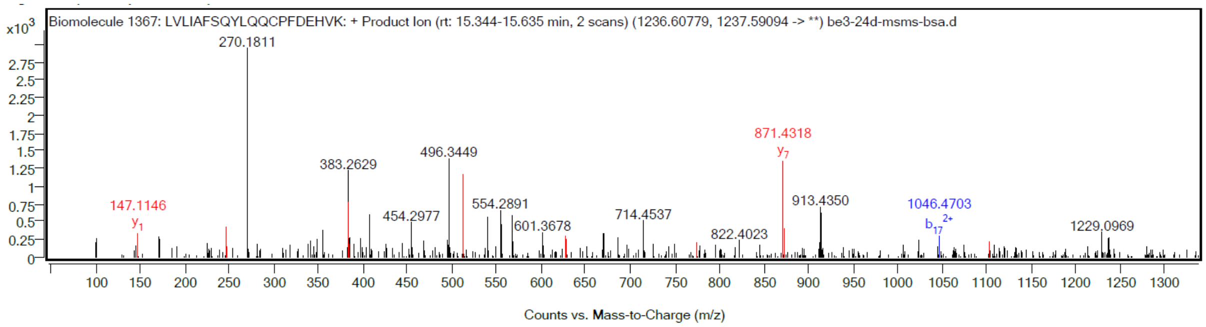

Fragment Spectrum Peaks

m/z	Diff (ppm)	Abund	Ion
147.1146	-12.48	335	y1
246.1820	-3.31	431	y2
383.2401	0.13	774	y3
512.2823	0.90	1164	y4
627.3086	1.76	303	y5
774.3742	4.97	210	y6
871.4318	-1.07	1352	y7
1102.5144	-14.29	222	y9
1046.4703	19.06	296	b17
1237.5979	7.08	280	Precursor

Identified peptide fragment (**2 sites**): NLIKQNCDQFEK (Sequence: AA 385-396, [Q389, Q390])

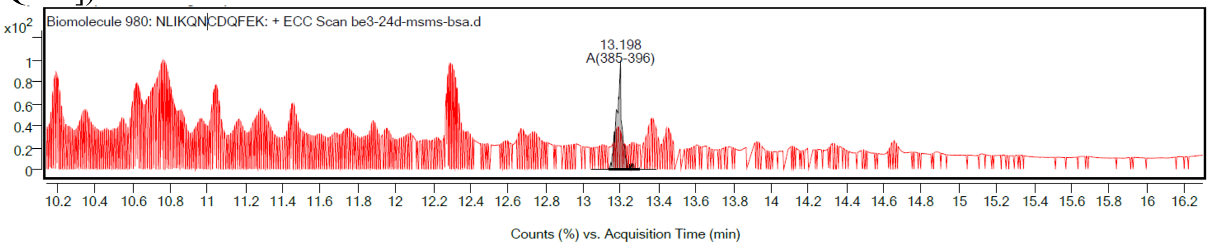

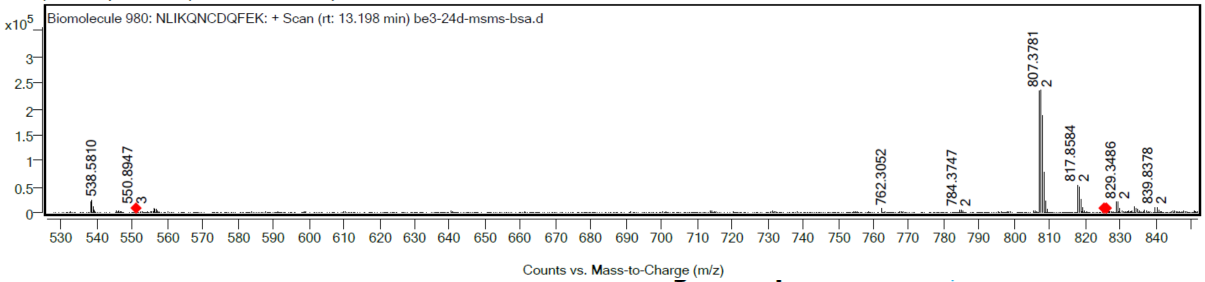

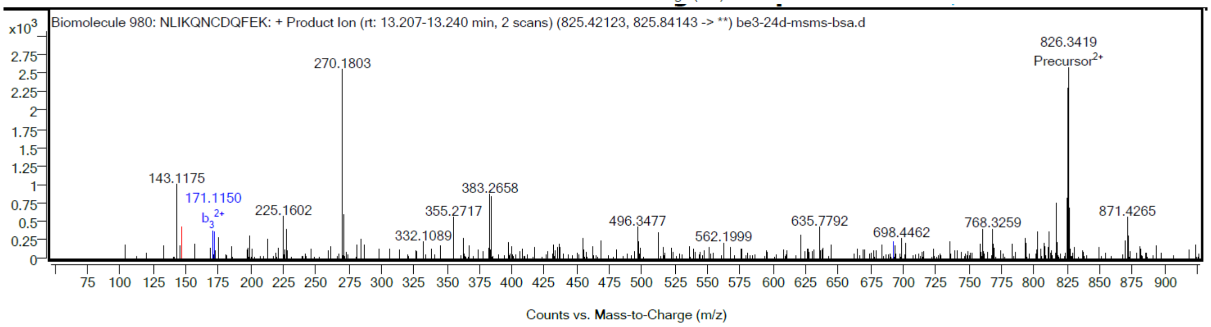

Fragment Spectrum Peaks

m/z	Diff (ppm)	Abund	Ion
147.1117	7.52	417	y1
692.3429	15.00	218	b5
171.1150	-12.61	369	b3
826.3419	1.09	2585	Precursor
826.8455	-1.27	680	Precursor

Modification of BSA with 3,4-Difluorophenylboronic Acid

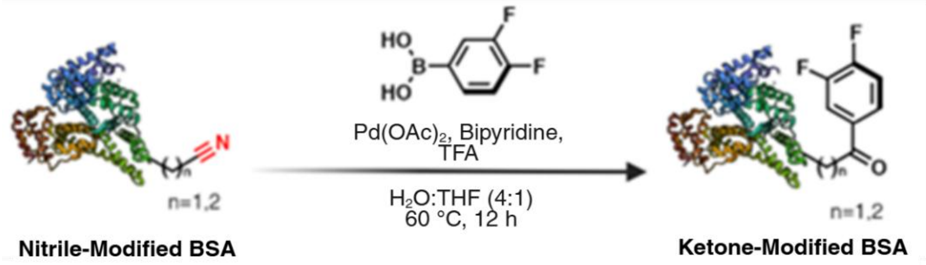

Reaction conducted according to **GP-XVI**. 2 mg of nitrile-modified bovine serum albumin (BSA) (60 μM final concentration) was dissolved in 350 μL of H_2O in a 1/2” dram vial, after which 2 μL of TFA was added. Next, 2 mg of 3,4-difluorophenylboronic acid dissolved in 50 μL THF was added to the mixture. N_2 was then bubbled into the solution for 3 minutes using an 18G x 1 ½” needle and balloon. In a separate vial, 1 mg of bipyridine and 1 mg of $Pd(OAc)_2$ were dissolved in 100 μL of 1:1 THF:H_2O and stirred for 15 minutes at room temperature to generate a pre-formed bipyridine-palladium complex. This pre-formed complex was then added to the reaction mixture, and N_2 was bubbled for an additional 2 minutes. The reaction vial was then sealed and stirred for 12 hours at 60 °C. The Pd was quenched by addition of 200 μL 1 M aqueous L-cysteine and 10 μL of 1 M NaOH. The crude reaction mixture was passed through Amicon^{TM} Ultra 3 kDa spin-concentrator and washed with H_2O (7×0.5 mL) to remove the small molecule impurities. The labeled protein was lyophilized, digested using SMART Digest^{TM} Trypsin Kit by Thermo Scientific, and then analyzed using LC-MS/MS. 2 modification sites were observed, with 24.3% conversion of nitrile sites to the carbometallation product.

MS/MS Analysis of Digested Modified Bovine Serum Albumin

Identified peptide fragment (**1 site**): CCQAEDKGACLLPK (Sequence: AA 167-180, Q169)

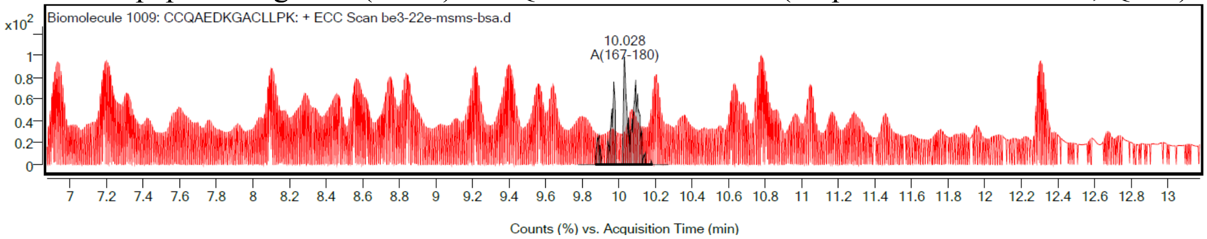

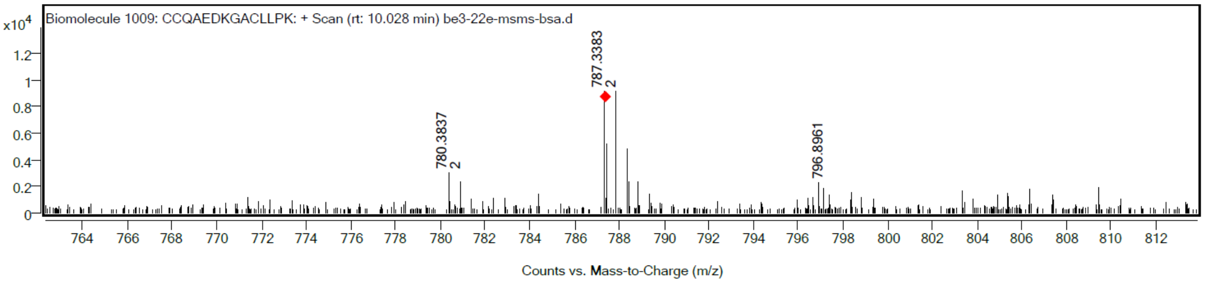

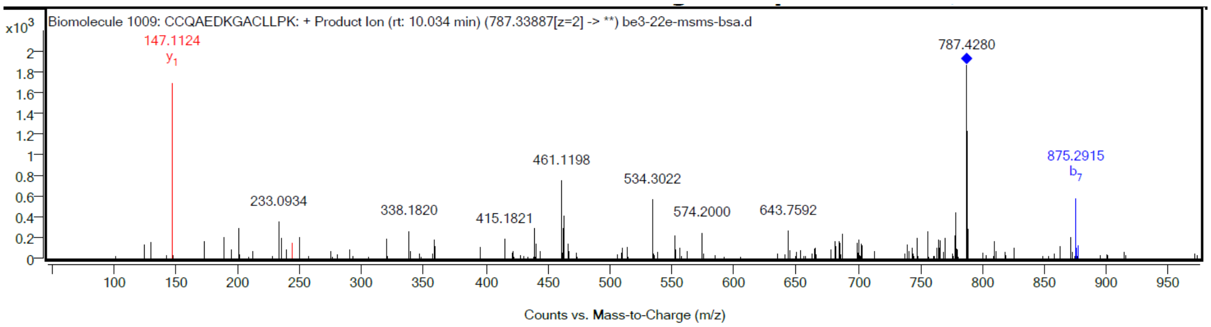

Fragment Spectrum Peaks

m/z	Diff (ppm)	Abund	Ion
147.1124	2.44	1683	y1
244.1674	-7.55	140	y2
875.2915	-4.69	568	b7
788.3391	6.80	274	Precursor

Identified peptide fragment (**1 site**): KDVCKNYQEAK (Sequence: AA 312-322, Q319)

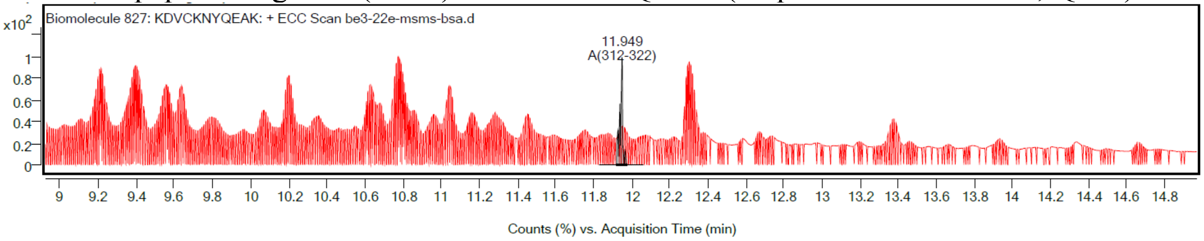

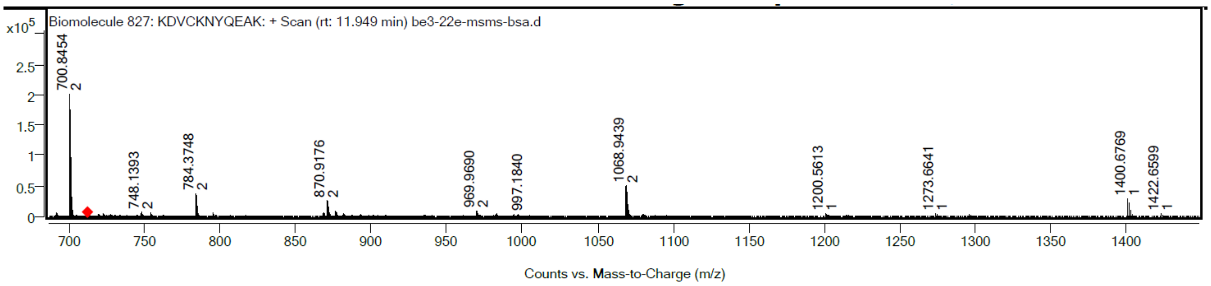

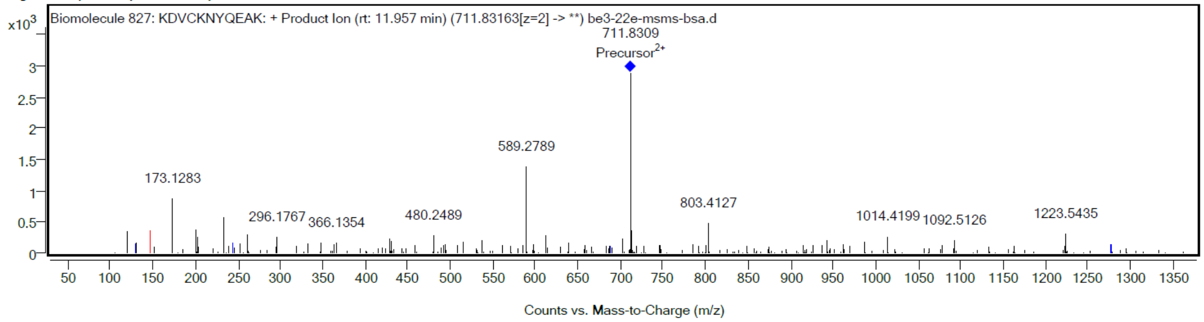

Fragment Spectrum Peaks

m/z	Diff (ppm)	Abund	Ion
147.1113	10.35	359	y1
129.1011	8.91	149	b1
244.1255	15.14	170	b2
688.3395	7.48	107	b6
1277.5454	4.55	136	b10
711.8309	-0.83	2885	Precursor
712.3311	1.22	2619	Precursor
712.8330	0.91	361	Precursor

Supplementary Fig. 22: Boronic Acid Modification of Nitrile on Diverse Proteins

Supplementary Fig. 22a: Scope of Boronic Acid Modification of Nitrile on Protein

Boronic Acid Modification of Lysozyme Chicken

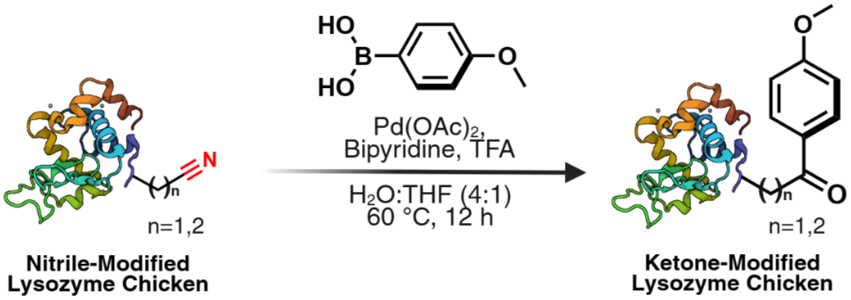

Reaction conducted according to **GP-XVI**. 2 mg of nitrile-modified lysozyme chicken (0.280 mM final concentration) was dissolved in 350 µL of H_2O in a 1/2" dram vial, after which 2 µL of TFA was added. Next, 2 mg of 4-methoxyphenylboronic acid dissolved in 50 µL THF was added to the mixture. N_2 was then bubbled into the solution for 3 minutes using an 18G x 1 ½" needle and balloon. In a separate vial, 1 mg of bipyridine and 1 mg of $Pd(OAc)_2$ were dissolved in 100 µL of 1:1 THF:H_2O and stirred for 15 minutes at room temperature to generate a pre-formed bipyridine-palladium complex. This pre-formed complex was then added to the reaction mixture, and N_2 was bubbled for an additional 2 minutes. The reaction vial was then sealed and stirred for 12 hours at 60 °C. The Pd was quenched by addition of 200 µL 1 M aqueous L-cysteine and 10 µL of 1 M NaOH. The crude reaction mixture was passed through Amicon™ Ultra 3 kDa spin-concentrator and washed with H_2O (7×0.5 mL) to remove the small molecule impurities. The labeled protein was lyophilized, digested using SMART Digest™ Trypsin Kit by Thermo Scientific, and then analyzed using LC-MS/MS. 2 distinct sites were modified with 4-methoxyphenylboronic acid.

MS/MS Analysis of Digested Modified Lysozyme Chicken

Identified peptide fragment (**1 site**): GYSLGNWVCAAKFESN: (Sequence: AA 22-37, N27)

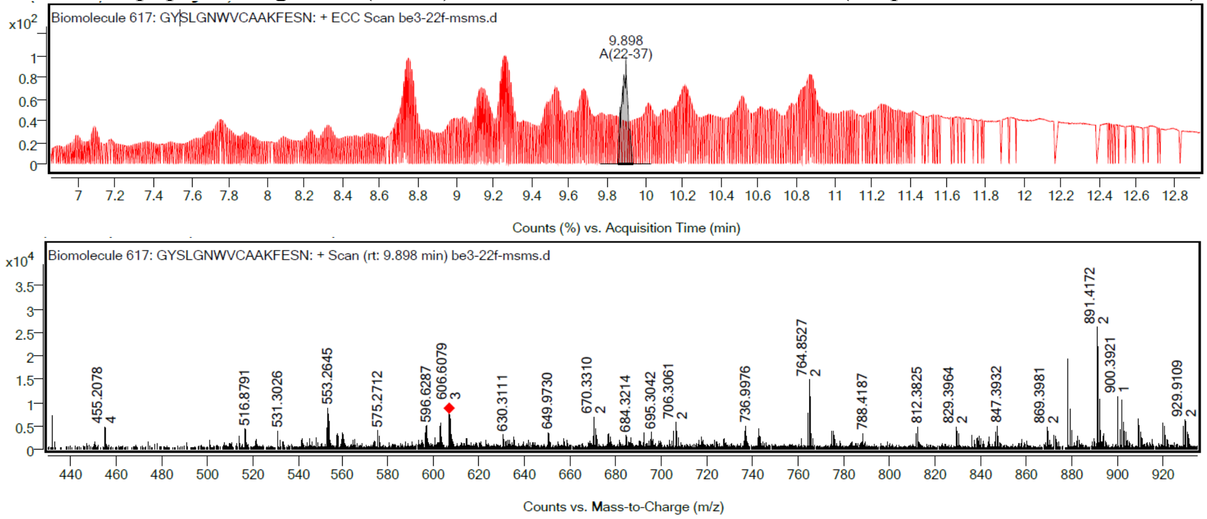

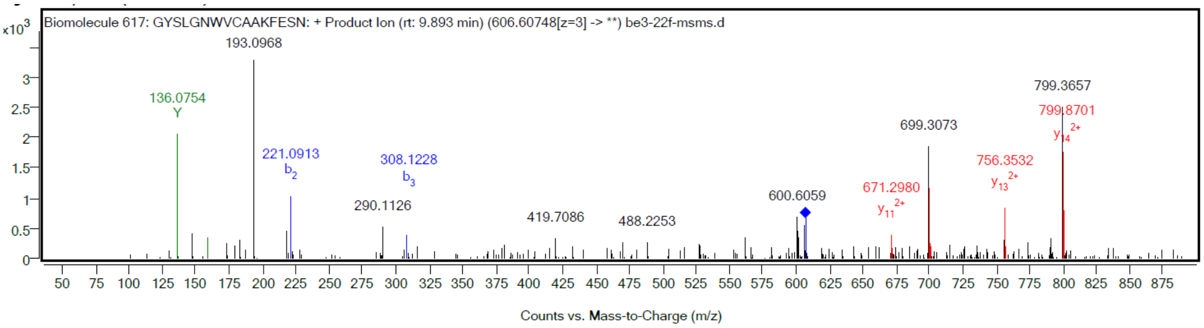

Fragment Spectrum Peaks

m/z	Diff (ppm)	Abund	Ion
671.2980	3.35	383	y11
699.8074	5.17	1160	y12
756.3532	-0.17	829	y13
799.8701	-1.36	1752	y14
221.0913	3.50	1017	b2
308.1228	4.06	383	b3
607.2633	12.63	660	b11
136.0754	1.82	2057	Y
159.0923	-4.05	342	W

Identified peptide fragment (**1 site**): NLCNIPCSALLSSDITASVNCAK: (Sequence: AA 74-96, N74)

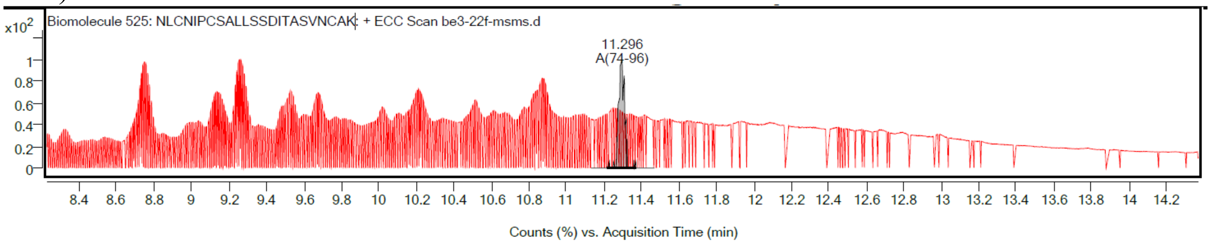

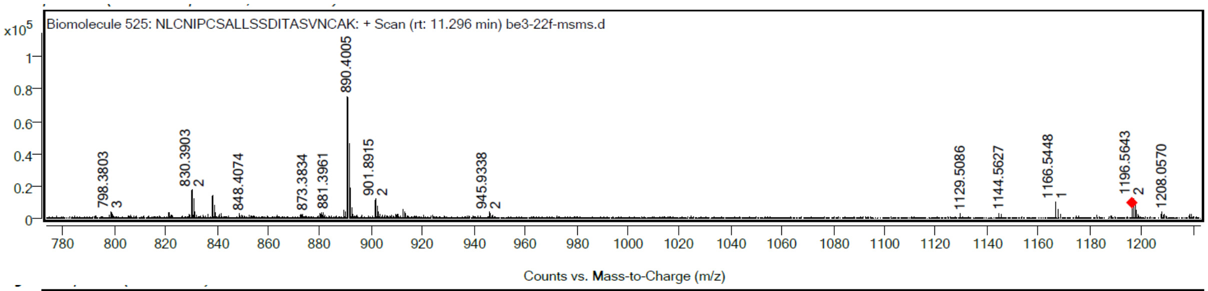

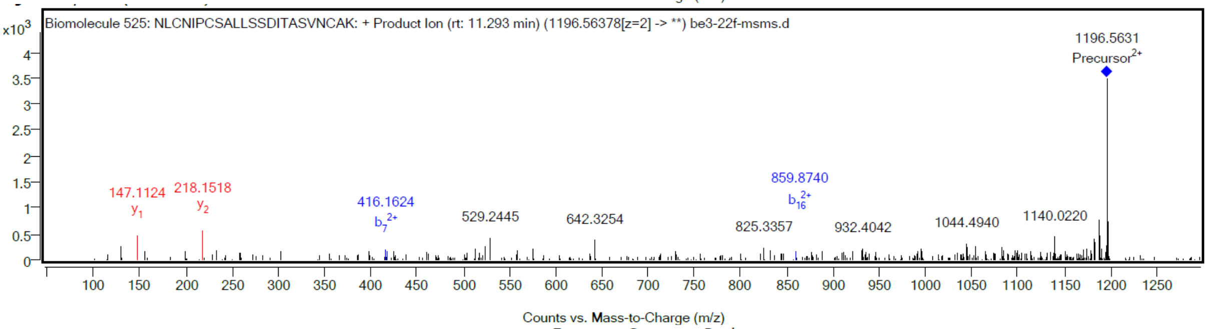

Fragment Spectrum Peaks

m/z	Diff (ppm)	Abund	Ion
147.1124	2.49	458	y1
218.1518	-8.43	555	y2
416.1624	42.45	191	b7
859.8740	41.87	156	b16
1196.5631	6.51	3481	Precursor
1197.0664	5.16	2698	Precursor
1197.5617	10.46	740	Precursor

Boronic Acid Modification of Ribonuclease A

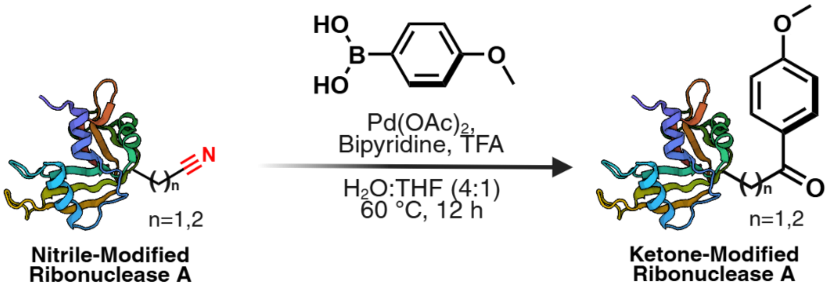

Reaction conducted according to **GP-XVI**. 2 mg of nitrile-modified Ribonuclease A (0.292 mM final concentration) was dissolved in 350 µL of H_2O in a 1/2” dram vial, after which 2 µL of TFA was added. Next, 2 mg of 4-methoxyphenylboronic acid dissolved in 50 µL THF was added to the mixture. N_2 was then bubbled into the solution for 3 minutes using an 18G x 1 ½” needle and balloon. In a separate vial, 1 mg of bipyridine and 1 mg of $Pd(OAc)_2$ were dissolved in 100 µL of 1:1 THF:H_2O and stirred for 15 minutes at room temperature to generate a pre-formed bipyridine-palladium complex. This pre-formed complex was then added to the reaction mixture, and N_2 was bubbled for an additional 2 minutes. The reaction vial was then sealed and stirred for 12 hours at 60 °C. The Pd was quenched by addition of 200 µL 1 M aqueous L-cysteine and 10 µL of 1 M NaOH. The crude reaction mixture was passed through Amicon™ Ultra 3 kDa spin-concentrator and washed with H_2O (7×0.5 mL) to remove the small molecule impurities. The labeled protein was lyophilized, digested using SMART Digest™ Trypsin Kit by Thermo Scientific, and then analyzed using LC-MS/MS. 5 distinct sites were modified with 4-methoxyphenylboronic acid.

MS/MS Analysis of Digested Modified Ribonuclease A

Identified peptide fragment (**2 sites**): NGQTNCYQS: (Sequence: AA 67-75, [N67, Q69])

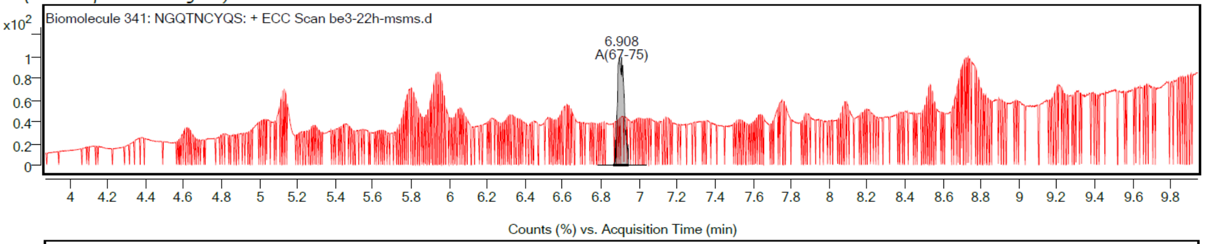

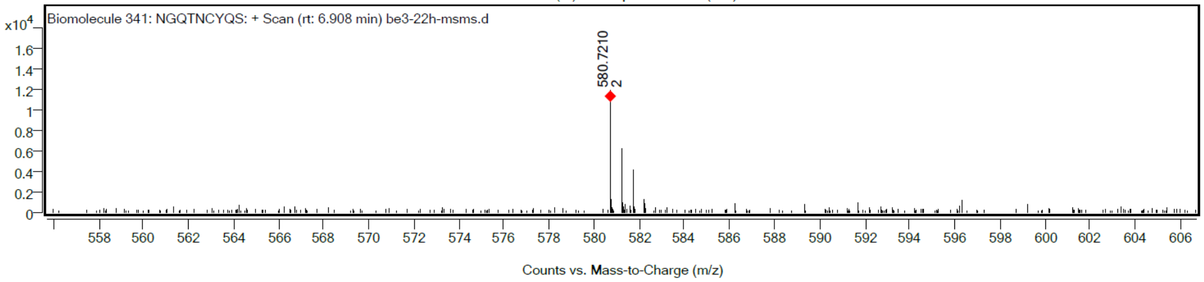

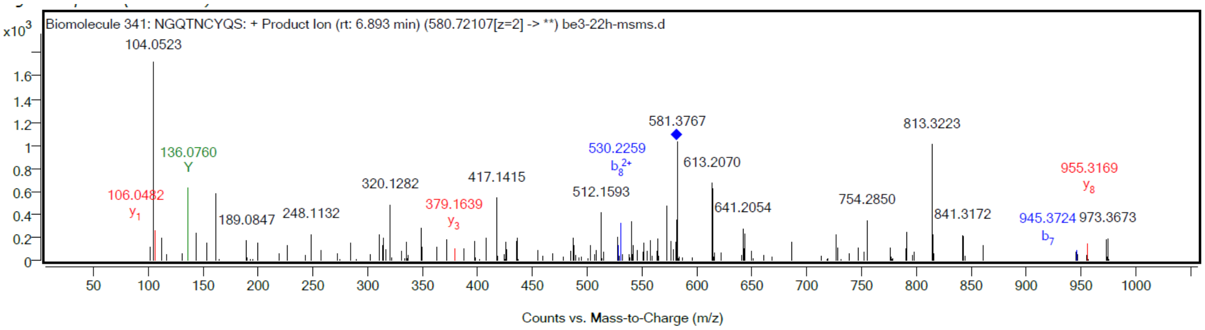

Fragment Spectrum Peaks

m/z	Diff (ppm)	Abund	Ion
106.0482	15.42	255	y1
379.1639	-7.17	103	y3
955.3169	46.61	146	y8
945.3724	-29.27	87	b7
530.2259	-36.10	320	b8
136.0760	-2.47	625	Y
580.7143	12.13	348	Precursor

Identified peptide fragment (**1 site**): ASSSNYCNQMMK: (Sequence: AA 20-31, N24)

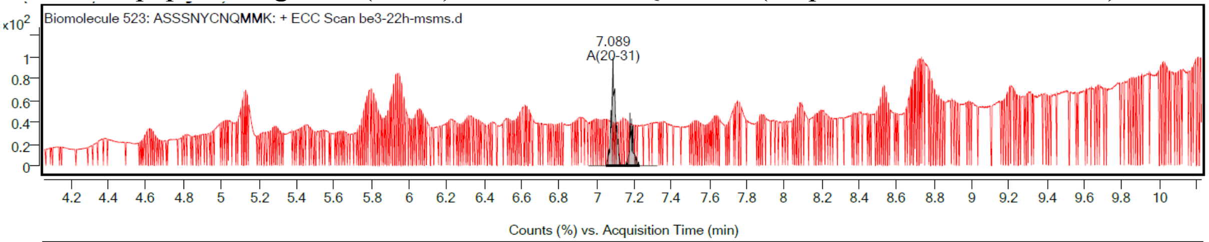

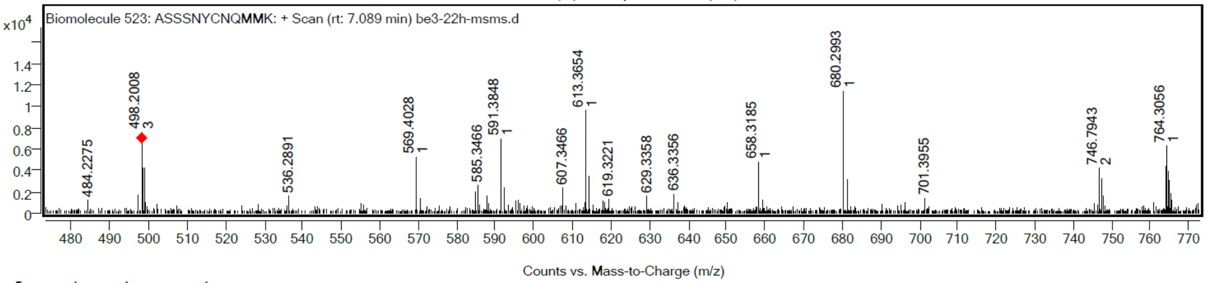

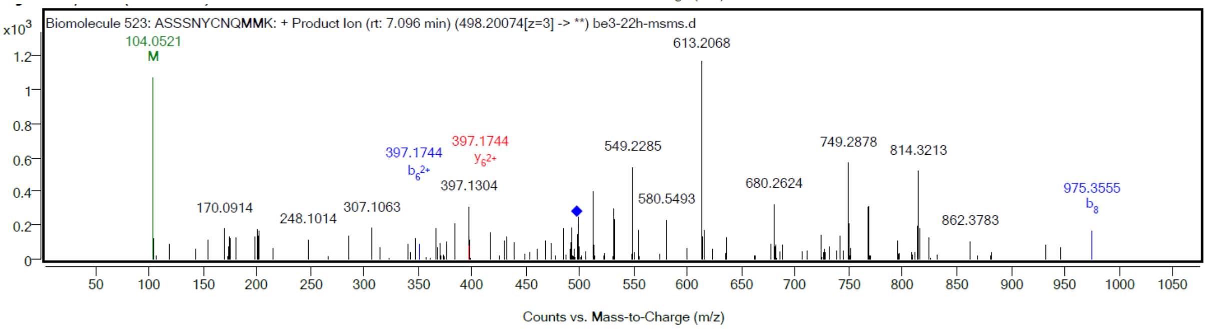

Fragment Spectrum Peaks

m/z	Diff (ppm)	Abund	Ion
397.1744	-32.95	78	y6
975.3555	-4.35	167	b8
351.1507	-23.26	87	b6
104.0521	7.41	1069	M

Identified peptide fragment (**1 site**): YPNCAYKTTQANKH: (Sequence: AA 92-105, N103)

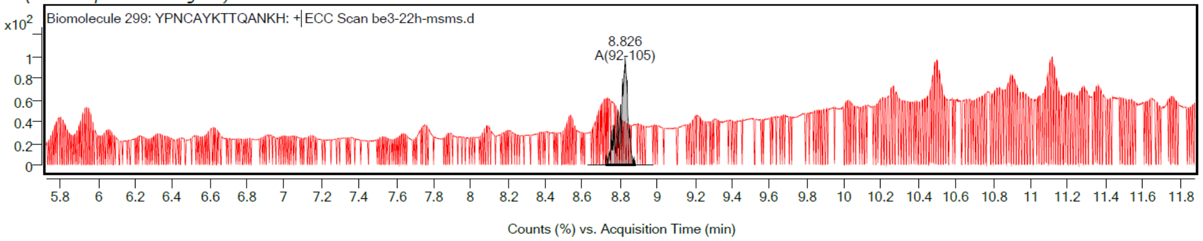

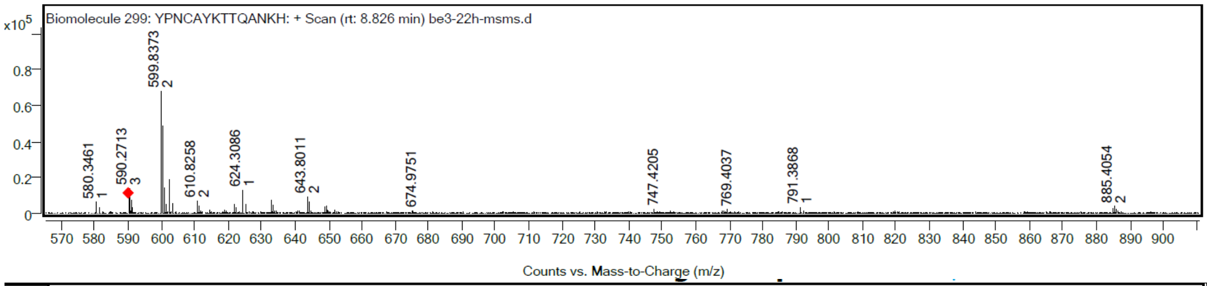

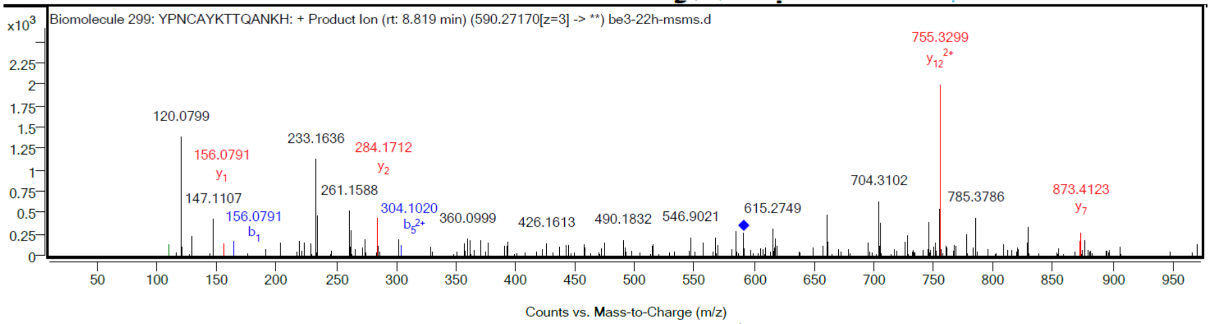

Fragment Spectrum Peaks

m/z	Diff (ppm)	Abund	Ion
156.0791	-14.83	128	y1
284.1712	1.78	427	y2
873.4123	19.62	262	y7
755.3299	17.57	1993	y12
164.0684	13.33	161	b1
304.1020	35.11	114	b5
110.0716	-3.27	120	H

Identified peptide fragment (**2 sites**): KYPNCAYKTTQANK: (Sequence: AA 92-105, [N94, N103])

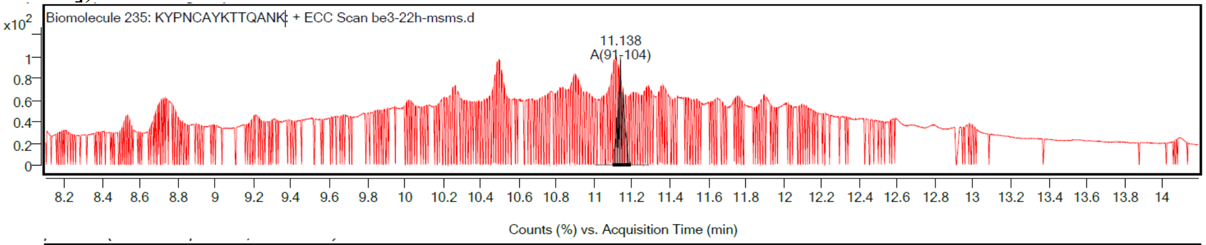

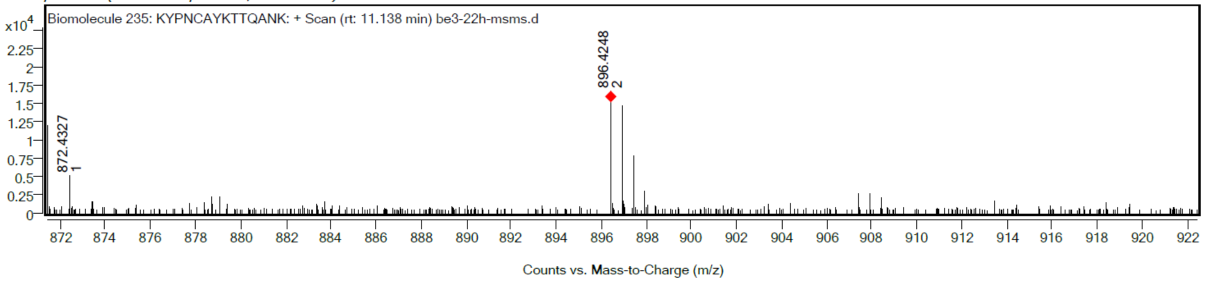

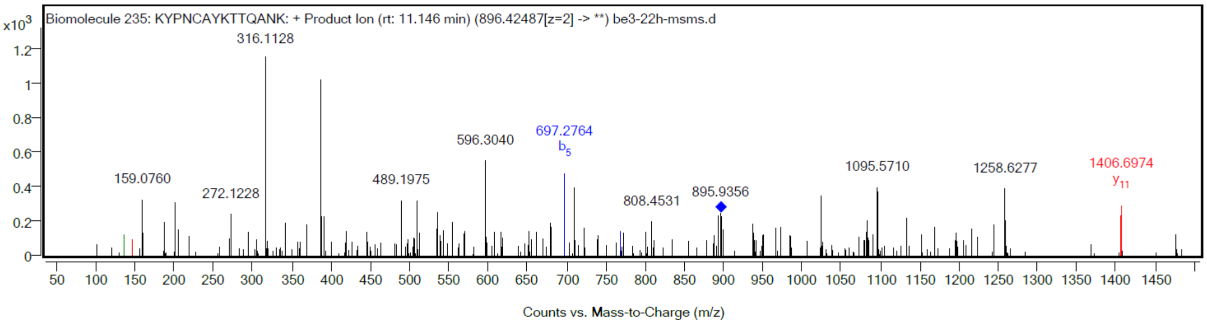

Fragment Spectrum Peaks

m/z	Diff (ppm)	Abund	Ion
147.1121	4.91	91	y1
1406.6974	-34.40	284	y11
697.2764	35.86	473	b5
768.3159	29.39	138	b6
136.0777	-14.47	116	Y

Boronic Acid Modification of Chymotrypsinogen

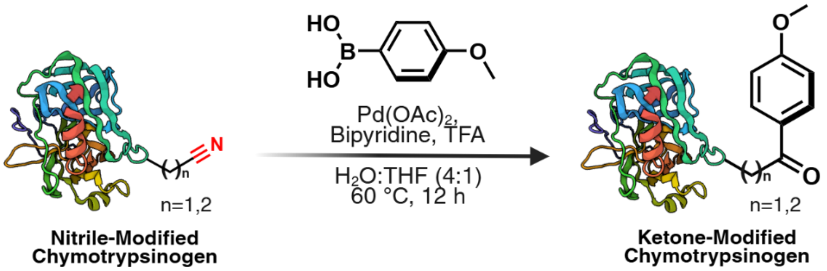

Reaction conducted according to **GP-XVI**. 2 mg of nitrile-modified chymotrypsinogen (0.156 mM final concentration) was dissolved in 350 µL of H_2O in a 1/2” dram vial, after which 2 µL of TFA was added. Next, 2 mg of 4-methoxyphenylboronic acid dissolved in 50 µL THF was added to the mixture. N_2 was then bubbled into the solution for 3 minutes using an 18G x 1 ½” needle and balloon. In a separate vial, 1 mg of bipyridine and 1 mg of $Pd(OAc)_2$ were dissolved in 100 µL of 1:1 THF:H_2O and stirred for 15 minutes at room temperature to generate a pre-formed bipyridine-palladium complex. This pre-formed complex was then added to the reaction mixture, and N_2 was bubbled for an additional 2 minutes. The reaction vial was then sealed and stirred for 12 hours at 60 °C. The Pd was quenched by addition of 200 µL 1 M aqueous L-cysteine and 10 µL of 1 M NaOH. The crude reaction mixture was passed through Amicon™ Ultra 3 kDa spin-concentrator and washed with H_2O (7×0.5 mL) to remove the small molecule impurities. The labeled protein was lyophilized, digested using SMART Digest™ Trypsin Kit by Thermo Scientific, and then analyzed using LC-MS/MS. 4 distinct sites were modified with 4-methoxyphenylboronic acid.

MS/MS Analysis of Digested Modified Chymotrypsinogen

Identified peptide fragment (**1 site**):
TGFHFCGGSLINENWVVTAAHCGVTTSDVVVAGEFDQGSSSEK: (Sequence: AA 37-79, N50)

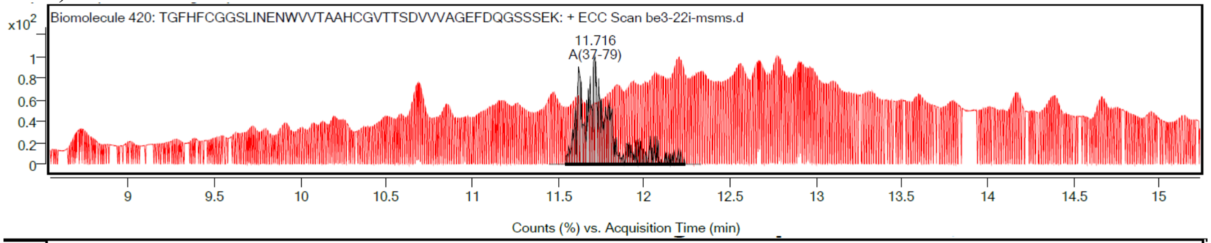

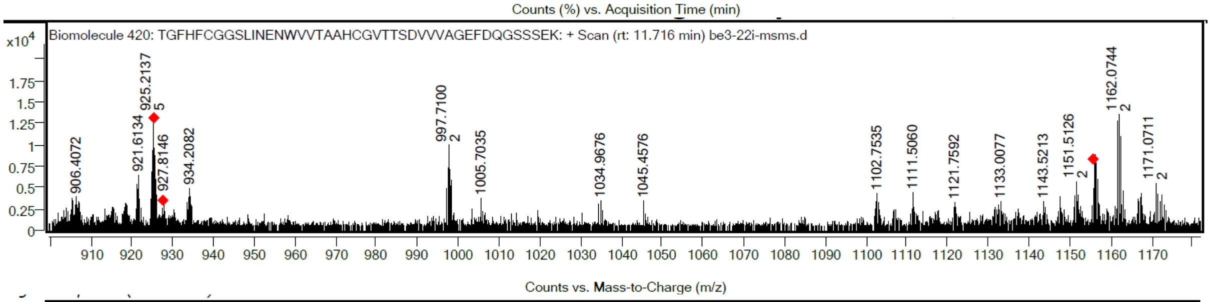

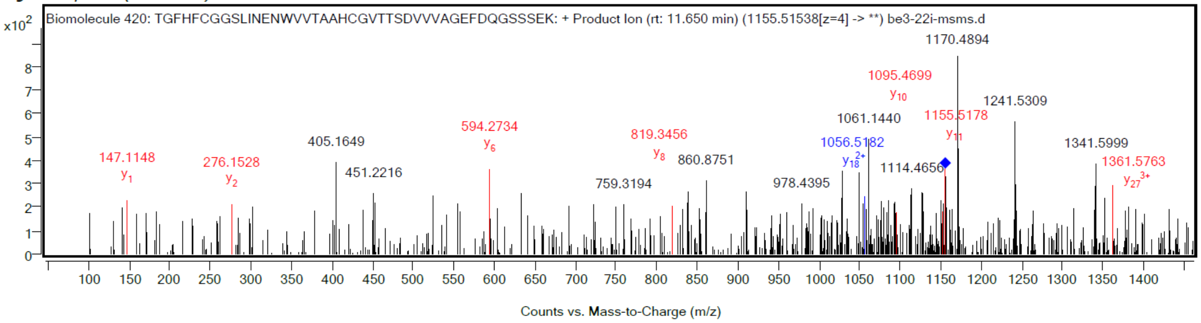

Fragment Spectrum Peaks

m/z	Diff (ppm)	Abund	Ion
147.1148	-13.51	230	y1
276.1528	9.37	211	y2
594.2734	-0.71	361	y6
819.3456	2.78	203	y8
1095.4699	-10.02	177	y10
1155.5178	-23.71	364	y11
1361.5763	32.65	293	y27
1056.5182	-26.80	246	b18

Identified peptide fragment (**1 site**):
STAASFSQTVSAVCLPSASDDFAAGTTCVTTGWGLTR: (Sequence: AA 109-145, Q116)

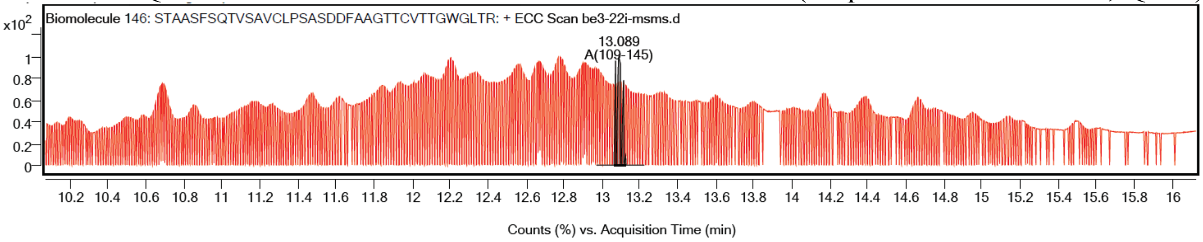

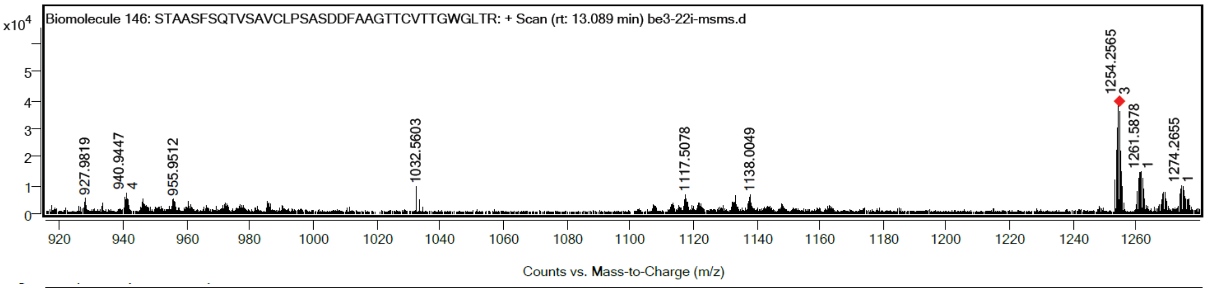

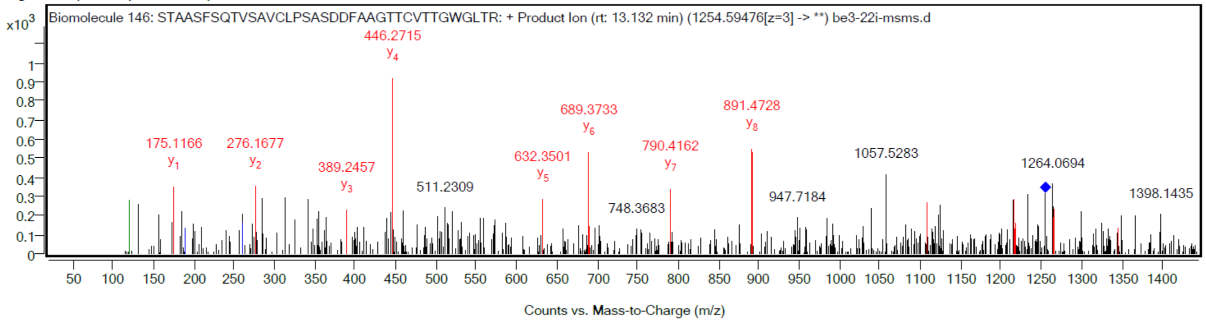

Fragment Spectrum Peaks

m/z	Diff (ppm)	Abund	Ion
175.1166	13.20	350	y1
276.1677	-3.74	351	y2
389.2457	12.78	226	y3
446.2715	1.46	917	y4
632.3501	2.18	284	y5
689.3733	-0.49	530	y6
790.4162	5.62	337	y7
891.4728	-5.02	548	y8
1108.0008	9.99	266	y22
1215.5357	17.39	286	y24
1265.5902	2.01	233	y25
1344.6266	0.52	133	y27
189.0847	12.29	132	b2
260.1260	-7.22	162	b3
120.0808	0.04	281	F

Identified peptide fragment (**2 sites**): LQQASLPLLSNTNC: (Sequence: AA 155-168, [N165, N167])

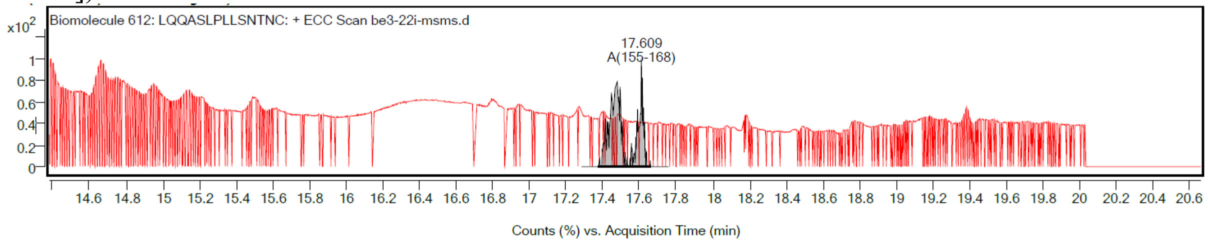

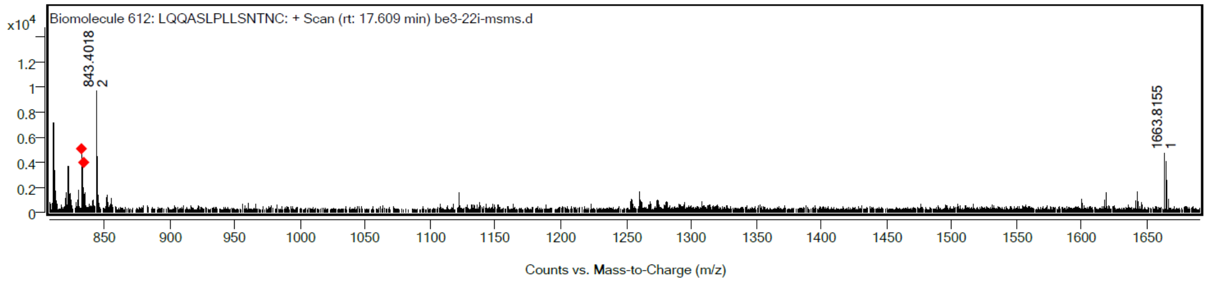

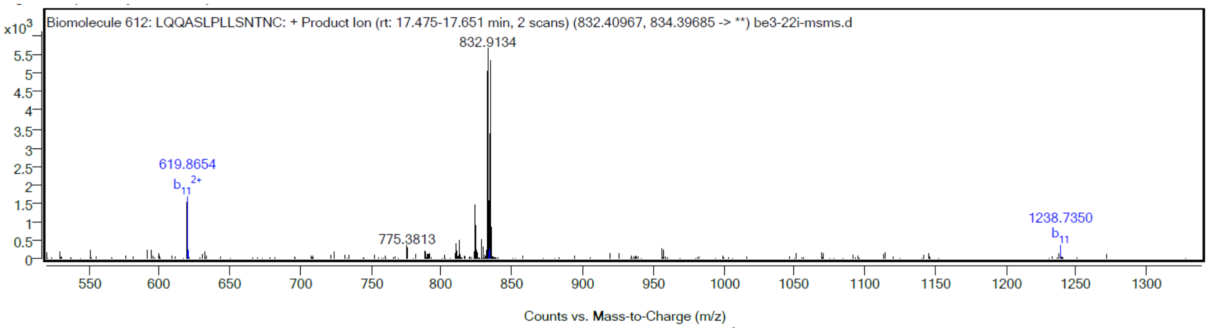

Fragment Spectrum Peaks

m/z	Diff (ppm)	Abund	Ion
1238.7350	-46.07	353	b11
619.8654	-36.83	1666	b11
833.4093	4.72	1568	Precursor

Identified peptide fragment (**1 site**): QQASLPLLSNTNCK: (Sequence: AA 156-169, N165)

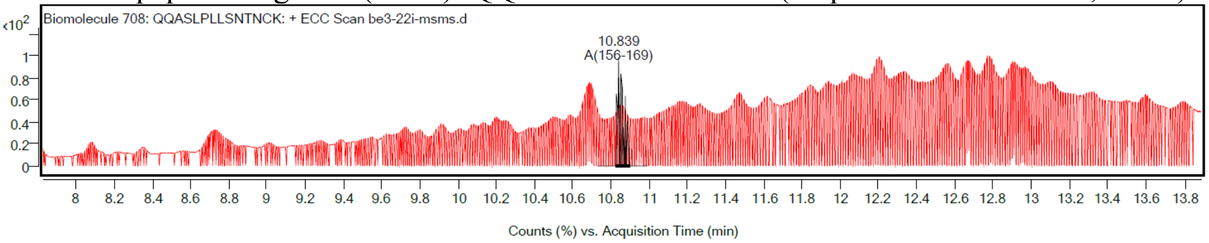

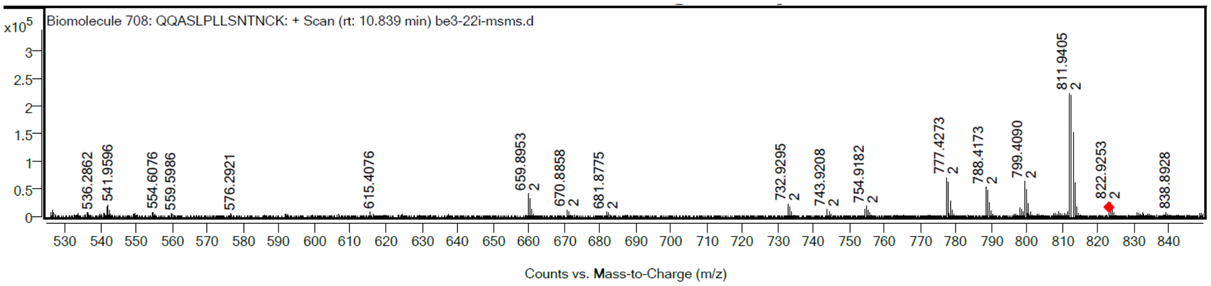

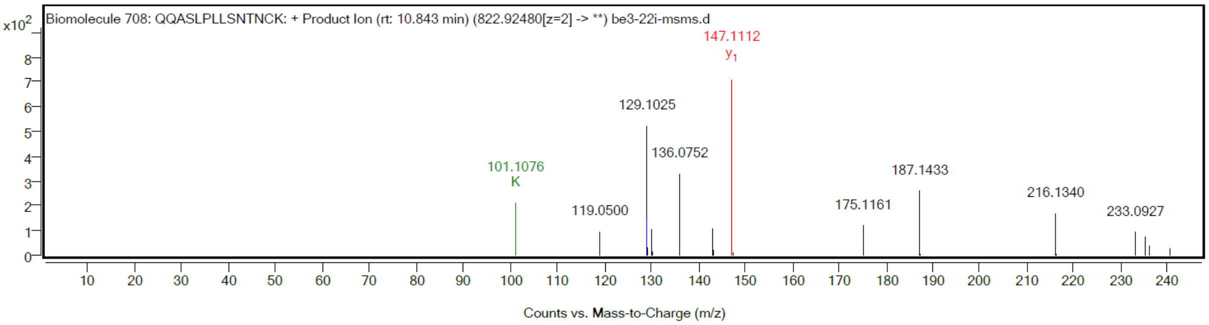

Fragment Spectrum Peaks

m/z	Diff (ppm)	Abund	Ion
147.1112	11.20	708	y1
129.0660	-0.89	151	b1
101.1076	-2.98	211	K

Identified peptide fragment (**1 site**): PLLSNTNCKK: (Sequence: AA 156-169, N165)

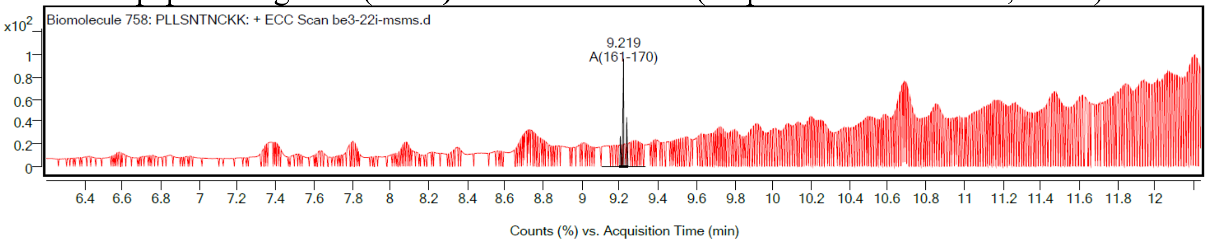

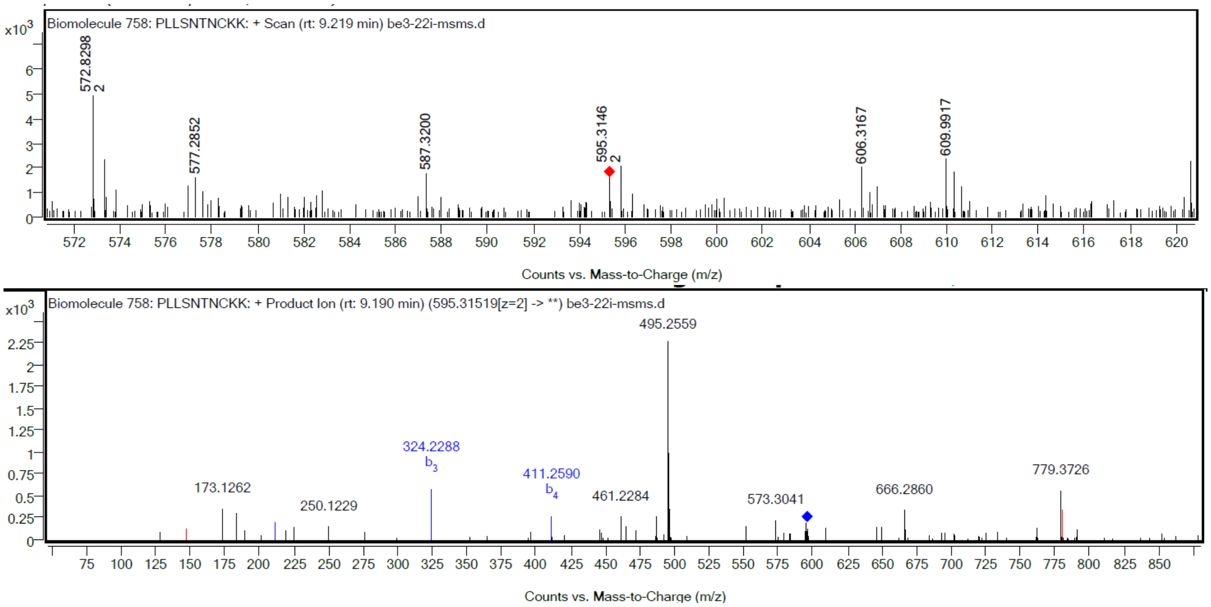

Fragment Spectrum Peaks

m/z	Diff (ppm)	Abund	Ion
147.1126	1.52	119	y1
780.3647	7.93	332	y6
211.1427	6.89	191	b2
324.2288	-2.03	573	b3
411.2590	3.00	261	b4

Boronic Acid Modification of Creatine Kinase

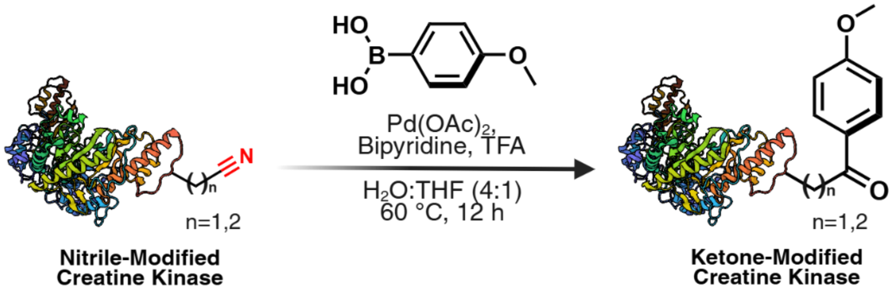

Reaction conducted according to **GP-XVI**. 2 mg of nitrile-modified creatine kinase (93.0 µM final concentration) was dissolved in 350 µL of H_2O in a 1/2” dram vial, after which 2 µL of TFA was added. Next, 2 mg of 4-methoxyphenylboronic acid dissolved in 50 µL THF was added to the mixture. N_2 was then bubbled into the solution for 3 minutes using an 18G x 1 ½” needle and balloon. In a separate vial, 1 mg of bipyridine and 1 mg of $Pd(OAc)_2$ were dissolved in 100 µL of 1:1 THF:H_2O and stirred for 15 minutes at room temperature to generate a pre-formed bipyridine-palladium complex. This pre-formed complex was then added to the reaction mixture, and N_2 was bubbled for an additional 2 minutes. The reaction vial was then sealed and stirred for 12 hours at 60 °C. The Pd was quenched by addition of 200 µL 1 M aqueous L-cysteine and 10 µL of 1 M NaOH. The crude reaction mixture was passed through Amicon™ Ultra 3 kDa spin-concentrator and washed with H_2O (7×0.5 mL) to remove the small molecule impurities. The labeled protein was lyophilized, digested using SMART Digest™ Trypsin Kit by Thermo Scientific, and then analyzed using LC-MS/MS. 6 distinct sites were modified with 4-methoxyphenylboronic acid.

MS/MS Analysis of Digested Modified Creatine Kinase

Identified peptide fragment (**1 site**): GNTHNKFKLNYK: (Sequence: AA 4-15, N5)

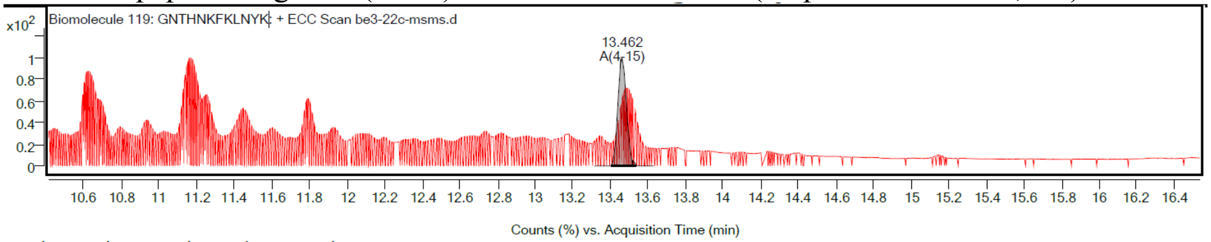

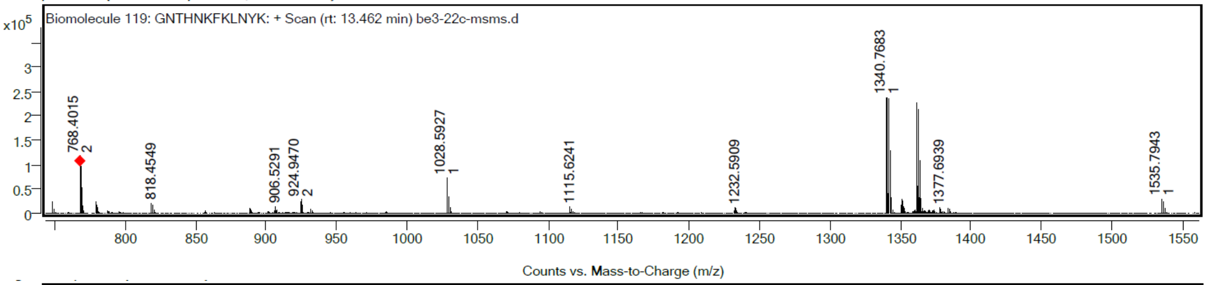

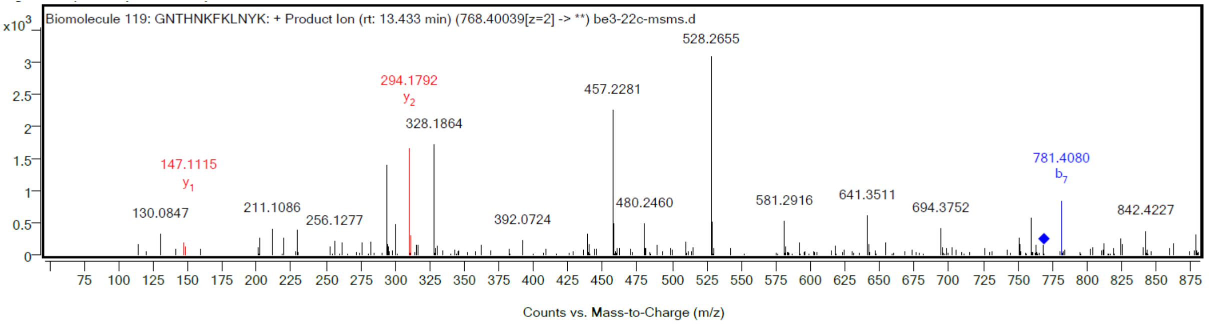

Fragment Spectrum Peaks

m/z	Diff (ppm)	Abund	Ion
147.1115	8.63	185	y1
310.1754	2.48	1665	y2
781.4080	-43.54	837	b7

Identified peptide fragment (**1 site**):
ETPSGFTVDDVIQTGVDNPGHPFIMTVGCVAGDEESYEVFK: (Sequence: AA 46-86, N63)

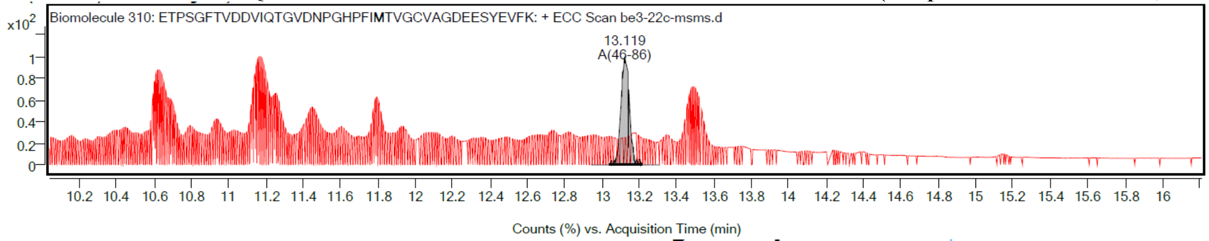

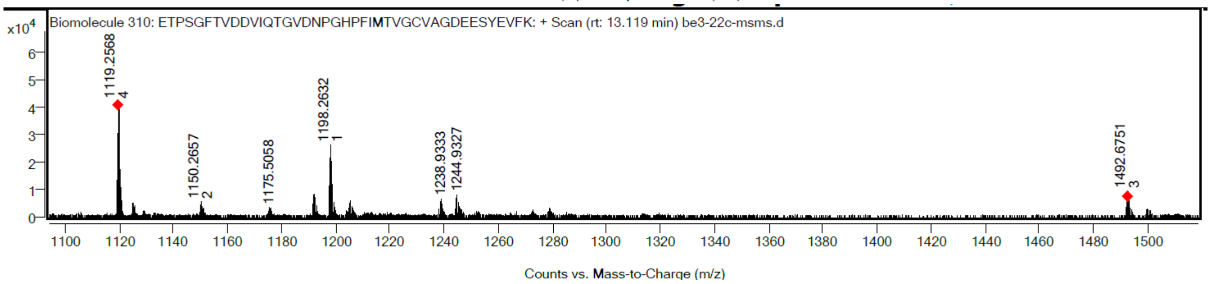

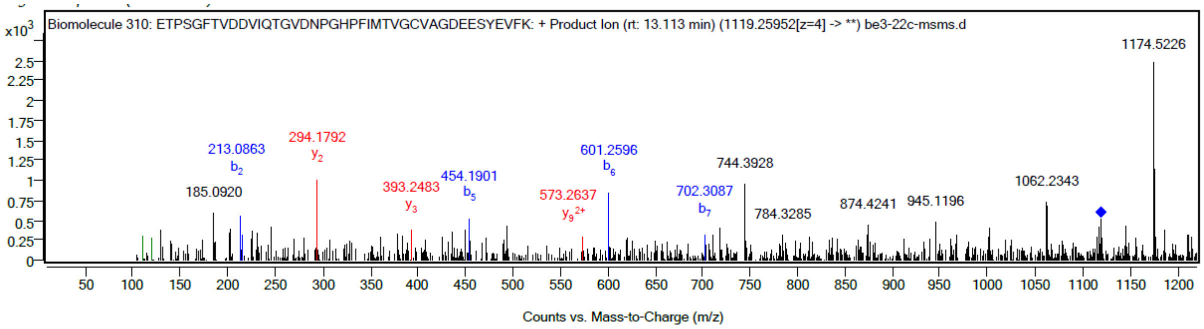

Fragment Spectrum Peaks

m/z	Diff (ppm)	Abund	Ion
294.1792	6.95	1007	y2
393.2483	3.40	370	y3
573.2637	-17.81	283	y9
213.0863	3.01	552	b2
454.1901	6.92	516	b5
601.2596	3.36	838	b6
702.3087	0.96	310	b7
110.0699	12.16	307	H
120.0806	1.09	275	F
1119.7467	12.48	434	Precursor
1120.2653	-2.59	270	Precursor

Identified peptide fragment (**2 sites**): TEKEQQQLIDDHFLFDKPVSPLLLASGMAR: (Sequence: AA 180-209, [Q184, Q185])

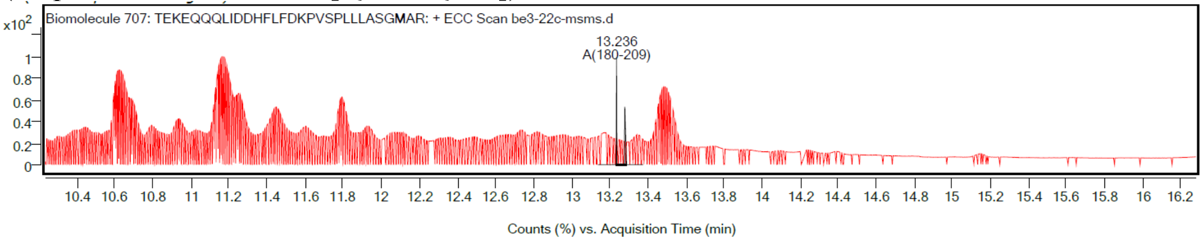

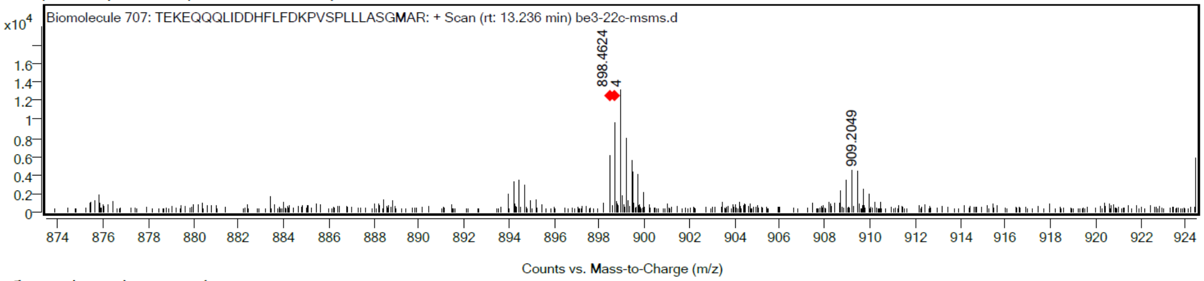

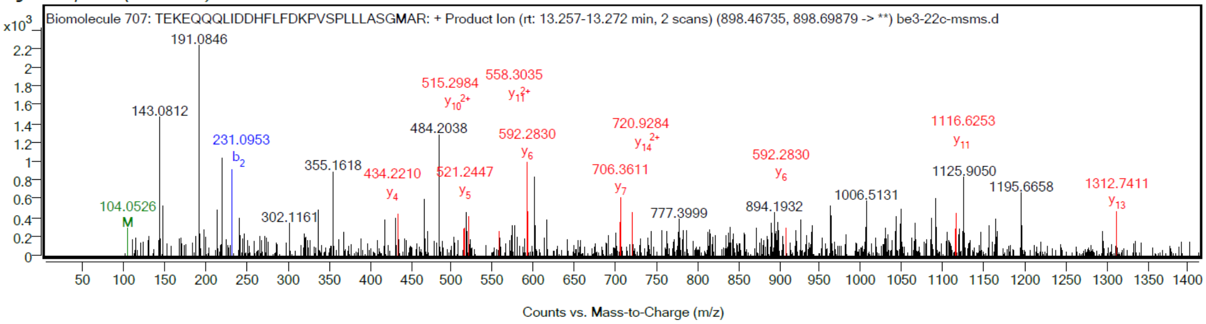

m/z	Diff (ppm)	Abund	Ion
434.2210	-6.80	437	y4
521.2447	10.31	408	y5
592.2830	7.10	988	y6
706.3611	19.03	610	y7
1116.6253	1.92	448	y11
1312.7411	5.76	465	y13
515.2984	5.83	289	y10
558.3035	21.87	254	y11
720.9284	-4.11	449	y14
908.0213	-8.56	283	y17
231.0953	9.72	912	b2
104.0526	2.22	288	M

Fragment Spectrum Peaks

Identified peptide fragment (**1 site**): TEKEQQQLIDDHFLFDKPVSPLLLASGMAR: (Sequence: AA 210-233, N222)

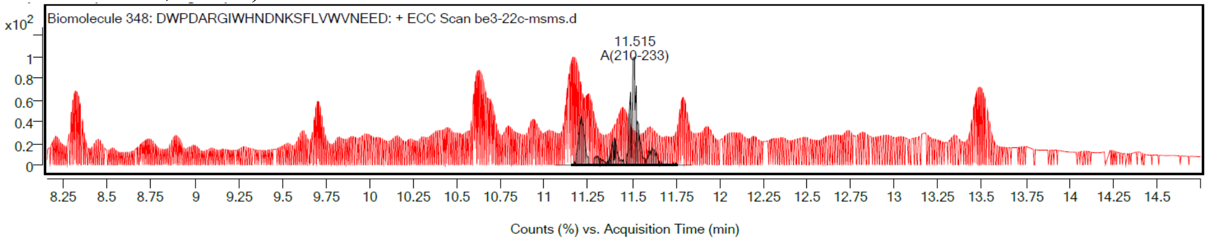

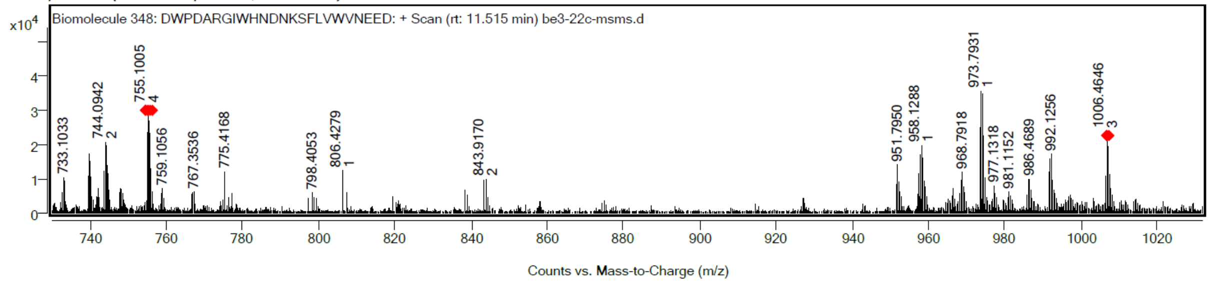

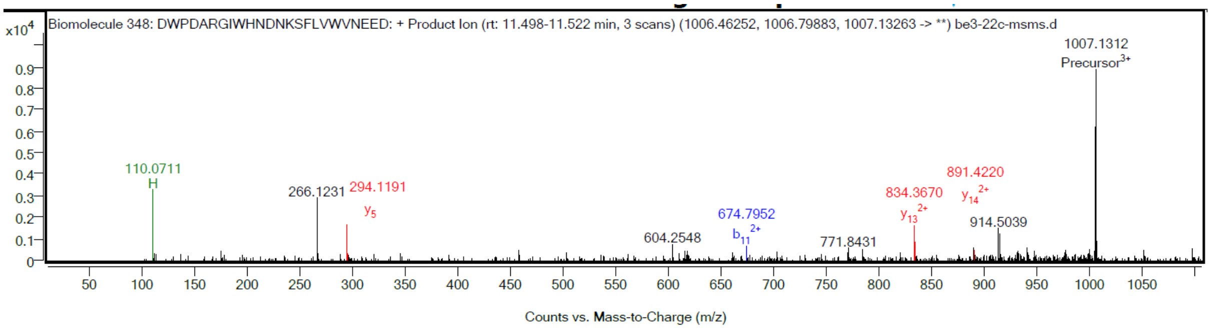

Fragment Spectrum Peaks

m/z	Diff (ppm)	Abund	Ion
294.1191	-0.22	1621	y5
834.3670	19.21	1602	y13
891.4220	-19.69	439	y14
674.7952	25.94	625	b11
110.0711	1.75	3268	H
1006.1162	12.88	391	Precursor
1006.4604	3.26	3228	Precursor
1006.7959	2.19	6187	Precursor
1007.1312	1.33	8891	Precursor
1007.4612	5.73	3541	Precursor

Identified peptide fragment (**1 site**): GHPFMWNQHLGYVLTCPSNLGTGLR: (Sequence: AA 268-292, Q275)

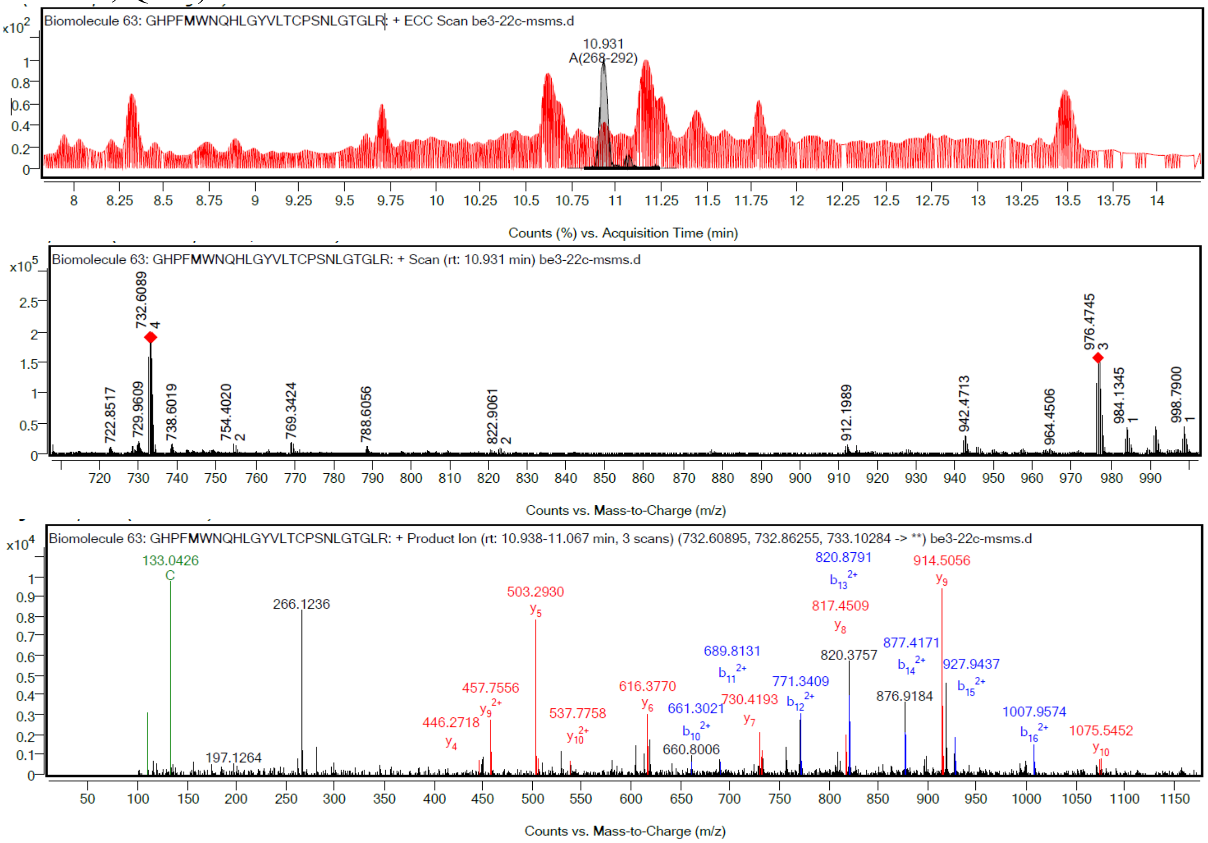

Fragment Spectrum Peaks

m/z	Diff (ppm)	Abund	Ion
446.2718	0.75	688	y4
503.2930	1.31	7819	y5
616.3770	1.14	3035	y6
730.4193	1.80	2115	y7
817.4509	2.18	1962	y8
914.5056	-0.24	9377	y9
1075.5452	-5.35	791	y10
457.7556	1.61	2716	y9
537.7758	-7.67	640	y10
661.3021	-2.65	604	b10
689.8131	-2.80	603	b11
771.3409	2.40	3049	b12
820.8791	-2.51	3991	b13
877.4171	2.23	2095	b14
927.9437	-0.94	1846	b15
1007.9574	0.77	1481	b16
110.0714	-0.87	3091	H
133.0426	2.84	9746	C

Boronic Acid Modification of Carbonic Anhydrase

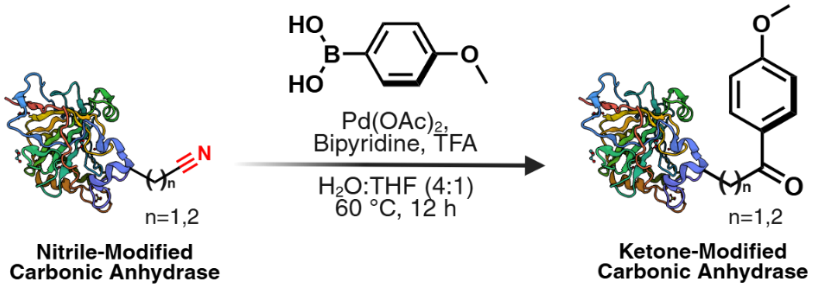

Reaction conducted according to **GP-XVI**. 2 mg of nitrile-modified carbonic anhydrase (0.139 mM final concentration) was dissolved in 350 µL of H_2O in a 1/2" dram vial, after which 2 µL of TFA was added. Next, 2 mg of 4-methoxyphenylboronic acid dissolved in 50 µL THF was added to the mixture. N_2 was then bubbled into the solution for 3 minutes using an 18G x 1 ½" needle and balloon. In a separate vial, 1 mg of bipyridine and 1 mg of $Pd(OAc)_2$ were dissolved in 100 µL of 1:1 THF:H_2O and stirred for 15 minutes at room temperature to generate a pre-formed bipyridine-palladium complex. This pre-formed complex was then added to the reaction mixture, and N_2 was bubbled for an additional 2 minutes. The reaction vial was then sealed and stirred for 12 hours at 60 °C. The Pd was quenched by addition of 200 µL 1 M aqueous L-cysteine and 10 µL of 1 M NaOH. The crude reaction mixture was passed through Amicon™ Ultra 3 kDa spin-concentrator and washed with H_2O (7×0.5 mL) to remove the small molecule impurities. The labeled protein was lyophilized, digested using SMART Digest™ Trypsin Kit by Thermo Scientific, and then analyzed using LC-MS/MS. 9 distinct sites were modified with 4-methoxyphenylboronic acid.

MS/MS Analysis of Digested Modified Carbonic Anhydrase

Identified peptide fragment (**1 site**): DFPIANGER: (Sequence: AA 57-73, N66)

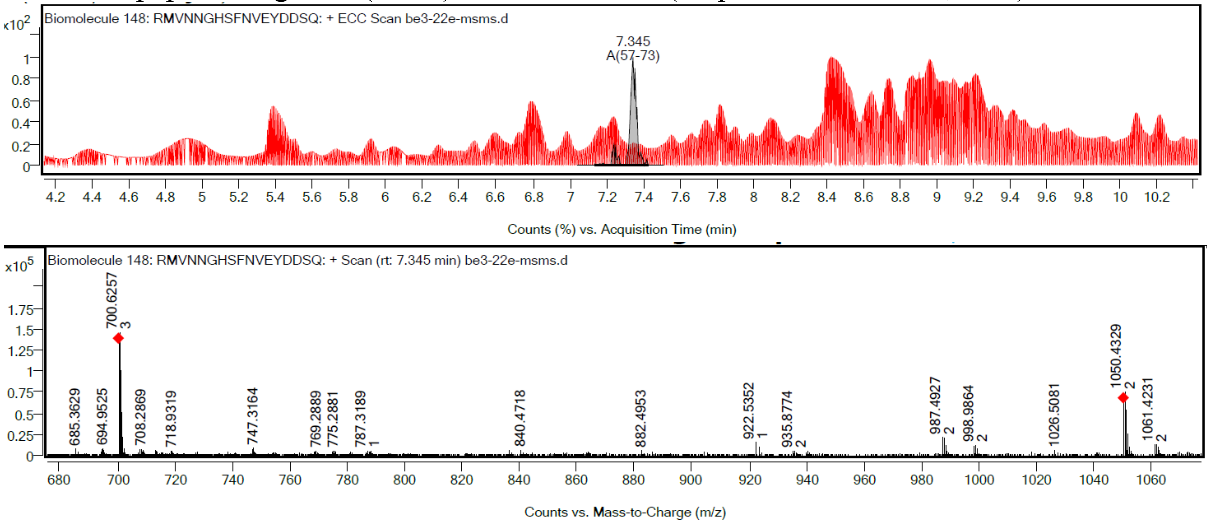

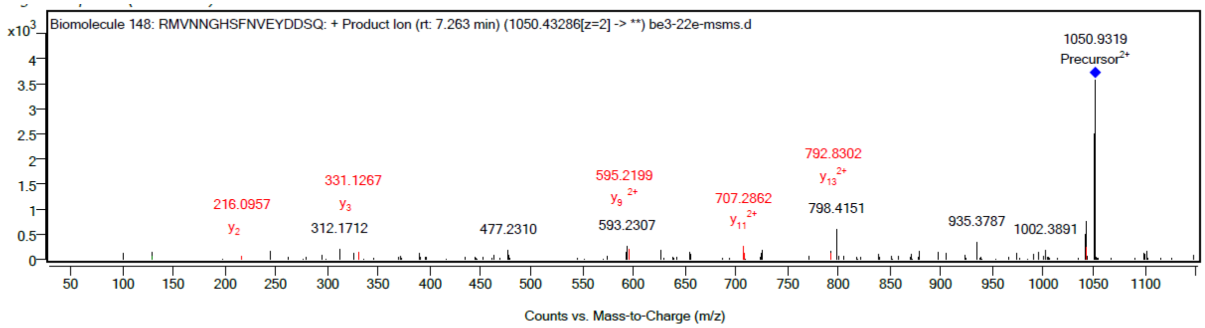

Fragment Spectrum Peaks

m/z	Diff (ppm)	Abund	Ion
216.0957	10.29	53	y2
331.1267	-5.52	145	y3
1042.4223	-21.38	232	y8
595.2199	30.19	191	y9
707.2862	-4.10	263	y11
792.8302	-18.57	123	y13
129.1114	15.89	54	R
1050.9319	7.20	3587	Precursor
1051.4295	11.03	952	Precursor

Identified peptide fragment (**1 site**): TLNFNAEGEPELLMLA: (Sequence: AA 226-241, N228)

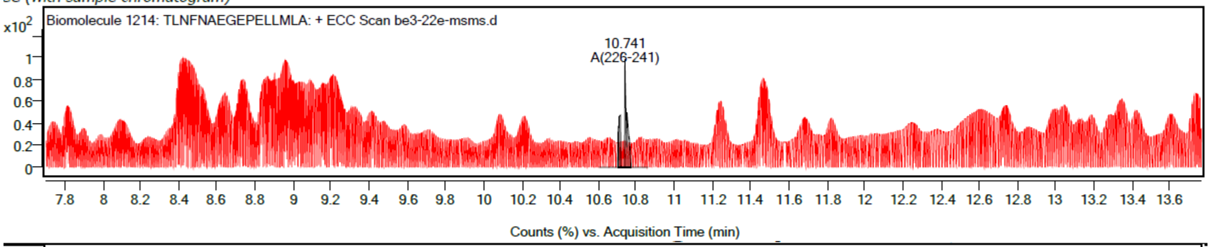

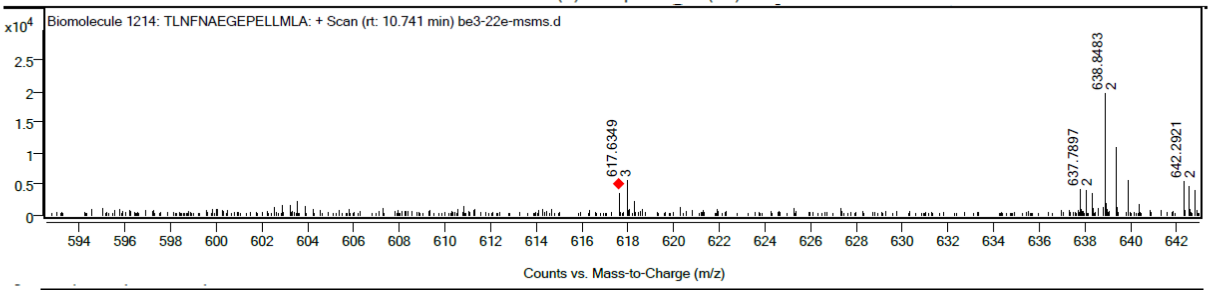

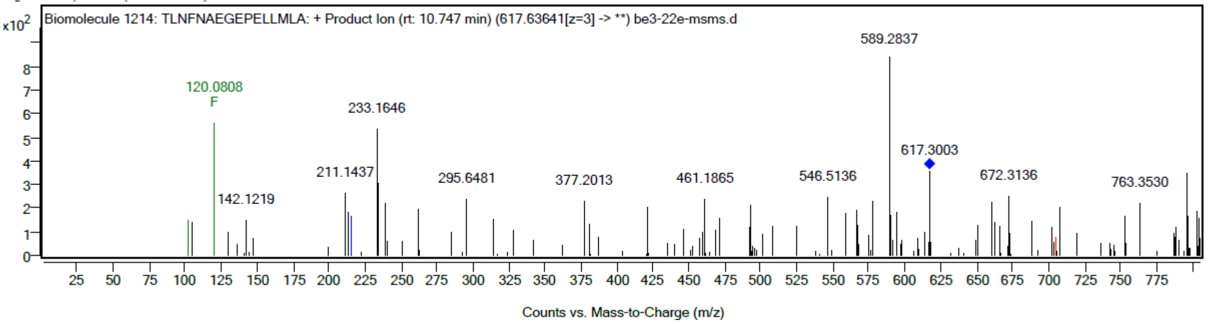

Fragment Spectrum Peaks

m/z	Diff (ppm)	Abund	Ion
705.3774	10.97	77	y6
215.1404	-6.40	164	b2
102.0562	-12.27	148	E
120.0808	-0.61	560	F

Identified peptide fragment (**1 site**): PELLMLANWRPAQPLKNR: (Sequence: AA 235-252, N242)

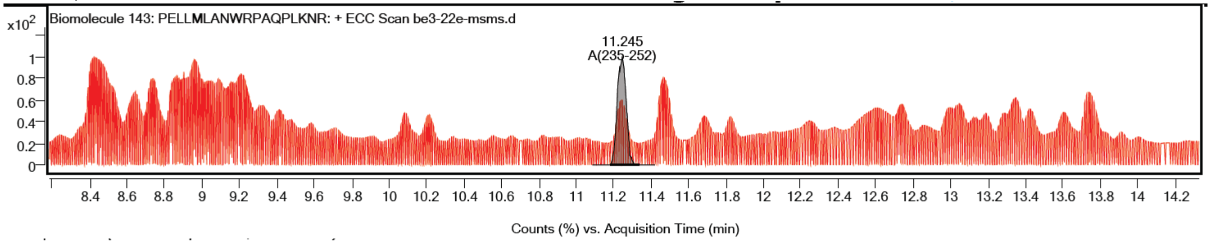

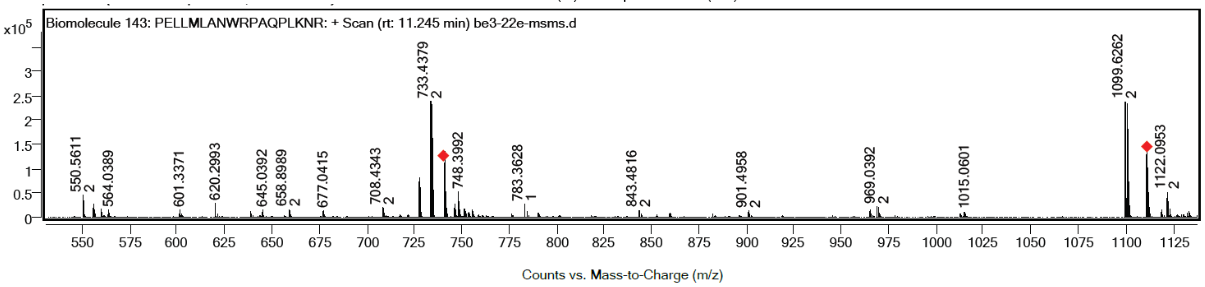

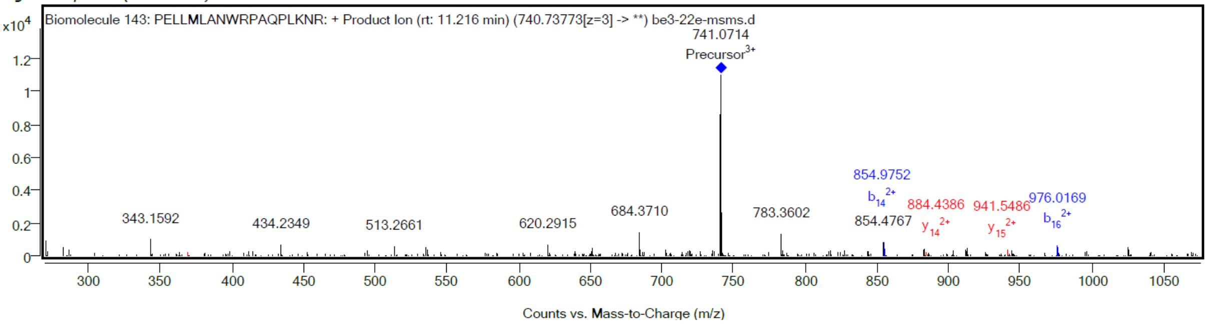

Fragment Spectrum Peaks

m/z	Diff (ppm)	Abund	Ion
369.2168	20.81	233	y6
884.4386	43.93	192	y14
941.5486	-29.20	347	y15
854.9752	-32.27	792	b14
976.0169	22.48	599	b16
740.7357	8.37	8593	Precursor
741.0714	6.66	11007	Precursor
741.4039	9.21	6991	Precursor
741.7357	12.81	2612	Precursor

Identified peptide fragment (**3 sites**): EPISVSSQQMLKFRTLNFNAEGEPELLMLANWRPAQPLK: (Sequence: AA 212-250, [Q219, Q220, N228])

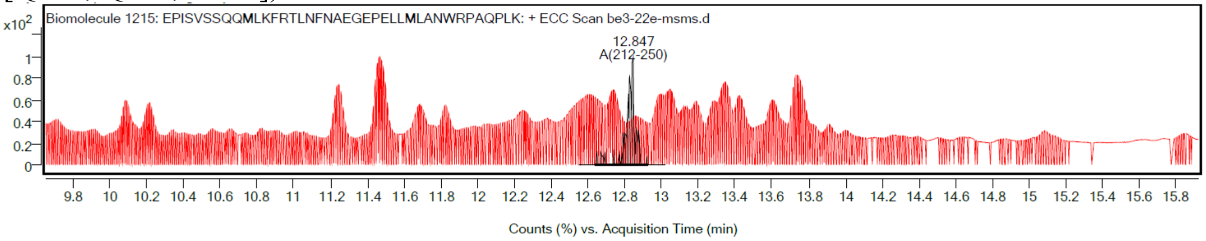

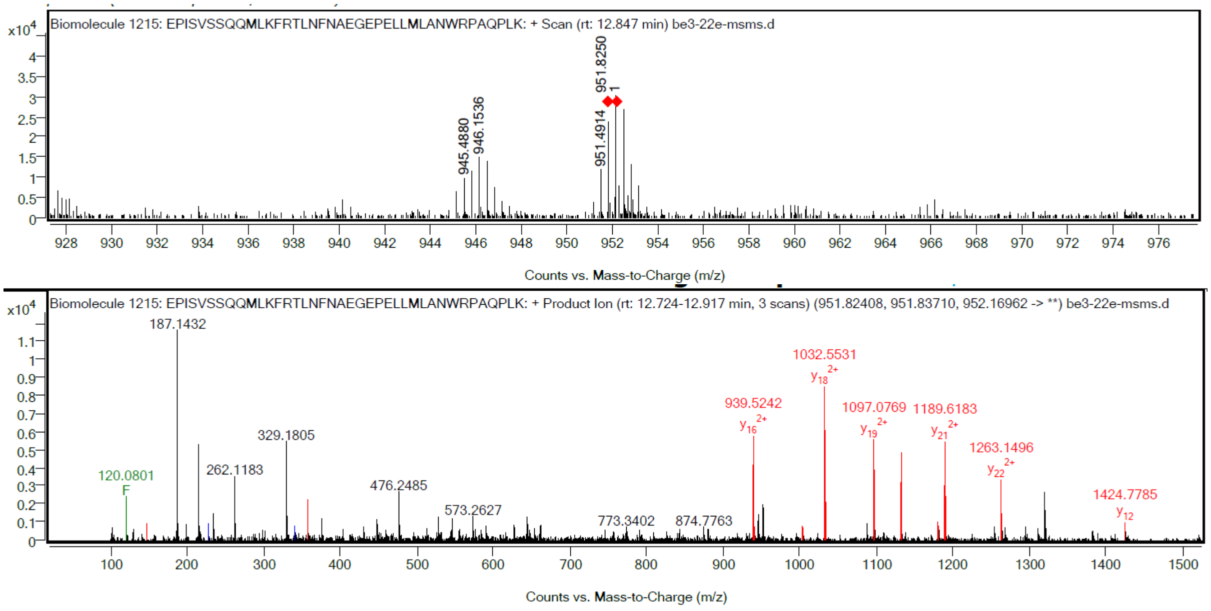

Fragment Spectrum Peaks

m/z	Diff (ppm)	Abund	Ion
147.1110	12.10	894	y1
357.2497	-0.25	2218	y3
1180.6568	1.51	1008	y10
1424.7785	3.21	961	y12
939.5242	4.67	5759	y16
1003.5489	-0.63	757	y17
1032.5531	7.34	8474	y18
1097.0769	4.57	5546	y19
1132.5962	3.75	4820	y20
1189.6183	3.10	5439	y21
1263.1496	5.17	3319	y22
227.1009	7.76	909	b2
340.1850	4.96	761	b3
1190.6156	8.16	2425	b18
120.0801	5.55	2391	F
952.5001	-9.50	1783	Precursor

Identified peptide fragment (**3 sites**): YGDFGTAAQQPDGLAVVGVFLKVGDAN: (Sequence: AA 126-152, [Q134, Q135, N152])

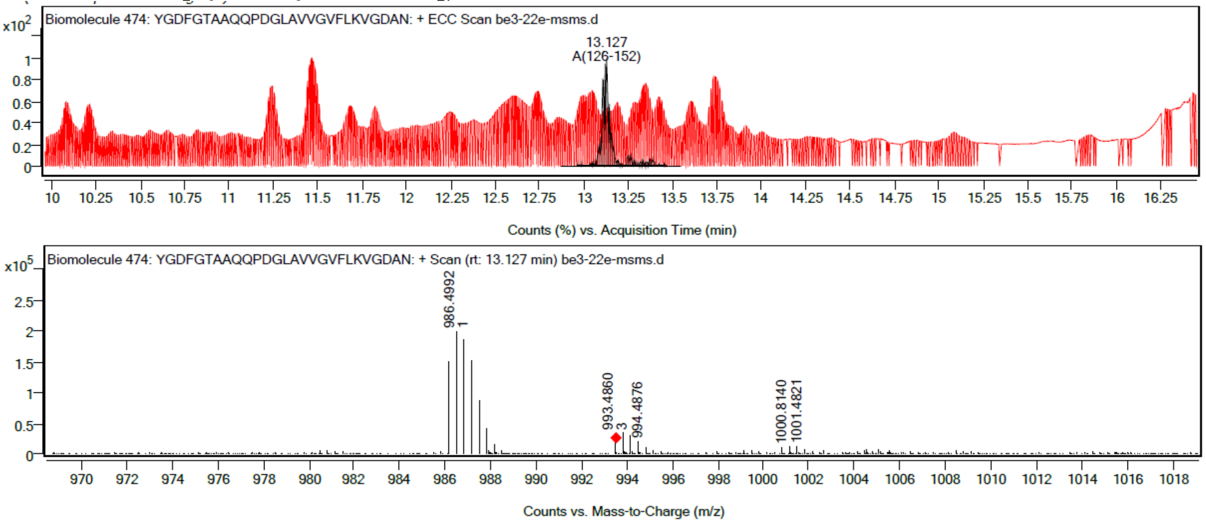

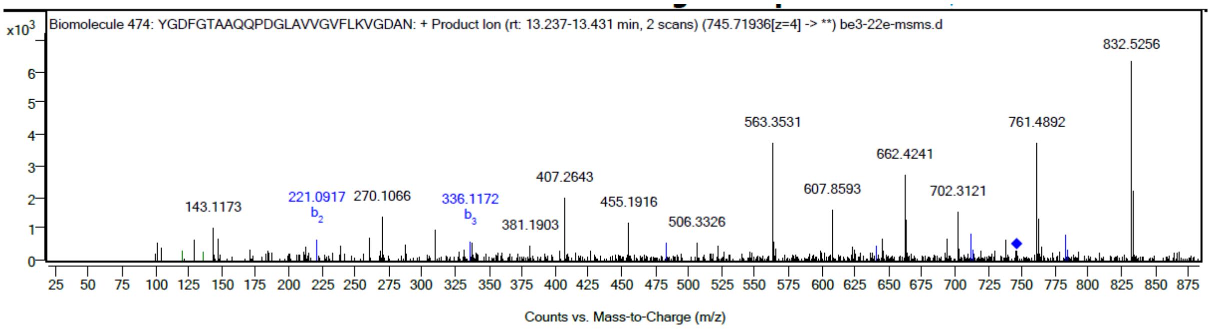

Fragment Spectrum Peaks

m/z	Diff (ppm)	Abund	Ion
221.0917	1.63	621	b2
336.1172	5.27	563	b3
483.1880	-1.24	541	b4
641.2527	6.10	444	b6
712.2925	1.73	816	b7
783.3301	0.94	770	b8
120.0811	-2.37	276	F
136.0775	-13.07	254	Y

Identified peptide fragment (**1 site**): KYAAELHLVHWNTKYGDFGTAAQQP: (Sequence: AA 112-136, Q135)

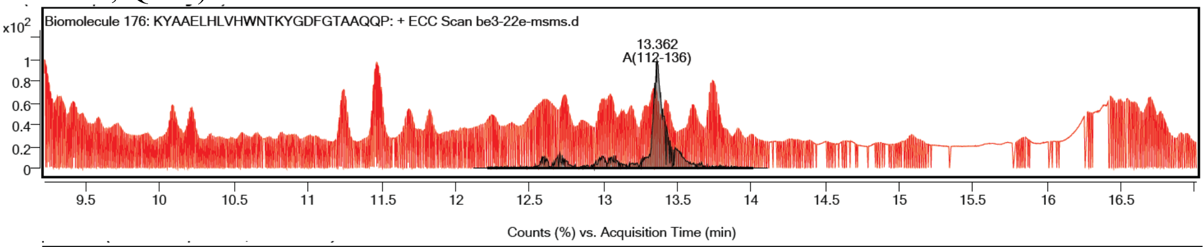

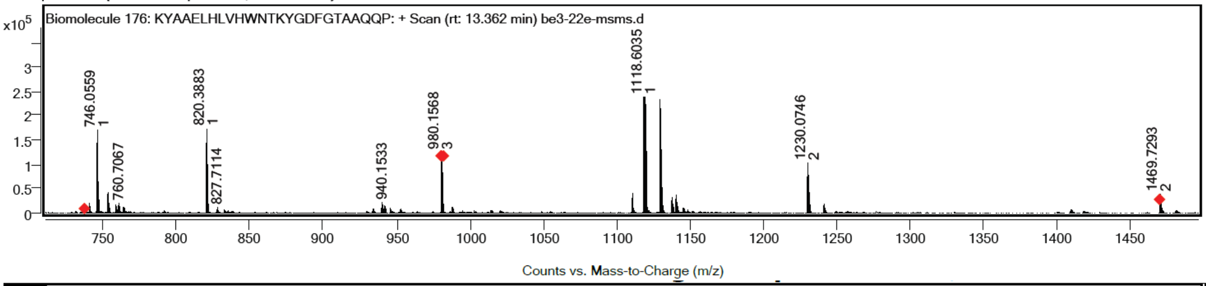

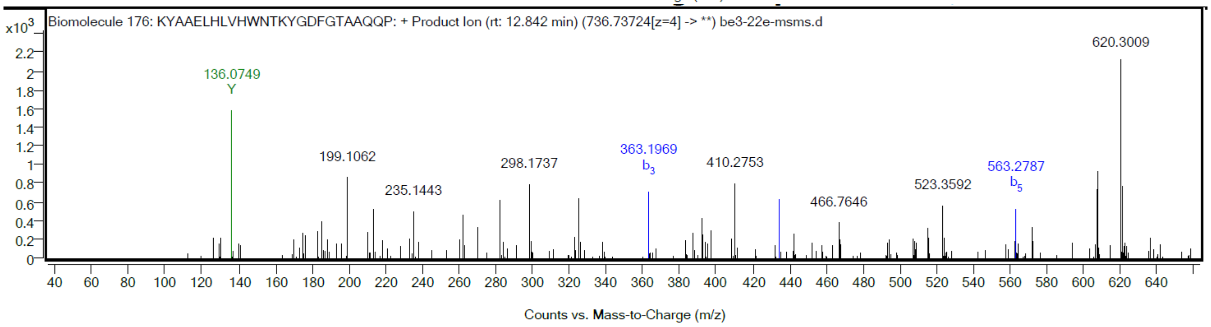

Fragment Spectrum Peaks

m/z	Diff (ppm)	Abund	Ion
363.1969	15.79	700	b3
434.2396	0.34	622	b4
563.2787	6.62	519	b5
136.0749	6.13	1578	Y

Identified peptide fragment (**1 site**): LNFNAEGEPELLMLANWRPAQPLK: (Sequence: AA 227-250, Q247)

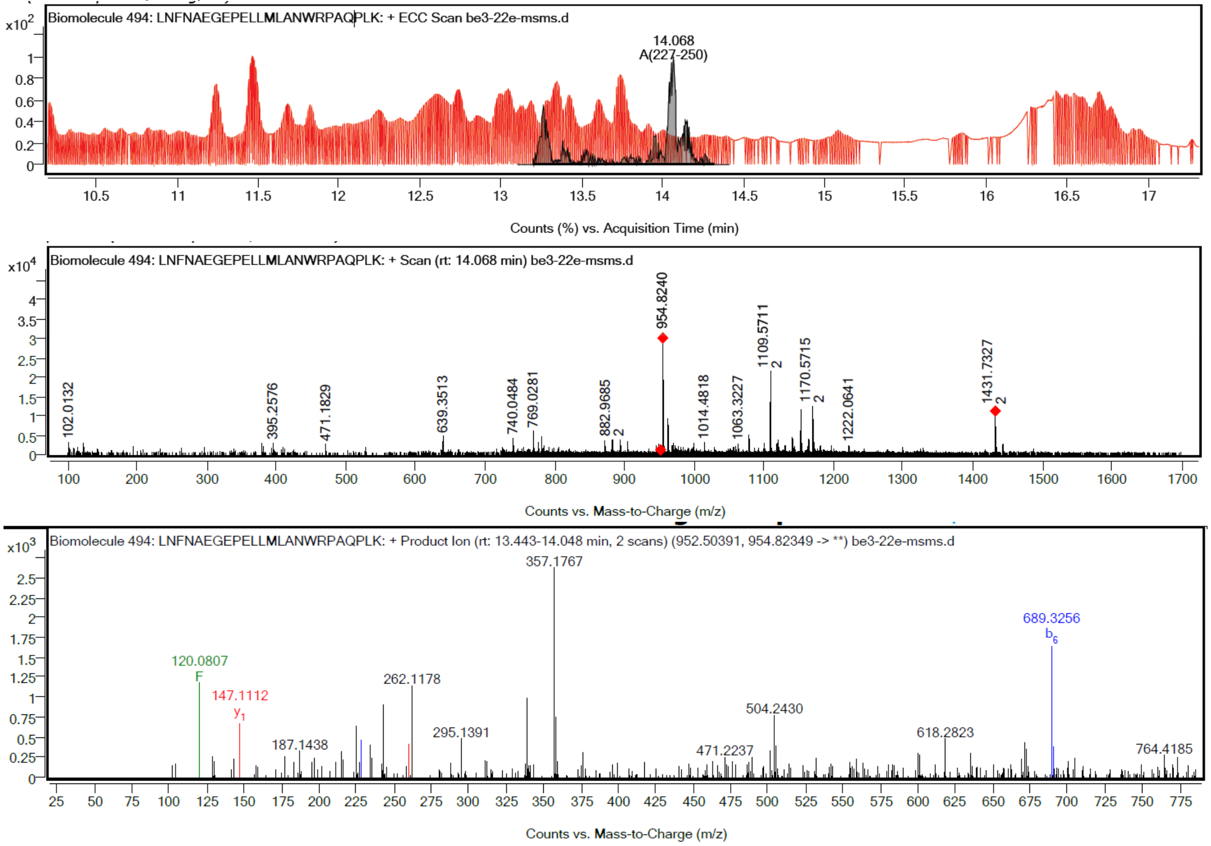

Fragment Spectrum Peaks

m/z	Diff (ppm)	Abund	Ion
147.1112	11.03	668	y1
260.1974	-1.91	412	y2
228.1329	5.84	465	b2
689.3256	-0.38	1643	b6
120.0807	0.74	1184	F

Supplementary Fig. 22b: Analysis of Ketone-Modified Sites

Microenvironment Analysis was conducted by hand through analyzing PDBs. The observations are tabulated below.

Protein_ID	Site_in_protein	Structures (PDB)	Notes
P61823	N24	**1A2W**	Alpha helix, extremely solvent exposed, no hydrogen bonding
P61823	N67		Alpha helix, extremely solvent exposed, hydrogen bonding with backbone
P61823	Q71		Alpha helix, extremely solvent exposed, hydrogen bonding with backbone
P61823	N94		Alpha helix, extremely solvent exposed, no hydrogen bonding
P61823	N103		Alpha helix, very solvent exposed, hydrogen bonding with backbone and Thr78
P00698	N27	**132L**	Alpha helix, very solvent exposed, hydrogen bonding with backbone and Ser24
P00698	N75		Alpha helix, very solvent exposed, hydrogen bonding with backbone
P00766	N50	**1AB9**	Alpha helix, extremely solvent exposed, no hydrogen bonding
P00766	Q116		Alpha helix, extremely solvent exposed, no hydrogen bonding
P00766	N165		Alpha helix, very solvent exposed, hydrogen bonding with backbone and Arg230
P00766	N167		Alpha helix, extremely solvent exposed, no hydrogen bonding
P00921	N67	**1G6V**	beta sheet, very solvent exposed, no hydrogen bonds
P00921	Q135		beta sheet, very solvent exposed, no hydrogen bonds
P00921	Q136		beta sheet, very solvent exposed, no hydrogen bonds
P00921	N152		
P00921	Q220		Alpha helix, very solvent exposed, hydrogen bonding with backbone
P00921	Q221		alpha helix, very solvent exposed, hydrogen bonding with backbone
P00921	N229		turn section, very solvent exposed, hydrogen bonding with backbone, very close to Lys227 and Lys167
P00921	N243		random coil, relatively hidden, hydrogen bonding with His96. Surrounded by aromatics (Phe230, Tyr7, Trp97)
P00921	Q248		turn section, very solvent exposed, hydrogen bonding with backbone
P00563	N5	**1U6R**	alpha helix, very solvent exposed, hydrogen bonding with Glu45 and Asn7
P00563	N63		Alpha helix, very solvent exposed, hydrogen bonding with backbone
P00563	Q184		Alpha helix, extremely solvent exposed, no hydrogen bonds
P00563	Q185		Alpha helix, extremely solvent exposed, no hydrogen bonds
P00563	N222		Alpha helix, extremely solvent exposed, no hydrogen bonds
P00563	Q275		Alpha helix, extremely solvent exposed, hydrogen bonding with Asp78

Supplementary Fig. 22c: CD Spectroscopy Studies on Modified Proteins

CD Spectroscopy Studies

CD Spectra were measured using a JASCO J-1500 CD Spectrometer with SpectraManager software. Three scans were averaged from 260-190 nm with a 0.2 nm data pitch and 100 nm s-1 scanning speed. 25 µL of the protein (native protein, nitrile, or ketone) dissolved in pH 7.45 10 mM NaP buffer (1 mg/mL concentration of protein) was added to a quartz High Precision Cell cuvette (Hellma Analytics, Jena, Germany) with a path length of 0.1 mm. Spectra were background corrected by subtraction of a blank spectrum of the pH 7.45 10 mM NaP buffer, and raw mdeg values were converted to mean residue ellipticity values [θ] using the following equation:

$$[\theta] = (\theta(\text{mdeg}) \times \text{MRW}) / (10 \times c \times L)$$

In the equation above, MRW is mean residue weight (e.g. ubiquitin MW (8565 g/mol) / number of residues (76)), c is the concentration of the protein in mg/mL (1 mg/mL for all proteins), and L is the path length of the cuvette in cm (0.01 cm).

Percent α-helicity was estimated from the mean residue ellipticity at 222 nm ($[\theta]_{222}$) using:

$$\% \alpha\text{-helicity} = (([\theta]_{222} - [\theta]_{coil}) / ([\theta]_{\alpha} - [\theta]_{coil})) \times 100$$

In the equation above, $[\theta]_{\alpha}$ = -33,000 deg·cm²·dmol⁻¹ was used as the reference ellipticity for a fully α-helical polypeptide at 222 nm and $[\theta]_{coil}$ = -2,000 deg·cm²·dmol⁻¹ was used as a random coil reference. Percent helicity values were calculated as averages of the three replicate measurements.

Importantly, lysozyme chicken maintains its secondary structure and calculated α-helicity across the full range of nitrile formation conditions (2-10 mM $Pd(OAc)_2$) as well as following aryl ketone installation, with only minor variations observed. Ubiquitin shows maintained secondary structure, and, as expected, ubiquitin exhibits low apparent α-helicity across all conditions, consistent with its predominantly β-sheet fold. Ribonuclease A similarly shows preserved secondary structure and helicity upon both nitrile dehydration and subsequent ketone formation. In contrast, creatine kinase, which is known to be more thermally sensitive (melting temperature ~50 °C),[5] displays a modest reduction in α-helicity following ketone formation, while largely retaining secondary structure after nitrile dehydration. This behavior highlights a practical boundary of the method for thermally labile proteins rather than a general limitation, and underscores the importance of protein-specific considerations when applying these reaction conditions.

Lysozyme Chicken Percent α-Helicity:

Condition	% α-Helicity
Unmodified Lysozyme Chicken	24.7%
Lysozyme Chicken Nitrile (2 mM Pd)	28.9%
Lysozyme Chicken Nitrile (3 mM Pd)	22.9%
Lysozyme Chicken Nitrile (4 mM Pd)	30.7%
Lysozyme Chicken Nitrile (5 mM Pd)	31.3%
Lysozyme Chicken Nitrile (10 mM Pd)	23.3%
Lysozyme Chicken Ketone	27.9%

Lysozyme Chicken CD Spectra:

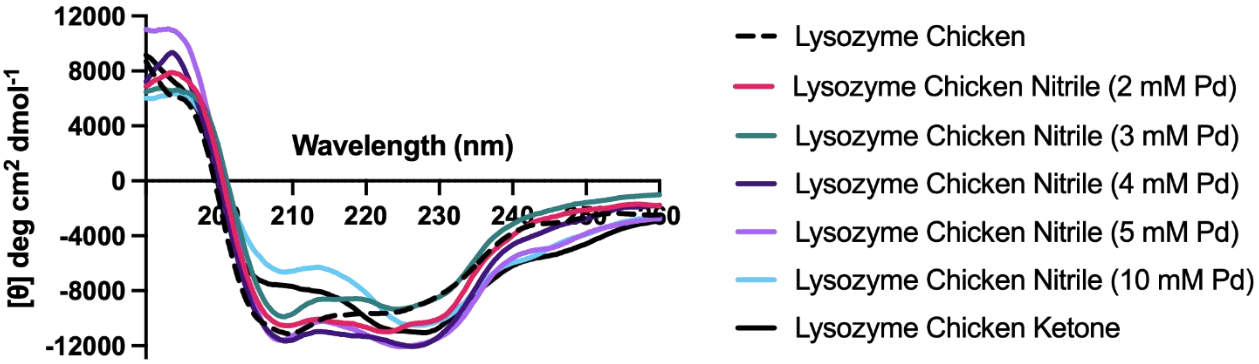

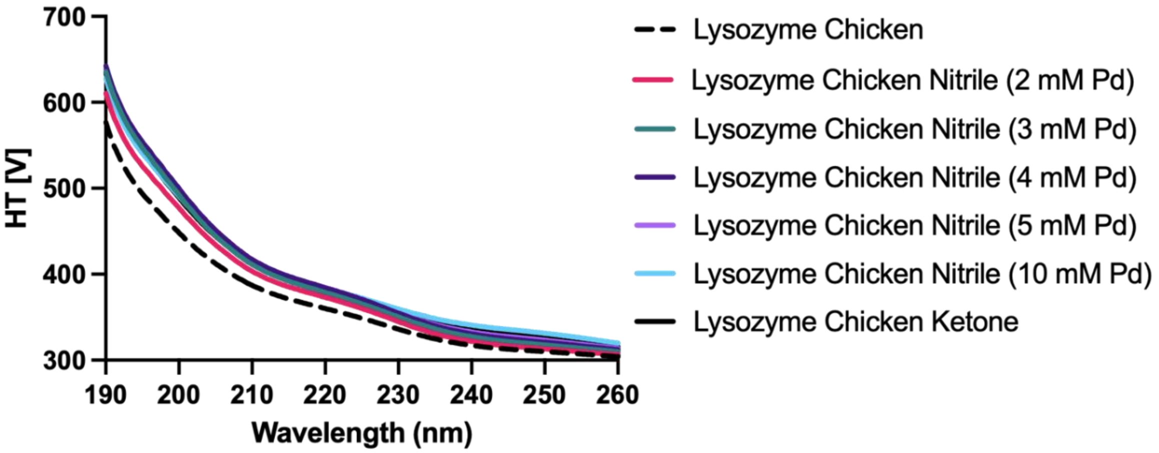

Ubiquitin Percent α-Helicity:

Condition	% α-Helicity
Unmodified Ubiquitin	8.9%
Ubiquitin Nitrile	8.6%
Ubiquitin Ketone	4.3%

Ubiquitin CD Spectra:

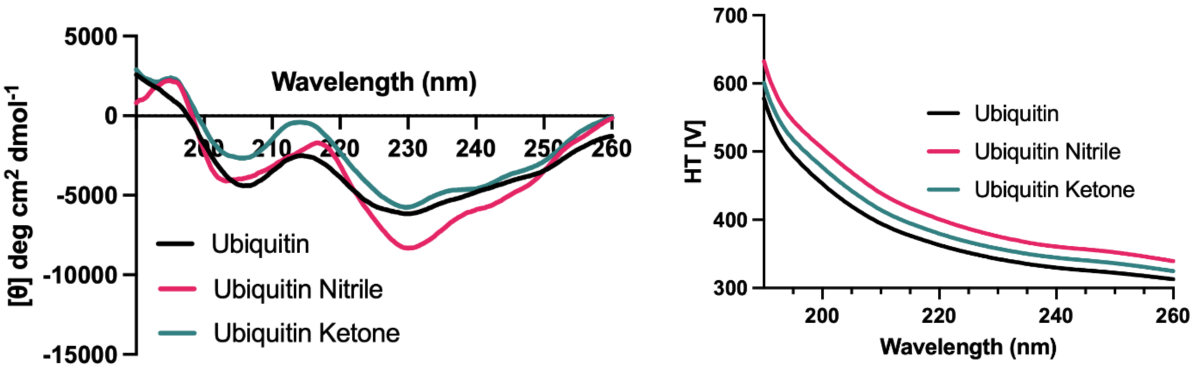

Ribonuclease A Percent α-Helicity:

Condition	% α-Helicity
Unmodified Ribonuclease A	20.3%
Ribonuclease A Nitrile	19.5%
Ribonuclease A Ketone	23.4%

Ribonuclease A CD Spectra:

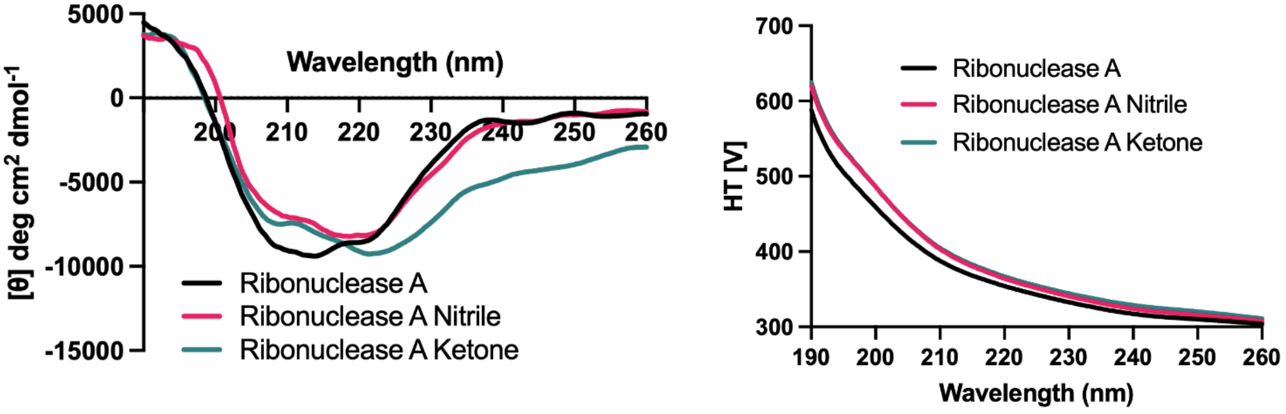

Creatine Kinase Percent α-Helicity:

Condition	% α-Helicity
Unmodified Creatine Kinase	33.3%
Creatine Kinase Nitrile	28.3%
Creatine Kinase Ketone	21.2%

Creatine Kinase CD Spectra:

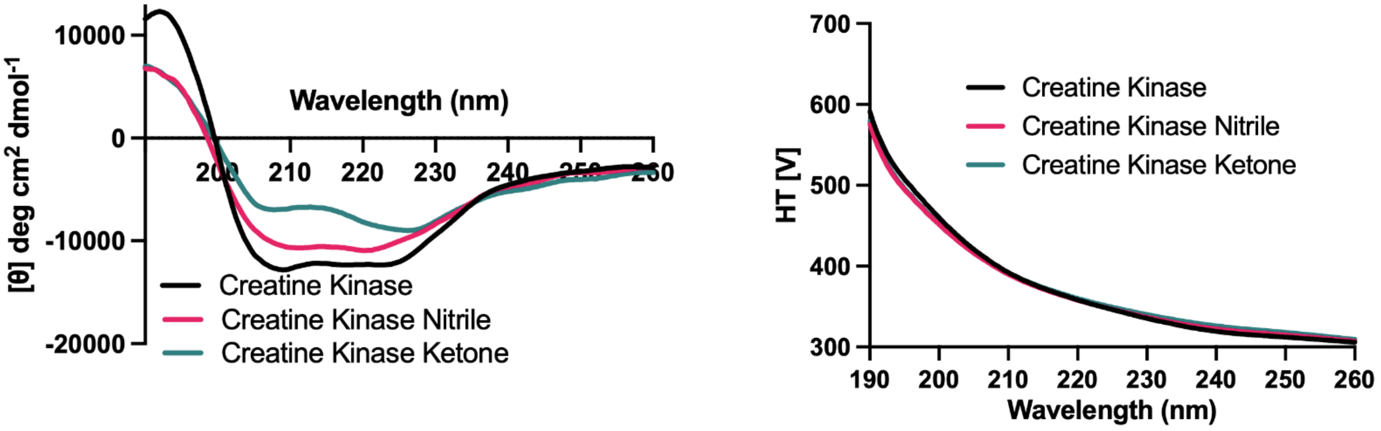

Supplementary Fig. 23: Incorporation of Affinity Handles via Boronic Acid Carbometallation

To demonstrate the various applications for the boronic acid cross-coupling chemistry on protein, boronic acids containing affinity tags (azide, alkyne, ketone, vinyl) were attached to nitrile-modified bovine serum albumin (BSA) through the carbometallation reaction.

Attachment of Azide Affinity Tag to BSA

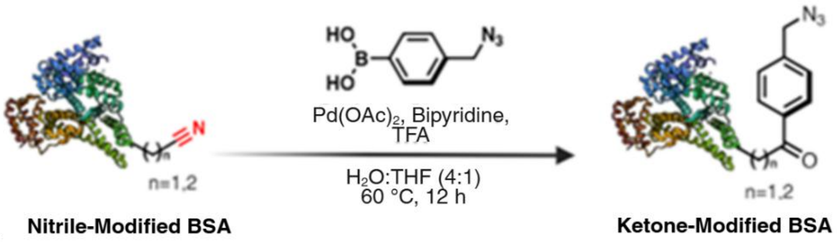

Reaction conducted according to **GP-XVI**. 2 mg of nitrile-modified bovine serum albumin (BSA) (60 µM final concentration) was dissolved in 350 µL of H_2O in a 1/2" dram vial, after which 2 µL of TFA was added. Next, 2 mg of azide-phenylboronic acid dissolved in 50 µL THF was added to the mixture. N_2 was then bubbled into the solution for 3 minutes using an 18G x 1 ½" needle and balloon. In a separate vial, 1 mg of bipyridine and 1 mg of $Pd(OAc)_2$ were dissolved in 100 µL of 1:1 THF:H_2O and stirred for 15 minutes at room temperature to generate a pre-formed bipyridine-palladium complex. This pre-formed complex was then added to the reaction mixture, and N_2 was bubbled for an additional 2 minutes. The reaction vial was then sealed and stirred for 12 hours at 60 °C. The Pd was quenched by addition of 200 µL 1 M aqueous L-cysteine and 10 µL of 1 M NaOH. The crude reaction mixture was passed through Amicon™ Ultra 3 kDa spin-concentrator and washed with H_2O (7×0.5 mL) to remove the small molecule impurities. The labeled protein was lyophilized, digested using SMART Digest™ Trypsin Kit by Thermo Scientific, and then analyzed using LC-MS/MS. 9 distinct sites were modified with the azide affinity tag.

MS/MS Analysis of Digested Modified Bovine Serum Albumin

Identified peptide fragment (**2 sites**): GLVLIAFSQYLQQCPFDEHV (Sequence: AA 21-40, [Q29, Q32])

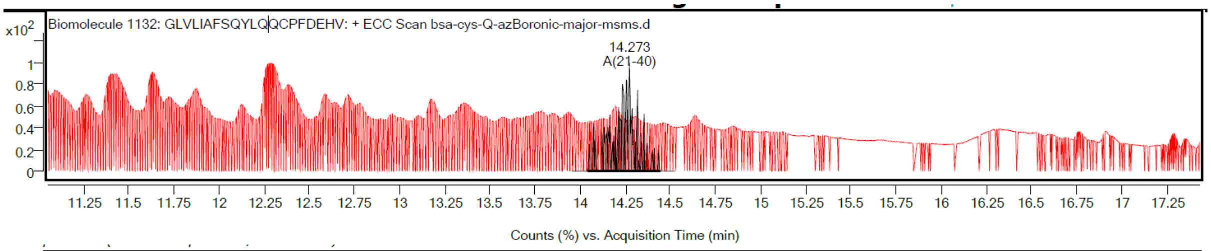

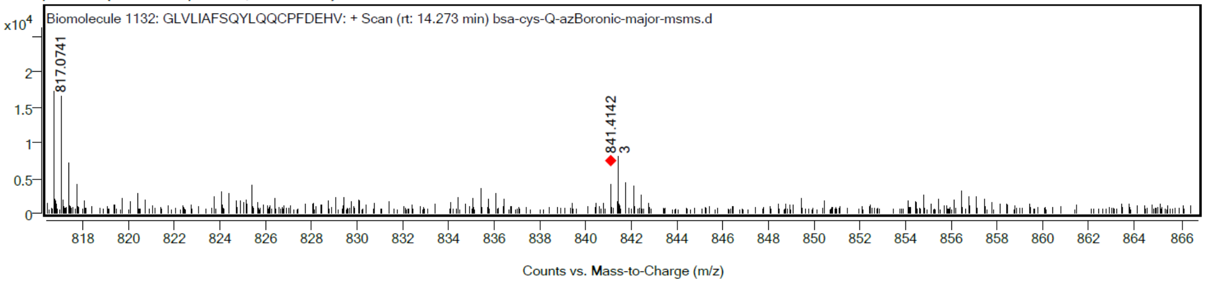

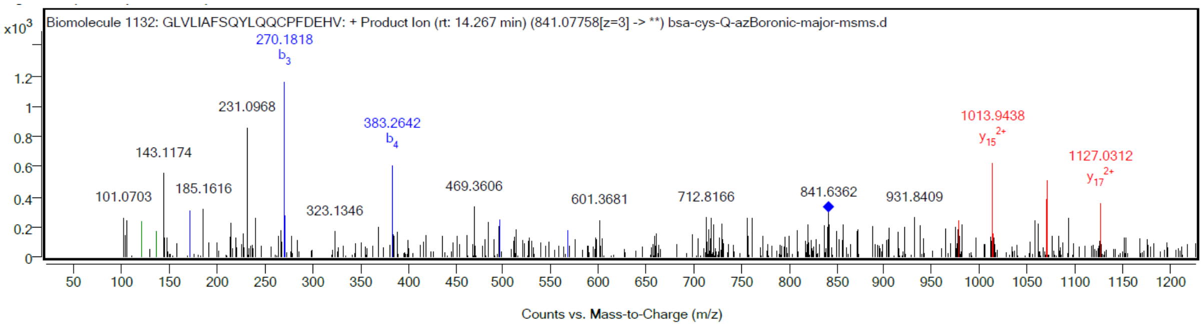

Fragment Spectrum Peaks

m/z	Diff (ppm)	Abund	Ion
977.9538	-34.40	239	y14
1013.9438	-3.41	620	y15
1070.9851	-0.99	507	y16
1127.0312	-6.04	354	y17
171.1134	-3.32	306	b2
270.1818	-2.08	1155	b3
383.2642	2.76	602	b4
496.3503	-1.92	244	b5
567.3881	-2.97	175	b6
120.0782	21.16	236	F
136.0757	-0.40	167	Y

Identified peptide fragment (**2 sites**): GLVLIAFSQYLQQCPFDEHVKLVNELTEFA (Sequence: AA 21-40, [Q29, N44])

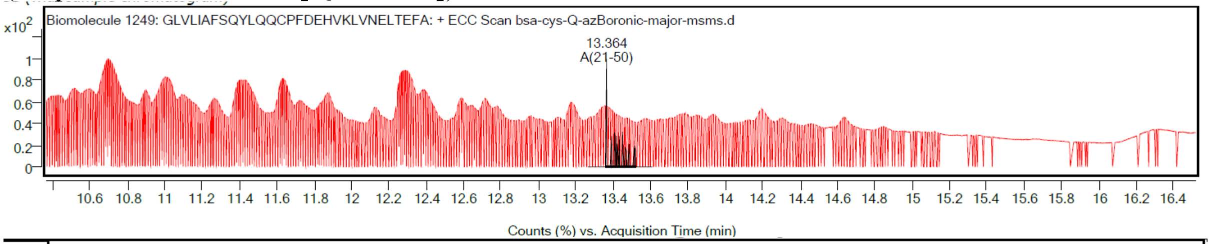

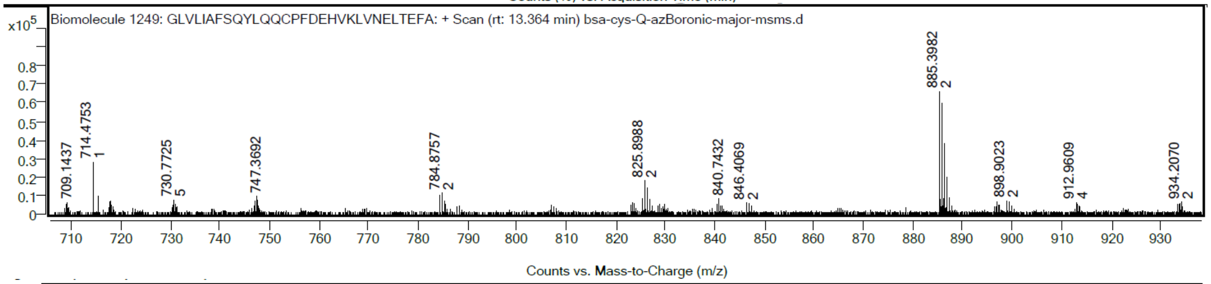

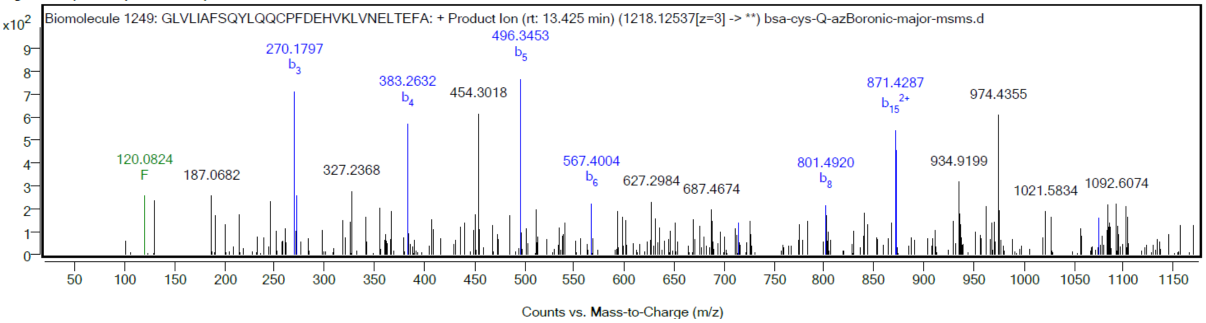

Fragment Spectrum Peaks

m/z	Diff (ppm)	Abund	Ion
270.1797	5.50	711	b3
383.2632	5.38	569	b4
496.3453	8.18	767	b5
567.4004	-24.56	221	b6
714.4448	14.14	140	b7
801.4920	-6.35	214	b8
1074.5904	7.32	161	b10
871.4287	21.90	542	b15
120.0824	-13.39	257	F

Identified peptide fragment (**1 site**): LLYYANKYNGVFQECCQAEDK (Sequence: AA 153-173, N161)

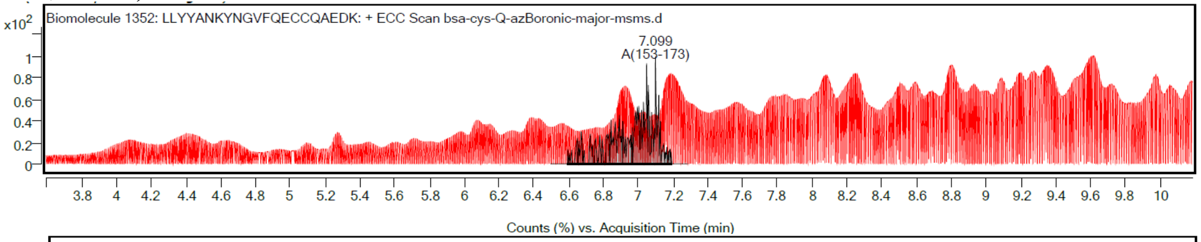

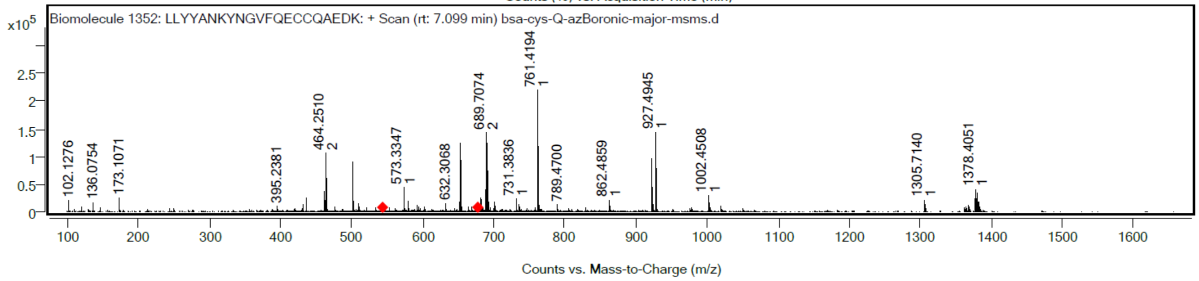

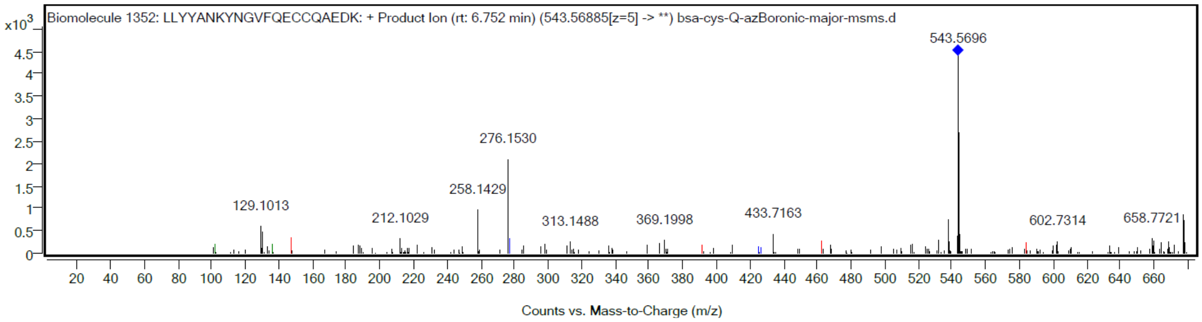

Fragment Spectrum Peaks

m/z	Diff (ppm)	Abund	Ion
147.1141	-8.98	339	y1
391.1830	-1.70	174	y3
462.2234	-8.64	277	y4
584.2382	-24.54	232	y9
277.1546	0.38	333	b4
424.7201	39.54	137	b7
102.0576	-26.14	197	E
136.0780	-16.95	199	Y
543.2422	8.47	382	Precursor
543.6441	7.45	234	Precursor

Identified peptide fragment (**1 site**): IQKFGERALK (Sequence: AA 202-211, Q203)

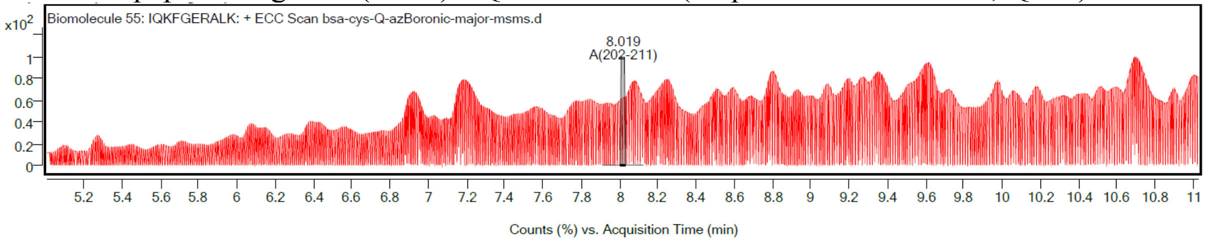

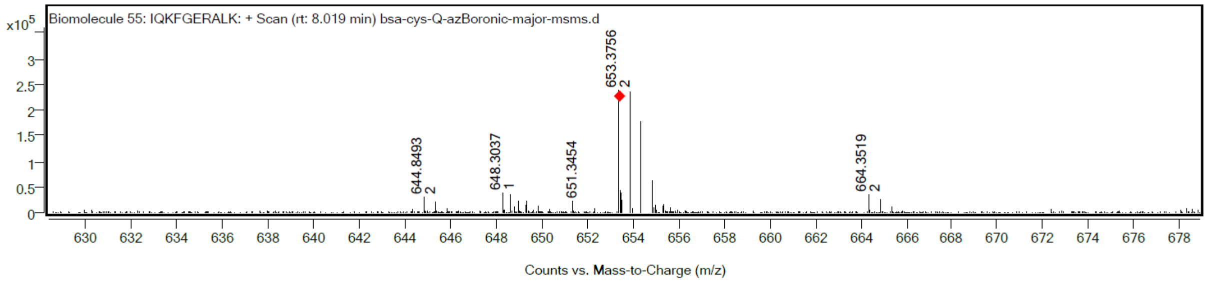

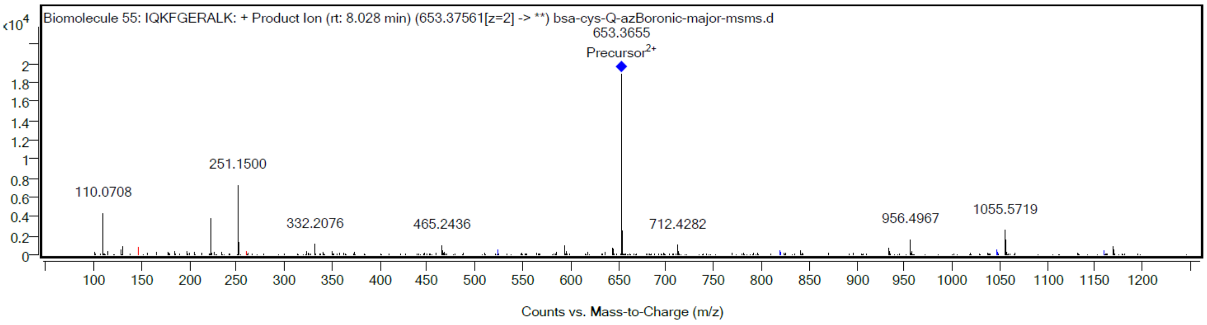

Fragment Spectrum Peaks

m/z	Diff (ppm)	Abund	Ion
147.1132	-2.76	739	y1
260.1980	-4.18	356	y2
819.3939	25.49	381	b6
1046.5246	27.13	491	b8
1159.6028	29.55	453	b9
523.7568	44.58	487	b8
653.3655	14.47	18866	Precursor

Identified peptide fragment (**1 site**): HLVDEPQNLIKQNCD (Sequence: AA 378-392, N390)

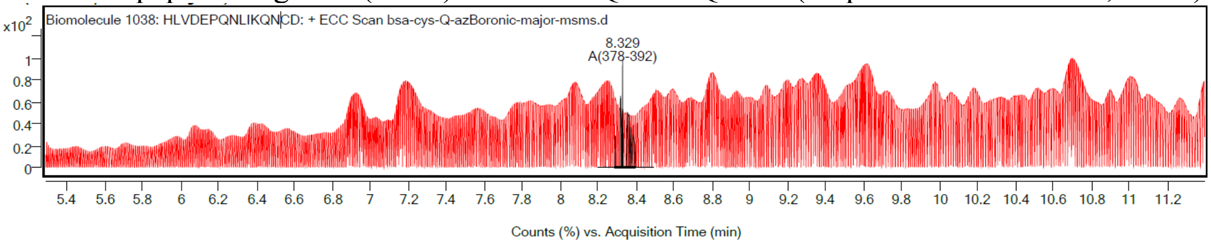

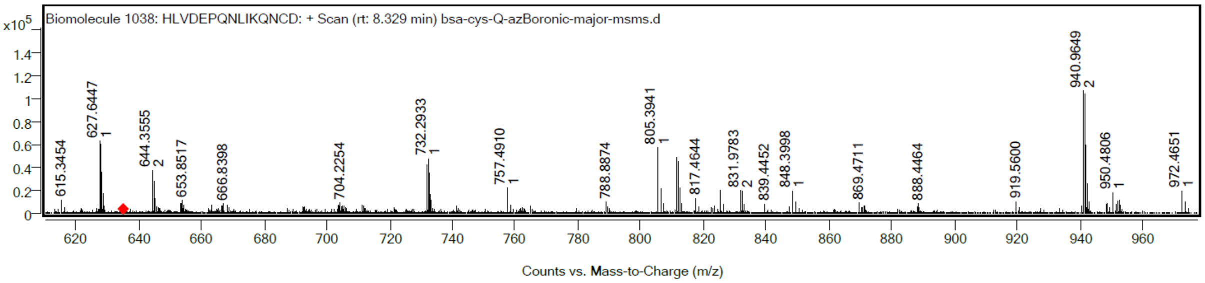

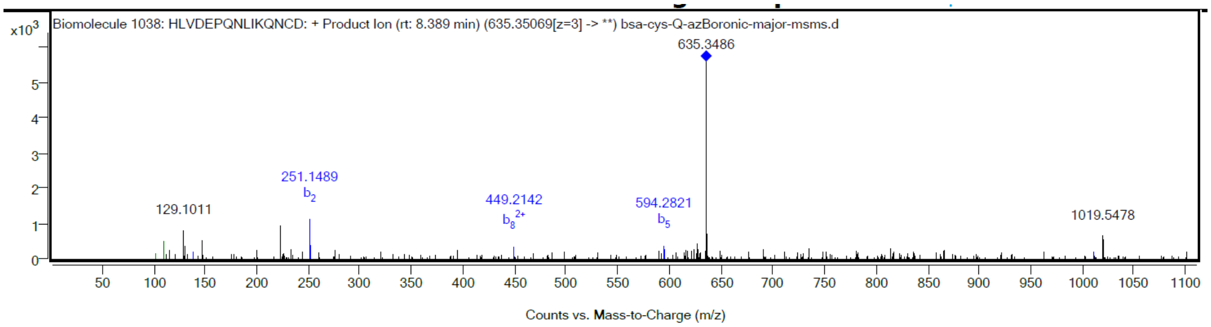

Fragment Spectrum Peaks

m/z	Diff (ppm)	Abund	Ion
138.0676	-9.98	173	b1
251.1489	5.50	1099	b2
594.2821	10.19	351	b5
1010.5036	1.77	172	b9
449.2142	0.36	330	b8
102.0539	10.58	140	E
110.0708	3.86	491	H
634.9626	9.53	161	Precursor
635.3064	-5.13	922	Precursor
635.6373	0.40	204	Precursor
635.9751	-4.76	488	Precursor

Identified peptide fragment (**1 site**): NCDQFEKLGEYGFQNALIVR (Sequence: AA 390-409, N404)

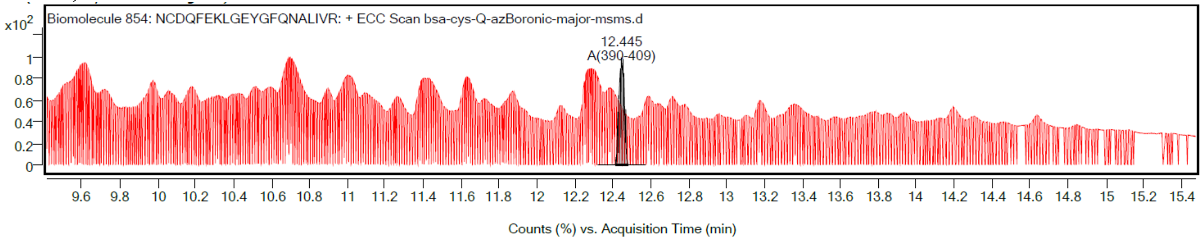

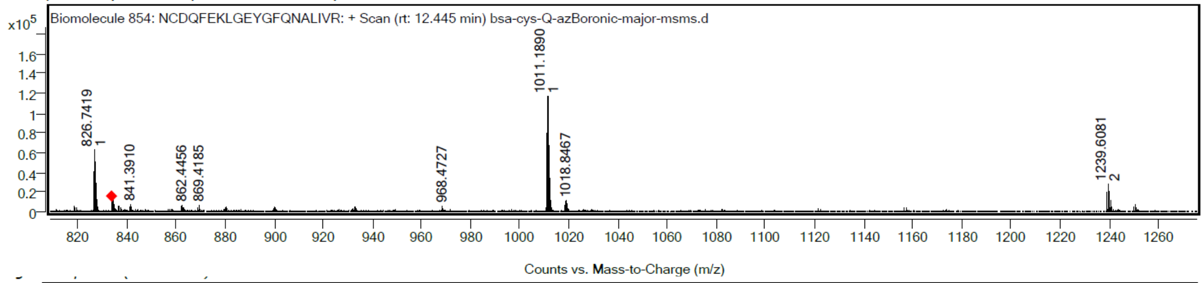

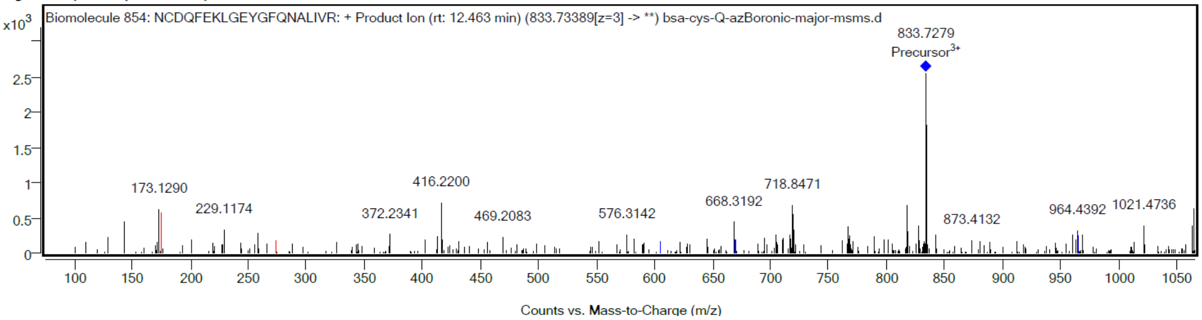

Fragment Spectrum Peaks

m/z	Diff (ppm)	Abund	Ion
175.1195	-3.34	570	y1
274.1865	3.02	175	y2
604.7811	-32.78	160	b9
669.3032	-30.72	192	b10
965.4403	-35.20	246	b15
833.7279	7.25	2543	Precursor
834.0659	3.03	2443	Precursor
834.4013	1.94	1806	Precursor
834.7336	4.53	510	Precursor

Identified peptide fragment (**1 site**): VTKCCTESLVNRRPC (Sequence: AA 472-486, N482)

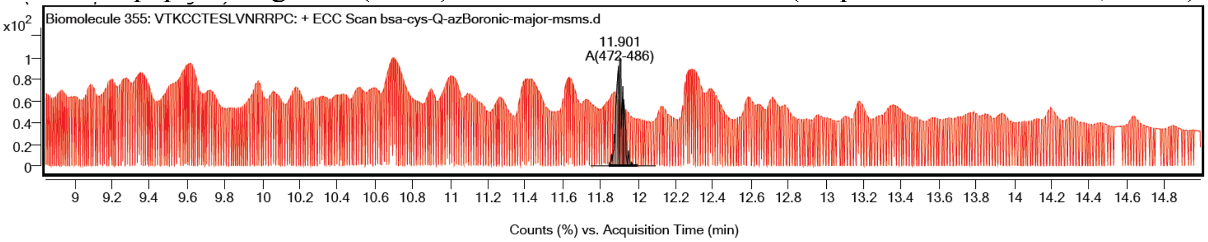

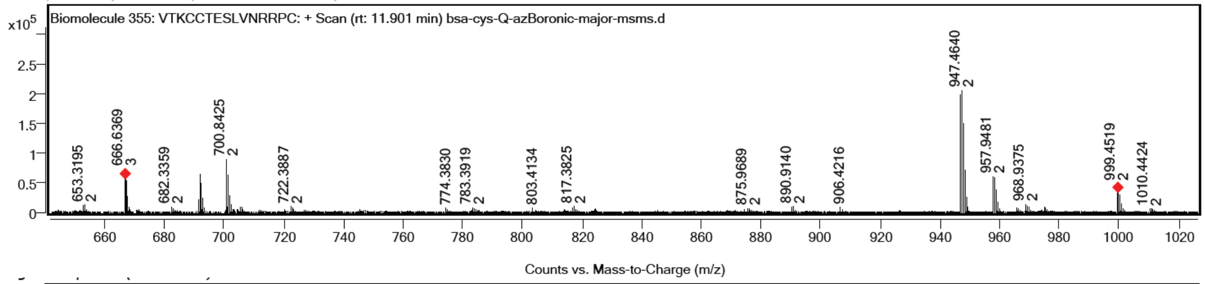

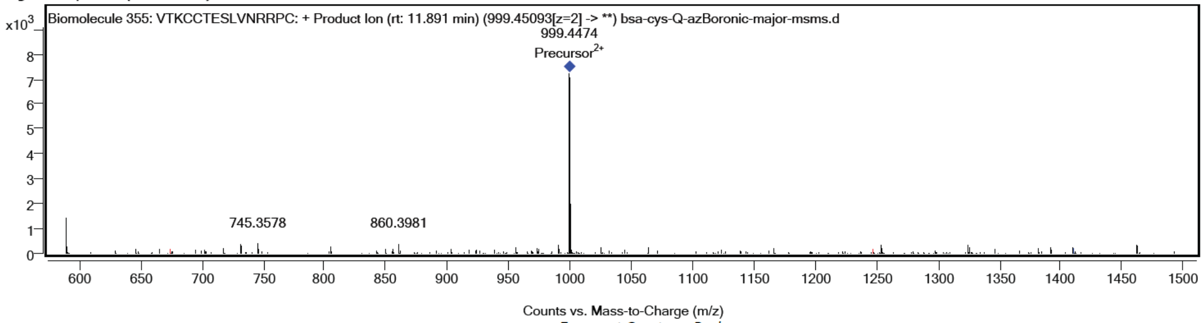

Fragment Spectrum Peaks

m/z	Diff (ppm)	Abund	Ion
1246.5722	31.10	190	y9
674.3183	21.76	171	y10
1410.6190	-11.50	212	b11
999.4474	5.80	7206	Precursor
999.9500	4.79	7054	Precursor
1000.4507	5.78	1999	Precursor
1000.9486	9.56	161	Precursor

Identified peptide fragment (**1 site**): TLPDTEKQIK (Sequence: AA 514-523, Q521)

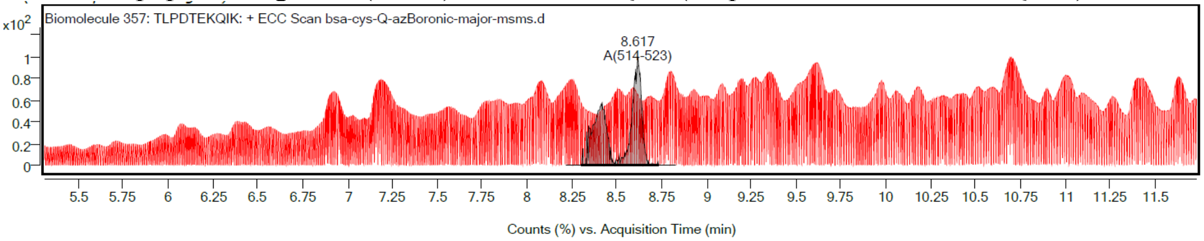

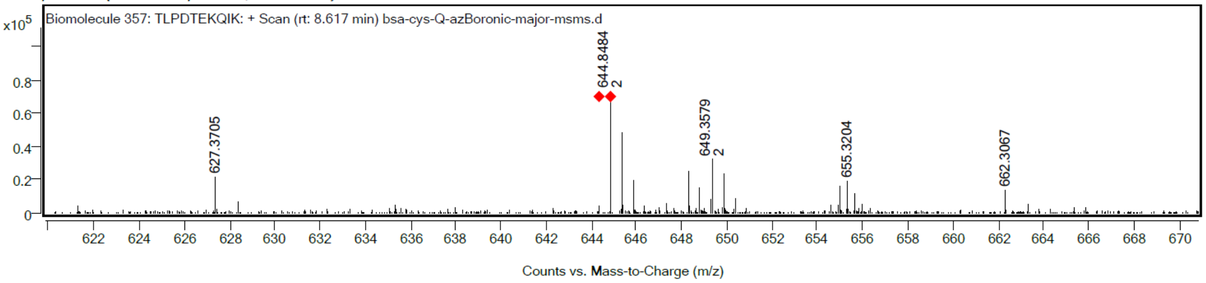

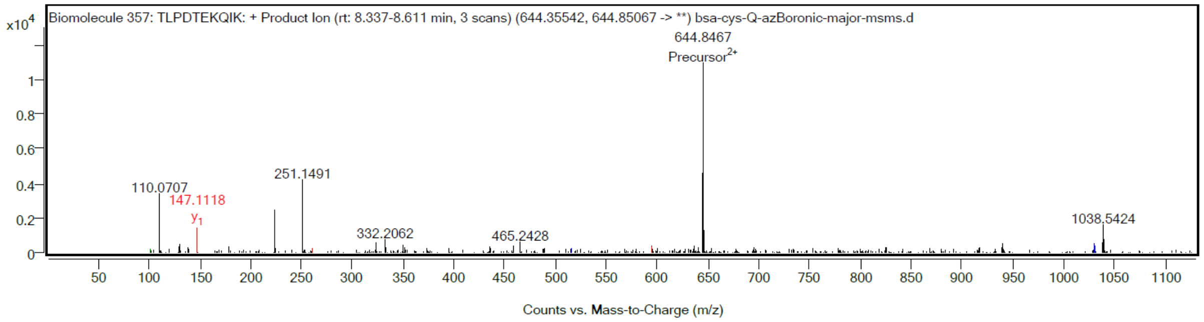

Fragment Spectrum Peaks

m/z	Diff (ppm)	Abund	Ion
147.1118	6.71	1403	y1
260.1980	-4.46	254	y2
594.2970	46.40	345	y9
1029.5229	-22.23	499	b8
515.2575	-7.45	255	b8
101.1071	2.51	209	K
644.8467	2.65	10938	Precursor
645.3464	5.78	9848	Precursor
645.8537	-3.03	1288	Precursor

Attachment of Alkyne Affinity Tag to BSA

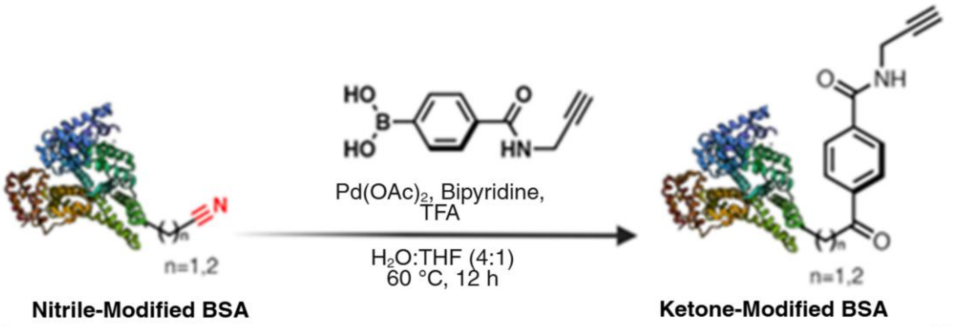

Reaction conducted according to **GP-XVI**. 2 mg of nitrile-modified bovine serum albumin (BSA) (60 µM final concentration) was dissolved in 350 µL of H_2O in a 1/2" dram vial, after which 2 µL of TFA was added. Next, 2 mg of alkyne-phenylboronic acid dissolved in 50 µL THF was added to the mixture. N_2 was then bubbled into the solution for 3 minutes using an 18G x 1 ½" needle and balloon. In a separate vial, 1 mg of bipyridine and 1 mg of $Pd(OAc)_2$ were dissolved in 100 µL of 1:1 THF:H_2O and stirred for 15 minutes at room temperature to generate a pre-formed bipyridine-palladium complex. This pre-formed complex was then added to the reaction mixture, and N_2 was bubbled for an additional 2 minutes. The reaction vial was then sealed and stirred for 12 hours at 60 °C. The Pd was quenched by addition of 200 µL 1 M aqueous L-cysteine and 10 µL of 1 M NaOH. The crude reaction mixture was passed through Amicon™ Ultra 3 kDa spin-concentrator and washed with H_2O (7×0.5 mL) to remove the small molecule impurities. The labeled protein was lyophilized, digested using SMART Digest™ Trypsin Kit by Thermo Scientific, and then analyzed using LC-MS/MS. 4 distinct sites were modified with the alkyne affinity tag.

MS/MS Analysis of Digested Modified Bovine Serum Albumin

Identified peptide fragment (**1 site**): VLIAFSQYLQQCPFDEHVK (Sequence: AA 23-41, Q29)

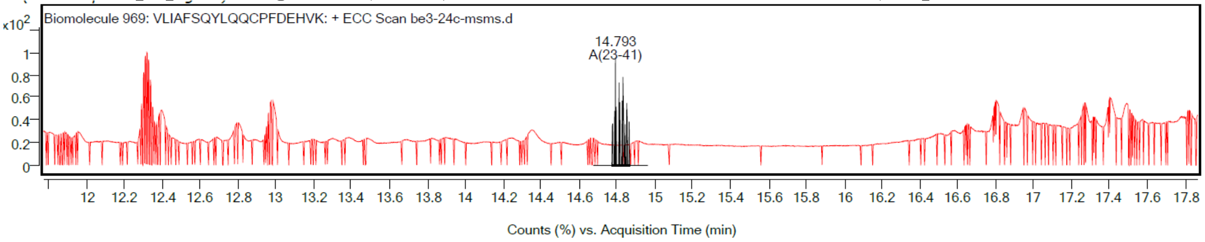

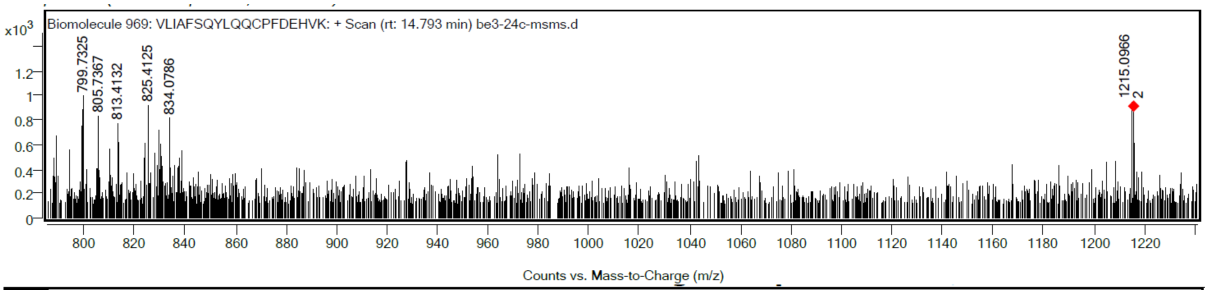

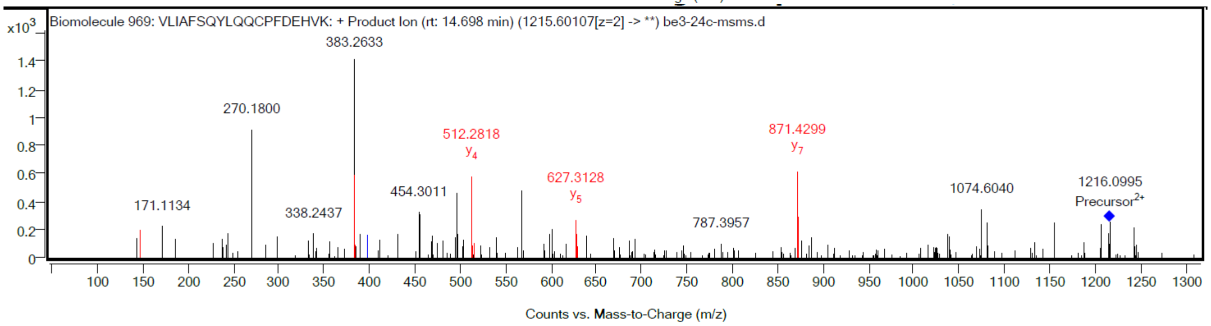

Fragment Spectrum Peaks

m/z	Diff (ppm)	Abund	Ion
147.1152	-16.59	194	y1
383.2393	2.28	585	y3
512.2818	1.84	572	y4
627.3128	-5.02	260	y5
871.4299	1.12	608	y7
397.2778	7.95	160	b4
1215.1017	-5.48	170	Precursor
1215.6091	-10.21	90	Precursor
1216.0995	-0.93	248	Precursor

Identified peptide fragment (**2 sites**): VLIAFSQYLQQCPFDEHVK (Sequence: AA 161-173, [Q165, Q169])

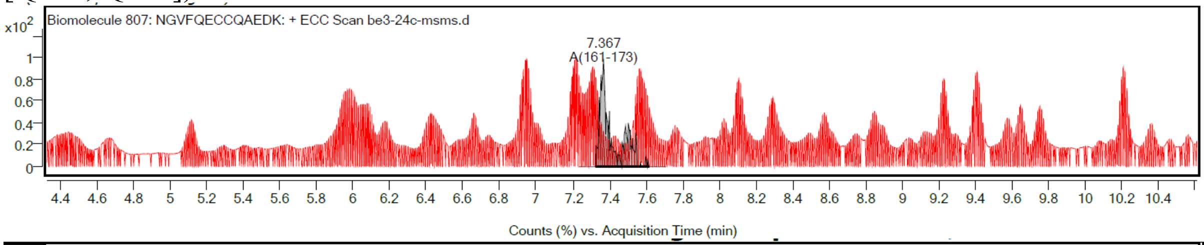

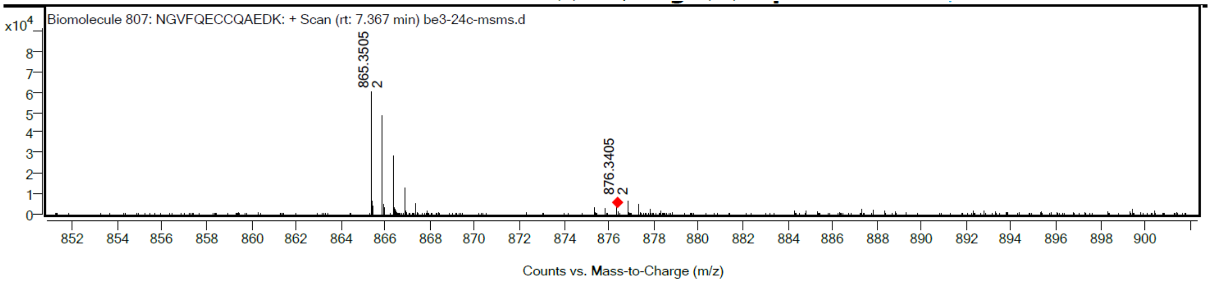

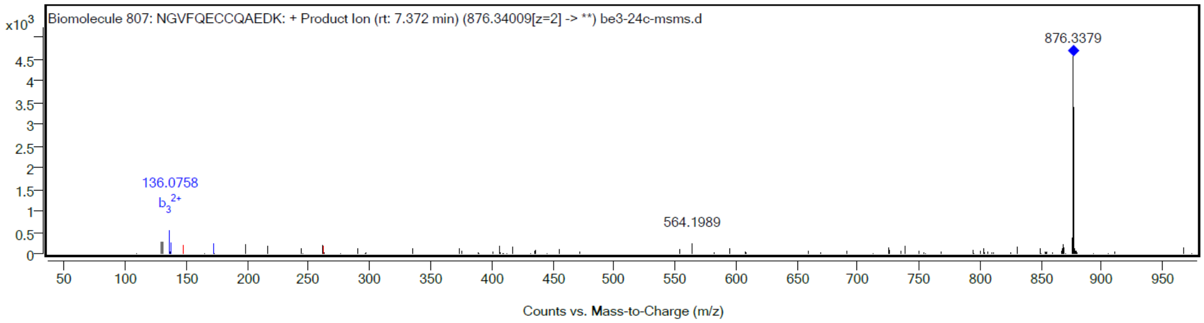

Fragment Spectrum Peaks

m/z	Diff (ppm)	Abund	Ion
147.1130	-1.10	197	y1
262.1324	28.21	172	y2
172.0718	-0.93	239	b2
136.0758	-15.48	543	b3
877.8566	-13.27	60	Precursor

Identified peptide fragment (**1 site**): AWSVARLSQK (Sequence: AA 212-221, Q220)

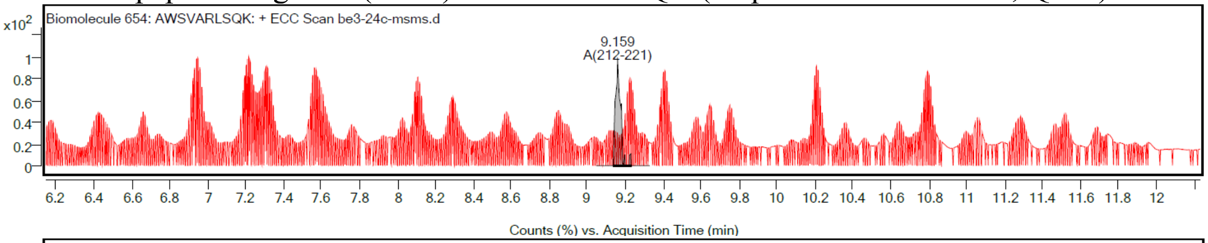

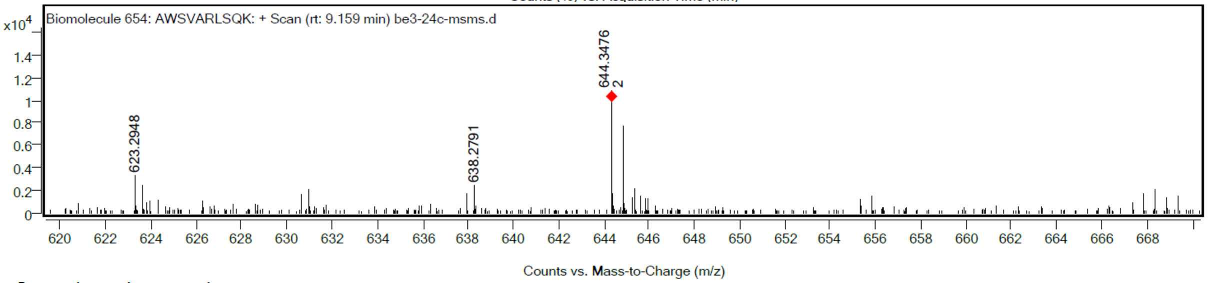

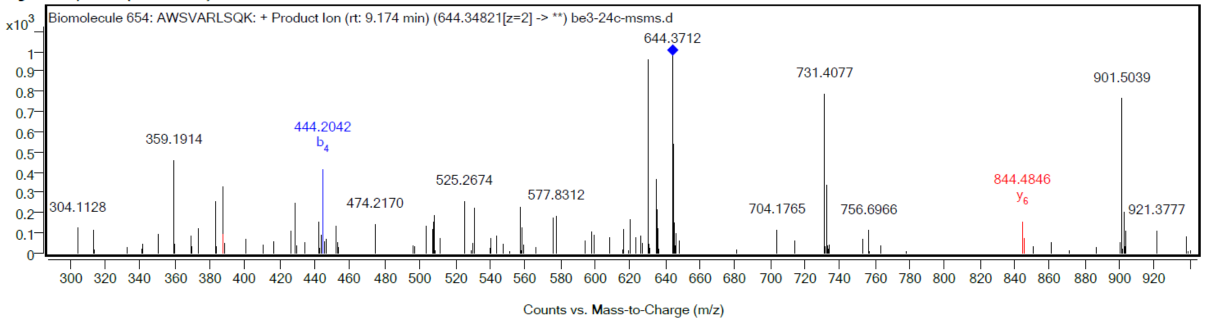

Fragment Spectrum Peaks

m/z	Diff (ppm)	Abund	Ion
844.4846	-20.21	153	y6
387.2091	25.12	92	y5
444.2042	44.88	414	b4
644.3532	-11.44	800	Precursor
644.8548	-11.24	539	Precursor
645.3427	10.14	104	Precursor

Attachment of Ketone Affinity Tag to BSA

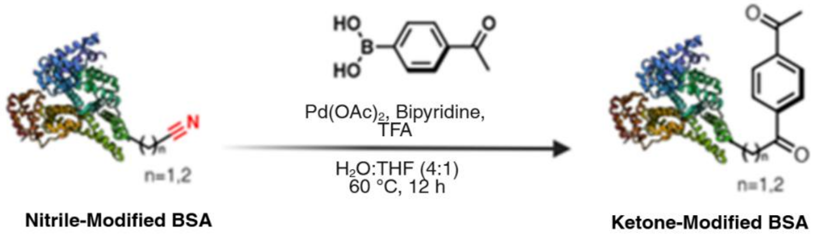

Reaction conducted according to **GP-XVI**. 2 mg of nitrile-modified bovine serum albumin (BSA) (60 µM final concentration) was dissolved in 350 µL of H_2O in a 1/2” dram vial, after which 2 µL of TFA was added. Next, 2 mg of 4-acetylphenylboronic acid dissolved in 50 µL THF was added to the mixture. N_2 was then bubbled into the solution for 3 minutes using an 18G x 1 ½” needle and balloon. In a separate vial, 1 mg of bipyridine and 1 mg of $Pd(OAc)_2$ were dissolved in 100 µL of 1:1 THF:H_2O and stirred for 15 minutes at room temperature to generate a pre-formed bipyridine-palladium complex. This pre-formed complex was then added to the reaction mixture, and N_2 was bubbled for an additional 2 minutes. The reaction vial was then sealed and stirred for 12 hours at 60 °C. The Pd was quenched by addition of 200 µL 1 M aqueous L-cysteine and 10 µL of 1 M NaOH. The crude reaction mixture was passed through Amicon™ Ultra 3 kDa spin-concentrator and washed with H_2O (7×0.5 mL) to remove the small molecule impurities. The labeled protein was lyophilized, digested using SMART Digest™ Trypsin Kit by Thermo Scientific, and then analyzed using LC-MS/MS. 4 distinct sites were modified with the ketone affinity tag.

MS/MS Analysis of Digested Modified Bovine Serum Albumin

Identified peptide fragment (**2 sites**): VFQECCQAEDK (Sequence: AA 163-173, [Q165, Q169])

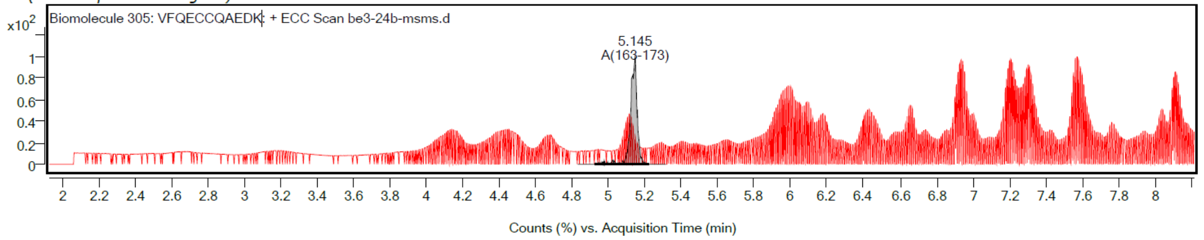

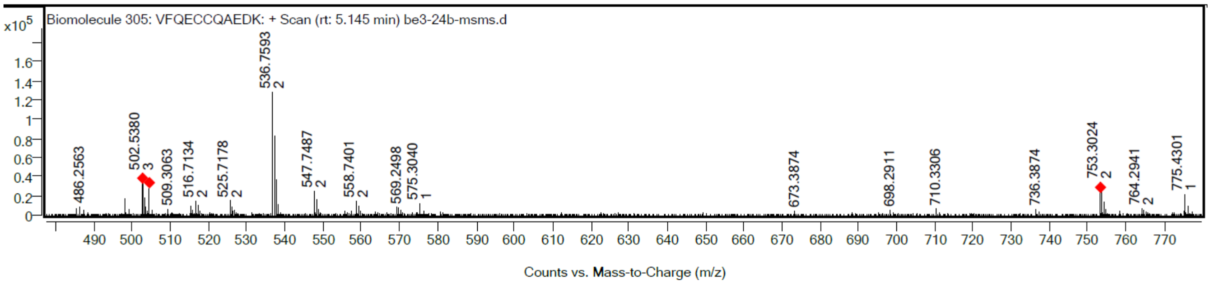

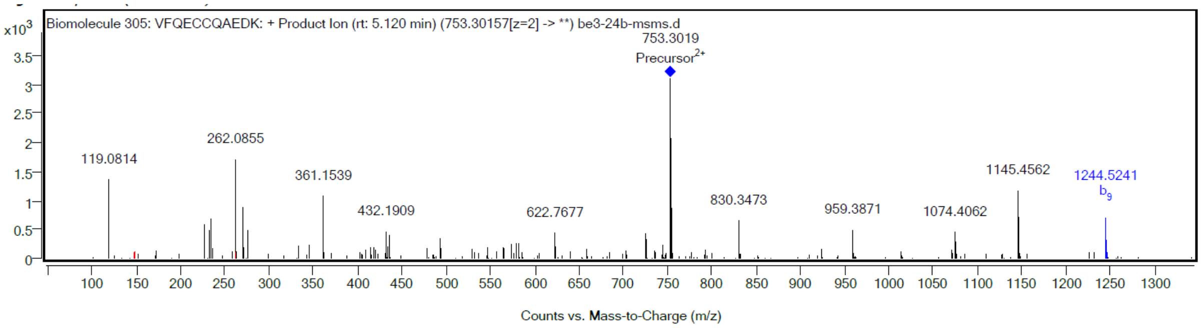

Fragment Spectrum Peaks

m/z	Diff (ppm)	Abund	Ion
148.1151	7.28	124	y1
262.1266	49.98	112	y2
1244.5241	-48.39	696	b9
753.3019	-0.13	3093	Precursor
753.8047	-1.58	2075	Precursor
754.3013	5.15	874	Precursor

Identified peptide fragment (**1 site**): PQVSTPTLVEVSR (Sequence: AA 415-427, Q417)

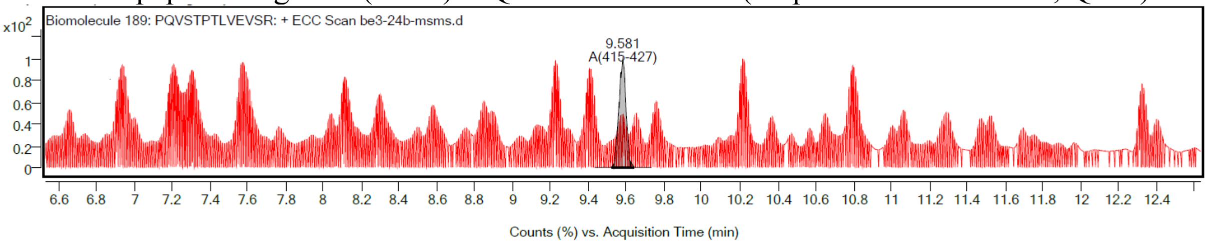

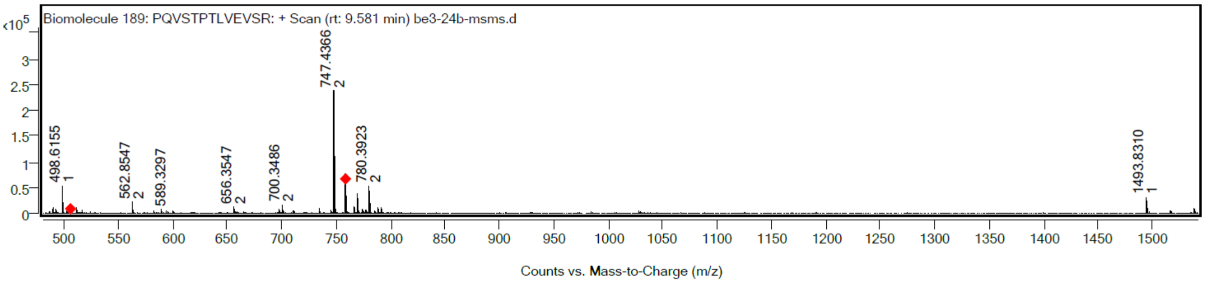

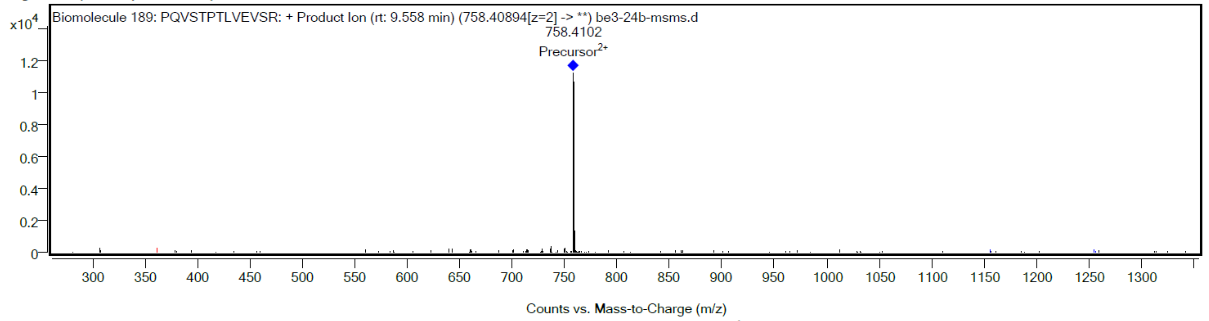

Fragment Spectrum Peaks

m/z	Diff (ppm)	Abund	Ion
361.2180	3.90	255	y3
1155.5891	3.55	121	b10
1254.6652	-2.81	126	b11
758.4102	-5.15	11201	Precursor
758.9101	-2.72	10627	Precursor
759.4089	1.00	1347	Precursor

Identified peptide fragment (**1 site**): VMENFVAFVDK (Sequence: AA 546-556, N549)

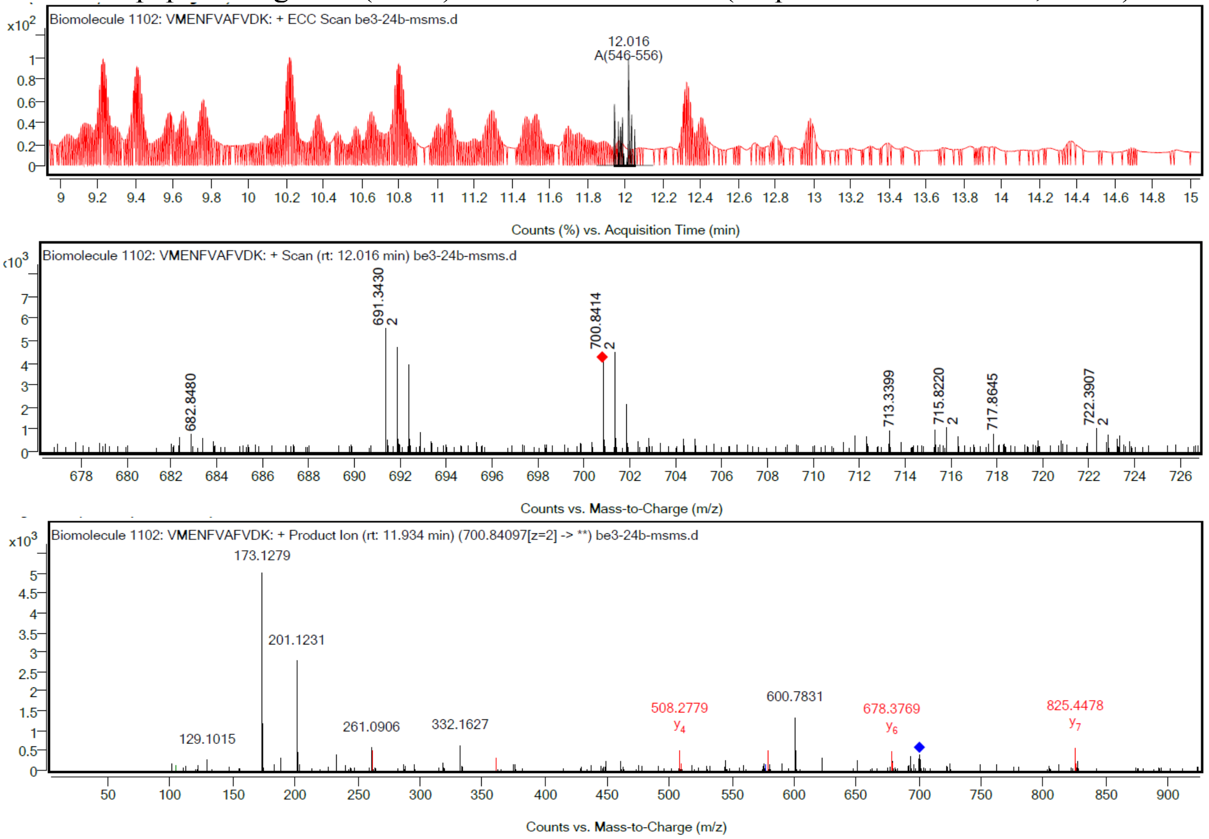

Fragment Spectrum Peaks

m/z	Diff (ppm)	Abund	Ion
361.2059	6.14	310	y3
508.2779	-2.52	493	y4
579.3144	-1.28	485	y5
678.3769	7.74	460	y6
825.4478	3.30	551	y7
577.2402	-13.03	131	b4
104.0512	15.79	115	M
701.3419	-0.43	259	Precursor

Attachment of Vinyl Affinity Tag to BSA

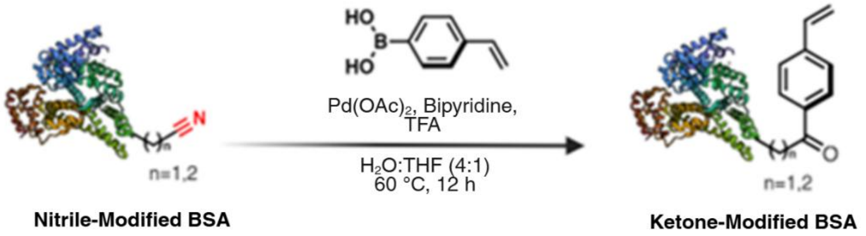

Reaction conducted according to **GP-XVI**. 2 mg of nitrile-modified bovine serum albumin (BSA) (60 µM final concentration) was dissolved in 350 µL of H_2O in a 1/2” dram vial, after which 2 µL of TFA was added. Next, 2 mg of 4-vinylphenylboronic acid dissolved in 50 µL THF was added to the mixture. N_2 was then bubbled into the solution for 3 minutes using an 18G x 1 ½” needle and balloon. In a separate vial, 1 mg of bipyridine and 1 mg of $Pd(OAc)_2$ were dissolved in 100 µL of 1:1 THF:H_2O and stirred for 15 minutes at room temperature to generate a pre-formed bipyridine-palladium complex. This pre-formed complex was then added to the reaction mixture, and N_2 was bubbled for an additional 2 minutes. The reaction vial was then sealed and stirred for 12 hours at 60 °C. The Pd was quenched by addition of 200 µL 1 M aqueous L-cysteine and 10

μL of 1 M NaOH. The crude reaction mixture was passed through Amicon™ Ultra 3 kDa spin-concentrator and washed with H_2O (7×0.5 mL) to remove the small molecule impurities. The labeled protein was lyophilized, digested using SMART Digest™ Trypsin Kit by Thermo Scientific, and then analyzed using LC-MS/MS. 9 distinct sites were modified with the vinyl affinity tag.

MS/MS Analysis of Digested Modified Bovine Serum Albumin

Identified peptide fragment (**1 site**): EHVKLVNELTEFAK (Sequence: AA 38-51, N44)

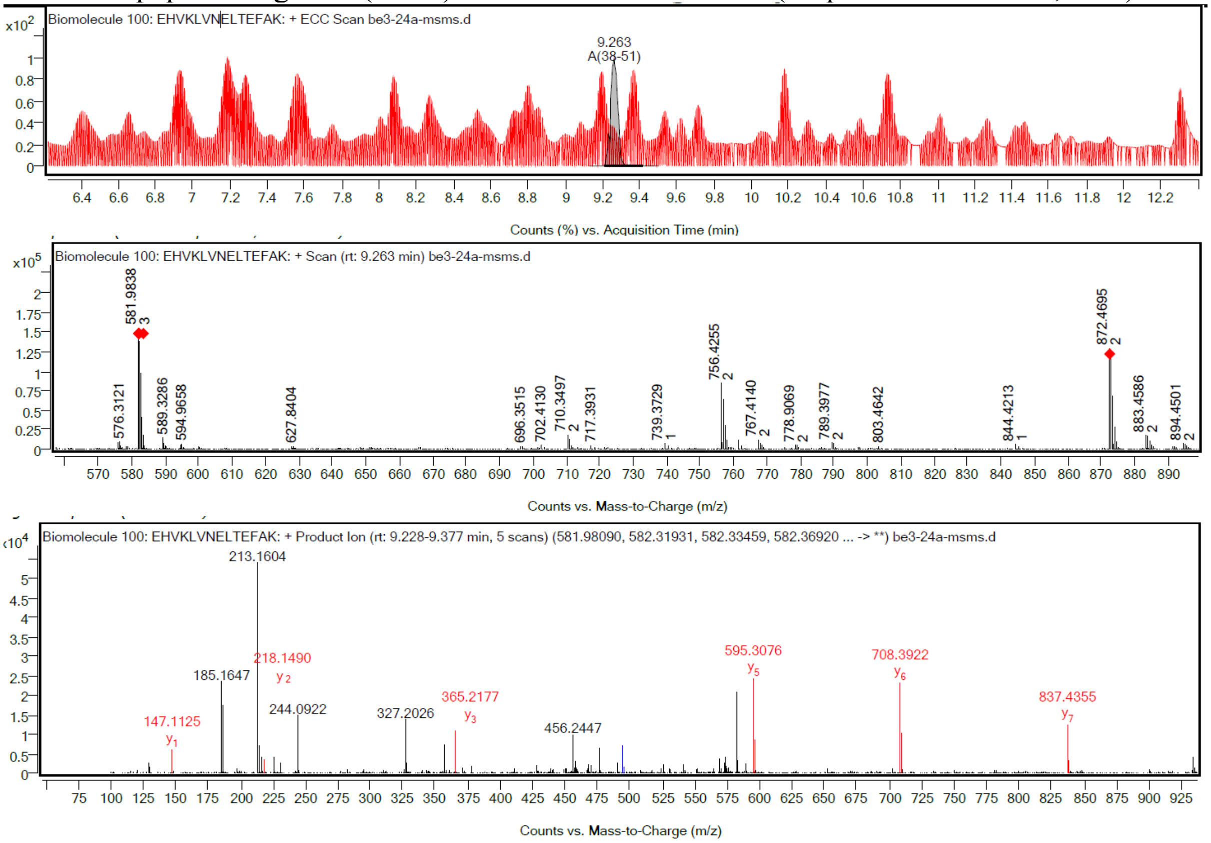

Fragment Spectrum Peaks

m/z	Diff (ppm)	Abund	Ion
147.1125	1.85	5943	y1
218.1490	4.28	3534	y2
365.2177	1.72	10856	y3
595.3076	1.75	24251	y5
708.3922	0.69	23228	y6
837.4355	-0.33	12387	y7
494.2609	22.82	7130	b4

Identified peptide fragment (**1 site**): NGVFQECCQAEDK (Sequence: AA 161-173, Q69)

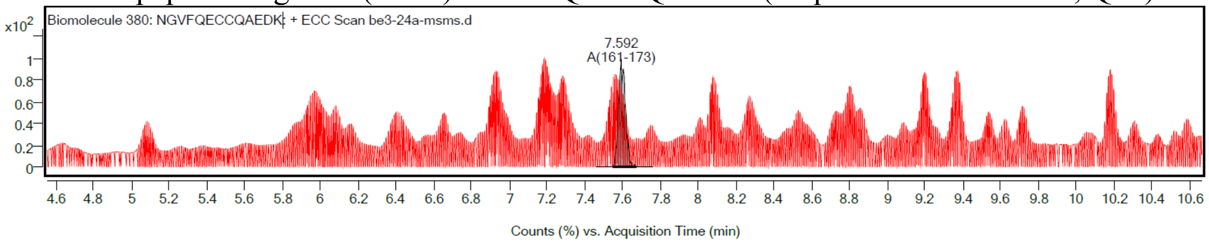

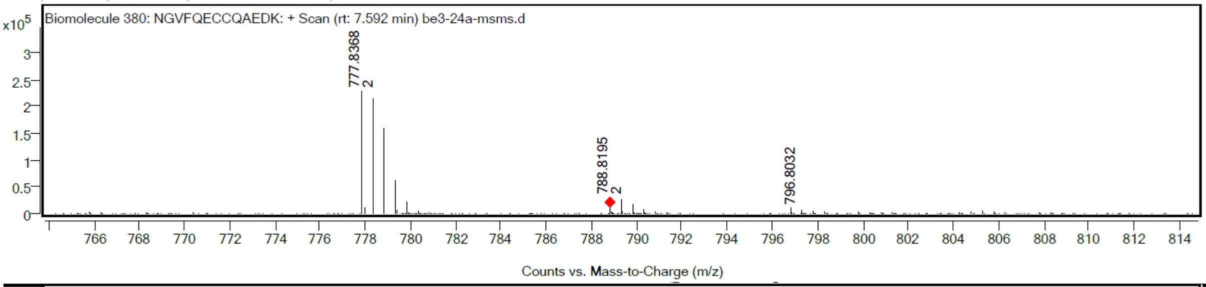

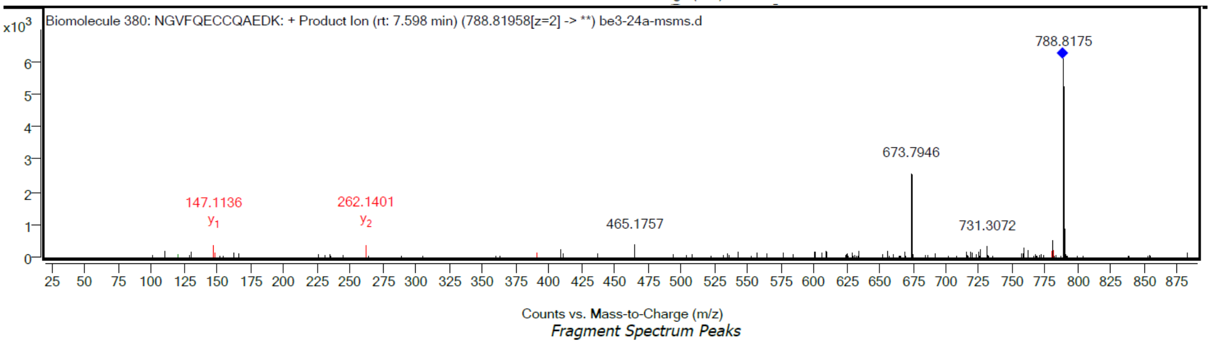

Fragment Spectrum Peaks

m/z	Diff (ppm)	Abund	Ion
147.1136	-5.67	336	y1
262.1401	-1.37	357	y2
391.1980	-40.07	130	y3
781.3018	31.81	183	y6
120.0803	3.58	68	F
789.8188	3.07	861	Precursor

Identified peptide fragment (**2 sites**): NQDTISSKLK (Sequence: AA 266-275, [N266, Q267])

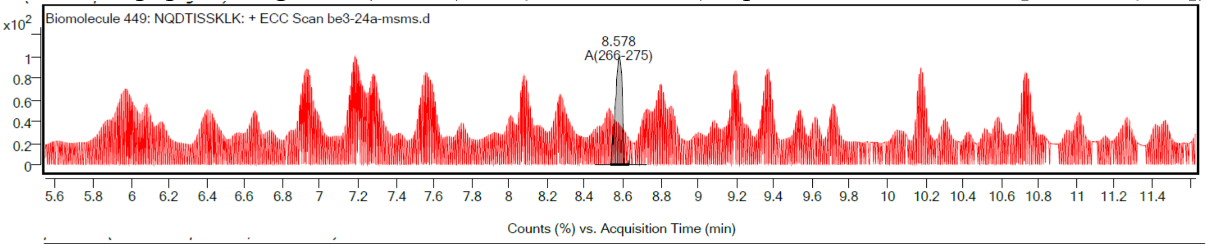

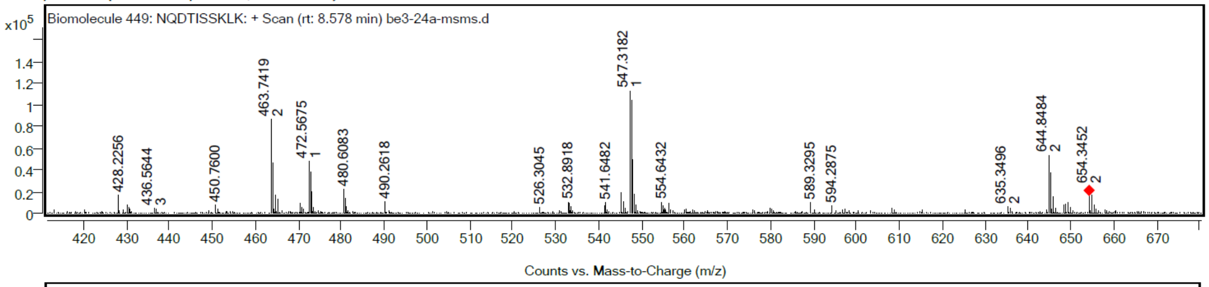

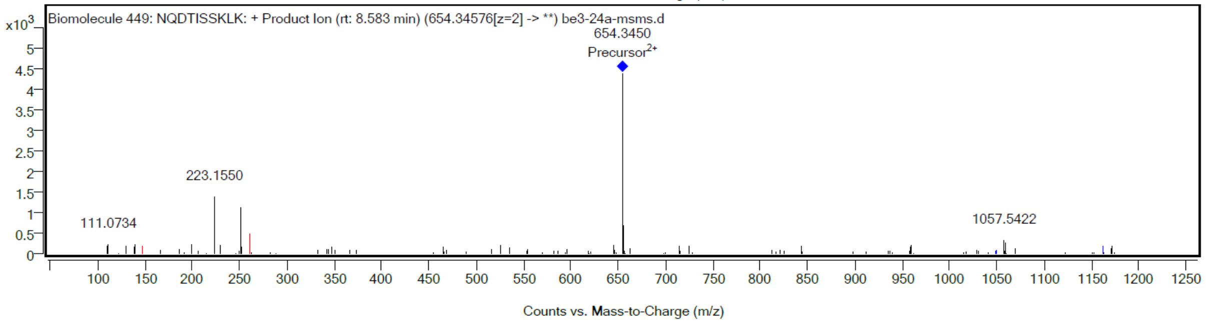

Fragment Spectrum Peaks

m/z	Diff (ppm)	Abund	Ion
147.1106	14.74	188	y1
260.1990	-8.30	476	y2
1049.5025	-0.52	91	b8
1161.5709	10.13	178	b9
654.3450	4.14	4374	Precursor
654.8452	6.38	3494	Precursor
655.3455	8.50	682	Precursor

Identified peptide fragment (**2 sites**): NYQEAKDAFLGS (Sequence: AA 317-328, [N317, Q319])

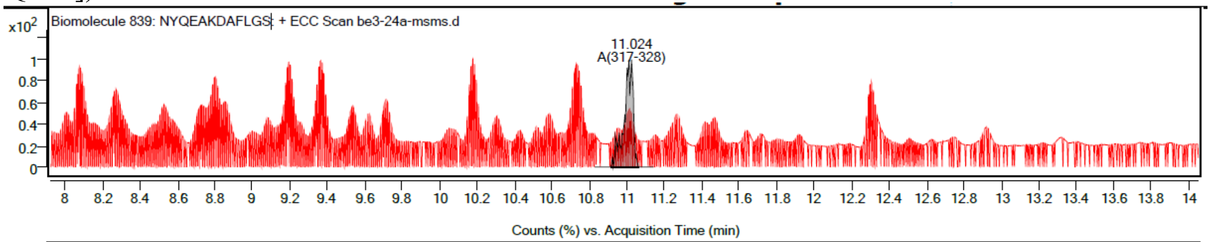

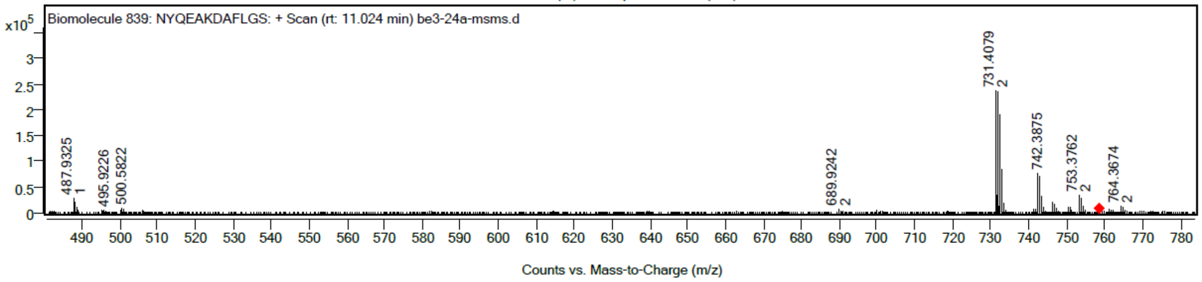

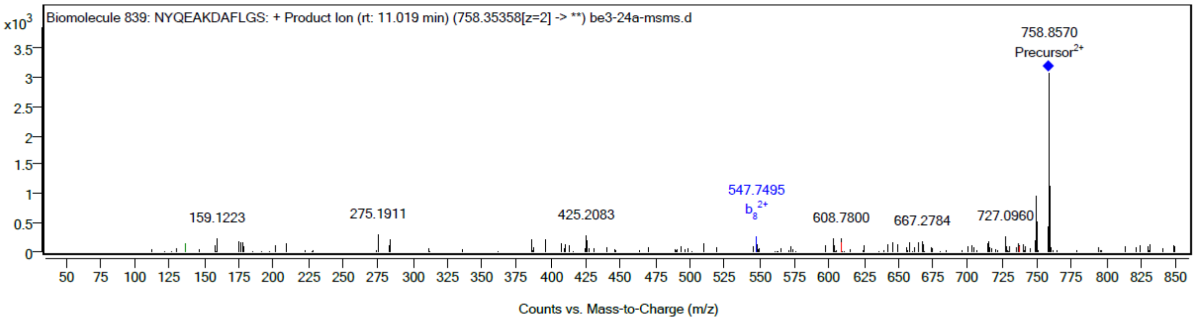

Fragment Spectrum Peaks

m/z	Diff (ppm)	Abund	Ion
609.2729	24.64	151	y6
737.3562	36.18	88	y7
547.7495	-8.01	257	b8
136.0760	-2.21	149	Y
758.8570	-4.80	3059	Precursor
759.3537	1.74	1123	Precursor

Identified peptide fragment (**2 sites**): HLVDEPQNLIKQNCDQFEKLGEYGFQNAL (Sequence: AA 378-406, [N384, Q385])

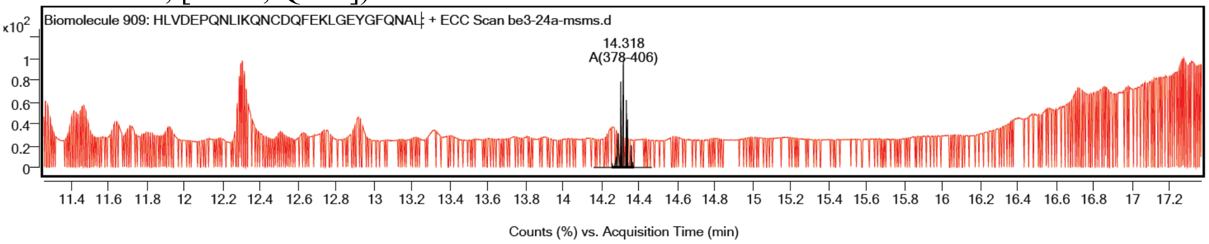

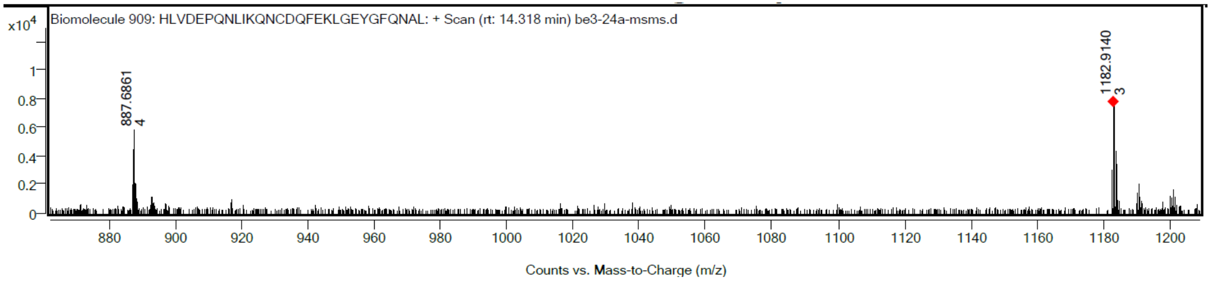

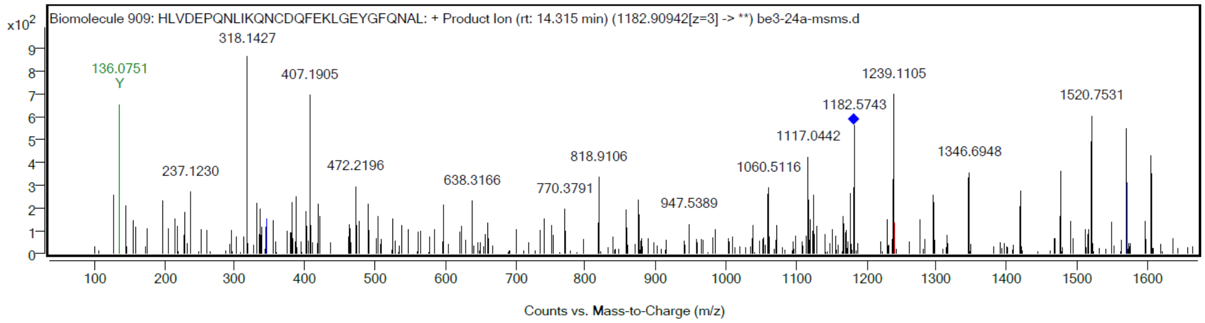

Fragment Spectrum Peaks

m/z	Diff (ppm)	Abund	Ion
1239.6214	12.47	133	y11
1571.7727	33.69	312	b12
346.1713	8.28	151	b6
136.0751	4.53	652	Y

Identified peptide fragment (**1 site**): VMENFVAFVDK (Sequence: AA 546-556, N549)

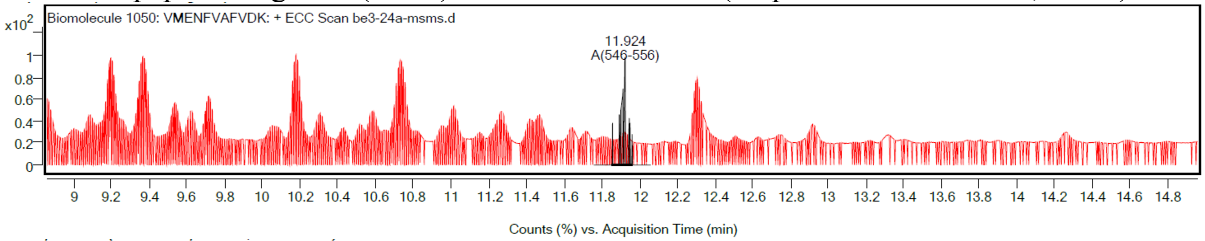

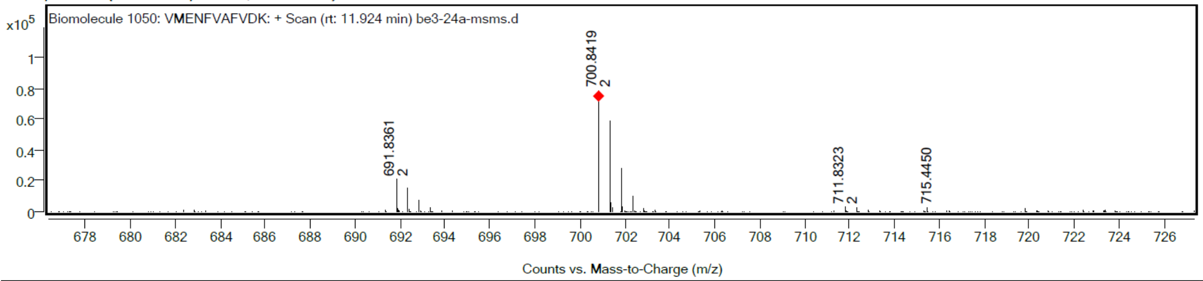

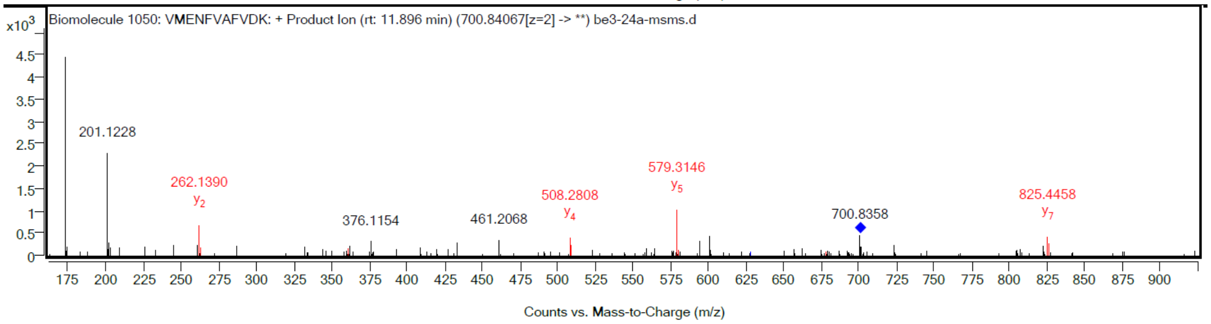

Fragment Spectrum Peaks

m/z	Diff (ppm)	Abund	Ion
262.1390	2.85	666	y2
361.2191	-30.14	139	y3
508.2808	-8.21	380	y4
579.3146	-1.58	1000	y5
678.3725	14.14	77	y6
825.4458	5.67	400	y7
628.2834	8.64	74	b10
701.3379	5.22	185	Precursor

Supplementary Fig. 24: Tagging of Boronic Acid Carbometallation Ketone Product with Hydroxylamine-647 Fluorophore in Cell Lysate

Proof of Concept: Dual Modification of H_2N-FRN(Nitrile)FG-CO_2H with Azide Boronic Acid and O-Benzylhydroxylamine

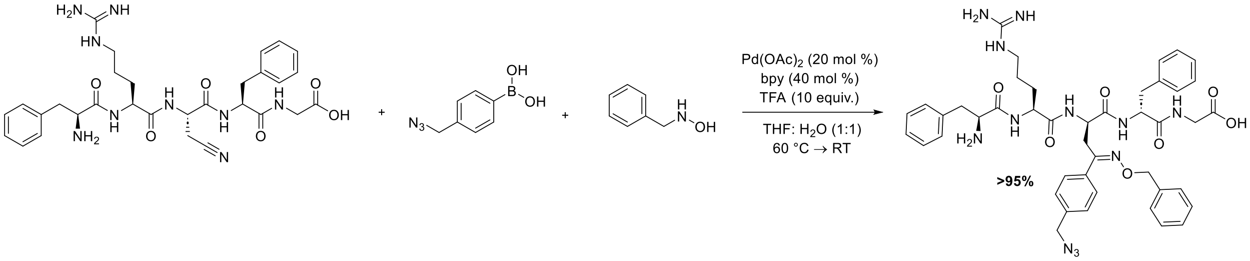

H_2N-FRN(nitrile)FG-CO_2H (**9**) (10.0 mg, 16.1 μmol, 1 equiv.) and 4-azidomethylphenylboronic acid (17.1 mg, 96.5 μmol, 6 equiv.) were added to a 1/2" dram vial. Next, 2,2'-bipyridyl ligand (1.0 mg, 6.4 μmol, 40 mol%) was added from a freshly prepared stock solution (300 μL, 1:1 H_2O:THF). TFA (~20 μL, 161 μmol, 10 equiv.) was then transferred to the solution, after which 2.7 mL 1:1 H_2O:THF was added to bring the total reaction volume to 3 mL. N_2 was bubbled for 2 minutes using an 18G x 1 ½" needle and balloon. Finally, $Pd(OAc)_2$ (1.5 mg, 3.2 μmol, 20 mol%) was added from a freshly prepared stock solution (20 μL, 1:1 H_2O:THF). N_2 was bubbled for another 60 seconds using an 18G x 1 ½" needle and balloon, and then the vial was flushed with N_2. The reaction was left stirring for 12 hours at 60 °C. After this period, the reaction was quenched with 3-MPA (3 μL, 20 equiv.) and ketone carbometallation product was purified via HPLC (>95% conversion using **HPLC Method A**) and lyophilized. Next, the peptide (5 mg, 8.1 μmol) was redissolved in 1:1 H_2O:THF (1 mL) and O-benzylhydroxylamine hydrochloride (25.5 mg, 162 μmol, 20 equiv.) was added to the reaction mixture. The reaction was stirred for an additional 12 hours at RT. The reaction was analyzed via **HPLC Method A** to determine >95% conversion to H_2N-FRN(Dual Modification)FG-CO_2H.

H_2N-FRN(Dual Modification)FG-CO_2H: LCMS, m/z 431.2248 (calcd. $[(M+2H^+)/2]$ = 431.2241, m/z 861.4433 (calcd. $[M+H^+]$ = 861.4482), Purity: >99% (HPLC analysis at 220 nm). Retention time using **HPLC Method A**: 15.1 min.

HPLC Trace of H_2N-FRN(Dual Modification)FG-CO_2H

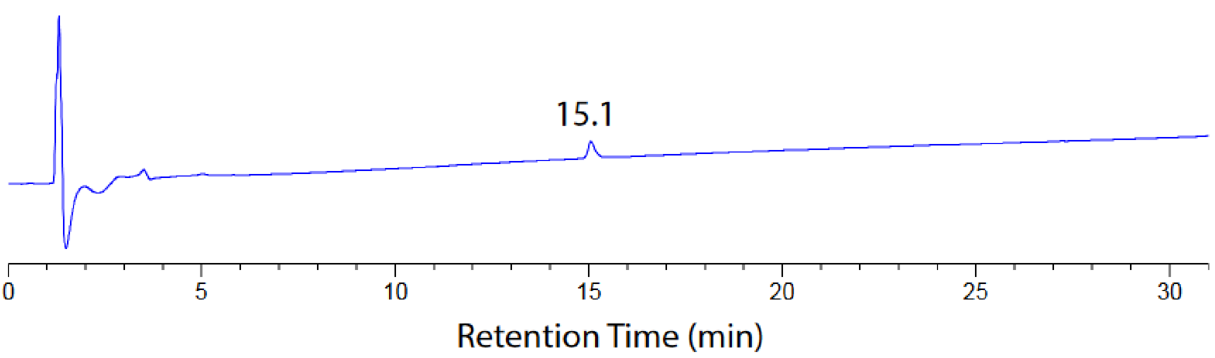

HRMS of H_2N-FRN(Dual Modification)FG-CO_2H

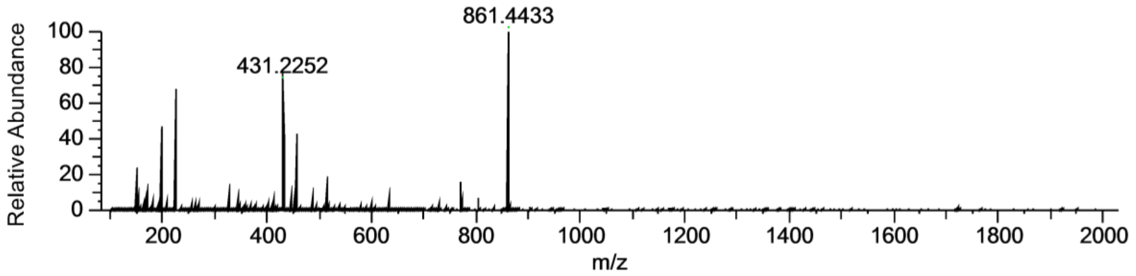

Dual Modification of Cell Lysates with Ketone and O-Benzylhydroxylamine-647 Fluorophore

Dose-dependent boronic acid carbometallation modification of lysates and conjugation with hydroxylamine-647 fluorophore. 3 tubes (individual reactions) of 500 µg of lysate in degassed MeCN:NaP buffer pH 7.4 (1:4, 400 µL) were treated with freshly prepared 250 µM, 500 µM, and 1 mM of $Pd(OAc)_2$ that had been re-suspended in 50 µL of acetonitrile. The reaction was stirred at room temperature for 2 hours. Upon completion of reaction, samples were acetone precipitated, followed by carbometallation treatment. To do the boronic acid modification, nitrile-modified lysates were dissolved in 200 µL of NaP buffer (10 mM, pH 7.4) and 50 µL of THF, followed by addition of 1 µL of TFA. Preformed bipyridine-palladium complex (200 µg of bipyridine and 100 µg of PdOAc dissolved in 50 µL of 1:1 THF and H_2O) was added, and the reaction was stirred at 50 °C for 15 min. 200 µg of 4-methoxyphenylboronic acid in 50 µL of THF was added and the reaction was stirred for 8 hours followed by quenching with 100 µL of 1 M solution of L-cysteine and 5 µL of 1 M NaOH solution. The crude reaction mixture was acetone precipitated to remove the small molecule impurities. Proteins were dissolved in 100 µL of water, followed by the addition of 50 µL of 100 µM solution of hydroxylamine-647 fluorophore. The reaction was stirred for 2 hours and acetone precipitated, followed by analysis of proteins through in gel fluorescence imaging and Coomassie blue staining. Samples were loaded on a Novex WedgeWell 4-20% Tris-Glycine gel. Gel was run in Tris-glycine running buffer at 180V. The gel was then stained with Coomassie brilliant blue for 1 hour and destained overnight.

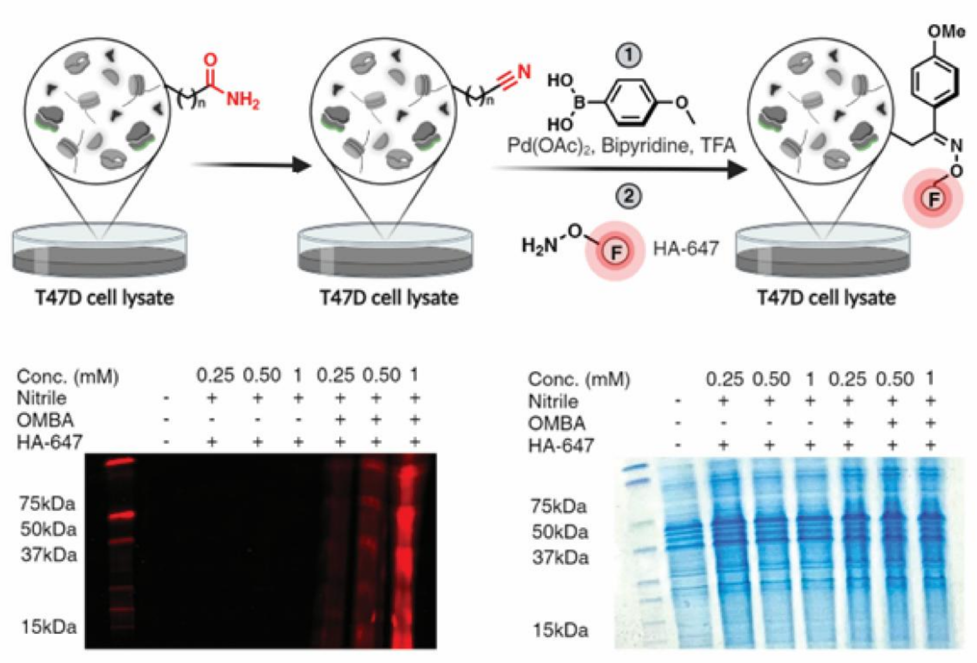

Supplementary Fig. 25: Boronic Acid Carbometallation for Synthesis of Trastuzumab-Fluorophore Conjugate

Nitrile Dehydration Using Trastuzumab

500 µg of Trastuzumab (8.6 µM) in degassed ACN:H_2O (1:4, 400 µL) was treated with freshly prepared 500 µM of $Pd(OAc)_2$ that had been re-suspended in 50 µL of acetonitrile. The reaction was stirred at room temperature for 1 hour followed by quenching with 200 µL of 1 M solution of L-cysteine and 10 µL of 1 M NaOH solution. The crude reaction mixture was passed through Amicon Ultra 3 kDa spin-concentrator and washed with H_2O (7×0.5 mL) to remove the small molecule impurities. The labeled protein was lyophilized, redissolved in 0.1% formic acid in H_2O and analyzed using LC-MS/MS.

MS/MS Analysis of Digested Modified Trastuzumab

Identified peptide fragment: HNHYTQKSLSLSPGDIQMTQSPSSLSASVGDR (Sequence: AA 436-467, [N437, Q441])

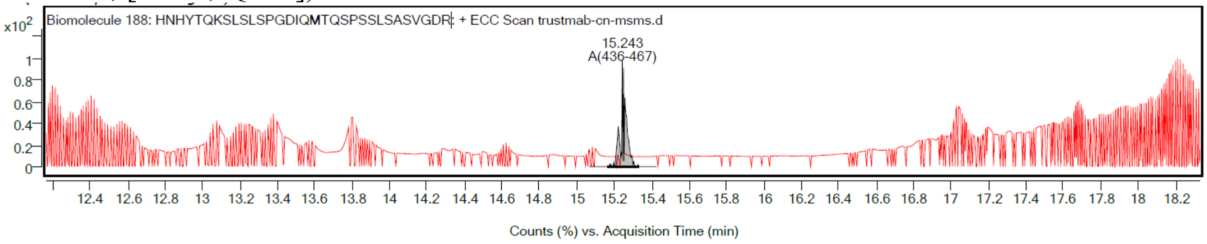

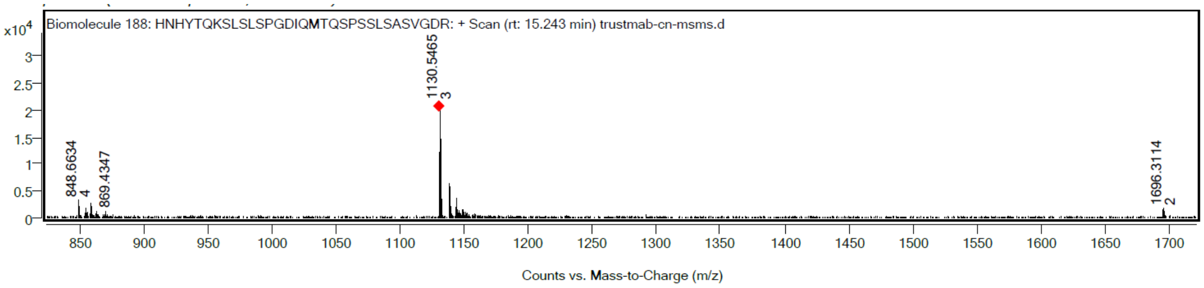

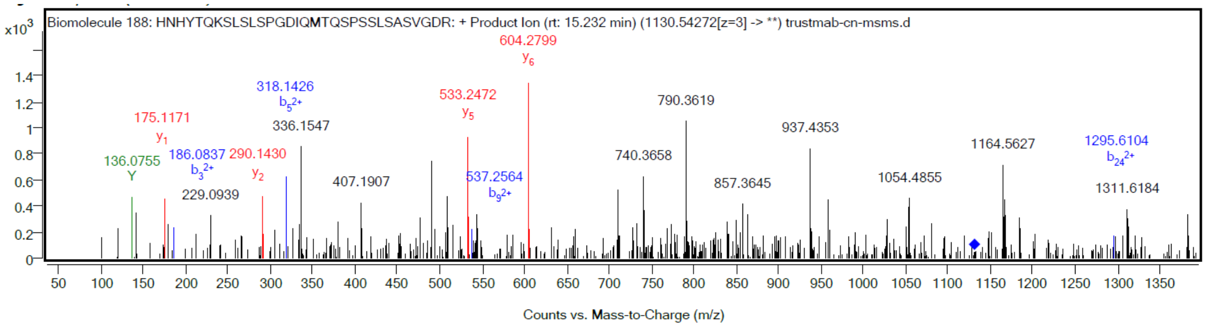

Fragment Spectrum Peaks

m/z	Diff (ppm)	Abund	Ion
175.1171	10.42	455	y1
290.1430	9.93	474	y2
533.2472	38.71	923	y5
604.2799	41.33	1342	y6
186.0837	-7.20	231	b3
318.1426	-14.89	625	b5
537.2564	20.51	220	b9
1295.6104	1.88	172	b24
136.0755	1.41	467	Y

Identified peptide fragment: VYACEVTHQGLSSPVTK (Sequence: AA 640-656, Q648)

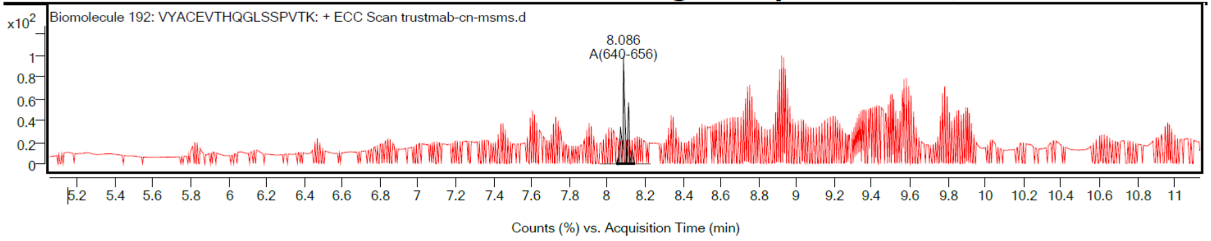

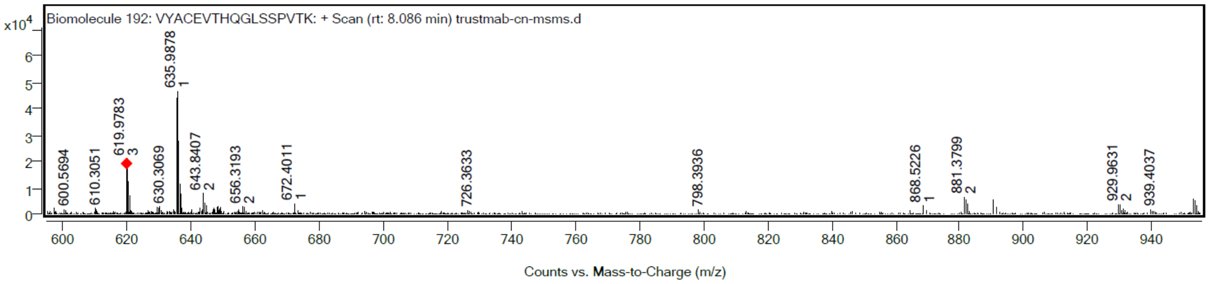

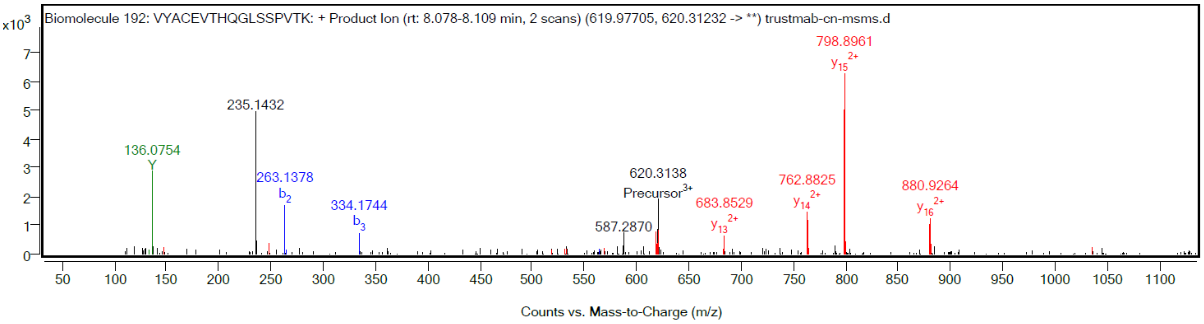

Fragment Spectrum Peaks

m/z	Diff (ppm)	Abund	Ion
147.1130	-1.47	249	y1
248.1594	4.55	389	y2
531.3110	4.99	178	y5
1035.5547	3.32	231	y10
518.2802	4.80	150	y10
568.8102	-6.39	215	y11
620.3477	-0.34	846	y12
683.8529	18.32	641	y13
762.8825	-6.68	1467	y14
798.8961	1.95	6289	y15
880.9264	5.23	1244	y16
263.1378	4.82	1694	b2
334.1744	5.24	733	b3
564.2415	16.13	171	b10
133.0400	22.49	153	C
136.0754	2.20	2892	Y
619.9781	-1.84	857	Precursor
620.3138	-3.78	1935	Precursor
620.6408	8.17	1243	Precursor
620.9749	8.78	198	Precursor

Boronic Acid Carbometallation on Nitrile-Modified Trastuzumab

500 µg of nitrile-modified Trastuzumab (6.9 µM final concentration) was dissolved in 300 µL of degassed H_2O, followed by addition of 1 µL of TFA. 200 µg of 4-methoxyphenylboronic acid in 50 µL of THF was then added. Preformed bipyridine-palladium complex (200 µg of bipyridine and 100 µg of $Pd(OAc)_2$ dissolved in 100 µL of 1:1 THF:H_2O) was added, and the reaction was stirred at 50 °C for 8 hours followed by quenching with 200 µL of 1 M solution of L-cysteine and 5 µL of 1 M NaOH solution. The crude reaction mixture was passed through Amicon Ultra 3 kDa spin-concentrator and washed with H_2O (7×0.5 mL) to remove the small molecule impurities. The labeled protein was lyophilized, redissolved in 0.1% formic acid in H_2O and analyzed using LC-MS/MS.

MS/MS Analysis of Digested Modified Trastuzumab

Identified peptide fragment: GNVFSCSVMHEALHNHYTQK (Sequence: AA 423-442, [N437, Q441])

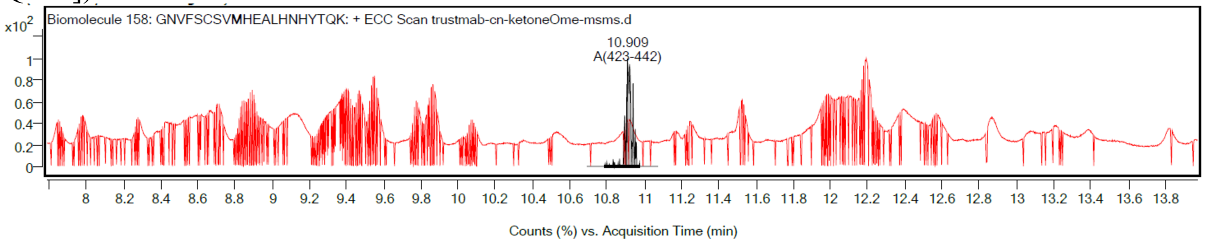

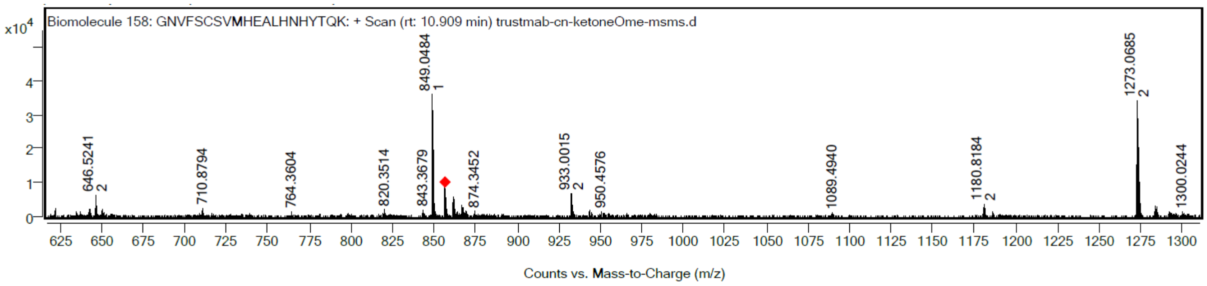

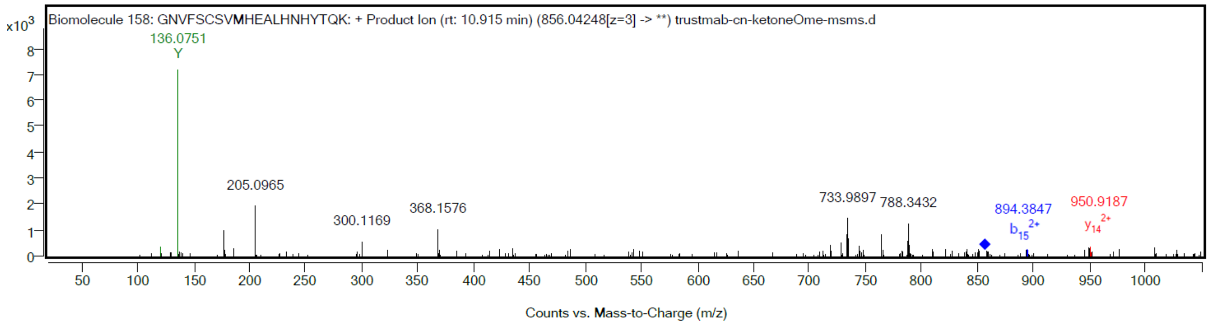

F

Fragment Spectrum Peaks

m/z	Diff (ppm)	Abund	Ion
950.9187	20.32	329	y14
894.3847	-5.85	234	b15
120.0802	4.86	345	F
136.0751	4.22	7160	Y

Identified peptide fragment: ADYEKHKVYACEVTHQG (Sequence: AA 633-649, Q648)

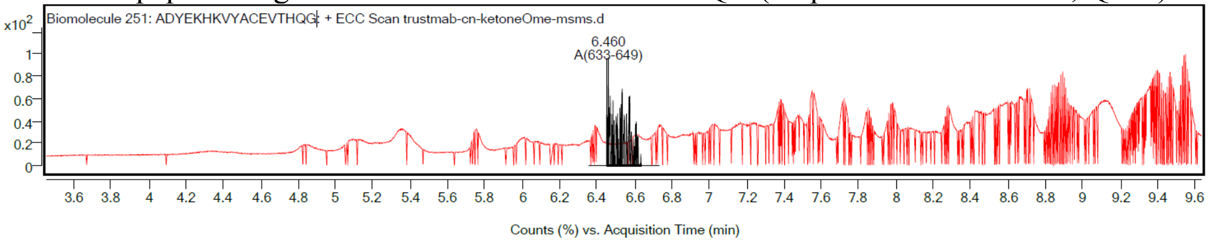

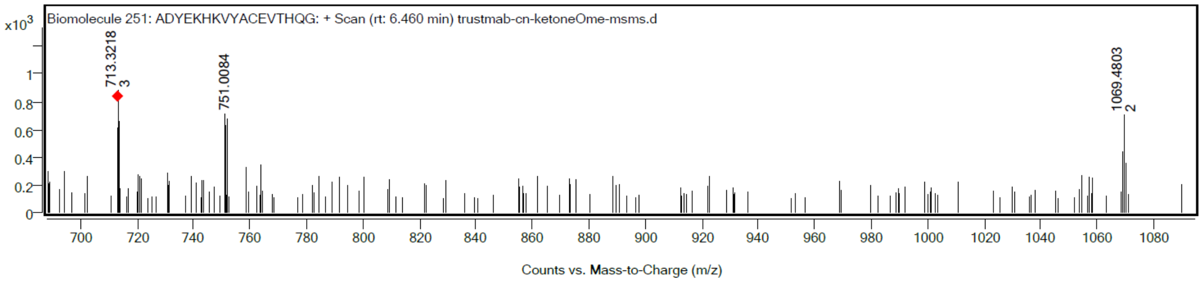

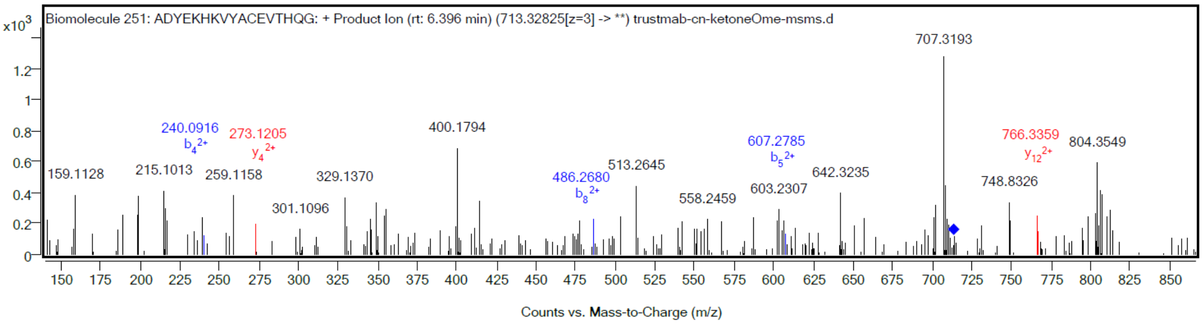

Fragment Spectrum Peaks

m/z	Diff (ppm)	Abund	Ion
273.1205	3.24	196	y4
766.3359	23.01	246	y12
607.2785	-10.31	131	b5
240.0916	2.71	122	b4
486.2680	-35.28	224	b8

Attachment of Fluorophore to Ketone-Modified Trastuzumab: Synthesis of a Trastuzumab-Fluorophore Conjugate

Ketone-modified Trastuzumab was dissolved in 100 µL of water, followed by the addition of 50 µL of 100 µM solution of hydroxylamine-647 fluorophore. The reaction was stirred for 2 h and passed through Amicon Ultra 3 kDa spin-concentrator and washed with H_2O (7×0.5 mL) to remove residual fluorophore. The fluorophore-labeled Trastuzumab was lyophilized and samples were loaded on a Novex WedgeWell 4-20% Tris-Glycine gel. The gel was run in Tris-glycine running buffer at 180V for 75 minutes. The gel was then imaged in a an iBright gel imager to visualize fluorescence, followed fixation for 30 min, stanning with Coomassie brilliant blue for 1 h, and destained overnight. The gel was again imaged for Coomassie. Two bands can be seen in the Coomassie, likely attributable to mono and dual addition of hydroxylamine-AlexaFluor647.

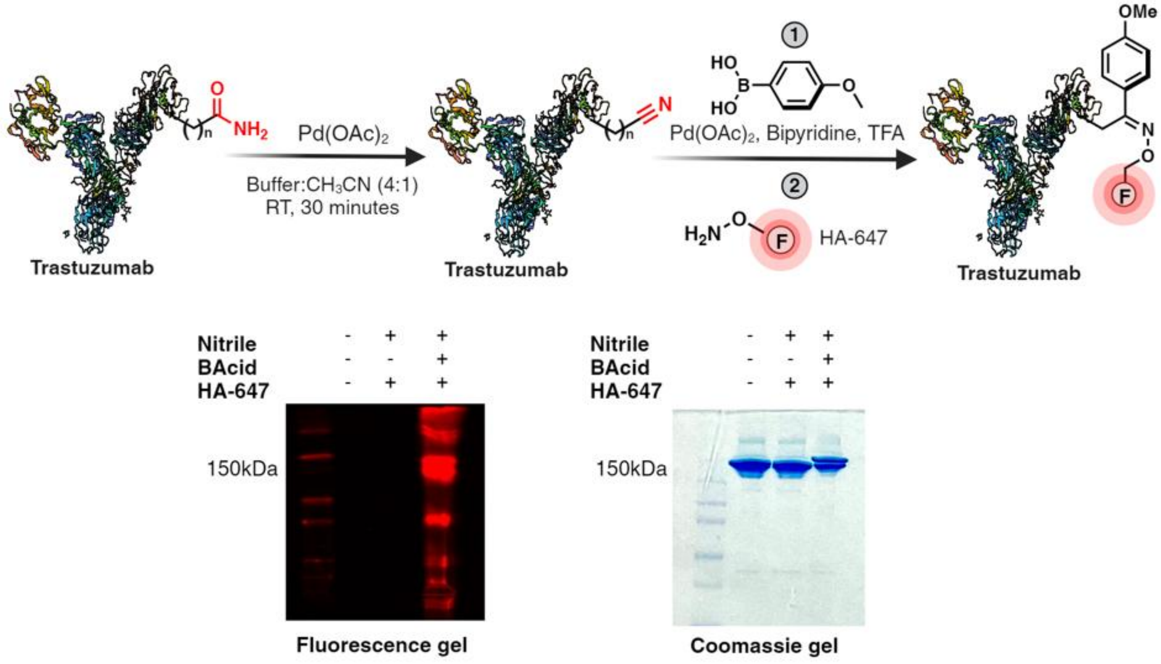

Supplementary Fig. 26: Immunofluorescence Microscopy Imaging Cells with Trastuzumab Fluorophore Conjugate

Live BT-474 and MCF 10A cells were plated on a glass bottomed 8-well plate at a density of 25,000 cells per well in media (RPMI 1640 for BT-474 and DMEM-F12 (1:1) for MCF 10A) and allowed to adhere overnight in an incubator at 37 °C, 5% CO_2. Cells were incubated with 300 µL of 10 µM of Trastuzumab-fluorophore conjugate in media for 1 hour then washed with 300 µL

PBS for 5 min (repeated 3 times). Cells were stained with Cell Mask (ThermoFischer, C37608) was added according to manufacturer protocol, and incubated for 10 min. Cells were subsequently washed with 300 µL PBS for 5 min (repeated 3 times). Then cells were stained with 300 µL of 1 µg/mL Hoechst for 5 min. Cells were subsequently washed with 300 µL PBS for 5 min (repeated 3 times). 300 µL of fresh media was added and cells were imaged on a Stellaris 8 Leica DMi8 microscope (20x objective) with fast lifetime contrast (FALCON) module. Samples were excited using a 40 MHz pulsed white light laser tuned to 405 (Hoescht), 522 (Cell Mask), and 647 (AlexaFluor647) nm with sequential acquisition. Emitted photons were detected using HyD® S and HyD. X (GaAsP hybrid photocathode). Experiment was repeated with 3 biological replicates on 3 separate days with different passage numbers of cells with similar imaging results. Images were processed and analyzed using ImageJ software.

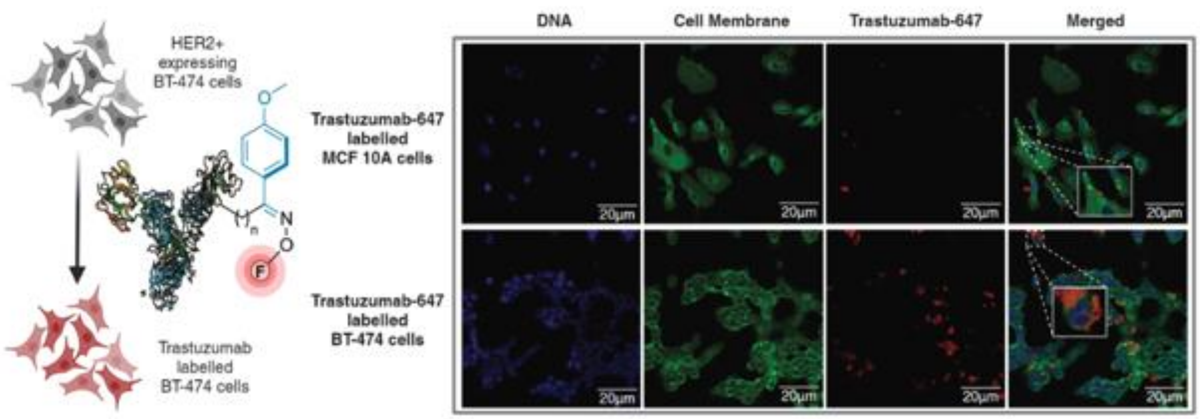

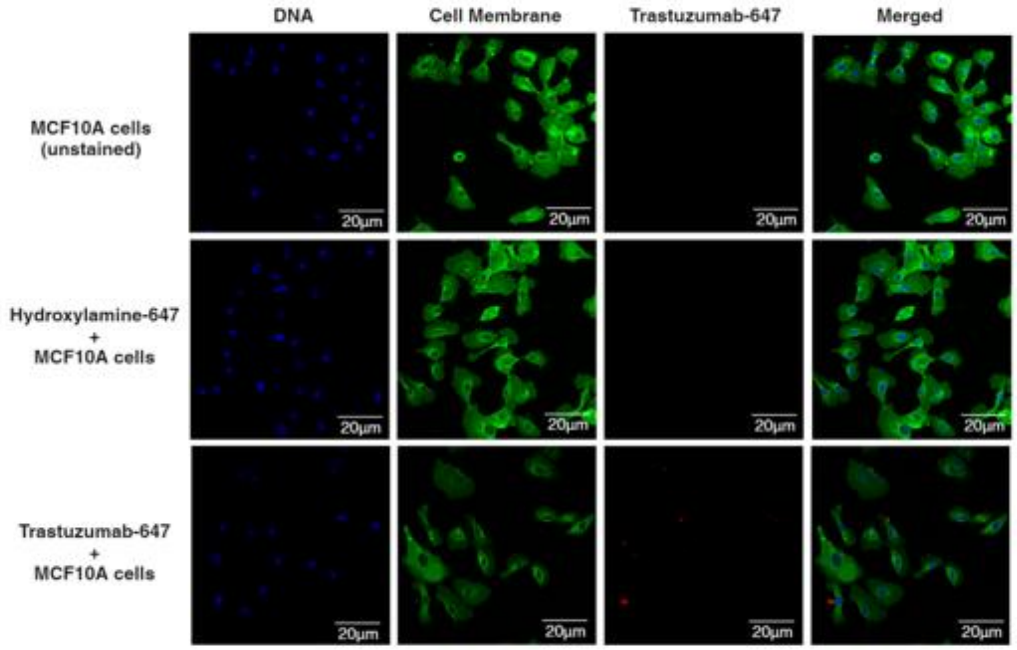

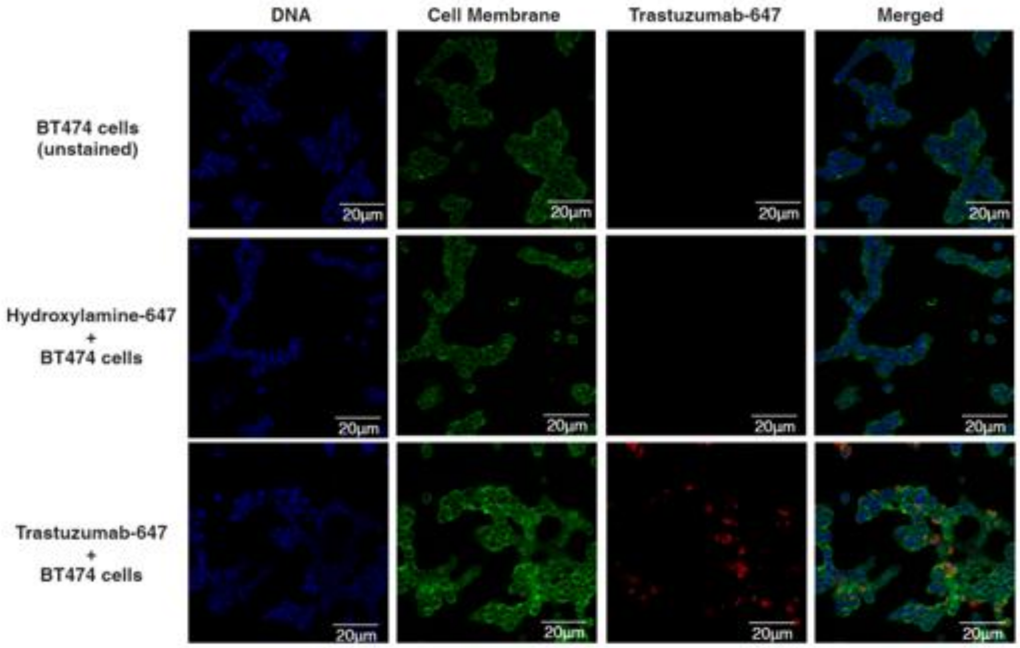

References

1. W. C. Chan, P. D. White, *Fmoc Solid Phase Peptide Synthesis: A Practical Approach*, Oxford Univ. Press, New York, **2000**.
2. X. Wang, X. Wang, M. Liu, J. Ding, J. Chen, H. Wu, "Palladium-Catalyzed Reactions of Arylboronic Acids with Aliphatic Nitriles: Synthesis of Alkyl Aryl Ketones and 2-Arylbenzofurans Synthesis of Alkyl Aryl Ketones from Nitriles," *Synthesis* **2013**, *45*, 2241.
3. Z. E. Paikin, J. M. Talbott, M. Raj, "Regioselective Aldehyde Decarbonylation through Palladium-Catalyzed Nitrile Boronic Acid Cross-Coupling," *Synlett* **2024**, *35*, 1924.
4. J. Weiser, P. S. Shenkin, W. C. Still, "Approximate atomic surfaces from linear combinations of pairwise overlaps (LCPO)," *J. Comput. Chem.* **1999**, *20*, 217.
5. Y.-S. Gao, J.-T. Su, Y.-B. Yan, "Sequential Events in the Irreversible Thermal Denaturation of Human Brain-Type Creatine Kinase by Spectroscopic Methods," *Int. J. Mol. Sci.* **2010**, *11*, 2584.
